# Supplementary material for: Triangulation supports agricultural spread of the Transeurasian languages
Source: Nature. 2021 Nov 10;599(7886):616–21. doi: 10.1038/s41586-021-04108-8 (PMC8612925; doi:10.1038/s41586-021-04108-8)
Supplement: Supplementary file 1 — This file contains a full guide to Supplementary Data Files 1–26. Note that Supplementary Data Files 3 and 21 are hosted externally; please refer to the links within this Supplementary Guide file for details. [file 41586_2021_4108_MOESM1_ESM.pdf]

nature portfolio

https://doi.org/10.1038/s41586-021-04108-8

Supplementary information

Triangulation supports agricultural spread of the Transeurasian languages

In the format provided by the authors and unedited

Supplementary Information Guide

Triangulation supports agricultural spread of the Transeurasian languages

Martine Robbeets[1]*, Remco Bouckaert[1,2], Matthew Conte[6], Alexander Savelyev[3,1], Tao Li[7,1,35], Deog-Im An [31], Ken-ichi Shinoda[11], Yinqiu Cui[18,19], Takamune Kawashima[8], Geonyoung Kim[6], Junzo Uchiyama[9,10], Joanna Dolińska[1], Sofia Oskolskaya[4,1], Ken-Yōjiro Yamano[17], Noriko Seguchi[12,13], Hirotaka Tomita[14,15], Hiroto Takamiya[16], Hideaki Kanzawa-Kiriyama[11], Hiroki Oota[20], Hajime Ishida[22], Ryosuke Kimura[22], Takehiro Sato[21], Jae-Hyun Kim[32], Bingcong Deng[1], Rasmus Bjørn[1], Seongha Rhee[5], Kyou-Dong Ahn[5], Ilya Gruntov[3,30], Olga Mazo[30,3], John R. Bentley[23], Ricardo Fernandes[1,29,34], Patrick Roberts[1], Ilona R. Bausch[26,27,28], Linda Gilaizeau[1], Minoru Yoneda[25], Mitsugu Kugai[33], Raffaela A. Bianco[1], Fan Zhang[18], Marie Himmel[1], Mark J. Hudson[1,24]*, Ning Chao[1,36]*
*corresponding authors

1 Max Planck Institute for the Science of Human History, Jena, Germany
2 Centre of Computational Evolution, University of Auckland, Auckland, New Zealand
3 Institute of Linguistics, Russian Academy of Sciences, Moscow, Russia
4 Institute for Linguistic Studies, Russian Academy of Sciences, Saint Petersburg, Russia
5 Hankuk University of Foreign Studies, Seoul, South Korea
6 Department of Archaeology and Art History, Seoul National University, Seoul, South Korea
7 Department of Archaeology, Wuhan University, Wuhan, China
8 Hiroshima University Museum, Higashi-Hiroshima, Japan
9 Sainsbury Institute for the Study of Japanese Arts and Cultures, Norwich, UK
10 Center for Cultural Resource Studies, Kanazawa University, Japan
11 National Museum of Nature and Science, Department of Anthropology, Tsukuba, Japan
12 Department of Environmental Changes, Faculty of Social and Cultural Studies, Kyushu University, Fukuoka, Japan
13 Department of Anthropology, The University of Montana, Missoula, MT, USA
14 Hokkaido Government Board of Education, Sapporo, Japan
15 Graduate School of Integrated Sciences of Global Society, Kyushu University, Fukuoka, Japan
16 Research Center for the Pacific Islands, Kagoshima University, Kagoshima, Japan
17 Research Center for Buried Cultural Properties, Kumamoto University, Japan
18 School of Life Sciences, Jilin University, China
19 Center for Chinese Frontier Archaeology, Jilin University, China
20 Department of Biological Sciences, Graduate School of Science, The University of Tokyo, Tokyo, Japan
21 Department of Bioinformatics and Genomics, Graduate School of Medical Sciences, Kanazawa University, Kanazawa, Japan
22 Graduate School of Medicine, University of the Ryukyus, Nishihara, Japan
23 Department of World Languages and Cultures, Northern Illinois University, USA
24 Institut d'Asie Orientale, ENS de Lyon, France
25 University Museum, University of Tokyo, Japan
26 Leiden University Institute of Area Studies, Netherlands
27 Sainsbury Institute for the Study of Japanese Arts and Cultures, Norwich, UK
28 Kokugakuin University Museum, Tokyo, Japan
29 Faculty of Arts, Masaryk University, Brno, Czech Republic.
30 National Research University Higher School of Economics, Moscow, Russia
31 Department of Conservation of Cultural Heritage, Hanseo University, Seosan, Korea
32 Department of Archaeology and Art History, Donga University, Korea
33 Miyakojima City Board of Education, Miyako Island, Okinawa, Japan
34 School of Archaeology, University of Oxford, UK.
35 Archaeological Institute for Yangtze Civilization, Wuhan University, China.
36 School of Archaeology and Museology, Peking University, Beijing, China

Supplementary information legends

Supplementary Data File 1: Comparative dataset including 3193 cognate sets representing 254 basic vocabulary concepts for 98 Transeurasian languages.

Filename:16_Eurasia3angle_synthesis_SI 1_BV 254.xls

Supplementary Data File 2: Basic vocabulary etymologies across the Transeurasian languages, underlying semantically equivalent cognate sets scored as (1) in Supplemenary Data File 1

Filename: 17_ Eurasia3angle_synthesis_SI 2_basic etymologies.doc

Supplementary Data File 3: Bayesian phylogeographic analysis modelling the spatiotemporal expansion of the Transeurasian languages - **This file is hosted externally at the following links:**

https://figshare.com/s/b9c67ca3ea47faf51d48

https://github.com/rbouckaert/Eurasia3angle

Supplementary Data File 4: Integration of qualitative assessment methods and Bayesian phylogeography in identifying the ancestral homelands of Transeurasian

Filename: 19_Eurasia3angle_synthesis_SI 4_homelands.docx

Supplementary Data File 5: Inherited and borrowed correspondence sets for agropastoral vocabulary across the Transeurasian languages.

Filename: 20_Eurasia3angle_synthesis_SI 5_subsistence.docx

Supplementary Data File 6: Archaeological database.

Filename: 26_Eurasia3angle_synthesis_SI 6_E3a Matrix.xls

Supplementary Data File 7: Qualitative analysis of the archaeological database.

Filename: 27_Eurasia3angle_synthesis_SI 7_qualitative analysis_REV30.08.pdf

Supplementary Data File 8: Interpretation of our Bayesian phylogenetic analysis of the archaeological database in Supplementary Data File 25.

Filename: 28_Eurasia3angle_synthesis_SI 8_Bayesian cultural interpretation_REV01.10.pdf

Supplementary Data File 9: Early crop remains with direct C14 dates from Northeast Asia. Compiled from published sources and from the radiocarbon database of the National Museum of Japanese History. Radiocarbon dates on rice from the Nabatake site (Saga) are omitted since several of the results from that site published in the early 1980s appear unreliable.

Filename: 29_Eurasia3angle_synthesis_SI 9_cerealC14_REV07.07

Supplementary Data File 10: List of abbreviations used for present-day Eurasian populations.

Filename: 30_Eurasia3angle_synthesis_SI 10_abbreviations.xlsx

Supplementary Data File 11: Sample information for newly-generated ancient DNA data and for co-analyses of published ancient individuals from East Eurasia.

Filename: 31_Eurasia3angle_synthesis_SI 11_aDNA sample info.xlsx

Supplementary Data File 12: Archaeological context for ancient DNA samples used in this study.

Filename: 32_Eurasia3angle_synthesis_SI 12_site info_REV01.10.docx

Supplementary Data File 13: Archaeolinguistic interpretation of our ancient DNA analyses

Filename: 33_Eurasia3angle_synthesis_SI 13_Archaeogenetic interpretation_REV21.09.pdf

Supplementary Data File 14: Inventory of excavated skeletal remains from Nagabaka.

Filename: 34_Eurasia3angle_synthesis_SI 14_Nagabaka skeletal.xlsx

Supplementary Data File 15: Isotope analyses of the key samples included in this study.

Filename: 35_Eurasia3angle_synthesis_SI 15_isotope.pdf

Supplementary Data File 16: qpAdm admixture modelling of modern Koreans as a result of admixture between lineages related to Jomon and mainland East Asians from the '1240k-Illumina' datasets.

Filename: 36_Eurasia3angle_synthesis_SI 16_qpAdm_REV07.07.pdf

Supplementary Data File 17: Sequencing details and summary of newly generated aDNA from this study.

Filename: 37_Eurasia3angle_synthesis_SI17_Sequencing details_REV07.07.xlsx

Supplementary Data File 18: Bayesian phylogenetic analysis of the linguistic dataset.

Filename: 38_Eurasia3angle_synthesis_SI 18_Bayesian linguistics_REV07.07.pdf

Supplementary Data File 19: BEAST XML files specifying the models, priors, hyperpriors and settings used to run the analyses of the linguistic database.

Filename: 39_Eurasia3angle_synthesis_SI 19_XML files_README_REV21.09.docx

Full versions of these files are hosted externally at the following links:

https://figshare.com/s/748bf751fe3ba7752046

https://github.com/rbouckaert/Eurasia3angle

Supplementary Data File 20: Bayesian phylogenetic analysis of the archaeological dataset.

Filename: 40_Eurasia3angle_synthesis_SI 20_Bayesian archaeology.pdf

Supplementary Data File 21: BEAST XML files specifying the models, priors, hyperpriors and settings used to run the analyses of the archaeological database

These files are hosted externally at the following links:

https://figshare.com/s/99f5aab9a2e43eb2ffd4

https://github.com/rbouckaert/Eurasia3angle

Supplementary Data File 22: Results of filtering contaminated samples included in this study, using qpAdm contamination control.

Filename: 42_Eurasia3angle_synthesis_SI22_aKorean_qpAdm conTam_REV07.07.xlsx

Supplementary Data File 23: Agropastoral vocabulary shared by the Turkic, Mongolic, Tungusic, Koreanic and Japonic languages.

Filename: 21_SI 23_agropastoral.xlsx

Supplementary Data File 24: Dated Bayesian phylogeny of the Transeurasian languages.

Filename: 43_Eurasia3angle_synthesis_SI 24_language phylogeny.pdf

Full versions of this file are hosted externally at the following links:

https://figshare.com/s/709f239fa45982911b87

https://github.com/rbouckaert/Eurasia3angle

The link to the figtree application is: https://github.com/rambaut/figtree/releases/tag/v1.4.3

Supplementary Data File 25: Bayesian phylogenetic analysis of the archaeological database.

Filename: 44_Eurasia3angle_synthesis_SI 25_cultural phylogeny.pdf

Full versions of this file are hosted externally at the following links:

https://figshare.com/s/65615dddc0817bc0184f

https://github.com/rbouckaert/Eurasia3angle

The link to the figtree application is: https://github.com/rambaut/figtree/releases/tag/v1.4.3

Supplementary Data File 26: Triangulation of linguistic, archaeological and genetic evidence with regard to spatiotemporal and subsistence patterns in Northeast Asia in the Neolithic and Bronze Age.

Filename: 15_Eurasia3angle_synthesis_ SI 26_triangulation_REV21.09.docx
