# Supplementary material for: Triangulation supports agricultural spread of the Transeurasian languages
Source: Nature. 2021 Nov 10;599(7886):616–21. doi: 10.1038/s41586-021-04108-8 (PMC8612925; doi:10.1038/s41586-021-04108-8)
Supplement: Supplementary file 2 — Reporting Summary [file 41586_2021_4108_MOESM2_ESM.pdf]

nature research

Corresponding author(s): Martine Robbeets, Mark Hudson and Chao Ning.

Last updated by author(s): 2021.08.30

Reporting Summary

Nature Research wishes to improve the reproducibility of the work that we publish. This form provides structure for consistency and transparency in reporting. For further information on Nature Research policies, see our Editorial Policies and the Editorial Policy Checklist.

Statistics

For all statistical analyses, confirm that the following items are present in the figure legend, table legend, main text, or Methods section.

n/a	Confirmed	
☐	☒	The exact sample size (*n*) for each experimental group/condition, given as a discrete number and unit of measurement
☐	☒	A statement on whether measurements were taken from distinct samples or whether the same sample was measured repeatedly
☐	☒	The statistical test(s) used AND whether they are one- or two-sided<br>*Only common tests should be described solely by name; describe more complex techniques in the Methods section.*
☒	☐	A description of all covariates tested
☐	☒	A description of any assumptions or corrections, such as tests of normality and adjustment for multiple comparisons
☒	☐	A full description of the statistical parameters including central tendency (e.g. means) or other basic estimates (e.g. regression coefficient) AND variation (e.g. standard deviation) or associated estimates of uncertainty (e.g. confidence intervals)
☐	☒	For null hypothesis testing, the test statistic (e.g. *F*, *t*, *r*) with confidence intervals, effect sizes, degrees of freedom and *P* value noted<br>*Give P values as exact values whenever suitable.*
☐	☒	For Bayesian analysis, information on the choice of priors and Markov chain Monte Carlo settings
☒	☐	For hierarchical and complex designs, identification of the appropriate level for tests and full reporting of outcomes
☒	☐	Estimates of effect sizes (e.g. Cohen's *d*, Pearson's *r*), indicating how they were calculated

Our web collection on statistics for biologists contains articles on many of the points above.

Software and code

Policy information about availability of computer code

Data collection	The code used in the Bayesian analysis of the linguistic and cultural topologies is fully referenced. Readers can access the code underlying our Bayesian analyses of linguistic and cultural datasets through the supplementary information. The files in SI 19 relate to languages and those in SI 21 to cultures; see https://figshare.com/s/748bf751fe3ba7752046 and https://figshare.com/s/99f5aab9a2e43eb2ffd4<br>Illumina sequence data were processed using the following programs to obtain genotype data used in the analysis: EAGER v1.92.55, AdapterRemoval v2.2.0, BWA v0.7.12, DeDup v0.12.2, bamUtils v1.0.13, pileupCaller (https://github.com/stschiff/sequenceTools), mapDamage v2.0.9, ANGSD v0.910, Schmutzi v1.5.1. These programs are publicly available. .
Data analysis	The code used in the Bayesian analysis of the linguistic and cultural topologies is fully referenced. Population genetic data analysis in this study was performed using the following publicly available programs: Smartpca v16000, ADMIXTURE v1.3.0, PLINK v1.90, lcMLkin v0.5.0, qp3Pop v435, qpDstat v755, qpWave v410, qpAdm v810, DataGraph v4.5.1. Non-default parameters used in our analysis are described in the Methods section. The base map in Figure 1 was downloaded from the Nature Earth map dataset (https://www.naturalearthdata.com/), granted for the public domain use and is free for use in any type of project. Calibration of AMS 14C dating results was done by OxCal v4.4, using the IntCal20 database.

For manuscripts utilizing custom algorithms or software that are central to the research but not yet described in published literature, software must be made available to editors and reviewers. We strongly encourage code deposition in a community repository (e.g. GitHub). See the Nature Research guidelines for submitting code & software for further information.

Data

Policy information about availability of data

All manuscripts must include a data availability statement. This statement should provide the following information, where applicable:

- Accession codes, unique identifiers, or web links for publicly available datasets
- A list of figures that have associated raw data
- A description of any restrictions on data availability

All linguistics and archaeological datasets are available through the supplementary information. Files that require applications were uploaded on two external sources, i.e. GitHub (https://github.com/rbouckaert/Eurasia3angle) and FigShare. For our genetic datasets, the DNA sequences reported in this paper have been deposited in the European Nucleotide Archive (ENA) under accession PRJEB46162. Haploid genotype data of ancient individuals in this study on the 1240k panel are available in the EIGENSTRAT format from the following link:https://edmond.mpdl.mpg.de/imeji/collection/59JGAaOpSxRb96Vh

Field-specific reporting

Please select the one below that is the best fit for your research. If you are not sure, read the appropriate sections before making your selection.

☒ Life sciences ☐ Behavioural & social sciences ☐ Ecological, evolutionary & environmental sciences

For a reference copy of the document with all sections, see nature.com/documents/nr-reporting-summary-flat.pdf

Life sciences study design

All studies must disclose on these points even when the disclosure is negative.

Sample size	No sample-size calculation was performed. The study proceeding by attempting to sample ancient DNA from contexts that were not previously analyzed and every new sample contributed meaningful new information. The uncertainties due to limited sample size are clearly indicated when there are concerns.
Data exclusions	Data were excluded for analysis based either on evidence for sample contamination, or low coverage data. We clearly indicate these cases.
Replication	As our study is an evolutionary analysis of language, culture and genes and the evolutionary process only proceeds once, replication was not possible.
Randomization	This is not relevant to our study because we are dealing with an evolutionary process not a human-designed experiment.
Blinding	Blinding was not possible for this study because the analysts needed to understand the historical background of the samples.

Reporting for specific materials, systems and methods

We require information from authors about some types of materials, experimental systems and methods used in many studies. Here, indicate whether each material, system or method listed is relevant to your study. If you are not sure if a list item applies to your research, read the appropriate section before selecting a response.

Materials & experimental systems

n/a	Involved in the study	
☒	☐	Antibodies
☒	☐	Eukaryotic cell lines
☐	☒	Palaeontology and archaeology
☒	☐	Animals and other organisms
☒	☐	Human research participants
☒	☐	Clinical data
☒	☐	Dual use research of concern

Methods

n/a	Involved in the study	
☒	☐	ChIP-seq
☒	☐	Flow cytometry
☒	☐	MRI-based neuroimaging

Palaeontology and Archaeology

Specimen provenance	Skeletal samples newly analysed in the study are under the custodianship of archaeologists or anthropologists in our team who contributed them to the study and whose permission to analyse the samples is indicated through co-authorship of the manuscript.
Specimen deposition	The analyzed samples are under the custodianship of the co-authors who contributed them to the study; the provenance of each sample is described in SI 11 and SI 12. Our co-authors will give access to the parts of the samples remaining after ancient DNA and radiocarbon analysis to anyone who requests it. We also shared photos in SI 13 and commit to sharing more photographic material of skeletal samples before and after sampling.

Dating methods	We dated the root of our linguistic family and the nodes in the family using Bayesian estimation methods, based on calibrating against known time spans provided by dated written records; see Extended Data Fig 1 and BEAST XML files in SI 19.We further report existing radiocarbon dates of archaeological specimens and new radiocarbon dates on bone in this paper; see SI 14 and SI 15.

☒ Tick this box to confirm that the raw and calibrated dates are available in the paper or in Supplementary Information.

Ethics oversight	No ethical approval or guidance was required because we did not perform research on living human participants or animals.

Note that full information on the approval of the study protocol must also be provided in the manuscript.
