# Supplementary material for: Triangulation supports agricultural spread of the Transeurasian languages
Source: Nature. 2021 Nov 10;599(7886):616–21. doi: 10.1038/s41586-021-04108-8 (PMC8612925; doi:10.1038/s41586-021-04108-8)
Supplement: Supplementary file 3 — This zipped file contains Supplementary Data Files 1, 2 and 4–6; see Supplementary Information file for full descriptions (Supplementary Data File 3 is hosted externally; see Supplementary Information file for links). [file 41586_2021_4108_MOESM3_ESM.zip › 2021-02-02920E-s3/17_Eurasia3angle_synthesis_SI 2_basic etymologies.pdf]

Supplementary Information 2

Basic vocabulary etymologies across the Transeurasian languages, underlying semantically equivalent cognate sets scored as (1) in SI 1

1. Methods

In SI 1, we collected a comparative dataset including 3193 datapoints representing 254 basic vocabulary concepts for 98 Transeurasian languages, including contemporary and historical varieties. We established cognacy classes and coded them as present (1) or absent (0) for all languages in the dataset. The determination of cognacy classes is based on the etymologies given in Section 2 below. These etymologies contain more participants than do the cognacy classes in SI 1 because they allow a certain degree of semantic latitude, whereas in S1 only semantically equivalent cognates are scored as (1). All etymologies reflect regular sound correspondences, as listed in Section 3. of this document.

Our 254 basic vocabulary concepts are based on a merger of the Leipzig-Jakarta 200 list (Haspelmath and Tadmor 2009) and the Jena 200 list (Anderson and Heggarty n.d.). The underlying Leipzig-Jakarta 200 list is reduced to 195 items due to the merger of a few meanings (e.g., ‘breast’ and ‘chest’). It is supplemented by the Jena 200 list, which is an updated version of the Swadesh 200 list, currently applied to a comparison of Indo-European languages in the CoBL (Cognacy in Basic Lexicon) project by Anderson and Heggarty. Given the large overlap between the two lists, the final list amounts to 254 concepts.

Compared to the traditional Swadesh list, which is mainly based on intuition, the Leipzig-Jakarta list takes a more systematic and empirical approach to the basic vocabulary because it is based on a quantitative comparison of stable words in the languages across the world. The strength of basic vocabulary is not in the stability of a single concept, but in the overall stability of the body of concepts as a whole.

2. Etymologies

1. FIRE

pJ *_pɨ(r)i_ ‘fire’: J _hi_ (1.3b), OJ _pi_$_2$ ‘fire’, OJ _po_- ‘fire’ in OJ _potaru_ ‘firefly’, OJ _pokusi_ ‘bonfire’, OJ _pokuso_ ‘tinder’, OJ _potopor_- ‘get heated’, OJ _poter_- ‘flush, be all aglow’ etc.; Yonamine (Okinawa) _πii_, Shuri (Okinawa) _fii_ [hwii], Ishigaki (Yaeyama) _pïï_ (B), Hatoma (Yaeyama) _pii_, Yonaguni _cii_ (B) ‘fire’
pK *_pɨl_ ‘fire’: K _pul_, MK _·pul_ ‘fire’

Frellesvig and Whitman (2008) have proposed to add two mid vowels (*_e_, *_o_) and a high central vowel (*_ɨ_) to the four vowels (*_i_, *_a_, *_u_, *_ə_), traditionally reconstructed for proto-Japonic. According to their analysis, apophony of OJ _o_ and _i_$_2$ reflects a contraction whereby *_ɨi_ > OJ _i_$_2$. The alternation between OJ _po_- and OJ _pi_$_2$ ‘fire’ thus reflects pJ *_pɨi_ ‘fire’. Comparison with the final liquid in MK _·pul_ ‘fire’ indicates that

the proto-Japonic diphtong is the result of liquid loss in an earlier pJ **pɨ(r)i* ‘fire’. The word for 'fire' is also reflected in Ryukyuan languages.

3. TO GO

pJ **na*- ‘to go out, become’: OJ *in*- ‘to go away, leave, depart’ A, OJ -*in*- perfective auxiliary, J *nar*- B, OJ *nar*- ‘to become, come into being’, J *nas*- B, OJ *nas*- ‘to make, do, give birth to’
pK **na*- ‘to go out’: K *na*-, MK ·*na*- ‘to go out, emerge, leave, become, come into being, come out’, MK ¨*nay*- ‘to take out, produce’ (*-*i*- causative), MK *nat*- ‘to appear’ (*-*t(i)*- passive), MK -·*na*- resultative
pTg **na*:- ‘to go out’: Evk., Even. -*na*:-, Sol. -*na*:-, Neg. -*na*-, Na. -*nda*-, Olch. -*ŋda*-, Oroch, Orok, Ud. -*na*-, Sibe -*na*-, Ma. -*na*- ~ -*ne*- ~ -*no*- ‘to go out (to verb)’

OJ *in***-** ‘to go, leave, depart’ belongs to the n-irregular verb paradigm (*na-hen*) along with only two other verbs: OJ *sin*- ‘to die’ and the perfect auxiliary OJ -*in*-, which are probably reflexes of the same etymon (Robbeets 2005: 123, 162.) The n-irregular verb paradigm is an exception to the athematic paradigm (*yodan*) because it has ‘long’ adnominals (*rentaikei*) -*uru* and subjunctives (*meireikei*) -*ure* in contrast to the ‘short’ adnominals -*u* and subjunctives -*e* of the athematic paradigm.

Whitman (1985) has argued that at some proto-Japanese stage *-*r*- was deleted after short vowels but retained after long vowels. The loss of the intervocalic -*r*- in the adnominals and subjunctives of the athematic paradigm is commonly attributed to this rule, so in the case of the n-irregular verb paradigm a preceding long original vowel must have blocked the application of the rule. Since the root vowel in monosyllabic morphemes was automatically long at the proto-Japanese stage, it is inviting to reconstruct monosyllabic pJ **na*- ‘to go’.

The prefix in OJ *in***-** ‘to go, leave, depart’ is a lexicalized instance of the Old Japanese verb prefix *i*-. Various semantic and syntactic analyses of this prefix circulate in the literature[1], but, arguing that Old Japanese has active allignment in nominalized clauses, Yanagida and Whitman (2009: 117-119) demonstrate that the *i*-prefix is exclusively attached to active verbs, i.e. to transitive verbs and to intransitives verbs with an agentive subject. The separate accentuation of *i*- is high atonic 1.1. (Martin 1987: 668), which explains the B register in a number of lexicalisations such as J *imasu* B ‘deign to be/stay/go/come’ (< OJ *mas*- A ‘to deign to be/stay/go/come’), OJ ino_2r- B ‘to pray’ (< OJ no_2r- A ‘to declare’) and OJ *ituk*- B ‘to purify’ (< OJ *tuk*- B ‘to soak’). Lexicalized stems showing a reduced form of *i*- such as OJ *ik*- / *yuk*- ‘to go’, OJ *yokos*- ‘to send here’ and OJ *yusug*-‘to wash out, rinse’ have A register. OJ *in***-** ‘to go, leave, depart’ and OJ *sin*- ‘to die’ have A register. However, in reference to Kindaichi, Martin (1987: 201) points out that the original accent type may be B because “these verbs originally had a fall (instead of just low) on the ending of the predicative […] and that of the infinitive […] like verbs of Type B.” From this perspective, pJ **na*- ‘to go’ may underly derivations such as OJ *nar*- ‘to become, come into being’ and OJ *nas*- ‘to make, do, give birth to’ which have B register. The grammaticalization of ‘to go’ into a change-of-state verb is cross-linguistically well attested (Heine and Kuteva 2002: 156-157.)

A similar pathway of grammaticalization probably underlies in MK ·*na*- ‘to go out, emerge, leave, become, come into being, come out’. In addition to the most common

[1] Martin 1987: 94, 668: independent adverb; Hino 1997: 2-5: agentive marker; Unger 2000: 676: reanalysis of a preceding -*i* converb, Russell 2006: 141-142: goal focus marker; Vovin 2009: 561: directive-locative focus marker.

meaning ‘to become’, the Korean verb is used in the sense of K *na-ka-* ‘to go out, leave’, e.g. in *nwun-ey nata* ‘go out of a person’s favor’. Rhee (1996: 215-216) shows that the basic denotation of MK *·na-* was the motion of getting out of a bounded space. Derivations such as MK *¨nay-* ‘to take out, produce’ with the causative suffix **-i-* and MK *nat-* ‘to appear’ with the passive **-t(i)-* support this sematic analysis. Martin (1992: 263, 702, 933) further considers the so-called “effective suffix” MK -*·na-*, that can only apply to the verb MK *·wo-* ‘to come’ yielding MK *·wo·na-* ‘to end up by coming, ultimately come’, to be a grammaticalization from the auxiliary MK *·na-* ‘to go out, emerge’.

The Tungusic languages share a suffix that denotes departure from a place to other places or towards the object of an action (Benzing 1955a: 1068, Gorelova 2002: 239-240), such as Ma. *feku-* ‘to jump’ → *fekune-* ‘to jump away from the speaker, to jump to the other side’, *guri-* ‘to move’ → *gurine-* ‘to move to another place’ and Ma. *omi-* ‘to drink’ → *omina-* ‘to go to drink’. In Manchu, this construction can be replaced by a periphrastic converb construction with the verb *gene-* ‘to go’. From the viewpoint of cyclic grammaticalization, the synthetic construction may also go back to an original verb pTg **na-* ‘to go out’. Its origin as an independent verb is further supported by the observation that there is no development of vowel harmony for the suffix, except in Manchu.

4 WATER

pK **mɨl* 'water': K *mul*, MK *·mul* 'water'

pTg **mu*: ~ *mö*: '1 water': Evk *mu*: '1', Even *mö*: ~ *mu:* ~ *mo:* '1', Even *mö:v-* ~ *mo:v-* 'dilute with water', Even *mö:de-* ~ *mu:de* ~ *moda-* 'grow, swell (of water in a river)', Even *mö:lken* ~ *mo:lkan* 'wattery', Neg *mu:* '1', Solon *mu:* '1', Oroch *mu:* '1', Ud. *mude* 'inundation', Olcha *mu:* ~ *mue* '1', Orok *mu:* '1', Na. *mue* ~ *muke*, *moko* '1', Sibe *muke:*, *muku:* '1', Ma. *muke* '1', Jur. *mo* '1'

pMo **mören* 'river': MMo. *muren* (SH), *müren* (HY), *mören* (Muq) 'river', WMo. *mören* 'large river, lake', Khal. *mörön*, Ordos *mörön,* Bur. *müre(n),* Kalm *mörn,* Dag. *mur(e),* Dong. *moren*, *moran*, *moron*, Bao. *moroŋ*, Eastern Yugur *mereen*, Mgr. *muro:n*

The common reconstruction of the proto-Tungusic form is **mu:* 'water'. However, Even, Nanai and Jurchen preserve alternant forms such as Even *mo:* 'water', *mo:v-* 'dilute with water' and *moda-* 'grow, swell (of water in a river)', Na. *moko* 'water' and Jur. *mo* that reflect pTg **mö*: 'water' according to Benzing's sound correspondences.[2] Similar to the etymologies under 53 'to give', 79 'to blow' and 80 'wood', an open monosyllabic form with length in Tungusic corresponds to a disyllabic form with a liquid onset in the second syllable in the other Transeurasian languages. This is

---

[2] According to the correspondences in Benzing 1955a:

pTg	Ma.	Jur	Evk	Even	Sol	Neg	Oroch	Ud.	Olch.	Orok	Na
*ö	u		u	o	u	u	o/u	o	o/u	o/u	u
*u	u		u /-ö-	u/ -ö-	u/ -ö-	u/-ö-	u	u	u	u	u

indicative of liquid loss in Tungusic.

As far as Mongolic is concerned, some modern forms suggest **müren*, leaving uncertainty about he original first vowel, but the older varieties as well as the external comparisons support the reconstruction of **mören*.

5 MOUTH

pJ **kutɨ-i* ‘mouth, opening’: J *kuti* (2.1) , OJ *kuti* ‘mouth, aperture, opening’, J *kutu-* 'mouth, opening' in J *kutuwa* ‘mouth-piece, bit’, J *kutugaeru* ‘be overturned, capsize’, etc.; Yamatohama (Amami) *kuci*, Asama (Amami) *kucɨɨ*, Yoron (Amami) *kuci*, Shodon (Amami) *k'uci*, Yonamine (Okinawa) *kucii*, Shuri (Okinawa) *kuci*, Irabu (Miyako) *futsï*, Hirara (Miyako) *futsï* (A), Ishigaki *ɸucï* (Yaeyama), Ikegami (Yaeyama) *futsï* (A), Hatoma (Yaeyama) *huci*, Yonaguni *tti* (A), pR **kuti* 'mouth'; OKog **kʊtsi* 'mouth' (Beckwith 2007: 112, 115, 129)

pK **kwut* ‘hollow, cavity’: K *kwut*, *kwutengi*, MK *·kwut* ‘hollow, pit, cave’

The distinction between i_1 < **i* and i_2 < **ɨi* is not preserved following dental stops in Old Japanese, but it is possible to reconstruct an earlier **ɨi* on the basis of the apophonic pair *kutu-* ~ *kuti* 'mouth'. The word for 'mouth' is reflected in Ryukyuan languages and a possible cognate has been reconstructed in Old Koguryo, which makes reconstruction at the proto-Japanic level inviting. The suffix pJ **-i* is a substantivizer following nouns (e.g. OJ *aka* ‘red’ → ake_2 ‘red object, red cloth’), cognate with the bound noun OJ *i* ‘fact (that); that (which)’ (Robbeets 2015: 341).

pTg **amga* < ? **ama-g* 'mouth': Evk. *amŋa*, Even *amŋe*, Neg. *amŋa*, Solon *amma*, *angai*, Olcha *aŋma*, Orok *amŋa* ~ *aŋma*, Na. *amga*, Oroch *amma*, Ud. *aŋma*, Sibe *aŋa* ~ *aŋə*, Ma. *aŋga*, Jur. *am-ŋa*

pMo **ama-n* 'mouth, opening': MMo. *ama(n)*, WMo. *ama(n)*, Khal. *am(an),* Bur. *ama(n),* Kalm. *amn*, Dag. *am*, *ama*, Eastern Yugur *aman*, Mgr. *ama*, Bao. *amaŋ, aman, amɔŋ,* Dong. *amaŋ, aman,* Mogh. *aman*, *amun*

The Tungusic reconstruction **amga* 'mouth' may ultimately derive from **ama-g*, through the addition of a collective suffix pTg *-*g* (Benzing 1955a: 1016). This suffix is also present in pTg **de:re-g* 'face' reflected in Ma. *dere*, Nanai *dereg* and Olcha *dere* ~ *dereg* 'face'. The Tungusic words for 'hole', i.e. Evk. *aŋa*, Neg. *aŋa* and Olcha *aŋgala* are probably connected to this etymon.

In the Mongolic languages we find an unstable stem-final nasal element, morphophonologically alternating with zero, that expresses singularity in contrast with plural forms on -*d*. This stem-final -*n* was added to the simple stem, yielding pMo **ama-n*. The Mongolic languages, e.g. MMo. *amasar*, WMo. *amasar,* Khal. *amsar,* Bur. *amhar,* Kalm. *amsr,* Dag. *amsər* etc., reflect a derived form pMo **ama-sar* 'opening, cavity, hole'.

pJ **ipa-* ‘to tell’: J *iw-* (A), OJ *ip-* ‘say, tell, talk, speak’, J *iwaw-*, OJ *ipap-* (B) ‘congratulate’; Yamatohama (Amami) *ʔyuuri*, Asama (Amami) *ʔyuɴ,* Yoron (Amami) *ʔjuɴ,* Naze (Amami) *ʔyun*, Yonamine (Okinawa) *ʔyuɴ,* Shuri *ʔyuɴ,* Irabu (Miyako) *nzï*, Hirara (Miyako) *a*^{z}*ï*, Ishigaki (Yaeyama) *izuŋ,* Hatoma (Yaeyama) *azuɴ*, Yonaguni *Nduŋ* 'say'

pK **ip* ‘mouth’: K *ip*, MK *·ip* ‘mouth’

The -*z*- in the Yaeyama and Miyako forms is due to the development of proto-Ryukyuan *-*y*- to -*z*- in these languages, but not in Yonaguni. A similar development is seen in pR **iyu* 'fish' under 40. FISH. The a- in Hirara *a*^{z}*ï* and Hatoma *azuɴ*, is probably epenthetic (cf. Miyako *asï* 'do').

40. FISH

pJ **(y)iwə* 'fish': J *uo* (2.1), OJ *iwo* 'fish', Yamatohama (Amami) *ʔjuu*, Asama (Amami) *ʔyuu*, Yoron (Amami) *ʔyuu*, Shodon (Amami) Ɂ*yuu,* Yonamine (Okinawa) Ɂ*yuu,* Shuri (Okinawa) Ɂ*iyu* (A), Irabu (Miyako) *ïzu*, Hirara (Miyako) ^{z}*ïzu*, Ishigaki (Yaeyama) *idzu* [*izu*] (A), Yonaguni *iyu*, pR **iyu* 'fish'

7. BLOOD

pJ **ti* 'blood; spirit, force': J *ti* (1.1), OJ *ti* 'blood', OJ *ti* 'spirit, force'; Yamatohama (Amami) *ci(i),* Asama (Amami) *Cɨɨ*, Yoron (Amami) *cii*, Shodon (Amami) *cii*, Yonamine (Okinawa) *cii*, Shuri (Okinawa) *cii*, Irabu (Miyako) *ahacï(ï),* Hirara (Miyako) *tsïtsï*, Ishigaki (Yaeyama) *tsïï* [*cïï*] (A), Hatoma (Yaeyama) *sii*, Yonaguni *ccii* 'blood' (A), pR **ti* 'blood'

pMo **či* < **ti* 'blood': MMo. *čisu(n),* WMo. *čisu(n),* Dag. *čos*, Khal. *cus(an),* Ordos *ǰusu*, Bur. *šuha(n)*, Kalm. *cusn*, Eastern Yugur *čüsən*, *čusun*, Mgr. *ćisə, cəʒə,* Bao. *ćisuŋ, ćisɵŋ,* Dong. *čusun*, Mogh. *čusun*, *čɪsu*

pTk **tï:n* ‘spirit, breath’: OT *tïn* ‘1 spirit, breath’, Tk. *tin* ‘1', Tat. *tɨn* ‘1', Uz. *tin* ‘1', Uigh. *tin* ‘1', Kirg. *tɨn* ‘1', Kaz. *tɨnɨs* ‘1', Nog. *tɨnɨs* ‘1', Bash. *tɨn* ‘1', Balk. *tin* ‘1', Kpak *tɨn* ‘1', Kum. *tɨnɨš* ‘1', Khak. *tɨn* ‘1', Shor *tɨn* ‘1', Tuva *tɨn* ‘1', Tofa. *tɨn* ‘1', Yak. *tɨ:n* ‘1', Dol. *tɨ:n* ‘1', Chu. *čĕm*‘1'; Tkm. *dɨ:nč* '2 rest', Az. *dinč* '2', Karaim *tɨnc* '2', Gag. *dinnen*- 'to rest'

Reflexes of pJ **ti* 'blood; spirit, force' are found in Mainland Japanese as well as in the Ryukyuan languages. The metaphorical extension of the meaning 'blood' to 'spirit, force' in Old Japanese supports the comparison with pTk **tï:n* ‘spirit, breath’. The Mongolic forms reflect a petrified suffix -*sun* that occurs in numerous body part terms, e.g. WMo. *gede-sün* 'bowel' and *suda-sun* 'arteria' (Poppe 1973: 238-240).

8. BONE

pJ **pəne* 'bone': J *hone* (2.3), OJ *pone* ‘bone, skeleton, rib’, Yamatohama (Amami) *hunï*, Asama (Amami) *huunɨ*, Yoron (Amami) *puni*, Shodon (Amami) *hunï*, Yonamine (Okinawa) *puni*, Shuri (Okinawa) *huni*, Irabu (Miyako) *puni*, Hirara (Miyako) *puni*, Ishigaki (Yaeyama) *puni*, Igarashi (Yaeyama) *puni*, Yonaguni *huni*, pR **pone* 'bone'

pK **peCi* 'bone': K *ppye* , MK *·spye ~ spey ~ pspey* ‘bone’

pTg **peni-ken* 'knee': Evk. *henŋen*, Even *henŋen*, Solon *eŋẽ,* Neg. *heńŋen*, Olcha *peńe(n),* Orok *pene*, *peŋe(n),* Na. *peiŋẽ*, Oroch *heŋŋe*, *heŋen*, Ud. *heŋe*

Within Frellesvig and Whitman's (2008) seven vowel hypothesis, many instances of OJ *e*$_{(1)}$ in word-final position cannot be etymologized as contractions, but they result from an original pJ **e*. Indeed, the Ryukyuan forms support the reconstruction of pJ **pəne* 'bone' with a final mid-vowel, which raised only in the Ryukyuan languages.

The Middle Korean forms *·spye ~ spey ~ pspey* 'bone' have complex initials, which are all secondarily generated through phonological or morphological developments in Korean. If the initial *s*- in *·spye ~ spey* can be separated as a relic of a genitive *s* in compound structures, reinterpreted as the initial of the second noun

(**pye* ~ **pey*) with '-bone', then we can reconstruct pK **piCe* or **peCi* which contracted to the tonic open monosyllable in Middle Korean.

The Tungusic forms may incorporate an assimilation of the diminutive suffix pTg *-*kA:n* (Benzing 1955a: 1006-1007). This suffix is used with other body parts, e.g. Even *ŋal* 'hand' and *ŋal-ka:n* '(little) hand'.

9. 2 SG pronoun

pJ **na* '2 SG pronoun': OJ *na*, *nare* '2 SG pronoun'; Koniya (Amami) *nam*, Shuri (Okinawa) *naa,* Namizato (Okinawa) *naa*, Sesoko (Okinawa) *naa, naN,* Taketomi (Yaeyama) *naara* 'you, self', pR **naa* '2 SG honorific pronoun'
pK **ne* '2 SG pronoun': MK *ne* '2 SG pronoun'

In Old Japanese *na* is used along with *nare* as the main neutral pronoun to express the second person singular 'you'. Only *nare* is used to express the second person plural. However, since OJ *na* is petrified in some expressions in reference to a first person singular (e.g. OJ *na se* 'my older brother / fellow'), it is possible that it has grammaticalized from an original **na* 'person', reflected in among others OJ *womi*$_1$*na* 'woman' and OJ *oki*$_1$*na* 'old man' (Robbeets 2005: 241). Therefore, I do not exclude that the correspondence with the Korean form may be coincidental. The Ryukyuan pronouns have honorific uses in most languages given above. Contrary to Vovin's (2008: 65) claim "The distribution in the Ryukyus (no attestations in Sakishima) and its mild honorific nature suggest that Ryukyuan *naa* is a loan from Japanese," there is an attestation of this form in the Sakishima languages, notably Taketomi (Yaeyama) *naara* 'you, self'. Hence, Vovin's deduction that the Old Japanese pronoun *na* must be a loan from Korean *ne*, at a time the Ryukyuan languages had already separated, (Vovin 2008: 65, 2013: 254-255) becomes less convincing.

pTg **si* '2 SG pronoun': Evk. *si*, Even *hi:*, Neg. *si:*, Solon *š́i*, Olcha *si*, Na. *śi*, Oroch *si*, Ud. *si*, Sibe *ši:*, Ma. *si*, Ma. *si-ni* '2 SG-genitive', *sim-be* '2SG-accusative', *sin-de* '2 SG-dative', *sin-ci* '2 SG-ablative'
pTk **si* '2 SG pronoun': OT *si-ni* '2 SG-accusative', *sin-tä*, *sin-dä*, *sin-idä* '2 SG-locative', *sin-idin* '2 SG-ablative', *siz* '2 PL pronoun'; Tk. *sen* '2 SG pronoun', *siz* '2 PL pronoun'; Tkm. *sen* '2 SG', *siδ* '2 PL'; Gag. *sen/sän* '2 SG', *siz* '2 PL'; Az. *sän* '2 SG', *siz* '2 PL'; KKalp. *sen* '2 SG', *siz* '2 PL'; Kirg. *sen* '2 SG', *siz(der)/siler* '2 PL'; Bash. *hin* '2 SG', *heδ* '2 PL'; Tat. *sin* '2 SG', *sez* '2 PL'; Karaim *sen* '2 SG', *siz* '2 PL'; KBalk. *sen* '2 SG', *siz* '2 PL'; Kum. *sen* '2 SG', *siz* '2 PL'; Nog. *sän* '2 SG pronoun', *siz* '2 PL pronoun'; Khak. *sin* '2 SG pronoun', *sɨrär* '2 PL pronoun'; Uig. *sän* '2 SG', *silä* '2 PL'; Uz. *sen* '2 SG', *siz* '2 PL'; Shor. *sen* '2 SG', *siler/sler* '2 PL', Tuva *sen* '2 SG', *siler* '2 PL', Tofa. *sän* '2 SG pronoun', *sɨlär* '2 PL pronoun'; Yak. *än* '2 SG pronoun', *ähigi* '2 PL pronoun'; Dolg. *än* '2 SG', *ähigi* '2 PL'; Khalaj *sän* '2 SG pronoun', *siz* '2 PL pronoun'; Chu. *e-sĕ* '2 SG pronoun', *e-sir* '2 PL pronoun'

The Turkic second person plural pronoun can be analyzed as a plural in *-*z* from an original singular stem **si*. The plural -*z* was no longer productive in Old Turkic, but it is attested in a number of petrified forms including paired body parts such as OT *kö-z* 'eyes', *ti-z* 'knees', *agï-z* 'lips' and *kökü-z* 'breasts' and ethnonyms such as OT *ogu-z* and *kïrgï-z*. Similar to the Tungusic paradigm, the Turkic paradigm is marked by the insertion of an oblique suffix -*n*- in some oblique cases. The elision of initial **s*- in Yakut is a regular development, while Yak. -*higi* is a plural suffix.

11. TO COME

pJ **kə-* ‘to come’: J *ko-*, OJ *ko*$_2$*-* to come’ (OJ *ko*$_2$*nu* 'not come'; *ko*$_2$*ba* 'if come'; *ko*$_2$*si* 'came' etc.); Yamatohama (Amami) *ki(i),* Asama (Amami) *kjuɴ*, Yoron (Amami) *kjuɴ,* Yonamine (Okinawa) *sun*, Shuri (Okinawa) *cuuɴ,* Irabu (Miyako) *fuï*, Ikema (Miyako) *fu:,* Ishigaki (Yaeyama) *kï(:)ŋ* [*kï(ï)ɴ*], Yonaguni *kuŋ,* pR **ko-*
pTk **kel-* 'to come': OT *kel-*, Tk. *gel-*, , Az. *gäl-*, Tkm. *gel-*, Gag. *gel-*, Uig. *käl- ~ kil*, Uz. *kel-*, S.-Yugh. *kel-*, Karaim *kel-*, Kpak *kel-*, Tat. *kil-*, Kirg. *kel-*, Kaz. *kel-*, Bash. *kil*, Nog. *kel-*, Balkar *kel-*, Kumyk *gel-*, Sal. *gel-*, *gej-*, Khak. *kil-*, Shor *kel-*, Tuva *kel-*, Tofa *kel-*, Yak. *kel-*, Dol. *kel-*, Khalaj *käl-*, Chu. *kil-*

12. BREAST

pJ **kɨkɨ-rə* 'heart': J *kokoro* (3.5b), OJ *ko*$_2$*ko*$_2$*ro*$_2$ ‘heart’, MJ *kokoti* ‘heart, feelings, mood’; Yamatohama (Asami) xohoro, Asama (Asami) kukuuru, Yoron (Amami) kuuru, Shodon (Asami) *k'ohoro*, Shuri (Okinawa) *kukuru*, Hirara (Miyako) *kukuru*, Irabu (Miyako) kukuru, Ishigaki (Yaeyama) *kukuru* (B), Hatoma (Yaeyama) kukuru Yonaguni *kukuru* (B), pR **kokoro* 'heart'; OKog **kir* ~ **kür* 'heart' (Beckwith 2007: 80, 115)
pTg **xökö-n* 'breast': Evk. *uku-* '1 to suck', *ukun ~ xukun ~ xukur ~ xukuxu* '2 breast, udder, milk', Even *ök- ~ uk-* '1', *ökri ~ ukri* 'pectoral/ chest-; infant, baby', <u>*okan* '2',</u> Solon *uxũ* 'milk', Neg. *öxö- ~ uku-* '1, breast-feed', *öxön ~ ökön* 'milk', Oroch *ovoci- ~ o:ci- ~ ueci-* 'suck', *oko(n)* 'breast, nipple, milk', Ud. *kos’o* - '1', *kos’o ~ oko* ‘2', Olcha *kueci- ~ ko:ci-* ‘1', *kue(n)* 'breast, milk', Orok *ku:tci-* '1', *qu:(n) ~ qo:(n)* '2', Na. *ku:ci- ~ uku-* '1', *kũ* '2', Sibe *xuxuŋ* 'breasts', Ma. *xuxun* 'breast, nipple', *xuxuri* 'infant, baby, suckling, infancy', Jur. *xuxun*‘breasts’
pMo **kökö-n* 'breast': MMo. *koko-* (SH) '1 to suck the breast', *kokan* '2 breast', pl. *kokot*, MMo. *köke-* (Muq) '1', *köken* '2', WMo. *kökö-* '1', *kökö(n) ~ köke(n)* '2', Khal. *xöxö-* '1', *xöx* '2', Ordos *gökö-* '1', *gökö* '2', Bur. *xüxe-* '1', *xüxe(n)* '2', Kalm. *kök-* '1', *kökn* '2', Dag. *mək-* '1', *mək* '2', Eastern Yugur *hkö- ~ hgö-* '1', *hkön ~ hgön* '2', Mgr. *kugo-* '1', *kugo* '2', Bao. *kugo-ɣo-* (*-ɣo-* causative) '1', *kugo* '2' , Dong. *gogo-* '1', *gogo* '2', Mog. *kökȧ-* '1', *kökä* 'nipple'
pTk **kökü-r*$_2$ 'breast': OT *kögüz* '1 breast, 2 reason, sense, emotion', Tk. *göɣüs ~ kökrek* (dial.) '1', Tat. *kögüs* (dial.) ~ *kükrɛk* '1', Uz. *kụks* '1, 2', *kɔkräk* '1', Uigh. *köküs ~ kökräk* '1', S-Yug. *köküs ~ gö:s* '1', Az. *köks ~ köküs* '1', Tkm. *gövüs ~ kükrek* '1', Kirg. *kökürök* '1', Kaz. *kökirek* '1', Nog. *kökirek* '1', Bash. *kükräk* '1', Balkar *kökürek* '1', Gag. *gü:s* '1', Karaim *kökis ~ kökräk* '1', Kpak. *kökirek* '1', Salar *göfrix* '1', Kum. *kökürek* '1', Khak. *kögis* '1', Shor *kögüs* '1', Yak. *köɣüs* ' middle of the back', Dolg. *köksü* 'back', Chu. *kăgăr* '1'

If the Japanese form for 'heart' indeed incorporates a petrified plurality marker pJ **-rə* of the type found in among others OJ *ko*$_1$*-ra* 'children', *woto*$_2$*me*$_1$*-ra* 'young girls', *ye-ra* 'branches', *kinu-wata-ra* 'silk clothes' (Antonov 2007: 195, 197), then the plurality can be taken as indicative of a pre-Japanic semantic shift from 'breasts' to 'heart'. Some of the Ryukyuan forms such as Yoron (Amami) *kuuru* are rather rare. These forms are used in expressions like 'good-hearted' or 'bad-hearted', while the Chinese anatomical borrowing *sinzou* 'heart' is used for the organ, e.g., Yamatohama (Asami) *siɴdo*, Hatoma (Yaeyama) *siɴzoo*, etc. The Ryukyuan attestations and the tentative

Old Koguryo reconstruction suggest that the reconstruction goes back to the proto-Japanic level.[3]

With exception of the Manchu and Nanai words, which may be borrowings from Mongolic, all Tungusic forms support the reconstruction of an initial **x*- in proto-Tungusic.[4] Given the reflexes with alternating *o/u* in Oroch, Olcha and Orok, Udehe *o* in *oko* 'milk' and Even *o* in *okan* 'breast, milk', the vowel should be reconstructed as **ö* in line with the correspondence in footnote 2. The word for 'milk' clearly is a derivation from the verb 'to suck' with the nominalizer pTg *-*n* (Robbeets 2015: 385-391), but as the derivation is shared between most Tungusic languages, it probably had already taken place at the proto-Tungusic or pre-Tungusic level.

Given a parallel derivation of 'milk' from 'to suck' in Mongolic with a cognate nominalizer pMo *-*n* (Robbeets 2015: 391-393), it is likely that the derivation can be traced back to the proto-Transeurasian level. From this perspective, Japanic and Turkic have probably preserved a formation with the common plural suffix pTEA *-*rA*, while Tungusic and Mongolic have preserved the formation with the common deverbal nominalizer and singular pTEA *-*n*. The plural -*z* < pTk *-r_2 has been mentioned under item 9. 2 SG pronoun.

14. 1 SG pronoun

pJ **wa* ~ **wa-n-* '1 SG/PL pronoun': OJ *wa* (1.3 a) ~ *ware* '1 SG pronoun', OJ *ware* '1 PL pronoun'; Yamatohama (Amami) *waɴ,* Asama (Amami) *waɴ*, Yoron (Amami) *waa*, Shodon (Amami) *waa*, Yonamine (Okinawa) *wanuu,* Shuri (Okinawa) *waa* (nominative / genitive) ~ *waɴ* (oblique) (B), Irabu (Miyako) *a-*, Hirara (Miyako) *baŋ*, Ishgaki (Yaeyama) *banu* (A), Hatoma (Yaeyama) *baa*, Yonaguni *banu* (A), pR **wa-nu* '1 SG pronoun'

pMo **ba* ~ **ba-n-* '1 PL excl. pronoun': MMo. *ba* (nominative) ~ *ma-n-* (dative/ locative), WMo. *ba* ~ *man-*, Dag. *ba:* ~ *ma:n-* Khal. *man-*, Bur. *man-*, Kalm. *man-*, Ordos *man-*, Dong. *mani* / *mayi* (genitive/accusative), *mai* 'our (postnominal possessive)', Bao. *man-*, Eastern Yug. *bə*, *manə* 'our (postnominal possessive)', Mog. *mo:n-*

Both OJ *wa* and *ware* are used for the first person singular in Old Japanese, but only *ware* is used for the plural. In contrast to modern Japanese (e.g. *watakusi* 'I' ~ *watakusi-tati* 'we') none of these pronouns can be followed by productive plural markers. This observation suggests that the suffix -*re (*< -ra-(C)i)* goes back to the plural suffix OJ -*ra*, mentioned under 12. BREAST. When the suffix -*re* lexicalized in a way that it was no longer identified with the plural, the petrified plural *ware* probably spread by analogy to the singular. The final nasal in the Ryukyuan forms is probably a reflex of an original oblique case suffix pJ *-*n(u)-,* which is also reflected in the East Old Japanese dative case *wa-nu-ni*.

The Mongolic languages display a similar alternation between pMo **ba* and **ma-*

---

[3] I use the term "Japanic" in reference to a genealogical unity that comprises the historical continental varieties of the Japanese language as well as the varieties spoken on the Japanese Islands, including the Ryukyu Islands. The label "Japonic" is usually restricted to a branch of Japanic, namely the language family composed of Mainland Japanese and the Ryukyuan languages.

[4] Benzing (1955a: 976, 989) lists the following correspondences.

pTg	Ma.	Na.	Olch.	Orok	Oroch.	Ud.	Sol.	Neg.	Ev.	Lam.
*x-	Ø	x-	x-	x-	Ø	Ø	Ø	Ø	Ø	Ø
*k-	k-~x	Ø	Ø	Ø	k-~x	x-~g	x-	x-	k-	k-

n- the nominative and oblique case forms of the first person plural exclusive pronoun. The initial **b* assimilated to the nasal oblique suffix. Some oblique forms survived in languages that lost **ba* itself.

pTg **bi* '1 SG pronoun': Evk. *bi*, Even *bi*, Neg. *bi*, Solon *bi*, Oroch *bi*, Ud. *bi*, Na. *mi*, *bi* (dial.), Orok *bi*, Olcha *bi*, Sibe *bi:,* Ma. *bi*, Jur. *mi-n*
pMo **bi* '1 SG pronoun': MMo. *bi*, WMo. *bi*, Dag. *bi*:, Khal. *bi*, Bur. *bi*, Kalm. *bi*, Ordos *bi*, Dong. *bi*, Bao. *bi*, *bu*, *bə*, Eastern Yugur *bu*, *bə*, Mgr. *bi*, *bu*, Mog. *bi*
pTk **bi* '1 SG pronoun': OT *min* '1 1 SG pronoun', OT *biz* '2 1 PL pronoun'; Tk. *ben* '1', Tk. *biz* '2'; Az. *män* '1', *biz* '2'; Tkm. *men* '1', *bi:z* '2'; Gag. *ben* '1', *bis* '2'; Uz. *men* '1', *biz* '2'; Uig. *män* '1' ~ *biz* '2', Kirg. *men* '1', *miz* '2'; Karaim *men* '1', *biz* '2', Kaz. *min* '1', *biz* '2'; Nog. *män* '1', *biz* '2', Balkar *men* '1', *miz* '2'; Kpak *men* '1', *biz* '2'; Salar *me:(n)* '1', *pise(r)* '2'*;* Kum. *men* '1', *biz* '2', Tat. *min* '1', *bez* '2'; Bash. *min* '1', *beð* '2'; S-Yug.*pɨz* '2', Khak. *min* '1', *pɨs* '2', Shor *men* '1', *pis* '2', Tuva *men* '1', *bis* '2', Tofa. *män* '1', *bi's* '2'; Yak. *min* '1', *bihigi* '2', Dolg. *min* '1', *bihigi* '2'; Khalaj *män* '1', *biz* '2', Chu. *e-pĕ ~ e-bĕ* '1', *e-pir ~ e-bĕr* '2'

Parallel to the Turkic second person plural pronoun discussed under 9. 2 SG pronoun, the first person plural pronoun can be analyzed as a plural in **-z* from an original singular stem **bi or *bä*. Only the Turkic forms with a high front vowel as well as Chu. *e-pĕ* reflect the original first singular pronoun **bi*.

16. LOUSE
pMo**sirke* 'louse': MMo. *sərke* 'nit', WMo. *sirke* 'a kind of flea', Khal. *širx* '1 cattle louse', Bur. *šerxe* '1', Kalm. *širkə* '1', Ordos *širχe* '1'
pTk **sirke* '1 nit': OT (Karakh.) *sirke* '1', Tk. *sirke* '1', Az. *sirkä* '1', Tkm. *sirke* '1', Gag. *sirkä* '1', Tat. *sĭrkɛ* '1', Kirg. *sirke*, Kaz. *sĭrke*, Nog. *sirke* '1', Bash. *hĭrkä* '1', Balk. *sirke* '1', Karaim *sirke* '1', Kpak. *sirke* '1', Kum. *sirke* '1', Uz. *sĭrkɛ* '1', Uig. *sĭ(r)kä* '1', Khak. *sĭrge* '1', Tuva *sirge* '1', Tofa. *si'rxe* '1', Khalaj *sirkä* '1', Chu. *šărga* '1'

19. ARM/HAND
pJ **ta(r)i* 'upper limb, arm, hand': J *te* (1.3a), OJ *te*, OJ *ta-* in e.g. OJ *tanapidi* 'elbow' (<*ta-* 'arm' + *na* genitive + *pidi* 'joint') and OJ *tomo* 'archer's arm protector' (< *ta-* 'arm' + *omo* 'surface'); Yamatohama (Amami) *θï(ï),* Asama (Amami) *tɨɨ*, Yoron (Amami) *tii*, Shodon (Amami) *t'II*, Yonamine (Okinawa) *tii*, Shuri (Okinawa) *tii*, Irabu (Miyako) *tii*, Hirara (Miyako) *tii*, Ishigaki (Yaeyama) *tii* (B), Hatoma (Yaeyama) *tii*, Yonaguni *tii* (B), pR **te* 'arm, hand'
pK **tali* 'lower limb, leg': K *tali*, MK *·tali* 'leg, limb'

The Transeurasian languages display a tendency to use a single term for both the limb and its extremity. When new words for 'arm, hand' or 'leg, foot' come in, they are likely to push the meanings of previous forms into designating a specific subpart of the limb. This development is expected to cause overlapping meanings such as between MK *·pal* 'foot, leg, paw' and MK *·tali* 'leg, limb'. The crossing-over of the semantic correspondence between upper limb and lower limb in both etymologies may be explained in the context of naming animal limbs.

pJ **sune* 'lower limb, leg': J *sune* (2.3), OJ *sune* ' leg, shin, shank'; Asama (Amami) *sɨnɨɨ*, Naze (Amami) *sïnï*, Shuri (Okinawa) *şini*, Hirara (Miyako) *karasïni*, Ishigaki (Yaeyama) *sïni* (B), Yonaguni *ccini* (B), pR **sune* 'leg, shin, shank'

pK **son* ‘upper limb, arm, hand’: K *son*, MK *·swon* 'hand'

Since the quality of the mid vowel is not distinguished after *n* in Old Japanese, we could be dealing with either e_1 (<**i(C)a*, **i(C)ə*, **i(C)ɨ*, **e*) or e_2 (< **a(C)i* /**ə(C)i*). However, as there are no apophonic alternations attested for this root and since nominal roots rarely consist of more than two syllables, preference is given to the reconstruction of pJ **sune*. The Ryukyuan forms support this reconstruction.

pMo **gar* 'hand, arm': MMo. *qar*, *qa:r*, *ɣar* 'hand, wing (of an army)', WMo. *ɣar* 'hand, arm, flank, wing', Khal. *gar* 'hand, arm, wing (of an army)', Bur. *gar*, Kalm. *ɣar*, Ordos *Gar*, Dong. *qa*, Bao. *xar*, Dag. *gari*, *gaŕ*, Eastern Yugur *ġar*, Mgr. *ġar*, Mog. *ɣar*
pTk **kar-ï* 'arm' (pTk *-*ï* 3SG possessive) : OT *kar* '1 arm', *karï* (OUig.) '2 forearm, cubit (measure of length based on the length of the forearm from the elbow to the tip of the middle finger, ca. 46 centimeters)', Karakh. *karï* '1', Tk. *karaǯa* 'upper arm, biceps and triceps muscle', Tkm. *garï* '3 shin-bone of animal', 4 measure of length', Az. *garï* (dial.) '3', Tat. *qarï* '1', *qara* (dial.) '4', Kirg. *qar* '1', *qarï* '1', Kaz. *qar* '2', *qarï* '2, 3', Nog. *qarï* '4', Bash. *qar* '3', Balk. *qarï* '4', Kpak *qar* '2', *qarï* '2, 4', Uz. *qari* '1, measure of length, ca. 140–145 centimeters', *qara* (dial.) '3', Uig. *qeri* '4', *qaya* (dial.) '3', S-Yug. *qar* '2', Shor *qarɨ* '1', Oyrat *qarï* '1', Yak. *xarï, xarï:, xara* '2, 3', Tuva *qïrï* '2', Tofa. *qïrï* '2', Dlg. *karï* 'radius (bone on the forearm)', Khalaj *qarï* '4', Chu. *xor, xur* '2, 4', *xurǎ* 'measure of length, ca. 2 meters'.

In Turkic, we can reconstruct pTk **kar* 'arm' followed by a lexicalised possessive suffix *-*ï*. This possessive suffix frequently occurs with words for primary parts of the body (*Róna-Tas & Berta* 2011: 492-494). The common denominator 'arm' is reflected in a variety of meanings denoting different parts of the arm in addition to the meanings relating to measures of length, ranging between the length of only the forearm to that of one or two arms. The attestation of Hungarian *kar* 'arm' supports the reconstruction of West Old Turkic **kar* 'arm'. Since the Chuvash word lacks a final vowel, it is likely that it was also lacking in West Old Turkic. Cross-linguistically the borrowing from the word for 'arm' has been observed in a trade context, involving the meaning 'measure of length' or in a martial context, involving the meaning 'part of an army'. Interestingly, the Turkic and Mongolic forms share the primary meaning 'arm', but they have each developed a distinct secondary meaning, respectively in a trade and military context. This observation argues against a borrowing scenario.

22. EAR
pMo **kul* 'ear': WMo. *qulki*, *qulaquu* '1 earwax, middle ear', *qulmayi-* 'to have the ears laid back, to be crop-eared (intr.)', Dag. *xoĺgi*, Khal. *xulxi* '1', *xulga* '1', Bur. *xulxa* '1', *xolxi* '1', Kalm. *xulxə* '1', *xuĺxə* '1', Ordos *xuluGu(n)* '1', Mgr. *xoŋGo*.
pTk **kul-kak* 'ear': OT *kulxak* ~ *kulkak* ~ *kulgak*, Tk. *kulak*, Az. *GulaG*, Tkm. *Gulaq*, Gag. *qulaq*, Uz. *qulɔq*, Uig. *qulaq*, Tat. *qolaq*, Kirg. *qulaq*, Kaz. *qulaq*, Nog. *qulaq*, Bash. *qolaq*, Balkar *qulaq*, Karaim *qulax*, Kpak *qulaq*, Salar *Gulaχ*, Kum. *qulaq*, S-Yug. *qulaq*, Khak. *xulax*, Shor *qulaq*, Tuva *qulaq*, Tofa. *qulaq*, Yak. *kulga:k*, Dolg. *kulga:k*, Khalaj *qulaq*, Chu. *xǎlɣa*.

The Mongolic languages reflect a root pMo **kul* 'ear', derived with different suffixes *-*ki*, *-*ku* and *-*mayi-*. The suffix *-*ki* can be identified as a locational suffix, indicating 'that which is located in/on/at', e.g. *degere* 'top, on top of, above' →

degereki 'being above or on top, upper', *emüne* 'south, southern, front' → *emüneki* 'being in front or ahead of (with gen.), southern, preceding' and *qoyitu* 'back, rear, behind, north' → *qoyituki* 'that which is behind or will follow, northern, back, rear, placenta'. Hence, pMo **kul-ki* 'earwax, middle ear' can be analysed as 'that which is located in the ear'.

The Turkic languages reflect a common suffix pTk **-xAk* used in body part terms, such as *tiz* 'knee' → *tirsgäk* 'elbow', *müŋüz* 'horn' → *müŋüzgäk* 'hard skin on the hand, which results from manual labour', *yan* 'side' → *yaŋak* 'cheek-bone' (Erdal 1991:74-76). The alternation of OT *-gAk* with *-kAk* following *l*, *n* and *r* can be explained by reconstructing pTk **-xAk*, which is still present in the attestation of OT *kulxak*. The voiceless fricative was voiced to *-γAk and merged with -gAk* in vocalic environments, but underwent defricativization to *-kAk* in certain continuant environments (Robbeets 2015: 411-416). Therefore, we can reconstruct pTk **kul* as the root for 'ear'.

24. FAR

pJ **mara* 'rare, from afar': J *mare* (2.1), OJ *mare* 'rare, unusual' ~ OJ *mara-* 'from a distance' in e.g. *marapito* ‘a person who came to visit from another place, guest' (< *mara* 'from a distance' + *pito* 'person')

pK **melɨ-* ‘be far’: K *mel-*, MK *mel-*/¨*me(l)-* ‘be far, distant’

25. TO DO/MAKE

pJ *-*ka-* ‘to produce a sound or a sensation like the base onomatopoea’: J, OJ -*k*-, pR *-*k*-, e.g. in pJ **na* 'crying sound' in OJ *ne* (1.1) ‘sound, crying, weeping’ → OJ *nak-*A ‘to cry’; Yamatohama (Amami) *naxjuri*, Asama (Amami) *nakyuɴ*, Yoron (Amami) *nakyuɴ*, Shodon (Amami) *nak'yum*, Yonamine (Okinawa) *nacuɴ*, Shuri (Okinawa) *nacyun* [*nacuɴ*], Irabu (Miyako) *nafu*, Ishigaki (Yaeyama) *na(ʔ)kun* [*nakuɴ*], Hatoma (Yaeyama) *nakuɴ,* Hateruma (Yaeyama) *naguɴ*, Yonaguni *naguɴ,* pR **naki* 'to cry'

pK *-*ki-* ‘to produce a sound or a sensation like the base onomatopoea’: K, MK -*i*-, e.g. in K *kutek* 'nodding' → K *kuteki*, MK *kuteki-* 'to nod (one's head)'

pTg *-*ki:-* ~ -*gi:-* ‘to produce a sound or a sensation like the base onomatopoea’: Ma./Sibe -*ki-* ~ -*gi-*, Evk. -*ki:-* ~ -*gi:-*, Neg./Solon -*ki-* ~ -*gi-*, Even -*k-* ~ -*g-*, -*kA-* ~ -*gA-*, Orok/Olcha/Oroch /Ud./Na. -*ki-* ~ -*gi-*. e.g. in pTg **sim-ki* 'to cough' in Even *hi:mke-*, Evk. *simki-*, Neg. *simki-*, Olcha *siŋbi-*, Orok *sipki-*, Na. *siŋbi-*, *simki-*, Oroch *simpi-*, Ud. *simpi-* and Solon *simki-*.

pMo **ki-* 'to do, make; produce a sound or a sensation like the base onomatopoea': MMo. *ki-*, WMo. *ki-*, Khal. *xij-*, Bur. *xe-*, Kalm. *ke-*, Ordos *ki:-*, Dong. *kie-*, Bao. *ke-*, *giə-*, Dag. *ki:-*, *xi:-*, *ši:-*, Mgr. *gi-*, *gə-*, Mogh. *ki-*, Eastern Yugur *gə;* iconic in e.g. **čis* (mimetic for *chirping)* → WMo. *čiski-* ‘to chirrup, chirp, twitter, tweet’

pTk **kï(-)l-* 'to do, make, produce a sound or a sensation like the base onomatopoea': OT *kïl-*,Tk. *kïl-*, Tat. *qïl-*, Uzb. *qil-*, Uigh. *qil-*, Az. *gïl-*, Tkm. *qïl-*, Khak. *xïl-*, Balkar *qɨl-*, Shor *qïl-*, ,Tuva *qïl-*, Yak. *kïn-*, Dolg. *gïn-* Kirg. *qïl-*, Kaz. *qïl-*, Nog. *qïl-*, Bash. *qïl-*, Karaim *qïl-*, Kpak *qïl-*, Kum. *qïl-*, Chu. *ĕś-xĕl* ‘deed’; iconic in e.g. **su* (mimetic for snapping) → OTk. *sukï-* ‘snap (one’s fingers)'.

The aberrant vowel in Japonic can be explained by resonance with the wide-spread *a*-vocalism of suffixes in the Japanese verbal paradigm.

In Turkic and Mongolic, the verb ‘to do, make’ seems to be the source of grammaticalization for the iconic suffix. In Turkic, Yakut and Dolgan have a different root-final consonant, which could suggest that the original root is **kï-* and that *-l-* and *-n-* are petrified suffixes. The problem with this explanation, however, is that the

suffix -*(X)l*- derives passives and that -*(X)n*- derives medial verbs in Turkic. The verb *kïl*-, however, is typically causative. For a detailed explanation of this etymology, see Robbeets (2015: 239-245).

26. HOUSE

pJ **(y)ipi* ‘house, hut’: J *ie* (2.3), WOJ ipe_1, EOJ ipi_1, *ipa* 'house'
pK **cip* < ? **cipi* 'house': K *cip*, MK *cip* 'house'
pTg **ji:b* 'house' in **ji:b-na:-* 'go visiting': Oroch *ji:ma-*, Olcha *ji:ma*, Ud. *jima-*, Orok *je:ma-*, Na. *ji:ma-* 'go visiting'.

Although Old Japanese distinguishes between *a* and *ya*, $o_{(1/2)}$ and $yo_{(1/2)}$, *u* and *yu* and $e_{(1/2)}$ and *ye*, **yi* and *i* have completely neutralized as OJ *i*. It is thus not self-evident to derive all instances of OJ *i* from pJ **i*. As the choice between pJ **i* and pJ **yi* is always dependent on the external cognates, I insert *(y)* between brackets in the reconstruction of pJ **(y)ipi* ‘house, hut’. The vowel alternation between WOJ ipe_1 and EOJ *ipa* 'house' suggest the reconstruction of pJ **(y)ipi-(C)a*, see for instance the alternation between the progressive adnominal form WOJ ipe_1ru 'say' and EOJ *ipar*- 'say', which derives from **ip-i ar-u* (say-CONV exist-ADN). Therefore a complex origin as **ipi-ya* with OJ/pJ **ya* 'house' cannot be excluded.

The low pitch of the Middle Korean form points to a disyllabic low-high origin, in which the second vowel may be a reduced vowel or the neutral vowel *i.

The Tungusic reconstruction **ji:b* 'house' with a high front vowel is only maintained in the derivation with pTg *-*na*:- ‘to go out’; see 3. TO GO. The assimilation to *-*ma*- leaves a trace of the original final **b* in the word. The free words for 'house' have shifted their vowels backwards in Evk. *ju:w-ča:-* 'to do housework', Evk. *ju:* 'house'; Even *ju:,* Neg. *jo:,* Olcha *ju:(ɣ),* Orok *du:-qu̧*, Na. *jo:,* Oroch *ju(g),* Ud. *jugdi*, Solon *ju:ɣ* and Ma. *jeofi* 'round hovel thatched with straw or birch bark'. The vowel quality may be influenced through contact with Mongolic forms such as WMo. *juuqa*, Khal. *ju:x*, Bur. *zu:xa*, Kalm. *zu:xə*, Ordos *ju:xa*, and Dong. *jowo* for 'stove, hearth'.

Note that in my view, the initial correspondence reflects an original voiced dental stop pTEA **d*, rather than a voiced fricative **j* as suggested by Whitman's (1985) Japano-Koreanic reconstructions. Parallel to the voiceless velar fricative correspondence 21 for pTEA **x*, there is no evidence for a voiced palatal fricative series in Transeurasian. The fact that the correspondence between OJ *y*, MK *c* and Tg *j* and Mo *j* is only found preceding *i* or *y* supports this reconstruction.

27. STONE/ ROCK

pTg **kada:-r* 'rock, cliff' (pTg *-*r* plural suffix, e.g. Evk./ Even *oron* 'tame reindeer' → *oror* 'tame reindeers', Even *buyu:n* 'wild reindeer' → *buyu:r* 'wild reindeers'; Benzing 1955: 1025-1026): Evk. *kadar*, *kadaɣa* '1 rock', Even *qada:r* 'high and steep rock, cliff', *qadaqu̧* '1', Solon *xada:r* '1', Neg. *kada:* '1', Olcha *qadali̧* '1', Orok *qada* '1', Na. *qadar* 'granite, marble', Oroch *kada* '1', Ud. *kada* '1', Sibe *hadə* 'mountain peak', Ma. *χada* 'crag, small cliff, rocky summit; peak'
pMo **kada* 'rock, cliff': WMo. *qada(n)* '1 rock, cliff, crag, ravine ', MMo. *qada* (SH) ‘1', Khal. *xad(an)* '1', Bur. *xada* 'mountain', Kalm. *xadə* 'rock, mountain', Ordos *xada(n*) '1', Dag. *xada*, *xad*, *hade* '1', Dong. *ġada* '1', Mgr. *ġadā* '1', Eastern Yugur *ġada* '1'

The initial weakening in the Shirongolic Mongolic languages and in Eastern

Yugur is due to the following *-d-* (Nugteren 2007: 398).

30. TOOTH

pJ **pa* 'tooth': J *ha*, OJ *pa* 'tooth'; Yamatohama (Amami) *hwa*, Asama (Amami) *haa*, Yoron (Amami) *paa*, Naze (Amami) *ha*, Yonamine (Okinawa) *paa*, Shuri (Okinawa) *ha* 'tooth, blade', Hirara (Miyako) *paa*, Irabu (Miyako) *paa*, Ishigaki (Yaeyama) *paa* (B), Hatoma (Yaeyama) *paa*, Yonaguni *ha:* (B), pR **pa* 'tooth'
pK **pal* 'tooth': K *ispal*, MK *nispal* 'tooth' < **ni* '(specific) tooth' + *s* genitive + *pal* 'tooth', K *i*, MK *·ni* 'tooth'

Although Robbeets (2005: 400) included the Tungusic forms Olcha *paḷi* and Nanai *paloa* in this etymology, we have left them out here because of the poor distribution of the Tungusic form and the problematic reconstruction of the final vowel in pTg **palV* 'molar'.

pMo **ari-ga* 'molar, canine, fang' (pMo *-*GA* body part suffix, e.g. pMo **kil-ga* 'coarse hair'; see 31. HAIR): WMo. *araɣa ~ ariya* 'molar, tooth of a cogwheel', *aru araɣa* 'wisdom tooth', MMo. *ara'a ~ aral* 'fang', *aratai* 'river deer, predator', *ari'a sidün* 'molar; canine; fang, tusk' (MMo. *sidün* 'tooth'), Khal. *araa(n)* 'molar, tooth of a chisel', Bur. *ara:(n)* 'molar, fang, tooth of a chisel', Ordos *ara:* '1 molar, fang', *araŋġa* 'an extra tooth', Kalm. *aran* '1', Dag. *ara: (šid)* 'molar, fang, tooth of a chisel', Baoan *arə*, *ariya* '1', Mgr. *ara:* '1', Eastern Yugur *ara:* '1'
pTk **ar*$_2$*(-)ïg* 'molar, fang': OT *azïɣ* (OUig.) 'canine tooth of animals, tusk, fang', Karakh. *azïɣ*, Tk. *azï* 'molar tooth, tusk (of a boar)', Az. *azï*, Tkm. *azï*, Tat. *azaw*, Kirg. *azu:*, Kaz. *azuw*, Bash. *aδaw*, Balk. *azaw*, Kpak *azuw*, Kum. *azuw*, Uz. *ɔziq*, S-Yug. *azïɣ*, Khak. *azïɣ*, Shor *azïy*, Oyrat *azu*, *azu:*, Tuva *azïɣ*, Tofa. *azïɣ*, Yak. *ahï:*, dial. *ïhï:*

The segmentation of the Mongolic form is supported by the Manchu loanword *arsun* 'shoot, bud, fang', which is probably borrowed from pMo **ar-sun*, a derivation of this root for 'fang' with the Mongolic collective and body part suffix *-*sUn*; see 7. BLOOD. The Mongolic word **ariga* 'molar, canine, fang' is often combined with the root **sidün* 'tooth'. This root may derive from pMo **sil* 'tooth' followed by the same body part suffix; see etymology below.

Doerfer (1965: 55-56) proposed to derive OT *azïɣ* 'canine tooth of animals, tusk, fang' from the verb *az-* 'go astray'. This analysis should be rejected because from the attestation of Tkm. *azï* 'molar tooth' with a short vowel and Tkm *a:z-* 'go astray' with a long one, we know that proto-Turkic had two distinct roots with a difference in vowel length (Tekin 1986: 144-146). Contrary to his previous idea, Doerfer (1984: 37-38) derived OT *azïɣ* in the same way as Turkish *el* 'hand' can be derived from OT *elig* 'hand', suggesting a body part suffix *-*ig* ~ *-*ïg*. If Doerfer's analysis is correct, the original Turkic form should be pTk **ar*$_2$*(ï)* 'fang', an exact match with the Mongolic root.

There is no Chuvash cognate preserved, but as borrowings of a Bulgharic form into the surrounding Uralic Mari languages, one may add Hill Mari **arə̑* preserved in *ar-wü* 'fang' and Meadow Mari **ora* in *ora-püj* 'fang' (*-püj*, *-wü* 'tooth'). The Hill Mari form preserves the Volga Bulghar **arï*, and Meadow Mari reflects the same word with a Chuvash diminutive suffix. We would expect ***urə̑* and its diminutive ***ur-a* in contemporary Chuvash, but the root was lost.

pMo **sidün* < **sil-sün* ‘tooth' (pMo*-*sUn* collective and body part suffix; see 7. BLOOD): MMo. *šidun*, *šidu*, *sudu*, *sidon*, *sidon*, *sidun*, *sidūn* ‘tooth’, WMo *sidü(n)* ‘tooth; any projection suggestive in shape of a tooth’, Khal. *šüd(en),* Bur. *šüde(n),* Kalm. *šüdṇ*, Ordos *šüdü*, Dong. *šədun*, Bao. *(š)doŋ*, *hdoŋ*, Dag. *šide*, *šid*, Eastern Yugur *šdən*, *šədən*, Mgr. *šdi*, Mogol *südün* ‘tooth’
pTk **sil*$_2$ ‘tooth; sharp stick’: OT *siš* ‘1 sharp stick’, Karakh. *sïš*, MT *sɨš* ‘1’, Tk. *šiš* ‘1’, Tat. *šeš* ‘1’, Uigh. *šiš* ‘1’, Az. *šiš* ‘1’, Tkm. *čīš* ‘1’, Khak. *səs* ‘1’, Shor *šiš* ‘1’ Oyrat *šiš* ‘1’, Tuva *šiš* ‘1’, Tofa. *šiš* ‘1’, Kirg. *šiš* ‘1’, Kaz. *is* ‘1’, Bash. *šeš* ‘1’, Balkar *šiš* ‘1’, Karaim *šɨš* ‘1’, Kkp *is* ‘1’, Kum. *čiš* ‘1’, Chu. *šăl* ‘tooth’

It is appealing to compare pMo **sil* 'tooth' with proto-Bulgharic **sil*$_2$ ‘tooth’ underlying in Chuvash *šăl* ‘tooth’. This form corresponds to Old Turkic *siš* ‘sharp stick’ and contemporary cognates. The semantic development from ‘tooth’ to ‘sharp protrusion’ is cross-linguistically well attested, for instance, in the technological meaning of ‘tooth’ in English and in Russian *zubets* ‘a sharp protrusion’, which derives from *zub* ‘tooth’.

31. HAIR

pJ **kama ~ kami* 'hair on the head': J *kami* 'hair', OJ *kami*$_1$ ‘top hair’; Yamatohama (Amami) *xamaci* 'hair, head', Ishigaki (Yaeyama) *akamazï ~ ganǯi*, Hatoma (Yaeyama) *gamazi*, pR **kamadi* 'hair of the head'
pK **kama* ‘hair on the head’: K *kama*, MK *ka·ma* ‘the whirl of hair on the crown of one's the head’

pJ **kara ~ ka(r)i* 'hair': J *ke* (1.1), OJ *ke*$_2$ 'hair' ~ *ka* 'hair' in OJ *siraka* 'white hair'; Asama (Amami) *karaazɨɴkɨɨ*, Yoron (Amami) *hurazi*, Yonamine (Okinawa) *haraazi*, Shuri (Okinawa) *karazi* (B), Irabu (Miyako) *karazï*, Hirara (Miyako) *kara*^{d}*zï*, Yonaguni *karaN ~ kanaN* (A), *karaNgi* 'mane', pR **karadi* 'hair of the head'
pK **kal* ‘hair on the head’ (pK *-*(a/e)k* diminutive in body parts, e.g. *muluph* 'knee' → *muluphak* 'knee', *thel* 'hair' → *thelek* 'hair'): K *khal* ‘hair on the head’ (< pK **kal-ʌk)* MK¨*kalki* 'mane' (< MK¨*kalk-i* with -*i* diminutive in e.g. i 'spotted dog', *ka:yttong-i* 'little dog dropping (said affectionately to babies)' < pK **kal-ʌk-i*)

The two etymologies above indicate an alternation between pJ **kama ~ kami* 'hair on the head' and **kara ~ kari* 'hair on the head' which can be traced back to the Japano-Koreanic level because we can reconstruct both pK **kama* ‘hair on the head’ and **kal(a)* ‘hair on the head’ in proto-Koreanic.

pMo **kilga-sun* 'coarse hair': MMo. *kilqasun* (SH) '(horse's) tail hair', *qilɣasun* (Muq.) 'horse hair', Dag. *kilga:s* 'horse's tail hair', Khal. *x́algas(an)* 'hair of the mane and tail', Ordos *kilġasu(n),* Bur. *xilga:ha(n)* 'horse hair', Brg *šalgʊ:* 'coarse hair', Kalm. *kilɣəsn ~ kilɣsn*, Eastern Yug. *χalğasən* 'pubic hair', Dong. *imaŋ qağasun* 'goat's hair', Mog. *qilɣasun* 'thick hair', Mgr. *ćirGāʒə* 'coarse hair'
pTk **kïl(-k)* 'hair': OT *kïl*, Tk. *kɨl*, Az. *Gɨl*, Tkm. *Gɨl*, Uz. *qil*, Gag. *qɨl*, Uig. *qil*, S-Yug. *qɨl*, Khak. *xɨl*, Shor *qɨl*, Tuva *xɨl*, Tofa. *xɨl*,Yak. *kɨl*, Dolg. *kɨl* 'sealine', Tat. *qɨl*, Kirg. *qɨl*, Kaz. *qɨl*, Nog. *qɨl*, Bash. *qɨl*, Balkar *qɨl*, Karaim *qɨl*, Kpak *qɨl*, Kum. *qɨl*, Khalaj *qɨl*, Chu. *xĕlĕx*

The Mongolic languages reflect the collective and body part suffix *-*sUn*, which is

also identified under 7. BLOOD. Since stem-final *n* drops before this suffix, I do not exclude that pMo **kilga* ultimately goes back to **kil-ka:n*, a diminutive derivation common in body part terms, e.g. MMo. *alaqa:n*, WMo. *alagan* 'palm of the hand', MMo. *elike:n*, WMo. *eligen* 'liver', WMo. *böldegen* 'testicles', etc. (Poppe 1973: 225-226).

Although most Turkic languages reflect pTk **kïl*, Chuvash *xələx* indicates a final velar stop in Turkic. Mudrak (1989) argues that a root-final consonant cluster should be reconstructed to Proto-Turkic in the cases where Chuvash has an additional velar after a sonorant. The proposed rule is that PTk *-*lk* yields -*l* in Common Turkic but -*lĕx*/-*lĕk* in Chuvash. The bracketed *(-k)* in pTk **kïl(-k)* 'hair' may well be a trace of an ancient body part suffix pTk *-(*ï)k*~ -(*i)k,* which is also reflected in pTk **ka:p-ïk* 'bark, shell' and *top-ïk* 'ankle bone, wrist bone, knee cap'; see 68. SKIN.

32. BIG

pJ **kɨkɨ*- 'to be many': OJ *kokoda* 'very much, plenty', -*da* collective in OJ e_1 ~ e_1*da* 'branch', OJ *iku* - 'how much' in some compounds ~ OJ *ikuda* 'how much, some amount', OJ *sapada* 'much, a lot', etc.
pK **kɨkɨ*- 'to be big': K *khu*-, MK ·*khu*- 'to be big'
pTk **kök* 'big, healthy, thick': Tk. (dial.) *kök*, Az. *kök*, Khalaj *kök*

In line with Ramsey (1993: 438; 1997), verb stems with complex initials that are tonic and monosyllabic and have minimal vowels (MK *o*, *u*, *i*) are thought to be created through the loss of a first-syllable vowel. Taking into account velar lenition (cf. 32 BIG and 55 BURN), this internal analysis justifies the reconstruction of pK **kɨhɨ*- < **kɨkɨ*- 'big'.

The word is sparsely distributed in Turkic, but appears to have been borrowed as Khalkha *xöx* 'massive (of muscles, flesh etc.)'.

pJ **mana*- 'to be many, big', **manki*- 'to be big': J *amaneku* 'plenty', OJ *mane*- ~ *amane*- ~ *samane*- (B) 'to be many', *amane*- (B) 'to be extensive', Yonamine (Okinawa) *magiseɴ* ~ *µaagiseɴ,* Shuri (Okinawa) *mandoon* 'to be/have much' ~ *magisaɴ* (A) 'to be big', Ishigaki (Yaeyama) *maisjaaɴ,* Hatoma (Yaeyama) *maijaɴ,* Yonaguni *mai*- (A) 'to be big'
pTg **mani* 'crowd': Evk. *mani* '1 crowd, flock, herd', Neg. *man* '1', Oroch *mańi* '1', Ud. *mani* '1'; pTg **man(i)-di* 'crowdy': Olcha *mandu̡* '1', Orok *mandi̡* '1', Na. *mandu̡* '1', Solon *mandê*:, *mandi*: 'very, heavily'.
pMo **man* 'big, high' in pMo **man(-)da*- ~ *mandu*- 'to become big, high': MMo. *mandu*- 'to increase, develop', WMo. *mandu*- ~ *mantu*- ~ *manda*- 'to rise (sun, moon, flame), become higher, tower, spread, develop (intr.)', *manduɣur* 'big, fat', Khal. *mandgar*, *mantaj* 'big, fat', Bur. *mandagar*, *mantan*, *mantagar* 'big, fat'
pTk **banï* 'big, large': Chu. *mănă*

The Old Japanese verb *samane*- 'be many' probably contains the prefix *sa*-, which Yanagida and Whitman (2009) describe as a suffix marking inactive intransitive verbs, taking non-agentive and mostly non-human subjects. The simplex verb OJ *mane*- has B register, which is incongruent with the register of the Ryukyuan verbs. However, the assignment of a verb or adjective stem to type A or B is not always straightforward (see Robbeets 2015: 132). If this is the case here, the Ryukyuan forms reflecting **manki*- 'to be big' may be a contraction of the adjective root **mana*- 'to be

many, big' and the de-adjectival adnominal ending *-*ki*, giving rise to the voiced velar stop.

The Korean form MK *¨man·ho-* 'be many' and the derived adverb K *ma:nhi*, MK *¨man·h-i* 'many' are not considered here because *¨man* goes back to a Sino-Korean adjectival noun *¨MAN* 'myriad'.

The reconstruction pTg **mandi* 'crowdy' can be derived from pTg **mani* 'crowd' and the suffix pTg *-*di* common in the derivation of denominal property nouns such as pTg *xug-di* 'wide' (Olcha *xugdi*, Orok *xugji* , Na. *xugji*), pTg **bug-di* 'variegated'' (Evk. *bugdi*, Even *bu:di*, Orok *bugji*), pTg **peg-di* 'big' (Evk. *hegdi*), pTg **pa:* 'part' → *pa:-di* 'separately' (Evk. *ha:di* 'some part of', Even *ha:di(n)* 'some, other', Olcha *pa:ji*, Na. *pa:ji*, Orok *padi* 'separately', Solon *adĩ* 'some'), pTg **loko* 'thick' (Evk. *luku*, Even *no:ɣ*, Neg. *loxo*) → *lo:g-di* 'thick' (Evk. *lo:gdi*, Even *nụqtị*, Neg. *logdị*).

The Mongolic forms may include the fientive suffix pMo *-*dA*- which derives verbs with the meaning 'to become like the base' from property nouns, e.g. WMo. *idermeg* 'chip→ *idermegde*- 'to become chipped' (Robbeets 2015: 305). This leads to the reconstruction of pMo **man* 'big, high'.

The Turkic cognate is limited to Chuvash, the only surviving language of the Western Turkic branch. If this poor distribution indicates borrowing, the word should be borrowed at a prehistorical stage, when the proto-Mongolic property noun was still in use. However, the root-final vowel in Chuv. *mănă* 'big' is not mirrored in the Mongolic form and argues against borrowing.

Other instances in which Chuvash initial *m*- derives from pTk **b*- when followed by a nasal are among others Chuv. *miŋ*, Tat. *biŋ* 'mole on the face' (< pTk **beŋ*), Chuv. *măy*, OT *boyïn* 'neck' (< pTk **bo:ñïn*), Chu. *măyăx*, OT (Karakhanide) *bïyïk* 'moustache' (< pTk **bïñïk*).

pTg **amban* 'big': Olcha *amba(n)* '1 big, large; 2 very', Orok *ambaramjị* '2', Oroch *amba* '2', Na. *amba(n)* '1', Sibe *amə* '1', Ma. *amba* '1', Jur. *amban* (*amban-an*) '1', *amban-lar* 'many'
pMo **amban* 'big, large': WMo. *amban* '1 big, large; 2 minister, governor, dignitary', Khal. *amban* '1, 2', Bur. *amba* '1, 2', Kalm. *ambn* '1, 2', Mgr. *amba* '2'

33. ONE
pK **pilɨ*- ~ **pilʌ*- 'to begin': K *piloso* 'for the first time (adv.)', K *pilos ha*- 'to start, begin', *piluc*- 'to start having labor pains', MK *pi·lus* ~ *pi·los* 'from the beginning, beginning (adv./ adj. n.)'; K -*(u)s* < pK *-*s* deverbal suffix deriving nominal adjectives and adverbs, e.g. K *cilki*- 'to be tough'→ *cilkis* 'firm' (Robbeets 2015: 422-423)
pTk **bi(:)r* 'one': OT *bir*, Tk. *bir*, Tat. *ber*, Az. *bir*, Tkm. *bir*, Gag. *bir̍*, Uz. *bir*, Uig. *bir*, S-Yug. *bɨr*, Khak. *pər*, Shor *pir*, Tuva *bir*, Tofa. *bir*, Yak. *bi:r*, Dolg. *bi:r*, *bir*, Karaim *bir* ~ *bïr*, Kirg. *bir*, Kaz. *bir*, Nog. *bir*, Bash. *ber*, Balk. *bir*, Kpak *bir*, Salar *pyr*, *pir*, *pur*, Kum. *bir*, Khalaj *bi*, Chu. *pĕr*

36. HIT/BEAT
pJ **tuk*- 'to pound, hit with force': J *tuku* B*,* OJ *tuk*- 'to pound, husk, beat, hit with force'; Shuri (Okinawa) *cicun*
pK **t(ʌ)hi*- < **t(ʌ)ki*- 'to hit, strike': MK *·thi*- 'to hit, strike',
pTg **dug*- 'to hit with force': Evk. *dug*- '1 to hit, beat, hammer', Even *duɣ*- '1', *dụɣ*- '2 to batter, hit repeatedly', Neg. *dụw*- ~ *dụɣ*- '2', *dukte*- '1', Olcha. *dụ:čï*- '2', Orok

du: 1, *dụ:čï-* '2', Na. *du:-* 1, *do:čï-* '2', Oroch *du:-* '1, 2', Ud. *du:-* '2', *dukte-* '1', Ma. *du-* ~ *du:-* '1, thresh', Jur. *du-ŋu-mij* '1'

According to Ramsey's law (cf. 32. BIG) the reconstruction of a minimal vowel in pK **tʌhi-* is legitimate. . The correspondence between Chinese donorwords and Korean loanwords (e.g. Ch. *cak* 'foot (measure)' is borrowed as MK · *cah*), phonogram readings in the Kyelim Yusa (e.g. *¨hwalq-huy* for MK *holk* 'earth'), elements in Paekche placenames (e.g. *tin-¨qak* for MK *¨twolh* 'stone'), dialectal forms (e.g. dial. *tolk* for MK *¨twolh* 'stone'), and internal doublets (e.g. MK *siphu-* versus MK *sikpu-* 'want') all suggest that velar lenition (**k* > **h*) took place at an early stage in Korean (Martin 1996: 36-37). This observation supports the reconstruction of pK **tʌki-*. Although the semantic and formal correspondences among the Japanese, Korean and Tungusic participants are very close, we cannot exclude the possibility that we are ultimately dealing with a sound symbolic formation.

37. LEG/FOOT

pJ **panki* 'foot, (lower) leg': J *hagi* (?2.3), OJ *pagi*$_1$ 'shin, shank', Yamatohama (Amami) *hagi*, Yoron (Amami) *pagi*, Shodon (Amami) *hagi* 'foot', Hirara (Miyako) *pag*^{z}*ï* 'leg', Ishigaki (Yaeyama) *paŋ* [paɴ] 'leg, foot' (B), Hateruma (Yaeyama) *paŋ* 'leg, foot', Hatoma (Yaeyama) *paɴpisa*,Yonaguni *haŋ* 'leg', pR **pagi* 'foot, leg'; OKog **ɦa* 'foot' (Beckwith 2007: 111).

pK **pal* 'foot, leg' ~ **pʌlk* 'arm': K *pal*, MK *·pal* 'foot, leg, paw, extremity', K *phal*, MK *polh* 'arm'

pTg **palgan* 'foot': Evk. *halgan* '1 foot', Even *halgen* '1', Solon *alɣã* '1', Neg. *xalgan* '1', Olcha *palja(n)* '1', Orok *palja(n)* '1', Nan. *palgã* 'sole', Oroch *xaga* 'paw', Ud. *xaga* 'paw; bear's trace'

I do not exclude that the original root was pK **pʌl* 'limb, leg, foot' and that **pʌl-k* 'arm' is a diminutive derivation in the sense of 'small limb'. The pK diminutive suffix **-(a/e)k* occurs frequently in body parts, e.g. *muluph* 'knee' → *muluphak* 'knee', *thel* 'hair' *thelek* 'hair'. The reduced vowel in pK **pʌl* 'limb, leg, foot' may also be reflected in MK *¨polW-* 'to tread' (*¨ polp- / pol ·W[o]-* < pK **pʌ·lʌ· pʌ-* 'tread') but this derivation is problematic because of the accentual discrepancy.

39. THIS

pJ **i* 'you' (derogatory second person pronoun): OJ *i* 'you' (derogatory second person pronoun)

pK **i* 'this' (demonstrative pronoun of the proximal plan): K *i* , MK *i* 'this' (demonstrative pronoun of the proximal plan)

pTg **i* 'he, she' (3 SG pronoun): Ma. *i* 'he, she' ~ *i-n-* 'he, she (oblique)', *ineku* 'same, this (day, month, year); likewise, in the same way', Sibe *i:* 'he, she', Jur. *in* 'he, she', Solon *ini* 'his, her'

pMo **i* 'he, she, it' (3 SG pronoun) in **i-nu-* (3 SG-genitive) and **i-ma-* (3 SG-oblique): MMo. *ino* (SH) ~ *inu* (HY, Muq) '1 his, her, its' (3 SG possessive pronoun), MMo. *ima-* '2 he, she, it' (3 SG pronoun in oblique cases) 'WMo. *inu* '1', Dag *i(:)n* '1', yam '2', Khal. *ń* '1', Bur. *ń* '1', Kalm. *ń* '1', Mog. *ini* ~ *ni* ~ *ne* ~ *i* '1'

pTk **ï(-)n-* 'that' (demonstrative pronoun of the distal plan): OT *ïnča* 'the following, in the following way' (vs. *anča* 'the previous, in the previous way'), OT *ïntïn* 'the one on the other side' (vs. *muntïn* 'the one on this side'), OT *ïnaru* 'forward;

from ... on' (vs. *kerü* 'backward', OT *ïna* demonstrative interjection (vs. *muna*), OT *ïnčip* ~ *inčip* 'that having happened' (Erdal 2004: 206-207); Tkm. *ɨnaru* '1 forward, onward', Tuva *ɨnda* `there', *ɨndiɣ* 'such', Tofa. *ɨnda* `there', Khalaj *ína* 'that'

As a pronoun OJ *i* is used in reference to a derogatory second person pronoun, but the internal evidence for the reconstruction of pJ **i-* as a lexicalized demonstrative pronoun of the proximal plan following Whitman (1985: 217, 246) and Starostin et al. (2003: 577) is rather poor. It solely relies on the derivation of *ima* 'now' from the demonstrative **i-* followed by *ma* 'room, space'. The comparison with Ryukyuan *nama* 'now' suggests that *ma* 'room, space' is indeed the second member of the compound and Ryukyan *koma* 'here' and *kama* 'there' further suggest that the first member is a demonstrative, but unfortunately there are no other lexicalizations in which OJ *i-* and Ryukyuan *na-* occur as petrified proximal demonstratives.

The Mongolic pronouns *inu* 'his/her/its' and *anu* 'their' are the genitive forms of the ancestral pronouns **i* 'he, she, it' and **a* 'they'. The same vocalic opposition between singular and plural pronouns is found between **bi* 'I' vs. **ba* 'we' and **ci* 'you (SG)' vs. **ta* 'you (PL)'.

The Japanic, Koreanic and Turkic languages bear traces of a petrified demonstrative use of this pronoun. Since the development from a demonstrative into a personal pronoun is well attested (e.g. French personal pronoun *il* 'he' is derived from the Latin demonstrative *ille* 'that'), but the change in the other direction is not (Heine & Kuteva 2002: 112-113), it is inviting to reconstruct a proximal demonstrative 'this' in the ancestral language.

pJ **kɨ-* 'this' (demonstrative pronoun of the proximal plan): J *ko-* (1.1), OJ ko_2- 'this'; Yuwan (Amami) *ku-rɨ* 'this', *ku-ma* 'here', Shodon (Amami) *k'u-r*, Shuri (Okinawa) *ku-ri* 'this person, thing', *ku-ttaa* 'these people', *ku-ma* 'here', *ku-nu* 'this', Hirara (Miyako) *ku-i* 'this', Ishigaki (Yaeyama) *ku-ri* (A) 'this', Hateruma (Yaeyama) *ku-ri* 'this', Dunan (Yonaguni) *khú* 'this', *khú-ma* 'here', pR **ko-* 'this' (proximal demonstrative)
pK **kɨ-* 'that' (demonstrative pronoun of the mesial plan): K *ku-*, MK *ku-* 'that'
pTk **kö* 'this' (demonstrative pronoun of the proximal plan): Chu. *ku* 'this', S-Yug. *gu*, *go*, Salar *ku*

pJ **ə-* 'that' (demonstrative pronoun of the mesial plan): J *ore* 'I (first person pronoun), OJ *ore* 'self; you derogatory 2sg', OJ ono_2 'self'; Shuri (Okinawa) *ʔu-ri* 'that person, thing', *ʔu-ttaa* 'they', *ʔm-ma* 'there', *ʔu-nu* 'that' (mesial demonstratives), Amami *u-rɨ* 'that', *u-ma* 'there' (mesial demonstratives), Hateruma (Yaeyama) *u-ri* (distal demonstrative), Yonaguni *ú* 'that', *ú-ma* 'there' (mesial demonstrative)
pMo **e-* 'this' (demonstrative pronoun of the proximal plan) in pMo **e-ne* (this-SG) 'this': MMo. *ene* (SH/HY/Muq), WMo. *ene*, Khal. *ene*, Bur. *ene*, Ordos *ene*, Kalm. *en*, Dag. *ənə*, Eastern Yugur. *ene*, Mgr. *ne*, *ni*, Bao. *enə, nə,* Dong. *ənə,* Mog. *enä*; in pMo **e-de* (this-PL) 'these': MMo. *ede* (SH/HY), WMo. *ede(n),* Khal. *ed(en),* Bur. *ede*, Kalm. *edn*, Dag. *əd(ən)*; in pMo **e-n-de* (this-SG-LOC) 'here': MMo. *ende* (SH/HY/Muq), WMo. *ende*, Khal. *end*, Bur. *ende*, Kalm. *end*, Dag. *ənd*, Eastern Yugur. *ende*, Mgr. *nde*, Bao. *endə,* Dong. *əndə*, Mog. *endä*
pTg **e-* 'this' (demonstrative pronoun of the proximal plan): Even *ere* 'this, here', *ecin* 'in this way, indeed', *eduk* 'since this time, from here', *ele:* 'here', *eweski*: 'in this direction', *ewgi:le:* 'here', *ewgi:n* 'here', Evk. *er*, *eri* 'this', *ewgi:* 'in this direction', *ele:* 'here', *eduk* 'since this time, from here', Solon *eye: ~ er ~ eri* 'this', *elur* 'these', Neg. *ele: ~ eli:* 'in this direction, here', *ewuli*: ~ *ewule:* 'here', *ewgi:le: ~ ewgi:li:* 'in this

direction', *edu:* 'here, at this time', *eye* 'this', Ud. *ebede* 'like this', *eyi* 'this', Olcha *yedu* 'here', *yeji* 'with this', *yeki* 'here', *yele* 'from here', *ei* 'this', *e:wu* 'this', Na. *eyebe ~ yebe* 'here', *ei* 'this', Orok *edu ~ eyedu* 'here', *ewwe*, *eye*, *er*, *eri* 'this', *emete(n)* 'such, like this', Ma. *ere* 'this', *embici* 'or' (-*bi-ci* be-COND.CONV), *eici* 'or', Sibe *erə* 'this', Jur. *e(r)se* 'this'

In addition, Savelyev (2021 in press) reconstructs an emphatic demonstrative Proto-Turkic **e*- retained as an archaism in the Bulgharic branch: PTk **e*- + **kö* (demonstrative) > Chuv. *agə̂* 'this'; PTk **e*- + **bu* (demonstrative) > Chuv. *avə̂* 'that'; and PTk **e*- + **bV* (1SG pers. pron.) > Old Chuvash (18-19th Century) *a-bə* > modern Chuvash *ebə* 'I'. He assumes that the latter development is taking place in order to restore vowel harmony. A parallel development can be found in or Old Chuvash *a-zə* > Chu. *ezə* 'you sg.', OChu. *abir* > Chu. *ebir* 'we', and OChu. *azir* > Chu. *ezir* 'you pl.'.

40. FISH

pJ **(y)iwə* 'fish': J *uo* (2.1), OJ *iwo* 'fish', Yamatohama (Amami) *ʔjuu*, Asama (Amami) *ʔyuu*, Yoron (Amami) *ʔyuu*, Shodon (Amami) *ʔyuu,* Yonamine (Okinawa) *ʔyuu,* Shuri (Okinawa) *ʔiyu* (A), Irabu (Miyako) *ïzu*, Hirara (Miyako) *ᶻïzu*, Ishigaki (Yaeyama) *idzu* [*izu*] (A), Yonaguni *iyu*, pR **iyu* 'fish'

pMo **diɣa*- 'fish': MMo. *jiqasun* (SH/HY) ~ *jiɣasun* (Muq) '1 fish', *jixuči* 'fisher', WMo. *jiɣasu(n)* '1', Khal. *zagas(an),* Bur. *zagaha(n),* Ordos *jaɣasu*, Kalm. *zaɣsn*, Dag. *jagas*, *jaus*, Eastern Yugur *jaɣasən ~ jəɣasən*, Mgr. *ʒ́aġasə ~ ʒ́iġasə ~ jagarsï*, Bao. *ʒilɣasuŋ,* Dong. *ǯağasun*

For the neutralization of pJ **yi* and *i* as OJ *i*, see 26 house.

The Mongolic deep velar consonant with velar origin WMo. *ɣ* < **g* only occurs in stems with back vowels. In intervocalic position, it converged with the deep-velar consonant with bilabial origin WMo. *ɣ* < **β* < **p*/**b* (Poppe 1955: 98). In cases like this, where *ɣ* occurs in stems with back vowels, the origin of the consonant can be either velar or labial.

43. BLACK

pMo **kara* 'black': MMo. *qara* (SH/HY/Muq), WMo. *qara* 'black, dark, obscure', Dag. *xar*, Khal. *xar*, Bur. *xara*, Ordos *xara*, Kalm. *xar*, Eastern Yugur *xara*, Mgr. *xara ~ qara*, Bao. *χəra*, *χara*, Dong. *qara* 'black, dark', Mog. *qara: ~ qaro: ~ qʌrɔ*

pTk **kara* 'black': OT *qara,* Tk. *kara*, Tat. *qara*, Az. *ɣara*, Tkm. *ɣara*, Gag. *qara*, Uz. *qɔrɛ*, Uig. *qara*, dial. *qare*, Khak. *xara*, Shor *qara*, Tuva *qara*, Tofa. *qara*, Yak. *xara*, Dolg. *kara*, Kirg. *qara*, Kaz. *qara*, Nog. *qara*, Bash. *qara*, Balk. *qara*, Karaim *qara*, Kpak. *qara*, Salar *ɣara*, Kum. *qara*, Khalaj *qara*, Chu. *xora*

In addition to the neutral color term *xar* 'black', Dagur also has *kara* 'black (horse colour)', which is a borrowing from Tungusic (Nugteren 2007: 404). The Tungusic terms Evk. *kara:* and Ma. *qara* are specialized to denote the horse colour and so is MK *kala* (*mol*) 'black horse'. This observation suggests that the Tungusic and Korean terms were borrowed in the context of the spread horse-ridden pastoralism, while the Turkic and Mongolic neutral color terms are cognates.

45. STAND

pJ **tata*- 'to stand': J *tat*- (B) 'to stand, rise, run high (intr.)', OJ *tat*- 'stand, be built; leave; (time) pass, elapse', J *tate*- (B), OJ *tate*- 'to stand, erect (tr.)'; Yamatohama (Amami) *θaθuri*, Asama (Amami) *tacjuɴ,* Yoron (Amami) *tacjuɴ,* Shodon (Amami) *that*- 'to stand (intr.)', Yonamine (Okinawa) *tacuɴ,* Shuri (Okinawa) *tat*- [*tacuɴ*] (B), Irabu (Miyako) *tacï ~ tafu*, Hirara (Miyako) *tḁtsï*, Ishigaki *tasuŋ* [*tacuɴ*], Yonaguni *tatuŋ*, pR **tat*- 'to stand (intr.)'

pK **tʌtʌ*- 'to run': K *tataT*-, MK *tatoT*- 'reach, attain, get to' (< *ta(h)*- 'reach' + *toT*- 'run'), K *taT*-, MK *toT*- 'to run, rush'

pK **ilʌ*- ~ *ilɨ*- 'to become, come up': K *i:l*- 'to come up, spring up, rise, happen (intr.)', MK *il-/¨i(l)*- 'to become, come up'
pTg **ili*- 'to stand up, rise': Evk. *il*- '1 stand up, 2 stand still, stop', Even *ịl*- '1, 2', Neg. *ịl*- '1, Solon *il*- '1 stand up' , Oroch *ili*- '1, 2', Ud. *ili*- 'stand', Olcha *ịlị*- '1, 2', Orok *ili*- '1, 2', Na. *ịlị*- '1, 2', Ma. *ili*- ~ *ile*- '1,2', Sibe *ila*- ~ *ili*- '2', Jur. **ili*- 'rise'

46. BITE

pJ **kam*- 'to bite, chew': J *kamu* (B)*,* OJ *kam*- 'to bite, gnaw, chew, masticate, eat'; Yamatohama (Amami) *xamuri* 'to eat', Asama (Amami) *kamyu*ɴ 'to eat', Shuri (Okinawa) *kamu*ɴ 'to eat', Hirara *kam* 'to bite', Ishigaki *kamuŋ* 'to bite', Yonaguni *kamuŋ* 'to bite'*,* pR **kamu*- 'to bite, eat'
pMo **keme*- 'to bite' (+*-*lA-/* *-*li*- intensive-iterative suffix): MMo. (Muq) *kemile*- 'to gnaw', WMo. *kemeli*-, *kemele*- '1 to gnaw, nibble, crack with one's teeth (tr.)', *kemki*- '2 to bite, snap with the teeth (tr.)', Khal. *ximle*-, *xemle*- '1', Bur. *ximel*- '1', Bur. (Bargu dial) *ximil*-, Kalm. *keml̥*- '1', Ordos *kemele*- '1', *kemχel*- '2', Bao. *kamel*, Bao. (Dahejia dial.) *kaməl*- 'to bite', Dag. *keme*- '1', Eastern Yugur *kemle*-, *kelme*- '1'
pTk **kem*- 'to bite, chew (intr.)' (+ *-*(U)r* causative): OT (Karakh.) *kemür*- '1 to gnaw, chew (tr.)', Tk. *gemir*-, *kemir*- '1', Az. *gämir*- '1', Tkm. *gemir*- '1', Gag. *kemir*- '1', Uz. *kemir*- '1', Uig. *kemi(r)*- '1', Tat. *kimer*- '1', Khak. *kimər*- '1', Karaim *kemir*- '1', Kirg. *kemir*- '1', Kazakh *kemir*- '1', Nog. *kemir*- '1', Bash. *kimer*- '1' Balk. *kemir*-, Kpak *kemir*- , Kum. *gemir*-, Tuva *xemir*- '1', Tof. *xemir*- '1'

In his review of Robbeets (2005), Georg (2007: 273) objects: "Had they used more scientifically oriented sources […] or any Mongolistic expertise for a change, they would have found the *meaning* of this verb to be 'to crack open a bone with one's teeth and to suck the marrow', which makes clear that it is derived from *kemi* 'marrow of bones' and has to be eliminated from this "etymology"." However, these Mongolic forms can be analysed in two different ways: whereas Georg derives them from pMo **kemi(n)* 'marrow of the bones', I derive them from pMo **keme*- 'to bite'. Thus, I take the general meaning 'to bite' as the primary one and assume that the peripheral attestation of MMo. *kemi-le*- is a case of metathesis. Both -*lA*- and -*li*- are attested as deverbal iterative-intensive suffixes in Mongolic. The intensive-iterative pMo *-*lA* is frequently lexicalized in verb pairs such as WMo. *alqu*- 'to step, walk (intr.)' *alqula*- 'to march, walk with quick steps (intr.)', WMo. *seji*- 'to butt with the horns' → *sejile*- 'to butt repeatedly with the horns', WMo. *ili*- 'to caress, stroke'→ *ilile*- 'to touch or stroke repeatedly'. However, the suffix *-*lA*- in Georg's analysis may also be the denominal verb suffix, e.g. WMo. *šibaɣun* 'bird' → *šibaɣu-la*- 'hunt birds'. The suffix *-*ki* in WMo. *kemki*- 'to bite, snap with the teeth (tr.)' can be explained either as a deverbal transitivizer or as a denominal verb formant; the second explanation based on Georg's analysis, is more problematic, however, since *-*ki*- is a

grammaticalized form of MMo. *ki-* ‘to make’ with the meaning ‘to make the verb base’, e.g. WMo. *saγad* ‘hindrance’ → *saγadki-* ‘to hinder’. The expected meaning of the derived verb would thus be ‘to make marrow’ rather than ‘to bite’. In the present analysis, WMo. *kemki-* ‘to bite, snap with the teeth (tr.)’ reflects a deverbal transitivizer pMo *-*ki*, lexicalized in verb pairs such as WMo. *kel-* ‘to be strung (as pearls) (intr.)’ → *kelki-* ‘to string pearls (tr.)’. Furthermore, the final vowel in all contemporary attestations reflects -*e*- rather than -*i*-, which suggests that **keme-* is the primary form.[5]

According to Clauson (1972: 723), the Turkic transitive verbs meaning ‘to gnaw, chew’ can be derived as a causative of pTk **kem-*. The causative suffix *-*(U)r* is lexicalized in Turkic verb pairs such as OTk. *ač-* ‘to be hungry’ → *ačur-* ‘to starve (tr.)’, OTk. *keč-* ‘to be late (intr.)’ → *kečür-* ‘to delay (tr.)’ (Erdal 1991: 710-726).

pK **mɨlɨ-* 'to bite (tr.)': K *mul-*, MK (¨)*mu(l)-/ mu·lu-* 'to bite (tr.)'
pTg **mödö-* 'to gnaw, nibble, bite': Evk. *mudu-* ~ *modo-* '1 to gnaw, nibble, bite, 2 to tear with the teeth', Even *mud-* '1, 2, to bite to death', Orok *mudji-* '1'

The reconstruction of the vowel pTg **ö* in Tungusic is based on the *u*~*o* alternation in Evenki, even if the vowel *u* in Even and Orok suggests the reconstruction of pTg **u* in this from; see footnote 2.

50. WHAT?
pJ **ka* (wh-interrogative particle): J *ka* interrogative particle in yes/no and wh-questions, OJ *ka* interrogative particle used in wh-questions; Shuri (Okinawa) *ga* wh-interrogative particle, Tarama (Miyako) *ga* wh-interrogative particle, Yonaguni *nga* wh-interrogative particle, pR **ga* wh-interrogative particle
pK **ka* (interrogative particle): K *ka* interrogative particle in yes/no and wh-questions, MK *·ka* interrogative particle mainly used in yes/no questions
pTg **xa* (wh-interrogative pronoun) in pTg **xa-i* ‘who, which one’: Evk. *i:,* Even *i:*, Solon *i:*, Ud. *i:*, Olcha *xay*, Na. *xay*, Ma. *ay*, Sibe *ai*; in pTg **xa-ba-siki:* ‘where to’: Evk. *awaski:,* Even *awaski: ~ awuski:*, Neg. *awaski*, Oroch *awasi*, Olcha *xawasi*, Na. *xaosi,* Ma. *absi;* in pTg **xa-li:* ‘when’: Na. *xali*, Ud. *ali*, Neg. *ali*, Evk. *ali:*; in pTg **xa-du:* ‘how much’: Evk. *adi:*, Even. *adi:* Na. *xadu*, Olcha *xadu*, Ud. *adi*, Neg. *adi*; pTg **xa-son* ‘how big, how many’: Evk. *asun*, Even *asun,* Neg *asun*, Na. *xasun*, Olcha *xasun* and in pTg **xa-oni* ‘how’: Evk. *o:n*, Even *o:n* Na. *honi*, Olcha *xon*, Ud. *ono* (Benzing 1955a: 114)
pTk **ka* (wh-interrogative pronoun) in OT *kanï* ‘where?’ (< **ka*-ACC), *kaŋa* ‘to which place’ (< **ka*-oblique-DAT), *kanta* ‘where’ (< **ka*-oblique-LOC), *kantan* ‘from where’ (< **ka*-oblique-ABL), *kanča* ‘how much, how far, by which way’ (< **ka*-oblique-equative), *kač* ‘how much’, *kañu* / *kayu* ‘1 which’, Tkm. *qay* ‘1', Uz. *qay* ‘1', Uig. *qay* ‘1',Tat. *qay* ‘1', Kirg. *qay* ‘1', Bash. (dial.) *qay* ‘1', Tuva *qayɨ* ‘1', Tofa. *qai* ‘1', Kaz. *qay* ‘1', Yak. *xaya* ‘1', Dolg. *kaya* ‘1', Khalaj *qa:yan* 'whereto' (< **ka*-DAT + *yan* 'side')

Vovin (2008: 128-129) rejects the comparison of the interrogative markers MK *˙ka* and OJ *ka,* noting the fact that MK *·ka* marked yes/no questions, whereas the Old

[5] Dagur has a verb *kəmʸ-* ‘to ruminate, chew the cud’, which reflects a final high front vowel. However, in view of the meaning of this form, it is probably a reflex of pMo. **kebi-* ‘to chew, to ruminate’ (Nugteren 2011: 407.)

Japanese *ka* usually marked wh-questions. It can be objected, however, that there are instances in which MK *·ka* does marked wh-questions, e.g. MK *¨nwu-·lul ka·colpi-lq ·ka* [who-ACC compare-ADN INT] 'Whom would one compare [with him]?' (Martin 1992: 863) as well as instances in which OJ *ka* marked yes/no questions, e.g. OJ *tano*$_1$*si-ku mo*$_2$ *ar-u ka* [delightful-INF PT exist-ADN INT] 'Isn't it delightful?' (KK 54; Vovin 2009: 1224). In both Old Japanese and Middle Korean the interrogative *ka* was preceded by a noun or adnominal form of the verb and the Korean form does not display vowel harmony. Therefore, the Japanese and Korean particle seems to go back to an original independent interrogative pronoun. The pronominal origin of the interrogative marker is further supported by the observation that the Old Japanese interrogative is not restricted to sentence-final position, e.g. OJ *iku yo*$_1$ *ka ne-t-uru* [how.many night INT sleep-PERF-PCP] ‘How many nights have we slept?’ (KK 25; Vovin 2009a: 1220). In sum, the seemingly contrastive situation in Old Japanese and Middle Korean may well be the result of a historical change and does not stand in the way of tracing the origin of the interrogative markers back to an original interrogative pronoun in the ancestral language. This observation makes the comparison with the Altaic interrogative pronoun even more plausible.

It can be noted that the Mongolic languages share an interogative root pMo **ka* 'where?': MMo. *qa'a ~ qa:na* 'where?', WMo. *qa ~ qana ~qamiɣa ~ qaɣa* 'where' (WMo. *-GA* derives local and temporal adverbs; *-A* dative-locative), Khal *xaa*, Bur. *xaa*, *xaana*, Kalm. *xa ~ xama* (*-ma* indefinite in **yama* 'something', *kemüge* 'something'), Dag *xa:nə*, Eastern Yugur *xana*, Bao *xalə ~ xali ~ hala*, Dong *qala*, Mog. *qana*. However, as the semantics remain restricted to the local interrogative, I suspect that the forms may represent early borrowings from Turkic. From the functional viewpoint, the general interrogative interrogative pronoun pMo **ke* is a much more plausible cognate, but here the vowel does not fit. The interrogative is reflected as pMo **ke* ‘what’ in MMo. *ja’u ke* ‘whatsoever’; pMo **ke-n* ‘who (SG)’, **ke-d* ‘who (PL)’ in MMo. *ken* (ket PL), WMo *ken* (ked PL), Dag *keŋ*, Mgr. *kän*, Mog. *ken*, Urd. *ken*, Kalm. *ken*, Khal, Bur. *χeŋ*; pMo **ke-r* ‘how’; pMo. **ke-li*: ‘when?' and pMo. **ke-gün* ‘something’in Kalm. *kü:n*, Urd *küm*.

51. CHILD

pJ **wara-pa* 'child': J *warawa* (3.5b), OJ *warapa* 'child'; Yamatohama (Amami) *warabï*, Asama (Amami) *warëë*, Yoron (Amami) *warabi*, Shodon *warabï*, Shuri *warabi*, Nakijin (Okinawa) *warabii*, Irabu (Miyako) *yarabi*, Ishigaki *yarabi ~ meerabi*, Hatoma (Yaeyama) *yarabi*, Yonaguni *myaarabi*, pR **wara-be* 'children' ~ *me-wara-be* 'girl-child'

pTk **ba:la ~ ba:la-pan* ‘young animal, child': OT (Karakh.) *bala* '1 young animal, nestling, 2 child', Tk. *bala* '1, 2', Az. *bala*, *balaɣ* '1, 2', Tkm. *ba:la* '1, 2', Uz. *bɔla* '1, 2', *palapan* (dial.) '1' , Uig. *bala* '1, 2', Tat. *bala* '1, 2', Karaim *bala* '2', Nog. *bala* '2', Bash. *bala* '2', *belekej* 'small', Balk. *bala* '2', Kpak *bala* '2', Salar *bala* '1, 2', Kum. *bala* '1, 2', Kirg. *bala* '2', Kaz. *bala* '2', *balapan* '1', S-Yug. *mɨla*, *mle* '2', Khak. *pala* '2', Shor *pala* '2', Khalaj *bala* '2'

All terms for 'child' participating in this etymology are not kinship terms but refer to a certain age-group. In the Ryukyuan languages, *-bi* < *pR **-be* is a collective suffix for persons, e.g. Chabana (Yoron) *wuhii-bi* 'brother' (Thorpe 1983: 269).

pTg **puri* 'child, offspring': Even *hurel ~ urul ~ urel* '1 child, infant, 2 grandchild, 3 younger brother or sister', *hurken ~ urke:n ~ hurkan* '4 adolescent, 5 young (person),

6 single (person)', Evk. *hurkan ~ urkan ~urke:n ~ hurke:ke:n*, *urkeke:n* '6, 7 young boy, 8 son', Solon *ukkēxẽ̄*, *urkēxẽ̄* '4, 5, 7, 8', *uril* '1, 7, 8', Neg. *xujil* '1, 8, 9 daughter', Olcha *purul* '1, 8, 9', Orok *puril* '1, 8, 9', *puriɣe ~ pure:* '5', Na. *puril* '1, 8, 9', *puri ~ furi* 'family', Oroch *xi:* 'children, descendants'
pMo **püre* 'offspring, seed, fruit': MMo. *xüre* (HY), *hüren* (Muq) '1 offspring, descendant, seed, fruit, result', WMo. *üre* '1', Dag. *xur*, *hure* '1', Khal. *ür* '1', Bur. *üre* '1', Ordos *ür*, *üre* '1', Kalm. *ürn* 'child, offspring, seed, fruit, result', Eastern Yugur *hure* '1', Mgr. *fure*:, *fure*, *furie*:, *xuru*, *xure*:, *xurie*, Bao. *fure*, *fərɛ*, Dong. *fure*

The final liquid in the Tungusic forms is a petrified plural suffix pTg *-*l*, e.g., Evk. *ŋa:le* 'hand' → *ŋa:le-l* 'hands', Neg. *oyo* 'reindeer' → *oyo-l* 'reindeers', Even *adal* 'net' → *adal-al* 'nets' (Benzing 1955a: 1023-1026). The Tungusic forms in *-*kan* reflect a lexicalized diminutive suffix pTg *-*kA:n*, e.g. Even *ŋa:l-ka:n* 'small hand', Orok *ke:či-ke* 'puppy', Na. *toke-kan* 'small sleigh', etc. The Manchu form *fursun* 'breed' is probably a borrowing from Mongolic as -*-*sUn* is a collective and body part suffix in Mongolic (see 7. BLOOD) but not in Manchu (Rozycki 1994: 83).

53. GIVE
pJ **tama*- 'to give': J *tamaw*- (B), OJ *tamap*- 'to give, bestow on', J *tamawar*- (B) 'to be given', J *tabe*- (B) 'to eat', OJ *tab*- 'to deign to give', OJ *tabar*- 'to humbly receive, give (to me)'; Yamatohama (Amami) *θaboruri* 'be given from above (only used as an auxiliary)', Asama (Amami) *taboojui* be given from above', Yoron (Amami) *tabeN*, *tabaw*- 'be given from above', Nakijin (Okinawa) *taboori* 'please (give me) (imperative only)', Shuri (Okinawa) *tabir*- 'give', Irabu (Miyako) *tabori* 'please (give me) (imperative only)', Ishigaki (Yaeyama) *tabooruN* 'be given from above', Hatoma (Yaeyama) *tabooruN* 'be given from above'.
pTg *tama*- 'to pay': Evk. *tama*- '1 to pay', Even *tam*- '1', Neg. *tama*- '1', Solon *tama*- '1', Olcha *tama*- '1', Orok *tama*- '1', Oroch *tama*- '1', Ud. *tama*- '1', Na. *tamã* 'price'

pJ **(w)ura*- 'to sell': J *ur*- (A), OJ *ur*- 'to sell', Naze (Amami) *uryuŋ* 'to sell', Shuri (Okinawa) *uyuŋ*, Nakasuji (Miyako) *ʔvi(i),* Yonaguni *uruŋ,* pR **uri*- 'to sell'
pK **pʌlʌ-kʌ*- 'to sell': K *phal*-, MK *pho(l)- /·pho·l(o)*- 'to sell'
pTg **bu:*- 'to give': Evk. *bu:*-, Even *bö:*-, Neg. *bu:*-, Solon *bu:*-, Olcha *bu:wu*- Orok *bu:*-, Na. *bu:*-, Oroch *bu:*-, Ud. *bu:*-, Sibe *bu*-, Ma. *bu*-

The early drop of the initial labial glide before the rounded u in Japonic may have blocked conditioning factor 39b, which would be expected to yield Japonic ***wauru*- > ***waru*-. Note however, that Martin (1996: 76) suggests that pJ **wara*- 'to split, break' is related to pJ **(w)ura*- 'to sell', which would be in line with 39b.

The Koreanic form involves a deverbal inchoative suffix pK *-*kɨ*- ~ *kʌ* (Robbeets 2015: 256-258). The suffix derives verb pairs such as pK **ulɨ*-'to cry' in MK ¨*wul*- 'to cry (intr.)' → MK *wulG*- 'to howl, roar, shout loudly (intr.)' (< pK *ulɨ-ɣɨ*- < **ulɨ-kɨ*-) and MK *nul*- 'to increase, be(come) longer, be better' → MK *nulk*- 'to be old, grow old (intr.)' (< pK **nɨl-kɨ*-).

Similar to the etymologies under 4 WATER, 79 BLOW and 80 WOOD an open monosyllabic form with length in Tungusic corresponds to a disyllabic form with a liquid onset in the second syllable in the other Transeurasian languages. This is indicative of liquid loss in Tungusic.

54. NEW

pJ **ara*- ‘to be new, pure’: J *arai* (A) ‘to be rough, natural, crude’, OJ *ara*- ‘to be rough, fresh, new’, J *ara* ‘new, fresh’, J *arau* (A), OJ *arap*- ‘to wash’, Yamatohama (Amami) *ʔarauri* 'to wash', Asama (Amami) *ʔaroyuɴ* 'to wash', Yoron (Amami) *areɴ* 'to wash', Yonamine (Okinawa) *ʔareɴ* 'to wash', Shuri (Okinawa) *ʔarayuɴ* 'to wash', Irabu (Miyako) *aroo* 'to wash', Hirara (Miyako) *aroo* 'to wash', Ishigaki (Yaeyama) *a:ro: ŋ* [*aarooɴ*] 'to wash', Hatoma (Yaeyama) *arauɴ* 'to wash', Yonaguni *ara*- ‘to be new’, *aruŋ* 'to wash', pR **ara*- 'to be new', **ara-wu*- 'to wash'

pMo **ari*- ‘to be pure: WMo. *ariɣ* ‘pure, clear’, *ariɣun* ‘1 clean, pure, clear; purity’ (WMo -*ɣun* / -*gün* deverbal noun deriving quality words (Poppe 1954: 46)), *arči*- ‘2 to wipe, clean, erase’, MMo. *ariun* ‘1’, *arči*- ‘2’, *aril*- ‘3 to be(come) clear, clear up’, *arilqa*- ‘2’ , Khal. *ariun* ‘1’, *arči*- ‘2’, *aril*- ‘3’, Bur. * aŕūn* ‘1’, *arša*- ‘2’, Kalm. *ärü:n* ‘1’, *arč*- ‘2’, Ordos *aru:n* ‘1’, *arci*- ‘2’, Dong. *arun* ‘1’, *ači*- ‘2’, Bao. *aruŋ* ‘1’, Dag. *aru:n* ‘1’, *arči*- ‘2’, Mgr. *arin* ‘1’, Mgr. *arili*- ‘3’, Mgr. *ariŋge* ‘cleanly’, *arire*- ‘to become pure’, Mogol *oru:n* ‘1’, Eastern Yugur *aru:n* ‘1’

pTk **arï*- ‘to be(come) pure: OTk. *arï*- ‘1 to be(come) clean, pure’, Az. (dial.) *arï* ‘2’, *arït*- ‘3’, Tkm. *arïg* ‘2’, *art*- ‘3’, Tk. *arï* ‘2 clean, pure’, *art*- ‘3 to clean, purify’, Osm. *arït*- ‘to wipe’, Uz. (dial.) *ari*- ‘1’, Uig. *eriq* '2', Tat. *aru* '2', Kirg. *aru:* '2', Kaz. *aru* '2', Bash. *arɨw* '2', Balk. *arɨw* '2', Kpak. *aruw* '2', S-Yug *arɨɣ* '2', Tuva *arïɣ* ‘2’, *arït*- ‘3’, Khak. *arɨɣ* '2', Yakut: *ɨra:s* '2', *ɨrɨt*- 'sort out small fruit', Dolg. *ɨras* '2', Khalaj *arɨɣ* ‘2’, *arut*- ‘3’, Chu. *irӑ* 'good', *irt*- 'sort out small fruit, take away the inner part of a tree'

The intensive-iterative suffix pJ *-*pa*- (Robbeets 2015: 294) is assumed to derive the verb *arau* A ‘to wash’ from a verbal adjective with the meaning ‘to be clean’.

In his review of Robbeets 2005, Kara (2007: 96) suggests that the Mongolic forms in this etymology should be treated as early copies from Turkic. He does not provide a motivation for this copying scenario, but Marcel Erdal and Hans Nugteren (pc.) argue that the suffix -*l*- reconstructed in *aril*- is foreign to Mongolic since the commonly attested suffix WMo. -*l*- derives transitive verbs rather than intransitives as in this case. However, Poppe (1954: 61, 66 vs. 64) distinguishes two different homophonous suffixes WMo. -*l*-: one is a causative or transitive suffix (e.g. WMo. *uɣu*- ‘drink’ → *uɣul*- ‘to give to drink’), while the other is an intensive-iterative suffix (e.g. WMo. *dusu*- ‘fall (of drops)’ → *dusul*- ‘to drip’) that can derive transitive as well as intransitive verbs. The latter suffix also lexicalized in a number of verb pairs granting an inchoative meaning to natural processes, e.g. WMo. *ɣasi-ɣun* ‘bitter’ → *ɣasal*- ‘to lament, mourn’ , *öte-gü* ‘grey’→ *ötel*- ‘to become old’ (Ramstedt 1912: 7-8). It is interesting to observe that, although the transitive suffix practically disappeared in Monguor (De Smedt & Mostaert 1964: 93-94 note), the intensive suffix still surfaces as Mgr. -*li*- (De Smedt & Mostaert 1964: 148) in e.g. Mgr. *ɣasen* ‘bitter’ → *ɣaseli*- ‘to become bitter’, *sači*- ‘to sow’ → *sačili*- ‘to scatter, disperse’ and *arin* ‘clear, pure’ → *arili*- ‘to clear up (intr.)’. The Monguor forms Mgr. *ariŋge* ‘cleanly’ and *arire*- ‘become pure’ are relevant because they can be segmented in a root **ari*- ‘to be clean’ and native suffixes. The form Mgr. *arire*- ‘become pure’ represents the equipollent anticausative counterpart of the causative *arči*- ‘wipe, clean,’ which is well represented elsewhere in Mongolic (Robbeets 2015: 288). Given the presence of the Japanese cognate and the native origin of the suffix -*l*- in MMo. *aril*-, these forms are more likely to have derived from inheritance than from code-copying.

55. BURN

pJ *tak- ‘to burn, boil (tr.)’: J *taku* (A)*,* OJ *tak-* ‘to burn, boil, cook (tr.)’, Yoron (Amami) *takjuN* 'boil (rice/food), make (alcohol/salt)', *yakjuN* 'burn, roast', Shuri (Okinawa) *tak-* ‘to burn’, Hirara (Miyako) *yak*^{s}*ï*, Ishigaki (Yaeyama) *yakuŋ*, Hatoma (Yaeyama) *takuN* 'boil (gruel/glue)', *yakuN* 'fry (including deepfry)', Yonaguni *daguŋ,* pR **tak-* 'to burn, boil'

pK **tʌhʌ-* < pK **takʌ-* ‘to burn (intr.)’: MK *·tho-* ‘to burn, be on fire (intr.)’, MK *ta·hi-*, K *ttay-* ‘to make (fire), heat (with fire) (tr.)’ (MK *-i* causative-passive),

pTk **ya-k-* ‘to ignite, burn (tr.)’: OT (Karakh.) *yak-* ‘1 to ignite, burn (tr.)’, Tk. *yaq-* '1', Tkm. y*aq-* '1', Gag. *yaq-* '1', Az. *yax-* '1', Uz. *yɔq-* '1', Uig. *yaq-* '1', Kirg. *ǯaq-* '1', Kaz. *žaq-* '1', Bash. *yaq-* '1', Nog. *yaq-* '1', Tat. *yaɣ-* '1', Karaim *yaq-* '1', Kum. *yaq-* '1', Kpak *žaq-* '1', Yak. *saq-*, Tofa. *ča'q-* 'to produce fire', Khalaj *ya:q-* '1', Chu. *śot-* '1'

According to Ramsey’s law (cf. 32. BIG, 36. HIT/BEAT), the original root underlying MK *·tho-* ‘to burn, be on fire (intr.)’ can be reconstructed as pK **tʌhʌ-* ‘to burn’. In line with conditioning factor 32b, pK **tʌhʌ-* ‘to burn’ can be assumed to be an assimilation to the second syllable vowel from pK **tahʌ-*. The transitive verb MK *ta·hi-* ‘make (fire)’ can be derived from this root by adding a causative-passive suffix *-i-*. As expected, the addition of a final suffix *-i-* blocks the weakening process of the vowels. Velar lenition supports the reconstruction of pK **takʌ-* ‘to burn’. The correspondence between Chinese donorwords and Korean loanwords (e.g. Ch. *cak* ‘foot (measure)’ is borrowed as MK *· cah*), phonogram readings in the Kyelim Yusa (e.g. *¨hwalq-huy* for MK *holk* ‘earth’), elements in Paekche placenames (e.g. *tin-˙qak* for MK *¨twolh* ‘stone’), dialectal forms (e.g. dial. *tolk* for MK *¨twolh* ‘stone’), and internal doublets (e.g. MK *siphu-* versus MK *sikpu-* ‘want’) all suggest that velar lenition (**k* > **h*) took place at an early stage in Korean (Martin 1996: 36-37).

The correspondence with the Turkic verbs may of course be coincidental. Indeed, the proto-Turkic verb **yak-* ‘to ignite (tr.)’ may represent a complex form, while the inclusion of the Turkic form would lead us to expect register B rather than A in Japanese. The attestation of OTk *yal-* ‘to blaze, burn, shine (intr.)’ and OTk *yan-* ‘to burn, blaze up (intr.)’ suggests that these verbs are morphologically complex. The underlying verb being pTk **ya-* ‘to burn (tr.)’, OTk *yal-* ‘to blaze, burn, shine (intr.)’ would represent a derivation with a passive suffix pTk **-(X)l-* (Erdal 1991: 651-693), OTk *yan-* ‘to burn, blaze up (intr.)’ a derivation with an anticausative suffix pTk *-*(X)n-* (Erdal 1991: 584-638) and, Karakh. *yak-* ‘to ignite, burn (tr.)’ with an inchoative suffix pTk **-(X)k-* (Erdal 1991: 645-650). This inchoative suffix can be traced back to proto-Transeurasian (Robbeets 2015: 255-266) Ultimately, Japanese and Korean may only have inherited the Transeurasian complex inchoative form.

56. NOT

pJ **ana-* negative verb: J *na-*, OJ *na-* ‘not to exist’, OJ *-an-* negative suffix; Hirara *ɲa:ŋ*, Ishigaku *ne:nu*, Yonaguni *minuŋ*, pR **n(i)ano* ‘not to exist’, Yuwan (Amami) -*aN*, Yonamine (Okinawa) *-aɴ,* Shuri (Okinawa) *-aɴ,* Irabu (Miyako) *-(a)n-*, Tarama (Miyako) *-n*, Hateruma (Yaeyama) *-an-*, Ishigaki (Yaeyama) *-anu-*, Hatoma (Yaeyama) *-anu-*, Yonaguni *-anu-*, pR **-an(u)-* negative suffix

pK **an-* negative verb: MK *a·ni* verbal negator (< pK **an-* negative verb + pK **-i* deverbal adverbializer), K *anh-*, MK *anh-* ‘not to be/ do’ (< *ani* + MK *ho-*, K *ha-* ‘to do, be’)

pTg **a:na-* negative auxiliary: Even *a:n ~ a:ŋ* (< pTg **a:na-* + **-xA* resultative nominalizer), *ac* (< pTg **a:na-* + **-c* perfective nominalizer), *acca* (< pTg **a:na-* + **-ca* resultative nominalizer) negative noun, Evk. *a:cin,* Neg *a:cin* (< pTg **a:na-* + *-

cin resultative nominalizer) negative noun, Solon *aš́ĩ* negative noun Ud. *anci* (< pTg **a:na-* + **-cin* resultative nominalizer) negative noun, *ata-* (< pTg **a:na-* + **-ta-* subjunctive) subjunctive negative verb , Na. *ana* ~ *ana:* (< pTg **a:na-* + **-xA* resultative nominalizer) negative noun, Oroch *ana*, Olcha *ana*, Orok *ana* ~ *anaɣa* negative noun, Ma. *aku:*, Sibe *aku* negative noun (< pTg **a:na-* + **-xU* resultative nominalizer), Jur. *a-ĉwi, o-ha* < **oka* 'no, not'
pTk **an-* ‘not to be(come), be unbecoming’: OTk. *anïg* ~ *ańïg* ~ *ayïg* ‘evil, sin, bad; badly, extremely’ (OTk. -*(X)g* deverbal noun suffix), Yak. *aȷ̃ɨ̄*, Dolg. *ańɨ̄*

pTg **e-* negative verb: Evk. *e-* independent negative verb and auxiliary, Even *e-*, Neg. *e-*, Sol. *e-* Na. *e-*, Olcha *e-*, Orok *e-*, Ud. *e-*, Oroch *e-* negative auxiliary , Jur. *ei-xe*, *esi(n)* negative noun
pMo **e-* negative verb in **e-se* (pMo **-sA* resultative nominalizer): MMo *ese*, WMo *ese* ~ *ise* preposed negative adverb, Khal. *es*, Bur. *ehe*, Kalm. *es*, Ordos *ese*, Dong. *ese*, Bao. *ese*, Dag. *es*, Mgr. *sə*, *si:*, Mog. *sa*, *sɛ, sö*
pTk **e-* negative verb in pTk **e-ŋ negative-imperative*: OT (Kharakh.) *eŋ* ‘no, not’, Chu. *an* prohibitive

See Robbeets (2015: 174-205) for an extensive description of the diachrony of negation across the Transeurasian languages. The negative particles Mgr *bi:* (**bu/*bü*) 'don't', *li:* 'not' (**ülü*), *si:* 'not' (**ese*) all replaced their original vowels by an irregular *i:*, probably due to group analogy.

58. KNOW
pK **alʌ-* 'to know': K *a:l-*, MK *al-/¨a(l)-* 'to know'
pTg **ala-* ‘make known, know': Evk. *alawu:-* '1 to teach, explain' (Evk. -*w*- causative), Neg. *alačị-* ' to offer as sacrifice' (Neg. -*čị*- < pTg **-ti-* causative), Olcha *alaụ-* 1, be responsible', Na. *alo:sị-* '1', Orok *alaụ-* '1', Oroch *alụ-* '2 to tell', *alaw-* '1', Ud. *alau-* '1' (Ud. -*u*- causative), Ma. *ala-* 'to tell', Sibe *alə-* 'to tell, to inform', Jur. *alawa-gi* ' royal decree'

62. HEAR
pJ **uka-* 'to receive, perceive, hear': J *uke-* (B), OJ *uke₂-* 'receive', J *uketamawar-* (B), OJ *uke₂tamapar-* ‘to humbly listen, hear, receive’ (OJ *tamapar* - ‘humbly receive, be given’); Shodon *uk'ïïyum*, Shuri *ukiyuŋ,* Yonaha (Miyako) *ukïï*, Ishigaki (Yaeyama) *uki(ru)ŋ* 'to receive'
pMo **uka-* 'to understand, think': MMo. *uqa-* (HY, SH) '1 to understand, think', *uqa* (SH) ~ *uxa'an* (HY) '2 mind', WMo. *uqa-* '1', *uqaɣa(n)* '2', Dag. *ogo*, *owo* 'brain', *uka:* ~ *uha:n* '2', Khal. *uxa-* '1'; *uxa:* '2', Bur. *uxa-* '1', *uxā(n)* '2', Kalm. *uxə-* '1', *uxa:n* '2', Ordos *uxa*: ~ *uxa:n* '2', Eastern Yugur *χGua-tu* '2'
pTk **uk-* 'to hear, understand': OT *uq-* '1 understand', Az. (dial.) *uɣuz* 'knowing much', Uz. *uq-* '1', Uig. *uq-* '1', Tat. (dial). *ux-* '2 hear', Kirg. *uq-* '2', Kaz. *uɣɨn-* '1', Kpak. *uq-* '1', Khak. *ux-* '1, 2', Shor *uq-* '1', Tuva *uɣ-* '1'

In spite of the incongruent register, the verb *ukagaw-* (A) ‘peep through, spy, watch, infer, visit, inquire, hear’ is probably a complex verb derived from pJ **uka-* ‘receive, hear, listen’ and pJ **kapa-* ‘cross, exchange, mutually do’. These roots are reflected in J *uke-* (B) ‘receive’ and *kaw-* ‘buy, cross, exchange, mutually do’. In addition to the compound *uketamawaru* ‘humbly receive, listen, hear’, this verb witnesses to the original semantic component 'hear'.

The lexicon of the Transeurasian languages tends to be verb-based: nouns tend to be derived from verbs, rather than the other way around, e.g., pMo **uka*- 'to understand, think' -> *uka-gan* 'mind' or pTg **xökö*- 'to suck' -> **xökö-n* 'breast'. When a deverbal noun is shared but the underlying verb is not, borrowing is the most parsimonious explanation. This is the case for Tungusic forms for 'mind' such as Evk. *uka:n*, that cannot be derived from a Tungusic verb 'to understand'.

63. SOIL

pJ **tuti* < **tutu-i* 'soil, ground': J *tuti* (2.3), OJ *tuti* 'earth, soil'; Kin (Okinawa) *sicii* (B), Hirara (Miyako) ^{d}*zï:* 'ground', *dzï:* (B), Hatoma (Yaeyama) *sici* (B), Ishigaki (Yaeyama) *cïcï* (B), Yonaguni *di:* (B), pR **zi* 'ground'
pK **tutɨ* 'bank, ridge, ground': K *tutek* ~ *tuteng* ~ *tuleng* (*-enh* 'dike) 'embankment, levee, bank around a field', MK *twu·ten* ~ *twutul* , *twu·tulk* 'bank, ridge, levee, mound, low hill'

Vovin (2008: 124) argues that the Japanese form is a loanword from Korean because "there is only one isolated attestation in Ryukyuan: Ishigaki *tsïtsï* 'earth'". However, the forms for 'ground, earth' are well distributed across the Ryukyuan languages and they all have accentuations, which are consistent with the Mainland Japanese accentuation, so we can presume they are not borrowings from the Mainland. Therefore, Vovin's argumentation can be dismissed. According to Frellesvig and Whitman (2008: 16), the Old Japanese vowel i_2 can derive from a contraction of pJ **ii* or pJ **ui*. If Martin is right that *tuti* 'earth, soil, ground' is the first element in *tuti-hanmyoo* 'Cantharis', while the synonym *niwa-tut/zu* 'Cantharis' indicates *tutu* 'ground', the root may be **tutu* 'ground', probably substantivized by way of pJ **-i*. Additional support may come from the verb J *tutum-* (B) 'to pile up dirt, pack up, cover with, enshroud in' if it is derived with a denominal verbalizer pJ **-ma-* 'to reach a spatial or temporal concept', e.g., OJ *api*$_1$*da* 'interval' → OJ *api*$_1$*dam-* 'to take a break, rest', OJ *ki*$_1$*pa* 'limit, brink' → OJ *ki*$_1$*pam-* 'to reach the limit, reach an extreme' (Robbeets 2015: 246-250).

pK **twu·tɨ* seems to be a legitimate reconstruction on the basis of MK *twu·ten* 'bank, ridge, levee, mound, low hill' and MK *twutul* , *twu·tulk* 'id'.

65. RED

pK **pɨl-kɨ-* 'to be red' (pK *-·*kɨ-* ~ -·*kʌ-* inchoative, e.g. MK *nul-* 'to increase, be(come) longer, be better' → MK *nulk-* 'to be old, grow old (intr.)' < pK **nɨl-·kɨ-* (Robbeets 2015: 257-258): K *pwulk-*, MK *pulk-* 'to be red, be crimson (intr.)'
pTg **pula-* 'to be red': Evk. *xularin (Evk. -rin* colour suffix*)*, *xulama* 'red' (Evk. *-ma* ~ *-me* ~ *-mo-* deverbal noun suffix), *hularga-* 'to redden, turn red' (Evk. *-rgA-* deverbal intransitive inchoative suffix), Even *xulal-* 'to become red', *xulańa:* 'red' (Even *-ńa:* ~ *ńe:* deverbal colour suffix), Neg. *xolayin* 'red', Solon *ụlā̃* 'red', Na. *folgā̃(n),* Ud. *xulaligi*, Ma. *fulara-* 'to be red, to blush', *fulġan* 'red', Sibe *fələġan*, *fulġan* 'red', Jur. *fula-gian* 'red'
pMo **pula-γan* 'red' (*-GAn* resultative deverbal noun suffix, e.g. *uda-* 'to tarry' → *udaγan* 'slow'): MMo. *xula'an* (HY, SH) ~ *hola:m* ~ *hula'an* ~ *hula:n* (Muq), *hulan* 'red', WMo. *ulaγan*, Dag. *xula:n* ~ *hula:n*, Khal. *ula:n*, Bur. *ula:n*, Kalm. *ula:n*, Ordos *ula:n*, Dong. *xulan*, *xulaŋ, fulaŋ,* Bao. *felaŋ*, *fulaŋ*, Eastern Yugur *ła:n*, Mgr. *fula:n*, Mog. *ulo:n*

67. HIDE

pK *_swum_- 'to hide (intr.)': K _swu:m_-, MK _·swum_- 'hide (oneself), conceal oneself, take cover, lurk in (intr.)'
pTg *_sume_- 'to hide (intr.)': Evk. _sumet_- '1 to hide, conceal' (Evk. -_t_- ~ -_ci_- causative/ distributive/intensive/progressive), Even _humec_- 'to act in secrecy' (Even -_c_-/ -_t_- ~ -_ci_- causative/ distributive/intensive/progressive), _humke:c_- ~ _humke:n_- '2 to whisper' (Even -_ka:c_- ~ -_ke:c_- intensive), Neg. _sume:t_- '1' (Neg. -_c_-/ -_t_- ~ -_ci_- distributive/intensive/progressive), Olcha _sumeci_- '1', Na. _sumeci_- '1' (Na. -_ci_- ~ -_si_ distributive/intensive/progressive), Oroch _sumeci_- '2', Ud. _sumemesi_- '2'

The causative suffix pTg *-_t_- ~ -_ti_- is reflected in the Tungusic languages as Ma. -_tA_- ~ -_cA_-, Evk. -_t_- ~ -_ci_- , Even -_c_-/ -_t_- ~ -_ci_-, Neg. -_c_-/ -_t_- ~ -_ci_-, Ud. -_si_- and Na. -_ci_- ~ -_si_ (Robbeets 2015: 281-287). However, only Even, Evenki and Manchu preserve the valence-increasing function of the causative proper next to secondary non-valence changing uses; the other Tungusic languages have all lost the primary causative function but maintained non-valence changing meanings relating to the object such as distributive and complete affectedness of the object, or, to the action such as intensive, iterative and progressive. All languages further preserve resultative function and Even displays valence-decreasing function, namely passive.

68. SKIN
pJ *_kapa_ 'skin, bark, shell': J _kawa_ (2.3), OJ _kapa_ 'skin, leather, fur, pelt, bark, shell'; Yamatohama (Amami) _xo(o),_ Asama (Amami) _koo_, Yoron (Amami) _hoo_, Shodon (Amami) _k'oo_ 'skin, bark', Yonamine (Okinawa) _haa_, Shuri (Okinawa) _kaa_, Irabu (Miyako) _kaa_, Oura (Miyako) _kaa_, Ishigaki (Yaeyama) _kaa_, Hatoma (Yaeyama) _kaa_, Yonaguni _kaa_, pR *_kawa_ 'skin, bark'
pK *_kap(ʌ)-k_ 'skin, bark, shell, outer layer' (pK *-_(a/e)k_ diminutive suffix frequent in body parts, e.g. _muluph_ 'knee' > _muluphak_ 'knee', _thel_ 'hair' > _thelek_ 'hair'): K _kkaphwul_ 'skin, outer layer, film', MK _ka·phol_ 'sheath, scabberd, protective case for a sword', MK _kephul_ 'animal hide', (MK -(_u/o)l_ diminutive), K _kkaptayki_ ~ _kkepteyki_ 'skin, shell' (-_tayki_ ~ _teyki_ diminutive 'thingy'), K _kkepcil_, MK _kepcil_ 'skin, bark, husk'
pTk _ka:p-ïk_ 'bark, shell' (pTk *-_ïk_~ -_ik_ diminutive in body parts or parts of plants, e.g. OT _top_ 'round thing' → _topïk_ 'ankle bone, wrist bone, knee cap', _kasïk_ 'bark, peel, snake-skin', _čopik_ 'remainder of fruit discarded after eating'; Erdal 1991: 43): OT (Karakh.) _kabïk_ '1 bark, 2 shell', Tk. _kabuk_ '1, 2', Az. _ɣabɨɣ_ '1, 2', Tkm. _ɣa:bɨɣ_ '1, 2', Gag. _qap_, _qabɨ_ '1, 2, external cover, covering', Uz. _qɔbiq_ '1, 2', Uig. _qobuq_ '1, 2', Tat. _qabɨq_ '1, 2', Kirg. _qabɨq_ '1, 2', Kaz. _qabɨq_ '1, 2', Nog. _qabɨq_ '1, 2', Bash. _qabɨq_ '1, 2', Balk. _qabuq_ '1, 2', Karaim _qabux_ '1, 2', Kpak. _qabɨq_ '1, 2', Kum. _qabuq_ '1, 2', Khak. _xabɨx_ '1, 2', Shor _qabɨq_ '2', Tuva _xavɨq_ '2', Chu. _xobă_ '1, 2'

In Korean we find various derivations of a root *_kap_ or *_kaph_ 'skin, bark', mainly with dimunitive suffixes. The aspiration in the root *_kaph_ is reminiscent of K _to:l_ ~ dial. _tolk_ , MK _¨twolh_ 'stone' in which the final -_h_ is considered to be a diminutive suffix which lenited from the diminutive suffix pK *-_(a/e)k_ (Martin 1996: 36, 90). There is a vowel harmonic alternation between *_kep_ and *_kap_, but the external evidence indicates that the alternant with the retracted vowel is the original root.

69. SUCK
pTg *_xökö_- 'to suck': Evk. _uku_- '1 to suck', Even _ök_- ~ _uk_- '1', Neg. _öxö_- ~ _uku_- '1,

breast-feed', Oroch *owoci-* ~ *o:ci-* ~ *ueci-* '1', Ud. *kos'o* - '1', Olcha *kueci-* ~ *ko:ci-* '1', Orok *ku:tci-* '1', Na. *ku:ci-* ~ *uku-* '1'
pMo **kökö-* 'to suck': MMo. *koko-* (SH) '1 to suck the breast', MMo. *köke-* (Muq) '1', WMo. *kökö-* '1', Khal. *xöxö-* '1', Ordos *gökö-* '1', Bur. *xüxe-* '1', Kalm. *kök-* '1', Dag. *mək-* '1', Eastern Yugur *hkö-* ~ *hgö-* '1', Mgr. *kugo-* '1', Bao. *kugo-ɣo-* (*-ɣo-* causative) '1', Dong. *gogo-* '1', Mog. *kökȧ-* '1'

See 12. BREAST

70. CARRY
pJ **əpə-* ~ *ənpə-* 'to carry on the back (tr.)': J *ow- (*B*),* OJ *op-* 'to bear, carry on the back', EOJ *opuse-*, OJ *opose-*, J *oose-* (B) 'to charge with', J *obuw-* (B*),* OJ *obup-* 'to carry on the back'; Yoron (Amami) *uujuN* 'return a favour', Yamatohama (Amami) *ʔouri* '(cattle/horse) carries on back', Nakijin (Okinawa) *ʔuuruN* 'carry on back', Shuri (Okinawa) *ʔuuyuN* 'to carry a load, carry a responsibility or sin', Hatoma (Yaeyama) *uuN* 'carry on back'
pK **ep-* 'to carry on the back': K *ep-*, MK *ep-* 'to carry on the back (tr.)'
pTg **ebe-* 'to carry': Na. *ïwarï-* 'to tunload', Evk. *ewe-* 'to carry', Even *iw-* 'to bring in, to carry inside, to import', Oroč. *ewu-gi-* 'to bring', *iwa-dala-* 'to put a person on one's shoulder'

The Yaeyama form *offasïN* 'to carry' is considered to be a loan from mainland Japanese because in case of cognacy initial *u-* would be expected. (Vovin 2008: 235). Although Written Mongolian has a verb WMo. *eɣüre-*, *eɣür-* 'to carry or load on one's back; to bear; to take a burden upon oneself (tr.)' that seems to correspond in form and function, it will not be considered here. In view of the poor distribution of this verb in other Mongolic languages, where we find verbs of the shape pMo **ergü-* 'to lift, raise, carry' and pMo **üɣür-* 'to carry on the back' instead, I follow Poppe's (1955: 48) and Nugteren's (2011: 332) explanation of WMo. *eɣüre-* as a contamination of these verbs.

73. TAKE
pJ **tɨra-* 'to take, pick up': J *tor-* 'to take, get' (B), OJ *to*$_2$*r-* 'to take, pick up, capture', J *toras-* 'to let one have, give', OJ *to*$_2$*ras-* 'to deign to take', J *torae-* (B'), OJ *to*$_2$*rape*$_2$*-* 'to capture'; Yamatohama (Amami) *θururi*, Asama (Amami) *tuyuɴ*, Yoron (Amami) *tuyuɴ*, Yonamine *tuɴ,* Shuri (Amami) *tuyuɴ* 'to take', *tur-* (B) 'to take', *turas-* (B) 'to let one have, give', Irabu (Miyako) *tuï* , Hirara (Miyako) *tul* 'to get', Ishigaki (Miyako) *turuŋ* [turuɴ] 'to take, get', Hatoma (Miyako) *turuɴ* 'to take, get', Yonaguni *turuŋ* 'to get', *turan* (B) 'to let one have, give' pR **tor-* 'to take, get'
pK **tɨlɨ-* 'to hold, lift up': K *tul-*, MK *tul-* 'to hold, carry, raise, lift up'

Bentley (1999) distinguished two Old Japanese verbs, notably OJ *to*$_1$*r-* 'to hold, support' and OJ *to*$_2$*r-* 'to take, pick up, capture'. The latter verb is the closest semantic fit to the basic vocabulary item and regularly corresponds to the Korean form.

pTg **al-* 'to take, receive': Even *al-* '1 to take, receive', Evk. *al-* '1', Neg. *al-* '1', Solon *ali-* '1', Orok *aḷi-* '1', Na. *aḷi-* '1', Olcha *alu:-* '2 to give, hand over ', Oroch *alo:-* '2', Ud. *ali-* '1', *alu-* '2', Sibe *iali-* '1', Ma. *ali-* '1', Jur. *ali-* '2'
pTk **al-* 'to take': OT *al-* 'to take', Tk. *al-*, Tkm. *al-*, Az. *al-*, Gag. *al-*, Uz. *ɔl-*, Uig. *al*, Tat. *al-*, S-Yug. *al-*, Kirg. *al-*, Kaz. *al-*, Nog. *al-*, Bash. *al-*, Balk. *al-*, Karaim *al-*,

Kpak. *al-*, Salar *al-*, Kum. *al-*, Khak. *al-*, Shor *al-*, Tuva *al-*, Tofa. *al-*, Yak. *ɨl-*, Dolg. *ɨl-*, Khalaj *al-*, Chu. *il-*

The Mongolic languages reflect an imperative form 'give!': WMo *ali*, Khal. *aĺ*, *aliv*, Bur. *aĺe:*, Kalm. *aĺ*, *aĺə*, Ordos *ali*, Dong. *ali*, Bao. *an*, Mgr. *ali*. This situation represents a typical case of borrowing, whereby a verb form is only borrowed in its imperative form, but does not reflect a complete verbal paradigm (e.g. *allez* 'let's go, come on!' in Flemish from French). Dagur is the only language reflecting a complete verbal paradigm of the verb *ali-* ' to take, receive', but given the extensive contact situation with Tungusic languages, this may be seen as a loanverb.

74. OLD

pJ **muka-* ‘to be long ago, ancient' (pJ **-si* adverbializer for verbal adjectives, e.g. OJ *suko*$_1$*si* 'a little'): J *mukasi* (?3.1.a), OJ *mukasi* ‘long ago, old times’; Yoron (Amami) *mukasi*, Asama (Amami) *muka:si*, Hirara (Miyako) *mkya:ŋ*, Ishigaki (Yaeyama) *mukaṣï*, Hatoma (Yaeyama) *mukasi*, Yonaguni *Nkaci* (A), pR **muka-si* ‘long ago, ancient times'

pK **muk-* ‘be(come) old’: K *mwuk-*, MK *mwuk-* ‘to become old, outdated’

pMo **kari-* 'to weaken': WMo. *qari-* '1 to weaken, to grow less intense', Khal. *xari-* '1', *xarši-* ' 2 to become exhausted, become emaciated, weaken from lack of food or water', Bur. *xarаši-* '2', Kalm. *xäŕ-* '1'

pTk **karï-* 'to be(come) old': OT *karï-* ' 1 to become old', karï 'old', Tk. *karɨ*, *kart* '2', Az. *Garɨ*, *Gart* '2', Tkm. *Garra-* '1', *Garrɨ* '2', Gag. *qarɨ* '2, 3 old woman or man, woman', Tat. *qarɨ*, *qart* '2', Kirg. *qarɨ-* '1', *qarɨ*, *qart* '2', Nog. *qart* '2', Bash. *qarɨ*, *qart* 2, Kpak. *qarɨ*, *ɣarrɨ* '2', Kum. *qart* '2' Uz. *qari-* '1', *qari* '2', Uig. *qeri-* '1' *qeri* '2, 3', Kaz. *qarɨ* ~ *qɛri*, *qart* '2', Khak. *kirə-* '1', *kirə* '2', Shor *qarɨ-* '1' *qarɨ* '2', Tuva *qɨrɨ-* '1', Tofa. *qɨrɨ-* '1', Yak. *kɨrɨj-* '1', Dolg. *kɨrɨj-* '1'

77. THICK

pJ **puta-* ‘to be thick’: J *hutoi* (B) ‘to be thick, burly, fat’, OJ *puto*$_1$*-* ‘to be thick, fat’ (< **puta-wo-ra* (thick-COP-ADN); see Robbeets 2015: 339-340); Shuri (Okinawa) *butasaN*, Yamatohama (Amami) *hwïssa* 'big', Ikema (Miyako) *udakai*, Irabu (Miyako) *vdaham* 'fat, thick', Hatoma (Yaeyama) *buttaaN* 'fat', pR **buta-* ‘stout, thick’

pK **pwutɨ-* ‘to become thick’: K *pu:s-* ‘to swell (intr.)‘, MK *¨pwuT-* ‘to swell, increase’

pMo **büdü-* ‘to be large’: WMo. *büdügün*, *bidügün* ‘large, huge, big’ (WMo *-ɣun* / *-gün* deverbal noun deriving quality words (Poppe 1954: 46)), MMo. *bidun*, Dag. *budun*, *budu:n,* Khal. *büdü:n*, Bur. büdü:n, Kalm. *büdü:n*, *bödü:n*, Ordos *büdü:n*, *bidü:n*, Dong. *biedun*, Bao. *beidoŋ*, Eastern Yugur *budü:n*, Mgr. *budin*, *bidun*, Mog. *beidü:n*, *beidun*

The Old Japanese initial *p-* may require special notice because it has been suggested that its articulatory definition had already become a bilabial fricative *F* by the time of Old Japanese. Miyake (1999: 396-400) has argued against the spirantization of OJ *p*, demonstrating that *p* remained unchanged until Middle Japanese when it became a fricative *f*.

In Mongolic, two verbally encoded adjective stems alternate: pMo **büdü-* ‘to be large’ and pMo **bedü-* ‘to be large’. The latter form may have arisen through

convergence with a form ancestral to OTk. *bädü-* ‘to be(come) big, great’ (Doerfer 1963: 235; 1975: 275.). If pMo **büdü-* is not to be considered as a mere vocalic alternant of **bedü-*, perhaps for iconic reasons, it may belong here as a cognate to the Japanese and Korean forms.

78. LONG

pJ **nanka-* 'to be long': J *naga-* (B), OJ *naga-* 'to be long'; Yamatohama (Amami) *nagasari*, Asama (Amami) *nagaahaɴ,* Yoron (Amami) *nagasaɴ,* Shodon (Amami) *nagasam*, Yonamine (Okinawa) *nagaaseɴ*, Shuri (Okinawa) *nagasaɴ,* Irabu (Miyako) *na'aham*, Hirara (Miyako) *nagakaɿï*, Ishigaki (Yaeyama) *na:sa:ŋ* [*naasaaɴ*], Hatoma (Miyako) *naaɴ* ~ *nagaaɴ,* Yonaguni *na:ŋ*, pR **naga-* 'to be long', OKog **na(-)mey* 'long (water)'

pK **nʌlkʌ-* ~ **nɨlkɨ-* ‘to be(come) old, long (in time) : K *nalk-*, MK *nolk-* 'to be(come) old, be outdated (of objects)', K *nulk-*, MK *nulk-* 'to become old, age (of people)', K *nul,* MK *nul-* ‘to increase, be(come) longer, be better’

If the Korean form is indeed morphologically complex (pK **-kɨ-* ~ *kʌ* deverbal inchoative in MK *nul-* ‘to increase, be(come) longer, be better’ → MK *nulk-* ‘to be old, grow old (intr.)’ < pK **nɨl-kɨ-*; see Robbeets 2015: 256-258), the Japanese form can only be explained as a borrowing.

pMo **uri* 'long past, former' in **uri-da* 'before' (**-da* ~ *-de* local and temporal adverb, e.g. WMo. *nasun* 'lifetime' → *nasuda* 'always'; Poppe 1954: 57): MMo. *urida* (SH, HY, Muq), WMo. *urida*, Dag. *ward*, Khal. *urd*, Bur. *urda*, Kalm. *urd*, Eastern Yugur *urda*, Mog. *urdɔ* 'vicinity, near' and in **uri-ji* 'before last': MMo. *uriju ödür* ~ *urji ödür* 'day before yesterday', Kal. *urdjar*, Ordos *urjidur*, Bur. *uržader*, Kalm. *urj ödr*, Eastern Yugur *urjüdur*, Mgr. *uʒüdur*, Bao. *nʒiudər*, Dong. *uruẓu du*, Mog. *ujaʊdur*

pTk **ur₂a-* 'to be(come) long (in time/ space)' (pTk **-(X)n* deverbal noun/adjective suffix, e.g OTk. *yak-* 'to be near, approach' → *yakïn* 'near'; pTk **-(O)k* deverbal noun/adjective suffix, e.g. OT *yagu-* 'to come near' → *yaguk* 'near'): OT *uza-* 'be(come) long and drawn out', *uzun* '1 long', *uzak* '2 long-lasting', Tk. *uzun* '1', *uzak* '3 far', Az. *uzun* '1', *uzaɣ* '3', *uzan-* 'to grow', Tkm. *uzɨ:n* '1', *uzaq* '3', Gag. *uzun* '1', Uz. *uzun* '1', *uzɔq* '3', Uig. *uzun* '1', *uzaq* '3', Khak. *uzun* '1', Shor *uzun* '1', *uzaq* '2', Tuva *uzun* '1', *uzaq* '3', Tofa. *uzun* '1', *uza-* 'to lengthen, elongate (intr.)', Yak. *uhun* '1', Dolg. *uhun* '1', Tat. *ozɨn* '1', *ozaq* '3', S-Yug. *uzun* '1', *ozaq* '3', Kirg. *uzun* '1', *uzaq* '2,3', Kaz. *uzun* '1', Nog. *uzɨn* '1', Bash. *oδon* '1', Balk. *uzun* '1', *uzaq* '3', Karaim *uzun* '1', Kpak. *uzɨn* '1', *uzaq* '3', Salar *uzɨn* '1', *uzɨχ* '3', Khalaj *uza:n-* 'to grow', *uza:q* '3', *uzu:n* '1', Chu. *vărăm* '1', *vărax* '3'

pK **ola-* ‘to last long’: K *olay-*, MK *wo·la-* ‘to be long(lasting), be a long time since’

pMo **ora-* 'to be late' in **orai* 'late' (pMo **-i* adverbializer, e.g. WMo *daru-* 'to follow' → *darui* 'thereafter'; Robbeets 2015: 463): MMo. *orai* (SH) '1 late, in the evening, at night', WMo. *oroi*, *orai*, *orui* '1', Dag. *ore:* '1', Khal. *oroy* '1', Bur. *oroy* '1', Kalm. *ora:* '1', Ordos *orö*: '1', Eastern Yugur *öröi* '1', Mgr. *urui* '1', *ure:* '1', *uri:* '1'

I do not exclude that the above two etymologies ultimately go back to a single root with different vocalization.

79. BLOW

pK **puli*- 'to blow': K *pu:l*-, MK *pwul*- ~*¨pwu(l)*- 'to blow (an instrument/ of wind) (tr. / intr.)'
pTg **pu:*- 'to blow': Evk. *huw*-, Even *hu:*-, Neg. *xu:w*-, Solon *u:gu:*-, Olcha *pu:*-, Orok *pu:*-, Na. *pu:*-, Oroch *xu:*-

If the alternant with velar fricative initial in Middle Turkic *ür*- ~ *hür*- 'to blow' indicates an original labial stop initial in pTk **(p)ür*- 'to blow', the following Turkic verbs belong here as well: OT *ür*-, Turkish *ür*- (dial.),Tatar *ör*-, Khakassian *ür*-, Chuvash *vĕr*-, Yakut *ür*-, Dolgan *ür*-, Tuva *ür*-, Kazakh *ür*-, Noghai *ür*-, Bashkir *ör*-, Karaim *ür*-, Karakalpak *ür*-

80. WOOD

pJ **kɨ(r)i* 'tree, wood': J *ki* (1.3a) 'tree, wood', OJ *ki*$_2$ ~ OJ *ko*$_2$- 'tree, wood, low woody plant, plant' in OJ *ko*$_2$*dati* 'grove, stand of trees', *kopada* 'tree bark', *ko no pa* ‘leaves’, etc.; Yamatohama (Amami) *xï(i),* Asama (Amami) *kɨɨ*, Yoron (Amami) *sii*, Shodon (Amami) *kïï*, Yonamine (Okinawa) *kii*, Shuri (Okinawa) *kii*, Irabu (Miyako) *kii*, Hirara *kii,* Ishigaki (Yaeyama) *kii* (B), Hatoma (Yaeyama) *kii*, Yonaguni *kii* (B), pR **ke;* OKog **kɨr* 'tree, wood' (Beckwith 2007: 111, 115)
pK **kɨlɨ-k* > **kɨlɨh* 'tree, wood' (pK *-*(a/e)k* diminutive suffix, also in *namu* ~ *namk* 'tree'): K *kulwu,* MK *kuluh* 'a stump (e.g. of a tree), stubble, part of a tree or crop left in the ground after the it has been cut down; counter for trees; crop, sowing'

The Tungusic languages share a suffix denoting trees and bushes, suggesting an original shape *-*kire:* in addition to *-*kurA:* (Benzing 1955a: 1015). Reflexes of *-*kire* appear in Evk. *seŋkire*:, Even *heŋkeye*, Neg. *seŋkiye*:, Ma. *seŋkiri*, Oroch *seŋki*, Ud. *seŋkie* 'wild rosemary' and Oroch *oloŋki*: 'oak'. However, given the *u* reflex in Evenki and a faint trace of *o*~ *u* alternation in the Southern Tungusic languages, it remains unclear whether we should reconstruct *-*kurA:* or *-*körA:* on the basis of Olcha *seŋkure*, Orok *sekkure*, Nanai *se:ŋkure* 'wild rosemary', Olcha *χaŋgaqu̦ra*, Orok *χaŋgattu̦ra* 'elder', Evk. *dikte-mkure* 'blueberry bush', Nanai *xoroŋkola*, Ud. *oloŋkö* 'oak', Na. *χāŋGoã* 'elder'. Although pTg *-*körA* 'tree, bush' would make a good phonological match to the Japonic and Koreanic forms, I have not included it in the etymology because the reconstruction of the vowel remains uncertain.

pJ **morɨ-(C)i* 'woods, wooded hill': J *mori* (2.1) 'woods', OJ *mi*$_1$*-moro* 'place/grove where a god descends'; Yoron (Amami) *mui* 'hill', Asama (Amami) *mui* 'hill', Shuri (Okinawa) *mui* '(wooded) hill', Hirara (Miyako) *muï* 'woods, forest', Irabu (Miyako) *muï* 'forest, mountain', Ishigaki (Yaeyama) -*murï* 'hill' (bound form found only in place names), *yamamurasï* 'grove', Hatoma (Yaeyama) *muru* 'hill', pR **morï* 'woods, wooded hill'
pK **molo* 'hill, mountain': K *mey*, MK *¨mwoyh* ~ *¨mwoy* ‘hill, mountain’ (< **mwolih* < ? **mwolwo-hi*), *·phi mwo·lwo* (1445 Yong 4, 21b) ‘Torreya-nut Mountain (placename)’
pTg **mo:* 'tree, wood': Evk. *mo:* '1 tree; 2 stick, log, timber; 3 forest, wood', Even *mo:* '1, 2, 3', Solon *mo:* '1, 2' , Neg. *mo:* '1, 2, 3', Olcha *mo:* '1, 2', Orok *mo:* '1, 2, stem of a tree', Na. *mo:* '1, 2', Oroch *mo:* '1, 2, 3', Ud. *mo:* '1, 2', Ma. *mo:* '1, 2, 3', Sibe *mo:* '1, 2, 3', Jur. *mo* '1'
pMo **mo* 'wood, tree' in pMo **mo-dun* 'wood, tree' (*-*dun* ~ -*dün* collective suffix, e.g. in WMo. *sodun* 'pinion feathers of a bird', *sidün* 'tooth', etc.; Poppe 1973: 233): MMo. *modun* ~ *mudun* (SH, HY, Muq) '1 wood, tree', WMo. *modu(n)* '1', Dag. *mo:d*

'1', Khal. *mod(on)* '1', Bur. *modo(n)* '1', Kalm. *modn* '1', Ordos *mudu(n)* '1', Eastern Yugur *mu:dən* '1', Dong. *mutun* ~ *mutuŋ* '1', Mgr. *mo:du* '1', Bao. *mutoŋ*, *mortoŋ*, *modoŋ*, Mog. *modun* 'wood', *mudun* 'wooden stick' and in pMo **mo-ci* (*-*ci* agentive suffix, e.g. in WMo. *mal* 'cattle' → *malci* 'herdsman'; Poppe 1954: 41): MMo *moci* (SH) 'carpenter', Mgr. *moči* 'carpenter'

81. RUN

pJ **pasa*- 'to run': J *hase*- (?B), OJ *pase*- 'to run, gallop', J *hasir*- (B), OJ *pasir*- 'run'; Yamatohama (Amami) *hasiruri*, Asama (Amami) *hasɨyuɴ,* Nishikomi (Amami) *haryur* 'to run, flow', Yonamine (Okinawa) *paɴ,* Shuri (Okinawa) *hayuɴ* 'to run', Shuri *har*- (B) 'to run, flow', Irabu (Miyako) *piï*, Hatoma (Yaeyama) *paccaasuɴ, pariparuɴ,* pR **pa(si)r*- 'to run'.

pK **pas*- 'to hurry': MK *pach*- 'to rush (intr.)' (< **pas-kʌ*- with pK *-*kɨ*- ~ *kʌ* deverbal inchoative; Robbeets 2015: 256-258), K *pappu*-, MK *pachpo*- ~ *paspo*- 'to be busy' (< **pas-kʌ*- - with pK *-*pɨ*- ~ *pʌ* resultative deverbal adjective suffix, e.g. MK *sulh*- 'to grieve (intr.)' → *sulphu*- 'to be sad'; Robbeets 2015: 296).

The Amami examples reflect pR **pasir*-, while the others all point to pR **par*-. However, these forms are probably related over the loss of *-*s*- before a high front vowel. In Ryukyuan, pJ **pasira* 'pillar' becomes *paya* or *paiya*. Similarly, the Ryukyuan counter for animals is -*kara*, thought to be from pJ **kasira* 'head'. We would then not expect the -*s*- in the Yamatohama and Asama dialects, but it is quite possible that these forms reflect influence from Kagoshima dialect. The Nishikomi dialect of Amami Oshima has *haryur* 'run, flow', without the -*s*-.

82. FALL

pJ **tira*- 'fall, scatter': J *tir*- (A), OJ *tir*- 'fall, scatter (intr.)', OJ *tiras*- (A) 'scatter, strew (tr.)'; Shuri *cirir*- (A) 'scatter (intr.)', *ciras*- (A) 'dry up a bowl (< disperse the liquid)'

pK **ti*- fall, scatter': K *ci*- 'fall, scatter (of flowers/leaves), go down (of sun/moon), die, die out (of fire), fade away', MK *ti*- 'fall, go down, turn upside down, lose, die'

84. ASH

pJ **papi* 'ash': J *hai* (2.1), OJ *papi* 'ash'; Yamatohama (Amami) *hwe*, Yoron (Amami) *pai*, Shodon (Amami) *fə,* Yonamine (Okinawa) *pee*, Shuri (Okinawa) *fee* [hwee], Irabu (Miyako) *(kara)paï* , Hirara (Miyako) *pa^z^ï*, Ishigaki (Yaeyama) *pai* (A), Hatoma (Yaeyama) *pai*, Yonaguni *higun* (B) (<? **pi-nkomi* 'fire trash'), pR **paWe* 'ash'

pK **pap* 'waste in a production process': K *pap* 'waste material generated in a production process (e.g. sawdust, scraps of cloth/paper left after cutting, earth turned over by a plow, bits of thread)'

86. DOG

pJ **inu* 'dog': J *inu* (2.3), OJ *inu* 'dog'; Yamatohama (Amami) *ʔiɴ,* Asama (Amami) *ʔiɴ,* Yoron (Amami) *inu*, Shodon (Amami) *iɴ,* Yonamine (Okinawa) *ʔiɴnuᴋwaa*, Shuri (Okinawa) *ʔiɴ,* Irabu (Miyako) *in*, Hirara (Miyako) *iŋ*, Ishigaki (Yaeyama) *iŋ* [*iɴ*] (B), Hatoma (Yaeyama) *iɴ,* Yonaguni *inu* (loan?), pR **inu* 'dog'

pTg **ina-kun* ~ **ina-ki:* 'dog', **in(a)-da* '(going) with a dog' (pTg *-*kun* suffix common in animal names, e.g. pTg **ju-kun* 'otter', pTg **gia:-kun* 'sparrow hawk'; Benzing 1955: 1015; pTg *-*ki:* animal suffix, e.g. pTg *sula-ki:* 'fox', **xölü-ki:*

'squirrel', etc., pTg *-*na*:- (~ -*da:*- after *n*) see 3. GO OUT): Even. *ina* ~ *nina* ~ *ŋina, inakin* ~ *ŋinakin* '1 dog', Evk. *ŋin* '1' (*ŋinal* PL), *ŋinakin* ~ *ginakin, ŋinda*- 'to go out with the dogs, hunt with the help of dogs', Solon *inaxĩ* ~ *ninaxĩ* ~ *ninakin* '1', Neg. *ina* ~ *nina*, *ninakin*, *enakin* ~ *enaxĩ* '1', Oroch *inaki* ~ *inaxki* '1', Ud. *in'an* '1', Olcha *iŋda* '1', Orok *nina* ~ *ŋina* '1', Olcha *inda* '1', Na. *inda*, *inakĩ* '1', Ma. *indaxun* ~ *indaju* '1', Sibe *jonəhuŋ*, *inəhuŋ* '1'

The phonological development assumed to have taken place in Tunguisic is **ina-kun* ~ **ina-ki:* (contamination*)* > **ina-kin* > **ginakin* (assimilation) > **ŋinakin* > **ninakin.* I assume that the Evenki denominal verb *ŋinda*- 'to go out with the dogs, hunt with the help of dogs' contains a dissimilated form of the suffix pTg *-*na*:- 'to go out (with)'. Since this suffix is also reflected in the collective suffix pTg *-*nan* 'together with' (Benzing 1955: 1021, e.g. *aki:-nan* 'together with the older brother'), I assume that the southern Tungusic forms *inda* reflect such a collective derivation. Note also that the root pTg **inu:-ke* 'dog, wolf' (e.g. Evk. *ńēkē* 'sable', Even *ŋȫke* 'male (of dog, wolf, fox)', Sibe *juxə* 'wolf', Ma. *ńoxe* 'wolf', *nuxere* 'puppy', Oroch *ŋöksjö* 'wolf' (Cincius 1975: 587, 651, 665, 606) may be related.

87. CRY / WEEP

pK **ulɨ*-'to cry, howl': K *wu:l*- 'to cry, weep, shed tears (of humans); howl, sing (of animals); sound, ring (of things) (intr.)', MK *wul*- ~ *¨wu(l)*- 'to cry, howl, sound (intr.)'

pMo **uli* 'to howl': MMo. *uli*- '1 to howl (of dogs, wolves, etc.)', WMo. *uli*- '1', Khal. *uli*- '1', Bur. *uli*- '1', Kalm. *ul*́-, *ulə*- '1', Ordos *uli*- '1', Eastern Yugur. *olo*- '1'

pTk **u:lï*- 'to cry, howl': OT *ulï*- 'to cry (of humans), to howl (of wolves and other animals)', Tk. *ulu*-, Gag. *ulu*-, Az. *ula*-, Tkm. *u:lɨ*-, Uig. *ulu*-, Kaz. *ŭlɨ*-, Nog. *ulɨ*-, Bash. *ŭlŭ*-, Kpak. *ulɨ*-, Karaim *ulu*-, Tat. *ula*-, Kirg. *ulu*-, KBalk. *ulu*-, Kum. *ulu*-, Khak. *ulu*-, Tuva *ulu*-, Yak. *uluy*-, Dolg. *uluy*, Chu. *ălax*- 'to neigh'

This etymology was eliminated from the core evidence advanced in Robbeets (2005) because it may be a sound symbolic formation.

88. TIE

pJ * *kuku*- 'to tie, wrap': J *kukur*- (A), OJ *kukur*- 'to tie up, bundle, fasten', OJ *kukum*- (B) 'to tie up, wrap up', J *hagukumu* (A), OJ *pagukumu* 'brood over, foster' (< **pa* 'feather' + *-*n*- + **kuku-ma*- 'wrap'), Shuri (Okinawa) *kukur*- (A) 'to tie up, blindstitch'

pTg * *xuku*- 'to wrap': Evk. *ukuli:*- ~ *hukuli*- 'to wind round', Even *uk*- 'to roll up, put a strap together', Neg. *uxil*- 'to wrap up', Solon *uxuli:*- 'to enfold', Olcha *xuku*- 'to wind round, entrap', Orok *xukulitči*- 'to wind round, entrap', Na. *xuku*- 'to wind round, wrap up', Ma. *uxu*- 'to fold up', Jur. *hu-xun-mij am-si-da-lar* 'to contain'

In spite of the incongruent register in Japanese, it seems that J *kukur*- (A) 'to tie up, bundle, fasten' and J *kukum*- (B) 'to tie up, wrap up' can be derived from the same root pJ **kuku*- 'to tie, wrap'. See 32. BIG for the observation that the assignment of a verb register not always straightforward. The confusion may have arisen through contamination with J *kukum*- (B) 'to hold in the mouth'.

pMo **boɣo*- ~ *boɣa*- 'to tie up, wrap': MMo. *bo'o*- (SH) 'to block, obstruct', *bo:*- ~ *bo'o*- (Muq) '1 to tie up, bundle, wrap', *bo'am* (HY) 'dam, barrage', WMo. *boɣu*- '1', *boɣuca* 'bundle, string for tying a bag', Khal. *bo:*-, Bur. *bo:*-, Kalm. *bo:*-, Ordos *bo:*-,

Eastern Yugur *pu:-*, Huzhu Mgr. *bo:-*, Bao. *boɣəldə-*
pTk **bog-* 'to tie up': OT *boɣ-* '2 strangle, choke', *boɣ* 'bundle', Tk. *boɣ-* '1 to tie up, constrict by binding', *bo:-* '2', *boɣ* 'bundle', Az. *boɣ-* '1, 2', Tkm. *boɣ-* '1, 2', Gag. *bu:-* '2', Uz. *bọɣ-* '1, 2', Uig. *boɣ-* '2', Tuva *boɣ-* '1, 2', Yak. *buoy-* 'to hinder', Dolg. *buoj-* 'to hinder; to pacify, appease', Tat. *bu-* '2', Khak. *poɣ-* '1, 2', Kirg. *bu:-* '1, 2', Kaz. *bu-* '2', Nog. *buw-* '2', Bash. *bɨw-* '2', Balk. *buw-* '1, 2', Karaim *boɣ-* '2', Kpak. *buw-* '1, 2', Kum. *buw-* '2', Chu. *păv-* '2'

90. SWEET
pJ **ma-* 'to be sweet, tasty': J *ama-* (A), OJ *ama-* 'to be sweet', J *uma-* (B), MJ *muma-*, OJ *uma-* 'to be delicious, appetizing, sweet'; Yamatohama (Amami) *ʔamasari*, Yoron (Amami) *amasaɴ* 'to be sweet', Shodon (Amami) *maa-* 'to be tasty', Yonamine (Okinawa) *ʔamaaseɴ* 'to be sweet', Shuri *maa-san* 'to be tasty', *ʔamasaɴ* 'to be sweet', Irabu (Miyako) *adzïmaxam* 'to be sweet', Ishigaki (Yaeyama) *azïmasaaɴ* 'to be sweet', Hatoma (Yaeyama) *azimaaɴ* 'to be sweet' (< pR **adi-maa-* [taste-be.sweet/tasty] 'be sweet'), Yonaguni *maan*, pR **ma:-* 'to be tasty, sweet'
pK **ma-* 'to be tasty' in **mas* 'flavor' (pK **-s* deverbal suffix deriving nominal adjectives and adverbs, e.g. K *cilki-* 'to be tough'→ *cilkis* 'firm'; Robbeets 2015: 422-423): K *mas*, MK *·mas* 'flavor, taste'

The initial *(m)u-* may have been protetic in Japanese. The Middle Japanese variant *muma-* 'to be delicious, appetizing, sweet' as well as the Ryukyuan cognates support this idea. A parallel development has probably taken place in the word for 'horse', which is reflected in J *uma* and MJ *(m)uma* 'horse'. The Ryukyuan cognates of this word, e.g. Miyako *nuuma*, Yaeyama *nnma*, Hateruma *qman*/*nman* 'horse' lack an initial vowel and this is also true for its probable Old Chinese model 馬 **mˤraʔ* 'horse' (Robbeets 2017).

pK **tʌlʌ-* 'to be sweet': K *tal-* 'to be sweet, tasty, pleasant', MK *tol-* 'to be sweet'
pTg **da:l-* 'to be sweet': Even *da:l-* 'to be sweet, pleasant; to lick off', *dalra* 'tasty, sweet' (Even *-ra* ~ *-re* deverbal adjectival noun suffix, Robbeets 2015: 351), *dalsi:* 'tasty, sweet' (Even *-si*: deverbal adjectival noun suffix, Robbeets 2015: 351), Evk. *dalli*, *daldi* 'tasty, sweet' (Evk. *-li*, *-di* deadjectival adverbializer; Nedjalkov 1997: 306-307), Neg. *daḷịgdị:* 'tasty, sweet', Ma. *dalxi* 'relish, predilection'

Note that J *tara* (2.5), *tara-no-ki* 'Japanese angelica tree, fatsia, Aralia elata' (Shuri *taarasi* B 'Magnolia obovata' = J *hoonoki* in which hoo may derive from big-leafed) is a plant with candied stems which can be used in making cakes or sweets. If not related with the Korean word for 'rush, reed' and with the Tungusic words for 'quitch; reed, cane' (Robbeets 2005), the word may be a compound preserving **tara* 'sweet, sweetness'.

91. ROPE
pJ **turu* 'string, line': J *tura* (2.2.a), OJ *tura* 'row, line', J *turu* (2.2.a) 'string, bowstring, chord', OJ *turu* 'bowstring, string of a musical instrument', EOJ *tura* 'bowstring'; Asama (Amami) *cïruu*, Yoron (Amami) *ciru*, Nakijin (Okinawa) *ciruu*, Shuri *çiru*, Hatoma (Yaeyama) *siru*, Ishigaki (Yaeyama) *curu*, Irabu (Miyako) *ciru*, pR **turu* 'string of a musical instrument, bowstring'.
pK **cul* 'rope, string, line': K *cwul*, MK *·cwul* 'rope, cord, string, line'

Since OJ *tura* 'row, line' and OJ *tuna* 'rope, cord, string, line’ overlap in form and meaning, they could be regarded as a case of *-r-~-n-* assimilation. However, the accentuation of the forms is different: J *tura* (2.2.a) has the corresponding accent class A in Ryukyuan, while J *tuna* (2.3) has class B in Ryukyuan. The latter form is reflected in the Ryukyuan words for 'rope', notably Yamatohama (Amami) *cïna*, Asama (Amami) *Cɨnaa*, Yoron (Amami) *cina,* Yonamine (Okinawa) *cinaa*, Shuri (Okinawa) *cina*, Irabu (Miyako) *cïna*, Hirara (Miyako) *tsïna* 'rope', Hatoma (Yaeyama) *sina* 'rope' and Yonaguni *nna* 'rope'.

92. SHADE

pJ **kanka* 'shade, reflection ': J *kage* (2.5) 'shadow', OJ *kage*$_2$ ‘shadow, reflection, radiance, light’ ~ OJ *kaga-*, e.g. in J *kagami*, OJ *kagami*$_2$ ‘mirror’ (< ‘reflection’ + ‘look’); Yamatohama (Amami) *xage*, Asama (Amami) *kaagɨ*, Yoron (Amami) *hagi*, Yonamine (Okinawa) *hagi* 'shade', Shuri (Okinawa) *kaagaa* 'shade, reflection', Irabu (Miyako) *kaagi*, Hirara (Miyako) *kagi*, Ishigaki (Yaeyama) *kai* (B) ~ *kaagi*, Hatoma (Yaeyama) *kagi*, Yonaguni *kaNgi*, pR **kage* ~ *kaga-* 'shade, reflection'
pK **kʌnʌlh* < ? **kʌnhʌl* ‘shade, shadow’: K *kunul*, MK *·ko·nolh* ~ *·ku·nul* 'shade, shadow'

94. SALT

pJ **tura-* 'unbearable, bitter': J *tura-* (B), OJ *tura-* 'unbearable, bitter, hard'
pTg **turu-ke* 'salt' (pTg *-*xA*: ~ *-*kA*: resultative deverbal noun/adjective, e.g. Evk. *culbin-* → *culbika-* ‘thin, meager’, Evk. *upcu-* ‘to dispute’ → *upcuka* ‘disputable’) Southern Evenki (Chiringda) *turukə* ‘salt’, Northern Evenki (Tutonchany) *turukə* ‘salt’, Northern Evenki (Tura) *turukè* ‘salt’, Eastern Evenki *turukə* ‘salt’. Negidal *tos* ‘salt’ is likely to be a borrowing from Turkic.
pTk **tu:r*$_2$ '1 salt': OT *tuz* '1', Tk *tuz* '1', Az. *duz* '1', Tkm. *du:z* '1', Gag. *tuz* '1', Kirg. *tuz* '1', Kaz. *tuz* '1', Nog. *tuz* '1', Bash. *toδ* '1', Balk. *toδ* '1', Tat. *toz* '1', Karaim *tuz*'1', Kpak *duz* '1', Sal. *duz*, *düź*, *tuz* '1', Kum. *duz* '1', Uz. *tuz* '1', Uig. *tuz* '1', SYug. *duz* '1', Khak. *tus* '1', Shor *tus* '1', Tuva *dus*, Tofa. *tus*, Yak. *tu:s*, Dolg. *tu:s*, Khalaj *tu:z* '1', Chu. *tăvar* '1'

95. SMALL

pJ **tipi-* 'to be small' (pJ *-*sa* deverbal noun/adjective suffix): J *tiisa-* (B), OJ *tipi*$_1$*sa-* 'small, little'
pTg **čipi-* ‘to be small, narrow’ ~ pTg **čip-ti-* 'to make small, narrow' (pTg *-*t-* ~ -*ti-* causative suffix; see 67. TO HIDE): Evk. *čipikte* '1 narrow' (Evk. *-kta* ~ *-kte* deverbal resultative noun suffix; Nedjalkov 1997: 299), *čipileme* '1 narrow', *čipka-* '2 to squeeze, press', *či:pči:-* '3 to fill, stuff, push in' (Evk. *-t-* ~ *-či-* causative), Even *ču̩pu̩ti̩* '1', *či̩pču̩-* '3', Neg. *čipixet-* '1', *či̩pi̩xi̩la-* '2', *či̩pči̩-* '3' (Neg. *-č-*/ *-t-* ~ *-či-* causative), Na. *či̩p* 'tightly', Oroch *čipči-* '3', Ud. *čipči-*, Olcha *čipeči-* '4 to pick up with the fingers (of food remnants)', Orok *či̩po-* '4', Ma. *čibu-* '3'

96. WIDE

pJ **nənpa-* > **nənpɨ-* ‘to become long and wide’: J *nobe-* (B)*,* OJ *nobe*$_2$*-* ‘to stretch, spread, lengthen (tr.)’, J *nobi-* (B)*,* OJ *nobi*$_2$*-* ‘to extend, lengthen, stretch, spread, grow; be postponed (intr.)’, J *nobas-* (B)*,* OJ *nobas-* ‘to extend, lengthen, stretch, spread (tr.)’; Yoron (Amami) *nubasjuN* ~ *nubjuN* (tr.), *nubajuN* (intr.), Shuri (Okinawa) *nubir-* 'to spread, extend (tr.) (B) *nubas-* (B) 'to extend, lengthen (tr.)', Hatoma (Yaeyama) *nubasuN* (tr.), *nubiruN* (intr.)

pK **nelp(ɨ)-* ‘to be wide’: K *nelp-* ‘to be wide’, MK *nep-* ‘to be wide’, MK *nelu-* ‘to be wide’

pTg **nepte-* ‘to become flat and wide’: Even *nebde-* ‘to pull off the skin in one piece’, *nebde* ‘open(ness); wide(ness)’, *nebden-* ‘to unfold widely; open up (of cloth, wings); straighten out; open up (of leaves) (intr.)’ (Even *-(A)*$n_{(2)}$*-* processive**)**, *nebdeńe:* ‘flat, wide’ (Even *-ńA* deverbal adjectivizer), *nebder-* ‘to open, come out (of flowers) (intr.)’, *nebdeku* ‘opened up; wide’, Evk. *nepte-* ‘to unfold, smooth out, spread out’, *nepteme* 'even, flat', Neg. *nepte-nepte* ‘even’, Na. *nepte-nepte* ‘even’, Olch. *nepte-nepte* ‘even’, Orok *nette-* ‘spread out’, Oroch *nepteŋge* ‘even, flat’, Ud. *neptele* ‘even, flat’

pMo **nebse-* ‘to be(come) wide and long’: WMo. *nebseger* ‘wide and long’ (WMo. -*GAr* deverbal quality noun (Poppe 1954: 46)), WMo. *nebseyi-* ‘to be wide and long (of clothes), to be tattered, in rags (intr.)’ (pMo **-yi-* anticausative), WMo. *nebsegene-* ‘to move (of something wide and long)’ (WMo. *-GA-* factitive; Poppe 1954: 61, pMo **-nA-* processive; Robbeets 2015: 235-237), Khal. *nevsiy-*, Bur. *nebšɨ-*

Robbeets (2005: 375; 2008, 2015) argues that the voiced series in Japanese, which are internally derived from original nasal clusters, can be traced back to clusters in the Transeurasian languages. The original clusters can be divided into homoganic and heteroganic clusters. Homoganic clusters are composed of a sonorant and a stop (pTEA **-Rp-*, **-Rt-*, **-Rk-*) and merge in a nasal cluster (pJ **-np-* > OJ *-b-*, pJ **-nt-* > OJ *-d-*, pJ **-nk-* > OJ **-g-*) in Japanese. In heteroganic clusters, as illustrated in this etymology, on the other hand, the nasal and the stop have a different place of articulation, which results in the insertion of a parasitic stop (pTEA *-$m^{(P)}T$-, *-$n^{(T)}K$-, *-$ŋ^{(K)}T$-). The nasal is lost in the continental Transeurasian languages (**-PT-*, **-TK-*, **-KT-*), whereas Korean and Japanese lose the final stop (pJ **-mp-* > OJ *-b-*, pJ **-nt-* > OJ *-d-*, pJ **-ŋk-* > OJ **-g-*.)

pMo **dalba-* 'to be wide and flat': MMo. *dalbaru* 'wide and flat' (PMo **-ru ~ rü* adverbializer), WMo. *dalbayi-* '1 to be wide and flat' (pMo **-yi-* anticausative), *dalban* 'wide, broad, flat' (pMo **-n* deverbal noun suffix), *dalbaɣar* (pMo **-ɣar ~ -ger* deverbal nominal adjectivizer), Khal. *dalbay-* '1', Bur. *dalba-* '1', Kalm. *dalwä:-* '1'

pTk **yalpa-* 'to be wide, flat’ in pTk **yalpa-k* ‘wide, flat’ (pTk **-(O)k* deverbal noun/adjective suffix, e.g. OT *yagu-* 'to come near' → *yaguk* 'near'): OT (Karakh.) *yalbɨ* '1 broad, flat', Tk. *yalpɨk* '1', Gag. *yalpaq* '1', Tkm. *yalpa* '2 blade, paddle', *yalpaq* '1, 3 shallow', Uz. *yɔlpɔq* '1', Uig. *yalpaq* '1', Khak. *čelbax*, *nalpax* '1', Tuva *čalbaq* '1', Tofa. *čalbaq* '1', Yak. *salbax* '2', Dolg. *halbax* 'span; foot', Tat. *ǯɛlpɛk* '1', Kirg. *ǯalpaq* '1', Kaz. *žalpaq* '1', Nog. *yalpaq* '1', Bash. *yalpaq* '1', Kpak. *žalpaq* '1', Kum. *yalpaq* '1', Karaim *yalpaq* '1'

97. STAR

pJ **pəsi* ‘star’: J *hosi* (2.1), OJ *posi* 'star'; Yamatohama (Amami) *husi*, Asama (Amami) *husɨɨ*, Yoron (Amami) *pusi*, Shodon (Amami) *husy*, Yonamine (Okinawa) pusii, Shuri (Amami) *husi*, Irabu (Miyako) *pusï*, Hirara (Miyako) *pụsï*, Ishigaki (Yaeyama) *fusï* [*pusï*](B) ~ *pusu*, Hatoma (Yaeyama) *pusi*, Yonaguni *huci* (A), pR **posi* 'star'

pK **peli* 'star': K *pye:l*, MK ¨*pyel* ‘star’ (Hyangka 12, 7. semantogram + li = ? MK¨ *pyeli*)

98. IN

pJ **soko* 'depth, bottom: J *soko* (2.1), OJ *so$_2$ko$_2$* ‘bottom, sole, depth’; Yamatohama (Amami) *suxu*, Asama (Amami) *syuku*, Yoron (Amami) *siki*, Shodon (Amami) *suku* Yonamine (Okinawa) *suкuu* ~ *siкuu*, Shuri (Okinawa) *suku*, Irabu (Miyako) *suku,* Ooura (Miyako) *suku*, Ishigaki (Yaeyama) *suku*, Hatoma (Yaeyama) *suku*, Yonaguni *sugu* (A) 'bottom', pR **soko* 'bottom'
pK **soko* 'depth, inside': K *so:k* 'inside, interior', MK *¨swok* ‘deep down, within, inside’

pMo **örü* ‘interior’: MMo. *oro* ~ *ore* '1 inside, interior, heart, abdomen' WMo. *örü* ~ *öri* '1', Dag. *erecu*: 'chest, bosom, breast' , Khal. *ör* '1', Bur. *üre* '1', Kalm. *örə* '1', Ordos *örö*, *ör* '1', Mog. *ourä* 'heart, mind'
pTk **ö:r$_2$*‘interior': OT *öz* '1 interior part of an organism, pith, marrow, spirit, self', Tk. *öz* '1', Az. *öz* '1', Tkm. *ö:z* 'self', Gag. *yöz* '1', Uz. *ọz* '1', Uig. *öz* '1', Yak. *üös* '1', Dolg. *üös* '1', Tat. *üz* '1', Kirg. *öz* '1', Kaz. *öz* '1', Nog. *öz* '1', Bash. *üδ* '1', Karaim *öz* '1', Kpak *öz* '1', Kum. *öz* '1', Khalaj *ẹ̄̂z* '1', Chu. *var* '1'

99. HARD
pJ **kata*- ‘to be hard, dense’: J *kata-* (A)*,* OJ *kata*- ‘to be hard, solid, tough, rigid’; Yoron (Amami) *hatasaN* 'thick (of liquid), dense (e.g. of seedlings), Yamatohama (Amami) *xaθasari* 'thick (of glue, gruel), densely packed (of hair, grass)', Nakijin (Okinawa) *hataaseN* 'strong (of tea), densely packed', Shuri (Okinawa) *katasaɴ* (A) ‘to be hard, sturdy, sure, saturated’, Irabu (Miyako) *kataham* 'thick (of liquid), dense (seeds, holes in a sieve)', Hatoma (Yaeyama) *kataaN* 'strong (tea), hard', pR * *kata*- 'to be gathered together in a dense fashion'
pK **kata*- ‘to be hard, severe’: K *kwut-*, MK *kwut*- ‘to be hard’, K *kkatalop-*, MK *skatalwop*- ‘to be hard, difficult, complicated; be harsh, severe’ (adj. n. + MK *-lwop*- ‘to be characterized by’; pK **s(u/o)-* intensive prefix)
pMo **kata*- ‘to become hard, dry’: WMo. *qata*- ‘1 to become hard, dry (intr.)’, *qata-ɣu* ‘2 hard’ (WMo *-ɣu* / *-gü* deverbal noun deriving quality words (Poppe 1954: 46)), MMo. *qata'u* ‘2’, *qətəmər* 'dried (meat)', Dag. *katən*, *katen*, *katu:* '1', Khal. *xat*- '1', *xatu:* ‘2’, Kalm. *xatə*- '1', *xatu:* '2', Ordos *ġatu:* '2', Kalm. *xatə*- '1', *xatu:* '2', Ordos *ɣatu:* '2', Dong. *qɨdun*, *qɨtun* '2', Bao. *χotoŋ* '2', Eastern Yugur *ɣadu:* 1, Mog. *xata* '2', Mgr. *xada:*- ‘1’, *xadoŋ* ‘2’
pTk **kat*- ‘to be hard’: OT *kat*- ‘to be hard, firm, though’*, katïg* ‘2 hard’ (OTk. *-(X)g* deverbal noun suffix), OT (Karakh.) *kat*- ‘1’, *katïg* ‘2’, Tk. (dial.) kat ‘2’, Az. *gatï* ‘2’, Tkm. *gat, gatï* ‘2’, Uz. *kətik* ‘2’, Uig. *ketik* ‘2’, Khak. *xatïɣ* ‘2’, Shor *kadïɣ* ‘2’, Tuva *ka'dïɣ* ‘2’, Yak. *kïta:nax* ‘2’, Dolg. *kïta:nak* ‘2’, Tat. *katï* ‘2’, Kirg. *katu:*‘2’, Kaz. *kattï* ‘2’, Nog. *kat* ‘2’, Bash. *katï* ‘2’, Kpak. *kattï* ‘2’, Kum. *katɨ* ‘2’, Chu. *xɨdă* ‘2’

In Korean, relatively retracted and non-retracted vowels alternate phonologically in certain color adjectives, mimetic and expressive adjectives, a phenomenon referred to as “ablaut” by Vovin (2008: 6) and as “heavy and light isotopes” by Martin (1992: 343-344). The non-retracted vowels *e*, *ey*, *wu*, *wi* (< MK *wuy*) are typical of the heavy isotopes, while the retracted vowels *a*, *ay*, *o* (MK *wo)*, *oy* (MK *woy*) are typical of the light isotopes. The non-retracted vowels are associated with weighty, bulky concepts, while the retracted vowels are used for small and unsubstantial things, e.g. K *ce:k*- ‘to be small in number or quantity, few’ vs. K *ca:k*- ‘to be small in size, tiny’[6]. It is not

[6] It can be noted that the interpretation of “heavy and light isotopes” in terms of a distinction between relatively high front versus relatively low back vowels (e.g. Martin (1992: 343-344) would suggest that lower vowels were used for smaller things. This runs counter the notions of “naturalness” of phonetic

surprising that the adjective meaning ‘to be large’ has a more advanced vowel in its default form K *khu-*. A trace of a retracted alternant, however, can be found in the obsolete adjective K *ha-* (< MK *·ho-*) ‘to be large in number, much, many, be great’, lexicalized, for instance, in K *hankul* ‘hankul, lit. great script’. Similarly, the stem meaning ‘to be hard’ has developed an advancedvowel in its default form K *kwut-*, MK *kwut-* ‘to be hard’, while there is a trace of a retracted —and probably original— alternant in the adjective with metaphorical meaning K *kkatalop-*, MK *skatalwop-* ‘to be hard, difficult, complicated; to be harsh, severe’. This form can be derived from **s-kata-lwop-* (INT-hard-be.characterized.by). The first element is the intensive prefix pK **s(u/o)-* > MK *s-* > K reduplication (Lee 1977: 145, Ramsey 1977: 64, Martin 1996: 24, 27, 91), e.g. MK *tih- ~ stih-* ‘to pound’.[7] The last element is the verbal adjective formant pK *-*lwop-* > MK -*lwop-* > K -*lop-* ‘to be characterized by’ (Martin 1992: 677), e.g. K *say* ‘new’ vs. *saylop-* ‘to be new’. Apophony between the non-retracted vowel *wu* and the retracted vowel *a* can be found in other adjective pairs, such as in K *phalah-*, MK *·pha·la ho-* ~ K *phwulu-*, MK *phwulwu-*, *phwulu* ‘be blue’, where it is used for its expressive effects only.

100. CRUSH / GRIND

pJ **pinta-ka-*‘to crush’ (pJ *-*ka-* deverbal inchoative, e.g. OJ *par-* ‘to open (ground), clear (land) (tr.)’ → *paruk-* ?B ‘to clear up, open up (intr.)’; Robbeets 2015: 255-256): MJ *fidak-* (A), OJ *pidak-* 'to crush'

pTg **pinče-* ‘to crush’: Even *hênčik- ~ ênčik- ~ hincuk-* 'to crush' (Even -*k* deverbal suffix; Benzing 1955b: 47), *hinčil- ~ hênčil-* 'to break down' (Even -*l* deverbal inchoative; Benzing 1955b: 44), Neg *xiŋčel-* 'to crush, break down'

pJ **sura-* 'to grind, rub': J *sur-* (B), *sure-* (B) ‘to rub against eachother’, OJ *sur-* 'to grind, rub', J *surari* ‘without trouble, smoothly’ (-*ri* adverbializer), *sura-sura* ‘without a hitch, smoothly’, Shuri *sir-* 'rub, grind', *ṣiyuŋ* 'to rub', Shodon *k'usryum*, Hirara *sïpagᶻï*, Ishigaki *sïsuŋ*, Kabira *suri*, Yonaguni *ccituŋ*, *ciruŋ,* pR **suri-* ~ **kosuri-* 'to rub'

pTg **suru-* ‘to grind: Ma. *šuru-* ‘to grind, whet, sharpen’

pTk **sür(ü)-* 'to rub, smear' (pTk *-*ti-* causative-passive; e.g. OT *arï-* ‘to be(come) clean (intr.)’ → *arït-* ‘to clean (tr.)’; Robbeets 2015: 290-292): OT *sürt-* '1 to rub, smear (tr.)', MT *sür-*, *sürüt-*, *sürt-* '1', Tk. *sür-*, *sürt-*, Az. *sürt-*, Tkm. *sür-*, *sürt-*, Gag. *sürüt-*, Uz. *surt-*, Tuva *sür-*, Yak. *ür-*, Khak. *sürt-*, Kirg. *sür-*, *sürt-*, Kaz. *sürt-*, Nog. *sür-*, *sürt-*, Bash. *hŭr-*, *hŭrt-*, Balk. *sürt-*, Karaim *sürt-*, Kpak *sür-*, *sürt-*, Kum. *sürt-*, Chu. *sĕr-*

In a few cases Manchu displays a palatal sibilant *š-* rather than *s-* in correspondence with words with initial *h-* in Even and initial *s-* in the other Tungusic languages. There is no internal ground for this palatalization, such as a following high vowel.

---

symbolism (a.o. Sapir 1929).

[7] Whereas Martin reconstructs pK **s(u/o)-*, Ramsey and Lee refer to the s-clusters as reinforced pronunciations that do not necessarily go back to an original silibant prefix. The authors agree that the verbs with s-clusters represent intensive meaning. The intensification was apparently restricted to processive verbs in Middle Korean. Other examples of such verb pairs are MK *kužu- ~ skužu-* ‘to pull’, MK *pipuy- ~ spi·puy-* ‘to rub’, MK *twutuli- ~ stwu·tuli-* ‘to beat’, MK *sip- ~ ssip-* ‘to chew’, MK *·sus- ~ ·ssus-* ‘to wash’, MK *kulh- ~ skulh-* ‘to boil’, MK *sa·hol- ~ ssa·hol-* ‘to chop’, MK *ku·cit- ~ skucit-* ‘to scold’. Instances of a descriptive verb pairs reflecting the intensive prefix are MK *polo-* (-*ll-*) ‘be straight, fast, act quickly’ vs. MK *·spol-* (-*ll-*) ‘be fast; be sharp, pointed’, MK *·so-* ‘be cheap’ vs. MK *·sso-* ‘id.’, MK *kel-* (*~¨ke(l)-*) ‘get stuck, obstructed’ vs. MK *¨skelW-* ‘be difficult’.

However, as it concerns only a few cases and since the palatalization is restricted to Manchu, Benzing (1955a: 989-990) refrains from establishing a separate palatal sibilant **š-* in proto-Tungusic. I do not consider Even *huruŋ-* 'to grind, pound, mash, pestle, crumble, divide in small parts', *huruwe:* 'bits and pieces, crumbs' to be related here. These forms derive from pTg **puru-* 'to crush', reflected in Evk. *huru-*, *hurgu-*, *horo-* 'crush', Ma. *furu-* 'to chop, cleave', Olcha *pori-* 'to crush', Na. *purtu* 'crumbs'.

pK **niki-* 'to crush to a pulp, knead': K *iki-*, MK *niki-* 'to crush to a pulp, mash, knead, beat water into flour'

pMo **niku-* 'to knead, crush': WMo. *niqu-* ~ *nuqu-* '1 to knead (flour), mash, crumple, rub, press, massage', *niquɣur* 'implement for kneading dough', MMo. *nuqu-* '1', Khal. *nuxa-* '1', Bur. *ńuxa-* '1', Kalm. *nuxə-* '1', Ordos *nuxu-* '1', Bao. *noġə-* '1', Dag. *nogu-* '1', Mgr. *nuġu-* '1', Mog. *nuqu-* ~ *noqu-* 'to crush', Dong. *nuqu-* 'to hit with force'

pTk **yïk-* 'to crush, demolish, destroy': OTk. *yïk-* '1 to crush, demolish, destroy', OT (Karakh.) *yɨq-* '1', Tk. *yɨk-* '1', Az. *yɨx-* '1', Tkm. *yɨq-* '1', Gag. *yɨq-* '1', Tat. *yɨq-* '1', Kirg. *ǯɨq-* '1', Karaim *yɨq-* ~ *yɨx-* '1', Kaz. *žɨq-* '1', Nog. *yɨq-* '1', Bash. *yɨq-* '1', Kpak. *žɨq-* '1', Kum. *jɨq-* ~ *jɨx-* '1', Uz. *yiq-* '1', Uig. *yiq-* '1', Khak. *yuq-* '1' , Oirat *yɨq-*, *d́ɨq-* '1', Khalaj *yuq-* '1', Chu. (dial.) *śăx-* '1'.

101. MOUNTAIN

pJ **yama* 'mountain': J *yama*, OJ *yama* (2.3) 'mountain', Asama (Amami) *yamaa* 'mountain', Irabu (Miyako) *yama* 'mountain', Ishigaki (Yaeyama) *yama* 'mountain', Yonaguni *dàma* 'mountain'

pK **yem* 'mountain': K *yem* 'small stoney island, small rocks sticking out of the water', K *yemso* 'goat', MK *·yem-·sywó* 'goat' (*·sywo* 'cow'), K *yemkyo* 'shallot', MK *·yem-kywo* 'scallion (*Allium chinense*)' (Sino-Korean 蕎 *kywo* 'onion')

This etymology has been recently suggested by Ratte (2016: 323-325). Given the comtemporary Korean meaning 'island consisting of rocks' and the preservation of the word in compounds such as MK *·yem-·sywó* 'goat' (mountain-cow) and MK *·yem-kywo* 'scallion' (mountain-onion), the reconstruction of the meaning 'mountain' is very likely.

102. TO SIT

pJ **wo-* 'to sit, be': J *iru* A, OJ *wi-* 'to sit, be' (< **wo* + **-(C)i-*), J *oru* A, OJ *wor-* 'to be, exist' (< **wo-* + **-ra-* anticausative), OJ *wos-* ?A 'deign to control/rule/eat/drink/ wear' (< **wo-* + **-sa-* causative), OJ *wo* focus marking in nominalized clauses of the type O-*wo* S-*ga* V, Yamatohama (Amami) *içjuri* 'to sit', Shuri (Okinawa) *jijuN* 'to sit', Shuri *ir-* A 'to sit', Shuri *un* 'to be, exist', Irabu (Miyako) *bïzi:du buz* 'to sit', Ishigaki (Yaeyama) *bïriɴ* 'to sit, settle', pR **wir-* 'to sit' (< **wo* + **-(C)i-*); Ryukyuan imperfect (ad)nominals e.g. Shodon *-un, -ur, -um* (< **wo* + nominalizer).

pK **o-* 'to be': MK *-·*w^{u}*/o-* modulator

pTg **o:-* 'to become, make': Evk. *o:-*, Even *o:-*, Neg. *o:-*, Ma. *o:-*, Sibe *o:-*, Jur. *o-fia*, Olch. *o-*, Orok *o-*, Na. *o-*, Ud. *o-* / *o:-*, Solon *o:-*,

pMo **bo(:)l-* 'to become': WMo. *bol-* 'become, take place, be, exist; be able, be possible', (SH/ HY/ Muq.) MMo. *bol-*, (IM) MMo. *bul-* 'to become, be; be possible', Khal. *bol-*, Bur. *bolo-*, Kalm. *bol-*, Ordos *bol-*, Dag. *bol-*, Eastern Yugur *bol-*, Huzhu Mgr. *olə-*, *o:li-*, Minhe Mgr. *boř-* 'to become', *bər-* 'to be able', Bao. *ol-*, Dong. *bolu*, *olu-* 'to be possible, be proper', Mog. *bol-*, Khitan **po-* 'to become, promote' (Kane 2009: 112)

pTk *(*b)o:l-* ‘to become’: OTk. *bol-* ‘to become’, Karakh. *bol-* ‘to become’, *ol-* ‘to be’, MTk. *bol-* ‘to become’, *ol-* ‘to be’, Khalaj *ộl-*, Tk. *ol-*, Az. *ol-*, Gag. *ol-* ‘to become’, Tkm. *bol-*, Tat. *bul-*, Uzb. *bụl-*, Uig. *bo(l)-*, Sary-Yughur *pol-*, Khak. *pol-*, Shor *pol-*, Oyr. *bol-*, Chu. *pol-*, Yak. *buol-*, Dol. *buol-*, Tuva *bol-*, Kirg. *bol-*, Kaz. *bol-*, Nog. *bol-*, Bash. *bul-*, Balk. *bol-*, Krm. *bol-*, KKalp. *bol-*, Sal. *vol-*, *vo:-*, *bo:-*, Kum. *bol-* ‘to become’

pTk **ol-* ‘to sit down, be sitting’ (**-Ur* /**-tUr* causative): Karakh. *oltur-*, Tk. *otur-*, Tat. *utɨr-*, MTk. *oltur-*, Uzb. *ụtir-*, Uig. *oltur-*, Az. *otur-*, Tkm. *otur-*, Khak. *odɨr-*, Oyr. *otur-*, Chu. *lar-*, Yak. *olor-*, Dol. *olor-*, Tuva *olur-*, Tof. *olɨr-*, Kirg. *otur-*, Kaz. *otɨr-*, Nog. *oltɨr-*, Bash. *ultɨr-*, Balk. *oltur-*, Gag. *otur-*, Krm. *otur-*, KKalp. *otɨr-*, Sal. *oht(ɨr)-* ‘to sit down, be sitting’

The Japanese verbs OJ *wi-* ‘to sit, be’, OJ *wor-* ‘to be, exist’ and OJ *wos-* ‘to deign to control/rule/eat/drink/ wear’ are usually derived from a common root followed by the suffixes **-(C)i-* causative-anticausative (Robbeets 2015: 315), **-ra-* anticausative (Robbeets 2015: 309) and **-sa-* causative respectively (Martin 1989: 698, 742, 743). The semantics of OJ *wos-* suggest a common semantic denominator ‘take possession of’, which can be interpreted as the causative of an existential auxiliary. Yanagida & Whitman (2009: 127-129, 134) find that the function of the object marker OJ *wo* in nominalized clauses of the type O-*wo* S-*ga* V is really a focus marker and that its relative position is such that it always precedes the subject. Therefore, they suggest that it has grammaticalized from an original copular verb. The original form of the copula is traditionally considered to involve an o_2 and is thus derived from pJ **wə-* according to Frellesvig & Whitman’s (2008) analysis. However, it may also be derived from pJ **wo*, because OJ makes no distinction between o_1 (< **o*) and o_2 (< **ə*) after *w*. Moreover, Pellard (2011: 10) advances evidence from Ryukyuan that the original shape indeed was pJ **wo-*. The expected reflex of pJ **wə-(C)i-* ‘to sit, be’ in Ryukyuan is pR **wer-*. In reality, however, attestations such as Shuri *jijuN* reflect an original pR **wir-* ‘to sit’ (Thorpe 1983: 328-29), which derives from pJ **wo-(C)i-* ‘to sit, be’. Taking into account grammaticalization theory (Heine & Kuteva 2002: 278), the primary meaning of pJ **wo-* should be reconstructed as ‘to sit’. We cannot exclude the possibility, however, that the polysemy ‘to sit, to be’ may already have been present in the proto-language ancestral to Ryukyuan and Japanese.

As proposed by Martin (1996: 13, 83; 2006: 222), the Korean cognate is the modulator MK *-·w*^{u}*/o-*. It is a bound stem that attaches itself to other verb stems and is more often than not followed by the (ad)nominalizers MK *-(·u/o)m*, MK *-(·u/o)l* and MK *-(·u/o)n* (Martin 1992: 269-273; Ramsey & Lee 2011: 205-207; Robbeets 2015: 347, 366, 383). These suffixes may appear either in the modulated or unmodulated form, e.g. MK *yel-um* ‘fruit’ and MK *yel-wu-m* ‘the bearing (of fruit)’ from MK *yel-* ‘to bear (fruit) (tr.)’. The modulated alternants are assumed to go back to a periphrastic construction consisting of copula and (ad)nominalizer (Robbeets 2015:160-161). Note that the Ryukyuan imperfect (ad)nominals, e.g. Shodon *-un, -ur, -um* are derived in a parallel way from an original copula pJ **wo* and an (ad)nominalizer.

Benzing (1955a: 120) and Doerfer (1965: 358) suggest a historical connection between pTg **o:-* ‘to become, make’ and the other Altaic forms. The open monosyllabic form with length in Tungusic may be indicative of original liquid loss, as it is in the etymologies for 4 WATER, 53 GIVE, 79 BLOW, 80 WOOD and 118 YEAR. Benzing assumes that the root-final liquid was dropped in Tungusic because it was reanalyzed as the inchoative suffix **-l*. The initial voiced labial stop may have

been lost before a long labial vowel, as it probably did in Korean as well. As far as the labial lenition is concerned, however, observing that Tungusic lacks words with initial **w*- followed by a labial vowel, Doerfer suggests that it went over **w*- to zero here.

The historical and contemporary Mongolic languages support the reconstruction of pMo **bo(:)l*- 'to become', which grammaticalized into a copula or into verbs of ability and possibility. The final vowel in Mangghuer and Dongxiang is epenthetic in nature (Nugteren 2011: 244-245). The Huzhu Monguor form *o:li*- may point to original vowel length in Mongolic. Note that there is no final liquid in the Khitan reconstruction **po*- 'to become, promote'.

The historical and contemporary varieties of Turkic support the reconstruction of pTk **bo:l*- 'to be(come)'. Vowel length is reflected in Yakut *buol*- 'to become'. There is sporadic elision of initial **b*- in the forms with the meaning 'to be' in Old Turkic and Middle Turkic. The *b*- was also elided in the verbs meaning 'to become' in Khalaj, in the Oghuz Turkic languages in the West and in Salar, probably through an intermediate form with *v*-. Sporadic lenition of initial **b*- is found in the same geographic distribution for other monosyllabic roots with non-high vowels and final liquids, such as pTk **be:r*- 'to give' reflected as Khalaj *ver*-, Tk. *ver*- , Az. *ver*-, Gag. *ver*-, Sal. *be(r)*-, *ve(r)*-, *ve:(r)*-, pTk **ba:r* 'existence' reflected as Khalaj *va:r*, Tk. *var* , Az. *var*, Gag. *var*, Sal. *ba:r*, *va:r* and pTk **bar*- 'walk' reflected as Khalaj *var*-, Tk. *var*- , Az. *var*-, Gag. *var* , Sal. *ba:r*-, *var*-. In view of the sporadic lenition of initial **b*-, the copular verb pTk **bo:l*- 'to be(come)' may be internally related to pTk **olur*- ~ **oltur*- 'to sit down, be seated, settle down, reside', an etymology that has been proposed by Räsänen (1969: 79, 360). The original verb stems are reflected in the alternation between OTk. *olur*- and *oltur*- 'to sit down, be sitting' and in historical and contemporary varieties reflecting one stem or the other.[8] The underived root pTk **ol*- 'to sit down, be sitting' may be reflected in Mongolic borrowings such as WMo. *olbuɣ* 'square cushion for sitting, mattress'. Clauson (1972: 150) has proposed to explain the Turkic reflexes of **oltur*- as instances as resulting from dissimilation of -*l*- to -*lT*-, but it is more likely to regard pTk **ol-ur*- ~ **ol-tur*- as derivations with the suffixes -*Ur* and -*tUr*, which both derive causatives in Turkic (Erdal 1991: 710, 799). Robbeets (2015: 290) shows that the lexicalization of causative suffixes, such as pTk *-*t(i)*- in Soy. *olït*- 'to sit, be sitting' is recurrent in posture verbs, suggesting the development of valence-neutral meaning such as intensive and progressive-resultative from original causative suffixes. If the posture verbs and copular verbs can indeed be related, grammaticalization theory would lead to the reconstruction of pTk **bo:l*- 'to sit down, be sitting; become, be', with static and dynamic posture as the original meaning and static and dynamic copula semantics 'to become, be' as the secondary meaning.

Given the polysemy of 'to sit, be' reconstructed for Japonic, it cannot be excluded that the grammaticalization from the posture verb 'to sit down, be sitting' into a copular verb 'to become, be' was already under way in proto-Transeurasian. The original polysemy seems to have left traces at the peripheries of the family, namely in Turkic and Japonic. The loss of the final liquid in proto-Japanese-Korean may be explained by the originally monosyllabic nature and long vowel of the verb pTEA **bɔ:l*- 'to take a position, become, be'. The same explanation can account for the development of pTk **bo:*- 'to become' into Sal. *vo*:-/ *bo:*-. Other studies reconstructing a common copular verb pJK **bo*- 'to be' or pTEA **bo(:)l*- 'to become'

[8] .

include Poppe 1960: 99, Menges 1968b: 145-146, Miller 1981: 851, Street 1985: 639, Kortlandt 1997, Starostin et al. 2003: 372-73 and Martin 2006: 222-223.

106. RIGHT

pK **palʌ*- 'to be at the righthand side': K *palu*- (*-ll-*), MK *palo*- (*-ll-*) 'to be to the right; be right, correct, honest; be direct, straight, immediate', K *palun* 'that is on the righthand side (modifier); the right side (noun)'

pMo **bara*- 'to be at the righthand side' (*-*ɣun* ~ *-gün* deverbal noun suffix for qualities and abstract nouns): WMo. *baraɣun* 'righthand side, right; west, westrightist (political)', MMo. *bara'un*, *barān*, *bărawun* '1 righthandside, right; 2 west, western', Khal. *baru:n* '1, 2', Bur. *baru:n* '1, 2', Kalm. *baru:n* '1, 2, southwest', Ordos *baru:n* '1, 2', Dgx. *borun* '1, 2', Dag. *baran*, *baren* '1, 2', Eastern Yugur *baru:n* '1, 2', Mgr. *baroŋ*, *waroŋ* '1, 2', Mogol *baranɣɔ̄l* '1, 2'.

Given the attestation of K *palay*- 'fade out, discolor, get washed out (intr.); fade, bleach, wash out (dye) (tr.)' (*-*i* causative) and WMo. *bara*- 'finish, expire, exhaust, wear out', WMo. *baraɣan* 'object visible in the distance, silhouette' and WMo. *baraɣan* 'dark', I do not exclude the possibility that the Transeurasian verb **bara*- 'to be at the righthand side' ultimately derives from a common verb **bara*- 'to fade out, to sink (of the sun)'

108. TO GRASP

pJ **tuka*- 'to grasp, hold' (pJ *-*ma*- deverbal inclinational suffix; Robbeets 2015: 248-250): J *tukam*-, OJ *tukam*- (B) 'to grasp, catch, hold', J *tuka*, OJ *tuka* (2.3)'hilt, grip, handle', Asama (Amami) *cɨkamjuɴ* 'to grasp', Yonamine (Okinawa) *sɪᴋaamiɴ* 'to grasp', Irabu (Miyako) *tskam* 'to grasp', Ishigaki (Yaeyama) *cïkamuɴ* 'to grasp, capture', Yonaguni *khácimirun* 'to grasp'

pK **tʌkʌ*- 'to receive': K *tha*-, MK *·tho* - 'to get, receive'

pTg **tuk*- 'to take into one's arms' (*-*i* resultative deverbal noun suffix): Evk. *tukin* 'carrying (a baby) on one's back, shoulders, arms', *tukihin*- 'to take on back, shoulders, into arms', Even *tok*- 'to carry on one's back; to take into one's arms', Olcha *tūwu(n)* 'an armful', *tūwu-lə*- 'to take into one's arms', *tūwu-lə-či*- 'to carry a baby in arms', Na. *tuxi* 'armful', *tuxi-lə*- 'take in one's arms', *tuxi-si*- 'carry in one's arms', Ud. *tugələ-nə*- ~ *tugələ*- ~ *tuələ*- 'to take into one's arms; to embrace'

The Old Japanese *tuka* 'hilt, grip, handle' may reflect a noun derived from the verb root underlying OJ *tukam*- (B) 'to grasp, catch, hold'. Note that this pattern of nominalization also underlies in derivations such as OJ *tuk*- (A) 'build' → OJ *tuka* (2.2) 'mound, hillock, tumulus', OJ *por*- (B) 'to dig' → OJ *pora* (2.4?) 'cave, cavern', OJ *nap*- (B) 'twist, make rope' → OJ *napa* (2.3) 'rope, cord' and OJ *tuk*- (B) 'be attached, come in contact' → OJ *tuka* (2.3/2.4?) 'bundle' (Robbeets 2015: 156).

In line with Ramsey (1993: 438; 1997), verb stems with complex initials that are tonic and monosyllabic and have minimal vowels (MK *o*, *u*, *i*) are thought to be created through the loss of a first-syllable vowel. Taking into account velar lenition (cf. 32 BIG, 55 BURN), we can reconstruct pK **tʌkʌ*- 'to receive'.

113. TWO

pJ **puta* 'two' (pJ *-*tu* substantivizer): J *hutatu*, OJ *putatu* 'two', Yamatohama (Amami) *taacï*, Shuri (Okinawa) *taaci*, Irabu (Miyako) *futaacï*, Ishigaki (Yaeyama) *ɸutaazï*, Yonaguni *ttaaci* 'two', pR **puta-tu* 'two.
pK **p(ʌ)cak* 'pair': K *ccak*, MK *pcak* 'pair'

114. BOTTOM
pJ * *sita* 'bottom, below': J *sita*, OJ *sita* (2.2a) 'under, lower, bottom', Yoron (Amami) sica 'below', Shuri sica 'below', Hirara (Miyako) *sïta* 'below', Ishigaki (Yaeyama) *sïta* 'below', Yonaguni *ttara* 'below'< **sita-ra*.
pK **s(i)ta* 'ground' (pK *-(*ɨ/ʌ)k* place suffix): K *ttang*, MK *·stah* 'ground'

The final -*h* in MK *·stah* 'ground' probably is a reflex of the place suffix *-(*ɨ/ʌ)k, e.g.* in MK *pas ~ pask* 'outside' (< **pasʌ-k*), (< **pasʌ-k*), *math* 'yard' (< **matʌ-k*), *alph* 'front' (< **alpʌ-k*), MK *path* '(dry) field' (< **patʌ-k*), MK *muth* 'dry land' (< **mutʌ-k* 'dry land'), etc. Forms with complex initials that are tonic and monosyllabic are likely to be the result of the loss of a first-syllable minimal vowel (MK *o*, *u*, *i*). Therefore, it is reasonable to reconstruct pK **s(i)ta-k* 'ground' whereby the choice of -*i*- as the lost minimal vowel is supported by the comparison with Japanese.

115. TO LIE DOWN
pJ **na*- 'to lie down': J *neru,* OJ *ne*- (A) 'to lie down, sleep', *nasu* 'to deign to sleep, put to sleep', Asama (Amami) nɨɴdui 'to lie down, sleep', Shuri (Okinawa) *niɴtooɴ* 'sleep (stative)', *niɴzuɴ* 'to lie down (dynamic)', Hirara (Miyako) *niv* 'to lie down, sleep', Irabu (Miyako) *nivvi buï* 'to lie down, sleep', Ishigaki (Yaeyama) *nibiɴ* 'to lie down, sleep', Yonaguni *níndun* 'to lie down, sleep'
pTg **ne:*- 'to lay down, put down': Evk. *nə:*- 'to put, to set; to select; to accept; to let down', Even *nə:*- 'to put', *nə:-ku* 'barn', *nə:-kči*- 'to save', Solon *nə:*- 'to put', Neg. *nə:*- 'to put, to set; to collect in one place; to bury', Na. *nə:*- '1 put, set, place; 2 leave, save; 3 nominate', *nə:-ktə*- 'to put, spread many objects', *nə:-ku*- '1 to put smth. back; 2 to keep smth. for future use; 3 to nominate smb again for the same position; 4 to marry a widow of a relative', Olcha *nə:- ~ nə:gbu*- 'to put', Ud. *nə*-'to put', Oroch *nə:*- 'to put, to place, to set'

118. YEAR
pK **selV* 'year, age': K *se:l* 'the New year (season)', *sel-nal* 'New years Day', MK *¨sel* 'the New year (season)', MK *¨sel* 'years of age', K *sal* 'years of age'
pTg **se:* 'year, age': Sibe *sə* 'age', Ma. *sə* '1 year (of age), age', Jur. *se* 'year', Olcha *sə:* 'age', Na. *sə:* 'age', Ud. *sə* 'year (age)', Oroch *sə*: '1 year (of age); 2 age of a person'

Whitman (1985: 235) analysed OJ *ko*$_2$*zo*$_2$ 'last year, tonight, last night' as a compound of the proximal demonstrative OJ *ko*$_2$- < pJ **kə*- 'this' and pJ **sə* 'year'. However this analysis is problematic because the OJ *ko*$_2$*zo*$_2$ means 'last year', not 'this year'; compare for instance with OJ *ko*$_2$*yo*$_2$*pi* 'this night', which is derived from the demonstrative and OJ *yo*$_2$*pi* 'night'. Since there is no unambiguous internal evidence for the reconstruction of pJ **sə* 'year', I have not included it here, even if it would make a regular fit to the Korean and Tungusic forms.

Similar to the etymologies 4 WATER, 53 GIVE, 79 BLOW and 80 WOOD, an open monosyllabic form with length in Tungusic corresponds to a disyllabic form

with a liquid onset in the second syllable in the other Transeurasian languages. This is indicative of liquid loss in Tungusic.

pMo **jil* 'year': WMo. *jil* '1 year', MMo. *jil* '1', Khal. *žil* '1', Bur. *žel* '1', Kalm. *jil* '1', Ordos *jil* '1', Dag. *jil* '1', Eastern Yughur *jəl* '1', Mgr. *ǯir*, *ǯur*, *jil* '1', Mog. *jil* 'time'
pTk: **yïl* 'year': OT *yïl* '1 year', Karakhanid *yïl* '1', MTk. *yïl* ~ *il* '1', Tk. *yïl* '1', Az. *il* '1', Tkm. *yïl* '1', Gag. *yïl* '1', Tat. *yïl* '1', Kirg. *jïl* '1', Kaz. *žïl* '1', Nog. *yïl* '1', Bash. *yïl*, Balk. *žïl*, Karaim *yïl* '1', Kkpak. *žïl* '1', Salar *yel* '1', Kum. *yïl* '1', Uz. *yil* '1', Uig. *yil* '1', S-Yug. *yil* '1', Khak. *čil* '1', Shor *čil* '1', Tuva *čïl* '1', Tofa. *čïl* '1', Yak. *sïl* '1', Dolg. *hïl* '1'

121. TO WEAVE
pJ **orə-* 'to weave': J *oru* A 'weave', OJ *oro*$_2$*s-* 'deign to weave', Asama (Amami) *ʔujuɴ* 'to weave', Shuri (Okinawa) *ʔujuɴ* 'to weave', Irabu (Miyako) *uï* 'to weave', Ishigaki (Yaeyama) *uruɴ* 'to weave', Yonaguni *úrun* 'to weave'
pK **olʌ* 'unit of woven fibers, component of woven fabric': K *o:l*, MK ¨*wol* 'strand of rope, ply, warp', K *olk-* 'to tie up, bind, weave' (< pK **olʌ* 'woven fabric' + *·kʌ-* inchoative; Robbeets 2015: 258)
pTg **poro-* 'to spin (nettle and hemp threads); to rotate, turn': Evk. *horol-* 'to spin, whirl, go around', *horoli:* 'around', Neg. *xoyil-* ~ *xoyol-* 'to spin; to circle; to eddy (about water)', *xoyil* ~ *xoyol*, *xoxsin* 'eddy', Ud. *xoli-* 'to circle (about birds); to circle, to whirl', Sibe *foro-* 'to spin; to turn', Ma. *foro-* 'to spin; to turn, to turn around, to face, to turn toward', *forko* 'spinning wheel', *foron* 'swirl, curl, whirl; rotation (of an arrow between the fingers)', *foro-no-* 'to turn (in that direction)', *forontu* 'curly, having curly hair', Olcha *pori-* 'to weave (nets)', *porpu(n)* 'a spindle; a device for weaving nets'; Oroch *porpụ* 'a spindle for spinning nettle and hemp threads'
pMo **poro-* 'to tie around, entwine; rotate, turn' in **poro-go-* 'to wrap' (**-gA-* causative) and **poro-ti-* 'roll, rotate' (**-ti-* intensive): WMo. *oriya-* '1 to tie around, entwine, wrap, bandage, wind, roll (tr.)', *oruya-* '1', *orči-* '2 to turn around, roll, rotate' (intr.)', MMo. *hura-* '1', *xorči-*, *horči-*, *orči-* '2', *orčul-* '2', Khalkha *orō-* '1', *orči-* '2', Buriat *ořo:-* '1', *oršo-* '2', Kalmuck *ora:-* '1', *orčə-* '2', Ordos: *oro:-* '1', *orčin* 'around', Dgx. *xoro-* '1', Baoan *horə-*, Dagur *oře:-*, Eastern Yughur *horo:-*, Mgr *furo:-*, *xuro:-* '1'
pTk **pö:r-* 'to plait, weave': OT (Karakh.) *ör-* 'to plait (hair or other fibers)', MTk *ör-* '1 to weave, plait, twist things together', *örmek* 'cloth woven from camel hair', Kirg. *ör-* '1', Kaz. *ör-* '1', *örïm* 'woven part of sth (e.g. of a whip); bundle', Nog. *ör-* '1', Bash. *ür-* '1', Karaim *ör-* '1; to spin', Kkpak *ör-* '1', Tat. *ör-* 'to plait, to knit, to darn, to interlace, to interweave, to build (a wall), to lay bricks or stones in a building', Tk. *ör-* '1', Az. *hör-* '1; to knit', Tkm. *ö:r-* '1', Gag. *yör-* '1; to knit', Uz. *ọr-* '1', Uig. *ö(r)-* '1', Yak. *ör-* '1', *örǖ* 'plaiting', Dolg. *ör-* 'to plait, bind together, wind', *örǖ* 'plaiting', Khalaj *hiri-*, *hör-* 'to plait', Chu. *var* 'best part; sort of fiber; flax', *vĕren* 'cord, rope'

The expected reflex of pTEA **p-* is **p-* in proto-Japonic and proto-Koreanic However, an initial labial stop sporadically drops before a (long?) rounded pJK **o(:)*, as it probably also did in the reflexes of pTEA **bɔ:l-* 'to sit down, become, be' in Japanese and Korean; see 102 TO SIT. Old Japanese makes no distinction between *o*$_1$ (< **o*) and *o*$_2$ (< **ə*) in initial position, but I have opted for **o* in pJ **orə-* 'to weave' because it entails a regular correspondence. The root-final vowel of pJ **orə-* is an irregular fit, which may be due to vowel reduction in root-final position.

The regular reflexes of pTg **p*- are Nanai/ Olcha/ Orok *p*-, Manchu *f*-, Evenki/ Even *h*-, Negidal/Oroch/ Udehe *x*- and Solon Ø (Benzing 1956: 981). Except for Oroch *po:rpu*, *po:rfu* ‘spindle’, which is probably a borrowing from Olcha, the cognates are thus corresponding regularly and suggesting the reconstruction of an initial pTg **p*-.

The initial labial stop pMo **p*- is regularly preserved in the peripheral Mongolic languages, notably as *f*- in Monguor *furō*-, as *h*- in Shira-Yughur *horō*- or Baoan *horə*- and as *x*- in Dongxiang *xoro*-, but it disappeared in the central Mongolic languages.

For Turkic, it is commonly assumed that word initial pTk **p*- developed over a bilabial fricative into *h*-, leaving only a trace in Khalaj *h*- and finally disappeared in most of the contemporary Turkic languages. Given the attestation of Khalaj *hör*- ‘plait’ it is legitimate to reconstruct pTk **pö:r*- ‘to plait, weave’.

Although the semantic association between 'to weave' and 'to spin' is not explicitely mentioned in List et al. (2014), the polysemy can be observed in Karaim *ör*- ‘to weave, plait; spin’ (Bakov et al. 1974: 442) in which the original meaning 'to weave' developed into 'to spin'. This is also the direction of the semantic shift assumed for the Tungusic and Mongolic proto-forms.

122. AT

pJ *-*tu* denominal suffix expressing locative relationship: OJ -*tu* genitive-locative marker, e.g. *pe*$_1$*-tu nami*$_1$ (seaside-GEN.LOC-wave) 'waves on the beach'.
pK **tʌy* locative postnoun: K *tey*, MK *·toy* 'place, case, circumstance', K *eti* 'where?', MK *e-·toy* 'where, which place?' (*e*- interrogative/indefinite)
pTg *-*du* dative-locative suffix (for recipient, goal, direction and location) (Benzing 1955: 79, 83): Even -*du* ~ -*tu* ~ -*duˁ* ~ -*tuˁ,* Solon -*du,* Evk. -*du* ~ -*tu,* Neg. -*du:* ~ -*tu:*, Na. -*do* / -*du,* Olcha -*do* / -*du*, Orok -*do* / -*du*, Ud. -*du*, Oroch -*do* / -*du*, Ma. *də*, Sibe -*d*, Jur. -*dolo*, e.g., Ud. *zugdi-du* 'in the house', *Bali-du* 'in the city of Khabarovsk', *kniga-du* 'in the book', etc. (Nikolaeva 1999: 75);
pMo *-*du* denominal suffix expressing locative relationship (Poppe 1955: 196, Gruntov & Mazo forthc.): WMo. -*du* ~ -*tu* ~ -*dü* ~ -*tü* denominal locative, dative-locative, e.g. *emüne-dü* ‘southern’ (*emüne* ‘front’), *ɣadaɣa-du* ‘outer’ (*ɣadaɣa* 'outside'), *morin-du* 'to the horse' (*morin* 'horse'), *ɣajar-tu* 'in the country' (*ɣajar* 'country'); MMo. -*du* ~ -*tu*, e.g. *aman-du* 'in the mouth', *ɣartu* 'on the hand'; Mog. -*du* ~ -*tu* dative-locative suffix; Mgr. -*du* dative suffix; Dgx. - *də*; Kangjia -*dʉ* ~ -*tʉ*; Oirat -*du* ~ -*tu* dative-locative suffix; Ordos -*du* ~ -*tu* dative-locative suffix; Kitan -*də* ~ -*dɔ* ~ -*du* dative-locative suffix

The genitive-locative suffix -*tu* is no longer productive in Old Japanese, although it occurs more frequently in early than in late Old Japanese texts (Vovin 2005: 152), e.g. *pe*$_1$*-tu nami*$_1$ (seaside-GEN.LOC-wave) 'waves on the beach'. The marker is not preserved in the Ryukyuan languages, except in one compound related to J matuge 'eyelashes' (< pJ **ma-tu-nkai* eye-GEN.LOC-hair), i.e., Shuri *maçigi*, Nakijin *maciigi*, Yamatohama *macïgï* and Ishigaki *macïni* 'eyelashes'.

In Written Mongolian and Middle Mongolian, the denominal locative suffix has an allomorph in -*tu* following all consonants other than *n*, *ng*, *l* and *m*. In Mogol, Ordos and Oirat, the allomorph -*tu* is restricted to the environment following *q*, e.g. Mog. *morin-du* 'to the horse' and *xoq-tu* 'to the earth'.

The lack of vowel harmony for the suffix in Mongolic, except Written Mongolian and Tungusic, supports the origin of the marker as a locative postposed noun meaning 'place, at', a status, which is preserved in Korean.

pTg *-*da*: locative postnoun 'side' (Benzing 1955: 1009): Even -*da* ~ -*də* ‘side, part’, e.g., in Even *amar-da:-n* 'backside', Even *jul-də:-lə:* 'in front', Evk. -*gida* ‘space, side’, e.g. *amar-gida* ‘backside’ (amar ‘back’), Neg. -*gida:*, -*da:* ‘side’, Solon *ami-da:-du* 'afterwards'
pMo *-*da* ~ *-*de* dative-locative (direction, location) (Poppe 1955: 195): WMo. -*da* ~ -*de* adverbial marker of time and space, e.g. *urtu-da* 'long time', *nasu-da* 'always', *en-de* 'here'; MMo. -*da* ~ -*de* dative-locative, e.g. *na-da* 'to me', *üile-de* 'to the work'; Khal. -*da* ~ -*ta* ~ -*de* ~ -*te* dative-locative; Bur. -*da* ~ -*ta* ~ -*de* ~ -*te* dative-locative; Kalm. -*da* ~ -*ta* ~ -*de* ~ -*te* dative-locative; Dag. -*da* ~ -*ta* ~ -*de* ~ -*te* dative-locative, Ordos -*da:-n* ~ -*ta:-n* ~ -*de:-n* ~ -*te:-n* dative-locative in reflexive-possessive declension
pTk *-*da* ~ -*de* locative suffix (Tenišev et al. 1997): OT -*DA* (-*t*- ~ -*d*- ~ -*a*- ~ -*ä*-) '1 locative', e.g. *köl-tä* 'at the lake'; Tk. -*DA (-d- ~ -t-, -a ~ -e)* '1', Az. -*dA* (-*a*- ~ -*ä*-) '1', Tkm. -*dA (-a ~ -e)* '1', Gag. -*DA (-d- ~ -t-, -a ~ -e)* '1'; Bash. -*DA (-d- ~ -t- ~ -ð- ~ -l-, -a- ~ -ä-)* '1', Tat. -*DA (-d- ~ -t-, -a ~ -ä)* '1', KBalk. -*dA (-a ~ -e)* '1', Karaim -*DA (-d- ~ -t-, -a ~ -e)* '1', Kkpak. -*DA (-d- ~ -t-, -a ~ -e)* '1', Kaz. -*DA (-d- ~ -t-, -a ~ -e)* '1', Khak. -*DA (-d- ~ -t-, -a ~ -e)* '1', Kirg. -*DA (-d- ~ -t-, -a ~ -e ~ -o ~ -ö)* '1', Kum. -*dA (-a- ~ -ä-)* '1', Nog. -*DA (-d- ~ -t-, -a ~ -e*) '1', Altay -DA (-d- ~ -t-, -a ~ -e ~ -o ~ -ö) '1', Uig. -*DA (-d- ~ -t-, -a ~ -ä)* '1', Uz. -*da* '1'; Shor -*DA (-d- ~ -t-, -a ~ -e)* '1', Tofa. -*DA (-d- ~ -t-, -a ~ -e)* '1', Tuvan -*DA (-d- ~ -t-, -a ~ -e)* '1'; Khalaj -*dA (-a ~ -ä)* ablative, Chu. -*RA (-r- ~ -t-, -a ~ -e)* '1'

123. EDGE
pJ **pasi* ‘edge, extremity’: J *hasi*, OJ *pasi* ~ *pazi* (2.1) ‘extremity, margin, verge’, Yoron (Amami) *pasi* 'edge', Yonamine (Okinawa) *pasii* 'edge', Yonaguni *haci* 'edge'
pK **pasʌ-k* ‘outside, beside' (pK *-(*i/ʌ)k* place suffix): K *pakk*, MK *pas* ~ *pask* 'outside (of a limit), exterior boundary, beside, other'

The final -*k* in MK *p* probably is a reflex of the place suffix *-(*i/ʌ)k,* e.g. in MK *math* ‘yard’ (< **matʌ-k*), *alph* ‘front’ (< **alpʌ-k*), MK *path* ‘(dry) field' (< **patʌ-k*), MK *muth* 'dry land' (< **mutʌ-k* 'dry land'), etc. In this case, the place suffix is maintained as -*k* following *s*; there are no **sh* clusters in Middle Korean. Forms with complex initials that are tonic and monosyllabic are likely to be the result of the loss of a first-syllable minimal vowel (MK *o*, *u*, *i*).

126. CHEEK
pJ **po* ‘cheek’: J *hoho*, OJ *popo* (2.3) ‘cheek’
pK **pol* ‘cheek’: K *pol*, MK *pwol* ‘cheek’

The Japanese word is generally taken as a reduplication. Rate (2016: 2018) explains the *-*r* loss in Japanese by a process whereby pJ **po(r)-por* reformed analogically to **po-po* by dropping final **r* in order to preserve the transparency of the reduplication.

129. HOLE
pJ **ana* ‘hole’: J *ana*, OJ *ana* (2.3) ‘hole’, Yoron (Amami) *ana* 'hole', Yonamine

(Okinawa) *ana* 'hole', Irabu (Miyako) *ana* 'hole', Hatoma (Yaeyama) *ana* 'hole', Yonaguni *ànanpu* 'hole'

pK **an* 'interior, inside' (pK *-(*ɨ/ʌ)k* place suffix): K *an*, MK·*anh* ‘interior, inside’

For the place suffix pK *-(*ɨ/ʌ)k* place suffix; see 123. EDGE.

130. TO GROW

pJ **ura*-‘to mature, ripen’ (*-*(C)i*- causative-anticausative): J *ure*-, OJ *ure*- (B) ‘to mature, ripen’

pTg **ure*- ‘to grow, ripen': Evk. *uruktu* ‘bush’, Sol. *urilə* ‘grain’, Ma. *ursan* ‘new shoots that sprout from old roots, new branches that appear on a tree that has been cut away’, *ursana*- ‘to sprout from old roots or an old stock’, Sibe *ursan* ‘seedlings, bystander branches’, *ursa-na*- ‘to revive’, Jur. *ure*- ‘to ripe’, *uri*- ‘to become ripe’, Na. *urə*- ‘to grow, ripen; to develop’, *urə-ktə* ‘willow rod’, Olcha *urə*- ‘to grow’, *urə-qtə* ‘a rod’, Orok *urəktə* ‘a rod’

pMo **ur-ga*- ~ **urgu*- 'to grow' (WMo. -*GA*- < pMo *-*gA* factitive; Poppe 1954: 61): WMo. *urɣu*- 'to grow, sprout; to appear, show up; to rise (of sun) (intr.)', *urɣuca* 'harvest, yield, crop', MMo. *urqu*-, *hurɣa*-, *urɣu*- '1 to grow', Khal. *urga*- '1', Bur. *urga*- '1', Kalm. *urɣə*- '1', *urɣəmər* 'harvest, yield, crop', Ordos *urGu*- '1', Dag. *orgu*-, *orege*-, *orgo*- '1', Mog. *urɣu*- '1', Eastern Yugur *uğarma* 'plant' (< **urga-ma*)

pTk **ur* ‘growth, excrescence': Tk. *ur* '1 growth, excrescence', Az. *ur* 'burl, wart (on a tree); tumor; crop', Tat. *ŭrɨ* '1', Kirg. *ur* 'burl, wart (on a tree), Bash. *ŭrŭ* 'burl, wart (on a tree); bump; nodule; outgrowth, excrescence', Nog. *urɨ* 'gland; excrescence', Kaz. *ŭra* 'bump, excrescence', Tuva *uru* '1', Shor *ur* '1', Oyrat *ur* '1', Yak. *ur* '1'

Nugteren (2011: 533) considers the Middle Mongolian form *hurɣa*- in the Muqaddimat al-adab as a secondary form. The Mongolic forms of the shape **ur-ga*- 'to grow' can be derived as a factitive derivation of an original root pMo **ur*- 'to grow', while the forms of the shape **urgu*- 'to grow' could represent an assimilation of the stem-final vowel to the preceding one. However, the derivation with a factitive suffix remains problematic because the resulting verb is not transitive.

132. BELLY

pJ **para* ‘belly’: J *hara*, OJ *para* (2.3) ‘belly, stomach, abdomen'

pK **pʌ(l)i* < **pʌ(l)ʌi* ‘belly’: K *pay*, MK·*poy* ‘belly’

MK·*poy* can be derived through liquid loss and vowel contraction from pK **pʌ(l)i* ‘belly’. Proto-Korean words of the shape *·*(C)V*(·)*li*, in which *V* is any vowel except the minimal vowel *ɨ* and has an initial high pitch, have lost the liquid and contracted to tonic words of the shape ·*(C)Vy* or ¨*(C)Vy* in Middle Korean. One illustrative doublet that bears an internal trace of the lost liquid is MK ·*pu*·*li*- ‘to practice (magic), summon a ghost; to make move (an object)’ vs. MK ¨*pwuy*- ‘to rub, twist’. Ratte (2016: 200) derives both verbs from the same original root and explains the alternations of *u* and *wu* in Middle Korean as a neutralization or confusion at an earlier stage. MK ·*pu*·*li* < *·*pɨ* ·*li*- < ·*pu* ·*lɨy*- neutralized the original vowel to *u* [ɨ] and therefore, retained the liquid, whereas MK ¨*pwuy*- < *·*pu* ·*li*- < ·*pu* ·*lɨy* preserved the original vowel *wu* [u] and therefore, lost the liquid and contracted the vowels. EMJ *purup*- ‘call forth, raise spirits; brandish an object, make move’ and OJ *purup*- ‘to sift, sieve’ < pJ **puru*- ‘to shake , brandish’ are probable cognates. Another potential

internal doublet is MK *·so(l)-* / *·so·l(o)-* < *·*sʌ·lʌ*- ‘to burn; melt, make vanish (tr.)’ and MK *·soy*- 'to leak, escape out (intr.)' < *·*sʌ·li*- < *·*sʌ·lʌy* < *·*sʌ·lʌ-i*- (make.vanish-INTR). OJ *sar*- (A) 'leave, depart' < pJ **sara*- is a likely cognate. Other Middle Korean words of similar shape in which external evidence sugests liquid loss are MK *·kwuy* ‘ear' < pK *·ku(l)i* 'ear' in comparison with pMo **kul* 'ear' and pTk **kul-kak* 'ear' (see 22 EAR); MK *·pi* ‘rain’ < pK *¨*puy* < *·*pu·(l)-i* (rain-NMLZ) in comparison with OJ *pur*- < pJ **pura*- 'to rain' and Orok *pure:*- < pTg **pur*- 'to drizzle' (Martin 1996: 47, 95; Starostin et al. 2003: 1105-1106); MK *·say* 'new' < pK *·*sa(l)i* in comparison with OJ *sara* (2.5.) 'new'.

In support of this process of liquid loss following high-pitched vowels other than **ɨ* , it can be observed that Middle Korean verbs ending on *-li* with initial low pitch display a variety of vowels in their intial syllable, e.g. MK *ka·li*- 'to branch off', MK *se·li*- 'to get steamed up', MK *wu·li*- 'to soak', MK *cwo·li*- 'to decrease, omit', MK *tu·li*- 'to hang down' and MK *no·li*- 'go down' belonging to accent Class 2.2 and MK *wuli*- 'to soak out', MK *kwuli*- 'to stink' and MK *huli*-'to be muddy' belonging to accent Class 2.0. By contrast, the large majority of Middle Korean verbs ending on *-li* with initial high pitch display a vowel *u* in their initial syllable, e.g. MK *·kuli*- 'to draw', MK *·kuli*- 'to miss', MK *·nuli*- 'to mark off', MK *·puli*- 'to work, use sth', MK *·tuli*- 'to humbly give', MK *·tuli*- 'to put in, dye', with exception of MK *·choli*- ~ *·chali*- 'to prepare, concentrate' and MK *·poli*- 'to crack open, break'. This observation suggests that following all other vowels than *u* the liquid got lost and made the verb root contract to one yodized syllable.

136. TO DIG

pJ **uka*- ‘to dig, excavate': J *ugat*- ‘to dig, excavate; pierce; put on’, OJ *ukat*- ‘dig, bore, wear’, OJ *uke₂*- ‘to open up, be bored’

pMo **uka*- > **uku*- ‘to dig, excavate’: WMo. *uqu*- 'to dig, excavate, poke out, chisel out; to cut out; to find or expose someone's fault', *uqumi* 'carving tool, chisel, cutter, gouge', *uqara* ~ *uqur* 'spoon'; MMo. *uqu*- 'to dig, delve', *uqali* ' axe', Khal. *uxa*- ' to dig, dig out, cut out; to dig into (e.g. past); to burgle, break in', *uxam* 'excavation, pit, cavity; gouge', *uxmal* 'dug out, carved out'; Bur. *uxami* ' adze', Kalm. *uxə*- 'to dig, delve', Ordos *uxa*- 'to dig, delve'.

138. HOT

pJ **yu* 'hot water': J *yu* (1.3a), OJ *yu* ‘hot water’, Shodon (Amami) *yu*, Shuri (Okinawa) *yu:*, Hirara (Miyako) *yu:*, Ishigaki (Yaeyama) *yu:,* Yonaguni *du:*, pR **yu* 'hot water' (Thorpe 1983: 298)

pK * *tʌ*- ~ **tɨ*- ~**ta*- ~ **te*- ‘to be hot, warm’: K *te:p*- (*-w-*), MK *¨teW*- ‘'be hot, warm', (pK *-*pɨ*- ~-*pʌ*- suffix deriving verbs from property words ‘to spontaneously develop the property expressed by the base’; Robbeets 2015: 294), K *ttakap*- ~ *ttukep*- ‘be hot, heated, burning’ (*-kep*- adjective suffix), *ttakkun ha*- ‘be hot, heated’, K *ttattus ha*- ‘be warm, mild’, K *ttasu ha*- ‘be warm, mild’, K *ttasup*- ‘be nice and warm’, MK *toso*-, MK *toso ho*-, MK *tosi ho*- ‘be warm, mild’, MK *tostosi* ‘warmly’, MK *tusi* ‘warmly’ (pK *-*s* resultative deverbal noun suffix, e.g. K *cilki*- ‘to be tough, be durable, be lasting, be persisting’ → *cilkis* ‘firm, unyielding’; Robbeets 2015: 422, pK *- *i* adverbializer)

pTg **du:l*- 'to be warm': Evk. *du:l*- ~ *dulil*- ‘to rise (about the sun); to warm (about the sun)’, Even *dụ:l*- ‘to be warm (about weather)’, *dụ:lan* ‘warm (wind, air, day)’, *dụ:lan*- ‘to get warm', Jur. *dulu'u* ‘warm (of spring)’

pMo **dula*- 'to be(come) warm' (WMo. -*GAn* resultative deverbal noun suffix, e.g.

uda- 'to tarry' → *udaɣan* 'slow'): WMo. *dulaɣan* 'warm, heated (not of liquids); warmth', *dulaɣara-* 'to become warm (of weather and room temperature) (intr.)'; MMo. *dula'an* 'hot, sunheat', Khal. *dula:n* 'warm; heat, warmth; temperature'; *dula:ra-* 'to get warm, warm up (weather)', Bur. *dula:n* 'warm', Kalm. *dul* 'warm; warmth', *dula:n* 'warm', *dula:ra-* 'to get warm, be warmer (of weather), warm oneself', *dulwus* 'light of the warming son', *dulwä:-* 'to give warmth (of the sun)', Ordos *dul* 'without wind and not cold', *dula:n* 'warm', Dag. *dɔ:l-* 'to become warm (of weather)', *dula:n* 'warm', Eastern Yugur *dula:n* 'warm' (Nugteren 2011: 319).
pTk **yïlï-* 'to be(come) warm' (OTk. *-(X)g* deverbal noun suffix, e.g. *biš-* 'to ripen, reach maturity; cook (intr.)' → *bišig* 'ripe, mature; cooked' ; Erdal 1991: 172–232): OT *yïlïɣ* '1 warm', Karakhanid *yïlï-* 'to become hot', *yïlïɣ ~ ïlïɣ* '1, lukewarm', MTk. *yïlïq ~ ïlïq*, Tk. *ïlïk* 'tepid, lukewarm', Az. *ilik̦* 'warm, warmish', Tkm. *yïlï* '1', Gag. *ïlï* 'lukewarm', Tat. *jïlï* '1', S-Yug. *ilïɣ* '1', Kirg. *jïluu* 'warm, kind, tender; warmth, soul, nest', Kaz. *žïlï* 'warm, kind, tender; warmth', Nog. *yïlï* '1', Bash. *yïlï* 'warm, hot (of meal); warmth', Balk. *žïlï*, Karaim *yïlï ~ ïlï* 'warm; lukewarm', KKalp. *žïllï* 'warm, kind, tender; warmth', Kum. *yïlï* 'warm, kind, tender', Sal. *yili* '1', Uz. *iliq* '1', Uig. *ilman* '1', Tofa. *čïlïɣ* '1', Tuva *čïlïɣ* '1', Khak. *čïlïɣ* 'warm, kind, tender; warmth, warm weather, kindness, tenderness', Shor *čïlï-* 'to become warm', Oyrat *d́ïlu* '1', Yak. *sïlās* '1', Dolg. *hïlās* '1'

As in the above etymology, the alternation between pK * *tʌ-* ~ **tɨ-* ~**ta-* ~ **te-* 'be hot, warm' is probably expressive in origin

Given the occurence of the Written Mongolian resultative deverbal noun suffix -*GAn*, it is inviting to reconstruct an underived verbal form pMo **dula-* 'to be(come) warm'. This simplex verb root is further supported by the attestation of the verb Dag. *dɔ:l-* 'to become warm (of weather)' as well as by underived nominal forms such as Kalm. *dul* 'warm; warmth' and Ordos *dul* 'without wind and not cold'. Moreover, Kalmuck displays other derivations from the original simplex root such as *dulwus* 'light of the warming son', *dulwä:-* 'to give warmth (of the sun)'.

Similarly, most Turkic forms are derived with the deverbal noun suffix *-(X)g* and allow the reconstruction of a verbal form pTk **yïlï-* 'to be(come) warm', which is reflected as such in Karakhanid *yïlï-* 'to become hot' and Shor *čïlï-* 'to become warm'.

This etymology assumes stem-final **-l-* loss at a time when Japano-Koreanic separated from Altaic, similar to the liquid loss found in 101 SIT.

140. TO REMAIN

pJ **nəkə-* 'to remain, be left over': J *nokor-*, OJ *no₂ko₂r-* (B) 'to remain, be left over, survive', *nokos-*, OJ *no₂ko₂s-* (B) 'to leave behind, keep back, leave undone', Yamatohama (Amami) *nohoruri* 'to remain', Shuri (Okinawa) *nukujuɴ* 'to remain', Irabu (Miyako) *nukuï* 'to remain', Ishigaki (Yaeyama) *nukuruɴ* 'to remain'
pK **nek* 'remnant': K *nek-nek* 'sufficient', *nekul-nekul* 'generous'

pJ **ama-* 'to remain, be left over': J *amaneku* 'widely, generally', J *amari*, OJ *amari* (3.4) 'rest, surplus, excess'; J *amar-*, OJ *amar-* (B) 'to remain, be left over; be in excess, be too much', J *amas-*, OJ *amas-* (B) 'to excel, be in excess, increase', Yoron (Amami) *amajuN* 'be left over', Yamatohama (Amami) *amaruri*, Nakijin (Okinawa) *?amaaruɴ* 'be left over; surpass', Shuri (Okinawa) *qamar-* 'be exessive', *amas-* 'to let remain', Hatoma (Yaeyama) *amaruN.*
pTg **eme:-* 'to leave, remain' (pTg *-*-nA* processive, e.g. Evk. *kiki:-* 'to whistle' → *kikine-* 'to howl (of wind)'; Robbeets 2015: 230-235; pTg **-p-* reflexive-anticausative,

e.g. Even *beri:*- 'to lose' → *beri:b*- 'to get lost, disappear'; Robbeets 2015: 296-298): Evk. *əmə:- ~ əmə:n*- ‘to leave; to lose’, *əmə:ntə* ‘remnant; forgotten’, *əmə:p*- ‘to stay; to stay behind’, Even *əmən*- ‘to leave, to quit’, *əməb- ~ əməp*- ‘to stay; to stay behind’, Neg. *əmə:n*- ‘to leave, to lose’, Sol. *əmə:n*- ‘to leave’, Oroch *imən*- ‘to leave smb in the care of smb’, Ud. *imənə*- ‘to leave’,

141. COLD

pTg **bege*- 'to be cold, freeze' (**-n* deverbal noun): Evk. *bəgi:- ~ bəi:*- ‘to get cold; to freeze’, *bəgin ~ bogin* ‘a cold; freezing’; Even *bəyi*- ‘to freeze’, *bo:ɣə* ‘frazil’, Neg. *bəyi:*- ‘to freeze smth.’, Ma. *bəyə*- ‘to freeze, to be cold’, *bəyən* ‘freezing, cold’, Sibe *bəyə*- ‘to feel cold’, Olcha *bəyi*- ‘to be cold’, Na. *bəi*- ‘to freeze smth. (cheeks, ears, etc.)’

pMo **bege*- 'to be cold' (**-rA*- anticausative, e.g. WMo. *ebde*- 'to destroy' → *ebdere*- 'to fall to pieces'; Robbeets 2015: 313; **-dA*- passive, e.g. *dugul*- 'to hear' → *dugulda*- 'to be heard'): WMo. *begere*- 'to suffer extremely from frost, freeze, become stiff or numb from cold (intr.)', WMo. *begelei* 'glove(s), mitten(s)', WMo. *begdere*- 'to be left stupefied, to be paralyzed with fear', Khal. *be:re*- 'to be cold', Bur. *be:re*- 'to be cold', Kalm. *be:r*- 'to become stiff (due to age or cold)', Ordos *be:re*- 'to be cold', Dag. *be:re- ~ be:de*- 'to be cold'

In Mongolic the root **bege*- 'to be cold' can be inferred from derivations with the anticausative suffix **-rA*- such as WMo. *begere*- 'to suffer extremely from frost, freeze, become stiff or numb from cold (intr.)', literally 'to colden oneself' and with the passive **-dA*- such as WMo. *beg-de-re*- 'to be left stupefied, to be paralyzed with fear' and Dag. *be:-de*- 'to be cold'.

pTg **gilči*- 'to be cold (tactile)' (**-si* deverbal resultative nominalizer; Robbeets 2015: 423; **-rA* deverbal (ad)nominalizer; Robbeets 2015:349-352; pTg **-lu*- deverbal inchoative; Robbeets 2015: 259): Evk. *gildi ~ gilli ~ gilləmə ~ gildəmə* ‘shiny (about water, ice surface); cold (about water, ice)’, *gildihi:* ‘clear (about water)’; Even *gịlra*, *gịlsị* ‘cold (about liquids)’, *gịlral*- ‘to become cold (about water, weather)’; Sol. *getti*- ‘to freeze’; Neg. *gelegdi ~ giligdi*, *gelesi ~ gilisi* ‘cold (about liquid); it is cold (because of the cold water)’, Olcha *gitisi* ‘cold’, *gituli ~ gituwli* ‘cold (tactile)’, Orok *ɢịtčuḷị ~ ɢịčuḷị* ‘cold (about liquids/tactile)’, *gịtčịlụ*- ‘to become cold’; *ɢịtčịsị- ~ ɢịčịsị*- ‘to get cold’; Na. *geče* ‘it is cold (tactile)’, *gečena*- ‘to become cold’, *gečese:* ‘cold’, *gəcəse*- ‘to feel cold, to be cold’; Oroch *giči-si* ‘cold’, Ud. *gilihi* ‘cold (about objects and liquids); cold (adv.)’, *gilili*- ‘to get cold’ (Ud. *-li*- deverbal inchoative), Jur. *geti*- ‘to freeze’

pTk **kïš* 'winter': OT *kïš* '1 winter', Karakhanid *kïš* '1', Tk. *kïš* 'winter, winter cold', Az. *ġïš* '1', Tkm. *ġïš* '1', Tat. *kïš* '1', Khak. *χïs, xïsχï* '1', Kirg. *kïš* '1', Kaz. *kïs* '1', Nog. *kïs* '1', Bash. *kïš* '1', Balk. *kïš* '1', Karaim *kïš* '1', KKalp. *kïs* '1', Kum. *kïš* '1', Uz. *kiš* '1', Uig. *kiš* '1', Tuva *kï'š* '1', S-Yug. *kïs* '1', Shor *kïškï, kïš* '1', Yak. *kïs*, *kïhïn* '1', Dolg. *kïhïn* '1', Khalaj *kïš* '1', Chu. *xəl* '1'

144. THIN

pMo **nari*- 'to be thin' (**-GAn* resultative deverbal noun suffix, e.g. *uda*- 'to tarry' → *udaɣan* 'slow'; **-d*- fientive; e.g. *sula* 'weak' → *sulad*- 'to be weak'; **-n* deverbal noun suffix, e.g. MMo. *ayu*- 'to be afraid' → *ayun* 'fear'; Robbeets 2015: 391-393): WMo. *narid*- 'to become thin, slender, narrow, tight', *narin* 'fine (not coarse); narrow; tight; thin, slender; high-pitched', *nariqan* 'rather fine, thin, slender'; MMo. *narin*, *narən*,

na:ri:n 'thin'; Khal. *nari:d-* 'to be too narrow, too fine', *nari:n* 'thin, fine', Bur. *narin* 'thin, fine', Kalm. *närn̥* 'thin, fine', Ordos *nari:n* 'thin, fine'. Dgx. *naruŋ* 'thin, fine', Bao. *naroŋ* 'thin, fine', Dag. *narin*, *narien* 'thin and cylindrical, slim, slender', *narie-eme* 'millet' (ame 'grain'), Eastern Yugur *narən* 'thin, fine', Mgr. *narin*, *narəŋ* 'thin, fine', Mogol *no:rin*, *nɔrin* 'thin, fine'

pTk **yarï-* 'to be thin, poor' (OT *-I* deverbal noun suffix, e.g. *kön-* 'to be straight' → köni 'straight' ; Erdal 1991: 340; OT *-lXg* denominal adjective suffix 'possessing the quality of the base', e.g. *küč* 'power' → *küčlüg* 'powerful'; Erdal 1991; 139): OT *yarlïg* 'poor, destitute', Karakhanid *yarlïg* '1 poor, destitute; 2 miserable, pathetic', MTk (Kypchak) *yarlï* '1', Tkm. *yarlï* (dial.) '1', Tat. *yarlï* '1; 2', Tat. *yarï* 'thin film, membrane', Nog. *yarlï* '1, 2', Bash. *yarlï* '1', Bash. *yarï* 'thin film, membrane', KBalk. *ǰarlï* '1', Karaim *yarlï* '1, 2', KKalp. *žarlï* '1', Kum. *yarlï* '2', Kirg. *ǰaro:* 'lean, skinny (of an animal)', *ǰardï* ~ *ǰarlï* ~ *ǰarïlï* '1', Kaz. *žara-* 'to be poor', *žar(ï)lï* '1', Tuva *čarïɣda-* 'to spend, consume', Chu. *yur*, *yură* 'poverty'

Both the Mongolic and Turkic forms can probably be derived from original property verbs, but even if this would not be the case, the etymology would remain valid. I regard the Turkic forms going back to OT *yarlïg* 'poor, destitute' as an adjective in *-lïg* from the deverbal noun in *-ï/ø* from pTk **yarï-* 'to be thin, poor'. The Chuvash word Chu. *yur* ~ *yură* 'poverty' reflects the underived deverbal noun pTk **yar(ï)* 'poor, poverty'. It is also witnessed in Bashkir and Tatar *yarï* 'thin film, membrane', preserving the original meaning 'to be thin'. The common meaning 'thin' is also present in Kirg. *ǰaro:* 'lean, skinny (of an animal)'. In support of the deverbal derivation, it can be noted that the suffix *-lXg* is more frequently added to deverbal nouns than to underived nouns, e.g., *isi-* 'be hot' → *isi-ndi* 'heated (thing)' → *isi-ndi-lig* 'heated' (Erdal 1991: 152). In addition, the Kazakh descriptive verb *žara-* 'to be poor' supports the deverbal derivation suggested here.

147. SOUR

pJ **su-* 'to be sour': J *su-* 'to be sour, acid, tart', OJ *su-* B 'to be sour, acid, tart', J *su*, OJ *su* (1.3) 'vinegar, sour', OJ *suye-* (B) 'to turn sour, spoil (intr.)', Yamatohama (Amami) *sïïsari* 'sour', Shuri (Okinawa) *siisaɴ* 'sour', Irabu (Miyako) *sïvkam* 'sour', Ishigaki (Yaeyama) *sïïsaaɴ* 'sour', Yonaguni *ccan* 'sour'

pK **sɨy-* ~ *sʌy- 'to be sour': K *swi* -, MK *soy-* ~ *suy-* 'to be sour'

149. DAY

pK **nal* 'day': K *nal* 'day, 24 hours, weather', MK *·nal* 'day'

pMo **nara-n* 'sun': WMo. *nara(n)* '1 sun', MMo. *naran*, *narăn* '1', Khal. *nar(an)* '1', Bur. *nara(n)* '1', Kalm. *narn̥* '1', Ordos *nara(n)* '1', Dgx. *naran* '1', Bao. *naraŋ* '1', Dag. *nar*, *nare* '1', Eastern Yugur *naran* '1', Mgr. *nara* '1', Mog. *naran*

152. WHITE

pJ **sero-* ~ **sera* '(to be) white': J *siro-* B, OJ *siro*$_1$- 'to be white', J *siro* (2.5), OJ *siro*$_1$ 'white', J/ OJ *sira-* in e.g. J *sirakami,* OJ *sira-kami*$_1$ 'white hair', Yamatohama (Amami) *sirusar* 'white', Shuri (Okinawa) *sirusaɴ* 'white', Irabu (Miyako) *ssukam* 'white', Ishigaki (Yaeyama) *ssusaaɴ* 'white', Yonaguni *ccudari* 'white', pR **siro-* 'white' (Thorpe 1983: 347), OKog **tśiar* 'silver' (Beckwith 2004: 100, 112)

pK **siala-* 'be white': MK *syey-* 'to become white (of hair, of face)', K *hay-* 'be white (preserved only in compounds)' ~ K *huy-* 'be white, be fair; be gray, hoary', MK *·hoy-*

~ MK *·huy-* ‘to be white’, K *ha:yah-* ~ *he:yeh-* , MK *·ha·ya ho-* 'be very white, be pure (snow) white, be immaculate'
pTg **sia:ra-* ‘to be light, white’: Ma. *šara-* ‘to become white’, *šari* ‘1 a kind of wild lettuce with edible leaves and roots; 2 bright, shining’, *šariša-* ‘to flash white, light up’, Sibe *šarambi* ‘white hair’, *šarinǯa-* ‘to become white’, Evk. *se:ru:-* ~ *he:ru:-* ‘to sparkle with all colours’, *se:run* ‘a rainbow’ (pTg **-n* deverbal noun; Robbeets 2015: 385-390), *serəmə* ~ *sərəmə* ‘yellow’ (pTg **-mA* nominalizer; Robbeets 2015: 367-374), Orok *se:rro* ~ *sịro* ‘rainbow; half-moon’
pMo **siara* ‘yellow’: WMo. *sira*, MMo. *šira*, *šira:*, Khal. *šar*, Bur. *šara*, Kalm. *šarə*, Ordos *šara*, Dgx. *šəra*, *šira*, Bao. *šira*, Dag. *šara*, *šar*, *šari*, Mgr. *śira*, Mogh. *šira*, *sira:* ‘yellow’
pTk **sia:rï-* ‘to be white, yellow’ (pTk **-(X)g* deverbal noun suffix; Erdal 1991:172-232): OTk. *šarïg*, *sarïg* ‘1 yellow’, Karakh. *sarɨɣ*, MTk. *sariɣ*, Tk. *sarɨ* '1', Az. *sarɨ* '1', Tkm. *sa:rɨ* '1', Tat. *sarɨ* '1', Uz. *sariq* '1' Uig. *seriq* '1', SYug. *sarɨɣ* '1', Tuva *sarɨɣ* '1' , Kirg. *sarɨ* '1', Kaz. *sarɨ* '1', Bash. *harɨ* '1', Sal. *sarɨ* '1', Khalaj *sa:ruɣ* ‘orange’, Chu. *šur*, *šură* ‘white’, *šur-* ‘to become white’

The seven vowel system of proto-Japanese, recently introduced by Frellesvig and Whitman (2008) proposed a double origin for OJ *i*; the front vowel derives either from pJ **e* or from pJ **i*. External etymologies reflecting pJ **e* are rather few in my dataset, but there seems to be a correspondence with diphtongs such as pK **ie*, pTg **ia*, pMo **ia* and pTk **ia*; see sound correspondence 40b in SI 1. In the case of OJ $siro_1$- ‘be white’, there is no internal or Ryukyan evidence, supporting the reconstruction of a mid front vowel. However, Frellesvig and Whitman (2008: 37) take the attestation of MK *syey-* ‘become white (of hair, of face)’ as external evidence for the reconstruction of pJ **sero* ‘white’. The Koguryo cognate OKog **tśiar* ‘silver’ proposed by Beckwith, while semantically rather distant, would support the high front vowel, early in pre-Japonic .

In Korean, we find a number of related verb stems such as MK *·hoy-* ~ MK *·huy-* ‘to be white’ and MK *·ha·ya ho-* 'be very white, be pure (snow) white, be immaculate'. MK *·ha·ya* 'white' can be considered as the nominal encoding of the verbal adjective MK *·ha·ya-* 'to be white'; see Robbeets 2015: 94-95). Although Martin (1996: 36) and Whitman (1999) reconstructed an original **s-* underlying these Korean forms, the current opinion tends towards original **h-* developing into s- (Whitman 2012, Francis-Ratte 2016: 425-426). Given that the sound development s > h is cross-linguistically more frequent than the other way around and given the words with initial **s-* corresponding to Korean words with initial **h-* in Japanese and the other Transeurasian languages, I think that the original sound was *s-, not **h-*. I propose the following development in Korean, whereby the fientive ending **-i-* in the derived verb blocked the development from the palatalized sibilant **si-* into **h-*. MK *o* [ʌ] was the RTR variant of the high central vowel MK *u* [ɨ] and in most cases it comes from a weakened a. Therefore, I suggest a development from pK **haya-* 'be white' into MK *·hoy-* with an expressive vocalic alternation in MK *·huy-*.

pK **siala-* (be.white) > **hala-* > **haya-* in MK *·ha·ya ho-* > MK *·hoy-* ~ MK *·huy-*
pK **siala-i-* (be.white-FIENTIVE) > pK **siələy-* > pK **siəyəy-* > MK *syey-*

In comparison with other syllables with a palatalized onset, there is a disproportionally high number of syllables of the shape Cye in Korean. This observation makes the origin as an original diphtong more likely.

It is clear that Tungusic forms such as Ma. *sira* ‘yellow’, Even *hiraŋan* ‘yellowish (of Reindeer skin)’ and Ud. *si:* ‘yellow paint’ are copies from Mongolic (Miller & Street 1975: 133, Doerfer 1985: 302, Rozycki 1994: 184). These are nominal forms with high front vowels meaning ‘yellow’. However, the Tungusic stems proposed in the etymology can be derived from verbally encoded adjectives, reflect pre-glided low vowels and share the meaning ‘to be light’ or ‘to be white’.

Similar to the vowel development of pMo **čagaan* 'white' into MMo. *čiɣa: n,* Dag. *čiɣa:(n)*, Mgr. *ćiġa:n,* Bao'an *ćiġaŋ* and Kgj *čiχɔ* (Nugteren 2011: 298), it cannot be excluded that the form giving rise to MMo. *šira* 'white' had a palatalized initial of the shape **šara* ~ **siara*. The proto-Mongolic form may be borrowed from Turkic because the Mongolic words are restricted to the meaning ‘yellow’ and cannot be derived form verbally encoded adjectives. Contrary to Doerfer (1963: 220-221) who considers the parallel between the Mongolic and Turkic forms as a coincidence, Georg (2007: 274) explains it in terms of a loan connection. He finds that “adding Japanese to this does not lead to any serious objections on the semantic side, but the vowel does not fit the Turkic etymon (which is the source of Mongolian here.)” But if Turkic indeed is the loan source of Mongolian, this indicates that the model was pTk **sia:ra*- ‘yellow’ with a diphtong that matches the *-*e*- in the Japanese etymon.

Since the formant *-(X)g* is very frequent in deverbal nouns in Old Turkic, e.g. OTk. *isi*- ‘to be hot’ -> *isi-g* ‘hot; heat’, the adjective pTk **siarï*- ‘to be white, yellow’ may originally have been verbally encoded. This is supported by the Chuvash descriptive verb *šur*- ‘to become white’. The palatal sibilant in Chuvash, Khazar and in the Hungarian loanwords *šār* ‘yellow’ and *šārgå* ‘yellow’, as well as the palatalized variant Orkhon OTk. *šarïg* support the diphtong in the reconstruction.[9] Note that Róna-Tas et al. (2011: 691-695) propose that the West Old Turkic models underlying H *šār* ‘yellow’ and *šārgå* ‘yellow’ are WOT **šarï* and **šarug*, respectively, but they derive both forms from a single origin **si̯arï-g*. In my view, pTk **sia:rï*- ‘to be white, yellow’ represents the original proto-Turkic form, rather than deriving it from pTk **sa:rï*-, as is proposed by them. Róna-Tas et al. (2011: 693) further point out that the meaning ‘yellow’ is probably secondary because it denotes a light yellow colour, which probably evolved from the word for ‘white’. The original meaning ‘white’ is preserved in Chuvash and in the Khazar place name.

153. SEW

pJ **nup*- ‘to sew, stitch’: J *nuu* B*,* OJ *nup*- ‘to sew, stitch, embroider’, Yoron (Amami) *nuuju*ɴ 'to sew', Shuri (Okinawa) *nooju*ɴ, Hirara (Miyako) *nuu* 'to sew', Igarashi (Yaeyama) *nooŋ* 'to sew', Yonaguni *nuŋ* 'to sew', pR **noCu*- 'to sew',
pK **nupi*- ‘to sew, quilt’: MK *nwu(·)pi*- ‘to quilt’, MK *nwu·pi* ‘quilting’
pTg **nup*- ‘to prick, pierce’: Evk. *lupa*- ‘to prick’, *lupu:*- ‘to go through, pierce’, Even *nụbas an*- ‘to prick’, Neg. *lepu*- ‘to pierce’, Na. *loqpa*- ‘to prick (intr.)’, Olch. *loqpa*- ‘to prick oneself’, *loqpụ(n)* ‘a splinter’, Orok *lụkka*- ~ *lụqpa*- ~ *lupqa*- ‘to prick oneself, to impale oneself upon smth; to prick smth’

The Tungusic verb stem is probably a compound of pTg **nup*- ‘to prick, pierce’ with a suffix *-*kA*-, perhaps the alternant of the inchoative suffix pTg *-*xA*- in voiceless

[9] Old Turkic distinguished between two sibilants in native words: alveolar /s/ and palatal /š/ (Erdal 2004: 82-83). The distinction is found in most runiform inscriptions of Orkhon Old Turkic. Manichean writing uses two different characters, but other Old Uighur texts do not distinguish consistently, as is the case here for Orkhon OTk. *šarïg* vs. Uighur OTk. *sarïg*.

clusters (see Robbeets 2015: 259.) Poppe (1960, 74) finds that the initial *l*- in the Tungusic languages is a secondary development from an original **n*-: “Das anlautende *l* im Mandschu-Tungusischen ist sekundärer Herkunft und geht gewöhnlich auf ein anlautendes **n* (meistens vor einem folgenden **m*) zurück.” This view is consistent with the general absence of initial liquid phonemes across the Transeurasian languages. The environment in which this development takes place needs further study, but it should probably be extended to the position before *-*PK*- clusters, e.g. pTg **nabga:n*- ‘to glue, stick’ in Evk. *labgan*-, Even *nabgan*-, Neg. *labga:n*-, Orok *lamba*-, Ud. *lagbamu*-; pTg **nobgi* ‘squirrel nest’: Evk. *lopi* (dial. loki:); Neg. *lo:bi̦*, Ulcha *logbu̦*, Na. *lo:bi̦*, Ud. *loi;* pTg **napki* ‘tiers, straps (for skis)’: Ulcha: *la:xi̦*, Orok *la:pu̦*, Na. *la:pi̦*, Oroch *lappi*; pTg **napku*- ‘to insert, hang: Evk. *lapku*-, Even *napkü*-. Note that Even consistently retains the initial nasal here. Semantically, the Tungusic cognate is a weak fit because the semantic association between 'to sew' and 'to pierce' does not appear in List's (2014) database.

pK **sili* 'thread' < ? pK **sil(i)*- 'to sew, tie together'? (pK *-*i* deverbal noun; Robbeets 2015: 459): K *sil*, MK ¨*sil* ‘thread’

pTg **sira*- 'to sew together, tie together': Evk. *sira*- ~ *hira*- ‘to piece down, to lengthen, to add (cloth, belt)’, Even *hi̦raq*- ‘to piece down, to lengthen, to add (a belt, a rope), to connect, to add’, *hi̦raqan* ‘phalanx of a finger’, Neg. *seya*- ~ *siya*- ‘to lengthen, continue a belt, a rope; connect two parts’; *siya:n* ~ *siyə:n* ‘thread; way (figurative)’; *siyəktə* ‘thread’; Orok *si̦ra*- ‘to lengthen, to piece down; to add a belt, a rope etc.; to connect together ends of a rope, a thread etc.’; Ma. *sira*- ‘to continue, follow; to connect, tie together; to inherit’, *sirame* ‘next (in sequence); step- (e.g. *sirame ama* ‘stepfather’; *siran* ‘continuation, succession, sequence, order’; Sibe *sira*- ‘to inherit; connect; to join; to continue’; Jur. *sir(a)-ru* 'inherit'; Olcha *si̦ra*- ‘to connect’, *si̦ra-ǯu̦*- ‘to add; to marry a widow of the elder’s brother; to inherit’, *si̦ra-ču̦-ǯu̦*- ‘to tie together’; Na. *sera*- ‘to sew together cords, threads; to continue telling a fairy-tale which was interrupted’, *sera-go*- ‘marry a widow of one’s elder brother; keep on fire of one’s clan; to tie together two cords’, *sera-kta*- ‘to tie together several cords, threads’, *sera-ča* ‘knot (to lengthen a cord, a thread); a cord, a thread (for lengthening)’; Oroch *siya*- 'to sew together, piece down'; Ud. *sǣ*-, *sǣ-si*- ‘to piece down, to lengthen (cloths), to sew together (climbing skins); to add (a rope, a belt)’; *sǣ* ‘cross joint of climbing skins (in piecing down)’

pMo **siri*- 'to sew, stitch, quilt': WMo. *siri*- '1 to quilt, stitch (tr.)', Khal. *šire*- 'to make firm by sewing, to quilt', Bur. *šere*- 'to stitch (usually the sole of a shoe)', Kalm. *šir*- '1', Ordos *šire*-, *širi*- '1', Dgx. *šïri*- '1', Dag. *širi*-, *šire*- '1', Eastern Yugur *širə*- '1', Mgr. *śiri*-, *śirə*- 'to cover a bed with a counterpane'

pTk **sïrï*- 'to sew, stitch, quilt': Karakhanid *sïrï*- 'to sew, to quilt or smock a garment', Tk. *sïrï*- 'to sew tightly, quilt', Az. *sïrï*- 'to quilt; to foist, impose', Tkm. *sïra*- 'to sew; to stitch; to quilt', Tat. *sïr*- 'to quilt', Kaz. *sïr*- 'to stitch', Nog. *sïrï*- 'to quilt', Bash. *hïr*- 'to quilt', KKalp. *sïrï*- 'to sew; to quilt', Kum. *sïrï*- 'to sew; to quilt; to fasten, attach', Uig. *širi*- 'to quilt', Khak. *sïrï*- 'to sew; to quilt', Tuva *sïrï*- 'to sew; to quilt'

Given the rising tone, MK ¨*sil* ‘thread’ can be derived from an original polysyllabic form, whereby the contraction was likely to be due to the equality of the vowel in both syllables, thus pK **sili* 'thread'. Given the reconstruction of a deverbal noun noun suffix pK *-*i* ~ *ø*, attested in e.g., MK *hal*- 'to slander' → MK *hali* 'slandering' and MK *nwu·pi*- 'to quilt' → *nwu·pi* 'quilting', it is possible that the Korean form originally derived from a verb pK **sil(i)*- 'to sew, tie together'. Note that the deverbal

derivation from 'to sew, tie together' as 'thread' is also attested in the Tungusic languages, e.g. Na. *sera-* ‘to sew together (cords, threads)' → *sera-ča* ‘cord, thread'.

161. MANY

pJ **opə-* 'to be many, be much': J *oo-*, OJ *opo-* (B) ‘to be many, much, copious, abundant; great, big', J *ookii*, OJ *opo$_2$ki-* (B) ‘big, large, great, grand’, Yoron (Amami) *uppuusa* 'many', *upisjaɴ* 'big', Shuri (Okinawa) *ʔuhooku* 'many', Irabu (Miyako) *upukam* 'big', Yonaguni *ubusaan* 'big'

pK **op-* 'enough', K *opwus* (*ha-*) ~ *opus* (*ha-*) ‘substantial, sufficient, moderately, wealthy’(K *-us* ~ *-s* suffix deriving impressionistic adverbs, e.g., K *cop* - ‘to be narrow’ → K *copus* (*ha-*) ‘narrow’; Martin 1992: 912)

162 TO CHEW

pMo **kebi-* 'to chew, ruminate': WMo. *kebi-* '1 to ruminate, chew the cud', MMo. *kebi-* '1', Khal. *xeve-* ~ *xive-* 'to chew; to ruminate, chew the cud', Bur. *xibe-* 'to chew, ruminate (only said about bovines)', Kalm. *kew-* '1', Ordos *kewe-* '1', Eastern Yugur *kewə-* '1', Mgr. *ke:yi-* '1'

pTk **ke:b-* 'to chew, ruminate': Karakhanid *kevše-* '1 to ruminate, chew the cud', Tk. *gev-* 'to try to gnaw or chew; to champ (the bit)', *geviš* 'rumination', Az. *gövšä-*'1', *göyüš* 'cud', Tkm. *gä:vüše-* 'to ruminate; to eat, have a snack', *gä:vüš* 'cud', Gag. *gevšen-* '1', Tat. *küšä-* 'to ruminate; to eat, have a snack', *kevšek* 'cud', Khak. *kipsen-* '1', Kirg. *küyšö-* 'to ruminate; 'to shiver (of the upper part of the body), to move (of shoulder blades); to husk (grain)', Kaz. *kŭyĭs* 'cud', Nog. *küyze-*'1', Bash. *köyšä-* 'to chew; to ruminate', *köyöš* 'cud', Karaim *kövšen-* 'to chew', KKalp. *güyse-* 'to ruminate; to say the same thing many times'; Salar *küšä-*'to chew', Kym. *güyše-*'1', Uz. *kawša-* 'to chew, ruminate', Uig. *köyši-* 'to ruminate', *kösändi* 'cud', Tuva *kegžen-* '1', *kegženig* 'cud', Tofa. *kegže-*'1', Shor *kepžen-* 'to eat, have a snack', Yak. *kebiy-* 'to ruminate; to gnaw', Khalaj *kä:viš* 'rumination, cud', Chu. *kavle-* ~ *kevle* ~ *kelle-* '1', *kavle*, *kavlek* 'rumination, cud'

164. WET

pJ **uru-* ‘to be wet’ (pJ **-pa-* ~ *-pə-* reflexive-anticausative; Robbeets 2015: 292-294): OJ *urup-* (?B) ‘to get muddy, be wet’, J *uruow-*, OJ *urupop-* (?B) ‘to get damp, get moist, receive profits, get enriched’, OJ *urum-* (?B) ‘to get wet, moist’

pK **uli-* ‘to soak’: K *wuli-*, MK *wuli-* ‘to steep, soak, bleach’

pTg **ula-* ‘to wet, soak’ (pTg **-p-* anticausative; Robbeets 2015: 296-298): Evk. *ula-* ‘to soak, to wet (legs); to become wet; to melt (about falling water snow)’, *ulap-* ‘to become wet’ (Evk. *-p-* anticausative; Robbeets 2015: 298); Even *u̯l-* ‘to soak, to wet’, *u̯lab-* ~ *u̯lap-* ‘to get wet’, Sol. *u̯lakku:* ‘wet’, Ma. *ulga-* ~ *ulha-* ‘to wet, dampen, dip in a liquid’, *ulgaku:* ‘inkwell, well for ink on an inkstone’, Jur. *ul(h)a-* ‘to wet, to dampen, to dip in liquid’, Orok *u̯la-* ‘to soak; to wet’, Na. *u̯larïko:* (dial.) 'wet', Ud. *ula-* ‘to soak, to wet; to set a net’, *ula-sa-* ‘to become wet (about dried fish); to be settled (about net)’

Although the semantic association between 'be wet' and 'to soak' is not given in List's (2014) database, the polysemy is attested in Even *ula-* ‘to soak, to wet (legs); to become wet'.

pJ **nura-* ‘to be(come) wet’: J *nure-*, OJ *nure-* (A) ‘to be(come) wet, damp; to come undone, loose’, Yoron (Amami) *nidituɴ* 'to be wet', Shuri (Okinawa) *ndir-* (A) 'to get wet', *ɴditooɴ* 'to be wet', Hatoma (Yaeyama) *zoori bee* 'to be wet'

pMo **nor-* 'to be(come) wet': WMo. *nor-* 'to become wet, soaked, drenched, damp, moist (intr.)', *norγa-* 'to wet, moisten, soak (tr.)', MMo. *nur-* 'to be wet, soak', *norma* 'moistened', Khal. *nor-* 'to get wet', Bur. *noro-* '1 to become wet, soaked', Kalm. *nor-* 'to become wet', *norγa:-* 'to make wet', *norγu:(n), noru:* 'wet', Ordos *nor-* '1', Dgx. *noro- ~ nuru- ~ noru-* '1', Dag. *noir-* '1', *noirga:-* 'to make wet', Mgr. *no:ri- ~ nori-* '1'

165. FOUR

pJ **yə* ‘four’ (pJ **-tu* substantivizer): J *yotu*, OJ *yo*$_2$ (1.1) ‘four’, Yamatohama (Amami) *yuucï* 'four', Shuri (Okinawa) *yuuci* 'four', Irabu (Miyako) *yuucï* 'four', Ishigaki (Yaeyama) *yuucï* 'four', Yonaguni *duuci* 'four'

pTg **dü-gin* ‘four’: Neg. *diγin ~ diyin* ‘four’, *diγi-ni: ~ diγ-ni:* ‘four persons’, *diγi-təl ~ dik-təl* ‘in fours’, *diγijə: ~ diγjə:* ‘four times’, *diγi:* ‘fourth’; Ma. *duin* ‘four’, *duiŋ-gə* ‘folded in four, in fours, fours times’, *duiŋ-gəri* ‘four times’, *dui-tə* ‘four each’; Sibe *duin* ‘four’, *dui-tə* ‘each four’, Na. *duĩ* ‘four’, *duyə-(či-)gu-* ‘to do smth. for a fourth time’, *duyə(-čiə)* ‘fourth’, *dui-kə̄n* ‘only four’, *dui-ltə* ‘four days’, *dui-ltə:* ‘during four days’, *dui:-dui:* ‘in four (distr.)’, *dui:ni* ‘four persons’, *duirsu-* ‘to quadruple; to fold four times’; Olcha *dui(n)* ‘four’, *duiy* ‘fourth’, *dui-ltə* ‘four days’, *dui-ńi* ‘four persons'; Orok *ji:n* ‘four’, *ji:n-do ~ ji:n-ni:* ‘four persons’, *ji:-ltə* ‘four times; four days’, *ji:yə* ‘fourth’; Ud. *di:* ‘four’; Oroch *di:(n)* ‘four’, *di:-wæ* ‘fourth’, *di:-mdə* ‘four times’, *di:-ntə ~ di:-ńji* ‘in fours’, *di:-ńæ* ‘four persons’, Jur. *du'in* ‘four’, Evk. *dygin* ‘four’, Even *diγən* ‘four’, *diγi* ‘fourth’ and Sol. *digi* ‘four’.

pMo **dö-* 'four' (pMo **-rbAn* suffix in lower numerals, e.g., **gu-rban* 'three', **ha-rban* 'ten'; *-cin* suffix in decimal numeral units, e.g., **gu-cin* 'thirty'): WMo *dörbe(n)* '1 four', *döci(n)* '2 forty', MMo. *dorben*, *dorbä:n*, *durbän* '1', *docin*, *ducin*, *ducin* '2', Khal. *döröv* '1', *döc(in)* '2', Bur. *dürbe(n)* '1', *düše(n)* '2', Kalm. *dörwn̥* '1', *döcn̥* '2', Ordos *dörwö* '1', *döci* '2', Dgx. *ǯieruan*, *ǯieron* '1', Bao. *deroŋ*, *deraŋ* '1', Dag. *durube(n), durub*, *durəb* '1', *duci(n), duc*, *duci* '2', Eastern Yugur *dörwən*, *dörwen* '1', *döǯin* '2', Mgr. *de:ran*, *de:ren* '1', *te:ǯin*, *teǯin* '2', Mog. *dürbo:n*, *dorbɔ:n* '1'

I do not exclude that the Koreanic and Turkic reconstructions proposed for 'four', i.e. pK **yə* and pTg **tö:rt* are ultimately related. However, as the vowel do not correspond regularly in Korean, while the initial consonant does not correspond regularly in Turkic, I do not include these forms in the etymology. The Korean form is reconstructed on the basis of MK *ye·tulp* 'eight', whereby Whitman (2012) analyzes this form as **yə-tuWul* (four-two), i.e. four times two wheras Ratte (2016: 447) explains it as pK **yə-tə-tɨWɨ* 'four-COPULA-ten'. As the exact nature of the second element remains unclear, the reconstruction is rather speculative.

The reconstruction of pMo **dö-* 'four' is further supported by pMo **dö-nen* 'four-year-old'.

The reconstruction of pTk **tö:rt* 'four' is based on OT *tört* '1 four', Karakhanid *tört* '1', Tk. *dört* '1', Az. *dörd* '1', Tkm. *dö:rt* '1', Gag. *dört* '1', Tat. *dürt* '1', Kirg. *tört* '1', Kaz. *tört* '1', Nog. *dört* '1', Bash. *dürt* '1', Balk. *tört* '1', Karaim *dört* '1', Kkpak *tört* '1', Kum. *dört* '1', Uz. *tọrt* '1', Uig. *tö(r)t* '1', Tuva *dört* ‘1', Tofa. *dört* ‘1', S-Yug. *dürt*, *türt* '1', Khak. *tört* '1', Shor *tört* ‘1', Yak. *tüört* ‘1', Dolg. *tüört* ‘1', Chu. *tăvat* ‘1’.

166. SOFT

pJ **mɨrɨ-* ‘to be weak, fragile’: J *moro-* (B), OJ *moro*$_2$*-*‘to be brittle, fragile’

pK **mɨlɨl-* ‘to be(come) soft, mellow’: K *mwulu-*, MK *mulu-* ‘to soften, become tender’, K *mwulleng ~ mollang ha-* ‘to be soft, tender’

pJ **yapa- ~ *yopa-* 'to be soft, weak': J *yawaraka-* ‘to be soft, tender, smooth’, Mod. J *yafa-* ‘to be soft’, OJ *yapa-* ‘to be hungry’, J *yowa-* ‘to be weak’, OJ *yo*$_1$*wa-* (B) 'to be weak', Yamatohama (Amami) *yawarasari* 'soft', Shuri (Okinawa) *yahwarasaɴ* 'soft', Irabu (Miyako) *yapaham*, *yaaraham* 'soft', Ishigaki (Yaeyama) *yaɸarasaaɴ* 'soft'

pK **yepuy-* 'to become weak' < ? **yepu-* 'to be weak' (pK **-i-* fientive; Robbeets 2015: 320): K *yewui-*, MK *ye·wuy-* 'to become thin, emaciated, haggard, to grow gaunt; to get impoverished', North Kyengsang *yebinda*, South Hamkyeng *yəbinda*, South Cenla *yabunda* 'become thin, weak' (Vovin 2010: 221)

pTk **yaba-* 'to be(come) soft, mild, quiet' (OT -*(X)š* deverbal property noun/adjective, e.g. *arï-* 'to be clean, pure' -> *arï:š* 'clear, pure'; Erdal 1991: 262-275): OT *yaval-* '1 to be(come) tamed, calmed, quietened', Karakhanid *yaval-* '1', *yava* (*yer*) 'warm, cosy (place)', *yavaš* 'mild, meek; gentle'; Tk. *yavaš* 'quiet, calm, even-tempered; cautious; slow ', Az. *yavaš* 'quiet; cautious; slow', Tkm. *yuvaš* 'gentle, mild, quiet; calm, even-tempered', Gag. *yavaš* 'mild; quiet; slow', Tat. *yuwaš*, *yuaš* 'mild; gentle; calm; modest; submissive; Khak. *čabas* 'mild; gentle; quiet', Kirg. *ǰo:š* 'mild; gentle', Kaz. *žuwas* 'mild; quiet; submissive', Nog. *yuwas* 'mild; gentle; quiet; calm', Bash. *yïwaš* 'mild; gentle; quiet; submissive', Balk. *ǰuwaš* 'mild, quiet, submissive', Kum. *yuvaš* ''mild; gentle; quiet; calm, harmless'; *yavaš* 'quiet, calm', Uz. *yuwɔš* 'mild; quiet; submissive', Uig. *žuvaš (~ žuɣaš ~ yavaš ~ yuvaš ~ yuɣaš)* 'mild; quiet; slow', Tuva *ča:š* 'mild; quiet; submissive'; Shor *čobaš-* 'to be(come) tamed, calmed, quietened', *čobaš* 'mild; quiet; submissive'

The Japanese adjective *yawaraka-* ‘soft, tender, smooth’is a derivation from the verbal adjective stem underlying OJ *yapa-* ‘to be hungry’and Edo mod. J *yafa-* ‘to be soft’. The original root pJ **yapa-* probably stood in expressive vowel alternation with pJ **yopa-*, reflected in J *yowai* ‘weak’. According to List et al. (2014), the meanings 'soft' and 'weak' are commonly combined on the same lexeme across the languages of the world.

In spite of Vovin's (2010: 221-222) remark that there are "no good Koreo-Japonic etymologies reflecting pJK **y-*, creating another gap that strongly speaks against a genetic relationship", the present sound correspondence probably does not reflect a consonantal but a vocalic correspondence, going back to an original diphtong pTEA **ia*. In this basic vocabulary list, it occurs word-initially in 101 MOUNTAIN and 194 OPEN and word-medially in 12 BREAST, 152 WHITE and 178 WOMAN.

Given the frequency of the deverbal noun and adjective suffix -*(X)š* in Turkic in addition to the attestation of verbal forms such as OT *yaval-* '1 to be(come) tamed, calmed, quietened' and underived property nouns such as Karakhanid *yava* 'warm, cosy', it is legitimate to derive the Turkic forms from an original verbal adjective pTk **yaba-* 'to be(come) soft, mild, quiet'. The common denominator of the Turkic meanings is 'soft' or 'weak' in the metaphoric sense.

167. LOOK

pTg **kara-* 'to look, watch, observe' (pTg **-mA-* optative; Benzing 1955: 121; **-ku* deverbal instrumental noun; Robbeets 2015: 471; pTg **-n* deverbal noun; Robbeets 2015: 386): Evk. *karaw-* ‘to guard; to watch out for (an animal)’ (Evk. -*w-* anticausative; Nedjalkov 1997: 228), *karama-*, *karma-* 'to guard, protect'; Even *qaran- ~ qaraŋčị-* ‘to stare’ (Even -*(A)n-* deverbal processive for spontaneous action;

Robbeets 2015: 232-233) , *qarqị-* ‘to wait’, Sibe *karma-* ‘to protect; to defend; to bless’, *karman* ‘protection; blessing’; Ma. *kara-* ‘to look down from a height, to gaze into the distance’, *karan* ‘a lookout tower, watchtower, platform’, *karaba* ‘mutual protection’, *karma-* ‘to protect, to take care of’, *karman*, *karmačun* ‘protection’, *karmaku:* ‘protector’, *karmasi* ‘protector, patron’, Olcha *qargačụ-* ‘to watch out for; to hunt a large animal’, *qargakụ* ‘a hunting watchtower’; Na. *qargači-* ‘to watch over smth., from a high place or rising on toes; to look between one’s legs, bowing (about children)’; Orok *qarɢa-* ‘to observe; to look from a high place; to observe a place’, *qarɢaytụ* ‘observation post’, *qarɢaqụ* ‘observation tower; binocular’
pMo **kara-* 'to look, watch': WMo. *qara-*, *qaraɣul* [check], MMo. *qara-* '1 to look', *qara'ul* '2 patrol, watch', Khal. *xara-* 'to look, look at, face; to look after, care for; to look out for', *xaru:l* 'watch-post; guard, watch'; Bur. *xara-* '1'; Kalm. *xarə-* 'look out over, gaze; have nothing to do, hang around', Ordos *xara-* '1', Dag. *xara:la-* '1', *xaro:l* '2', Eastern Yugur *χaru:l* '2', *χarəmul* 'sight', Mgr. *xaru:la-*, *xarə-* '1', *xarəmul* 'sight', Mog. *qara-* '1'

Since the verb root pTg **kara-* is well distributed across the Tungusic languages and since most of the derivations are morphologically transparent, consisting of the verb root and an attested deverbal suffix, I do not follow the suggestion made by Doerfer 1985 (51, 138) and Rozycki (1994: 135) that all Tungusic forms are borrowed from Mongolic. Nevertheless, this does not exclude that some of the morphologically unanalyzable forms in Tungusic such as Ma. *karun* 'outpost sentry, border, guard' may be nevertheless borrowed from Mongolic.

169. THAT
pJ **ə-* 'that' (demonstrative pronoun of the mesial plan): J *ore* ‘I (first person pronoun), OJ *ore* 'self; you derogatory 2sg', OJ *ono₂* ‘self’; Shuri (Okinawa) *ʔu-ri* 'that person, thing', *ʔu-ttaa* 'they', *ʔm-ma* 'there', *ʔu-nu* 'that' (mesial demonstratives), Amami *u-rɨ* 'that', *u-ma* 'there' (mesial demonstratives), Hateruma (Yaeyama) *u-ri* (distal demonstrative), Yonaguni *ú* 'that', *ú-ma* 'there' (mesial demonstrative)
pTg **e-* 'this' (demonstrative pronoun of the proximal plan): Even *ere* 'this, here', *ecin* 'in this way, indeed', *eduk* 'since this time, from here', *ele:* 'here', *eweski*: 'in this direction', *ewgi:le:* 'here', *ewgi:n* 'here', Evk. *er*, *eri* 'this', *ewgi:* 'in this direction', *ele:* 'here', *eduk* 'since this time, from here', Solon *eye: ~ er ~ eri* 'this', *elur* 'these', Neg. *ele: ~ eli:* 'in this direction, here', *ewuli*: *~ ewule:* 'here', *ewgi:le: ~ ewgi:li:* 'in this direction', *edu:* 'here, at this time', *eye* 'this', Ud. *ebede* 'like this', *eyi* 'this', Olcha *yedu* 'here', *yeji* 'with this', *yeki* 'here', *yele* 'from here', *ei* 'this', *e:wu* 'this', Na. *eyebe ~ yebe* 'here', *ei* 'this', Orok *edu ~ eyedu* 'here', *ewwe*, *eye*, *er*, *eri* 'this', *emete(n)* 'such, like this', Ma. *ere* 'this', *embici* 'or' (*-bi-ci* be-COND.CONV), *eici* 'or', Sibe *erə* 'this', Jur. *e(r)se* 'this'
pMo **e-* 'this' (demonstrative pronoun of the proximal plan) in pMo **e-ne* (this-SG) 'this': MMo. *ene* (SH/HY/Muq), WMo. *ene*, Khal. *ene*, Bur. *ene*, Ordos *ene*, Kalm. *en*, Dag. *ənə*, Eastern Yugur. *ene*, Mgr. *ne*, *ni*, Bao. *enə, nə,* Dong. *ənə,* Mog. *enȧ*; in pMo **e-de* (this-PL) 'these': MMo. *ede* (SH/HY), WMo. *ede(n),* Khal. *ed(en),* Bur. *ede*, Kalm. *edn*, Dag. *əd(ən)*; in pMo **e-n-de* (this-SG-LOC) 'here': MMo. *ende* (SH/HY/Muq), WMo. *ende*, Khal. *end*, Bur. *ende*, Kalm. *end*, Dag. *ənd*, Eastern Yugur. *ende*, Mgr. *nde*, Bao. *endə,* Dong. *əndə*, Mog. *endȧ*

pJ **a-* 'that' (demonstrative pronoun of the distal plan): J *a-*, OJ *a-* (1.2) 'that (distal)' in J *ano*, *are* 'that one', *asoko*, J *atira* 'over there', Amami *a-* 'that (distal)' in *a-rï* 'that

one', *a-ma* 'over there', Shuri (Okinawa) *ʔa-* 'that (distal)' *ʔa-ri* 'that one', *ʔa-ttaa* 'they', *ʔa-ma* 'there', *ʔa-nu* 'that', Kuroshima (Yaeyama) *a-ma* 'over there'

pTk **a-n-* 'that' (demonstrative pronoun of the distal plan in the oblique cases) (pTk *-*n-* oblique suffix in pronouns): OT *a-n-ïŋ* 'that (GEN)', *a-ŋa* 'that (DAT)', *a-n-ï* 'that (ACC)', *a-n-ta* 'that (LOC)', *a-n-tïn* 'that (ABL)', *a-ŋaru* 'that (DIR)'; Tkm. ana 'here, look! (particle)'; Tat. *a-n-da* 'that (LOC)', *a-ŋa* 'that (DAT)', *a-n-ar* 'that (DIR)'; S-Yug. *a* 'that (NOM)'; Khak. *a-n-da* 'that (LOC)', *a-ɣaa* 'that (DAT)'; Kirg. *a-n-ta* 'that (LOC)', *a-(ɣ)a* 'that (DAT)', Bash. *a-n-ta*, *a-n-da* 'that (LOC)', *aŋ-a* 'that (DAT)'; Balk. *a-n-da* 'that (LOC)', *aŋ-ɣa* 'that (DAT)'; Karaim *a-n-da* 'that (LOC)', *a-n-ar* 'that (DAT)'; Salar *a-n-dï* 'that (LOC)', *aŋ-a* 'that (DAT)'; Tuva *a(ŋ)-a* 'that (DAT)'; Tofa. *a(ŋ)-a* 'that (DAT), Shor *a-n-da* 'that (LOC)', *a-(ɣ)a* 'that (DAT)', Yak. *anara*: 'here, look! (particle)', Khalaj *ǟ-rä* 'that side', Chu. *ă-n-a* 'that (DAT)'

In most Turkic languages, except Sary-Yughur, the root of the distal demonstrative *a-* is restricted to the oblique cases and marked with the oblique nasal suffix *-n-*. It acts as an oblique counterpart for the distal demonstrative *ol* 'that'. Although Old Turkic displays a two-way distinction in its demonstratives, i.e. OT *bo* / *bun-* ‘this’ vs. *ol* / *an-* ‘that’, many contemporary Turkic languages such as Turkish make a three-way distinction, e.g., Tk. *bu* ‘this’, *şu* ‘that’, *o* ‘that (over there)’. Demonstrative pronouns in earlier and contemporary varieties of Mongolic and Tungusic exhibit a proximal-distal distinction, e.g., MMo. *ene* ‘this’ vs. *tere* ‘that’, Khal. *e-* ‘this’ vs. *te-* ‘that’, Ma. *ere* ‘this’ vs. *tere* ‘that’ and Evk. *er(i)* ‘this’ vs. *tar(i)* ‘that’. Demonstrative pronouns in Contemporary and Middle Korean, however, show a proximal-mesial-distal opposition: K *i* ‘this’, *ku* ‘that’, *ce* ‘that over there’ and MK *i* ‘this’, *ku* ‘that’, *tye* ‘that over there’. This is also true for Contemporary Japanese: J *ko-* ‘this’, *so-* ‘that’, *a-* ‘that over there’. In contrast to most accounts of Old Japanese demonstratives, which posit a three-way contrast between OJ *ko*$_2$ ‘this’, *so*$_2$ ‘that’ and *ka* ‘that over there’, Frellesvig (2010: 139-142) and Frellesvig & Whitman (2008: 27-29) argued that the disal demonstrative OJ *ka* was not a productive member of the demonstrative system, but originally just a vowel-harmonic alternant of the proximal demonstrative OJ *ko*$_2$ and that pre-Old Japanese had a simple proximal-distal distinction; see 39 THIS. Similarly the distal pronouns discussed here, pJ **a-* and pJ **ə-* 'that' may originally have been vowel-harmonic variants. Even if most Ryukyuan languages have a proximal-mesial-distal opposition, Yaeyama is limited to a proximal *kuri* vs. distal *uri* (Aso 2016: 429). This supports the claim that proto-Japonic originally had a two-way distinction as elsewhere in Transeurasian. Proto-Japonic may have shifted to a three-way opposition under substratum influence of pre-Ainu, where each demonstrative pronoun denotes a different degree of proximity: *ta an* ‘this’ (distal), *ne an* ‘that’ (mesial), *to an okai* ‘that over there’ (distal).

170. TO CUT

pJ **kira-* ‘to cut’: J *kiru* B*,* OJ *ki*$_1$*r-* ‘to cut’, OJ *ki*$_1$*ras-* B ‘to run/ sell out of’, J *kireru* B*,* OJ *ki*$_1$*re-* ‘to be sharp, get cut, run out’, Yamatohama (Amami) *kiruri* 'to cut', Shuri (Okinawa) *cijuɴ* 'to cut', Irabu (Miyako) *cïï* 'to cut', Ishigaki (Yaeyama) *kïsuɴ* 'to cut', Yonaguni *ccùn* 'to cut', pR **kiri-* 'to cut' (Thorpe 1983: 276)

pTg **giri-* ‘to cut out’: Evk. *gir-* ~ *ger-* ~ *kir-* ‘to cut out (with scissors)’, *giri-ptun* ‘cloth piece’, Even *gịr-* ‘to cut out’, Neg. *gey-* ~ *giy-* ‘to cut out’, Ma. *giri-* ‘to trim with a knife or scissors, to cut evenly, to cut a strip’; *giri-n* ‘strip; section, area’; *gir-dan* ‘cloth or strips of pelts cut with scissors; evenly cut slices of meat; pennant;

border trim on a banner'; *giriku*: 'a small knife for trimming skin, paper, and cloth', Olcha *gịrị-* 'to cut out', *gịrsụ* 'woman's knife for cutting things out', Orok *gịrị-* 'to cut out', *gịrị-ptụla* 'cloth pieces, remnants', Na. *gere-* 'to cut out with a knife', *gerego-* 'to recut out, to cut out anew', *gerekta-* 'to cut out many objects', *gerekto* 'cut out', *geremsa*, *gereptola* 'cloth rags', *gereče-* ~ *gerenase-* 'to cut out many times', *gerso:* 'woman's knife for cutting objects out', Oroch *gi:-* ~ *giyi-* 'to cut out with a knife', Ud. *gi:-* ~ *gi:na-* 'to cut out with scissors'

pTk **kïr-* 'to cut, scrape': Karakh. *kïr-* 'to scrape, strip (hair), pluck out (hair), to cut off', Tk. *kïr-* 'to break, split (wood); to rough-grind, crush (grain); to hurt, injure', Az. *ġïr-* 'to break; cut', Tkm. *ġïr-*'to scrape', Gag. *ḳïr-* 'to break, crack', Tat. *ḳïr-* 'to break; to scrape; to shave; to grate; to whet; chafe', Khak. *xïr-* 'to cut; to scrape; to shave; to chafe', Kirg. *kïr-* 'to scrape; to shave', Kaz. *kïr-* 'to scrape; to shave; to chafe', Nog. *kïr-* 'to scrape; to shave', Bash. *kïr-*''to scrape; to shave; to grate; to chafe; to whet', Karaim *kïr-* 'to scrape; to shave', Kkalp. *kïr-* 'to scrape; to shave', Uz. *kir-* 'to scrape; to shave; to break', Uig. *ki(r)-* 'to scrape; to shave; to break', Yak. *kïrïy-* 'to cut', *kïra* 'small', Dolgan *kïrïy-, kïrpala:-* 'to cut', Tuva *kïr-* 'to break; to exterminate all without exception', Khalaj *kïr-* 'to break', Chu. *xïr-* 'to scrape'

The Mongolic forms WMo. *kira-*, *kiru-*, Khal. *x΄ar-* and Kalm. *kur-* that support the reconstruction of pMo **kira-* 'to cut into small pieces, mince' have been omitted since they can be assumed to have been copied from Turkic. If they were cognates, we would expect an initial voiced velar (**g*-) in Mongolic (Robbeets 2004b: 170-172).

The Chuvash verb *xïr-* 'to scrape' has an irregular vowel correspondence and may thus be due to borrowing.

171. MOTHER

pJ **əmə* 'mother': OJ *omo* (2.4?), EOJ *amo* 'mother'

pK **ema* 'mother': K *enni* 'older sister', K *emeni* 'mother' (dialect *emennim*, *emni*, *emi* 'mother'), MK *·e·ma¨nim* > *·e·mi*, K *emma* 'mommie', MK *·e·ma* 'mother!'

pTg **eme* 'mother (in law)': Sibe *əmhə* 'mother-in-law', *əmilə* 'female', Ma. *əmə* 'mother', *əməkə* 'husband's mother, mother-in-law', *əmhə* 'wife's mother, mother-in-law (in some early texts it may also refer to a husband's mother)', *əmilə* 'the female of birds', Jur. *emuhe* 'mother in law', Ud. *əmə* 'mother in law (mother of the wife)'

pMo **eme* 'woman', **eme-gen* 'elderly woman, grandmother' (pMo *-*gAn* diminutive, e.g. *beri-gen* 'daughter in law', **küri-gen* 'brother in law', *kurï-gan* 'lamb', **bota-gan* 'camel foal', etc.): WMo. *eme* '1 woman, wife; female (n. and adj.)', *emege* 'grand-mother, grand- (adj.)', MMo. *eme* '1', *emege* ~ *emegen* '2 elderly woman, grandmother', Khal. *em* '1', *emgen* '2', Bur. *eme* 'woman; female', *emgen* '2', Kalm. *emə* '1', *emgn* 'elderly woman, wife, woman', Dag. *emgun* '1' (< **eme-küün* female-person), *emeg* '2', Eastern Yugur *eme* 'female', *emgen* '2', Mgr. *imu* 'girl (term of endearment)', *mugзn* '2', Bao. *emə* 'wife', Kgj. *eme* '1', Dgx. *əmə* 'woman, wife, female'

pTk **eme* 'mother; old woman': Tk. (dial.) *eme* 'old woman', Az. (dial.) *ämä* 'old woman', Chu. *ama* 'mother; 'female; queen bee; a mother goddess; part of plough; a part of gates'

The forms mentioned here form no exception to the general observation that formations for 'mother' or 'father' across the world are commonly nursery terms. For

this reason, the etymology was removed from the core evidence in Robbeets (2005). However, as the term of 'mother' is part of the basic vocabulary list, I mention it here. Besides, given the regular sound correspondences, it cannot be excluded that the nursery formation took already place in the ancestral Transeurasian language.

176. WARM

pJ **nuku-* ‘to be warm’: J *nuku-*, Mod. J *nuku-* (B) ‘to be warm, mild, genial’, dial. *noko-* ‘to be warm’, Yoron (Amami) *nukusaɴ* 'warm’, Shuri (Okinawa) *nukusaɴ* 'warm’, Irabu (Miyako) *nufukam* 'warm’, Ishigaki (Yaeyama) *nuɸusaaɴ* 'warm’, Yonaguni *nûkucan* 'warm’, pR **nuku-* ‘to be warm’ (Thorpe 1983: 345)
pK **nuk-* ~ **nok-* ‘to be(come) warm, mild, soft’: K *nwuk-*, MK *nwuk-* ‘to be damp, moist, tender, soft, to become mild, become warm’, MK *nwok-* ‘to melt, dissolve, become warm’

The alternation between pK **nwuk-* and **nwok-* is probably expressive in origin. In Korean, relatively retracted and non-retracted vowels tend to alternate phonologically in certain color adjectives, mimetic and expressive adjectives, e.g. K *ce:k-* ‘to be small in number or quantity, few’ vs. K *ca:k-* ‘to be small in size, tiny’ or K *nwuleh-* vs. *nolah-* 'to be yellow'. A similar phenomenon may be at the origin of the vowel alternation in standard Japanese *nuku-* and dialectal *noko-* ‘to be warm’.

177. TO COVER

pMo **bürü-* 'to cover' (pMo **-yi-* anticausative, e.g., pMo **kaja-* ‘to bend (tr.)' → WMo. *qajayi-* ‘to be crooked (intr.)’; Robbeets 2015: 263): WMo *bürüg* 'dark, dusky, overcast; timid', *bürüyi* 'twilight, dusk; obscure, dark', *bürülje-* 'to become clouded or dimmed (of eyes etc.)' (WMo. *-ljA-* distributive suffix in rythmic motion), MMo. *buri-* '1 to cover', Khal. *büre-* 'to cover, to face, to line', *büriy* '2 dusk', Bur. *büri-* '1', *bürü:l*, *bürü:r* '2', Kalm. *bür-* 'to wrap sth. in sth, to cover, to supply a blanket, lid or roof (tr.)', *büri-* 'to be dark, to be overcast, to dawn (intr.)', Ordos *büri-* '1', Dag. *buri:* '2', *burgie:n* '2', Eastern Yugur *burʁoloG* '2', Mgr. *burə-*, *buri-* '1'
pTk **bürü-* 'to cover' (pTk **-(X)n-* processive suffix, e.g. OT *to-* 'to close' → *ton-* 'to be closed'; Robbeets 2015: 237-239): Karakhanide *bürün-* 'to be covered, wrapped', Tk. *bürü-* 'to cover up, fill (of smoke in a room); to wrap, enfold, envelop', Az. *bürü-* 'to cover; to wrap; to close; to surround; to fill', Tkm. *büre-* 'to cover', Gag. *bürü-* 'to cover', Dolg. *bürüy-* 'to cover', Yak. *bürüy-* 'to cover'

In Turkic, the majority of forms reflects a common shape *bürke-: Bash. *börkä-* 'to close (tr.)', KKalp. *bürke-* 'to cover; to close; to be misty, grey, cloudy (of a day, weather)', Kaz. *bürke-*'to cover; to wrap; to mask, disguise; to hide', Khak. *pürge-* 'to cover; to wrap', Kirg. *bürkö-* 'to cover', Kum. *bürke-* 'to cover; to envelop, shroud', Nog. *bürke-* 'to cover', Tat. *börkä-* 'to close', Shor *pürge-*'to cover; to wrap', Altay *bürke-*'to cover; to close; to wrap (tr.)', Tuvan *bürge-* 'to surround; to be misty, grey, cloudy (of a day, weather)' and Chu. *pə^{w}rke-* 'to cover; to dress'. Since it is not possible to identify a deverbal verb suffix of the shape **-kA* in Turkic, these verbs are probably borrowed from a Mongolic model, i.e. pMo **bürke-* 'to cover, become cloudy' < **bürü-kü e-* (cover-NMLZ-VBLZ) reflected in WMo. *bürke-*, Khal. *bürxe-* and Bur. *bürxe-* 'to cover, become cloudy' (Nugteren 2011: 59, 294-295).

178. WOMAN

pJ **me* 'woman, female': OJ *omina* 'old woman, woman', OJ *womina* 'young woman' (OJ *o*- 'aged', OJ *wo*- 'young', OJ *na* ‘name, person’), J *onna* (3.2b) 'woman', J *me* (1.3b), OJ *me*$_1$ 'female', Yamatohama (Amami) *onagu* 'woman', *miï̈* 'female', Yonamine (Okinawa) *inaaguu* 'woman', *miimuɴ* 'female', Irabu (Miyako) *midum* 'woman', *mii-munu* 'female', Ishigaki (Yaeyama) *miiduɴ* 'woman', *mii-munu* 'female', Yonaguni *mînuŋa* 'woman', *mii-munu* 'female'
pK **mye* 'woman': K *myanuri* ‘daughter in law, son’s wife’, MK *mye·nol*, *mye·noli* 'son's wife'

Whitman and Frellesvig (2008) argued in favor of reconstructing a seven vowel system in proto-Japonic, adding two mid vowels (**e*, **o*) to the traditionally reconstructed system. They proposed that **e* raised in similar environments as **o* in proto-Japonic, merging with **i* in all positions except word-finally, where it became OJ *e*$_2$. The reconstruction pJ **me* 'woman, female' yields a good example of the alternation between the fully raised word-nonfinal OJ *i* in OJ *o-mi-na* 'old woman, woman' and the partially raised word-final OJ *e*$_2$ in OJ *me*$_1$ 'female'.

Even if the second part of the Korean word remains unexplained, the comparison with pK **mye* 'woman' seems to point to an original diphtong pTEA **ia*. In our basic vocabulary list, this correspondenceoccurs word-initially in 101 MOUNTAIN, 136 WHICH, 166 SOFT and 194 OPEN and word-medially in 12 BREAST and 152 WHITE.

179 DEEP

pJ **kipa*- ‘to be characterized by a rapid decline': J *kiwa*, OJ *ki*$_1$*pa* (2.3) ‘side, edge, margin, brink', OJ *kipam*- ‘to take to outermost limit’, J *kewasii*, OJ *kepasi*- (B) ‘steep, precipitous’
pK **kip-kʌ*- ‘to be deep’ (pK**-kɨ*- ~*-kʌ*- inchoative, e.g. MK *·mul* 'water' → MK *mwulk*- 'be watery'; Robbeets 2015: 257-258): MK *kiph*- ‘to be deep’, K *kiphi*, MK *ki·phuy* ~ *ki·phuy* ‘depth’

The verb MK *kiph*-‘to be deep’ goes back to an original thematic stem pK **kipkʌ*-, with the vowel reflected in MK *ki·phuy* ~ *ki·phuy* ‘depth’, a derivation with the deverbal noun suffix pK **-i* (Robbeets 2015: 459).

pJ **puka*-‘to be deep’: J *huka*-, OJ *puka*- (B) ‘to be deep, thick, dense', OJ *pukame*$_2$- (B) ‘to make deep (tr.)’, *puke*$_2$- ‘to deepen (tr.)’, Yamatohama (Amami) *hukasari* 'deep', Irabu (Miyako) *fukaham* 'deep', Hatoma (Yaeyama) *hukaa*ɴ 'deep', Yonaguni *kkân* 'deep'
pK **pokʌ*- ~ *pukɨ*- 'to be deep' → **pokʌ-k* ~ *pukɨ-k* 'deeply' (pK **-(ɨ/ʌ)k* adverbializer, e.g. MK *kuž*- 'to draw a line' → *kužuk* 'secluded, private'): K *phok* ~ *phuk* 'deeply, completely; falling down, sinking in (adv.)', MK *phok* 'deeply', MK *·pwok* ‘deep inside, mist’ is a bound noun in MK *·poy-s-·pwok* , K *pay-kkop* ‘navel, belly button’(MK *poy* ‘belly’) and in K *pwok-phan* ‘the very midst’

180. ABOVE

pJ **u-pa-(C)i* 'above' (pJ **pa* 'place') : J *ue* (2.2.a), OJ *upe*$_2$ 'above, upper part', Yoron (Amami) *ui* 'above', Shuri (Okinawa) *ʔwii* 'above', Irabu (Miyako) *waara* 'above', ishigaki (Yaeyama) *ui* 'above', Yonaguni *ui* 'above'

pK **u-k* 'above' (pK *-*(ɨ/ʌ)k* place suffix): K *wi*, MK *wuh* 'above, upside, upper part',

In Korean there is an exceptional class of non-rising low nouns of one syllable which may end in a voiced or voiceless consonant. Martin (1996: 45) regards these nouns as reductions from 2 syllables with a minimal vowel MK *u/o* in the second, so that a high accent may be imputed for the second syllable. A good number of these words seem to be compounds with the place suffix pK *-*(ɨ/ʌ)k,* e.g. MK *·stah* 'ground', MK *pas ~ pask* 'outside' (< **pasʌ-k*), *math* 'yard' (< **matʌ-k*), *alph* 'front' (< **alpʌ-k*), MK *path* '(dry) field' (< **patʌ-k*), MK *muth* 'dry land' (< **mutʌ-k* 'dry land'), etc.

183. OTHER

pJ **pəka* 'other' < **pəka*- 'to come beside, outside': J *hoka*, OJ *poka* (2.4.) 'other, another, different', Asama (Amami) *hukaa* 'other', Shuri (Okinawa) *huka* 'outdoors', Irabu (Miyako) *puka* 'outdoors', Ishigaki (Yaeyama) *huka* 'outdoors', Yonaguni *hùga* 'other'

pK **pekɨ*- 'to come next, follow': K *pekum* 'second, next', MK *pekwum* 'next, following', MK *peke* 'next', MK *pe· kun* 'being second (attributive)'

Given a parallel formation in Korean, whereby MK *pek-e* 'next' can be derived from an original verb pK **pekɨ*- 'to come next, follow', the Japonic reconstruction pJ **pəka* 'other' can probably be analyzed as a deverbal noun on the basis of the nominalizing suffix pJ *-*a* hypothesized by Sakakura (1966: 286–303); see also Robbeets 2015: 156.

186. TO RISE

pJ **ɨkɨ*- 'to rise, raise': OJ *oki*$_2$- (B) 'arise', *okoru* (B?), OJ *oko*$_2$*r*- 'to arise', *okosu* (B), OJ *oko*$_2$*s*- 'to raise', Shodon (Amami) *ʔwïhïïjum* 'to rise', Shuri (Okinawa) *ukijuɴ* 'to rise', Irabu (Miyako) *ukii* 'to rise', Ishigaki (Yaeyama) *ukiruɴ* 'to rise', Yonaguni *ugiruɴ* 'to rise', pR **oke*- 'to rise' (Thorpe 1983: 321)

pTg **ög(ö)*- 'to rise, go up, mount', **ögö-i*: 'above' (pTg *-*i:* deverbal noun suffix, e.g., Even *das*- 'to cover' → *dasi*: 'a cover'; Robbeets 2015: 461-462): Evk. *uw*- ~ *ug*- 'to mount (a horse etc.)', *ugi:*- 'above, up (postposition)'; Even *oy*- 'above (postposition)', *oyər*- 'to raise', *u:*- 'to mount'; Solon *ụgụ*- 'to mount a horse', *uge-sxi*: 'up'; Neg. *uyi*- ~ *uyi*- 'to raise', *uyi-də:* 'upper side, space above', *uyi* 'up, above (postposition)', Ma. *wəsi*- 'to rise, go up; to advance (in rank)'; *wəsi-hun* 'upward, up; eastward, east; honorable, revered, respected; your (honorific)'; Sibe *wəsi*- 'to rise; to flourish; to increase in price'; Olcha *u:wu* 'upper', *uy*- 'on, above (postposition)'; Orok *uwwə: ~ uwu ~ ui* 'upper side, upper space; high'; *u:-won*- 'to seat; to put; to mount smb on a deer or a horse'; Na. *uwui* 'upper; highest' (Onenko 1980: 423), *o:*- 'to get (in a boat, in a plane etc.); to start; to but in to conversation; to transmit a disease', *uyə* 'upper side', *uyə*- 'on smth.; above smth. (postposition)', *uyə-ji* 'upside down', *uyəktulə*- 'above (post)', *uy-lə* 'on top of (adverb)'; Oroch *ui*- ~ *uyi*- 'to raise', *uyə* 'upper', *ui*- 'upper side (postposition)'; *ụ:*- 'to sit down in a boat, on a sledge, to mount a horse etc.'; Ud. *ui*- 'above (postposition)', *una*- 'to sit down in a boat, to take a train, to mount a horse etc.; to get an animal trace'

pMo **öge* 'going up; going upward, upstream; being above' < ? **ög*- 'to go up' (pMo. *-*dA* adverbial marker deriving nominally encoded adjectives, e.g., WMo. *batu* 'firm' → *batu-da* 'firmly'; pMo *-*sA*- denominal verb suffix, e.g., WMo. *ayajim* 'slow, slowly (adv. / adj)' → *ayajimsa*- 'to slow down, become slow'): MMo. *o'ede* '1

upwards, upstream, above', WMo. *ögede* ‘upwards, uphill, upstream, towards’, *ögede bol-* 'to rise, get up' (WMo. *bol-* 'be(come), exist'), WMo. *ögse-* ‘2 to go up’, Khal. *ö:d* '1', *ögsö-* '2', Bur. *ö:de* '1', *ügse-* '2', Kalm. *ö:də* '1', *öksə-* '2', Dag. *wə:d* '1', Ordos *ö:dö* '1', Kgj *ʉde*, Dgx. *ojie* '1', Bao. *odə* '1', Mog. *öädä* '1'

pTk **ög-* ‘to raise, heap up' (OT *-(X)š* deverbal property noun/adjective, e.g. *arï-* 'to be clean, pure' -> *arï:š* 'clear, pure'; Erdal 1991: 262-275): OT *ük-* '1 to heap up', *üküš* 'many', Karakh. *ük-* 'to heap up; to accumulate', *üküš* 'many', MTk. *ük-* 'to accumulate', *üküš* 'many', Tk. *ögüš* ‘many’, Tat. *ŭy-* '1', Khak. *üg-* 'to heap up; to hill up, raise the earth around plants', Kirg. *üy-* '1', Kaz. *üy-* 'to heap up; to accumulate', Nog. *üy-* '1', Bash. *ŭy-* '1', KKalp. *üy-* '1', Uig. *üy-* '1', Uz. *uy-* 'to heap up; to accumulate', Altay *ü:-* '1', Dolg. *ügüs* 'many', Yak. *ügüs* 'many'

The sporadic attestation of the Tungusic vowel reflex *o* where *u* would be expected, for instance in Even *oy-* ‘above (postposition)’, *oɣər-* ‘to raise’ and in Nanai *o:-* ‘to get (in a boat, in a plane etc.); to start; to but in to conversation; to transmit a disease' may lead to the reconstruction of pTg **ög-* rather than **ug-* ‘to ride, mount’.[10]

The nominal adjective pMo **öge* 'going up' can be reconstructed, given the occurence of an adverbial marker **-dA*, which derives from nominally encoded adjectives and of a denominal verb suffix **-sA-* in Mongolic. Parallel to the derivation in the Tungusic cognate, it cannot be excluded that pMo **öge* 'going up' further derives as the verbal noun in pMo **-i* (Robbeets 2015: 462-463) from an original verb **ög-* 'to go up'. Similar to the *i* ~ *e* alternation in word pairs such as WMo. *kemeli-* ~ *kemele-* ‘to gnaw', the compounds **ög-i-de* (go.up-NMLZ-ADVZ) 'above' and **ög-i-se-* (go.up-NMLZ-VBLZ) 'to go up' may have assimilated to WMo. *ögede* ‘upwards, uphill, upstream, towards’ and *ögse-* ‘to go up’.

The historical stages of Turkic have a voiceless velar, but the modern forms lead to the reconstruction of pTk **ög-* ‘to heap up’ with a voiced velar stop.

pJ **taka-* ‘to be high, elevated’: J *taka-* (B), OJ *taka-* ‘to be high, elevated’, J *take* (2.3/ 2.4), OJ *take*$_2$ 'peak, mountain', Yoron (Amami) *takasa*ɴ 'tall', Shuri (Okinawa) *takasa*ɴ 'tall', Irabu (Miyako) *ta'aham* 'tall', Ishigaki (Yaeyama) *takasaa*ɴ 'tall', Yonaguni *thâgan* 'high, tall’, pR **taka-* ‘to be high, tall’ (Thorpe 1983: 296),

pK **tekɨ-* ‘to increase, make go up, make high': MK *teu-* ‘to increase (tr.)’, MK *te* ‘more, longer, further’, K *tek*, *teki* ‘plateau’

pTg **dege-* ‘to rise, go up, fly' (pTg **-dA-* fientive suffix; e.g. Even *ya:* 'what' → *ya:-da-* 'use what, make from what'): Ma. *dəyə-* ‘to fly’, *dəkdə-* ‘to rise; to float’, Sibe *dəyə-* ‘to fly’, *dəyə-tun* ‘aircraft’, Jur. *de-* ‘to fly’, Even *dəɣ-* ‘to rise; to ascend, to go up; to fly away’, *dəɣi* ~ *dəyi* ‘bird’, Neg. *dəɣ-* ‘to fly, to fly away’, *dəɣi*: ‘a bird’; Solon *degeli-* ‘to fly’, *degae-* ~ *dedde-* ‘to raise’, *degi* ‘bird’; Olcha *dəgdə-* ‘to fly’; Ud. *d’e:li-* ~ *d’eyli-* ‘to fly’, *dəgdə-* ‘to rise (about the sun); to raise a heavy thing; to go up on a wing, to learn to fly (about birdlings)’, Oroch *dəili-* ~ *də:li-* ‘to fly’, Na.

[10] According to the correspondences in Benzing 1955a:

pTg	Ma.	Jur	Evk	Even	Sol	Neg	Oroch	Ud.	Olch.	Orok	Na
*ö [ʌ]	u		u	o	u	u	o/u	o	o/u	o/u	u
*u	u		u /-ï-	u/ -ï-	u/ -ï-	u/-ï-	u	u	u	u	u

dəgdə- ‘1 to fly, 2 to go up to the water surface, to go up (e.g. about dust)’, *dəgdə-gu-* ‘to rise (about the sun)’

pMo. **dege-* ‘to rise, go up': WMo. *degere* ‘top, on top of, high; higher or better than (with the ablative) (n. / adj /postpos.)’ (WMo *-ra* / *-re* adverbializer < *-*r* deverbal noun + *-*a* dative; Robbeets 2015: 355-357), WMo. *deɣedü* ‘higher, upper, best (adj.)’ (WMo. *-du* / *-dü* derives local nominal adjectives; Poppe 1954: 50), WMo. *degegši* ‘upward, above, higher than (adv.)’ (WMo. *-ɣši / -gši* derives directional adverbs; Poppe 1954: 58), WMo. *degde-* ‘to rise, float, fly up (intr.)’ (WMo. *-da-* / *-de-* fientive/passive suffix; Robbeets 2015: 305-306), WMo. *degüli-* ‘to jump across, jump a distance, leap’ (pMo *-*li-* intensive), MMo. *de'ere* ‘top, on top of, high’, Khal. *de:-* ‘1 above, up’, *degde-* ‘2 ‘to rise, fly up (intr.)’, *dü:lle-* ‘3 to jump, leap’, Bur. *de:r(e)* 1, *degde-* 2, Kalm. *de:-* 1, *degdə-* 2, Ordos *de:-* 1, *degde-* 2, Dgx. *ǯierə* 1, *ǯiedu* ‘higher, upper, best (adj.)’, Bao. *de-Goŋ* 1, Dag. *de:re* 1, *derede-*, *degede-* 2, Mgr. *dəre* 1, *dē-di*, *te-šə* 1, *digine:-* ‘to jump on one leg’, *dü:li-* ‘to jump, dance, beat (heart)’, Mog. *de:rä* ‘top, on top of, high’, *dēkši* ‘upward, better than’, *dei-du* 'above', *dekšə-* ‘to come up’

pTk **yeg* ‘upper part’: OTk. *yeg* ‘1 better than (adv. with object of comparison in the ablative)’, Karakh. *yeg* ‘1', Tk. *yeɣ*, *yey* ‘1', MTk. *yik*, Az. (dial.) *yeg* ‘1' , Tkm. *yeg* ‘1', Chu. *śi* ‘upper part, surface’

According to Martin (1996: 30) the MK verb *teu-* ‘to increase (tr.)’ and its adverb MK *te* ‘more, longer, further’ may have been derived from an original verb pK **teɣɨ-* < **tekɨ-*. The elision of the velar stop assumed here is reminiscent of the derivation of the MK verb *tao-* ‘to get exhausted, come to an end’ and its adverb *¨ta* ‘all’ from **taɣʌ-* < **takʌ-* by Martin (1996: 79). The latter derivation is supported by the rising tone of the adverb and by the preservation of a velar in MK *takoy* ‘all’, which is probably a lexicalized deverbal noun. Even though in the case of MK *teu-* ‘to increase (tr.)’, the adverb *te* ‘more, longer, further’ lacks the rising tone, additional support for an original velar is provided by K *teki* ‘plateau’, which may be a lexicalized deverbal noun.

The common semantic feature in all branches is ‘to rise, go up'. As indicated by the Even meaning ‘to rise, to ascend, to go up, to fly away’ (Doerfer et al. 1980: 168), the Tungusic semantics ‘to fly’ derive from ‘to go high’.

Since the suffix in WMo. *dege-re* ‘top, high; higher or better than’ is the adverbializer WMo *-ra* / *-re,* which derives from a deverbal noun suffix *-*r* in the dative *-*a*, it is clear that we are dealing with an original verbal base **dege-* 'to rise, go up'. The verbal origin is further supported by other lexicalizations, including various deverbal suffixes such as the fientive/passive suffix or the intensive suffix. OTk. *yeg* ‘better than’ has the same semantics as the Mongolic adverb and also puts its object of comparison in the ablative. In Chuvash Chu. *śi*, *śiye* ‘upper part, surface’ the meaning ‘high place’ is preserved.

pMo **kali-* 'to rise, go up, fly': WMo. *qali-* 'to soar, fly, skim (in flying); to flow over the brim, overflow', *qalil* 'steep slope', Khal. *xali-* 'to soar, sail (of bird)'; to overflow; to exceed, go beyond', Bur. *xali-* 'to soar, fly', Kalm. *xäl̇ə-* 'to rise; to fly up; to be very high; to overflow (of rivers); to emigrate (of people)', Ordos *xali-* 'to soar, fly'

pTk **kalï-* 'to rise' (pTk *-*(X)k* deverbal inchoative, e.g., OT *tar-* 'to disperse, scatter' → *tarïk-* 'to be driven away, go away'; Robbeets 2015: 264-266): OT *ḳalï-* 'to rise in the air; jump', Karakhanid *ḳalï-* 'to rise in the air; jump', Tk. *ḳalḳ(ï)-* 'to rise; to emerge; to depart, to start; to be cancelled; to disappear; to fall off, peel off; to be

collected, harvested', Az. *ġalχ-* 'to rise; to increase (intr.)', Tkm. *ġalk-* ' to increase (intr.)', Tat. *ḳalḳ-* 'to rise; to float (on a surface), to stand up, overtop', Kirg. *ḳalḳï-* 'to float (on a surface)', Kaz. *ḳalḳï-* ''to soar (of a bird); to float (on a surface); to remove fat from the surface of soup', Nog. *ḳalḳ-* 'to float (on a surface)', Bash. *ḳalḳ-* 'to rise; to emerge; to develop', KKalp. *ḳalḳï-* 'to float (on a surface); to swell; to take off', Kum. *ḳalḳ-* 'to float (on a surface); to wiggle, jiggle', Uz. *qalq-* 'to rise', Khak. *xalï-* 'to rise; to jump up', Yak. *kïlïy-* 'to rise on one leg', Tuva *xalï-* 'to rise, run'

Although the semantic association between 'to rise' and 'to fly' is not included in List et al. (2014), the polysemy is frequently attested on various lexemes in individual Transeurasian languages, such as in Even *dəɣ-* ‘to rise; to ascend, to go up; to fly away’ or in Kalmuck *xälə-* 'to rise; to fly up'. Therefore the semantic comparison between both meanings is legitimate.

189. TO BREAK

pJ **kaka-* ‘to break’: J *kaku,* MJ *kak-* (A) ‘to break, lack’, J *kakeru* (A)*,* OJ *kake*$_2$*-* ‘to break off, be broken off’

pTg **xaka-* ‘to break, cut, tear’: Neg. *aki- / kaki-* ‘to cut off branches or twigs from a tree, to lop’, Olcha *xəkpə-ču-* ‘to break off, to bite off’, *χaqpa-lu̥-* ‘to break a dead tree’, Orok *χaqpa*: ‘a piece’, *χaqpa-* ‘1 to take away; 2 to separate, to break off, to tear off’, Na. *χaga-* ‘to cut, to cut oneself’, *χa:ga-qta-* ‘to cut off many objects’, *χa:ga-oxã* ‘a wound’, *χa:ga-nase-* ‘to cut many times’, *χaqpa:-, xəkpə-* ‘to be torn off; to fall off’, *χaqpa-li-* ‘to tear off’, Ud. *akpinda-* ‘to prick, to stab; to cut off, to rip up’, *kakpaligi-* ‘to tear off’

pMo **kaka- ~ *kaga-* ‘to break, split’ (pMo **-l-* intensive-iterative, **-rA-* anticausative, **-cA-* progressive): MMo. *qaqal-* ‘1 to break, split, chip (tr.)’, *qaqaca-* ‘2 to separate, break away from (tr./intr.)’, MMo. *qaqara-* '3 to break, split, burst (intr.)'; WMo. *qaɣal- ~ qaɣala-* 'to break, split, chip, cut through (hard objects); to make an incision; to draw a dividing line; to plow or till land (tr.)' (WMo. *-l-* intensive-iterative, e.g. WMo. *dusu-* ‘fall (of drops)’ → *dusul-* ‘to drip’; Poppe 1954: 64, Robbeets 2015: 106), *qaɣaca-* ‘to break away from, separate, part with; to be separated (tr./intr.)’ (WMo -cA- progressive suffix for continuous action, e.g. WMo. *ergi-* ‘to turn or move around’ → *ergice-* ‘to keep going or coming back, make rounds’; Robbeets 2015: 199), *qaɣara- ~ qaɣar-* 'to break, fall asunder; crack, crash, split burst (intr.)' (WMo. *-rA-* anticausative; Robbeets 2015: 313, Poppe 1954: 65), Khal. *xagala-* ‘1’, *xagaca-* '2'*, xagara-* '3', Bur. *xaxal-* ‘1’, *xaxasa-* '2'*, xaxar-* '3', Kalm. *xaɣl-* ‘1’, *xaɣc-* '2', *xaɣr-* '3', Ordos *xagal-* ‘1’, *χaǧar(a)-* '3', Mog. *qaqala-* 'to cut up', *qaqara-* '3', Dag. *ġaǧalu-*, *xagala:-*, *hagere-*, *hagare-*, *hagela:-* ‘1’, *xaɣər-* *'3',* Eastern Yugur *χaǧača-* '2', *χaǧara:- ~ hqara-* '3', Mgr. *xġalə-* '1', *χa:ʒä-* '2', *xġara:-* '3', Bao. *ġal-* '1', *ġar-* '3', Dgx. *ġaǧalu-* '1', *ġaǧaca-* '2'*, ġaǧara-* '3'

pTk **kak-* ‘to beat, hit, injure, mow': Karakh. *kak-* ‘beat, hit, knock, tap’, Tk. *kak-* 'to beat, hit; beat into, nail, hammer; to push; engrave, stamp', Gag. *ḳaḳ-* 'beat into, nail, hammer', Az. *ġaχ-* 'to beat, hit; to say to one's face', Tkm. *kak-* 'to beat into, nail, hammer; to shake (off), jolt; to slam, clap'; Uzb. *kɔk-* 'to beat, hit; 'to knock, tap; to nail, hammer'; to shake (off), jolt; to slam, clap', Uig. *kak-* 'to beat, hit; nail, hammer; to shake (off), jolt; to knock, tap; to wave one's hand; to slam, clap; to flap one's wings'; Karaim *kak-*'to beat, hit; 'to knock, tap; nail, hammer; to thresh'; Tat. *kak-* 'to beat, hit; to knock, tap; nail, hammer; to shake; to slam, clap; to flap one's wings'; Bash. *kak-* 'to beat, hit', Kirg. *kak-* 'to knock, tap; to slam, clap; nail, hammer; to shake; to repel; to drive game', Kaz. *kak-* 'to knock, tap; nail, hammer; to shake; to

slam, clap; to hit, run over (of a car); to steal'; KBalk. *kak*- 'to beat, hit; to knock, tap; to shake; to wave one's hand; to print; to kill', KKalp. *kak*- 'to beat, hit; to beat into, nail, hammer; to shake; to knock, tap; to catch (a thought); to offend', Kum. *kak*- 'to beat, hit; to beat into, nail, hammer; to shake; to knock, tap; to slam, clap; to wave one's hand'; Nog. *kak*- 'nail, hammer; to shake; to knock, tap; to wave one's hand; to slam, clap; to charm', Khak. *xax*- 'to shake off; to throw out, dump; to play an instrument' , Tuva *kak*- 'to beat, hit; to knock, tap; to nail, hammer; to urge on (a horse); to mow; to depart', Tofa. *ka'k*- 'to beat, hit; to knock, tap; to mow; to send (a telegram)', Dolg. *kakrïy*- 'to break into small pieces'

In Mongolic, the Buriat and Moghol suggest **kaka*-, while Eastern Yugur *χaġara:- ~ hqara*- preserves an alternation between both variants **kaka*- and **kaga*-. The other Mongolic languages lead to the reconstruction of **kaga*-.

pJ **yanpu*- 'to break, tear, split' : J *yaburu* (?B'), OJ *yabur*- 'to tear, rip, break', J *yabureru* (?B'), OJ *yabure*- 'to get torn, get burst, be worn out', Yonamine (Amami) *yaɴ ~ yaɴbiɴ* 'to tear', Shuri (Okinawa) *yayuɴ ~ yanzuɴ ~ yanduɴ* 'to tear', Shuri *yandi ~ yaburi* 'to get torn', Irabu (Miyako) *yabuï ~ yaï* 'to tear', Ishigaki (Yaeyama) *yaruɴ* 'to tear', Yonaguni *nùda* 'to tear'
pTg **delpe*- 'to split, break': Ma. *dəlhə*- 'to divide, to separate', *dəlhə-bu*- 'to make split; to cut up (a slaughtered animal)', *dəlhən* 'dividing, separation', Sibe *dəlhə*- 'to separate; to split; to divorce', Na. *dətpəjkin*- 'to split, to crack', Evk. *dəlpəm- ~ dəlpərgə*- 'to crack (about ice); to burst' (*-rgA*- decausative inchoative), Even *delke*- 'to split, separate', Solon *delpe*- 'to split, to crack'
pMo **delbe*- 'to burst, break' (pMo **-lA* intensive-iterative; pMo **-rA* anticausative) WMo. *delbele*- '1 to break, crack (tr.)', WMo. *delbere*- '2 to burst, go to pieces (intr.)', Khal. *delbele*- '1', *delbere*- '2', Bur. *delbel*- '1', *delber*- '2', Kalm. *delwḷ*- '1', Ordos *delbel*- '1'

Robbeets (2005: 375; 2008) argues that the voiced series in Japanese, which are internally derived from original nasal clusters, can be traced back to clusters in the Transeurasian languages. The original clusters can be divided into homoganic and heteroganic clusters. Homoganic clusters are composed of a sonorant and a stop (pTEA **-Rp*-, **-Rt*-, **-Rk*-) and merge in a nasal cluster (pJ **-np*- > OJ *-b*-, pJ **-nt*- > OJ *-d*-, pJ **-nk*- > OJ **-g*-) in Japanese. The sound correspondence reflected here, namely OJ *-b*- < pJ **-np*- with pTg **-lp*- and pMo **-lb*- is regular from this perspective. Another illustration of a homoganic cluster correspondence is provided by etymology 32 BIG, 37 LEG/FOOT and 100 CRUSH.
In heteroganic clusters, on the other hand, the nasal and the stop have a different place of articulation, which results in the insertion of a parasitic stop (pTEA **-m*$^{(P)}$*T*-, **-n*$^{(T)}$*K*-, **-ŋ*$^{(K)}$*T*-). The nasal is lost in the continental Transeurasian languages (**-PT*-, **-TK*-, **-KT*-), whereas Korean and Japanese lose the final stop (pJ **-mp*- > OJ *-b*-, pJ **-nt*- > OJ *-d*-, pJ **-ŋk*- > OJ **-g*-.) Etymology 96 WIDE illustrates a heteroganic cluster correspondence.

pTg **butu*- 'to break': Evk. *butukte* 'piece', Even *bụ:t*- 'to break in pieces (glass, dishes etc.)', Neg. *boktal*- 'to break off', *boktakin*- 'to get broken off; to crack', Olcha *bụqta* 'piece', *bụqtaǯa*- 'to get broken off', *bụqtalụ*- 'to break in two', *bụqtalčụ*- 'to break in pieces', Na. *boqta:*- 'to break off', Oroch *buktaga*- 'to be broken, Ud. *buktala- ~ buktali*- 'to break across', *buktaga*- 'to be broken across'

pMo **buta*- 'to break (pMo *-*l*- intensive-iterative; pMo *-*rA* anticausative): WMo. *buta* 'in fragments, in pieces, completely broken (adverb)', *butara*- 'to break to pieces, smash; to disperse, scatter (intr.)', MMo. *butăra*- '1 to be broken', Khal. *butal*- 'to break', *butar*- '1', Kalm. *butu* 'piece of dust (in the air)', *butṛ*- 'to scatter, to disperse (e.g. of dust by the wind) (intr.)', Ordos *butara*- '1', Dgx. *pudura-~ putura*- '1', Mgr. *pudəra:*- '1'

191 TO SPIN

pJ **tumu* 'spindle': J *tumu* (2.4), OJ *tumu* 'spindle', J *tumug*- (B), OJ *tumug*- 'to spin, make into yarn', Shuri (Okinawa) *çing*- (B) 'to spin'

pTg **tom(u)*- > *tumu*- 'to spin' (pTg *-*ku* ~ *-*ko* deverbal instrumental noun suffix, e.g. Na. *xado*- 'to mow' → *xadoko* 'scythe'): Even *tum*- '1 to spin, wind, coil, spool, wrap', *tumenŋe* 'thread wind around a bobbin', *tomqo*- 'to spin strings for threads; to spin threads', *tomqon* 'spinning of strings; yarn'; Evk. *tum*- ' 1', *tomko* 'a thread'; *tomko*- 'to spin threads; to tie with a thread'; Neg. *tum*- '1', *tumu* 'string of thread, parcel, roll', *tomko* 'thread', *tomko*- 'to spin threads'; Solon *tum*- 'to bind, knit, string together, tie', *toŋxo*- 'to spin threads'; Oroch *tumu*- '1', *tompo* 'a sinew or nettle thread', *tompo*- 'to spin threads; to weave a net'; Olcha *tumu*- '1', *toŋpo* 'short threads of nettle or hemp', *toŋpo*- 'to spin threads'; Orok *tumu*- '1', *toqpo*- ~ *topqo*- 'to spin threads, rope, cord'; *toqpo* ~ *topqo* 'thread; rope'; Na. *tumu*- '1', *tompo*- 'to spin threads of fishskin', *tompo* 'threads made of fishskin'; Ud. *tompo*- 'to spin threads, ropes'

pMo **tomu*- ~ **tamu*- 'to spin': WMo. *tomu*-, *tamu*- '1 to twist or spin thread or rope', MMo. *tamu*- ~ *toma*- ~ *tomu*- ~ *doma*- '1', Khal. *tam*- ~ *tom*- '1', Bur. *tomo*- '1', Ordos *tamu*- '1', Kalm. *tom*- ~ *töm*- 'to twist, twine; to string together (rope), make rope (by turning horse hair between the hands)', Eastern Yugur *tomu*- ~ *tɔmɔ*- ~ *tomə*- '1', Dgx. *tomu*- '1', Bao. *toməl*- '1', Mgr. *tomu*- ~ *tamu*- '1'

The Tungusic reconstruction displays a vowel alternation between **tom(u)*- and **tumu*- 'to spin'. Given the preservation of **tom(u)*- in derived nouns with the instrumental suffix and re-verbalizations thereof, I assume that **tom(u)*- assimilated to **tumu*-.

In Mongolic we find a vowel alternation between **tomu*- ~ **tamu*- 'to spin', reminiscent of the alternation between pMo **dalan* 'seventy' and **dolaan* 'seven' (Nugteren 2011: 512).

194. TO OPEN

pJ **aka*- 'to open': J *ak*- (A) 'to open, be opened, become vacant (intr.)', J *ake*-, OJ *ake*$_2$- (A) 'to open (tr.); to get bright (intr.)', J *aki* 'gap, opening, aperture', Yonaguni *ágirun* 'to open'

pK **akɨ*- 'to open' (pK *-*s* resultative deverbal noun suffix, e.g. K *cilki*- 'to be tough, be durable, be lasting, be persisting' → *cilkis* 'firm, unyielding'; Robbeets 2015: 422, pK *-*i* deverbal noun suffix, e.g. MK *kiph*- 'be deep' → *kiphuy* 'depth'); Robbeets 2015: 459-460)': K *akali* 'mouth, opening', K *akus* 'a little open', MK *akwui* 'mouth, opening', MK *akwungi* 'mouth, beak'

pMo **agu*- 'to open' (pMo *-*i* deverbal noun, e.g., WMo *bü*- 'to exist' → *büyi* 'existence'; Robbeets 2015: 462-463): WMo. *aɣu*- 'to scoop out, to drain (tr.)', *aɣud*- 'to spread, become extended, vast (intr.)', *aɣudal*- 'to open (as a bag), to expand, extend (tr.)', WMo. *aɣuyi* 'cave, grotto; vast, extended, great'; Khal. *aguy* 'cave', *aguu*

'mighty, great', Bur. *agɨ* 'cave, hole’, Kalm. *aɣu:* 'grotto, cave; vast and great', *aɣ* 'small holes, gap', Ordos *aG*^{w}*i*: 'cave, hole’, Dag. *agui* 'cave, hole’

The semantic development from 'to open' to 'to open up, extend' with derived nouns meaning 'opening' and 'vast' proposed for Mongolic is frequently seen cross-linguistically, for instance also in the connection between OJ *paruk*- ‘to open up' and OJ *paruka* ‘far, distant, remote’ or between OJ *pi*$_1$*rak*- 'to open' and OJ *pi*$_1$*ro*- ‘wide, broad, spacious’.

pK *yele- 'to open': K *ye: l-*, MK*·yel*- ~ *¨ye(l)*- 'to open; start up, set up; hold (e.g. banquet)'
pMo **yara*- > **ira*- 'to open': WMo. *yara*- ~ *ira*- 'to open wide; to expose; to draw open; to cut open; to split', MMo. *ya:ra*- ~ *yarǎ*- '1 to open wide, split open', Khal. *yara*- 'to pull open, to pull apart, to slit, to part', Bur. *yara*- '1', Kalm. *ir*- 'to cut open, to split with a knife, to plough', *yar*- 'to split, to slaughter, separate meat in pieces and remove the soft parts from the bones', Ordos *ir*- '1', Dgx. *yara*- '1', Mgr. *ya:ra*- '1'
pTk **ya:r*- 'to split': OT *yar*- 'to split, cleave (with a sharp instrument)', Karakhanid *yar*- '1 to split', Tk. *yar*- 'to split, rend, cleave, cut through; to break through (of military)', Az. *yar*- '1', Tkm. *ya:r*- '1', Gag. *yar*- '1', Tat. *yar*- '1', Kirg. *jar*- '1', Kaz. *žar*- '1', Nog. *yar*- '1', Bash. *yar*- '1', Balk. *jar*- '1', Karaim *yar*- '1', Kkpak *žar*- '1', Salar *yar*- '1', Kum. *yar*- '1', Uz. *yɔr*- '1', Uig. *ya(r)-* '1', S-Yug. *yar*- '1', Shor *čar*- '1', Khak. *čar*- '1', Tuva *čar*- '1', Yak. *sar-ka:x* 'split (adj.)', Dolg. *sar-ka:k* 'split (adj.)', Chu. *śor*- '1'

Although the semantic association between 'to open' and 'to split' is not explicitely mentioned in List et al. (2014), the polysemy can be observed in WMo. *yara*- ~ *ira*- 'to open wide; to split'.

199. UDDER
pMo **deleng* ~ **deling* 'udder': WMo *deleng* '1 udder, teat', Khal. *delen(g)* '1', Bur. *delen(g)* '1', Ordos *deliŋ* '1', Kalm. *delŋ* '1', Dag. *delin* '1', Eastern Yugur *deleŋ* '1', Mgr. *dəlaŋ*, *diliŋ* '1', Bao. *dəlaŋ* '1', Kgj. *deliɔ* '1', Dgx. *ʒielien* '1'
pTk **yėlin* 'udder': Karakhanid *yelin* '1 udder', Tk. *yelin* '1', Az. *yelin* '1', Tkm. *yelin* '1', Gag. *yelin* '1', Tat. *ʒilen*, Kirg. *jelin* '1', Kaz. *želin* '1', Nog. *yelin* '1', Bash. *yelen* '1', Balk. *yelin*, *jelin*, *želin* '1', Kkpak *želin* '1', Kum. *yelin* '1', Uig. *yelin*, *yilim*, *žilim* '1', Yak. *silin* '1', Chu. *śilə* '1'

In Mongolic, Ordos *deliŋ,* Dag. *delin,* Mgr. *diliŋ* and Kgj. *deliɔ* seem to reflect a vocalic alternant pMo **deling*, which agrees with the Turkic cognate (Nugteren 2011: 315). This alternation is reminiscent of *i* ~ *e* alternation elsewhere in Mongolic, e.g., WMo. *kemeli*- ~ *kemele*- ‘to gnaw'; see 186 RISE for an alternation in proto-Mongolic. The final velar nasal in the Mongolic reconstruction may preserve a trace of the body part suffix pMo *-*GA*; see 30 TOOTH and 31 HAIR.

200. FIVE
pK **ta*- 'five' (pK *-*[C]ʌ/ɨs* suffix deriving low numerals): K *tases*, MK *ta·sos* 'five', K *tas mal* 'five mal (measure)', *tas toy* 'five toy (measure)', *tas kop* 'five hop (measure', *tas ton* 'old Korean nickel' (ton 'money), etc.

pMo **ta-* 'five': WMo. *tabu(n)* 'five', *tabi(n)* '2 fifty', MMo. *tabun* '1', *tabin* '2', Khal. *tav(an)* '1', *tav'* (*tavin*) '2', Bur. *taba(n)* '1', *tabi(n)* '2', Kalm. *tawn* '1', *täwn* '2', Ordos *tawu* '1', *tawi* '2', Dag *ta:wu*, *ta:wun* '1', *tabi*, *tab́* '2', Eastern Yugur *ta:βən '1', taβən* '2', Mgr. *ta:wən*, *ta:wun* '1', *tayin* '2', Bao. *tawuŋ* '1', Dgx. *tawuŋ* '1', Mog. *tabun* '1'

In Korean, the numbers MK *ta·sos* 'five' and MK *ye·sus* 'six' seem to contain a suffix MK *-u/os*; they occur in several shorter variants such as *tas ton* 'old Korean nickel' in which the *-s-* is a genitive and attributive marker and *ton* means 'money'. Since the medial *-s-* in both numbers did not undergo the expected lenition to *-ž-* in the environment **CVCʌ/i*, Martin (1996:55) suggests to derive both forms from **tas·[C]ʌs* and **yes·[C]ɨs*, in which the consonant cluster blocks the lenition. As such pK *-*[C]ʌ/ɨs* could be reconstructed as a suffix deriving low numerals.
The common element in the numerals for 'five' and 'fifty' is pMo **ta-*. The suffix *-*bun* in the lower numerals may be connected to the suffix *-*rbAn* in, **gu-rban* 'three', **dö-rben* 'four' and **ha-rban* 'ten'. The suffix in decimal numeral units in 'fifty' seems to be *-*bin*, while in the numerals 'thirty' and 'forty' it appears as *-*cin*: **gu-cin* 'thirty' and **dö-ci(n)* '2 forty'; see 165 FOUR.

3. Regular sound correspondences

Table 3.1 Reconstruction of the basic consonant inventory of Proto-Japonic

pJ	OJ	J	Amami	Okinawa	Miyako	Yaeyama	Yonaguni
*p	p	h-<br>-w- -ø-	ɸ- h- ç-<br>-ø-	p- ɸ-<br>-ø-	p- f-<br>-ø-	p-<br>-ø-	ɸ- h- tɕ^ʔ- ç-<br>-ø-
*np	^{n}b	b	b	b	b	b	b
*t	t	t	t	t	t	t	t
*nt	^{n}d	d	d	d	d	d	d
*k	k	k	k- k^h-<br>-k- -k^h-	k-<br>-k-	k- f-<br>-k- -f-	k- ɸ- f- h-<br>-k-	k- k^h- ø-<br>-g-
*nk	^{n}g	g	g	g	g	g ŋ ø	ŋ
*s	s	s	s	s	s	s	s c
*ns	^{n}z	z	^{d}z	z	z	z	d
*m	m	m	m	m	m	m	m
*n	n	n	n	n	n	n	n
*r	r	r	r	r	r	r	r
*w	w	w	w- b- y- g- ɸ-<br>-ø-	w- b- g- ʔ-<br>-ø-	b-<br>-ø-	b-<br>-ø-	b-<br>-ø-
*y	y	y	y	y	y	y	d-<br>-y-

Table 3.2 Reconstruction of the basic vowel inventory of Proto-Japonic

pJ	OJ	J	Amami	Okinawa	Miyako	Yaeyama	Yonaguni
*a	a	a	a	a	a	a	a
*ə	o$_{(2)}$	o	u	u	u	u	u
*o	o$_{(1)}$	o	u	u	u	u	u
*o	u	u	^{h}u	u	u	u	u
*u	u	u	^{ʔ}u, N	u, N	u, N, ø	u, N, ø	u, N, ø
*ɨ	o$_{(2)}$	o	u	u	u	u	u

*i	i$_{(1)}$	i	^{ʔ}i, N	^{ʔ}i, ^{y}i, N	ʅ, ɯ, s, N, ø	N, ø	i, N, ø
*e	i$_{(1)}$	i	^{h}ɨ, i	^{h}i, i	i	i	i
*e	e$_{(1)}$	e	yu	yu	yu	yu	du

Table 3.3 Reconstruction of the basic consonant inventory of Proto-Koreanic

pK	MK
*p	p, W /β/ > w (lenition)
*t	t, l /r/ (lenition)
*c	c
*k	k, G /ɣ/ > ø (lenition)
*h	h
*s	s, ž (lenition)
*m	m
*n	n
*r	l

Table 3.4 Reconstruction of the basic vowel inventory of Proto-Koreanic

pK	MK
*a	a
* ʌ	o /ʌ/
*ɨ	u /ɨ/
*e	e
*o	wo /o/
*u	wu /u/
*i	*i
*ia	*ye

Table 3.5 Reconstruction of the basic consonant inventory of Proto-Tungusic

pTg	Jur.	Ma.	Sibe	Oroch	Ud.	Na.	Na. Bikin	Orok	Olcha	Even	Sol.	Evk.	Neg.
*p-	f-	f-	f-	x-	x-	p-	f- x-	p-	p-	h-	ø-	h-	x-
*-p-	f	f b ø	v ø	p w ø	p f w	p	f	p	p	b w ø	p w g ø	p b w ø	p w
*b-	b-	b-	b-	b-	b-	b-	b-	b-	b-	b-	b-	b-	b-
*-b-	b w ø	b f w ø	v ø	b w ø	b w ø	b w ø	w ø	b w ø	b w ø	b w ø	b p w ø	w ø	w ø
*t-	t-	t-	t-	t-	t-	t-	t-	t-	t-	t-	t-	t-	t-
*-t-	t	t	t s	t	t	t	t	t	t	t	t	t	t
*d-<br>*ji-	d-<br>ʤi-	d-<br>ʤi-	d-<br>ʤi-	d-	d-	d-<br>ʤi-	d-<br>ʤi-	d-<br>ʤi-	d-	d-	d-	d-	d-
-d-<br>-ji-	d<br>ʤi	d<br>ʤi	d<br>ʤi	d	d	d<br>ʤi	d<br>ʤi	d<br>ʤi	d	d	d	d	d
*k-	x-	x-	x-	k- ø-	k- ø-	k- ø-	k- ø-	k- ø-	k- ø-	k- ø-	x- ø-	k- ø-	k- ø-
*-k-	k x ø	k x	k ɣ	k ø	k x ɣ ø	k ɣ ø	k ɣ ø	k ø	k ɣ ø	k	k x	k x	k x

*g-	g-	g-	g-	g- ŋ-	g- ŋ-	g- ø-	g- ø-	g- ŋ-	g- ŋ-	g- ŋ-	g- n-	g- ŋ-	g- ŋ
*-g-	ɣ w ø	ɣ w y ø	ø	ɣ w y ø	ɣ w y ø	ɣ w y ø	y ø	ɣ w y ø	ɣ w y ø	ɣ y	ɣ ø	ɣ	ɣ y w
*č-	č-	č-	č-	č-	č-	č-	č-	č- > t-	č- > t-	č-	s-	č-	č-
*-č-	č	č	č	č	s	č	č s	č > t	č	č	š	č	č
*x-	w- ø-	w- ø-	v- ø-	x- ø-	w- ø-	x- s-	x- s-	x- s-	x- s-	ø-	ø-	ø-	ø-
*-x-	x	x	x k ɣ	k	ø	x ø	x k	x ø	x ø	k	x	k	k x
*s-	s-	s-	s-	s-	s-	s-	s-	s-	s-	s-	s-	s-	s-
*-s-	s	s	s	s	s h ø	s	s	s	s	s	s	x	s
*m-	m-	m-	m-	m-	m-	m- ŋ-	m-	m-	m- ŋ-	m-	m-	m-	m-
*-m-	m	m	m	m	m	m	m	m	m	m	m	m	m
*n-	n-	n-	n-	n-	n-	n- l-	n- l-	n- l-	n- l-	n-	n-	n- l	n-
*-n-	n	n	n	n	n	n	n	n	n	n	n	n	n
*-r-	r	r	r	y ø	y ø	r	r	r	r	r	r	r	y ø
*-l-	l	l	l	l	l	l	l n	l	l	l	l	l	l

Table 3.6 Reconstruction of the basic vowel inventory of Proto-Tungusic

pTg	Jur.	Ma.	Sibe	Oroch	Ud.	Na.	Na. Bikin	Orok	Olcha	Even	Sol.	Evk.	Neg.
*a	a	a	a	a	a	a	a	a	a	a	a	a	a
*e	e	e	e	e	e	e	e	e	e	e	e	e	e
*o	o	o	o	o	o	o	o	o	o	o	o	o	o
*ö	u	u	u	o u	o	u	u	o u	o u	o	u	u	u
*u	u	u	u	u	u	u	u	u	u	u ö	u ö	u ö	u ö
*ü	u ei	u ei	u i	i	i u	u o	i o	i u	i u o	i	i	i u	i o
*i	i	i	i	i	i	i i̯	i i̯	i	i i̯	i	i	i i̯	i i̯
*ia	ie ^{i}a	iya ai ^{i}a	ia a	^{i}æ ei	^{i}æ æ a	^{i}a ea	^{i}æ	i̯:	i̯:	ia	i̯:	i̯:	i̯:

Table 3.7 Reconstruction of the basic consonant inventory of Proto-Mongolic

PMo	MMo	WMo	Khal.	Bur.	Kalm.	Dag.	EYu.	Mgr.	Dgx.	Bao.	Mog.
*p	h VøV Cb	ø	ø	ø	ø	x š	h	x f š	x f š	x f š	ø ʔ
*b	b	b	b- -v-	b	b- -w-	b -v- -r	b- p- -w-	b -ø- -yi	b- p- -v-	b- p- -v-	b -b- -f
*t	t	t	t	t	t	t	t d	t d	t -d	t	t
*d	d -t	d	d	d	d	d -r	d	d	d j	d	d
*k(A)	q x	q	x	x	x	k x	q x	x	q	x	q
*k(E)	k	k	x	x	k	k x	k	k	k	k	k
*k(i)	k	k	x	x	k	k x	k	ć	k	k	k
*g(A)	q -ɣ-	ɣ	g	g	g	g	ġ	ġ	ġ	ġ	ɣ
*g(E)	g	g	g	g	g	g	g	g	g	g	g
*g(i)	g	g	g	g	g	g	g	g	g	g	g
*č	č	č	č	s š	č	č	ć ǯ	č -ǯ-	č -ǯ-	č -ǯ-	č

										-ć	
*s	s š	s	s š	h š<br>-t	s š	s š<br>-r	s š	s š ʒ	s š	s š	s š
*m	m	m	m<br>-m -n	m<br>-m -n	m<br>-m -n	m<br>-m -n	m	m	m<br>-n	m	m
*n	n	n	n	n	n	n	n	n	n	n<br>-ŋ	n
*-r-	-r-<br>-r	-r-<br>-r	-r-<br>-r	-r-<br>-r	-r-<br>-r	-r-<br>-r	-r-<br>-r	-r-<br>-r	-r-<br>-ø	-r-<br>-r	-r-<br>-r
*-l-	-l-<br>-l	-l-<br>-l	-l-<br>-l	-l-<br>-l	-l-<br>-l	-l-<br>-l	-l-<br>-l	-l-<br>-l	-l-<br>-n	-l-<br>-l	-l-<br>-r

Table 3.8 Reconstruction of the basic vowel inventory of Proto-Mongolic

PMo	MMo	WMo	Khal.	Bur.	Kalm.	Dag.	EYu.	Mgr.	Dgx.	Bao.	Mog.
*a	a	a	a	a	a ä	a	a	a ä i<br>ø-	a ə	a e i	a o ö
*e	e	e	e i ö	e ü	e i ö	e ü	e<br>i- o-<br>ø-	e ə a i<br>u<br>ø-	e ie ü<br>ye-	e<br>-iN<br>-aN	e ü
*o	o	o	o	o	o ö	o<br>-(u)a-<br>wa-	o ö	o u ö<br>u- ø-	o u<br>-uaN	o u<br>o-	o u
*ö	o u	ö	ö	ü	ö	ü	ö o:	u o o:<br>o- ø-	o u<br>-uaN	o u<br>o-	ö ü
*ü	u	ü	ü	ü	ü	ü	u ə<br>ø- u-	u ə i<br>ø- u-	u	u e<br>u-<br>-oŋ	ü
*u	u	u	u	u	u ü	u o<br>-(u)a-<br>wa-	u ə<br>ø-	u o ə<br>ø-	u	u e a<br>o	u
*i	i	i	i	i e	i	i	i	i	i ə	i	i

Table 3.9 Reconstruction of the basic consonant inventory of Proto-Turkic

	OT	Chu.	Kh.	CTat	Kum.	KBal.	Krm.	Tat.	Bash.	Kaz.	Kirg.	Kpak	Nog.
-p-<br>-p	p<br>-p	b<br>-p	b<br>-p	b p<br>-p	b<br>-p	b<br>-p	b<br>-p	b p<br>-p	b<br>-p	b<br>-p	b p<br>-p	b p<br>-p	b<br>-p
*b-	b-	p-	b-	b-	b-	b-	b-	b-	b-	b-	b-	b-	b-
-b-<br>-b	b	v ø	v ø	v	w y ø	w y ø	w y<br>ø	w y<br>ø	w y<br>ø	b w<br>y ø	b y<br>-ø	b w<br>y ø	b w y<br>ø
*t-	t-	t- č-	t-	t-	t-	t-	t-	t-	t-	t-	t-	t-	t-
-t-<br>-t	t	-d-<br>-t	t	-d-<br>-t	t	t	t	t	t	t	t	t	t
*y-	d-	y-	d-	y-	y-	y-	y-	y-	y-	y-	y-	y-	y-
-d-<br>-d	d	y r<br>-ø	d	y	y	y	y	y	y	y	y	y	y
*k(A)-	q-	x-	q-	q-	q-	q-	q-	q-	q-	q-	k-	q-	k-
*k(E)	k-	k-	k-	k-	k- g-	k-	k-	k-	k-	k-	k-	k-	k-
-k(A)-<br>-k	q	-g-<br>-k -ø	q	-ɣ-<br>-q	-ɣ-<br>-q	-ɣ-<br>-q	-ɣ-<br>-q	-ɣ-<br>-q	-ɣ-<br>-q	-ɣ-<br>-q	-ɣ-<br>-q	-ɣ-<br>-q	-ɣ-<br>-q
-k(E)-<br>-k(E)	k	-g-<br>-k -ø	k	-g-<br>-k	-g-<br>-k	-g-<br>-k	-g-<br>-k	-g-<br>-k	-g-<br>-k	-g-<br>-k	-g-<br>-k	-g-<br>-k	-g-<br>-k
*-g(A)-	ɣ	ø	ø	w y	w y	w y	g w	w y	w y	w y	w y	w y	w y

-g(A)<br>-g				-ø -<br>w -y	-ø -w<br>-y	-ø -w<br>-y	y ø	-ø -<br>w -y	-ø -<br>w -y	-ø -<br>w -y	-ø -w -<br>y	-ø -w<br>-y	-ø -w -<br>y
-g(E)-<br>-g(E)<br>*-g	g	v y	ɣ w<br>y	w y<br>-ø -<br>w -y	w y<br>-ø -w<br>-y	w y<br>-ø -w<br>-y	g w<br>y ø	w y<br>-ø -<br>w -y	w y<br>-ø -<br>w -y	w y<br>-ø -<br>w -y	w y<br>-ø -w -<br>y	w y<br>-ø -w<br>-y	w y<br>-ø -w -<br>y
-č-<br>-č	č	-ź-<br>-ś	č	j č<br>-č	č	č	j<br>-č	č	s	š	č	š	š
*-lč	-š	-ś	-š	-š	-š	-š	-š	-š	-š	-s	-š	-s	-s
*s-	s-	s- š-	s-	s-	s-	s-	s-	s-	h-	s-	s-	s-	s-
-s-<br>-s	s	z ž<br>-s -š	s	s	s	s	s	s	θ	s	s	s	s
-m-<br>-m-	m<br>-n<br>-m	m<br>-n<br>-m	m<br>-n<br>-m	m<br>-n<br>-m	m<br>-n<br>-m	m<br>-n -m	m<br>-n<br>-m	m<br>-n<br>-m	m<br>-n<br>-m	m<br>-n<br>-m	m<br>-n<br>-m	m<br>-n<br>-m	m<br>-n<br>-m
-n-<br>-n	n	n m	n	n	n	n	n	n	n	n	n	n	n
*-r-	r	r	r	r	r	r	r	r	r	r	r	r	r
*-r_2-	z	r	z	z	z	z	z	z	δ	z	z	z	z
*-l-	l	l	l	l	l	l	l	l	l	l	l	l	l

PTk	Tk.	Az.	Gag.	Tkm.	Uz.	Uigh.	Tuv.	Tofa	Shor	Khak.	Yak.	Dolg.
-p-<br>-p	b p<br>-p	b p<br>-p	b p<br>-p	b p<br>-p	v p b<br>-v -p<br>-b	p<br>-p	-v-<br>-p	b p<br>-p	b<br>-p	b<br>-p	b<br>-p	b<br>-p
*b-	b-	b-	b-	b-	b-	b-	b-	b-	p-	p-	b-	b-
-b-<br>-b	v y<br>ø	v y ø	v ø	w y ø	v g y<br>ø	b v g<br>y ø	v ø<br>-g	b ø<br>-g	b ø<br>-g	b ø<br>-g	b ø	b ø
*t-	t-<br>d-	t- d-	t- d-	t- d-	t-	t-	t- d-	t- d-	t-	t-	t-	t-
-t-<br>-t	t d	t d	t d	-t- -d-<br>-t	t	t	-d-<br>-t	-d-<br>-t	-d-<br>-t	-d-<br>-t	t	t
*y-	y-	y-	y-	y-	y-	y-	d-<br>t-	d-	z-	z-<br>s-	t-	t-
-d-<br>-d	y	y	y	y	y	y	-d-<br>-t	d	z	-z-<br>-s	t	t
*k(A)-	k-<br>g-	k- g-	k- g-	k- g-	q-	q-	q- x-	q-	q-	x-	q- x-	k-
*k(E)-	k-<br>g-	ġ-	k-	ġ-	k-	k-	k-	k-	k-	k-	k-	k-
-k(A)-<br>-k(A)	-ɣ-<br>-q	-ɣ-<br>-q	-k-<br>-q	-k-<br>-q	q	q	-g-<br>-q	-g-<br>-q	-ɣ-<br>-q	-ɣ-<br>-q	-ɣ-<br>-x	-g-<br>-k
-k(E)-<br>-k(E)	-g-<br>-k	-g-<br>-k	-g-<br>-k	-g-<br>-k	k	k	-g-<br>-k	-h-<br>-k	-g-<br>-k	-g-<br>-k	-g-<br>-k	-g-<br>-k
-g(A)-<br>-g	ɣ w<br>y ø	ɣ w y<br>ø	ɣ w y<br>ø	ɣ w y<br>ø	-ɣ-<br>-q	-ɣ-<br>-q	ɣ ø	ɣ ø	ɣ ø	ɣ ø	ø	ø
-g(E)-<br>-g	ɣ w<br>y ø	ɣ w y<br>ø	ɣ w y<br>ø	ɣ w y<br>ø	-g-<br>-k	-g-<br>-k	g ø	g ø	g ø	g ø	ø	ø
-č-<br>-č	č j<br>-č	č j<br>-č	č j<br>-č	č j<br>-č	č	č	-ž-<br>-š	-j-<br>-š	č<br>-š	č<br>-s	-h-<br>-s	č<br>-s
*-lč	-š	-š	-š	-š	-š	-š	-š	-š	-š	-s	-s	-s
*s-	s-	s-	s-	s-	s-	s-	s-	s-	s-	s-	ø-	ø-
-s-<br>-s	s	s	s	s	s	s	-z-<br>-s	s	-z-<br>-s	-z-<br>-s	t	t
-m-<br>-m	m<br>-n<br>-m	m<br>-n<br>-m	m<br>-n<br>-m	m<br>-n<br>-m	m<br>-n<br>-m	m<br>-n<br>-m	m<br>-n<br>-m	m<br>-n<br>-m	m<br>-n<br>-m	m<br>-n<br>-m	m<br>-n<br>-m	m<br>-n<br>-m

-n-<br>-n	n	n	n	n	n	n	n	n	n	n	n	n
*-r-	r	r	r	r	r	r	r	r	r	r	r	r
*-r_2-	z	z	z	z	z	z	s	s	s	s	s	s
*-l-	l	l	l	l	l	l	l	l	l	l	l	l

Table 3.10 Reconstruction of the basic vowel inventory of Proto-Turkic

PTk	OT	Chu.	Kh.	CTat	Kum.	KBal.	Krm.	Tat.	Bash.	Kaz.	Kirg.	Kpak	Nog.
*a	a	u	a	a	a	a	a	a	a	a	a	a	a
*e	e	i	ä e	e	e	e	e	i	i	e	e	e	e
*o	o	u	o	o	o	o	o	u	o	o	o	o	o
*ö	ö	ü ă	e ö	ö o	ö	ö	ö	ü	ü	ö	ö	ö	ö
*u	u	vă-<br>-ă-	u	u	u	u	u	o	o	ŭ	u	u	u
*ü	ü	ă	ü i	ü u	ü	ü	ü	ö	ö	ü	ü	ü	ü
*ï	ï	ă ĕ	ï	ï i	ï	ï	ï	ï	ï	ï	ï	ï	ï
*i	i	ă ĕ	i	i	i	i	i	e	e	ĭ	i	i	i
*ia	a	yu	a	a	a	a	a	a	a	a	a	a	a

PTk	Tk.	Az.	Gag.	Tkm.	Uz.	Uigh.	Tuv.	Tofa	Shor	Khak.	Yak.	Dolg.
*a	a	a	a	a	a e	ɔ a ä	a	a	a	a	a	a
*e	e	ä e	e	e	e	ä e	e	e	e	i	i	i
*o	o	u	o	o	ọ	o	o	o	o	o	o	o
*ö	ö	ö	ö	ö	ọ	ö	ö	ö	ö	ö	ö	ö
*u	u	u	u	u	u	u	u	u	u	u	u	u
*ü	ü	ü	ü	ü	u	ü	ü	ü	ü	ü	ü	ü
*ï	ï	ï i	ï	ï	i	i	ï	ï	ï	ï	ï	ï
*i	i	i	i	i	i	i	i	i	i	ɘ	i	i
*ia	a	a	a	a	a e	ɔ	a	a	a	a	a	a

Table 3.11 Consonant correspondences between the Transeurasian languages

	pJ	pK	pTg	pMo	pTk	pTEA
1.	*p-	*p-	*p-	*p-	*b-/ *p-	*p-
2	*-p-	*-p-	*-p-	*-ɣ-	*-p-	*-p-
3.	*p- / *w-	*p-	*b-	*b-	*b-	*b-
4.	*-p-/*-w-	*-p-	*-b-	*-b-/ -ɣ-	*-b-	*-b-
5.	*-np-	*-pC-	*-PC-	*-PC-	*-P(C)-	*-m$^{(P)}$T-
6.	*-np-	*-Rp-	-RP-	*-RP-	*-RP-	*-Rp-
7.	*t-	*t-	*t-	*t-	*t-	*t-
8.	*-t-	*-t-	*-t-	*-t-	*-t-	*-t-
9.	*t- /*y-	*t- (ci-)	*d- (ji-)	*d- (ji-)	*y-	*d-
10.	*-t-/ *-y-	*-l-	*-d- (-ji-)	*-d- (-ji-)	*-d-	*-d-
11.	*-nt-	*-c-	*-TC-	*-TC-	*-TC-	*-n$^{(T)}$K-
12.	*-nt-	*-Rc-	*-RT-	*-RT-	*-RT-	*-Rt-
13.	*k-	*k-	*k-	*k-	*k-	*k-
14.	*-k-	*-k- (-h-)	*-k-	*-k-	*-k-	*-k-
15.	*k-	*k-	*g-	*g-	*k-	*g-
16.	*-k-	*-k- (-h-)	*-g-	*-g-	*-g-	*-g-
17.	*-nk-	*-kC-	*-KC-	*-KC-	*-KC-	*-ŋ$^{(K)}$T-
18.	*-nk-	*-Rk-	*-RK-	*-RK-	*-RK-	*-Rk-

19.	*t-	*c-	*č-	*č-	*č-	*č-
20.	*-t-	*-c-	*-č-	*-č-	*-č-	*-č-
20b.	*-si	*-l(i)/ -c	*-l(č)	*-l(č)	*-l(č)~ -š	*-lč
21.	*k-	*k-, h-	*x-	*k-	*k-	*x-
22.	*-k-	*-k-	*-x-	*-g-~-k-	*-g-~-k-	*-x-
23.	*s-	*s-	*s-	*s-	*s-	*s-
24.	*-s-	*-s-	*-s-	*-s-	*-s-	*-s-
25.	*m-	*m-	*m-	*m-	*b-	*m-
26.	*-m-	*-m-	*-m-	*-m-	*-m-	*-m-
27.	*n-	*n-	*n-	*n-	*y-	*n-
28.	*-n-	*-n-	*-n-	*-n-	*-n-	*-n-
29.	*-r-	*-l-	*-r-	*-r-	*-r-	*-r-
30.	*-r-	*-l-	*-r-	*-r-	*-r_2-	*-r-
31.	*-r-	*-l-	*-l-	*-l-	*-l-	*-l-

Table 3.12 Vowel correspondences between the Transeurasian languages

	OJ < pJ	MK < pK	pTg	pMo	pTk	pTEA
32.	-a- < *-a-	-a- < *-a-	*-a-	*-a-	*-a-	*-a-
32b.	*CaCa	*CʌCʌ	*CaCa	*CaCa	*CaC	*CaCa
33.	-a- < *-a-	-e- < *-e-	*-e-	*-e-	*-e-	*-ə-
34.	-o- < *-ə-	-e- < *-e-	*-e-	*-e-	*-e-	*-ə-
35.	-o- <? *-o-	-wo- < *-o-	*-o-	*-o-	*-o-	*-ɔ-
36.	-u- < *-o-	-wo- < *-o-	*-o-	*-o-	*-o-	*-ɔ-
37.	-o- < *-ɨ-	-u- < *-ɨ-	*-ö-	*-ö-	*-ö-	*-o-
38.	-u- < *-u-	-wu- < *-u-	*-u- (gü)	*-ü-	*-ü-	*-u-
39.	-u- < *-u-	-o- < *-ʌ-	*-u-	*-u-	*-u- /-ï-	*-ʊ-
39b.	PaRu- < *PauRu-	*PʌRʌ- ~ *PɨRɨ-	*PuRu-	*PuRu-	*PuR-	*PʊRʊ-
40.	-i- < *-i-	-i- < *-i-	*-i-	*-i-	*-i-/-ï-	*-i-
40b	-i- < *-e-	-ye- < *-ia-	*-ia-	*-ia-	*-ia-	*-ia-
40c	-e$_{(1)}$ - < *-e-	-ye- < *-ia-	*-ia-	*-ia-	*-ia-	*-ia-
40 d	-o- < *-ə-	-e- < *-ə-	*-ü-	*-ö-	*-ö-	*-iu-
41.	a- < *-a-	a- < *a-	*a-	*a-	*a-	*a-
41b.	a- < *-a-	e- < *e-	*e-	*e-	*e-	*ə-
42.	o- < *ə-	e- < *e-	*e-	*e-	*e-	*ə-
43.	o- <? *o-	wo- < *o-	*o-	*o-	*o-	*ɔ-
44.	o- < *ɨ-	ø < ? *ɨ-	*ö-	*ö-	*ö-	*o-
45.	u- < *u-	wu- <	*u-	*u-	*u-	*u-

		*u-				
46.	i- < *i-	i- < *i-	*i-	*i-	*i-	*i-
46b	i- < *e-	ye- < *ia-	*ia-	*ya-	*ya-	*ia-
46c	ya- < *ia-	ye- < *ia-	*ia-	*ya-	*ya-	*ia-
46d	o- < *-ə-	ye- <*iə	*ü-	*ö-	*ö-	*iu-

Abbreviations

Ama.	Amami
Az.	Azerbaijani
Bao.	Bao'an
Bash.	Bashkir
Bur.	Buriat
Chu.	Chuvash
Dag.	Dagur
Dlg.	Dolgan
Dong.	Dongxiang
EYugh.	Eastern Yughur
Even	Even
Evk.	Evenki
Gag.	Gagauz
J	Japanese
Jur.	Jurchen
Kalm.	Kalmuk
KBalk.	Karachay-Balkar
Krm.	Karaim
Kkp.	Karakalpak
Karakh.	Karakhanide
Kaz.	Kazakh
Khak.	Khakas
Khal.	Khalkha
Khalaj	Khalaj
Kir.	Kirgiz
K	Korean
Kum.	Kumyk
Ma.	Manchu
MK	Middle Korean
MMo.	Middle Mongolian
Miy.	Miyako
Mogh.	Moghol
Mgr.	Monguor
MT	Middle Turkic

Na.	Nanai
Neg.	Negidal
Nog.	Nogai
Oki.	Okinawa
Olcha	Olcha
OJ	Old Japanese
OT	Old Turkic
Ord.	Ordos
Oroch	Oroch
pJ	proto-Japonic
pK	proto-Koreanic
pMo	proto-Mongolic
pTEA	proto-Transeurasian
pTg	proto-Tungusic
pTk	proto-Turkic
Shor	Shor
Sibe	Sibe
Sol	Solon
Tat.	Tatar
Tofa.	Tofalar
Tk.	Turkish
Tkm.	Turkmen
Tuva	Tuva
Ud.	Udehe
Uigh.	Uighur
Uz.	Uzbek
WMo.	Written Mongolian
Yae.	Yaeyama
Yak.	Yakut
Yon.	Yonaguni

References mentioned in Supplementary Information 2

Antonov, A. *Le rôle des suffixes nominaux en /+rV/ dans l'expression du lieu et de la direction en japonais et l'hypothèse de leur origine "altaïque"* (Institut National des Langues et Civilisations Orientales Ph.D. dissertation, 2007).

Beckwith, C. *Koguryo. The language of Japan's continental relatives* (Brill, 2007).

Bentley, J. The verb *toru* in Old Japanese. *Journal of East Asian Linguistics* **8(2)**, 131-146 (1999).

Benzing, J. Die tungusischen Sprachen. Versuch einer vergleichenden Grammatik. *Abhandlungen der geistes- und sozialwissenschaftlichen Klasse* **11**, 949-1099 (1955a).

Benzing, J. *Lamutische Grammatik, mit Bibliographie, Sprachproben und Glossar* (Otto Harrassowitz, 1955b).

Cincius, V. I. (ed) *Sravnitel'nyj slovar' tunguso-man'čžurskich jazykov. Vol. 1-2* (Nauka, 1975-1977).

De Smedt, A. & Mostaert, A. *Le dialecte monguor, II: grammaire* (Uralic and Altaic Series 30.) (Mouton, 1964).

Doerfer, G. *Türkische und mongolische Elemente im Neupersischen, unter besonderer Berücksichtigung älterer neupersischer Geschichtsquellen, vor allem der Mongolen- und Timuridenzeit. Vol. 1-4* (Franz Steiner, 1963-1965-1967-1975).

Doerfer, G. The problem of Rhotacism / Zetacism. *Central Asiatic Journal* **28**, 36-42 (1984).

Erdal, M. *Old Turkic word formation. A functional approach to the lexicon* (Turcologica 7.) (Harrassowitz, 1991).

Frellesvig, B. & Whitman, J. in *Proto-Japanese: Issues and prospects* (Current Issues in Linguistic Theory 249) (eds Frellesvig, B. & Whitman, J.) 15–41 (Benjamins, 2008).

Georg, S. Review of Robbeets, Martine 2005. Is Japanese related to Korean, Tungusic, Mongolic and Turkic? *Korean Studies* **31**, 247-278 (2007).

Gorelova, L. M. *Manchu grammar* (Brill, 2002).

Haspelmath, M. & Tadmor, U. *Loanwords in the World's Languages: A Comparative Handbook* (Mouton de Gruyter, 2009).

Heggarty, P. & Anderson, C. (eds). n.d. *Cognacy in Basic Lexicon (CoBL)* (Max Planck Institute for the Science of Human History, 2015–).

Heine, B. & Kuteva, T. *World lexicon of grammaticalization* (Cambridge Univ. Press, 2002).

Kara, G. Review of Martine Robbeets, 2005, *Is Japanese related to Korean, Tungusic, Mongolic and Turkic?* Wiesbaden: Harrassowitz. *Anthropological Linguistics* **49,** 95–98 (2007).

Lee, K.-M. *Geschichte der Koreanischen Sprache* (Dr. Ludwig Reichert Verlag, 1977).

Martin, S. E. *The Japanese language through time* (Yale Univ. Press, 1987).

Martin, S. E. *A reference grammar of Korean* (Tuttle, 1992).

Martin, S. E. *Consonant lenition in Korean and the Macro-Altaic question* (University of Hawai'i Press, 1996).

Miyake, M. H. *The phonology of eighth century Japanese revisited: Another reconstruction based upon written records* (University of Hawai'i at Manoa Ph.D. dissertation, 1999).

Mudrak, O. A. in *Lingvističeskaja rekonstrukcija i drevnejšaja istorija Vostoka* [Linguistic Reconstruction and the Ancient History of the East] (eds Kullanda, S. V. et al.) 216-222 (Nauka, 1989).

Nedjalkov, Igor V. 1997. *Evenki (Descriptive Grammars)*. London: Routledge.

Nugteren, Hans. 2011. Mongolic phonology and the Qinghai-Gansu languages. Utrecht: LOT. Leiden University Ph.D. Dissertation.

Poppe, Nicholas. 1954. *Grammar of written Mongolian*. Wiesbaden: Otto Harrassowitz.

Poppe, Nicholas. 1955. *Introduction to Mongolian comparative studies* (Mémoires de la société Finno-Ougrienne 110). Helsinki: Suomalais-Ugrilainen Seura.

Poppe, Nicholas. 1973. Über die Bildungssuffixe der mongolischen Bezeichnungen der Körperteile. *Ural-Altaische Jahrbücher* 45: 223-243.

Ramsey, Samuel Robert. 1977. S-clusters and reinforced Consonants. In Kim, Chin-W. (ed.), *Papers in Korean Linguistics*, 59–66. Columbia: Hornbeam Press.

Ramstedt, Gustaf John. 1912. Zur Verbalstammbildungslehre der mongolish-türkischen Sprachen. *Journal de la Société finno-ougrienne* 28: 1–86.

Rhee, Seongha. 1996. *Semantics of verbs and grammaticalization: The development in Korean from a cross-linguistic perspective*. Austin: University of Texas Ph.D. dissertation.

Robbeets, M. Is Japanese related to Korean, Tungusic, Mongolic and Turkic? (Turcologica 64.) (Harrassowitz, 2005).

Robbeets, M. Diachrony of Verb Morphology: Japanese and the Transeurasian languages. (Trends in Linguistics Studies and Monographs 291.) (Mouton-De Gruyter, 2015).

Robbeets, M. Proto-Transeurasian: where and when? *Man in India* **95**, 921-946 (2017).

Róna-Tas András & Berta, Á. (eds.). *West Old Turkic. Turkic Loanwords in Hungarian* (Turcologica 84.) (Harrassowitz, 2011).

Rozycki, W. *Mongol elements in Manchu* (Uralic and Altaic Series 157) (Bloomington, 1994).

Russell, K. L. *A reconstruction and morphophonemic analysis of proto-Japonic verbal morphology* (University of Hawai'i Ph.D. dissertation, 2006).

Sapir, E. A study of phonetic symbolism. *Journal of Experimental Phonology* **12**, 225–239 (1929).

Savelyev, A. Staročuvašskij pamjatnik s različenijem dativa i akkuzativa [A pre-Standard Chuvash text with a dative-accusative distinction]. *Ural-Altaic Studies* 1(**40**), (in press, 2021).

Starostin, S., Dybo, A. & Mudrak, O. *Etymological dictionary of the Altaic languages* (Brill, 2003).

Tekin, T. Zetacism and Sigmatism: Main pillars of the Altaic theory. *Central Asiatic Journa*l **30**, 141-160 (1986).

Thorpe, M. L. *Ryukyuan language history* (University of Southern California Ph.D. dissertation, 1983).

Unger, J. M. Reconciling comparative and internal reconstruction: The case of Old Japanese /*ti, ri, ni*/. *Language* **76**, 655–681 (2000).

Vovin, A. 2008. *Koreo-Japonica: A re-evaluation of a common genetic origin* (Center for Korean Studies Monograph) (University of Hawai'i Press, 2008).

Vovin, A. *A descriptive and comparative grammar of Western Old Japanese, part 2: adjectives, verbs, adverbs, conjunctions, particles, postpositions* (Languages of Asia 8) (Global Oriental, 2009).

Vovin, A. First and second person singular pronouns: a pillar or a pillory of the 'Altaic' hypothesis? *Türk Dilleri Araštïrmalari* **21(2)**, 251-278 (2013).

Whitman, J. B. *The phonological basis for the comparison of Japanese and Korean* (Harvard University Ph.D. dissertation, 1985).

Yanagida, Y. & Whitman, J. Alignment and word order in Old Japanese. *Journal of East Asian Linguistics* **18(2)**,101–144 (2009).
