# Supplementary material for: Triangulation supports agricultural spread of the Transeurasian languages
Source: Nature. 2021 Nov 10;599(7886):616–21. doi: 10.1038/s41586-021-04108-8 (PMC8612925; doi:10.1038/s41586-021-04108-8)
Supplement: Supplementary file 3 — This zipped file contains Supplementary Data Files 1, 2 and 4–6; see Supplementary Information file for full descriptions (Supplementary Data File 3 is hosted externally; see Supplementary Information file for links). [file 41586_2021_4108_MOESM3_ESM.zip › 2021-02-02920E-s3/19_Eurasia3angle_synthesis_SI 4_homelands_REV01.10.pdf]

Supplementary Information 4
Integration of classical assessment methods and Bayesian phylogeography in identifying the ancestral homelands of Transeurasian

1. The Proto-Transeurasian homeland in the Neolithic

Location method	Evidence	Source	Estimated location	Lat. \ Long.
**Diversity hotspot principle**	Greatest linguistic diversity with regard to deepest subgroups (Koreanic, Tungusic, Mongolic, Turkic)	Robbeets 2020	Inner Mongolia, West Liao region	
**Cultural reconstruction**	9181 BP (5595 -12793 95% HPD) cultivation, millets, spades, vegetal fermentation, durable wild food resources, sedentism, textile production	Robbeets 2020 SI 4	Inner Mongolia, West Liao region	
**Bayesian phylogeography**	Basic vocabulary cognates	SI 2	Gobi desert (Inner Mongolia/Mongolia border)	43,04 110,29

Diversity hotspot principle. Below we estimate the location of the subfamilies which existed from the Neolithic onwards, i.e., Proto-Altaic, Proto-Japano-Koreanic, Proto-Turkic and Proto- Mongolo-Tungusic. As the West Liao River Region is in the core of the relatively compact territory where these proto-languages were once spoken, it would seem a plausible source area for their spread. The identification of the West Liao River Region as the core of the original spread zone is corroborated by the observation that it is today still here that the greatest linguistic diversity with regard to the deepest subgroups of Transeurasian is found; linguistic pockets of Korean, Tungusic, Mongolic and Turkic languages are represented in the area. Moreover, the area also lies in the center of ancient linguistic diversity, as the Khitan language (Macro-Mongolic, Liao Empire, 907–1125), the Jurchen language (Tungusic, Jin empire AD 1115–1234) and the Koguryo language (Macro-Japonic, Puyo state, 300 BC–AD 346) were once spoken in this area.

Cultural reconstruction. Combining the root age of Proto-Transeurasian estimated at 9181 BP (5595 -12793 95% HPD) with the nature of the vocabulary reconstructed in the ancestral language, we are looking for Early Neolithic populations that were sedentary, engaged in at least some small-scale cultivation, subsisted on millets and durable wild food resources, used harvesting tools and were familiar with textile production such as weaving. Across North and East Asia, only the Xiaohexi (before 8200 BP), Xinglongwa (8200–7400 BP) and Zhaobaogou (7400–6500 BP) cultures, situated in the West Liao River Region in Inner Mongolia between the 10th and 7th millennia BP, answer to these restrictions.

Bayesian phylogeography. Even if there is a large uncertainty in root location of our Bayesian approach,we infer a central point of origin in the Gobi desert on the border between present-day Inner Mongolia and Mongolia. Yang et al. (2015) demonstrated that this area was used as farmland before it became a desert ca. 4200 years ago through groundwater capture by the Xilamulun River. Integrating this location with those inferred by the traditional assessment methods, we propose a location for the Transeurasian homeland as indicated in Fig. SI 4.10.

Figure SI 4.1 The Proto-Transeurasian homeland identified on the basis of the diversity hotspot principle, cultural reconstruction and Bayesian phylogeography[1]

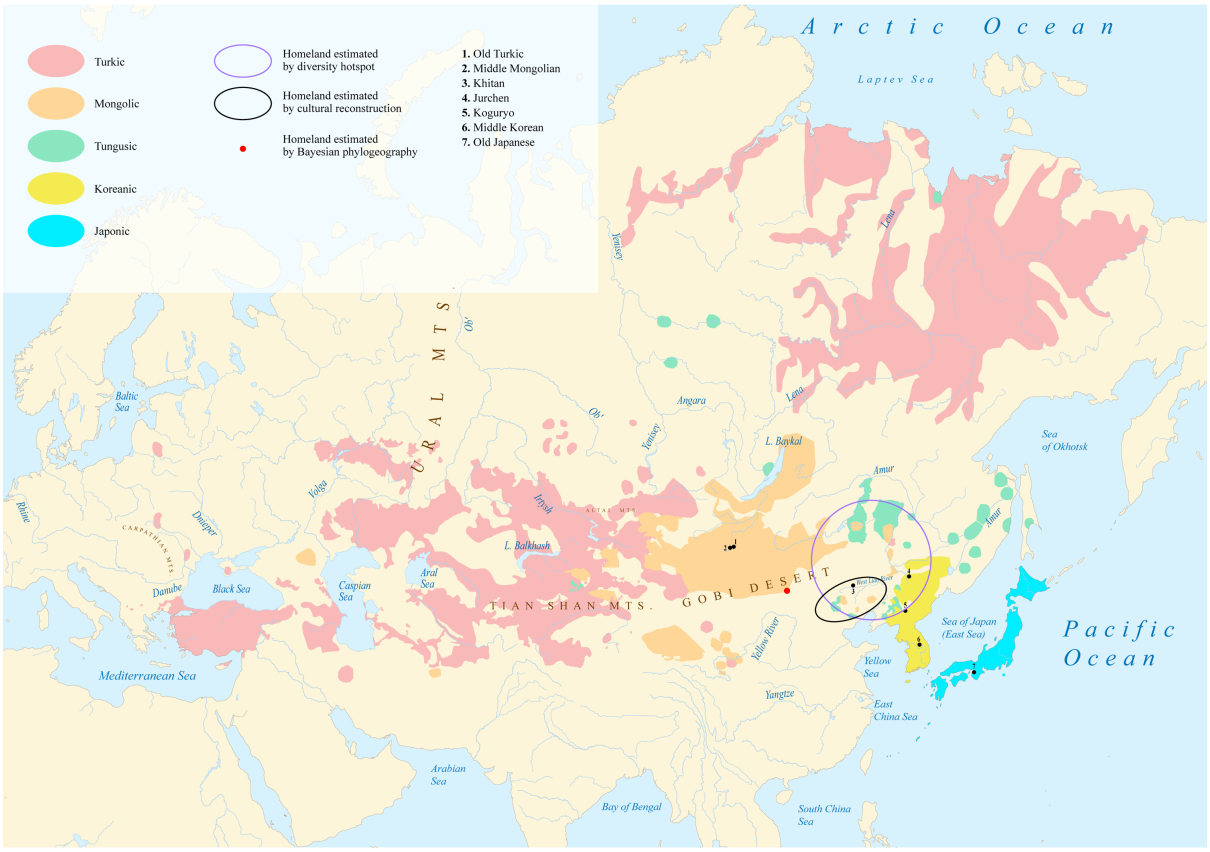

2. The Proto-Japano-Koreanic homeland in the Neolithic

Location method	Evidence	Source	Estimated location	Lat. \ Long.
**Diversity hotspot principle**	greatest linguistic diversity with regard to deepest subgroups present around 300 AD (Koguryo, Silla, Paekche, Kaya, pre-Old Japanese)	Hudson & Robbeets 2020	Mid-West Korea	
**Cultural reconstruction**	5458 BP (3335-8024 95%HPD)	Robbeets 2020 Whitman 2011	Liaodong Peninsula and Bohai coast	

	Maritime vocabulary, cultivation, harvesting, millets (no rice), agricultural tools, fermentation, pottery, durable wild food resources, textile production, hemp	Unger 2014<br>Francis-Ratte 2016<br>SI 4		
**Bayesian phylogeography**	Basic vocabulary cognates	SI 2	Mid-West Korea	36,30<br>126,15

Diversity hotspot principle. Since Japanese today is largely confined to the Japanese Islands, we cannot identify the diversity hotspot for contemporary Japanese and Korean. However, as it is a documented fact that two language families, Koreanic and Macro-Japonic, were once simultaneously present on the Korean peninsula, we can estimate the ancient diversity hotspot. The coexistence of Koreanic and Macro-Japonic on the Korean penisula was discontinued when Macro-Japonic relocated to the Japanese Islands in the first millennium BC and when the Koreanic expansion unified the languages of the Korean Peninsula from the seventh century AD onwards.

We do not have any historical information about the languages spoken on the Korean peninsula until the third century AD, when Chinese dynastic chronicles start to leave some vague records of languages spoken at that time and how they related to each other. According to these sources, the local inhabitants were divided into at least two ethno-linguistic groups: the Puyŏ and the Han. The Puyŏ were scattered over the Liaodong peninsula and the northern half of the Korean peninsula and included four groups, the Puyŏ proper, Koguryŏ, Okchŏ and Ye. Their language was probably more closely related to Japanese than to Korean (Beckwith 2007). The Han consisted of three related groups of people in the southern part of the Korean peninsula, the Mahan in the west, the Pyŏnhan in the Naktong River valley in the centre and the Chinhan in the east. In the Three Kingdoms period (AD 300–668), these groups respectively became the kingdoms Paekche, Kaya, and Silla, each with their individual languages. The Han languages are usually associated with various Koreanic languages (Lee and Ramsey 2011).

Nevertheless, as indicated by the green dots in Figure SI 3.2, there must have been pockets of Macro-Japonic speech communities among the Koreanic languages. Chinese chronicles, such as the *Hou Han Shu* (the fifth century AD 'History of the Later Han') state that the Pyŏnhan people were close to the Wa, the ethnonym for inhabitants of the Japanese Islands. Linguistically, the alleged presence of Macro-Japonic languages in Korea is confirmed by the historical Macro-Japonic toponyms, documented especially in the Mahan and Pyŏnhan regions (Bentley 1998, 2000) and by a small number of words in the *Nihon shoki* which might be of Kaya origin (Kōno 1987). Therefore, it seems likely that there were at least some Macro-Japonic languages among the Pyŏnhan and Mahan languages. Figure SI 3.2 marks the center of linguistic diversity between Puyŏ (Macro-Japonic) and Han (Koreanic) languages in purple.

Cultural reconstruction. The cultural vocabulary of Proto-Japano-Koreanic includes maritime vocabulary, words for cultivation, harvesting, agricultural tools, fermentation, pottery, millets, durable wild food resources and weaving terminology.

In addition to the reconstruction for 'ocean, sea' and 'boat', we find terms for 'crab, *Portunus trituberculatus*' and 'swellfish, *Takifugu chinensis*'. These species inhabit the

marine waters around China, Korea, and Japan, but are most prevalent in the Bohai Sea. Given the original situation of the Transeurasian homeland in the West Liao River basin, a movement to the coastal area of the Bohai Sea would be conceivable. This location of the Proto-Japano-Koreanic homeland alligns with that proposed by Unger (2014: 224) and Francis-Ratte (2016: 472–473).

Moreover, our Bayesian time estimation at 5458 BP (3335-8024 95%HPD) in combination with the reconstruction of Proto-Japano-Koreanic vocabulary dedicated to weaving is in line with archaeological finds of ceramic spindle whorls dating back to the Neolithic within the cultures on the Liaodong Peninsula, such as the Houwa (6350–4900 BP), Xiaozhushan (6000–4000 BP) and Shuangtuozi (5500-3400 BP) cultures (Supplementary Table 5: sites 49-71; Nelson et al. 2020). These cultures belonged to the same cultural system and were familiar with millet agriculture.

Given the lack of rice-vocabulary in Proto-Japono-Koreanic we can infer that the speech community must have been situated to the north of the cultures on the Yellow River and the Shandong Peninsula that were familiar with both millet and rice agriculture at that time. The original presence of Macro-Japonic in the Liaodong-Shandong interaction sphere indicates the location of the Proto-Japano-Koreanic homeland on the Liaodong Peninsula as the most parsimonious hypothesis.

Bayesian phylogeography. In line with the diversity hotspot principle, we infer a central point of origin in Mid-West Korea. Integrating this location with the one inferred through cultural reconstruction, we propose a location for the Proto-Japano-Koreanic homeland as indicated in Fig. SI 4.10.

Figure SI 4.2 The Proto-Japano-Koreanic homeland identified on the basis of the diversity hotspot principle, cultural reconstruction and Bayesian phylogeography

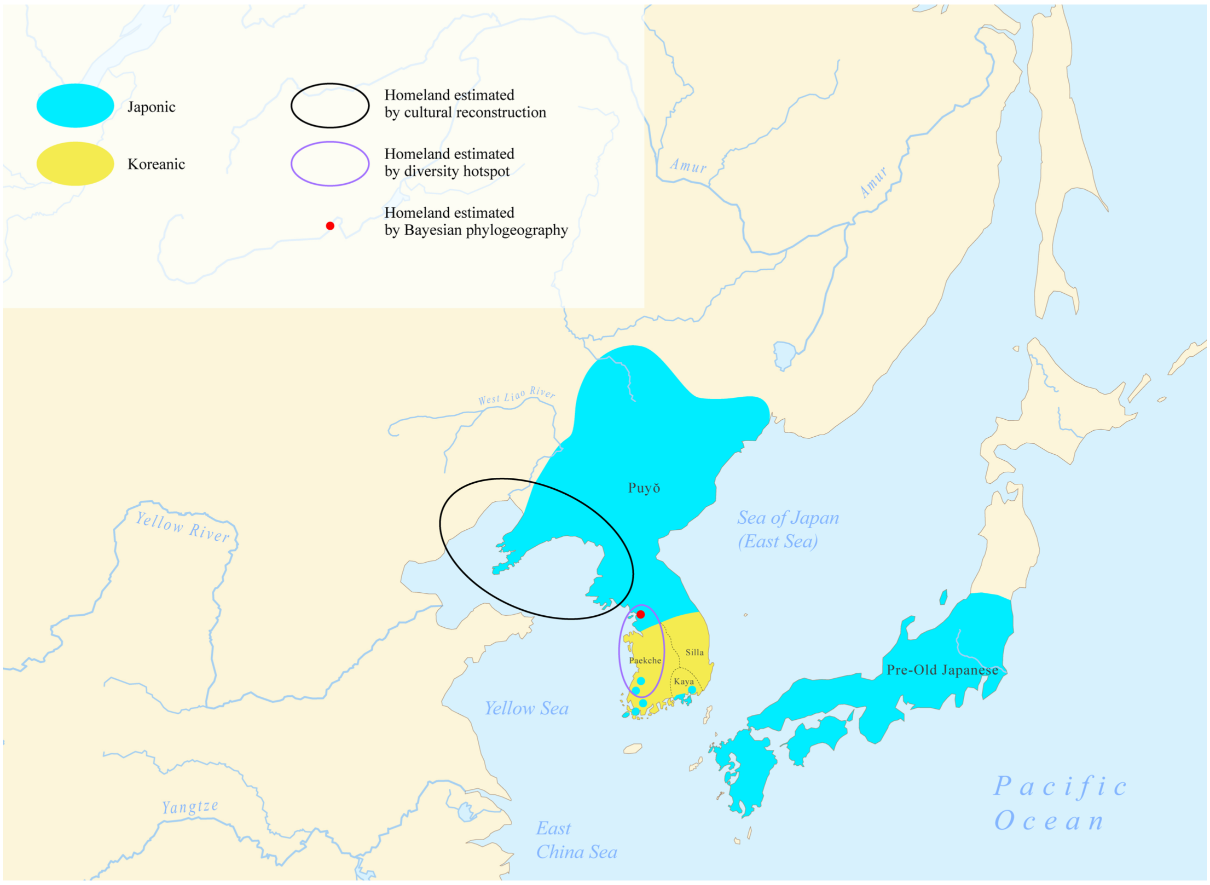

3. The Proto-Altaic homeland in the Neolithic

Location method	Evidence	Source	Estimated location	Lat. \ Long.
**Diversity hotspot principle**	greatest linguistic diversity with regard to deepest subgroups (Tungusic, Mongolic, Turkic)		Inner Mongolia, West Liao region	
**Cultural reconstruction**	6811 BP (4404-10166 95%HPD) cultivation, millets, agricultural tools, fermentation, durable wild food resources, pig husbandry (no dairying, no pastoralism), textile production	Robbeets 2020c SI 4	Inner Mongolia, West Liao region	
**Bayesian phylogeography**	Basic vocabulary cognates	SI 3	Northeastern Altai mountains	48,62 97, 13

Diversity hotspot principle. The West Liao River region is in the center of the spread zone of ancient written varieties such as Orkhon Old Turkic (AD 700), Middle Mongolian (AD 1200), Khitan (Macro-Mongolic) and Jurchen (Tungusic) and it is also the region representing the greatest linguistic diversity with regard to the deepest subgroups of contemporary varieties of Altaic, notably pockets of Tungusic, Mongolic and Turkic languages are present in the area.

Cultural reconstruction. Given the maritime character of Proto-Japono-Koreanic vocabulary as opposed to the vocabulary reconstructed for Proto-Altaic and its predecessor Proto-Transeurasian, it would be plausible to assume a break-away model, whereby there was geographical continuity between the Transeurasian and Altaic speech communities in the West Liao River region, while the Japano-Koreanic speakers left and settled in coastal areas after their separation. The Proto-Altaic vocabulary of subsistence is largely continuous with that of Proto-Transeurasian, except for the addition of terms relating to pig husbandry. There are no indications of cattle keeping, dairying or pastoralism in the vocabulary.

Bayesian phylogeography. We infer a central point of origin in the Northeastern Altai mountains. Integrating this location with the one inferred through the traditional assessment methods, we propose a location for the Proto-Altaic homeland as indicated in Fig. SI 4.10.

Figure SI 4.3 The Altaic homeland identified on the basis of the diversity hotspot principle, cultural reconstruction and Bayesian phylogeography

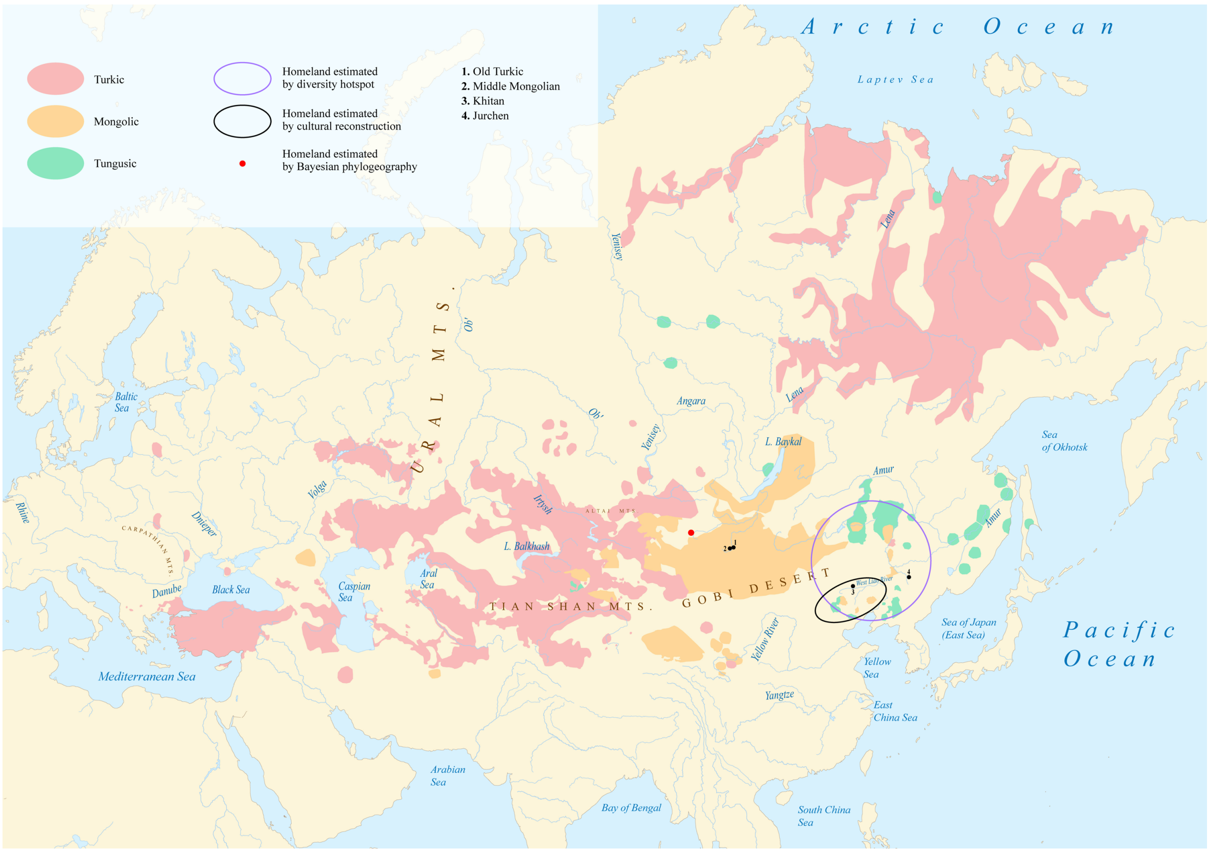

4. The Proto-Mongolo-Tungusic homeland in the Neolithic

**Location method**	**Evidence**	**Source**	**Estimated location**	**Lat. \ Long.**
**Diversity hotspot principle**	greatest linguistic diversity with regard to deepest subgroups (Tungusic, Mongolic)		Inner Mongolia, West Liao region	
**Cultural reconstruction**	4491 BP (2599-6373 95%HPD) cultivation, millets (no rice), agricultural tools, fermentation, durable wild food resources, pig husbandry (no dairying, no pastoralism), textile production	Robbeets 2020c SI 4	Inner Mongolia, West Liao region	
**Bayesian phylogeography**	Basic vocabulary cognates	SI 3	Northeastern Mongolia	47,47 114,30

Diversity hotspot principle. The West Liao River region remains at the center of the spread zone of ancient Mongolic and Tungusic written varieties such as Middle Mongolian , Khitan and Jurchen and it is also the region representing the greatest linguistic diversity with regard to the deepest subgroups of contemporary varieties of Mongolo-Tungusic, notably pockets of Tungusic and Mongolic are present in the area.

Cultural reconstruction. The Proto-Mongolo-Tungusic vocabulary of subsistence is largely continuous with that of Proto-Altaic, except for the addition of terms relating to hemp cultivation and of distinctions in pig vocabulary. The absence of evidence of dairying vocabulary at a time depth of 4491 BP excludes the region of central Mongolia, the eastern Mongolian steppe and the Yellow Rover region as possible homelands because evidence for dairying was already present in these areas from around 5000 BP onwards. According to Cai et al. (2018) some marginal domesticated cattle (*Bos taurus*) remains dating back to 5300 BP were found at the Houtoumuga site in Jilin. However, the earliest unambiguous evidence for domesticated cattle in China is from the Yellow river and cattle is rare in the West Liao River area until the Bronze age (Yu 2020); see SI 5 and 6. Moreover, milk consumption cannot be confirmed in the West Liao River region in the Neolithic through direct proteomic evidence (Warinner pc). This is consistent with the lack of dairying vocabulary in the proto-Mongolo-Tungusic vocabulary.

Bayesian phylogeography. We infer a central point of origin on the eastern steppe in Northeastern Mongolia. Integrating this location with the one inferred through the traditional assessment methods, we propose a location for the Proto-Mongolo-Tungusic homeland as indicated in Fig. SI 4.10.

Figure SI 4.4 The Proto-Mongolo-Tungusic homeland identified on the basis of the diversity hotspot principle, cultural reconstruction and Bayesian phylogeography

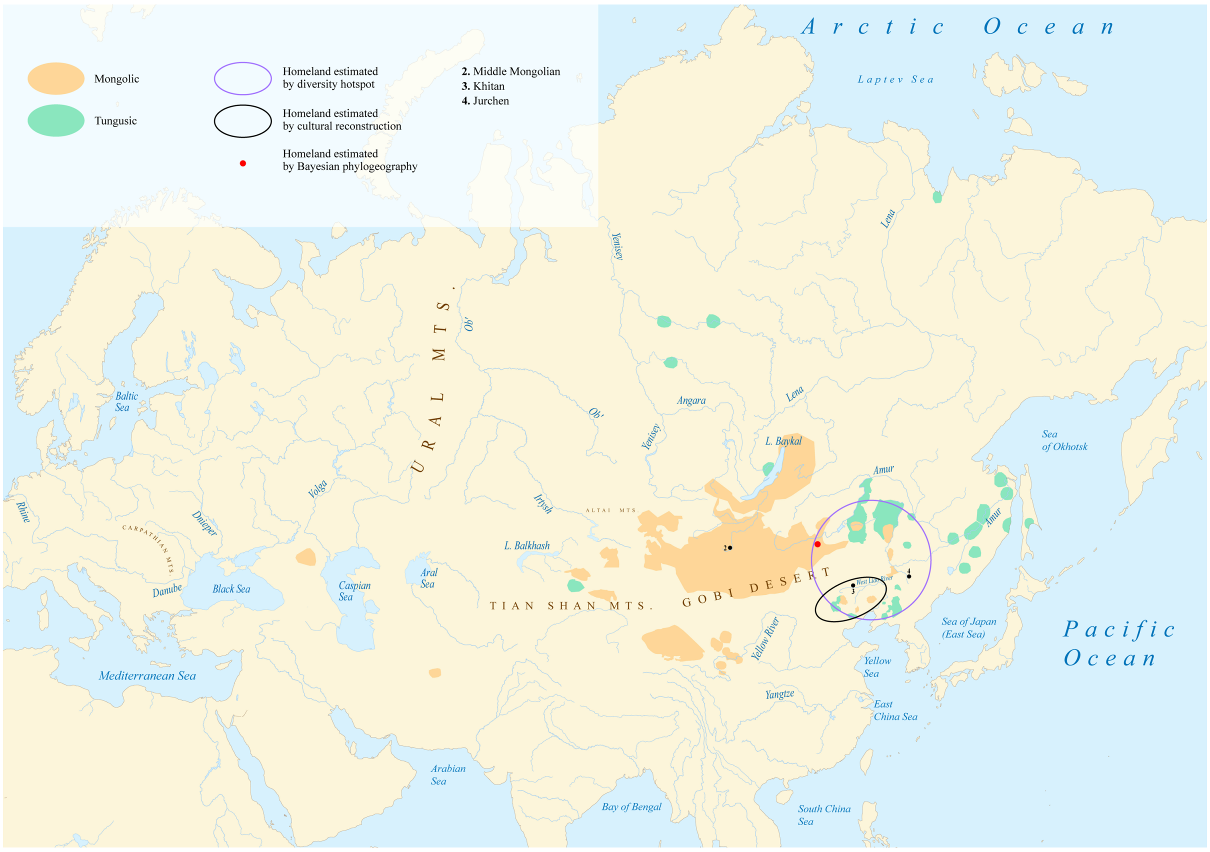

5. The Proto-Turkic homeland in the Bronze Age

Location method	Evidence	Source	Estimated location	Lat. \ Long.
**Diversity hotspot principle**	greatest linguistic diversity with regard to deepest subgroups (Bulgharic, Common Turkic)		Volga region	
**Cultural reconstruction**	2195 BP (1882-2493 95% HPD) Landscapes and fauna and flora from southern taiga-steppe zone, millet agriculture, animal husbandry, dairying, pastoralism	Teništev et al. 2001, 2006 Savelyev 2017 Robbeets et al. 2020 SI 4	From Ordos Plateau in Shanxi and Inner Mongolia to Sayan-Altai Mountains	
**Bayesian phylogeography**	Basic vocabulary cognates	SI 3	North Kazakhstan	50,18 65,16

Diversity hotspot principle. The primary split in the Turkic family is between the Bulgharic and the Common Turkic branch; see SI 24. In spite of its prehistorical origins in present-day Eastern Mongolia, the Bulgharic branch is historically attested only far to the west, i.e. in Eastern Europe, and its only living representative, Chuvash, survives nowadays in the Volga

region, the central-eastern part of European Russia. Therefore, the application of the diversity hot spot principle, using the contemporary location of the Turkic languages is deceptive in this case, as it points to the Volga region as the region with the highest linguistic diversity with regard to the primary subgroups. Nevertheless, internal diversity within Common Turkic, the other primary subgrouping, suggests a homeland that is situated more to the east, because diversity increases as we move towards the northeastern part of Central Asian region.

Cultural reconstruction. Associating Proto-Turkic vocabulary related to landscape, flora and fauna with palaeobotanical evidence, Tenishev and Dybo (2001-2006) locate the Proto-Turkic homeland in the transition zone between the mountain environment of southern taiga zone and steppe landscapes in present-day Mongolia. As the Proto-Turkic subsistence vocabulary reflects a gradual shift from agricultural subsistence to nomadic pastoralism (Savelyev 2017; SI 4), it is very likely that in an earlier period, prior to the spread of pastoralism, the Proto-Turkic speakers were millet farmers who occupied a more compact zone on the Ordos Plateau in present-day Shanxi province and Inner Mongolia. Moreover, the study of ancient linguistic borrowings (Dybo 2007, Helimski 2000, Lubotsky and Starostin 2003, Schönig 2003; SI 4) supports language contact between the speakers of Proto-Turkic and the Proto-Macro-Mongolic speakers in the east, the Sinitic speakers in the southwest, the Tocharian speakers in the west, and the Proto-Samoyedic speakers in the northwest. As such, cultural reconstruction indicates a Proto-Turkic homeland stretching from the Sayan-Altai Mountain region (South Siberia) in the west to present-day Inner Mongolia and Shanxi in the east.

Bayesian phylogeography. The central point of origin inferred as far west as North Kazakhstan may be biased due to the fact that all earlier linguistic diversity among Bulgharic languages has been erased and that these languages were originally situated much more to the east than Chuvash, the only surviving Bulgharic daughter language today. The integration of the three methods of homeland detection will thus lead to a rather large distribution, stretching from present-day Inner Mongolia and Shanxi in the east to North Kazakhstan in the west; see Section 4.10.2.

Figure SI 4.5 The Proto-Turkic homeland identified on the basis of the diversity hotspot principle, cultural reconstruction and Bayesian phylogeography

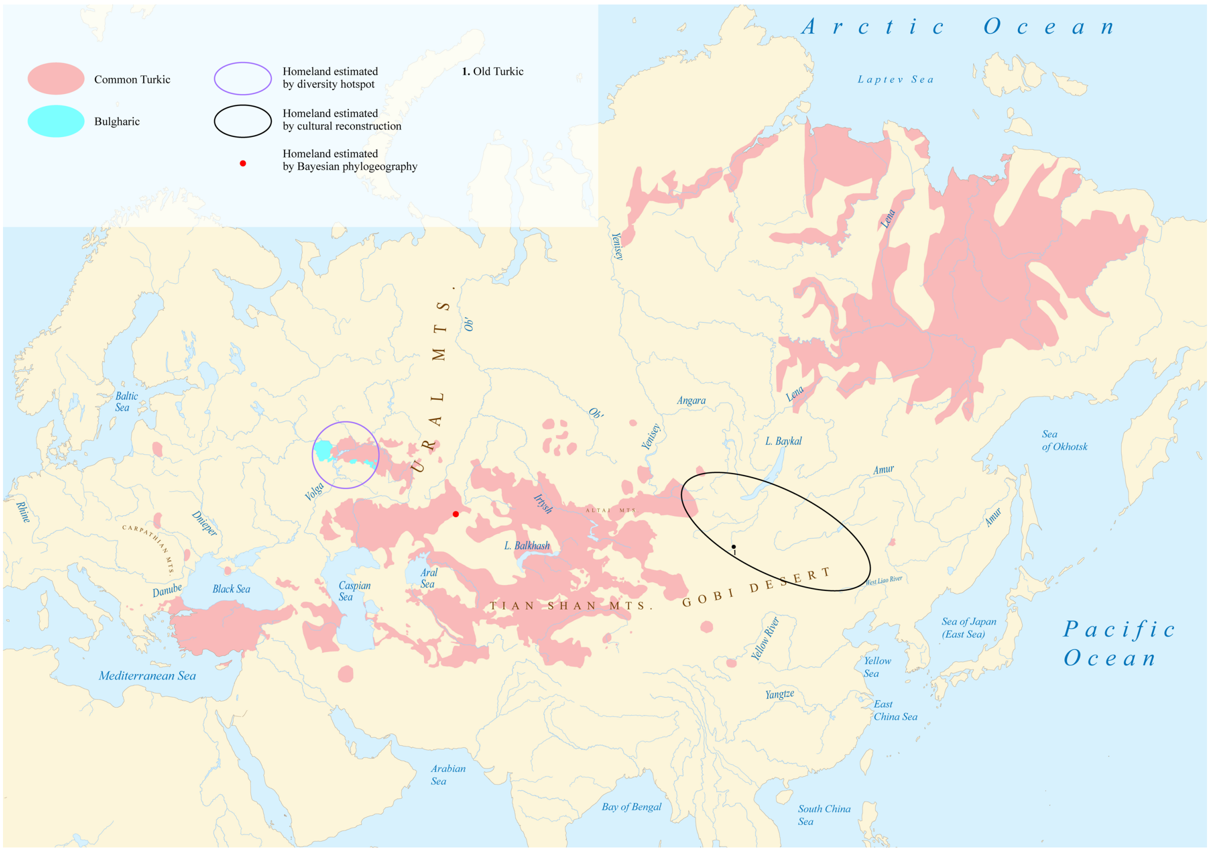

6. The Proto-Mongolic homeland in the Bronze Age

Location method	Evidence	Source	Estimated location	Lat. \ Long.
**Diversity hotspot principle**	greatest linguistic diversity with regard to deepest subgroups (Proto: Shirongolic, Mongolic proper; Macro: Khitan, Middle Mongolian)	Figure SI 3.6	Proto: Gansu-Inner Mongolia border<br><br>Macro: West Liao and Nen river region	
**Cultural reconstruction**	939 BP (871-1011 95%HPD) Agriculture, millets, pigs, cattle, dairying, pastoralism	Robbeets et al. 2020 SI 4	West Liao, Nen river region and Eastern Steppe	
**Bayesian phylogeography**	Basic vocabulary cognates	SI 2	Mongolian Plateau, south of Ulaan Bator	43,51 103,00

Diversity hotspot principle. The Mongolic language family, as we know it today from the contemporary Mongolic languages, represents the result of the diversification of Proto-Mongolic, which was the language spoken by the historical Mongols around the time of the Mongol Empire (1206–1368). Although the diversity hotspot principle points to a homeland on the border between the provinces of Gansu and Inner Mongolia, where languages of the Shirongolic and Mongolic proper branches come together, the source region of Mongolic can

be reliably located on the basis of historical information. Not surprisingly, it was the very region at the intersection of northeastern Mongolia and northwestern Manchuria from where the historical Mongols rose, that is, the region roughly defined by the basins of the rivers Onon and Argun and their tributaries. However, recent research has shown that there is an extinct branch parallel to the Proto-Mongolic lineage, which we call "Khitanic". It comprises Khitan, the dynastic language of the Liao Empire (907–1125), which is only fragmentarily attested along with earlier and hardly attested languages spoken by the Tabghach of the Northern Wei (386–534), the Xianbei or "Serbi" (208 BC–235 AD) and the Donghu (the first millennium BC). As these ethnolinguistic groupings were all concentrated around the West Liao River area, the earlier hotspot of Macro-Mongolic linguistic diversity can be situated in that region.

Cultural reconstruction. Only few agricultural items have been reconstructed for Khitan, but it appears that the speakers of Macro-Mongolic were familiar with agriculture, animal husbandry including pigs, chickens and cattle, dairying and horse-ridden pastoralism. This observation allows for a large area including the Manchurian Basin and the Mongolian Plateau. Bronze Age contacts with Proto-Sinitic, Proto-Turkic as well as Proto-Tungusic support the location of the Marco-Mongolic homeland in the West Liao river region. For proto-Mongolic a homeland at the intersection of northeastern Mongolia and northwestern Manchuria is historically supported.

Bayesian phylogeography. For Proto-Mongolic, we infer a central point of origin on the Mongolian Plateau, south of Ulaan Bator. As we do not have sufficient basic vocabulary for the Khitan language, the Khitanic branch is not represented in our dataset. Therefore, we cannot infer a location for Macro-Mongolic on the basis of Bayesian phylogeography. However, taking into account the locations indicated by the traditional assessment methods, we propose a location for the Mongolic homeland as indicated in Fig. SI 4.10.

Figure SI 4.6 The Proto-Mongolic homeland identified on the basis of the diversity hotspot principle, cultural reconstruction and Bayesian phylogeography

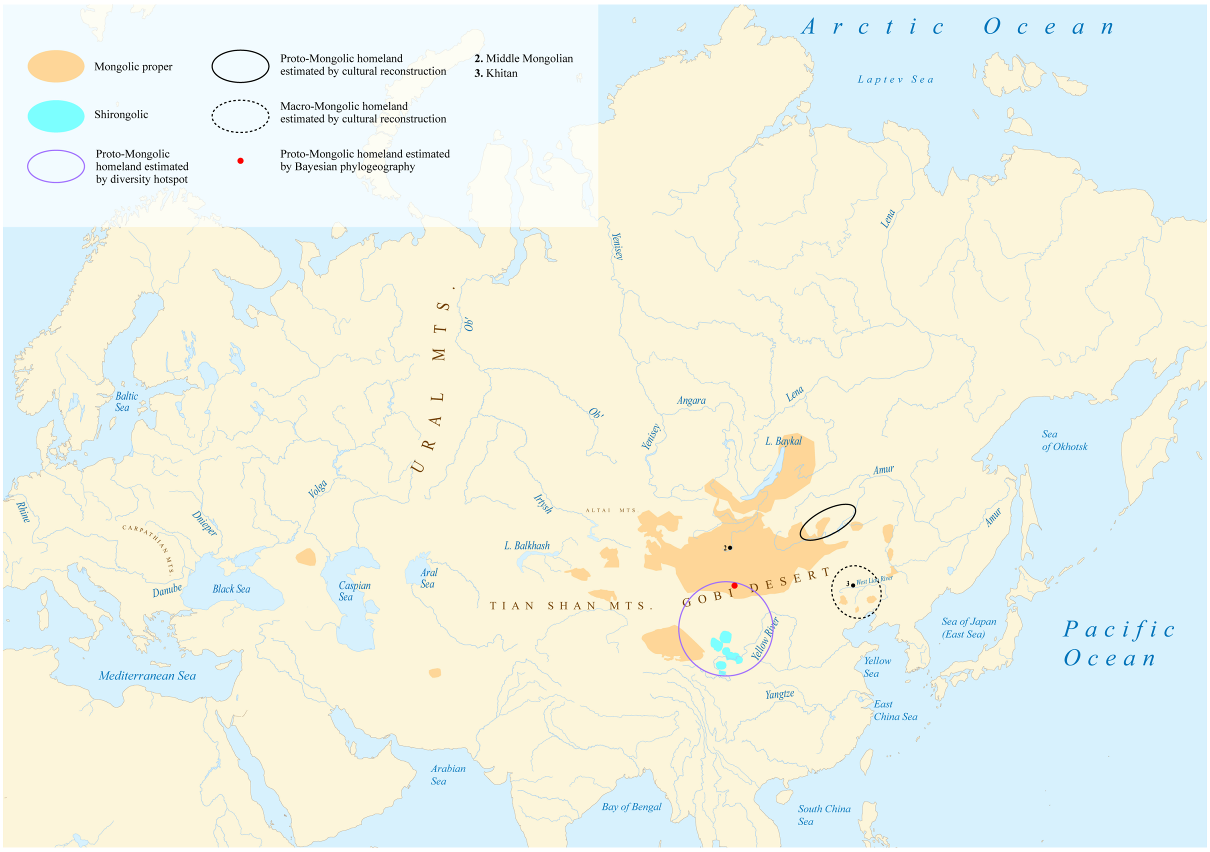

7. The Proto-Tungusic homeland in the Bronze Age

Location method	Evidence	Source	Estimated location	Lat. \ Long.
**Diversity hotspot principle**	greatest linguistic diversity with regard to deepest subgroups (Manchuric, Tungusic proper)	Figure SI 3.7	Northern Heilongjiang and Mid-Amur region	
**Cultural reconstruction**	1950 BP (1499-2412 95%HPD)<br>Agriculture, millets, barley, iron, fauna and flora specific for Khanka-Ussuri region.	Li et al. 2020<br>Wang and Robbeets 2020<br>SI 4	Khanka-Ussuri region	
**Bayesian phylogeography**	Basic vocabulary cognates	SI 2	Northern Heilongjiang, lesser Khingan range	47,07<br>124,42

Diversity hotspot principle In SI 24, we put the primary split in the Tungusic family between the Manchuric and Tungusic proper branches. As a result, the diversity hotspot principle locates the homeland in the region where languages belonging to both branches are spoken, namely on the Chinese-Russian border including Northern Heilongjiang province in China and the Mid-Amur region in Russia.

Cultural reconstruction. Since Neolithic times, the cultural edge in the Russian Far East was in the south, not in the north. The area around Lake Khanka and the Ussuri river is the most plausible homeland for the ancestral Tungusic speech community because after the introduction of the Zaisanovskaya culture (5200-3300 BP), it was the first center of millet cultivation in the Russian Far East. Tungusic reconstructions of agricultural vocabulary include the term for ‘broomcorn millet’, which is not native to the region and has been imported as a domesticated crop from the West Liao region. The reconstruction for ‘barley’ is a borrowing from Sinitic (see SI 4) and can be correlated to the archaeological evidence for barley being first imported through Chinese contact at the time of the Krounovskaya culture (2600-1800 BP) situated in the Southern Primorye south of Lake Khanka (Sergusheva and Vostretsov 2009: 214–215; Leipe et al. 2019). Besides, Proto-Tungusic vocabulary includes the reconstruction of 'iron', the first uncontested findings of which in the Russian Far East go back to the Krounovskaya culture. A location in the Khanka-Ussuri region is further supported by reconstructed words for fauna and flora, such as 'Korean pine (*Pínus koraiénsis*)' which are limited to that area.

Bayesian phylogeography. We infer a central point of origin on the intersection between the lesser Khingan range and North Heilongjiang Province. Integrating this location with the one inferred through the traditional assessment methods, we propose a location for the Proto-Tungusic homeland as indicated in Fig. SI 4.10.

Figure SI 4.7 The Proto-Tungusic homeland identified on the basis of the diversity hotspot principle, cultural reconstruction and Bayesian phylogeography

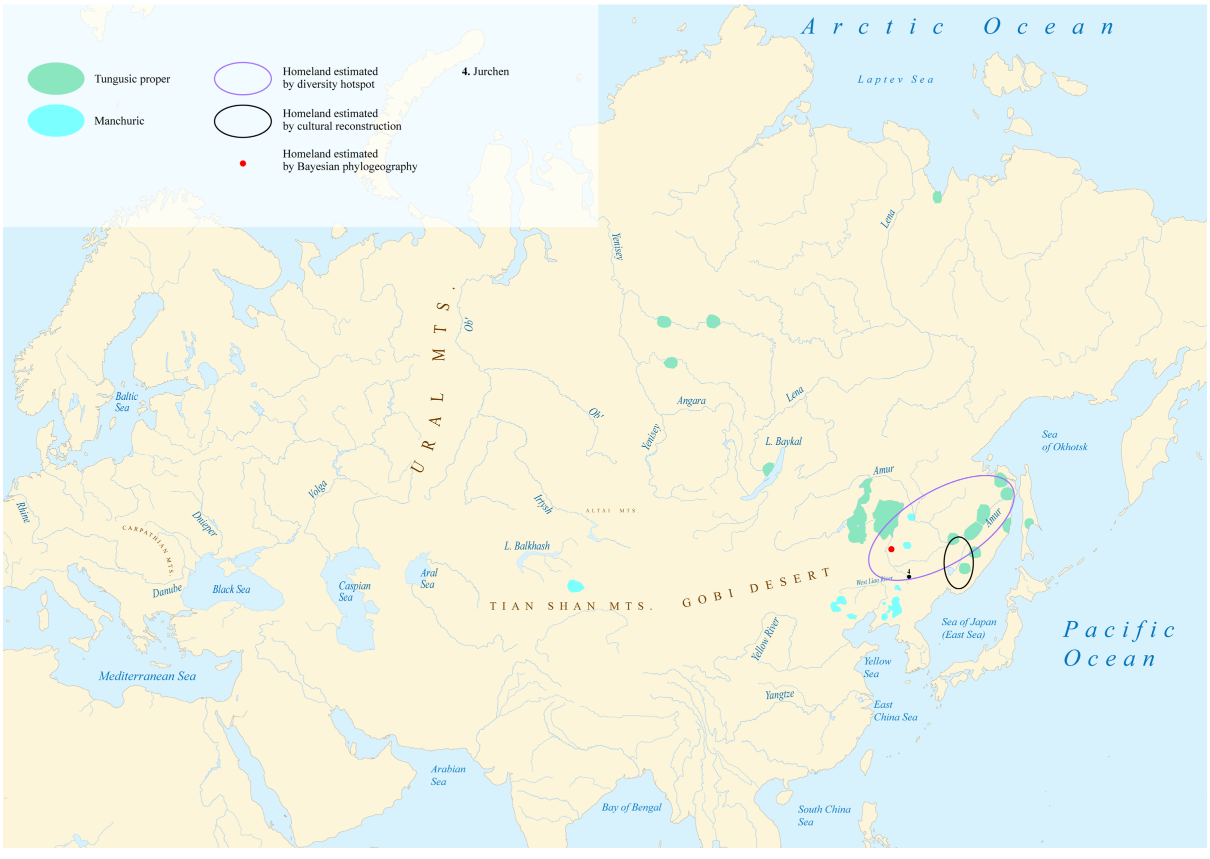

8. The Proto-Koreanic homeland in the Bronze Age

Location method	Evidence	Source	Estimated location	Lat. \ Long.
**Diversity hotspot principle**	greatest linguistic diversity with regard to deepest subgroups (Proto: Jeju and Korean proper; Macro: Paekche , Kaya and Silla)	Figure SI 3.8	Proto: Southwestern Korea<br><br>Macro: Southeastern Korea	
**Cultural reconstruction**	975 BP (528-1560 95%HPD)<br>Recycling of millet agricultural vocabulary as rice agricultural vocabulary, agricultural tools, animal husbandry, pig, ruminants, horse textile production	Robbeets et al. 2020 Hudson and Robbeets 2020	Proto: Korea<br><br>Macro: midwestern to southeastern Korea	
**Bayesian phylogeography**	Basic vocabulary cognates	SI 2	midwestern Korea: Seoul area	35,40 126,40

Diversity hotspot principle. The Koreanic language family, as we know it today from the contemporary Korean dialects, represents the result of the diversification of Proto-Koreanic. Given the first separation in the Proto-Koreanic tree in SI 24 between Jeju and the dialects of Korean, the diversity hotspot principle points to a homeland on the Southwestern Korean coast across Jeju Island. However, research has shown that in the Three Kingdoms period (AD 300–668) the kingdoms of Paekche, Kaya and Silla each had their individual languages, associated with different branches of Koreanic (Lee and Ramsey 2011). The Silla kingdom unified the Korean peninsula politically and linguistically in 668, erasing all pre-existing linguistic diversity. Due to this unification, the contemporary Korean dialects cannot be traced back any deeper in time than the end of the first millennium AD. Thus, the separation of Proto-Koreanic into the predecessors of the contemporary dialects corresponds roughly to the time of the break-up of Silla Old Korean and its location corresponds to the original territory of Silla in Southeastern Korea. Nevertheless, we assume that there are two other branches parallel to the Proto-Koreanic lineage that went extinct, notably the branches to which the languages spoken in the Paekche and Kaya kingdoms belonged. The location in Southeastern Korea where the Silla, Paekche and Kaya linguistic communities meet can thus be seen as an earlier hotspot of diversity, and hence homeland, of Macro-Koreanic.

Cultural reconstruction. Due to the bottleneck through which only Silla Old Korean was able to pass, the reconstruction of Proto-Koreanic vocabulary does not bring us much further back in time than the first millennium AD. Not surprisingly, the subsistence terminology at that time was varied, including terms for millet and rice agriculture, tool

technology, animal husbandry including pigs, ruminants and horses, complex textile production, etc. Nevertheless, there are some indications that rice terminology developed secondarily during the time before the break-up of Proto-Korean, through borrowing from neighboring languages or lexical recycling of millet vocabulary (see SI 4). For instance, we see a gradual semantic specialization from ‘millet’ or ‘grain in general’ to ‘rice’ in the reconstructed rice vocabulary suggesting that rice terminology developed from pre-existing millet terminology. If the ancestral Koreanic language was indeed present before the introduction of rice vocabulary, its homeland should be situated in the core area of Neolithic millet agriculture, stretching from midwestern to southeastern Korea.

Bayesian phylogeography. We infer a central point of origin in the Seoul area in midwestern Korea. Integrating this location with the one inferred through the traditional assessment methods, we propose a location for the Koreanic homeland from midwestern to southeastern Korea, as indicated in Fig. SI 4.10.

Figure SI 4.8 The Proto-Koreanic homeland identified on the basis of the diversity hotspot principle, cultural reconstruction and Bayesian phylogeography

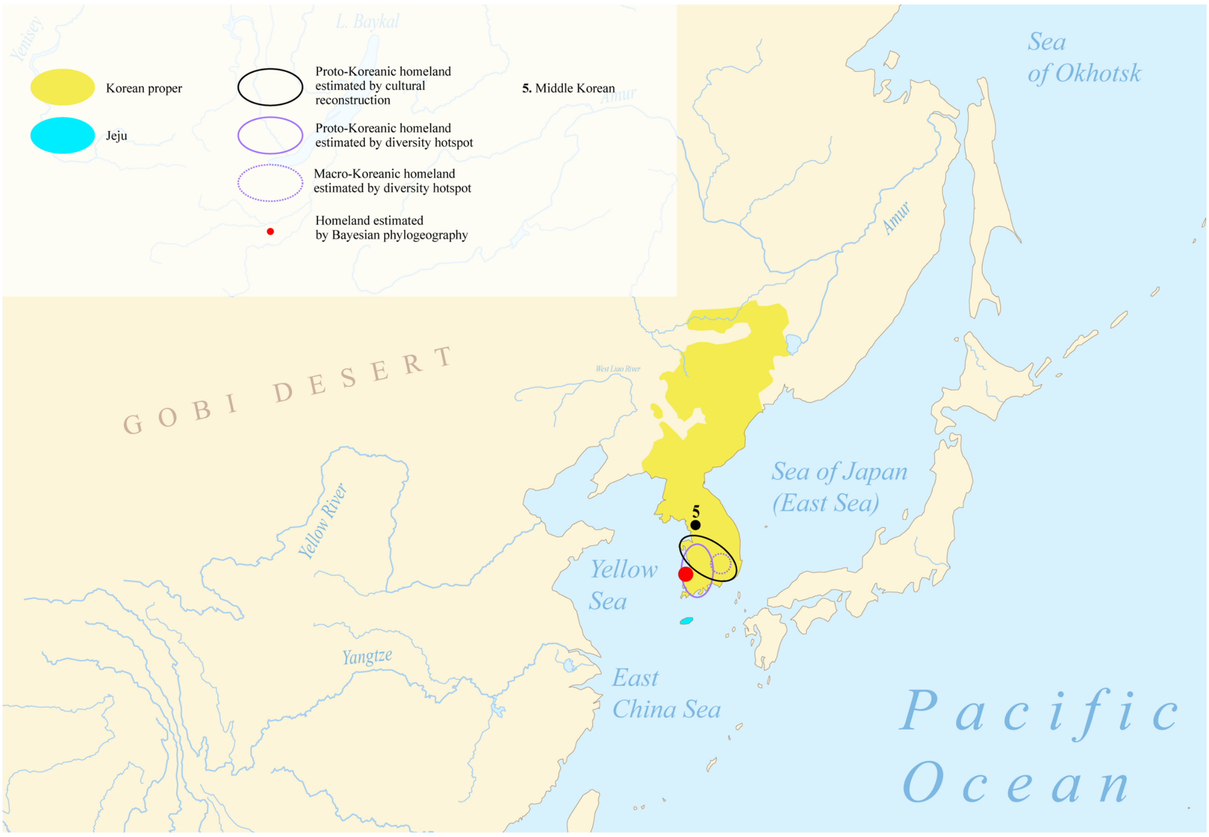

9. The Proto-Japonic homeland in the Bronze Age

Location method	Evidence	Source	Estimated location	Lat. \ Long.

**Diversity hotspot principle**	greatest linguistic diversity with regard to deepest subgroups (Japonic proper and Ryukyuan)	Figure SI 3.9	Islands South of Kyushu	
**Cultural reconstruction**	2136 BP (1499-2412 95%HPD)<br><br>Millet, rice, barley, wheat, agricultural tools, textile technology, bronzes	Robbeets et al. 2020	Kyushu	
**Bayesian phylogeography**	Basic vocabulary cognates	SI 3	Islands immediately South of Kyushu	31,07<br>130,05

Diversity hotspot principle. According to the classification in SI 24, Proto-Japonic first separated into a Ryukyuan and a Mainland Japanese branch. Therefore, the diversity hotspot is situated on the Islands to the South of Kyushu and North of the Ryukyu Islands. We mentioned above that prior to its arrival on the Japanese Islands, a language ancestral to Proto-Japonic was spoken on the Korean peninsula. Therefore, we refer to all continental varieties of Japonic as Macro-Japonic.

Cultural reconstruction. The subsistence vocabulary of Proto-Japonic is diverse, including terms for millet, rice, wheat and barley cultivation, harvesting technology, pig husbandry, bronzes, complex textile production etc. As the vocabulary is consistent with the nature of the agricultural expansion from the Korean Peninsula to Kyushu in the Bronze age, it indicates that Kyushu is a probable homeland for Proto-Japonic.

Bayesian phylogeography. We infer a central point of origin near the islands situated immediately south of Kyushu. Integrating this location with the one inferred through the traditional assessment methods, we propose a location for the Japonic homeland on Kyushu and the adjacent southern islands, as indicated in Fig. SI 4.10.

Figure SI 4.9 The Proto-Japonic homeland identified on the basis of the diversity hotspot principle, cultural reconstruction and Bayesian phylogeography

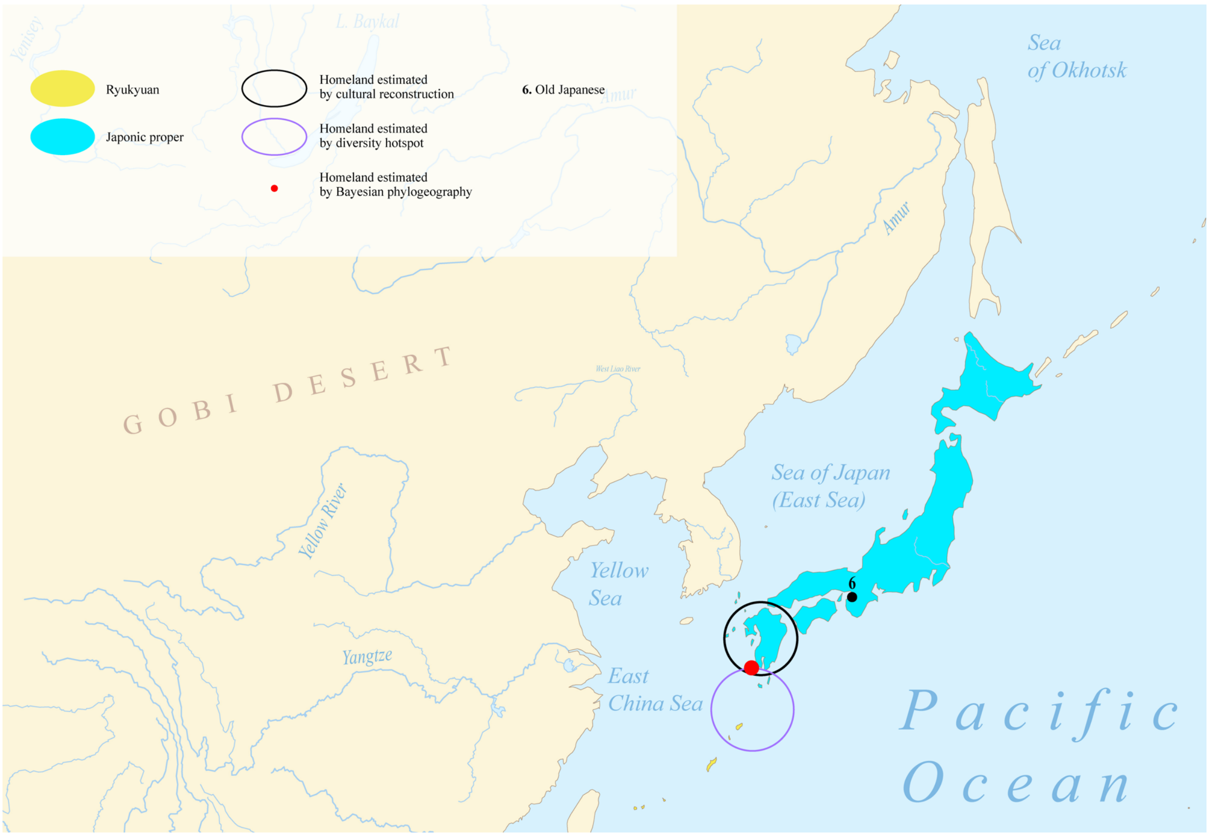

10. Integration of the different homeland detection methods

10.1. Methods and limitations

In order to estimate the location of the ancient speech communities involved, we combined Bayesian phylogeography, the diversity hotspot principle and cultural reconstruction in a single approach. Bayesian phylogeography models how speech communities may have moved using a phylogenetic tree as a guideline, by using the diffusion rate at which languages change their location to infer the spatial location of the root and all ancestral nodes. The diversity hotspot principle assumes that the homeland is located in the geographical area with the highest linguistic diversity with regard to the deepest subgroups in the family. Cultural reconstruction delimits an area where the natural elements, material objects, concepts and practices revealed in the linguistic reconstructions converge to the exclusion of other regions.

Each of these three methods has its own limitations. Bayesian methods provide a set of plausible homelands, an area associated with the highest likelihood or posterior probability, rather than single locations. Wichmann and Rama (2020) argue that BEAST tends to infer homeland areas that are too large to be of use, possibly because this software involves the simultaneous inference of phylogeny and phylogeography and is overparametrized. They suggest that other Bayesian methods, in particular BayesTraits, are more efficient. Second, "migrations" (Neureiter et al. 2021) or "early jumps" (Wichmann and Rama 2020), where one place is abruptly left for another are a major source of error. Bayesian methods are thought to produce more reliable results in the case of "expansions", whereby speech communities gradually expand their territory. Finally, Bayesian random walks cannot easily deal with directional trends emerging from geographical or ecological constraints, such as oceans, mountains and deserts (Wichmann and Rama 2020, Neureiter et al. 2021).

The identification of a homeland through the diversity hotspot principle depends on the location of the deepest subgroups and therefore, on how robustly the internal structure of a given family has been established. Uncertainty with regard to the primary branching in the family, will thus lead to uncertainty in the reconstruction of the homeland. Besides, the contemporary hotspot of linguistic diversity may diverge from an earlier one, for instance, due to the loss of diversity in the homeland due to later spreads.

Finally, cultural reconstruction relies on the assumptions that meanings of words can be accurately reconstructed to a proto-language, but it is a fact that semantic reconstruction is less precise than phonological reconstruction and leaves room for subjectivity and interpretation. In addition, it is not sufficient to reconstruct a single cultural or natural item to an ancestral language, but our reconstruction should be backed up by other members of the semantic domain to which it belongs. It may therefore be hard to come by a sufficient number of proto-words that are truly geographically diagnostic.

10.2. Overcoming the limitations specific to the Transeurasian case

Given these limitations and considering cases in which one method is not foolproof or even not applicable, the three methods serve to complement each other.

In our Bayesian phylogeographic approach, we use BEAST v2.6 with adaptive coupled MCMC. The best fit is provided by the pseudo Dollo covarion model with a relaxed clock. As this model is not available in BayesTraits and because BEAST is specialised in time trees, we opted for these settings and software. Our Bayesian phylogeographic analysis uses the posterior tree set from our lexical analysis. We assigned point positions to the tips and randomly sampled trees from the posterior while estimating geographical parameters through MCMC. The resulting maps with circles corresponding to the 95% highest posterior density intervals are shown in SI 3. In line with the reservations expressed by Wichmann and Rama 2020, these intervals are indeed too large to be informative. We solve this issue by representing the dots with the highest posterior probability estimated by the Bayesian phylogeography in red on the maps above and by integrating these dots with the locations suggested by diversity hotspot and cultural reconstruction. The resulting map in Fig. SI 4.10 with the linguistic homelands proposed for the root and the nodes in the Transeurasian family, therefore gives a realistic approximation of where the homelands were situated.

The population movements in the Transeurasian case are not to be understood as a permanent movement of an entire population, involving a whole language family leaving its original homeland. Rather, growing populations requiring more resources cause certain subgroups to leave the original homeland and to colonize new territory. This causes inhabitants of the new territory to abandon their local language and adopt the incoming language of the colonizers. Similar to most cases of farming/language dispersal, this scenario is in line with the expansion model rather than with the migration or “early jumps” model as defined by Neureiter et al. (2021) and Wichmann and Rama (2020). Therefore, we can expect a reliable performance of Bayesian homeland detection methods in the Transeurasian case.

For a few nodes in our tree the predicted issue with Bayesian random walks not being able to respond properly to geographical or ecological constraints indeed appears to be the case, such as the situation of Proto-Altaic in the Northeastern Altai Mountains or Proto-Japonic in the Kagoshima Bay immediately south of Kyushu. However, these are only minor aberrations that are easily removed by integrating the Bayesian results with those of the hotspot diversity principle and cultural reconstruction. The situation of the Proto-Transeurasian homeland in the uncultivable sand dunes of the Gobi desert may seem problematic in view the connection with agriculture proposed in our research. However, Yang et al. (2015) demonstrated that this

area was used as farmland before it became a desert ca. 4200 years ago through groundwater capture by the Xilamulun River.

There are several nodes in our tree, for which the application of the diversity hotspot principle would be misleading if we considered only contemporary linguistic diversity. This is the case for Proto-Japano-Koreanic, Proto-Koreanic, Proto-Mongolic and Proto-Turkic because we know from historical sources that earlier linguistic diversity has been erased by dominant linguistic expansions by the end of the first and the beginning of the second millennium AD, such as the Silla unification in Korea, the Mongolic unification under Jinghis Khan and the expansion of Common Turkic over the Eurasian Steppe. However, taking indications of earlier diversity into account, the results of the diversity hotspot principle reach a better convergence with the other homeland detection methods used in this study.

In the case of Proto-Turkic, the bias caused by the domination of the descendants of Eastern Old Turkic led to a rather large distribution of the homeland, stretching from present-day Inner Mongolia and Shanxi in the east to North Kazakhstan. The southeastern homeland indicated by cultural reconstruction probably represents the best approximation of the original location of Proto-Turkic, at a time following its separation from Altaic. Due to this early break-up, Proto-Turkic covers a long period from the Middle Neolithic to the Early Iron Age. Therefore, the Proto-Turkic homeland on the map in Fig. SI 4.10 can be considered as a dynamic entity, gradually expanding from Southeast to Northwest from the Middle Neolithic to the Early Iron Age.

Conversely, when the data for cultural reconstruction is limited or the archaeological record is slim, then that method may be less reliable and the other methods can step in. In this way, we gain by the interaction of the three homeland detection methods.

It could be argued that the different methods are not completely independent from each other: The diversity hotspot method depends on tree structure, so in that sense there is a certain overlap with Bayesian phylogeography. Phylogeography also relies on the logic of bottom-up reconstruction, which overlaps somewhat with cultural reconstruction using proto-forms. Nevertheless, the three detection methods applied in our study are sufficiently independent of each other as they do not only make use of different principles of analysis but also rely on different lines of evidence. As shown in the tables in each section above, the Bayesian approach is based on a dataset of basic vocabulary, whereas the diversity hotspot principle starts from geographic observations about the distribution of the languages involved and cultural reconstruction relies on the reconstruction of natural and agropastoral vocabulary. As such, the homeland detection applied here advances convergent evidence and not just convergent methods.

Fig. SI 4.10 infers a location for the homelands of the root and nodes of the Transeurasian family based on the integration of the three homeland detection methods applied in our research.

Fig. SI 4.10 Reconstructed Transeurasian linguistic homelands, distinguishing between ancestral languages spoken during Neolithic times (red) and those spoken in the Bronze Age and later (green). The estimated time depth represented in the bar plot is based on Bayesian inference presented in SI 24. The credible intervals for the break-up time of the ancestral languages represented in the bar plot are following: Proto-Transeurasian 9181 BP (5595 - 12793 95% HPD); Proto-Japano-Koreanic 5458 BP (3335-8024 95%HPD); Proto-Altaic 6811 BP (4404-10166 95%HPD); Proto-Mongolo-Tungusic 4491 BP (2599-6373 95%HPD); Proto-Turkic 2195 BP (1882-2493 95% HPD); Proto-Mongolic 939 BP (871-1011

95%HPD); Proto-Tungusic 1950 BP (1499-2412 95%HPD); Proto-Koreanic 975 BP (528-1560 95%HPD) and; Proto-Japonic 2136 BP (1499-2412 95%HPD).

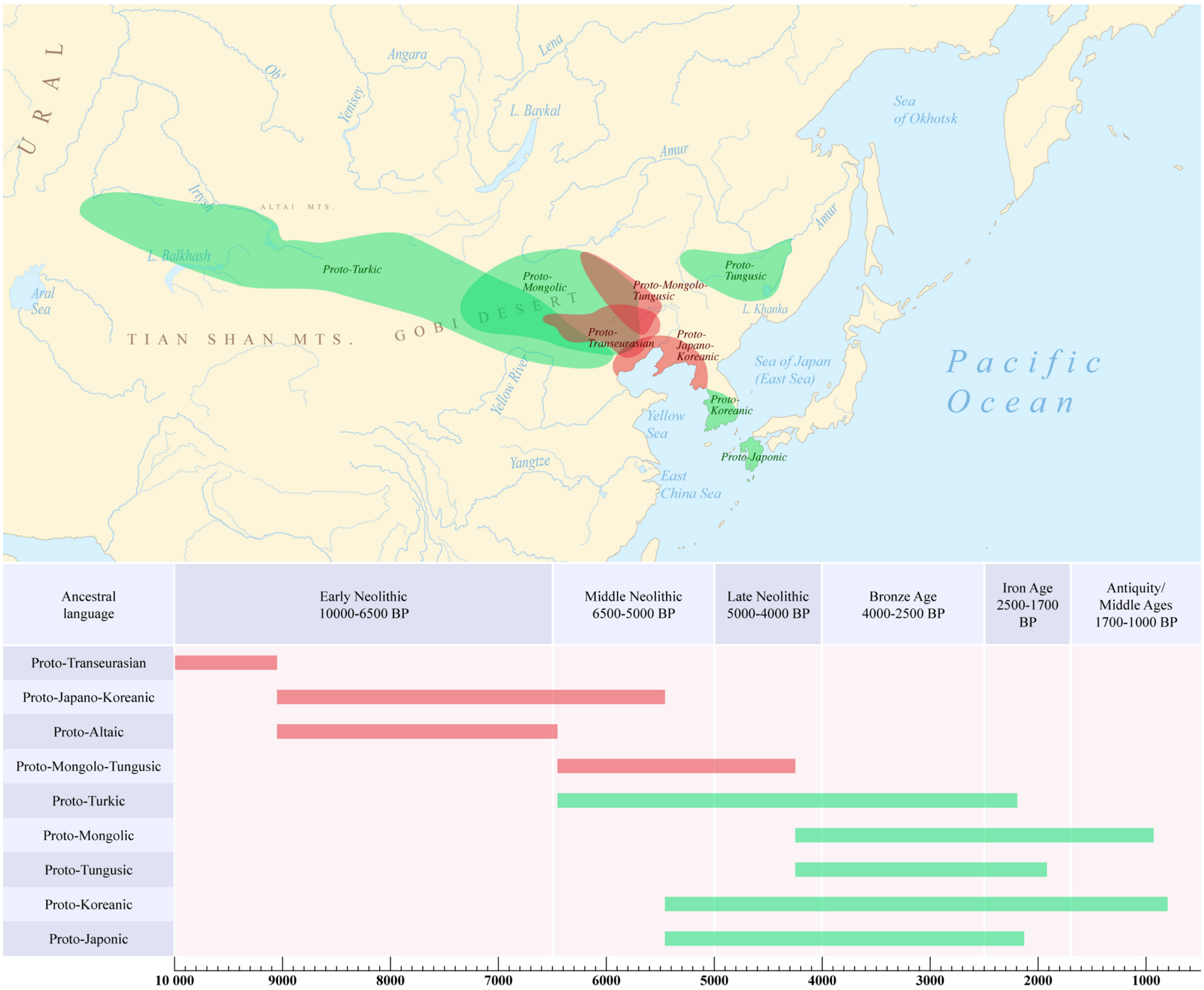

References mentioned in Supplementary Information 4

Beckwith, C. I. *Koguryo, the Language of Japan's Continental Relatives: An Introduction to the Historical-Comparative Study of the Japanese-Koguryoic Languages with a Preliminary Description of Archaic Northeastern Middle Chinese* (Brill, 2007).

Bentley, J. R. in *Selected Papers from the 11th International Conference on Korean linguistics* (eds Park, B-S. & Hye Sook Yoon, J.) 318–325 (International Circle of Korean Linguistics, 1998).

Bentley, J. R. A New Look at Paekche and Korean: Data from Nihon Shoki. *Language Research* **36.2**, 417–443 (2000).

Cai, D. et al. Ancient DNA reveals evidence of abundant aurochs (Bos primigenius) in Neolithic northeast China. *J. Archaeol. Sci*. **98**, 72-80 (2018).

Dybo, A. V. *Lingvisticheskije kontakty rannix tjurkov. Leksicheskij fond. Pratjurkskij period* [Language Contact of the Early Turks. The Lexical Stock. The Proto-Turkic Period] (Vostochnaja literatura, 2007).

Francis-Ratte, A. *Proto-Korean-Japanese: A New Reconstruction of the Common Origin of the Japanese and Korean Languages* (The Ohio State University PhD dissertation, 2016).

Helimski, E. *Komparativistika, uralistika. Lekcii i statji* [Comparative linguistics, Uralic studies. Lectures and papers] (Jazyki russkoj kul'tury, 2000).

Hudson, M. J. & Robbeets, M. Archaeolinguistic evidence for the farming/language dispersal of Koreanic. *Evolutionary Human Sciences* **2**, e52 doi:10.1017/ehs.2020.49 (2020).

Kōno, R. The bilingualism of the Paekche language. *Memoirs of the Research Department of the Toyo Bunko* **45**, 75–86 (1987).

Lee, K-M. & Ramsey, R. S. *A History of the Korean Language* (Cambridge University Press, 2011).

Leipe, C., Long, T., Sergusheva, E. A., Wagner M. & Tarasov, P. E. Discontinuous spread of millet agriculture in eastern Asia and prehistoric population dynamics. *Science Advances* **5(9),** eaax6225 (2019).

Li, T., Ning, C., Zhushchikhovskaya, I. S., Hudson, M., & Robbeets, M. Millet agriculture dispersed from Northeast China to the Russian Far East: integrating archaeology, genetics, and linguistics. *Archaeological Research in Asia* **22**, 100177 doi:10.1016/j.ara.2020.100177 (2020).

Lubotsky, A. & Starostin, S. in *Language in Time and Space. A Festschrift for Werner Winter on the occasion of his 80th birthday* (eds Bauer, B. L. M. & Pinault, G-J.) 257–270 (Mouton de Gruyter, 2003).

Nelson, S. et al. Tracing population movements in ancient East Asia through the linguistics and archaeology of textile production. *Evolutionary Human Sciences,* 1-20 (2020).

Neureiter N., Ranacher P., van Gijn R., Bickel B. & Weibel R. 2021 Can Bayesian phylogeography reconstruct migrations and expansions in linguistic evolution? R. Soc. Open Sci. **8**, 201079 (2021). https://doi.org/10.1098/rsos.201079

Robbeets, M. in *The Oxford Guide to the Transeurasian Languages* (eds Robbeets, M. & Savelyev, A.) 772–783 (Oxford Univ. Press, 2020).

Robbeets, M., Janhunen, J., Savelyev, A. & Korovina, E. in *The Oxford Guide to the Transeurasian Languages* (eds Robbeets, M. & Savelyev, A.) 754–771 (Oxford Univ. Press, 2020).

Savelyev, A. in *Language Dispersal Beyond Farming* (eds Robbeets, M. & Savelyev, A.) 117–148 (Benjamins, 2017).

Schönig, C. in *The Mongolic Languages* (ed Janhunen, J.) 403–419 (Routledge, 2003).

Sergusheva, E. A., & Vostretsov, Y. E. in *From Foragers to Farmers: Papers in Honour of Gordon C. Hillman* (eds Fairbairn, A. & Weiss, E.) 205–219 (Oxbow books, 2009).

Tenishev, Ė. R. & Dybo, A. (2001–2006). *Sravnitel'no-istoricheskaja grammatika tjurkskix jazykov* [Comparative-Historical Grammar of the Turkic Languages] 3 vols. (Nauka, 2001-2006).

Wang, C-C. & Robbeets, M. The homeland of Proto-Tungusic inferred from contemporary words and ancient genomes. *Evolutionary Human Sciences* **2**, e8 doi:10.1017/ehs.2020.8 (2020).

Wichmann, S. & Rama, T. Testing methods of linguistic homeland detection using synthetic data *BioRxiv preprint* (2020) doi: https://doi.org/10.1101/2020.09.03.280826

Yang, X., Scuderi, L. A., Wang, X., Scuderi L. J., Zhang, D. et al. *Groundwater sapping as the cause of irreversible desertification of Hunshandake Sandy Lands, Inner Mongolia, northern China*. Proceedings of the National Academy of Sciences **112** (3), 702–6. (2015).

Yu, C. *The Origin of Cattle in China from the Neolithic to the Early Bronze Age* (BAR Publishing, 2020).

[1] The base map in the Figures of this SI file was downloaded from the Nature Earth map dataset (https://www.naturalearthdata.com/), granted for the public domain use and is free for use in any type of project. We designed the maps by ourselves and supplemented the maps with our own data points.
