# Supplementary material for: Triangulation supports agricultural spread of the Transeurasian languages
Source: Nature. 2021 Nov 10;599(7886):616–21. doi: 10.1038/s41586-021-04108-8 (PMC8612925; doi:10.1038/s41586-021-04108-8)
Supplement: Supplementary file 3 — This zipped file contains Supplementary Data Files 1, 2 and 4–6; see Supplementary Information file for full descriptions (Supplementary Data File 3 is hosted externally; see Supplementary Information file for links). [file 41586_2021_4108_MOESM3_ESM.zip › 2021-02-02920E-s3/20_Eurasia3angle_synthesis_SI 5_agropastoral_REV21.09.docx]

**Supplementary Information 5**

**Inherited and borrowed correspondence sets for agropastoral vocabulary**

**across the Transeurasian languages**

**1 Methods**

Combining dictionary search with fieldwork, we collected a database of agropastoral vocabulary for each of the five Transeurasian subfamilies; see SI 23a for Turkic, SI 23b for Mongolic, SI 23c for Tungusic, SI 23d for Koreanic and SI 23e for Japonic. To this end, we started from agropastoral vocabulary in the earliest sufficiently documented historical stages of our languages (i.e., Old Turkic, Middle Mongolian, Manchu, Late Middle Korean and Old Japanese) and searched for potential cognates in contemporary languages and dialects. For a good number of comparative sets, this yielded a reconstructed proto-form, i.e., an ancestral Turkic, Mongolic, Tungusic, Koreanic or Japonic word, from which the cognates are thought to have descended.

We compared these reconstructions in order to select look-alikes with comparable form and meaning across the five Transeurasian subfamilies. When a set of two or more words taken from different languages look alike, their similarity may be accounted for by different reasons: it could be due to universal principles in linguistic structuring such as sound-symbolism or nursery terms, to coincidence, to borrowing between the languages concerned or, to inheritance from a common source. Given the shared cultural context, it is likely that the similarities in the Transeurasian agropastoral vocabulary have a historical motivation but among the possible historical accounts, it is difficult to distinguish borrowing from inheritance at face value.

To solve this issue, historical comparative linguists can rely on a number of formal and semantic guidelines to distinguish linguistic borrowing from inheritance (Robbeets 2012, 2016). Even if such principles are not foolproof, they are useful diagnostics. To identify whether our agropastoral correspondence sets are more likely to be the result of borrowing or whether they could better be explained by inheritance, we applied the following seven criteria.

1 *Phonologically regular correspondences of bare verb roots that are well distributed in 3 or more subfamilies are likely to be inherited*.

This guideline follows from a combination of five observations: first, regularity of sound correspondence as an indication of inheritance; second, the hierarchical tendency that nouns are more easily and frequently borrowed than verbs; third, the typological feature that the Transeurasian languages display a clear preference for borrowing verbs adding a native suffix or light verb to the loan verb; fourth, the fact that most examples of borrowed verbs have a binary setting in common, typically going from a model language into a recipient language but rarely progressing into a third or fourth language and; fifth, borrowings often have a limited distribution within their subfamily.

On these grounds, for instance, we regard the Tungusic, Mongolic and Turkic words participating to the etymology of Proto-Altaic **tari*- ‘to cultivate’ in Section 2.1. (10) as cognates, in spite of previous claims that they are borrowed.

2 *Comparative sets that violate the regular sound correspondences established for each subsequent consonant and vowel of the root (except the root-final vowel), are likely to be inherited*.

The sound correspondences are given in Section 5. They are established on the basis of correspondences in the basic vocabulary, see SI 2. Comparative sets for agropastoral vocabulary that violate these sound correspondences are likely to be the result of borrowing. For instance, in Section 2.1. (1), Proto-Mongolic **atar* ‘unploughed field’ is dismissed as a cognate of Proto-Transeurasian **pata* ‘field for cultivation’ because the initial ø does not correspond with initial **p*- in Transeurasian.

3 *Comparative sets that consist of a morphologically complex word in one language that cannot be analyzed as such in the other language are likely to be the result of borrowing*.

The donor words for ‘sheep’ (Section 3.1 (5)), ‘fermented buttermilk’ (Section 3.1 (7)), ‘horse’ (Section 3.2 (1)), ‘made of iron’ (Section 3.3 (2)) and ‘vegetable jar’ (Section 3.5 (3)), for instance, are morphologically segmentable, while the recipient words are not. This can be taken as a strong indication of borrowing.

4 *If the correspondence set shares a meaning that is restricted to a secondary semantic development of one of the participating words, then it is likely to be the result of borrowing*.

In Section 3.1 (3), for instance, the Turkic donorword means any ‘non-castrated male’ including but not restricted to male horses, while the Mongolic and Tungusic borrowings are semantically restricted to ‘non-castrated male horse’.

5 *If the correspondence set shares a phonological property that is restricted to a secondary phonological development of one of the participating words, then it is likely to be the result of borrowing*.

In Section 3.1 (2), for instance, it can be argued on morphological grounds that pTk **budgay* is the original form for ‘wheat’ and that it underwent metathesis to become **bugday*. Since the Proto-Mongolic look-alike **bugudaï* ‘wheat’ only corresponds to the Proto-Turkic metathesized alternant, it is likely to be borrowed.

6 *If the participants of a correspondence set have a limited distribution in one of the subfamilies, they are likely to be borrowed*

The poor distribution of potential cognates of Proto-Transeurasian **simi*- ‘to soak (food)’ (Section 2.2. (8)) in Turkic and Tungusic, for instance, is a legitimate ground to dismiss the words from the etymology. Similarly, the limited distribution of the words for ‘ox’ (Section 3.1 (4)) and ‘mare’ (Section 3.2 (2)) in Tungusic supports their being borrowed.

7 *If the correspondence set corresponds to a probable donor word outside the Transeurasian family, then it is likely to be the result of borrowing.*

For instance, the availability of plausible donor words in Indo-European, Sino-Tibetan, Sinitic or non-Transeurasian substrate language in the etymologies for ‘barley’ (Section 3.1 (1)), ‘horse’ (Section 3.2 (1)), ‘mare’ (Section 3.2 (2)), ‘barley, wheat’ (Section 3.3 (1)), ‘silk’ (Section 3.4 (2)) and ‘metal cooking pot’ (Section 3.5 (4)) is indicative of borrowing.

The application of these guidelines to our database allows us to distinguish agropastoral correspondence sets that are likely to be inherited from those that are likely to be borrowed. By examining the cultural, temporal and spatial patterns that characterize these two sets, we are able to make inferences about environment, subsistence strategies and culture of the speakers of the proto-language.

**2 Inheritance going back to the Neolithic**

The origin and spread of Transeurasian subsistence vocabulary involve two major phases that mirror the origin and dispersal of agriculture in Northeast Asia.

The first phase, represented by the root and the primary nodes (i.e. Japano-Koreanic, Altaic and Mongolo-Tungusic) of the Transeurasian family, goes back to the Early to Middle Neolithic, when millet farming originated in the West Liao River area and spread across contiguous regions in Northeast Asia. A small portion of the subsistence vocabulary shared between the Japonic, Koreanic, Tungusic, Mongolic and Turkic languages can be traced back to a common ancestral root, i.e., Proto-Transeurasian or to its early descendants, i.e. Proto-Japano-Koreanic, Proto-Altaic and Proto-Mongolo-Tungusic. It concerns vocabulary for cultivation and harvesting activities, millets and durable wild food resources, food production and preservation such as fermentation, grinding and soaking, domesticated pigs and dogs, as well as spinning and weaving technology. This indicates that the speakers of Proto-Transeurasian and their early descendants in the Neolithic were sedentary, combined millet cultivation and pig-raising with collecting food in the wild and engaged in complex textile technology. Surprisingly or not, there are no cognate sets reflecting familiarity with rice or other cereals such as barley and wheat. A similar gap exists for dairying terminology and for vocabulary for domesticates such as horses, cattle and smaller ruminants. We can thus infer that the ancestral speakers of Transeurasian in the Neolithic were millet farmers that did not engage in the cultivation of rice, barley or wheat nor in pastoralism or dairying.

The second phase, represented by the individual daughter branches (Proto-Turkic, Proto-Mongolic, Proto-Tungusic, Proto-Koreanic and Proto-Japonic) goes back to the Bronze Age, when new crops such as rice, West Asian barley and wheat were introduced and new subsistence strategies based on dairying and pastoralism developed. This did not only change the agricultural package but it also impacted demographic and linguistic dynamics. Interestingly, a large part of the subsistence vocabulary shared between the Japonic, Koreanic, Tungusic, Mongolic and Turkic languages can be traced back to multidirectional ancient borrowing between the individual ancestral languages. As illustrated in Section 2, it concerns vocabulary for cereals such as rice, wheat and barley, more sophisticated agricultural tools, dairying activities and products, various domesticates such as horses, cattle, sheep and goats, the use of bronze and iron instruments and sericulture.

There is a clear distinction in cultural and semantic categories between inherited and borrowed subsistence vocabulary that mirrors the provenance and timing of the introduction of the corresponding agropastoral activities. For instance, millet and general cultivation terms are inherited, while words relating to rice, barley and wheat cultivation tend to be borrowed; basic agricultural tools such as spades are inherited while more specialized tools, some of which made of iron, are borrowed; words for domesticated animal names associated with agriculture in the Neolithic, such as dogs and pigs, are inherited, while pastoral animal names associated with the Bronze Age such as those for ruminants and horses are borrowed; words for fermentation in general are inherited, while words for dairying and milk fermentation tend to be borrowed or repurposed from vegetal fermentation terms within the individual histories of Proto-Mongolic and Proto-Turkic and; bast fibres such as hemp and ramie are inherited from Japano-Koreanic while silk terminology is borrowed individually into these languages from Chinese. These observations point to cultural and linguistic continuity in the Neolithic followed by massive cultural and linguistic interaction between speakers of Transeurasian as well as non-Transeurasian languages in the Bronze Age.

The early dating of the ancestral Transeurasian speech community at 9181 BP (5595 -12793 95% HPD) (SI 2) and its location in the West Liao area (SI 4) can be associated with the onset of sedentism and small-scale millet cultivation in the West Liao region and the transition to a significantly wetter climate, starting in the 9^th^ millennium BP (Shelach-Lavi et al. 2019, Stevens et al. 2020).

The root age of a language family corresponds to the time when subgroups of the original speech community became first separated. It is sometimes assumed that this event is always determined by migration or geographical separation. However, the trigger for the separation can be any factor that reduces the connectivity of the speakers – whether geographical, environmental or social: growing populations may result in the formation of environmentally or socially separated subgroups of speakers, who are no longer in frequent contact and thus start to innovate their language in different ways. Due to a slight increase in population densities (ED Fig. 3) and the resulting need of land for cultivation, the earliest farmers in the West Liao region became separated into local communities. The clock of language separation started ticking as soon as the connectivity between the speakers was broken, it did not wait until actual migration to new farming grounds took place.

Our ancient DNA analyses (ED Fig. 8, 10) indicate that contemporary Sino-Tibetan speakers can trace a large proportion of their ancestry to Neolithic Yangshao farmers and their ancestors, supporting recent associations between the onset of agriculture in the Yellow River region and the origins of the Sino-Tibetan family (Sagart et al. 2019; Zhang et al. 2020, Ning et al. 2020). Lack of evidence for Sino-Tibetan influence in the ancestral Proto-Transeurasian language is consistent with the multi-centric origins of early millet cultivation suggested in archaeobotany.^26^ With the exception of the words for ‘millet gruel’ and ‘spade’ in Section 2.1. (8) and (9), there are only very few cases of borrowing of Proto-Sino-Tibetan agricultural vocabulary into Proto- Transeurasian or *vice-versa*. However, there are some indications that the Proto-Transeurasian agricultural vocabulary was coined language-internally, repurposing native terms that originally had a non-agricultural meaning. For instance, the Transeurasian word **pisi*- in Section 2.1. (5) extended its original meaning from ‘to sprinkle with the hands’ to ‘to sow (seeds)’ and was then used to derive deverbal nouns such as ‘seed, seedling’ in (6) and ‘millet seed’ in (7). This observation supports an *in-situ* invention of agriculture in the West Liao River region in the Neolithic, relatively free from Yellow River influence.

In contrast with the language-internal development of agricultural vocabulary in the Neolithic illustrated in Section 2, the pastoral vocabulary and terms related to rice, barley and wheat agriculture in Section 3 were introduced through intensive borrowing across East Asia and the Eurasian Steppe in the Bronze Age. There is also some evidence for repurposing agricultural vocabulary as dairying terminology, such as the fermentation verbs in (1) to (4) that were recycled as dairying verbs in Mongolic and Turkic.

Why did the Neolithic expansion of millet farming cause pre-existing hunter-gatherers to abandon their native language and shift to the incoming Transeurasian target language, thus ensuring its continuity, while Bronze Age interaction involved maintenance of the native language with extensive borrowing of agropastoral vocabulary? In line with Renfrew’s (1987: 123–131) demography/subsistence model, the socio-economical context of the interaction may explain the outcome of the linguistic encounters. When farming is introduced to populations with relatively less successful subsistence strategies, we expect them to shift to the incoming culture and language because such a shift would involve a revolutionary potential for prosperity and demographic growth. By contrast, when groups of more or less equal socio-economic status meet, whereby certain crops are added to a pre-existing agricultural package or farming is complemented by pastoralism, the need to radically shift language and culture is less urgent. In such cases, the encounters are expected to result in borrowing.

The case of the spread of Koreanic and Japonic languages across the Korean peninsula represents a thought-provoking example of how (agri)culture may determine the linguistic outcome of interaction between speech communities. If millet agriculture and Hongshan genome were spread to the Peninsula with Proto-Koreanic languages in the Neolithic, while secondary crop agriculture and Upper Xiajiadian genome were spread with Proto-Japonic in the Bronze Age, it raises the question of why Koreans do not speak a Japonic language now. Why did Proto-Japonic not replace Proto- Koreanic on the Korean Peninsula? The answer to this question is probably determined by the socio-economical context of the interaction (Hudson & Robbeets 2020). When millet agriculture was introduced to the Korean Peninsula, farmers met hunter-gatherers. By contrast, when secondary crop agriculture was introduced, (rice, barley, wheat) farmers met (millet) farmers. From a socio-economical perspective, the first movement is more revolutionary than the second and will lead to language shift, while the second is expected to lead to linguistic borrowing between the pre-existing farmers and the incoming ones.

This explains why the Neolithic and Bronze Age migrations involved different linguistic dynamics and led to an interplay of two different outcomes, continuity and borrowing. This prehistorical layering of borrowed upon inherited words makes it difficult for historical linguists to distinguish between both transmission modes and is therefore at the base of the Transeurasian controversy.

**2.1. Reconstructions for cultivation**

The etymologies (1) to (9) below suggest that the speakers of Proto-Transeurasian were familiar with agriculture, with (7) and (8) suggesting that millet was the central cultivar. They illustrate a continuity whereby agricultural terminology was passed on from the ancestral language to the Neolithic daughter branches Proto-Japano-Koreanic, Proto-Altaic and Proto-Mongolo-Tungusic. Even if absence of evidence is not necessarily evidence of absence, it is interesting to note that a specific vocabulary dedicated to rice cultivation is lacking in Proto-Transeurasian as well as in all Neolithic daughter branches.

Etymologies (1) and (2) suggest that the ancestral Transeurasian speakers made an explicit distinction between ‘field for cultivation’ and ‘uncultivated field’ and (12) and (13) show that the distinctions for different field types increased among the speakers of Japano-Koreanic, suggesting an intensification of agriculture.

Etymologies (3) to (5) indicate that the ancestral Transeurasian speakers were involved in activities, such as ‘sowing’,‘planting’ and ‘growing’ crops. The derivation of ‘seed’ as ‘what one uses for sowing’ and ‘millet seed’ as ‘what is sown’ in the etymologies (6) and (7) implies that seeds were not merely collected and consumed but planted and used for cultivation.

Etymology (3) further suggests that the ancestral speakers of Transeurasian repurposed a pre-existing verb for ‘sprinkling’ as a verb for ‘sowing’, which supports the independent development of agriculture, relatively free from external influence. Nevertheless, the etymologies in (8) and (9) indicate that Proto-Sino-Tibetan speakers living in the Yellow River area before 8000 BP exchanged agricultural knowledge with the speakers of Proto-Transeurasian in the West Liao River area by transferring the practice of preparing millet gruel and the use of certain agricultural tools such as a particular kind of spade. Between 8000 and 5500 BP, there was also contact between speakers of ancestral Sinitic and speakers of Proto-Japano-Koreanic situated on the Liaodong Peninsula, resulting in the transfer of additional Sinitic agricultural vocabulary, as illustrated by etymology (12).

**(1) Proto-Transeurasian **pata* ‘field for cultivation’**

Proto-Japonic **pata* ‘(dry) field’ + *-*ka* place suffix, *-*i* substantivizer

J *hata*, OJ *pata* ‘(dry) field’, J *hatake*, OJ *patake_2_* ‘field, farm, plantation, garden’, Miyazaki *hatsuke*, Kagoshima/ Koshiki/ Kumamoto*/*Ōita*/*Fukuoka*/*Saga*/*Fukue*/*Nagasaki *hatake* ‘agricultural field’, Yamatohama (Amami) *hathe*, Naze (Amami) *hatǝǝ*, Asama (Amami) *hatëë*, Yoron (Amami) *pattai*, Yonamine (Okinawa) *patʔaakʔi*, Shuri (Okinawa) *hataki*, Old Shuri *hataki*, Ishigaki (Yaeyama) *patagi*, Hateruma (Yaeyama) *pïte:gi,* Hatoma (Yaeyama) *pataki,* Oura (Miyako) *patagi*, Yonaguni *hatagi,* PR **patake* ‘field, croft’

Proto-Koreanic **patʌ* ‘(dry) field’ (+ *-(*ɨ/ʌ*)*k* place suffix)

MK *path* ‘(dry) field, field for cultivation farm, patch, garden’, K *path* ‘a (dry) field, a farm, a patch, a garden, an orchard; a piece of ground where a particular plat grows thickly; a patch; a position on a game-board’, JJ *was ~ pas* ~ *phas*, JJ *pas* ~ *phas* , KB *path* ~ *pach* ~ *pas*, KN *path*, JN *pas* ~ *path*, JB *pathtayki*, KW *pas*, CN *path*, CB *path*, KG *pas* ~ *path* ‘(dry) field, garden’.

Proto-Turkic **(p)ata* ‘delimited field irrigated for cultivation’

+ *-*(A)g* place suffix in MTk. (Middle Kipchak) *atov* ‘island’, MTk (Chagatay) *adaq* ‘island (overgrown with plants), an island with vegetation’, Chu. *udă* ‘island; a grove in a field; meadow; ravine, valley; locality; site, sector’, Tk. *ada* ‘island’, Gag. *ada* ‘cape; island; (*rarely*) swamp, bog, marsh’, Az. *ada* ‘island’, Tkm. *a:da* ‘island’, Karaim *ada* ‘island’, Kumyk *ataw* ‘island’, Uig. *ata* ‘island’, KKalp. *ataw* ‘island’, Bash. *ataw*‘island; a clearing in the wood, meadow, grassplot’

+ *-*r_2_* collective suffix in OT (Karakhanid) *atïz* ‘any strip of land between two dikes’, *atïzla*- ‘to create irrigation ditches (for cultivation)’, MTk. *atïzla*- ‘to create an irrigation canal in a field’, Uig. *etiz* ‘field, cornfield; arable land’, Tkm. *atïz* ‘furrow; strip of land’, Shor *adïz* ‘a measure for fields, 1/18 dessiatin (= ca. 607 square meters)’, Kirg. *adïr* ‘hilly terrain, hill ridge, highlands’, Kaz. *atïz* ‘a plot of land, watered by irrigation canals and properly limited’, *adïr* ‘hillock; highlands, hilly terrain’

The Proto-Turkic place suffix *‑*(A)g* is petrified, for instance in PTk **o:t* ‘fire’ → *o:t-ag* ‘tent, dwelling place’. The Proto-Turkic collective suffix *-*r_2_* is petrified in Old Turkic, for instance, in paired body parts such as OT *kö-z* ‘eyes’, *ti-z* ‘knees’, *agï-z* ‘lips’ and *kökü-z* ‘breasts’, ethnonyms such as OT *ogu-z* and *kïrgï-z,* sets of more than one such as *iki-z* ‘twins’, *üc-üz* ‘triplet’, *dörd-üz* ‘quadruplet’ and undefined quantities such as OT *yultu-z* ‘stars’, *yïldï-z* ‘roots’.

For Turkic, it is commonly assumed that word initial PTk **p*- developed over a bilabial fricative into *h*-, leaving only a trace in Khalaj *h*- and finally disappearing in most of the contemporary Turkic languages. There are only a few cases in which Khalaj *h*- supports the reconstruction and comparison of PTk **p*-, e.g. OT *adaq*, Khalaj *hadaq* ‘foot’ < PTk **pada-k* (compare K *patak*, MK *pa·taŋ*, *pa·twok* ‘bottom’ < **pata*-) or OT *ör*-, Khalaj *hör*- ‘to plait’ < PTk **pö:r*- (compare Khalkha *oro:*-, Mgr. *furo:*-, MMo. *hura*- < PMo **poro*- ‘to entwine’ and Evk. *horol*- ‘to spin’, Ma. *foro*- ‘to turn round’ < PTg **poro*- ‘to spin, weave’). When a Khalaj cognate is missing, we perceive a correspondence between initial ø in Turkic and PJ **p*-, PK **p*-, PTg **p*- or PMo **p*-, as is the case here. The initial *(*p)* in the Proto-Turkic reconstruction **(p)atï* ~ **(p)ata* is bracketed because the alleged loss of the initial labial stop **p*- cannot be confirmed since we lack a Khalaj cognate.

In Mongolic, we find Written Mongolian *atar* ‘virgin land, unploughed or fallow field’, Kalkha *atar*, Buriat *atar* and Monguor *atǝr*. Given the poor distribution of the word, the lack of evidence for initial **p*- and the lack of morphological segmentability, the Mongolic words are likely to be borrowed from Turkic.

**(2) Proto-Transeurasian **muda* ‘uncultivated field’**

Proto-Japonic **muta* ‘uncultivated land, marshland’

J (dial.) *muta* ‘swamp, marshland’, Miyako *muta* ‘land’, Shodon *mutha* ‘swamp’, Old Shuri has *mitʃa*: ‘swamp, marshland’, Hirara *mta*, Nagahama *mta*, Tarama *mta*, Ishigaki *Nta*, Yonaguni *Nta* ‘swamp’, PR **muta* ‘swamp, marshland’

Proto-Koreanic **mutʌ* ‘dry land’ + *-(*ɨ/ʌ*)*k* place suffix

K *muth*, MK *muth* ‘land, dry land’, JJ *mus*, KB *mwuch*, KN *much*, JB *mus*, CB *mwus*, CN *mwus*, KG *mwuch*, KW *mwus*

Proto-Tungusic **muda* ‘plain, open field, highland’

Na. *mudũ* ‘meadow, area for handicraft’, Kur-Urmi Na. *mudũ* ‘highland along a riverbank’,

Evk. *mudangna* ‘top; cape’

**(3) Proto-Transeurasian **iuse*- ‘to plant, grow (plants)’**

Proto-Koreanic **yes*- ‘to grow grain’ + *-*(ʌ/ɨ)k* > **isak*, *-*(ʌ/ɨ)k* edible plant suffix, e.g., MK *chulk* ‘kuzu (arrowroot, edible)’, MK *a·wok* ‘marshmallow (Althaea officinalis’), MK ·*milh* ‘wheat’, MK ·*phoch* ~ ·*phosk*  ‘red bean’, etc.

K *yes*, MK ·*yes* ‘taffy; sweet, sticky food, created by first cooking cereal, fermenting it with malt, boiling it into liquid, putting it into a sack, pressing it out, and simmering into a sticky state’, MK *isak* ‘ear of grain’, K *isak* ‘ear of grain, head, spike; shuckings’, KW *isulak*, *isilak*, KB *isak* ~ *isayk* ~ *isiki* ~ *isik*, JN *isilak*, JB *isayki* ~ *isulak* ~ *pey isak*, KW *isulak* ~ *isilak*, CB *isak*, KG *isak* ‘ear of grain, head, spike; shuckings’

Proto-Tungusic **üse*- ~ *üsi*- ‘to plant' +-*i* deverbal noun / + *-*n* deverbal noun

→ **üse* ~ *üsi* 'seed, seedling' / **üsi-n* 'field for cultivation'

Evk. *ihəw-* ‘to grow (of people)’; Even *isu:-* ~ *esu:*- 'to sprout, come out (of plants), blossom, grow', *isu:ce:* 'young sowings, seedling, sprout', *isuwken* ‘the planting’, *isuwken-* ‘to plant’, *isulmən* ‘sprouting’, *isumə* ‘sprout; teenager’, *isun* ‘growth’; Neg. *isew*- ‘to grow, become acclimatized (about plants); mature, grow up (about people)’; Sibe *use-* ‘to sow seeds’, *use* ‘seed, grain’, *usin* ‘field, farmland’; Ma. *use*- 'to plant, seed (tr.)', *use* 'seed; insect egg', *use tari*- 'to sow seed', *use use*- 'to plant seed', *usin* 'field for cultivation', *usisi* 'farmer'; Jur. *use* 'seedling' (*t’éh-léh-t’ūn-méi wúh-séh-t’iēn* ‘particular species’), *usi-in* 'field' (*wúh-šīh-yīn* ‘field’), *usi* 'field'; Olcha *use* 'seed', *usun* ‘field, garden’; Na. *use* 'seed', *usĩ* ‘arable field for cultivation; private garden for cultivating vegetables’, *usiŋku* ‘person who possesses such a garden’; *usilə-* ‘to labour a land for cultivating plants’; Orok *usi* ‘field (farm), garden’, *usi-* ‘cultivate, till; hunt a bear’; Oroch *usi* ‘seeds, grains’, *usin* ‘garden for cultivating vegetables and plants’, *usin-* ‘to sow, to plant in a garden’; Ud. *jehu*- ~ *jiu*- ‘to grow (about plants, people); to grow (about the moon)’, *uhi-* ‘to sow, to plant a garden’, *uhi* ‘garden for cultivating plants’

Proto-Mongolic **ös*- ‘to grow (of plants/animals)’

WMo. *ös*- 'to grow, multiply, increase (intr.)', *öske*- ‘to grow, raise, breed; to increase, multiply (tr./ causative of *ös*-)’.MMo. *ös*-, *us*-, *os*- ‘to grow (intr.)’, *ösge*-, *osge* ‘to grow (plants), breed (animals) (tr./ causative)’, Khal. *ös*-, Bur. *üde*-, Kalm. *ös*-, Ordos *ös*-, Dongxian *osǝ*-, *osɨ*, Baoan *ose*-, Dagur *euse*-, Eastern Yugur *ǖs*-, Monguor *ōsǝ*-

Proto-Turkic **ös*- ‘to grow (of plants/animals)’

OT *ös*- 'to grow, increase in size (intr.)', MTk *ös*-, Turkish *ös*-, Tatar *üs*-, Uzbek *ụs*-, Uighur *ös*-

Turkmen *ös*-, Khakas *ös*-, Shor *ös*-, Oirat *ös*-. Tuva *ö's*-, Kirghiz *ös*-, Kazakh *ös*-, Noghai *ös*-, Bashkir *üɵ*-, Balkar *ös*-, Karaim *ös*-, Karakalpak *ös*-, Kumyk *ös*-

**(4) Proto-Transeurasian **urə*- ‘to grow, ripen (of plants)’**

Proto-Japonic **ura*-‘to mature, ripen (of plants)’ + *-*(C)i*- causative-anticausative

J *ure*-, OJ *ure*- (B) ‘to mature, ripen (of plants)’, Kagoshima *uruʔ*, Miyazaki*/*Kumamoto*/* Ōita*/*Fukuoka *ururu,* Saga *uruʔ,* Nagasaki *ururu* ‘to ripen (of crops, fruits)’, Hatoma ʔurin ‘to ripen’

Proto-Tungusic **ure*- ‘to grow, ripen (of plants)'

Evk. *uruktu* ‘bush’, Sol. *urilə* ‘grain’, Ma. *ursan* ‘new shoots that sprout from old roots, new branches that appear on a tree that has been cut away’, *ursana*- ‘to sprout from old roots or an old stock’, Sibe *ursan* ‘seedlings, bystander branches’, *ursa-na*- ‘to revive’, Jur. *ure*- ‘to ripe’, *uri*- ‘to become ripe’, Na. *urə*- ‘to grow, ripen; to develop’, *urə-ktə* ‘willow rod’, Olcha *urə*- ‘to grow’, *urə-qtə* ‘a rod’, Orok *urəktə* ‘a rod’

Proto-Mongolic **ur-ga-* ~ **urgu*- 'to grow (of plants)' (WMo. -*GA*- < pMo *-*gA* factitive)

WMo. *urɣu*- 'to grow, sprout; to appear, show up; to rise (of sun) (intr.)', *urɣuca* 'harvest, yield, crop', MMo. *urqu*-, *hurɣa*-, *urɣu*- '1 to grow', Khal. *urga*- '1', Bur. *urga*- '1', Kalm. *urɣǝ*- '1', *urɣǝmǝr* 'harvest, yield, crop', Ordos *urGu*- '1', Dag. *orgu*-, *orege*-, *orgo*- '1', Mog. *urɣu*- '1', Eastern Yugur *uğarma* 'plant' (< **urga-ma*)

The initial *h*- in the Middle Mongolian form *hurɣa*- in the Muqaddimat al-adab is

secondary since *h*- before consonant clusters is found to be a quite a common phenomenon in Muqaddimat al-adab (Gruntov 2005). The Mongolic forms of the shape **ur-ga-* 'to grow' can be derived as a factitive derivation of an original root pMo **ur*- 'to grow', while the forms of the shape **urgu*- 'to grow' could represent an assimilation of the stem-final vowel to the preceding one. However, the derivation with a factitive suffix remains problematic because the resulting verb is not transitive.

pTk **ur* ‘growth, excrescence'

Tk. *ur* '1 growth, excrescence', Az. *ur* 'burl, wart (on a tree); tumor; crop', Tat. *ŭrɨ* '1', Kirg. *ur* 'burl, wart (on a tree), Bash. *ŭrŭ* 'burl, wart (on a tree); bump; nodule; outgrowth, excrescence', Nog. *urɨ* 'gland; excrescence', Kaz. *ŭra* 'bump, excrescence', Tuva *uru* '1', Shor *ur* '1', Oyrat *ur* '1', Yak. *ur* '1'

**(5) Proto-Transeurasian **pisi*- ‘sprinkle with the hands, sow’**

Proto-Koreanic **pis*- ‘to sprinkle, scatter, sow’

K *ppu:li*- ‘to sprinkle, rain slightly (intr.); to sprinkle, shower, water (tr.); to scatter, sow’, K *ppuli* ‘a root (of a plant)’, MK *spu·li*- ‘to sprinkle’ (MK -*(u)li*- transitivizer), MK *spih*- ‘to sprinkle; slander’

Proto-Tungusic **pisi*- ‘to sprinkle with the hands’

Ma*. fisi*- ‘to sprinkle with the hands; to shake, to toss (one’s sleeves)*,* Olcha *pisuri*- ‘to sprinkle’, Orok *pisitči*- ‘to sprinkle’, Na. *pisi*-, *fisi*- ‘to sprinkle’

Proto-Mongolic **pesü-*/**pisü-* ‘to sprinkle, scatter; jump around’ + *-*r*- intensive

Dag. *xǝsur*-, *xesurǝ*- ‘to sprinkle’, Mgr. *fiʒuru*- ‘to sprinkle, pour, cast (metal)’

Although the meaning ‘to sow’ is only explicitly attested in the Korean verbs, it can be reconstructed to the ancestral language, given the derived nouns for ‘seed, seedling’ and ‘millet seed’ in the following etymologies (4) and (5). The polysemy between ‘sprinkle’ and ‘sow’ is recurrent throughout the Transeurasian languages, including verb roots that are not cognate to the root under discussion such as Japanese *maku* ‘to sprinkle, scatter, strew, sow (seed)’, *hodokosu* ‘sprinkle, scatter, sow; give, perform, apply’, Sibe *swata*- ‘to sprinkle, sow’, Turkish *sač*- ‘to sprinkle, scatter, sow (seed)’, *ek*- ‘to sprinkle, scatter, drop, throw about, sow (seed)’, etc. Therefore, we can argue that the speakers of Transeurasian extended their verb for ‘to sprinkle (with the hands)’ to ‘to sow (seeds)’ aas they became familiar with plant cultivation.

**(6) Proto-Transeurasian **pisi*-*i* (sow- INS.NMLZ) ‘seed, seedling’**, *-*i/ø* instrumental deverbal noun suffix

Proto-Koreanic **pisi* ‘seed; lineage’

MK ·*psi* ‘(plant) seed, the hard substance within a fruit of a plant, which will sprout and grow into a new plant; the source of bearing and reproducing new animals; derogatory term for the blood or the origin of a family’, K *ssi* ‘seed, kernel, stone, pip; lineage, descent, breed, stock’, K *pye-psi* ‘rice seed’, JJ *ssi*, KB *ssi*, KN *ssi* ~ *ssikas*, JN *ssi* ~ *ssikas*, JB *ssikasi*, KW *ssi*, CN *ssi*, CB *ssi*, KG *ssi* ‘seed’

Proto-Tungusic **pisi-ke* ‘broomcorn millet (*Panicum miliaceum*)’ < **pisi*- ‘to sprinkle with the hands, sow’ + *-*xa* ~ -*kA* deverbal resultative nominalizer (Robbeets 2015: 402-408)

Ma. *fisihe ~ fisike* ‘glutinous millet, broomcorn millet (*Panicum miliaceum*)’, *fisitun* ‘a ritual vessel for offering millet; bowl for grinding millet, carved out from a piece of wood’ (< *fisi* + *tetun* ‘utensil’); Olcha *pikse;* Na. *pikse* ‘millet’; Kur-Urmi dialect *fisxe* ‘broomcorn millet (*Panicum miliaceum*)’; Jur. *fise bele* ‘yellow rice; coarse rice’ (*bele* ‘hulled rice, edible grain’)

Proto-Mongolic **pesi* ~ **pisi* ‘origin or base of a plant’

MMo. *nisi*, *hesi,* Written Mongolian *isi* ~ *esi* ‘foundation, basis, origin, source; a stalk of grain, trunk of a tree, stem of a plant, shoot; handle, grip’, Khal. *iš* ~ *eš* ‘1 source, basis; 2 stem, stalk, trunk, underground stem; 3 handle, shaft’; Bur. *eše* ‘1, 2, 3’, Kalm. *iš* ‘beginning, source; stalk (of plant), stem (of tree); handle, grip’, Ordos *eši* ~ *iši* ‘1, 2 , 3’, Bao. *jɛśi*, *heʂï* ‘handle, grip’, Dag. *xeš*, *xeši*, *heši* ‘handle, grip, knob’; Eastern Yugur *šǝ* ‘handle, stem’, Kangjia *heši* ‘handle, grip’ (Nugteren 2011: 354)

The derivation of Proto-Tungusic **pisi-ke* ‘broomcorn millet (*Panicum miliaceum*)’ as ‘what is sown’ recalls the derivation of Old Tukic *tarïg* ‘grain (generic); crop; cultivated land’ and Middle Turkic *tarïg* ~ *tarïḳ* 'cultivated field; millet' from the Old Turkic verb *tarï-* ‘to disperse, cultivate’, see (10). The suffix deriving the resultative noun from the verb is attested in numerous petrified pairs in Tungusic, such as Even *ewi:-* ‘to play’ → *ewi:ke:* ‘game, toy’, *te:w*- ‘to put’ → *te:pke* ‘cover’, *et*- ‘to conquer’ → *etka* ‘regime, power’, *hi:l*- ‘to suffer’ → *hi:lka* ‘suffering, agony’, Evenki *kalta*- ‘to split in halves’ → *kaltaka* ‘one of a pair’, *sukca*- ‘to ruin’ → *sukcaka*: ‘ruin’, etc. (Robbeets 2015: 402-408)

**(7)** **Proto-Transeurasian **kipi* ~ *kipe* ‘components that are removed from the grain harvest, barnyard grass (*Echinochloa crusgali*)’**

Proto-Japonic **kinpi* ~ **kimi* ~ **kipi-mi* ‘millets for human consumption such as barnyard millet (*Echinochloa esculenta*), broomcorn millet (Panicum miliaceum)’

J *kibi* (2.5), EMJ *kibi*, OJ *ki_1_mi_1_* ‘broomcorn millet (Panicum miliaceum)’, Hachijo/ Sodegaura *kimi* ~ *kibi* ‘broomcorn millet (Panicum miliaceum)’, Kagoshima *kiʔ*, Koshiki *ki(ː)bi*, Miyazaki/ Kumamoto/ Fukoka/ Saga/ Fukue/ Nagasaki *kibi* ‘broomcorn millet (Panicum miliaceum)’, Tokunoshima *kimi* ‘broomcorn millet (Panicum miliaceum)’, Wadomari (Okinoerabu) *kimi* ‘broomcorn millet (Panicum miliaceum)’, China (Okinoerabu) *kimii* ‘broomcorn millet (Panicum miliaceum)’, Hirara *tsïm* ~ *kïm* ‘barnyard millet’, Ogami *kïm* ‘barnyard millet’, Tarama *kïm* ‘barnyard millet’, Ishigaki *pi: ~kïŋ* ‘barnyard millet’, *kïŋ* ‘broomcorn millet’, Hateruma *sïN* ‘barnyard millet’, *sïŋ* ‘broomcorn millet’, Kohama *kïn* ‘barnyard millet’, Taketomi *ʃiŋ* ‘broomcorn millet’, Yonaguni *c'iNti* ‘barnyard millet, broomcorn millet’

Proto-Koreanic **kipi* > **phi* ‘barnyard millet (*Echinochloa esculenta*)’

MK ·*phi* ‘barnyard millet; It is about a meter high. It has thin, long leaves whose surface covers the stems like a sheath. During the summer, light green or purplish brown flowers bloom as panicles and seeds are born as awns. The seeds are eaten or used as fodder’, K *phi* ‘(Japanese) barnyard millet (*Echinochloa esculenta*)’, JJ *phi* , KB *phi*, KN *phi*, JN *phi*, JB *phi*, KW *phi*, CN *phi*, CB *phi*, KG *phi* ‘barnyard millet’

Proto-Tungusic **kipe* ‘components that need to be removed from the grain harvest, barnyard grass (*Echinochloa crusgali*)’

Manchu *hife* ‘barnyard grass (*Echinochloa crusgali*), tares, darnel (Lolium temulentum: “false wheat”)’

Proto-Turkic **kẹpek* ‘bran (from millet, barley), chaff’

OT *kepek* ‘bran’, MT *kebek* ‘bran’, Az. *käpäk* ‘bran, chaff, dandruff, sawdust’, Bash. *käbäk* ‘chaff, empty glume, dial. bran’, CTat. *kepek* ‘bran, dandruff’, Gag. *kepek* 'bran', Kaz. *kebek* ‘bran, husk, chaff’, Khak. *kibek* ‘egg-shell, nut-shell, cast-off (of hay, straw), dial. bark’, Kirg. *kebek* ‘husk, chaff (from millet, barley, etc.), bran’, KKalp. *kepek* ‘husk, chaff. bran (from millet, barley, etc.)’, Kum. *gebek* ‘bran’, Nogh. *kebek* ‘millet husk’, Tatar *kibɛk* ‘chaff’, Tur. *kepek* ‘bran, dandruff’, Tkm. *kepek* ‘bran, bran siftings’, Tuv. *xevek* ‘millet flour’, Uigh. *kepɛk* ‘bran’, Uzb. *kepak* ‘bran’, Chu. *kibek* ‘husk, chaff, peeling skin on face’, *kibengə* ‘husk’

*Echinochloa esculenta* is referred to by the common names (Japanese) barnyard millet or Japanese millet. It is still cultivated on a small scale in Japan, Korea and China as food for humans or animal fodder. It is grown in areas where the land is unsuitable or the climate too cool for paddy rice cultivation. Small-scale cultivation of domesticated barnyard millet in Japan goes back to around 4000BP, during the Jomon period. The wild ancestor of *Echinochloa esculenta* is *Echinochloa crusgali*, which is known as barnyard grass. As it causes forage crops such as broomcorn millet to fail by removing nitrogen from the soil, it is considered as a weed and thus a component that needs to be removed from the grain harvest.

The meaning of Early Middle to contemporary Japanese *kibi* and OJ *ki_1_mi_1_* is restricted to ‘broomcorn millet (*Panicum miliaceum*)’, while the Ryukyuan languages combine the meaning ‘broomcorn millet (*Panicum miliaceum*) and barnyard millet (*Echinochloa esculenta*)’. According to the 18th-century dialectal wordlist Butsurui Shooko, Modern Japanese *kimi* has shifted its meaning to 'maize, sorghum' in southern Tohoku. An interesting meaning differentiation is found in the modern Fukushima dialect in Tohoku, which has *kɨbɨ* for the plant and *kɨmɨ* for the grain of broomcorn millet. This might be a Fukushima innovation, but the meaning distinction raises the possibility of a morpheme boundary in Proto-Japonic, involving pJ *-*mi*, deriving from OJ *mi_2_*, J *mi* ‘fruit, nut, kernel’.

In Japanese *hie*, OJ *pi_1_ye* is used to refer to (Japanese) barnyard millet (*Echinochloa esculenta*) but Ryukyuan cognates such as Yamatoma *hwei*, Asama *hui* are used in reference to the wild ancestor barnyard grass (*Echinochloa crusgali).* Except for Miyazaki *hiye*, which can refer to both the wild and the domesticated variety, all the Kyushu dialects, i.e. Kagoshima *hie*, Koshiki (Kamikoshiki) *hiye*, Kumamoto *hiye*, Ōita *hiye*, Fukuoka *hie*, Saga *hie*, Fukue *hie* and Nagasaki *hie*, are restricted to the meaning ‘wild barnyard grass (*Echinochloa crusgali)’.*

This observation suggests that Proto-Japonic made a distinction between **kinpi* ~ *kimi~ *kipi-mi* ‘millets cultivated for human consumption such as broomcorn (*Panicum miliaceum)* and barnyard millet (*Echinochloa esculenta)*’ and **piyai* ~ **piyia* ~ **piye* ‘weeds on the grain field such as barnyard grass’. In addition, Proto-Japonic had a separate word **apa* for ‘Foxtail millet (*Setaria italica*)’.

Given that the Korean forms underlying Proto-Koreanic **kipi* > **phi* ‘barnyard millet (*Echinochloa esculenta*)’, the most parsimonious reconstruction for Japano-Koreanic is **kipi* ‘barnyard millet’. The semantics of the Tungusic and Turkic reconstructions point to a common Altaic form meaning ‘unwanted part of the millet production, barnyard grass’. Because of the mismatch in the vowel, it is not clear whether the Turkic participant belongs here. However, (millet) bran refers to components that are removed from the grain or millet harvest and thus makes a good semantic fit with barnyard grass. Therefore, the original Transeurasian meaning may have referred to the wild variety ‘barnyard grass’ and innovated as the domesticated variety ‘barnyard millet’ in Japano-Koreanic.

**(8) Proto-Transeurasian **amʊ* ‘cooked cereal, millet gruel’**

Proto-Japonic **amai* ‘cereal starch’

OJ *ame_2_* ‘gluten; starch from cereals used to hold foods together’, Tarama *ami*, Ishigaki *ami* ‘gluten’

Proto-Koreanic **amʌ* ‘cooked cereal’

K *a:m, a:m cwuk,* MK *am*, *amcywuk* 'cooked cereal; thick rice soup', KN *amcwuk*, JN *mam* *cwuk*

The long vowel in the contemporary Korean form indicates that earlier in Middle Korean the word had a rising tone and supports a contraction of an originally disyllabic shape with a final reduced vowel **amʌ*.

Proto-Mongolic **amu-n* ‘cooked cereals; millet’, **amu-sun* ‘various cereal dishes; cooked rice’, **narin amun* ‘broomcorn millet (*Panicum miliaceum*)’ (**narin* ‘small, fine’)

MMo. *amu(n)* ‘millet’, *amusun* ‘cooked cereal’ ; WMo. *amu(n)* ‘grain, cereals’, *amusun* ‘cooked cereal, porridge; food offering made to spirits’; Khalkha *amuu* ‘millet; millet groat; groat; cereal; grain’, *lianguu amuu* ‘white millet’, *mongol amuu* ‘Monghol millet’, *tutarga amuu* ‘rice, rice groat’, *xonog amuu* ‘fine millet’, *ams* ‘cooked cereal’; Bur. *amha(n)* ‘cooked cereal’, Ordos *a(:)mu* ‘broomcorn millet (*Panicum miliaceum*)’; Dagur *am* ‘grain, cereal; (in compounds only), *am budaa* ‘grain, groat, food’, *maŋŋələm* ‘broomcorn millet’ (< **moŋgal amun*), *narye:m* ‘millet’ (< **narin amun*); Eastern Yugur *amən* ‘grain, cereal’, *aməsən* ‘cooked cereal’, *narən amən* ‘millet’; Monguor *a(:)mu*; Bao. *amuŋ*; Kangjia *amu* ‘millet’; Dgx. *aməŋ* ‘rice gruel’(Nugteren 2011: 268).

Manchu *amsun* ‘offerings of wine and food to a deity’ is a borrowing from the Mongolic form derived with a collective suffix.

Borrowing

**Proto-Koreanic **amʌ* ‘cooked cereal’ >> Proto-Ainuic **ɒm* 'cereals, such as rice, millet, wheat, barley'**

Proto-Ainuic **ɒm(=)ɒm* 'cereals, such as rice, millet, wheat, barley': Yakumo (South-West Hokkaido Ainu) *am=ám* 'cereals, rice', Obihiro (North-East Hokkaido Ainu) *amám* 'cereals, rice', Raichiska (Sakhalin Ainu) *am=am(=uhu)* 'rice', Kuril Ainu *am=ama* 'rice' (Vovin 1993: 82). The consonant-final form and the meaning ‘cereals including rice’ in Ainu suggests that the word is more likely to have been borrowed from Proto-Koreanic than from Proto-Japonic.

**Proto-Sino-Tibetan **am* ‘to eat/drink (liquid food such as gruel); liquid food’ >> Proto-Transeurasian **amʊ* ‘cooked cereal, millet gruel’**

Proto-Tibeto-Burman **am* ‘eat, drink; food’ > Proto-Tibeto-Burman **am* ‘rice plant'

Dhimal am ‘to drink’, Thulung (Western Kiranti) um- ‘eat’, Anong (Nungic) am ‘eat’, Karen (Karenic) *ʔam*^2^ ‘eat’, Bue (Karenic) a^33^ ‘eat’, Kayan (Karenic) *ʔaŋ^11^*‘eat’, Kayaw (Karenic) *ʔo^11^*‘eat’, Pa-O (Karenic) *ʔam*^2^ ‘eat’, Pwo (Karenic) ʔà*n* ‘eat’, Taungthu *ʔam*‘eat,’ Lepcha *am* ‘food,’ Rawang Mutwang dialect *əm* ‘eat,’ Lushei (and general Kuki) *in* ‘drink’

(Matisoff n.d.)

Bengni (Tani group) am ‘rice plant’, Dulong (a.k.a Trung, Nungish group) am^55^ ‘rice’ (paddy), Sak (Sal group) *aŋ* ‘rice plant’ (Final *-m regularly shifts to -ŋ in Sak), Jingpo (Sal group) mam³³ ‘rice (paddy)’, Chepang ʔamʰ ‘cooked rice’, yam ‘rice plant’, Thulung (Kiranti) mam ‘grain of rice remaining unhusked after milling’ (Sagart 2018)

Proto-Sinitic **am* ‘to drink liquid food’ > **am* ‘rice gruel’

Old Chinese 飲 *ʔi̯əm* ‘drink’ (Baxter and Sagart 2014)

Minnan *am*^3^ 'rice gruel'

**Proto-Sinitic **am* ‘drink liquid food, rice gruel’ >> Proto-Austronesian **am* ‘rice gruel’**

Proto-Austronesian **am* ‘rice gruel’ (Sagart, pc)

Bunun *am* ‘water from cooked rice’, Kavalan *am* ‘rice gruel’, Thao *am* 'rice gruel; water from cooked rice; slop, leftover food that is left to the pigs', Amis *ʔaʔam* 'soft watery rice'.

Along with Proto-Transeurasian **sarpa* ‘spade’ in the following etymology (9), the Proto-Transeurasian reconstruction **amʊ* ‘cooked cereal, millet gruel’ is one of the few Neolithic subsistence terms that may have been influenced by Proto-Sino-Tibetan. Given the meanings ‘eat/drink’ in Tibeto-Burman languages and the meaning ‘drink’ in Old Chinese, the original meaning of the Sino-Tibetan root can be reconstructed as ‘to eat/drink liquid food such as gruel’ and by extension ‘liquid food such as gruel’. The nominalized meaning served as a model, not only for Proto-Transeurasian, but also for Proto-Austronesian. The Proto-Transeurasian word can be reconstructed as ‘cereal/ millet gruel’ and may have been borrowed at a time before the introduction of rice agriculture in the Yellow River region, i.e. before 6000 BP and probably also before the break-up of Proto-Sino-Tibetan in 8000 BP (Zhang et al. 2020). Since the Austronesian words all share the element ‘rice’, they go back to a time after 6000 BP, thus postdating the break-up of Proto-Sino-Tibetan. The contact probably took place at a time when Austronesian was still spoken on the continent, i.e. before 5000 BP.

**(9) Proto-Transeurasian **sarpa* ‘spade’**

Proto-Koreanic **salp(V)* ‘spade’

Paekche OK **sap* ‘spade’ (Bentley 2000), MK ·*salp* ‘spade, shovel; a tool for digging earth and scooping soil’, K *sap* ‘shovel, spade’, HN *sakkalay*, CN *sakkaley*, KB *sakkaylay*, PB *sanci*, HN *sapcang*, KW *saph* ‘shovel, spade’

Proto-Turkic **sarpan* ‘wooden plough (breast), spade’

OT (Karakh.) *saban* ‘plough, cultivating the land’, MTk (Codex Cumanicus) *saban* ‘plough, ploughed land’, Az. *sapan* 'a sort of wooden plough', Bashk. *haban* 'plough, plowing', Crim Tat. *saban* 'plough', Gag. *saban* 'wooden plough', Kaz. *saban* ‘plough, ploughland, ploughed field’, KBalk. *saban* 'plowing', Kar.  *saban* ‘(wooden) plough’, Kum. *saban* ‘plough, ploughed land’, Nogh. *saban* ‘plough, ploughing’, Sal. *sovan* ‘wooden plough’, Tat. *saban* ‘plough, ploughing, spring-planted’, Tur. *saban* ‘(wooden) plough’, Uig. *sapan* ‘wooden plough’, Chu. *sorban* ‘plough breast’

Proto-Sino-Tibetan **tsrop* ‘spade’ >> proto-Transeurasian **sarpa* ‘spade’

Old Chinese *[tsʰ]<r>op ‘pestle’ (Baxter & Sagart 2014)

Mpi *tɕoʔ³* ‘dig with a spade’, Lahu *tshᴀ⁵⁴* ‘spade’, Pumi *tʃhɛ̃³³ tsə⁵⁵*‘spade’, Thado *suʔ* ‘spade’, Tiddim *suk^3^* ‘spade’

The Turkic and Koreanic forms correspond regularly, including a labial cluster correspondence. Given the Chuvash meaning ‘plough breast’, the Proto-Turkic meaning may be specified as the spade that is attached to a plough handle. Therefore, we reconstruct the original Transeurasian meaning as ‘spade’. Formally, Old Chinese **[tsʰ]<r>op* could serve as a plausible model for Turkic and Koreanic, but the meaning of this word is ‘pestle’. However, based on the Tibeto-Burman cognates, we can reconstruct a Proto-Sino-Tibetan word **tsrop* with the meaning ‘spade’. This suggest that rather than the Old Chinese one, the Sino-Tibetan reconstruction served as a model for Proto-Transeurasian **sarpa* ‘spade’. As such, we may be dealing with an ancient borrowing, going back to 8000 BP, when Proto-Sino-Tibetan was spoken in the Yellow River area (Zhang et al. 2020). Neolithic Yellow River and West Liao River cultures shared certain aspects in common, including tools associated with the management of cereals such as hoes, spades and shovels (Liu & Chen 2012: 127-152, Shelach-Lavi 2015: 68-95, Stevens et al. 2020; SI 7).

On the basis of OJ *sapi_1_* ‘spade, hoe’, we can reconstruct pJ **sapi* ‘spade’. However, in addition to missing a voiced labial stop (*b* < **np*), which would be the required correspondence for the labial cluster, the word is not attested in the Ryukyuan languages. This indicates that the word is a borrowing, probably from the Paekche Old Korean word **sap* ‘spade’, which has developed from Proto-Korean **salp* through liquid loss. The context of this borrowing is probably the migration of Paekche elite to Japan in the period of the Silla unification (660-668). More recently, the Middle Korean word ·*salp* ‘spade’ was borrowed into Middle Japanese as EMJ *sarafi* ‘rake’, deriving as the verb MJ *sarafe*- ‘dig out’.

**(10) Proto-Altaic **tari*- ‘to cultivate’**

Proto-Tungusic **tari*- ‘to cultivate; to sow, plant’

Evk. *tari*- ~ *tare*- ~ *tale*- ‘to sow’; Sol. *tari*- ‘to sow seeds; to plant; to cultivate; to grow; to disseminate; to inject; to infect, to catch a disease’, *tariŋko* ‘injector’; Ma. *tari*- ‘to cultivate, farm; to plow’; Jur. *tali- ~ tari-* ‘to sow, plant, cultivate’; Ulcha *tari̇-* ‘to sow, plant’, Na. *tari*- ‘to sow seeds’, *tariko* ‘sowing machine’, *tarici-* ‘to sow (seeds) regularly’; Ud. *tali-* ‘to plant a garden’, *tali* ‘garden for cultivating plants’, Oroch *tariko*- ‘to plant’

Proto-Mongolic **tari*- ‘to prepare the soil for cultivation; to sow, plant’

MMo. *tari*- ‘to sow, plant’; WMo. *tari*- ‘to sow, plant; to plough; to inoculate against infection, vaccinate’; Khal. *tari*- ‘to sow; to plant; to infect; to perform a deed; to inject; to vaccinate’; Ordos *tari*- ‘to sow; to plant; to plow; to inoculate (smallpox)’; Bur. *tari*-; Kalm. *tär*-; Eastern Yughur *tarə*- ‘to sow, plant’; Mgr. *tarə*- ~ *tari*- ‘to sow; to cultivate land’; Bao. *tarə*- ~ *tar*-; Kgj. *tari*-; Dgx. *tari*- ‘to sow, plant’

Proto-Turkic **tarï*- ‘to cultivate (land); to prepare the soil for cultivation; to sow, disperse’

OT *tarï-* ‘to disperse, cultivate (a field)’, MTk. (Chagatay) *tar*-, *tarï*- ‘to scatter seeds, sow’, Kirg. *tarï*- ‘to sow, cultivate ground, plow’, Bash. *tarï-* ‘to sow’, Alt. *tarï*- ‘to sow, cultivate’, Uig. *dar*-, *tar*- ‘to sow’, *tarï*-, *taru*- ‘to cultivate ground’, S-Yug. *tarï*- ‘to sow’; Khak. *tarï*- ‘to sow’, Tuv. *tarï*- ‘to plow, cultivate ground, sow, plant’; Tofa. *tarï*- ‘to sow, plant’

It should be noted that the Old Turkic form *tarï-* means ‘to disperse’ in addition to ‘to cultivate’. Since this meaning is primary in Turkic, while the Tungusic and Mongolic languages only reflect the meaning ‘to cultivate’, this could be taken as an indication of borrowing (Doerfer 1963: 244-245, 1968: 480-482, Doerfer 1985: 77, Rozycki 1994: 203). However, the underived verbal nature of the compared roots, the rich distribution of cognates across the Altaic languages and the presence of a potential cognate in Korean argue against a borrowing scenario.

There is a parallel in Proto-Korean **tɨl*- ‘to cultivate’ and its derived noun PK **tɨl-ɨk* ‘field-’ (PK *-*ɨk* deverbal noun suffix, Robbeets 2015: 469), which is not included in the etymology because of the mismatch of the Korean vowel. The Korean verb is reflected in various forms such as K *kulwu tuli*- ‘turn over the soil and sow for a second crop’ (*kulwu* ‘aftercrop’), K *tuli*- ‘to winnow grain from the chaff’, K *noph-tuli* ‘an unproductive rice field in a high place with little water’ (*noph*- ‘to be high, elevated’), K *kiph-tuli* ‘a low-set rice field’ (*kiph*- ‘to be deep’; -*i* deverbal noun suffix), while the derived noun is found in K *tulh*, MK *tulh* / *tuluh* 'field-, wild'.

**(11) Proto-Mongolo-Tungusic **pure* ‘seed, sprout, offspring’**

Proto-Tungusic **puri* 'sprout, offspring, child'

Even *hurken* ‘adolescent; young (person); single (person)', *hurke* ‘pregnant (of animals)’, *hurel* ~ *urul* ~ *urel* 'child, infant; grandchild; younger brother or sister', Evk. *huri-l* ‘children’, *huru:* ‘family; mother of many children’, *hurkə:n ~ urkə:n ~ hurkə:kə:n ~ urkə:kə:n* ‘boy’, Solon *ukkəxən ~* *urkəxən* 'son, boy', *uril* 'children; offspring', Neg. *xujil* (plural of *xutə*) ‘child (son, daughter); baby of an animal’, Olcha *purul(i)* 'children (plural)', *puri-* ‘to give birth’, Orok *puril* 'children (plural)', *puriɣə* ~ *pure: ~ puriə ~ purə ~ purəɣə* 'young', Na. *puril* 'children (plural)', *puri*- ‘to give birth (of animals)’, Oroch *xi:(g) ~ xiji(g)* 'children (plural)’, Ma. *fursun* ‘shoots, sprouts (especially of a grain); sawdust’

The final liquid in the Tungusic forms is a petrified plural suffix pTg *-*l*, e.g., Evk. *ŋa:le* 'hand' → *ŋa:le-l* 'hands', Neg. *oyo* 'reindeer' → *oyo-l* 'reindeers', Even *adal* 'net' → *adal-al* 'nets' (Benzing 1955: 1023-1026). The Tungusic forms in *-*kan* reflect a lexicalized diminutive suffix pTg *-*kA:n*, e.g. Even *ŋa:l-ka:n* 'small hand', Orok *ke:či-ke* 'puppy', Na. *toke-kan* 'small sleigh', etc. The Manchu form *fursun* 'shoots, sprouts' may be a borrowing from Mongolic because the meaning ‘sprout’ is not attested elsewhere in Tungusic and because -*-*sUn* is a collective and body part suffix in Mongolic but not in Manchu (Rozycki 1994: 83).

Proto-Khitan-Mongolic **püre* ‘seed, fruit; offspring, child’

MMo. *xüre* (HY), *hüren* (Muq) '1 offspring, descendant, seed, fruit, result', WMo. *üre* '1', Dag. *xur*, *hure* '1', Khal. *ür* '1', Bur. *üre* '1', Ordos *ür*, *üre* '1', Kalm. *ürn* 'child, offspring, seed, fruit, result', Eastern Yugur *hure* '1', Mgr. *fure*:, *fure*, *furie*:, *xuru*, *xure*:, *xurie*, Bao. *fure*, *fǝrɛ*, Dong. *Fure;* Khitan **p.úr.s* ‘descendant’

**(12) Proto-Japano-Koreanic **non* ‘field for agriculture’’**

Proto-Japonic **no* ‘field’

J *no*, OJ *no_1_* ‘field’, Yonamine (Okinawa) *moo* ‘hill, overgrown field’, Shuri (Okinawa) *mo:* ‘overgrown field’, Old Shuri *mo:* ‘wild field’, Hirara (Miyako) *nu:* ‘field’, Nagahama (Miyako) *nu:,* Ikema (Miyako) *nu:,* Ōgami (Miyako) *nu:*, Tarama (Miyako) *nu:*, Ishigaki (Yaeyama) *nu:* ‘field’, Hatoma (Yaeyama) *nu:*, Hateruma (Yaeyama) *nu:,* Kohama (Yaeyama) *nu:,*Taketomi (Yaeyama) *nu:,* Yonaguni *nu:* ‘field’, PR **no:* ‘field’

Proto-Koreanic **non* *‘*irrigated field, rice paddy field’

K *non* ‘rice field, paddy field, rice paddy; a group (network) of rice paddy plots, paddy’, K *non kali* ‘plowing a rice field’, MK ·*nwon* ‘paddy field, irrigated piece of land where plants (mostly rice) are planted and cultivated’, JJ *non* , KB *non*, KN *non*, JN *non*, JB *mwunon* ~ *nonkwuteyngi* ~ *nonttayki*, KW *non*, CN *non*, CB *non*, KG *non* ‘paddy’

Borrowing

**Proto- Sinitic **noŋ* ‘agriculture’ >> Proto-Japano-Koreanic **non* ‘field for agriculture’**

Proto- Sinitic **noŋ* ‘agriculture’

Old Chinese **nˤ[o]ŋ* 農 ‘agriculture, peasant, farmer, to farm’ (Baxter & Sagart 2014)

Borrowed in Tibeto-Burman?

Achang *nuŋ³¹ ȵɛ³¹ /* *nɔŋ³¹ ȵɛ³¹*’agriculture’, Nusu *nõ³¹ nie³¹* ‘agriculture’, Hani (Gelanghe) *nuŋ³¹ jɛ³¹* ‘agriculture’, Jinuo *noŋ⁴² ȵə⁴²* ‘agriculture’, Lisu (Northern) *nɔ²¹ je²¹* ‘agriculture’, Gurung (Chachok) *nõ* ‘farm labourer’, Dulong (Nujiang) *luŋ³¹ ȵe³¹* ‘agriculture’, Jingpo *nuŋ³¹ je³³* ‘agriculture’, Tujia (Northern) *lũ²¹ ne²¹* ‘agriculture’, Tujia (Southern) *nõ¹³ ne³³ ‘*agriculture’, Bai (Dali) *nu iɛ* ‘agriculture’

The ancestral Sinitic form from which Old Chinese **nˤ[o]ŋ* ‘agriculture’ descends is a likely model word for Proto-Japano-Koreanic **non* ‘field for agriculture’. Although a Proto-Sino-Tibetan reconstruction **noŋ* ‘agriculture’ cannot be entirely excluded, the Tibeto-Burman evidence is not very solid because the bisyllabic forms can be explained by borrowing from Chinese later in history. If we are indeed dealing with an ancient borrowing, the Japano-Koreanic word was probably borrowed after the break-up up between the Sinitic and Tibeto-Burman branches around 8000 BP (Zhang et al. 2020) and before the break-up between the Japonic and Koreanic languages, according to our Bayesian estimations in 5458 BP (SI 4; SI 24).

**(13) Proto-Japano-Koreanic **mati* ‘delimited plot for cultivation’**

Proto-Japonic **mati* ‘delimited plot for cultivation’

J *mati* ‘field (sector/measure), quarters, town, market’, OJ *mati* ‘garden, plot of land for agricultural proposes’

Proto-Koreanic **mat(i)-k* ‘delimited plot for cultivation’

K *math* ‘yard, court; place, ground; threshing ground, yard used for threshing’, MK *math* ‘yard, flattened land in front of or behind a house, plot of land for agriculture’, KB *mata*, *mataey* ~ *matayng* ~ *matap* ~ *matayi* ~ *matayng*, KN *matai ~ matang*, KW *matayi* ~ *mateyi*, *mateyng* ~ *polimateyi*, CN *matayi ~ matang*, JN *matayng*, *hantimangtang*, JB *mateyi ~ matang*, JJ *matang* , JN *matayng*, *hanti mangtang,* CB *matayi*, KG *matang* ‘yard; threshing ground’

*2.2. Reconstructions for food production and preservation*

To produce and preserve food, the ancestral speakers of Proto-Transeurasian fermented cereals, vegetables and fruits (etymologies (1) to (4)), grinded (7) and crushed food to pulp (6), extracted liquid from fruit and vegetables (5), soaked food such as nuts to remove unwanted components or make it more digestible (etymologies (8) to (11)), and brewed alcohol (12).

Whereas roasting technologies and lactid-acid fermented food using animal milk such as yoghurt, butter and cheese originated in western Asia, Eastern Asia is the birthplace of boiling and steaming technologies and lactid-acid fermented food using cereals and vegetables, such as pickles, *kimchi*, *miso*, *sake*, etc. (Fuller & Rowlands 2011, Susano 2016; SI 7). Our etymologies (1) to (4) reflect the extensive use of fermentation techniques among the speakers of Proto-Transeurasian. They were probably applied to improve the nutritional value of the food, dissolve unwanted components, lessen the labor of food preparation and facilitate portability.

Whereas the original meaning of the verbs in (1) to (4) can be reconstructed as ‘to ferment’, the association with dairying only developed individually in the Mongolic and Turkic branches, after the separation between Mongolic and Tungusic around 4500 BP. Therefore, the etymologies suggest that the lactic-acid fermentation techniques that were first applied on vegetal and cereal sources in the Neolithic were extended to animal milk by speakers of Mongolic and Turkic in the Bronze Age. This argues for the recycling of basic vegetal lactic-acid fermentation techniques as dairying technology. It can be noted that contemporary Mongolic and Turkic populations such as Mongols, Kazakhs who subsist on dairying, surprisingly tend to have low lactase persistence (Jeong et al. 2020). In order to remove the lactose, they need to ferment their milk into cheese or fermented drinks before consuming it. The low lactase persistence suggests that they developed dairying relatively recently in their population histories and explains the close semantic connection between vegetal fermentation terminology and dairying vocabulary in the daughter languages that moved towards the eastern steppe.

Although grinding in general is more typical of western “roasting” cultures than of eastern “boiling” cultures, our etymologies (6) and (7) suggest that food was crushed to pulp and grinded. This is in line with Liu’s (2016: 247) findings about the continued importance of ‘grinding’ in the Neolithic of the West Liao River region as opposed to the Yellow River region. Whereas grinding gradually disappear from the archaeological record in the Yellow River region after 7000 BP, when millet-based agriculture was intensified, the Neolithic populations in the West Liao River area, relied intensively on grinding for their food-production. The starches involved in this process were not limited to millets, but were provided by various nuts such as walnut, chestnut, acorn and pine as well as roots. Combining our reconstructed vocabulary for ‘grinding’ with the etymologies for durable wild food resources in Section 2.3*,* therefore indicates a broad-spectrum subsistence strategy with some economic dependence on the cultivation of plants such as millets.

The high frequency of verbs for ‘soaking’ in our dataset ((8) to (12)) also catches the eye. They could be connected to food production because in the Neolithic, nuts were soaked to remove the tannic acid (Kawashima 2016). The polysemy of ‘soak’, ‘brew’ and ‘chew’ in etymology (12) may be associated with the addition of human saliva to add sugars in the fermentation process when brewing alcohol. However, as a number of soaking verbs developed secondary meanings such as ‘to stain’, ‘to paint’ and ‘to bleach’ in (8) and (11) or derived nouns meaning ‘clothes’ in (11), some may be connected to textile production because bark fibers such as tree bark, ramie and hemp need to be soaked before they can be turned into thread and textiles need to be soaked for dyeing.

**(1) Proto-Transeurasian *saga- 'to ferment'**

Proto-Japonic **saka*- 'to ferment, be in heat, bloom'

J *sake* ‘saké, rice wine, alcoholic liquor’, OJ *saka*-/ *sake_2_* 'wine’, OJ *sakar*- ‘be at a peak, be in heat', OJ *sak*- 'bloom'; Yamatohama (Amami) *sehe*, Asama (Amami) *sjakɨɨ*,Yoron (Amami) *sai*, Yonamine (Okinawa) *sakii*, Shuri (Okinawa) *saki*, Old Shuri *saki*, Hirara (Miyako) *saki*, Nagahama (Miyako) *saki*, Ikema (Miyako) *saki*, Ōgami (Miyako) *saki*, Tarama (Miyako) *ʃaki* ‘rice wine’, *sakaN* ‘be at peak’, Ishigaki (Yaeyama) *saki* ‘rice wine’, *sakarɨ* ‘be at peak’, Hatoma (Yaeyama) *saki*, Hateruma (Yaeyama) *saki,* Kohama (Yaeyama) *saki,* Taketomi (Yaeyama) *saki,* Yonaguni *sagi* ‘rice wine’, PR **sake* ‘rice wine’, **saka*- ‘be at peak’

Proto-Koreanic **sak*- 'to ferment, to rot'

K *sak*-, MK *sak*- 'to ferment, to rot’; K *sek*- 'to rot', *sekim* 'ferment', JJ *kalwu* *sak*-, *kalu* *sak-*, *sayki*- 'to ferment, to rot’, KB *ssak*-, *saykhi*- 'to ferment, to rot’, KN *sakha*- 'to ferment, to rot’, JB *sak*- ‘be fully fermented, get old, to die down (of anger), relent, fade’, JN *ssek*- 'to rot away, decay, be corrupt, stink’, CB *ssuk*- 'to ferment, to rot’, CN *ssek*- 'to rot away, decay, be corrupt, stink’, KG *sak*- ‘be fully fermented, get old, to die down (of anger), relent, fade’

Proto-Mongolic **saga-* ‘to ferment; to reduce (food); to milk’

MMo. *sa’a*-, *sa*- 'to milk', WMo. *saɣa*- 'to shorten, contract, abridge; to diminish, to lessen; to do something in shorter time; to milk (tr.)', WMo. *saɣa*- 'to ferment', WMo. *saɣali* 'milch animal (cow, camel, etc.); milking; milk products; dairy (n.)', WMo. *saɣamal* 'milch, milk-giving (adj.)', WMo. *saɣalta* 'milch animal (cow, camel, etc.); milking; milk products; dairy', Khal *sa:-* 'to milk; to lessen, diminish, shorten (by tying in a knot) (tr.)', Khal. *saga*- 'to ferment', *saal^y^* 'milk, yield of milk, dairy; trap, crossbow', *sa:mal* 'just being milked', *sa:lt* 'milking; shortening, reduction', *sa:m* 'fresh milk, milk', Bur. *ha:*- 'to milk; to shorten, diminish (tr.)', Bur. *haxaj*- 'to become covered with mud',, *ha:li* 'milking capacity; dairy production; milch-cow; trap, crossbow', *ha:m* 'milking capacity', *ha:lta* 'hindrance, obstacle, brake', Kalm. *sa:*- 'to pull closer, to pull towards oneself; to reduce; to milk', Kalm. *saxǝ*- 'to ferment', *sa:li* 'arch, trap; milking, milk animal', *sa:lɣan* 'milking; pulling towards oneself; straining a bow', *sa:m* 'yield of milk; period of time', Ordos *sa:*- 'to milk; to draw back; to reduce; to delay; to diminish in number'; Mog. *sɔ*- 'to milk', Dag. *sa:*- 'to milk; to shorten, lessen (tr.); to fish with a drag seine', Dgx. *sa*- 'to milk'; Bao. *sa:*- 'to milk'; S.-Yugh. *sa:*- 'to milk'; Mgr. *s(u)a:*- 'to milk', *sa:li* ‘milch animal, female (goat, sheep)’

Proto-Turkic **sag-* ‘to milk; to extract’

OTk *saɣ*- 'to milk (an animal)', OTk (Karakh.) *saɣ*-, Chu. *sъ^w^v*-, Tk. *saɣ*- 'to milk; to fleece, despoil; to extract honey from the hive; to pour out rain (cloud); to unwind'; *saɣïm* 'milking; quantity of milk taken at one time, quantity of honey taken at one time; milk-giving animal'; *saɣlï* 'kept for milking'; *saɣlïk* 'dairy animal', *sagmal* 'milch, kept for milking, milk-giving; fit to be fleeced (person)', Gag. *sa:*-, Az. *saɣ*-, Tkm. *saG-*, Sal. *sax-*, Khalaj *sa:ɣ*-, Uzb. *sɔɣ*-, Uig. *saɣ*-, Krm. *sav*-; Tat. *saw-*; Kirgh. *sa:*-; Kaz. *saw-*; KKalp. *saw-*; Kum. *sav*-; Nog. *saw-*; SUig. *saɣ*-; Khak. *saɣ*-; Oirat *sa:*-; Tuv. *saɣ*-, Yak. *їa*-

**(2) Proto-Transeurasian **sugu*- ‘to ferment’** (in vowel alternation to (1))

Proto-Koreanic **su(k)u*- 'to make alcohol'

MK *swuwul*, MK *swuul* 'rice wine, alcoholic liquor’, K *sul* ‘rice wine; (intoxicating) liquor, drink, booze’, JJ *swul*, KN *swul*, JN *swul*, JB *swul*, KW *swul*, CN *swul*, CB *swul* ~ *cwu*, KG *swusil* ‘rice wine’

Proto-Mongolic **su(g)a*- 'to milk'

Monguor *sua*:-, Mogol *sɔ*-, Mogol *suɒ:-* ‘to milk’ (Nugteren 2011: 479” “peculiar Monguor (MgrH) and Mogol forms”)

Proto-Turkic **sug*- ‘to procure cheese’

OT *sug*- ‘to procure cheese’, *sugut* ‘dried cheese’

**(3) Proto-Transeurasian **sü:-* ‘to reduce/preserve food by fermentation’**

Proto-Japonic **su*- 'to be sour, fermented in vinegar'

J *su*, OJ *su* 'vinegar', J *su-*, OJ *su*- ‘to be sour, vinegared, acid, tart’, J *susi* ‘sushi, vinegared fish and rice’ (-*si* deverbal noun suffix, e.g., in OJ *ki_1_tasi* ‘rock salt, dried and hardened salt’, *karasi* ‘mustard’), Kagoshima/Koshiki/ Miyazaki *su* */* Kumamoto *suː,* Ōita*/*Fukuoka*/*Saga */*Fukue*/*Nagasaki *su* ‘vinegar’, Yamatohama (Amami) *sïïsari*, Asama (Amami) *sjɨɨhai*,Yoron (Amami) *siisjaN*, Yonamine (Okinawa) *siiseN*, Shuri (Okinawa) *siisaN*, Old Shuri *şiisaN*, Hirara (Miyako) *su:munu*, *suv*, Nagahama (Miyako) *si:si*, Tarama (Miyako) *sïfusal*, Ishigaki (Yaeyama) *sïsanu*, Hateruma (Yaeyama) *ʃiʃahaN,* Kohama (Yaeyama) *so:hanu,* Taketomi (Yaeyama) *ʃi:sa,* Yonaguni *c'jaN*, PR **su*- ‘to be acidic, sour’

Proto-Koreanic **swu*- ‘to be sour’ > **swu*-*y*- ‘become sour’ (*-*i*- fientive)

MK *swuy*- ‘become sour’, JJ *swiwu*- ‘become sour’, KN *si*- ~ *ssi*- ‘be sour’, JB *swey*- ‘be sour’, CB *swiwu*- ‘become sour’, CN *si*- ‘be sour’, KG *ssi*- ‘be sour’, KW *si*- ‘be sour’

Proto-Mongolic **sü*- ‘strain, filter, skim off, reduce (food)’ -> **sü-sün* (strain-COLL) ‘strained food’ > **üsün* > **sün* 'milk'

Proto-Mongolic **siü*- ‘to strain, filter, skim off, reduce (food)’ -> **siü-sün* (strain-COLL) ‘juice; reduced food’.

PMo **sü*- in MMo. *sü:-* ‘filter’

PMo **süsün* ~ **üsün* ~ **sün* 'strained food; milk' in WMo *sü(n),* *üsün*, MMo. *sün*, *su:n*, *su*, MMo. *süsün* ‘provision for travelling’, Khal. *sü(n),* Bur. *hün*, dial. *ühe(n),* Kalm. *sün*, *üsn*, Ordos *üsü(n),* Dag. *su:,* Eastern Yugur *sun*, *hsʉn* Mgr. *sun* , Mog. *sün*, *sun* 'milk' The form *süsün* is only preserved in MMo, *while *sün* is represented in MMo, Mog, Khal., Bur and Dag, whereas Kalm. *üsn*, Ord *üsü(n)*, and EYu *hsʉn* go back to **üsün*. The Mongolian word is borrowed as Manchu *sun* 'milk' (Rozycki 1994: 190). PMo **sün* 'milk' is unlikely to be borrowed from proto-Turkic **sü-(t)* given the missing imitation of the Turkic suffix *-*t*.

PMo **siü*- ‘to strain, filter, skim off, reduce (food)’ in MMo. *ši’u*-, *šü*:- ‘to filter’, MMo. *ši’ü-kü* ‘a filter’, WMo. *sigü*-, *šügü*- ‘filter, strain; fish out with a net’, Khal. *šüüx*, Ordos *šü:-,* Bur *šüüxe*, Kalm *šüüx*, Dag *su:-* ‘to scoop up’, Eastern Yughur *šü:-,* Monguor *śu:-*

PMo **siü-sün* ‘juice; portion of meat allocated to a certain person’ in MMo. *ši’usun* ‘ration’, *ši’usu* ‘food, provisions’, WMo. *sigüsü(n),* *šügüsü*, *šüsü* ‘sap, juice, food (usually meat) for offerings, food for traveling officials, whole sheep cooked and served to honored guests’, Khal. *šüüs(en)* ‘sap, juice; food (usually meat) for offerings’, Ordos *šʉ:s(ʉ),* Bur *šüühe(n)* ‘juice’, Kalm *šüüsn*, Dag *ču:s* ‘half-cooked meat juice, etc’, Mgr. *su:sən* ‘sheep cooked in its entirety’.

Proto-Turkic **sü:*- 'to filter, strain (milk or soup), reduce (food)' -> **sü:-t* (strain-NMLZ) ‘strained food’ > **sü:t* ‘milk’, *-*t* deverbal noun suffix, e.g. OT *sug*- 'to procure cheese' -> OT *sugut* 'dried cheese', OT *kurï*- ‘to dry up’-> OT *kurut* ‘dried curds’, OT *yogur*- ‘to thicken, condense’ -> OT *yogurt* ‘curdled milk’

PTk **sü:-* 'to filter, strain (milk or soup)' + *-(*X)z* causative suffix (Erdal 1991: 757) in OT *süz*- ‘to filter, to strain (the action of filtering milk and soup)’

PTk **sü:t* ‘milk’ in OT *süt* (OUygh.), Karakh. *süt*, MT *süt*, Tk. *süt*, Az. *süd*, Tkm. *sü:t*, Gag. *süt*, Tat. *sü̆t*, Kirg. *süt*, Kaz. *süt*, Nog. *süt*, Bash. *hü̆t*, Balk. *süt*, Krm. *süt*, Kkpak *süt*, Salar *süt*, Kumyk *süt*, Uz. *sut*, Uig. *süt*, Tuva *süt*, S-Yug. *süt*, *söt*, Khak. *süt*, Shor *süt*, Yak. *ü:t*, Halaj *si:t*, Chu. *sǝʷt* 'milk'

**(4) Proto-Transeurasian **bilče*- 'to ferment a liquid, mix with a liquid’**

Proto-Japonic *pisi* ‘fermented liquid’

J *hisiko*, OJ *pisiko_1_* ‘anchovy’ (OJ *ko_1_*‘little one’), J *hisio*, OJ *pisipo* ‘a liquid made from barley and soy beans, mixed, fermented, and salted, and used as a pickling vehicle for vegetables’, J *sisi-bisio* ‘salted meats’. There are no cognates in the Ryukyuan languages.

Proto-Korean **pici*- ‘ferment (liquid), brew, make dough’

K *pic*-, MK *pic*- ‘to shape dough for (rice cakes), roll into balls (as dumplings); brew (rice wine), ferment’, JJ *ppici*-, KB *pic*-, JB *pic*-, JN *pic-,* CB *picu*-, CN *picu*-, KW *pici*-

Proto-Mongolic **bilca*- 'mix a liquid with food’

WMo. *bilča*-, *bilče*- 'to smear all over, to splash in the mud (tr./intr.), WMo *bilčayi*- 'to be of thin consistency (of though, batter), become smooth or flattened, spread under pressure', *bilčal*- 'to flatten completely, squash into pulp, crush, pound (millet) (intr.), Khal. *b́alca*-, Bur. *bilsa*-, Kalm. *bilcǝ*-, Ordos  *bilčal*- ‘to become flat and watery, to smear all over’. The Mongolian verb is borrowed as Manchu *bilca*- ‘to mix (flour), to glue’.

Turkic **bilči*- 'to ripen, to churn (milk, butter), to stir up, beat a liquid in order to make butter’

OT *biš*- ‘1 to become boiled, ripe’, MT *biš*- ‘1’, Tk *piš*- ‘1, Turkmen *biš*- ‘1’, *pišek* 'churn pestle', *pišek-le*- 'to churn', Az. *biš*- ‘1’, Gagauz *piš*- ‘1’, Tatar *beš*- ‘1’, *peš*- 'to ripen, stir up, chirn', Kirghiz *bɨš*- / *biš*- 'to ripen, churn (koumiss)', *biškek* 'churn-staff for kumis', Kaz. *pis*- 'to ripen, to stir up, chirn', *pispek* 'churn-staff', Nog. *piskek* 'churn-staff', Bashkir *beš*- ‘1’, *beše*- 'to churn; to beat', *beškäk* 'churn-staff', Karaim *biš*-, *piš*- ‘1’, Kkpak. *pis*- 'to ripen, stir up, chirn', *piskek* 'a big churn', Kumyk *biš*- ‘1’, Uighur *piš*- ‘1’, Uzbek *piš*- ‘1’, Uz. *piškak* 'churn pestle', Khakas *pɨs*- ‘1’, Sary-Yughur *pɨs*- ‘1’, Shor *pɨš*- ‘1’, Tofalar *bɨš*- ‘1’, Yakut *bus-* ‘1’, *bis*- 'to smear', Dolg. *bis*- 'to smear', Halaj *bɨš*- ‘1’, Chuvash *piś*- ‘1’, Chu. *pǝźer*- ‘to pound, beat’

**(5) Proto-Transeurasian **silɔ* ‘broth, soup, juice; liquid food extracted from vegetables, fruit or meat’**

Proto-Japonic **siru* ‘broth, soup, juice’

J *siru*, OJ *siru* ‘juice, sap; broth, soup’, Yamatohama (Amami) *siru*, Asama (Amami) *sɨruu*,Yoron (Amami) *sjuu* / *siru*, Yonamine (Okinawa) *siruu*, Shuri (Okinawa) *siru*, Old Shuri *siru*, Hirara (Miyako) *sunusï* / *su:,* Nagahama (Miyako) *sunusï* / *su:,* Ōgami (Miyako) *su:nusï*, Tarama (Miyako) *sïru*, Ishigaki (Yaeyama) *suru*, Hatoma (Yaeyama) *su:*, Hateruma (Yaeyama) *su:,* Kohama (Yaeyama) *su:,* Taketomi (Yaeyama) *ʃu:,* Yonaguni *c'iru*, PR **siru* ‘broth, soup’

Proto-Mongolic **silö* ‘meat broth, soup, juice’

MMo. *šulen*, *šülen*, *šīlɛ*, *šilän*, *sülen* ‘meat broth, soup’, WMo. *silü(n),* *šölü*, *šülü* ‘soup, bouillon, broth’, Khalkha *šöl*, Buriat *šülen*, Kalmuck *šöln*, *šüln*, Ordos *šölö*, Dongxian *šulie* 'soup; juice', Baoan  *šile* 'soup; juice', Dagur *šil*, *šile* 'soup; juice', Eastern Yoghur *šǝlen*

Monguor *šulō*

**(6) Proto-Transeurasian **niku*- 'to crush to pulp’**

Proto-Koreanic **niki*- 'to crush to a pulp, knead (flour)'

K *iki*-, MK *niki*- 'to crush to a pulp, mash, knead, beat water into flour’, KB *ikay*-, JB *cinniki*-, JN *ningkki*-~ *ingkkallu*- ~ *ingkkey*- ~ *ingkkuli*- ~ *ingkhay*-, CB *ungkki*-, CN *ukkay*-, KG *ukkay*-, KW *minggi*- ~ *minggay*-

Proto-Mongolic **niku*- 'to crush, knead (flour), '

WMo. *niqu*- ~ *nuqu*- '1 to knead (flour), mash, crumple, rub, press, massage', *niquɣur* 'implement for kneading dough', MMo. *nuqu*- '1', Khal. *nuxa*- '1', Bur. *ńuxa*- '1', Kalm. *nuxǝ*- '1', Ordos *nuxu*- '1', Bao. *noġǝ*- '1', Dag. *nogu*- '1', Mgr. *nuġu*- '1', Mog. *nuqu*- ~ *noqu*- 'to crush', Dong. *nuqu*- 'to hit with force'

Proto-Turkic **yïk*- 'to crush’

OTk. *yïk*- '1 to crush, demolish, destroy', OT (Karakh.) *yɨq*- '1', Tk. *yɨk*- '1', Az. *yɨx*- '1', Tkm. *yɨq*- '1', Gag. *yɨq*- '1', Tat. *yɨq*- '1', Kirg. *ǯɨq*- '1', Karaim *yɨq*- ~ *yɨx*- '1', Kaz. *žɨq*- '1', Nog. *yɨq*- '1', Bash. *yɨq*- '1', Kpak. *žɨq*- '1', Kum. *jɨq*- ~ *jix*- '1', Uz. *yiq*- '1', Uig. *yiq*- '1', Khak. *yuq*- '1' , Oirat *yɨq*-, *d́ɨq*- '1', Khalaj *yuq*- '1', Chu. (dial.) *śăx*- '1'.

**(7) Proto-Transeurasian **suru*- 'to grind, rub'**

Proto-Japonic **sura*- 'to grind, rub': J *sur*- (B), *sure*- (B) ‘to rub against eachother’, OJ *sur*- 'to grind, rub', J *surari* ‘without trouble, smoothly’ (-*ri* adverbializer), *sura-sura* ‘without a hitch, smoothly’, Shuri *sir*- 'rub, grind', *șiyuŋ* 'to rub', Shodon *k'usryum*, Hirara *sïpag^z^ï*, Ishigaki *sïsuŋ*, Kabira *suri*, Yonaguni *ccituŋ*, *ciruŋ,* pR **suri*- ~ **kosuri*- 'to rub'

pTg **suru*- ‘to grind

Ma. *šuru*- ‘to grind, whet, sharpen’

In a few cases Manchu displays a palatal sibilant *š-* rather than *s-* in correspondence with words with initial *h*- in Even and initial *s*- in the other Tungusic languages. There is no internal ground for this palatalization, such as a following high vowel. However, as it concerns only a few cases and since the palatalization is restricted to Manchu, Benzing (1955: 989-990) refrains from establishing a separate palatal sibilant **š*- in proto-Tungusic. I do not consider Even *huruŋ*- 'to grind, pound, mash, pestle, crumble, divide in small parts', *huruwe:* 'bits and pieces, crumbs' to be related here. These forms derive from pTg **puru*- 'to crush', reflected in Evk. *huru*-, *hurgu-*, *horo*- 'crush', Ma. *furu*- 'to chop, cleave', Olcha *pori*- 'to crush', Na. *purtu* 'crumbs'.

pTk **sür(ü)*- 'to rub, smear' (pTk *-*ti*- causative-passive; e.g. OT *arï*- ‘to be(come) clean (intr.)’ → *arït*- ‘to clean (tr.)’; Robbeets 2015: 290-292): OT *sürt*- '1 to rub, smear (tr.)', MT *sür*-, *sürüt*-, *sürt*- '1', Tk. *sür*-, *sürt*-, Az. *sürt*-, Tkm. *sür*-, *sürt*-, Gag. *sürüt*-, Uz. *surt*-, Tuva *sür*-, Yak. *ür*-, Khak. *sürt*-, Kirg. *sür*-, *sürt*-, Kaz. *sürt*-, Nog. *sür*-, *sürt*-, Bash. *hü̆r*-, *hü̆rt*-, Balk. *sürt*-, Karaim *sürt*-, Kpak *sür*-, *sürt*-, Kum. *sürt*-, Chu. *sĕr*-

**(8) Proto-Transeurasian **simi*- ‘to soak (food)’**

Proto-Japonic **sima*- ‘to soak, permeate’

J *sime*- ‘to be damp, soaked’, J *simi*- 'to soak', *simi* 'stain', OJ *sim*- ‘to pierce, soak, sink, permeate’, Shuri *suum*- ‘to soak’, Ishigaki (Yaeyama) *sïmiruŋ*- ‘to irrigate (tr.)’,

Although the Korean verb K *sumi*-, MK *·su·mui*-‘to soak, permeate, infiltrate, sink into’ is a good semantic match and looks similar, the Korean vowel -*u*- < pK **ɨ* represents an irregular sound correspondence.

Proto-Mongolic **sime*- ‘to soak (food), moisten, suck up’

WMo. *sime*- ~ *simi*- ‘1 to draw a liquid into the mouth, suck up, sip (tr.)’, *simed*- ~ *simede*- ‘to become soaked with, become saturated, become absorbed (intr.); to penetrate’, *simedke*- ‘to soak in, absorb (tr.)’, *sime* ‘3 essence, extract, sap, nourishment’, *simejin ariki* ‘liquor made from fermented milk’, MMo. *šimi*- ‘to soak; to suck’, Khal. *šime*- ‘to suck’, *šimte*- ‘to become soaked’, Khal *šime*- ‘1’, *šimde*- ‘2’, *šim* ‘3’, Buriat *šeme*- ‘2’ *šeme* ‘3’, Kalm. *šimǝ*- ‘to suck, suck out’, *süm*- ‘to suck (of cattle)’, *šime:ldǝ*- ‘to become absorbed, to penetrate’ (< **sime-ge-ldü*-), *šim* ‘juice, taste, nutritious liquid’, *mali:n šimǝ* ‘edible products, the juices of cattle (i.e. butter, cheese, milk, fat)’, Ordos *šime*- ‘2’, *šime* ‘3’, Dagur *šime*- ‘2’, *šim* ‘3’, Eastern Yugur *šǝme*- ‘2’, *šǝme* ‘3’, Mgr. *šǝme*-, *šǝmu*- ‘2’, *śime*, Mogh. *šimi* - 'to suck', Dgx. *ʂïməi*- ‘to sip, take in the mouth and taste’

A plausible cognate in Tungusic is Manchu *sime*- ‘to soak, to moisten, to seep into; to favor’, *simi*- ‘to suck’ and *simen* ‘moisture, juice, secretion, nutritive fluid’. However, the poor distribution of this verb in Tungusic, restricted to Manchu, signals that this word may be borrowed from Mongolic. If this is indeed the case, the borrowing yields additional support for reconstructing the Proto-Mongolic meaning as ‘to soak, to moisten’, preserved in the passive derivative pMo **sime-de*- ‘to become soaked’ and the derived noun **sime-n* ‘moisture, liquid’

In Turkic, we can reconstruct pTk **simür*- ‘to suck’ on the basis of Karakhanid *simür*- ‘1 to suck, swallow’, Turkish *süm*-, *sümür*- ‘1’, Az. *sümür*- ‘1’, Tkm. *sümür*- ‘1’, Kaz. *simir*- ‘1’, Nog. *simir*- ‘1’, Bash. *hĭmĭr*- ‘1’, Kkp. *simir*- ‘1’, Uz. *simir*- ‘1’, Uig. *sümür*- ‘1’ and Chu. *sim*, *sǝm* 'honey drink'. However, the limited distribution of the Turkic verb, the unmatched stem-final -*r* in Turkic and the fact that Turkic only preserves the secondary meaning ‘to suck’ makes us suspect that this form is borrowed.

**(9) Proto-Transeurasian **ulu*- ‘to soak, wet’**

Proto-Japonic **uru*- ‘to irrigate, make wet’ + *-*pa*- ~ -*pə*- reflexive-anticausative (Robbeets 2015: 292-294)

OJ *urup*- (?B) ‘to get muddy, be wet’, J *uruow*-, OJ *urupop*- (?B) ‘to get damp, get moist, receive profits, get enriched’, OJ *urum*- (?B) ‘to get wet, moist’, Old Shuri *ʔuri:-,* Hirara (Miyako) *uri*-, Nagahama (Miyako) *uri:*-, Yonaguni *uri*- ‘to irrigate (tr.)’, PR **ur*- ‘to irrigate (tr.), make wet’

Proto-Koreanic **uli*- ‘to soak’

K *wuli*-, MK *wuli*- ‘to steep, soak, bleach’, JJ *wuli*-, *wulenay*-, KB *wuli*-, KN *wula*- ~ *wulwu*-, JB *wulku*-, JN *wulkwu*-, *wulki*-, CB *wulkwu*-, CN *wulkwu*-, *wulkhwu*-, KG *wulkwu*-, *wulkwu*-, *wulkhwu*-, *wulkhi*-

Proto-Tungusic **ula*- ‘to wet, soak’ + *-*p*- anticausative (Robbeets 2015: 296-298)

Evk. *ula*- ‘to soak, to wet (legs); to become wet; to melt (about falling water snow)’, *ulap*- ‘to become wet’ (Evk. -*p*- anticausative; Robbeets 2015: 298); Even *ụl*- ‘to soak, to wet’, *ụlab*- ~ *ụlap*- ‘to get wet’, Sol. *ụlakku:* ‘wet’, Ma. *ulga*- ~ *ulha*- ‘to wet, dampen, dip in a liquid’, *ulgaku:* ‘inkwell, well for ink on an inkstone’, Jur. *ul(h)a*- ‘to wet, to dampen, to dip in liquid’, Orok *ụla*- ‘to soak; to wet’, Na. *ụlarïko:* (dial.) 'wet', Ud. *ula*- ‘to soak, to wet; to set a net’, *ula-sa*- ‘to become wet (about dried fish); to be settled (about net)’

**(10) Proto-Transeurasian **nɔr*- ‘to soak’**

Proto-Japonic **nura*- ‘to be(come) wet’

J *nure*-, OJ *nure*- (A) ‘to be(come) wet, damp; to come undone, loose’, Yoron (Amami) *nidituɴ* 'to be wet', Shuri (Okinawa) *ndir*- (A) 'to get wet', *ɴditooɴ* 'to be wet', Hatoma (Yaeyama) *zoori bee* 'to be wet'

Proto-Mongolic **nor*- 'to be(come) wet'

WMo. *nor*- 'to become wet, soaked, drenched, damp, moist (intr.)', *norɣa-* 'to wet, moisten, soak (tr.)', MMo. *nur*- 'to be wet, soak', *norma* 'moistened', Khal. *nor*- 'to get wet', Bur. *noro*- '1 to become wet, soaked', Kalm. *nor*- 'to become wet', *norɣa:-* 'to make wet', *norɣu:(n), noru:* 'wet', Ordos *nor*- '1', Dgx. *noro*- ~ *nuru*- ~ *noru*- '1', Dag. *noir*- '1', *noirga*:- 'to make wet', Mgr. *no:ri*- ~ *nori*- '1'

**(11) proto-Altaic **deb*- ‘to soak’**

Proto-Tungusic **debe*- 'to make wet, paste, paint'

Evenki *dewe*- 'to paint', Even *dewe*- ‘to paint', Negidal *dewekse* 'paint', Manchu *debe*- ‘to overflow, run over, to flood’ , Ulcha *dewekse* 'paint', Orok *dewe:*- 'to paint', Oroch *dewukse*, *dewekse* 'paint'

Proto-Mongolic **debe*- ‘to soak (e.g. clothes), make wet'

WMo. *debte*- ~ *debtü*- ‘1 to be soaked, steeped, saturated with a liquid; to swell, expand (intr.)’ (pMo *-*dA* passive), *debtege*- ‘to soak or steep in a liquid, dampen, moisten, soften’, e.g. *amu debtege*- ‘to soak grain’, WMo. *debül*- ‘to spout, gush, boil, bubble, to overflow’ (pMo. *-*l*- iterative), MMo. *debul*- 'to boil', WMo. *debege* ‘marshy place, damp or wet area, alpine grassland’ (pMo *-*GA* place suffix), Khalkha *devte*- ‘1’, Buriat *debte*- ‘1’, Kalmuck *deptǝ*- ‘1’, Ordos *debte*- ‘1’, Dagur *debte*-, *derte*- ‘1’, Eastern Yogur *debte:-* ‘1’, Monguor *tǝbdē*-, *tudē*- ‘1’, Mogol *debtäl*- `to make fall into the water'

Proto-Turkic **yẹbi*- 'to become wet, soak'

Karakhanid *yebe* 'dampness', Tatar  *ǯebe*- '1 to become wet, soak', Uzbek *ivi*- '1’, Uig. *ivi*- '1, Shor  *čibi*- '1, Oirat *d́ibi*- '1’, Chuvash  *śǝʷve* 'whey', Yakut *sibi:n*- 'fresh', Kirghiz *ǯibi*- '1’, Kazakh  *žibi*- '1’, Noghai  *yibi*- '1’, Bashkir *yebe*- '1’, Karaim *yibi*- '1’, *ibi*- '1’, Karakalpak  *žibi*- '1’, Kumyk  *yibi*- '1’

**(12) Proto-Japono-Koreanic **kama*- ‘to soak, brew’**

Proto-Japonic **kamǝ*- ‘to chew, brew, dye’

OJ *kam*- ‘to brew’, J/OJ *kamos*- (B) ‘brew’, OJ *kamu-pata* ‘multi-colored woven cloth’, J *kamu* (B)*,* OJ *kam-* ‘to bite, gnaw, chew, masticate, eat’; Yamatohama (Amami) *xamuri* 'to eat', Asama (Amami) *kamyu*ɴ 'to eat', Shuri (Okinawa) *kamu*ɴ 'to eat', Hirara *kam* 'to bite', Ishigaki *kamuŋ* 'to bite', Yonaguni *kamuŋ* 'to bite'*,* pR **kamu*- 'to bite, eat'

Proto-Koreanic **kʌmʌ-* 'to put in a bath'

MK ·*kom*-, K *ka:m*- 'to bathe, wash', JJ *mom kam*- (*mom* ‘body’), KB *mok kkam*-, KN *kkam*-, JB *kamu*-, CB *kkam*-, KW *kam*- ~ *kkam*-

*2.3. Reconstructions for durable wild food resources*

Our Transeurasian reconstructions reflect reliance on durable wild food resources such as nuts (etymologies (1), (2) and (4)) and wild roots (3). As these are collected in large quantities and stored for longer periods, they point to sedentism (Shelach et al. 2019).

During the Neolithic, the West Liao River region consisted for 55% of trees, a mix of conifer and broadleaf trees, the latter category being predominantly oak (*Quercus*) and walnut (*Juglans*) and also some chestnut. Wild walnuts (*Juglans mandshurica Maxima*) are found on the floors of houses at the Xinglongwa site (Shelach 2000: 380). Analyzing starch residue on grinding stones Liu (2016), found that Xinglongwa people in the West Liao River region processed acorns and several plant roots for starch at least as frequently as millets. It is probably significant that it is precisely nuts such as walnut, acorn, chestnut or pine nut, which were targeted for their starch and consumed by Xinglongwa people, that turn up in our etymologies. Walnuts and acorns were also stored at early agricultural sites of the Zaisanovka culture (5300-2500 BP) in the Russian Far East (Sergusheva 2009, Kuzmin 2013). Our archaeological dataset (SI 7) further shows that the consumption of acorns and walnuts remained common in farming societies in the Amur basin, the Primorye, Korea and Japan.

According to Liu (2016) roots and bulbs were also targeted for their starch in the Neolithic in the West Liao area. Our reconstruction (3) for Althaea rosa, a plant native to Northern China, may reflect the fact that the root of these plants was used medicinally.

**(1) proto-Transeurasian **kuru* ‘edible nut used for starch production such as walnut, acorn, chestnut or pine nut’**

Proto-Japonic **kuru* ‘walnut, chestnut’

J *kuri* (2.3), OJ *kuri* ‘chestnut’, J *kurusu* ‘chestnut grove’, OJ *kuri/u-kuma* ‘Chestnut Corner’, J *kurumi*, MJ *kurumi* ‘walnut (*Juglans regia*)’ (MJ *mi* ‘fruit, nut’). As chestnuts are not native to the islands, there are no cognates for this word in the Ryukyuan languages.

Proto-Koreanic **kul* ‘oak < ? walnut’

MK *kwul* ‘oak’ in K *kwul pa:m* ‘acorn’ (K *pa:m* ‘chestnut’), MK *kwul pam* ‘bristletooth oak (*Quercus serrata*)’, K *kwul cham-namu* ‘oriental oak (*Quercus variabilis*)’ (K *cham-namu* ‘oak tree’), K *kwul phi* ‘oak bark’ (K *phi* ‘bark’), *kwul phi namu* ‘Walnut-like tree (Platycaria strobilacea)’, KN *kal chamnamwu*, KG *kwul* *chamnangkwu*’oak’

Proto-Tungusic **kuri* ‘pine cone, pine nut’ (pTg *-*ktA* collective for small items)

Ma. *xuri* ‘cone of coniferous trees’, Jur. *xuri* ‘cone of coniferous trees’, Evk. *korekta* ‘cedar nut’ (Menges 1983: 274), Na. *koriči* ‘water chestnut’, *korekta* ‘pine cone, cedar cone’

**(2) proto-Transeurasian **xʊsi* ‘edible nut used for starch production such as walnut, acorn, chestnut or pine nut’**

Proto-Japonic **kusi* ‘chestnut’

OJ *kusi* ‘chestnut’. As chestnuts are not native to the islands, there are no cognates for this word in the Ryukyuan languages.

Proto-Tungusic **xusi* ‘acorn’ (pTg *-*ktA* collective for small items)

Ma. *usixa* ‘big nut’, Evk. *usikta* ‘oak tree’, Na. *xosaqta* ‘acorn’, Ud. *uhikta* ‘acorn’

Proto-Mongolic **kusi* ‘walnut’ (pMo *-*Ga(n)* diminutive, often in plant names, e.g. WMo. *čibaɣa(n)* ‘jujube’, *abuɣa* ‘marshmallow’ etc.)

WMo. *qusiga* ‘walnut, nut; testicles’, Khal. *xušga* ‘walnut’, Kalm. *xušg* ‘walnut’, Ordos *ġušiġa* ‘walnut’, WMo. *qusi* ~ *qosi* ‘cedar, Siberian pine’, Khal. *xuš* ‘cedar, Siberian pine’, Kalm. *xoš* ‘cedar, Siberian pine’.

**(3) proto-Transeurasian** **abu* ‘plant of the Althaea genus with roots rich of starch’

Proto-Japonic **apu* ‘hollyhock (*Althaea rosa*)’

J *aoi*, OJ *apupi_1_* ‘hollyhock (*Althaea rosa*)’; Ishigaki *Ɂaurɨ* ‘hollyhock (*Althaea rosa*)’

Proto-Koreanic **apok* ‘marshmallow (*Althaea officinalis*)’

K *awuk* ‘marshmallow, Althaea officinalis’, MK *a·wok, a·hwok* ‘(Vegetable) mallow. A biennial malvaceae plant. It is 50-70cm tall and has alternate leaves with five leaflets like maple leaves. Its soft stems and leaves can be boiled into soup. Its seeds are used as a diuretic in traditional Korean medicine’, KN *akwu* ~ *yawuk* ~ *awu* ~ *awup* ~ *awus*, KS *akwuk*, *apwuk*, *aok*, KB *apang*, *apok*, *apwuci*, *awung*, *awuk*, HG *apwuk*, JN *aong*, *awung*, *aok*, *wuk* KN *awu*, *awup*, *awus*, KG *aok* ~ *awuk*, JL *aok*, CC *aok*, HH *aok,* JJ *awuk*, JN *akwung*, JB *aok*, KW *awuk* ~ *aok*, CN *awuk*, CB *awuk* ‘marshmallow’

Proto-Mongolic **abu* ‘marshmallow (*Althaea officinalis*)’ (pMo *-*Ga(n)* diminutive, often in plant names, e.g. WMo. *čibaɣa(n)* ‘jujube’, *qusiga* ‘walnut, nut’, etc.), WMo. *abuɣa*, Khal. *avga* ‘marshmallow (*Althaea officinalis*)’

**(4) Proto-Japano-Koreanic **pami* ‘edible true nut, i.e. dry fruit with only one seed such as acorn, hazelnut and chestnut’**

Proto-Japonic **pami* ‘edible true nut such as hazelnut or acorn’

J *hami*, OJ *pami_1_* ‘Phlomis umbrosa, herb with three-sided nutlets used against inflammation’, J *hasibami*, pJ **pasibami_1_* ‘filbert, hazel’, OJ *turupami_1_* ‘acorn’, OJ *katabami_1_* ‘wood sorrel, oxalis’. There are no Ryukyuan cognates for this word. On the islands the plant is known as Hirara *natʃɨkɨ*, Nagahama *na:dʒɨtʃɨ*, Ishigaki *ma:sufusa* (lit. ‘salt grass’) and Yonaguni *nidʒɨtʃi*.

Proto-Korean **pami* ‘edible true nut such as chestnut or hazelnut

MK ¨*pam* ‘chestnut’, MK *kay(y)am,* *kayyem,* *kaywom,* *kayom* ‘hazelnut’ (< **kay pam*), K *pa:m*, KB *paam*, KN *paym ~ pam*, JJ *pam*, JN *pam*, JB *paym*, *pam*, KW *cham-pam*, CN *pam*, CB *pam*, KG *pam*, KS *ppam* ‘chestnut; nut’

The rising tone in Middle Korean suggests a disyllabic origin for this word, while the palatal glide in KN *paym and* JB *paym* indicates diphtongization from an original stem-final *-*i*.

*2.4 Reconstructions for domesticated animals*

Only two potentially domesticated animals can be unambiguously reconstructed: ‘dog’ (etymology (1)) and ‘pig’ (etymologies (2) and (3)), whereby the words for ‘pig’ do not go back any deeper in time than Proto-Altaic, estimated between 9000 and 7 000 BP (SI 4; SI 24). Our Altaic reconstructions for ‘pig’ are likely to refer to the domesticated variant because the preserved terms reflect distinct terms for ‘pig’ depending on age, sex, etc. Although some hunter societies such as the Mongolian Khamnigans may have a sophisticated system of classification of wild pigs, which are the object of hunting, the use of such detailed sex and age distinctions in Altaic, may point to pig raising and domestication. Our linguistic reconstructions are therefore in line with our archaeological findings (SI 7) that the earliest domesticated pigs in our data are from Xinglongwa in the West Liao basin (c. 8400-7200 BP).

Pig domestication is is more typical for agricultural than pastoral societies. Parallel to the semantic development observed in Section 2.2., whereby vegetal fermentation techniques are recycled as dairying processes in the Transeurasian daughter branches on the eastern steppe in the Bronze Age, we observe a semantic shift in etymology (2) in Turkic, repurposing the original meaning ‘pig’ as animal names related to Bronze Age pastoralism, such as 'cattle,’ ‘camel’ or ‘horse'.

**(1) Proto-Transeurasian **inu* ~ **ina* ‘dog’**

Proto-Japonic **inu* ~ **ina* 'dog'

J *inu* (2.3), OJ *inu* 'dog'; J *enu*, OJ *wenu* ‘puppy dog’ (< *wo ‘small’ + inu ‘dog’), Yamatohama (Amami) *ɁiN*, Asama (Amami) *ɁiN*,Yoron (Amami) *inu*, Yonamine *ɁiNnukɁwaa* (Okinawa), Shuri (Okinawa) *ɁiN*, Old Shuri *ɁiN*, Hirara (Miyako) *iŋ*, Nagahama (Miyako) *iŋ*, Ikema (Miyako) *iŋ*, Ōgami (Miyako) *iŋ*, Tarama (Miyako) *ina*, Ishigaki (Yaeyama) *iŋ*, Hatoma (Yaeyama) *iŋ*, Hateruma (Yaeyama) *inu,* Kohama (Yaeyama) *iŋ,* Taketomi (Yaeyama) *iŋ,* Yonaguni *iŋ,* PR **inu* ~ **ina* ‘dog’

Proto-Tungusic **ina* ~ **inu* ‘dog’

pTg **ina-kun* ~ **ina-ki:* 'dog', **in(a)-da* '(going) with a dog' (pTg *-*kun* suffix common in animal names, e.g. pTg **ju-kun* 'otter', pTg **gia:-kun* 'sparrow hawk'; Benzing 1955: 1015; pTg *-*ki:* animal suffix, e.g. pTg *sula-ki:* 'fox', **xölü-ki:* 'squirrel', etc., pTg *-*na*:- (~ -*da:-* after *n*): Even. *ina* ~ *nina* ~ *ŋina, inakin ~ ŋinakin* '1 dog', Evk. *ŋin* '1' (*ŋinal* PL), *ŋinakin ~ ginakin,* *ŋinda-* 'to go out with the dogs, hunt with the help of dogs', Solon *inaxĩ* ~ *ninaxĩ* ~ *ninakin* '1', Neg. *ina* ~ *nina*, *ninakin*, *enakin ~ enaxĩ* '1', Oroch *inaki* ~ *inaxki* '1', Ud. *in'an* '1', Olcha *iŋda* '1', Orok *nina* ~ *ŋina* '1', Olcha *inda* '1', Na. *inda*, *inakĩ* '1', Ma. *indaxun ~ indaju* '1', Sibe *jonǝhuŋ*, *inǝhuŋ* '1'

The phonological development assumed to have taken place in Tunguisic is **ina-kun* ~ **ina-ki:* (contamination*) > *ina-kin > *ginakin* (assimilation) *> *ŋinakin > *ninakin.* I assume that the Evenki denominal verb *ŋinda-* 'to go out with the dogs, hunt with the help of dogs' contains a dissimilated form of the suffix pTg *-*na*:- 'to go out (with)'. Since this suffix is also reflected in the collective suffix pTg *-*nan* 'together with' (Benzing 1955: 1021, e.g. *aki:-nan* 'together with the older brother'), I assume that the southern Tungusic forms *inda* reflect such a collective derivation.

pTg **inu:-ke* 'dog, wolf'

Evk. *ńēkē* ‘sable’, Even *ŋȫke* ‘male (of dog, wolf, fox)’, Sibe *juxǝ* ‘wolf’, Ma. *ńoxe* ‘wolf’, *nuxere* 'puppy', Oroch *ŋöksjö* 'wolf' (Cincius 1975: 587, 651, 665, 606)

**(2) Proto-Altaic **toru* 'young male pig'**

Proto-Tungusic **toro* 'male pig' + *-*ki:* animal suffix, e.g., pTg *sula-ki:* 'fox', **xölü-ki:* 'squirrel', **ina-ki:* 'dog', etc.

Evk. *toroki:* 'boar', Neg. *toroki:* 'boar, wild boar (upper dial.)'

Proto-Mongolic **toru* 'young male pig' + PMo *-*i* animal suffix, e.g., **gaka-i* ‘pig’, **noka-i*, **moga-i* ‘snake’

WMo. *torui* 'suckling pig', *toruɣu* 'one-year-old wild boar', Khal. *toroy* 'piglet; young of a domestic yak', *toru:* 'two-years-old piglet', Bur. *toroy* 'piglet', Kalm. *torä:* 'piglet', Ordos *torö:* 'young donkey'

Proto-Turkic **to:ru* 'young cattle/camel/horse'

OT (Karakhanide) *torum* 'young camel, camel colt', *torpï* 'calf which still follows its mother' Tk. *deve torunu* 'two-years-old camel' (*deve* ‘camel’), Tkm. *to:rum* 'camel colt (between 6 months and one year)', Tat. dial. *to:rbaḳ*, *turbaḳ* 'heifer', Kirgh. *toropoy* 'piglet', *torpoḳ* 'calf between 6 months and 1 year old; dial. calf; bear-cub (less than 1 year old); silly (of a person)', Uigh. *topaq-torum* 'young cattle (coll.)', *topak* 'one-year-old calf', Tuva *dorum* 'camel colt (in the second year)', Salar *torï* 'foal', Bash. *tana-torpo*, dial. *tana-turpaḳ* 'heifers and bull-calves (coll.)' (*tana* 'heifer'), Alt. *torboḳ* 'bull-calf in the second year', Khak. *torbaχ* 'one-year-old calf; calf in the second year', *torbaχ puɣa* 'bull-calf in the second year' (*puɣa* 'bull'), *torbaχ-turbaχ* '(bull-)calves in the second year (coll.)', MChul. *torβaḳ* 'calf in the second year', Shor *torbaḳ* 'heifer in the third year', Tuv. *dorum* 'camel colt (in the second year)', Yak. *torbos*, *torbuyaχ* 'calf'

Note that Kumyk *toray* 'child', KKalp. *toray* 'piglet', Nogh. *toray* 'piglet', Kaz. *toray* 'piglet' and Tof. *toray* 'bear-cub' are borrowings from Mongolian

**(3) Proto-Mongolo-Tungusic **uli* ‘pig’**

Proto-Tungusic **uli-gan* ‘pig’

Negidal *olgịn*, Sibe *vǝlǝǵan*, Manchu *ulǵan*, Jurchen **ulhian*, **uliyan*, **u(l)gia*, Ulcha *orgị(n),* Orok *orgị(n),* Nanai *olgịã*, Udighe *wagê*, Solon *ulgē̃*

The Proto-Tungusic form was borrowed into the ancestor of the Nivkh language as **ulgVn* > > Nivkh *olghong* [o:loŋ] ‘swine’

Khitan **uil(e)* 'pig' (Kane 2009: 111)

*2.5. Reconstructions for textile production*

The etymologies (1) to (3) show that the ancestral speakers of Transeurasian were familiar with making rope and sewing, but these activities are not necessarily associated with agriculture (Nelson et al. 2020). However, the reconstructions in (4) and (5) for weaving cloth and spinning refer to more sophisticated processes of textile production that are usually not performed by hunter-gatherers. Our archaeological database and analysis (SI 7) indicate that spindle whorls are one of the most diagnostic archaeological traits of Neolithic expansions in Northeast Asia.

The reconstruction of a verb for cutting cloth in (6) can be associated with the stone knives used to cut hemp and other products during the Neolithic in Northeast Asia, in absence of scissors (An 1955).

Proto-Transeurasian textile vocabulary tends to get lost in Turkic and Mongolic languages, which acquired a more pastoral vocabulary in the Bronze Age, but it is well preserved in Japonic and Koreanic languages, as shown in the etymologies (7) to (11). This seems to indicate that Transeurasian-speaking populations moving eastwards with agriculture retained more ‘traditional’ technologies such as textile production whereas those moving westwards were more exposed to west Eurasian technologies and replaced their vocabulary accordingly.

The separation between borrowings and inherited forms among words for textile fibres in Japonic and Koreanic languages mirrors the provenance and timing of the introduction of these fibres. In line with the introduction of splicing technologies for ramie and hemp from Korea into Japan at the beginning of the Yayoi (3000 -1550 BP) period, words for bast fibres such as hemp and ramie in (10) and (11) reconstruct back to the ancestral Japano-Koreanic language. By contrast, the word for ‘silk’ (Section 3.2 (2)) was borrowed from Chinese at a later time in the Middle Yayoi period, when sericulture was introduced from China.

**(1) proto-Transeurasian **nap*- ‘to make rope’**

Proto-Japonic **nap*- ‘to make rope’ + *-*a* deverbal nominalizer

J *nau* (B), OJ *nap*- ‘twist, plait, weave (into rope)’, J *nawa* (2.3) ‘cord’, OJ *napa* 'rope', Yamatohama (Amami) *noo* ‘fishing line’, Yoron (Amami) noo ‘fishing line’, Yonamine *naa* ‘cord’ (Okinawa), Shuri (Okinawa) *naa* ‘cord’, Old Shuri *na:*, Hirara (Miyako) *na:*, Nagahama (Miyako) *na:*, Tarama (Miyako) *na:*, Ishigaki (Yaeyama) *na:*, Yonaguni *Nna*, PR **nawa* ‘cord’

Proto-Koreanic **nap*- ‘twist, spin’

K *nah*- ‘spin, weave, make yarn’, K *kkunapwul* ‘ a string of cord’ < *kkun* ‘cord, string’ + **nap*- ‘twist, twine, spin’ + -*wul* deverbal nominalizer, Kyeylim Yusa phonogram EMK *na(h)* ‘string’, KN *napi-* ‘to quilt, sew’, HB *ney-*, *yey-*‘to quilt, sew’, JJ *napi* ‘quilting; quilted work’, CN *napi* ‘quilting; quilted work’

Proto-Tungusic **nap*- ‘to make rope’ + *-*ki* resultative nominalizer (Robbeets 2015: 407)

Ulcha *lāxị*, Orok *lāpụ*, Na. *lāpị*, Oroch *lappi* ‘tiers, straps (for skis)’

Proto-Tungusic lacks initial liquids, except **l*- going back to original nasal **n*- assimilation before labial consonants (Poppe 1960: 74, Robbeets 2005: 69).

Twining can produce cloth, string or rope. Cords for making traps and nets have been found in a number of upper Paleolithic sites across the world (Tedlock 2009: 66, Soffer et al. 2000: 512-514). Therefore, making rope is generally older than weaving textiles.

**(2) Proto-Transeurasian **nup*- ‘to sew'**

Proto-Japonic **nup*- ‘to sew, stitch’

J *nuu* B*,* OJ *nup*- ‘to sew, stitch, embroider’, Yoron (Amami) *nuuju*ɴ 'to sew', Shuri (Okinawa) *noojuɴ*, Hirara (Miyako) *nuu* 'to sew', Igarashi (Yaeyama) *nooŋ* 'to sew', Yonaguni *nuŋ* 'to sew', pR **noCu*- 'to sew'

Proto-Koreanic **nupi*- ‘to sew, quilt’

K *nwupi* ‘quilting, quilted work’, *nwupi*- ‘to quilt; to knit one’s brow, to frown’, MK *nwu(·)pi*- ‘to quilt, sew (tr.)’, MK *nwu·pi* ‘quilting, quilted clothes; needlework or products of needlework, realized by inserting a filling between two layers of cloth and sewing them together’, KW *nwupi*-, KS *nwupi*-, *nipi*-, JL *nwupi*-, JJ *nwupi*-, CB *nwupi*-, *nipi*-, JN *nwimay*-, *nwipi*-, *nipi*-, JJ *nopi*, *nipi* ‘quilting’, *nopi*- ~ *noi-* ~ *nipi*- ‘to quilt’, KB *nwupey*/*nwupi*/*nwupwu*/*nwipey*/*nwipi*/*nwiin* ‘quilting’, *noi*- ~ *nuypi*- ‘to quilt’, KN *nipi*/*nwuipi*/*nii* ‘quilting’, *nipi*- ~ *nwui*- ~ *nwipi*- ‘to sew, quilt’, JN *nwipi*/*nipi* ‘quilting’, *nwiki*-/*nwimay*-/*nipi*- ‘to quilt’, JB *nwipi* ‘quilting’, *nwipi*- ‘to quilt’, KW *nwipi* ‘quilting’, *nwipi*- ‘to quilt’, CN *nwupin* ‘quilting’, *nwupi*- ‘to quilt’ CB *nwupi*/*nwupwu*/*nwipi* ‘quilting’, *nwipi*-/*nipi*- ‘to quilt’ KG *nwipi*-*ipwul*/*nipi* ‘quilting’, *nwipi*-/*nipi*- ‘to quilt’

Proto-Tungusic **nup*- ‘to prick, pierce’

Evk. *lupa*- ‘to prick’, *lupu:-* ‘to go through, pierce’, Even *nụbas an*- ‘to prick’, Neg. *lepu*- ‘to pierce’, Na. *loqpa*- ‘to prick (intr.)’, Olch. *loqpa*- ‘to prick oneself’, *loqpụ(n)* ‘a splinter’, Orok *lụkka*- ~ *lụqpa*- ~ *lupqa*- ‘to prick oneself, to impale oneself upon smth; to prick smth’

The Tungusic verb stem is probably a compound of pTg **nup*- ‘to prick, pierce’ with a suffix *-*kA*-, perhaps the alternant of the inchoative suffix pTg *-*xA*- in voiceless clusters (see Robbeets 2015: 259.) Poppe (1960: 74) finds that the initial *l*- in the Tungusic languages is a secondary development from an original **n*-: “Das anlautende *l* im Mandschu-Tungusischen ist sekundärer Herkunft und geht gewöhnlich auf ein anlautendes **n* (meistens vor einem folgenden **m*) zurück.” This view is consistent with the general absence of initial liquid phonemes across the Transeurasian languages. The environment in which this development takes place needs further study, but it should probably be extended to the position before *-*PK*- clusters, e.g. pTg **nabga:n*- ‘to glue, stick’ in Evk. *labgan*-, Even *nabgan*-, Neg. *labga:n*-, Orok *lamba*-, Ud. *lagbamu*-; pTg **nobgi* ‘squirrel nest’: Evk. *lopi* (dial. loki:); Neg. *lo:bị*, Ulcha *logbụ*, Na. *lo:bị*, Ud. *loi;* pTg **napki* ‘tiers, straps (for skis)’: Ulcha: *la:xị*, Orok *la:pụ*, Na. *la:pị*, Oroch *lappi*; pTg **napku*- ‘to insert, hang: Evk. *lapku*-, Even *napkü*-. Note that Even consistently retains the initial nasal here.

Sewing enters the archaeological record with leather clothing, and is generally older than weaving textiles.

**(3) proto-Transeurasian **sili*- 'to sew’**

Proto-Koreanic **sili* 'thread' < ? pK **sil(i)-* 'to sew, tie together'? + *-*i* deverbal noun; (Robbeets 2015: 459)

K *sil ‘*thread; a thin fine thing; a long narrow thing’, MK ̈ *sil* ‘thread; thin strip made out of cocoon, fur or hemp, used for weaving or needlework’, JN *silkkwuli* ‘thread’, KB *ssil* ‘thread’, JJ *sil* , KN *sil*, JN *sil*, JB *silta*, KW *sil*, CN *sil*, CB *sil*, KG *sil* ‘thread’

Given the rising tone, MK ̈ *sil* ‘thread’ can be derived from an original polysyllabic form, whereby the contraction was likely to be due to the equality of the vowel in both syllables, thus pK **sili* 'thread'. Given the reconstruction of a deverbal noun noun suffix pK *-*i* ~ *ø*, attested in e.g., MK *hal*- 'to slander' → MK *hali* 'slandering' and MK *nwu·pi*- 'to quilt' → *nwu·pi* 'quilting', it is possible that the Korean form originally derived from a verb pK **sil(i)-* 'to sew, tie together'. Note that the deverbal derivation from 'to sew, tie together' as 'thread' is also attested in the Tungusic languages, e.g. Na. *sera*- ‘to sew together (cords, threads)' → *sera-ča* ‘cord, thread'.

Proto-Tungusic **sira*- 'to sew together, tie together'

Evk. *sira*- ~ *hira*- ‘to piece down, to lengthen, to add (cloth, belt)’, Even *hịraq*- ‘to piece down, to lengthen, to add (a belt, a rope), to connect, to add’, *hịraqan* ‘phalanx of a finger’, Neg. *seya*- ~ *siya*- ‘to lengthen, continue a belt, a rope; connect two parts’; *siya:n* ~ *siyə:n* ‘thread; way (figurative)’; *siyəktə* ‘thread’; Orok *sịra*- ‘to lengthen, to piece down; to add a belt, a rope etc.; to connect together ends of a rope, a thread etc.’; Ma. *sira*- ‘to continue, follow; to connect, tie together; to inherit’, *sirame* ‘next (in sequence); step- (e.g. *sirame ama* ‘stepfather’; *siran* ‘continuation, succession, sequence, order’; Sibe *sira*- ‘to inherit; connect; to join; to continue’; Jur. *sir(a)-ru* 'inherit'; Olcha *sịra*- ‘to connect’, *sịra-ǯụ*- ‘to add; to marry a widow of the elder’s brother; to inherit’, *sịra-čụ*-*ǯụ*- ‘to tie together’; Na. *sera*- ‘to sew together cords, threads; to continue telling a fairy-tale which was interrupted’, *sera-go*- ‘marry a widow of one’s elder brother; keep on fire of one’s clan; to tie together two cords’, *sera-kta*- ‘to tie together several cords, threads’, *sera-ča* ‘knot (to lengthen a cord, a thread); a cord, a thread (for lengthening)’; Oroch *siya*- 'to sew together, piece down'; Ud. *sǣ*-, *sǣ-si*- ‘to piece down, to lengthen (cloths), to sew together (climbing skins); to add (a rope, a belt)’; *sǣ* ‘cross joint of climbing skins (in piecing down)’

Proto-Mongolic **siri*- 'to sew, stitch, quilt'

WMo. *siri*- '1 to quilt, stitch (tr.)', Khal. *šire*- 'to make firm by sewing, to quilt', Bur. *šere*- 'to stitch (usually the sole of a shoe)', Kalm. *šir*- '1', Ordos *šire*-, *širi*- '1', Dgx. *šïri*- '1', Dag. *širi*-, *šire*- '1', Eastern Yugur *širǝ*- '1', Mgr. *śiri*-, *śirǝ*- 'to cover a bed with a counterpane'

Proto-Turkic **sïrï*- 'to sew, stitch, quilt'

Karakhanid *sïrï*- 'to sew, to stitch, to quilt or smock a garment', Tk. *sïrï*- 'to sew tightly, quilt', Az. *sïrï-* 'to quilt; to foist, impose', Tkm. *sïra-* 'to sew, to stitch, to quilt', Tat. *sïr*- 'to quilt', Kaz. *sïr-* 'to stitch', Nog. *sïrï-* 'to quilt', Bash. *hïr-* 'to quilt', KKalp. *sïrï-* 'to sew across; to quilt', Kum. *sïrï-* 'to sew; to quilt; to fasten, attach'*,* KBalk*.* sïrï- ‘to quilt’, Uig. *širi-* 'to quilt', Khak. *sïrï-* 'to sew; to quilt', Tuva *sïrï-* 'to sew; to quilt'

**(4) proto-Transeurasian **pɔrɔ*- ‘to weave (cloth)’**

Proto-Japonic **orə*- ‘to weave’

J *oru* A ‘weave’, OJ *oro_2_s*- ‘deign to weave’, Asama (Amami) *Ɂujuɴ* 'to weave', Shuri (Okinawa) *Ɂujuɴ* 'to weave', Irabu (Miyako) *uï* 'to weave', Ishigaki (Yaeyama) *uruɴ* 'to weave', Yonaguni *úrun* 'to weave'

The expected reflex of pTEA **p*- is **p*- in proto-Japonic and proto-Koreanic However, an initial labial stop sporadically drops before a (long?) rounded pJK **o(:)*, as it probably also did in the reflexes of pTEA **bɔ:l*- ‘to sit down, become, be’ in Japanese (pJ **wo*- ‘to sit, be’ in OJ *wi*- ‘to sit, be’, OJ *wor*- ‘to be, exist’) and Korean (pK **o*- ‘to be’ in MK *-^.^w^u^/o-* modulator). Old Japanese makes no distinction between *o_1_* (< **o*) and *o_2_* (< **ə* ) in initial position, but I have opted for **o* in pJ **orə*- ‘to weave’ because it entails a regular external correspondence. The root-final vowel of pJ **orə-* is an irregular fit, which may be due to vowel reduction in root-final position.

Proto-Koreanic **olʌ* ‘unit of woven fibers, component of woven fabric’

K *o:l* ‘strand of rope, ply, warp’, MK ¨*wol* ‘ply; classifier for plies; a strand of thread or a string’, KB *sil-olayki*, *sil-olayi*, *olakci*, *oli*, *hol*, *hool*, JN *sil-olaki*, *sil-ol*, *olaki*, *olakci*, *ollakci*, *olakcci*, *olayki,* KN *ool*, *olay*, *olakcci*, KS *olayki*, CN *olayki*, CB *oskatak* ‘ply, string’, JJ *ol* , JN *ol*, JB *olki*, CN *ol*, CB *ol*, KG *ol* ‘strand of rope’, K *olk*- ‘to tie up, bind, weave’ (< pK **olʌ* ‘woven fabric’ + ·*kʌ*- inchoative; Robbeets 2015: 258)

Proto-Tungusic **poro*- ‘to spin (nettle and hemp threads); to rotate, turn’

Evk. *horol*- ‘to spin, whirl, go around’, *horoli:* ‘around’, Neg. *xoyil*- ~ *xoyol*- ‘to spin; to circle; to eddy (about water)’, *xoyil* ~ *xoyol*, *xoxsin* ‘eddy’, Ud. *xoli*- ‘to circle (about birds); to circle, to whirl’, Sibe *foro*- ‘to spin; to turn’, Ma. *foro*- ‘to spin; to turn, to turn around, to face, to turn toward’, *forko* ‘spinning wheel’, *foron* ‘swirl, curl, whirl; rotation (of an arrow between the fingers)’, *foro-no*- ‘to turn (in that direction)’, *forontu* ‘curly, having curly hair’, Olcha *pori*- ‘to weave (nets)’, *porpu(n*) ‘a spindle; a device for weaving nets’; Oroch *porpụ* ‘a spindle for spinning nettle and hemp threads’

The regular reflexes of pTg **p*- are Nanai/ Olcha/ Orok *p*-, Manchu *f*-, Evenki/ Even *h*-, Negidal/Oroch/ Udehe *x*- and Solon Ø (Benzing 1955: 981). Except for Oroch *po:rpu*, *po:rfu* ‘spindle’, which is probably a borrowing from Olcha, the cognates are thus corresponding regularly and suggesting the reconstruction of an initial pTg **p*-.

Proto-Mongolic **poro*- ‘to tie around, entwine; rotate, turn’ in **poro-go*- ‘to wrap’ (*-*gA*- causative) and **poro-ti*- ‘roll, rotate’ (*-*ti*- intensive)

WMo. *oriya*- ‘1 to tie around, entwine, wrap, bandage, wind, roll (tr.)’, *oruɣa*- ‘1’, *orči*- ‘2 to turn around, roll, rotate’ (intr.)’, MMo. *hura*- ‘1’, *xorči*-, *horči*-, *orči*- ‘2’, *orčul*- ‘2’, Khalkha *orō*- ‘1’, *orči*- ‘2’, Buriat *oŕo:*- ‘1’, *oršo*- ‘2’, Kalmuck *ora:*- ‘1’, *orčǝ*- ‘2’, Ordos: *oro:*- ‘1’, *orčin* ‘around’, Dgx. *xoro*- ‘1’, Baoan *horǝ*-, Dagur *oŕe:*-, Eastern Yughur *horo:*-, Mgr *furo:*-, *xuro:*- ‘1’

The initial labial stop pMo **p*- is regularly preserved in the peripheral Mongolic languages, notably as *f*- in Monguor *furō*-, as *h*- in Shira-Yughur *horō*- or Baoan *horǝ*- and as *x*- in Dongxiang *xoro*-, but it disappeared in the central Mongolic languages.

Proto-Turkic **pö:r*- ‘to plait, weave’

OT (Karakh.) *ör*- ‘to plait (hair or other fibers)’, MTk *ör*- ‘1 to weave, plait, twist things together’, *örmek* ‘cloth woven from camel hair’, Kirg. *ör*- ‘1’, Kaz. *ör*- ‘1’, *örĭm* 'woven part of sth (e.g. of a whip); bundle', Nog. *ör*- ‘1’, Bash. *ür*- ‘1’, Karaim *ör*- ‘1; to spin’, Kkp. *ör*- ‘1’, Tat. *ör*- ‘to plait, to knit, to darn, to interlace, to interweave, to build (a wall), to lay bricks or stones in a building’, Tk. *ör*- ‘1’, Az. *hör*- ‘1; to knit’, Tkm. *ö:r*- ‘1’, Gag. *yör*- ‘1; to knit’, Uz. *ọr*- ‘1’, Uig. *ö(r)-* ‘1’*,* Yak. *ör*- ‘1’, *örǖ* ‘plaiting’, Dolg. *ör*- ‘to plait, bind together, wind’, *örǖ* ‘plaiting’, Khalaj *hiri*-, *hör*- ‘to plait’, Chu. *var* ‘best part; sort of fiber; flax', *vĕren* ‘cord, rope’

For Turkic, it is commonly assumed that word initial pTk **p*- developed over a bilabial fricative into *h*-, leaving only a trace in Khalaj *h*- and finally disappeared in most of the contemporary Turkic languages. Given the attestation of Khalaj *hör*- ‘plait’ it is legitimate to reconstruct pTk **pö:r*- ‘to plait, weave’.

Weaving is labor-intensive and technologically complex, requiring a loom system. Only a society with food-surplus can invest in the technology and labor required (Barber 1995). Therefore, weaving is generally linked to agriculture.

**(5) proto-Transeurasian **tɔmʊ*- 'to spin’**

Proto-Japonic **tumu* 'spindle'

J *tumu* (2.4), OJ *tumu* 'spindle', J *tumug*- (B), OJ *tumug*- ‘to spin, make into yarn’, Yamatohama (Amami) *Ɂumuri-*, Yoron (Amami) *umjuN*-, Yonamine (Okinawa), Shuri (Okinawa) *ciNzjuN*-, Old Shuri *ɕiNzuN*-, Hirara (Miyako) *m:*-, Nagahama (Miyako) *tsïmal*-,Tarama (Miyako) *mmi:*-, Ishigaki (Yaeyama) *umu*- ~ *bu:un*-, Kohama (Yaeyama) *ɸuŋ-,* Taketomi (Yaeyama) *m:muŋ-,* Yonaguni *umuŋ*-, PR **tumu*- ‘to spin (thread) (tr.)’

Proto-Tungusic **tom(u)-* > *tumu*- 'to spin' + *-*ku* ~ *-*ko* deverbal instrumental noun suffix, e.g. Na. *xado*- 'to mow' → *xadoko* 'scythe'

Even *tum*- '1 to spin, wind, coil, spool, wrap', *tumenŋe* 'thread wind around a bobbin', *tomqo*- ‘to spin strings for threads; to spin threads’, *tomqon* ‘spinning of strings; yarn’; Evk. *tum*- ' 1', *tomko* ‘a thread’; *tomko*- ‘to spin threads; to tie with a thread’; Neg. *tum*- '1', *tumu* 'string of thread, parcel, roll', *tomko* ‘thread’, *tomko*- ‘to spin threads’; Solon *tum*- 'to bind, knit, string together, tie', *toŋxo*- ‘to spin threads’; Oroch *tumu*- '1', *tompo* ‘a sinew or nettle thread’, *tompo*- ‘to spin threads; to weave a net’; Olcha *tumu*- '1', *toŋpo* ‘short threads of nettle or hemp’, *toŋpo*- ‘to spin threads’; Orok *tumu*- '1', *toqpo*- ~ *topqo*- ‘to spin threads, rope, cord’; *toqpo* ~ *topqo* ‘thread; rope’; Na. *tumu*- '1', *tompo*- ‘to spin threads of fishskin’, *tompo* ‘threads made of fishskin’; Ud. *tompo*- ‘to spin threads, ropes’

The Tungusic reconstruction displays a vowel alternation between **tom(u)-* and **tumu*- 'to spin'. Given the preservation of **tom(u)-* in derived nouns with the instrumental suffix and re-verbalizations thereof, I assume that **tom(u)-* assimilated to **tumu*-.

Proto-Mongolic **tomu*- ~ **tamu*- 'to spin'

WMo. *tomu*-, *tamu*- '1 to twist or spin thread or rope', MMo. *tamu*- ~ *toma*- ~ *tomu*- ~ *doma*- '1', Khal. *tam*- ~ *tom*- '1', Bur. *tomo*- '1', Ordos *tamu*- '1', Kalm. *tom*- ~ *töm*- 'to twist, twine; to string together (rope), make rope (by turning horse hair between the hands)', Eastern Yugur *tomu*- ~ *tɔmɔ*- ~ *tomə*- '1', Dgx. *tomu*- '1', Bao. *tomǝl*- '1', Mgr. *tomu*- ~ *tamu*- '1'

In Mongolic we find a vowel alternation between **tomu*- ~ **tamu*- 'to spin', reminiscent of the alternation between pMo **dolaan* 'seven' and **dalan* 'seventy' (Nugteren 2011: 512).

**(6) proto-Transeurasian **giri*- 'to cut (cloth)'**

Proto-Japonic **kira*- ‘to cut (e.g. cloth)’

J *kir-* B*,* OJ *ki_1_r-* ‘to cut, shear, chop’, OJ *ki_1_ras-* B ‘to run/ sell out of’, J *kireru* B*,* OJ *ki_1_re-* ‘to be sharp, get cut, run out’, J *kiri* 'texture', J *kiri*, OJ *ki_1_ri* 'awl, pointed tool for piercing small holes (as in cloth or leather)', Yamatohama (Amami) *kiruri-*, Asama (Amami) *k^Ɂ^ijuN-*, Yoron (Amami) *kjuN-*, Yonamine (Okinawa) *ciN-*, Shuri (Okinawa) *cijuN-*, Old Shuri *ciyuN-*, Hirara (Miyako) *kïsï-*, Nagahama (Miyako) *tsï:-*, Ikema (Miyako) *sï:tsï-*, Ōgami (Miyako) *kiʃi-*, Tarama (Miyako) *kï:-*, Ishigaki (Yaeyama) *kïsuŋ-*, Hatoma (Yaeyama) *ʃisuŋ-*, Hateruma (Yaeyama) *ssuŋ-,* Kohama (Yaeyama) *kïsï-,* Taketomi (Yaeyama) *ʃiʃuŋ-,* Yonaguni *ccuŋ-*, PR **kir*- ‘to cut’

Proto-Tungusic **giri*- ‘to cut out (e.g. cloth, paper, pelt)’

Evk. *gir*- ~ *ger*- ~ *kir*- ‘to cut out (with scissors)’, *giri-ptun* ‘cloth piece’, Even *gịr*- ‘to cut out’, Neg. *gey*- ~ *giy*- ‘to cut out’, Ma. *giri*- ‘to trim with a knife or scissors, to cut evenly, to cut a strip’; *giri-n* ‘strip; section, area’; *gir*-*dan* ‘cloth or strips of pelts cut with scissors; evenly cut slices of meat; pennant; border trim on a banner’; *giriku*: ‘a small knife for trimming skin, paper, and cloth’, Olcha *gịrị*- ‘to cut out’, *gịrsụ* ‘woman’s knife for cutting things out’, Orok *gịrị*- ‘to cut out’, *gịrị-ptụla* ‘cloth pieces, remnants’, Na. *gere*- ‘to cut out with a knife’, *gerego*- ‘to recut out, to cut out anew’, *gerekta*- ‘to cut out many objects’, *gerekto* ‘cut out’, *geremsa*, *gereptola* ‘cloth rags’, *gereče*- ~ *gerenase*- ‘to cut out many times’, *gerso:* ‘woman’s knife for cutting objects out’, Oroch *gi:-* ~ *giyi*- ‘to cut out with a knife’, Ud. *gi:*- ~ *gi:na*- ‘to cut out with scissors’

Proto-Turkic **kïr*- ‘to cut, scrape’: OT (Karakh.) *kïr*- ‘to scrape, strip (hair), pluck out (hair), to cut off’, Tk. *kïr-* 'to break, split (wood); to rough-grind, crush (grain); to hurt, injure', Az. *ġïr-* 'to break; cut', Tkm. *ġïr-*'to scrape', Gag. *ḳïr-* 'to break, crack', Tat. *ḳïr-* 'to break; to scrape; to shave; to grate; to whet; chafe', Khak. *xïr*- ‘to cut; to scrape; to shave; to chafe', Kirg. *kïr*- 'to scrape; to shave', Kaz. *kïr*- 'to scrape; to shave; to chafe', Nog. *kïr*- 'to scrape; to shave', Bash. *kïr*-''to scrape; to shave; to grate; to chafe; to whet', Karaim *kïr*- 'to scrape; to shave', Kkalp. *kïr*- 'to scrape; to shave', Uz. *kir*- 'to scrape; to shave; to break', Uig. *ki(r)*- 'to scrape; to shave; to break', Yak. *kïrïy-* ‘to cut’, *kïra* 'small', Dolgan *kïrïy-, kïrpala:-* 'to cut', Tuva *kïr*- ‘to break; to exterminate all without exception', Khalaj *kïr*- ‘to break’, Chu. *xïr*- 'to scrape'

The Chuvash verb *xïr*- 'to scrape' has an irregular vowel correspondence and may thus be due to borrowing. The Mongolic forms WMo. *kira*-, *kiru*-, Khal. *x´ar*- and Kalm. *kur*- that support the reconstruction of pMo **kira*- ‘to cut into small pieces, mince’ have been omitted since they can be assumed to have been copied from Turkic. If they were cognates, we would expect an initial voiced velar (**g*-) in Mongolic.

The Senchū Wamyō Ruijūshō (AD 930), an Early Middle Japanese lexicon, among others covering vocabulary for textile, employs the verb MJ *kir*- 'to cut' as the standard verb for cutting in the production process of textile (Omura & Kizawa 2017: 455). The semantic context of cloth production is further supported by deverbal nouns meaning 'texture' or 'awl'. Simularly, given the deverbal nouns meaning 'strip of cloth or pelt', the Tungusic verb is specialized for the cutting of cloth, paper or pelt, while the Turkic verb is often used with 'hair' or 'pelt' as an object. Therefore, the common semantic denominator seems to be the cutting of strips of cloth and the verb may have its origin within a context of textile production.

**(7) Proto-Japano-Koreanic **parʌ-* ‘to sew’**

Proto-Japonic **paru-i* ‘needle’ < **paru*- ‘to sew’ + *-*i* deverbal noun suffix (Robbeets 2015)

J *hari* (2.4), Iwate *haru*, OJ *pari*, EOJ *paru* 'needle', Shuri *haai* (B) 'needle'

Proto-Koreanic **palʌ-l* < **palʌ-* ‘to sew’ + *-*l* deverbal noun suffix (Robbeets 2015)

K *panul*, MK *pa·nol*, LMK *palol* ‘needle’, CB *kwulkunpanul*, *topppanul*, KB *panol*, *toppanul*, *tosppanul*, *papal*, JB *panel*, *tokpanul*, CN *panwul*, *tokpanul*, KS *panwul*, *panel*, *pal*, *tokppanel*, *tospanul*, KW *panwul*, *panel*, *topppanul*, *tospanul*, CC *tospanul*, KN *ttoppanel*, *ttospanel*, *ttospal*, *ttosppanel*, JJ *panong*, JL *panwul* ‘needle’

In Middle Korean, *lVl* sequences are occasionally confused with *nVl* sequences, e.g. *anil soy* 'not being' is assimilated to *alil soy* ‘not being’. However, the change proposed here takes an opposite direction, assuming dissimilation from MK *palol* to *panol*, even if the attestation of MK *pa·nol* is older.

This etymology suggests that the nouns for ‘needle’ are independently derived in Japonic and Koreanic by way of separate deverbal noun suffixes, but that the underlying verb ‘to sew’ is cognate and goes back to a common form in proto-Japano-Koreanic.

**(8) Proto-Japano-Koreanic **paca*- 'to weave (cloth) with a loom’**

Proto-Japonic **pata* ‘loom, woven cloth’ < **pata*- ‘to weave’

J *hata*, OJ *pata* (2.2a) 'loom, woven cloth', Yamatohama (Amami) *hat^h^amuN*, Asama (Amami) *hatoN*,Yoron (Amami) *patamunu*, Yonamine (Okinawa) *nunuubat^Ɂ^aa* / *pat^Ɂ^aa*, Shuri (Okinawa) *nunubata*, Old Shuri *nunubata*, Hirara (Miyako) *patamunu*, Nagahama (Miyako) *patamono*, Ikema (Miyako) *hatamono*, Ōgami (Miyako) *nunubata*, Tarama (Miyako) *pata*, Ishigaki (Yaeyama) *pato:munu*, Hatoma (Yaeyama) *patumunu*,Taketomi (Yaeyama) *hataunu,* Yonaguni *hata*, PR **pata* ‘loom’, *pata*- ‘to weave (with a loom)’

The frequent compounding of *pata*- with *mono* ‘thing’ as a ‘thing for weaving’ in the Ryukyuan languages, may indicate a verbal origin of the Proto-Ryukyuan form **pata*- ‘to weave’. The polysemy between 'loom' and 'woven cloth' in the Japanese member of this etymology further supports the derivation of these nouns as instrumental and object nominalizations of an original verb 'to weave'. Note that the suffix -*a* is reconstructed as a deverbal noun suffix in proto-Japonic (Robbeets 2015: 156) .

Proto-Koreanic **pʌcʌ*- > **pcʌ*- 'to weave' (pK *-*i* deverbal nominalizer / adverbializer; *-*k* deverbal nominalizer, e.g. K *ilwu*- 'to achieve' → *ilwuk* 'achievement')

MK ·*pca*- 'to weave, knit, tie up', K *cca*- ‘to weave’, JJ *cas*-, *cha*-, *cho*-, *chwuk* *cha*-, KB *cca*-, KN *cca*-, KW *cca*-, CN *cca*- ‘to weave’

In Manchu we find *fatan* 'the sole of a foot or a shoe; comb-like tool used for working silk on a loom, weaver's reed', but the technical term is probably a semantic extension of pTg **pata* 'sole, bottom, fundament'. A similar polysemy exists for Korean *patak* 'cloth, weave, texture' and *patak* 'bottom, sole'. MK *potoy*  'healds of a loom, series of wires attached to the loom frame' may be related here as well.

**(9) Proto-Japano-Koreanic **pu*- 'to spin, twist (thread)’**

Proto-Japonic **pu* 'twisted thread, weaving'

J *hu*, OJ *pu* '(woven) stitch, mesh, weave, knit, knot', J *ya-hu* (eight-weave) 'many stitches in a woven fence'

In Japanese, we also find J *hi*, OJ *pi_1_* 'shuttle', but the quality of the front vowel contradicts derivation from **pu-i* (twist-NMLZ) because that would lead to a vowel of the quality *i_2_* in Old Japanese.

Proto-Koreanic **pu*- 'to twist (thread)' (pK *-*i* deverbal nominalizer / adverbializer; *-*k* deverbal nominalizer, e.g. K *ilwu*- 'to achieve' → *ilwuk* 'achievement')

Ki *pi*-, MK *puy*- 'twisted (bound adverb), e.g. in K *pi:-thul*-, MK *puy-thul*- 'to twist, contort', *pi:-kko*:- 'to twist (thread), twist up', *pikki*- 'be bent, lie at an angle', *pi-kkule*-*may*- 'to tie, bind'; K *pwuk*, MK *pwuk* ‘shuttle (loom instrument)’ (< **pu-k* twist-NMLZ), JJ *pi* 'shuttle' (< **pu-i* twist-NMLZ)

**(10) Proto-Japano-Koreanic **ʌsa* ‘hemp’**

Proto-Japonic **asa* 'hemp'

J *asa* (2.3) ‘flax (plant), hemp (plant), ramie, jute, linen’, OJ *asa* ‘hemp’, Kagoshima/Koshiki/ Miyazaki */*Kumamoto*/*Ōita*/*Fukuoka*/*Saga*/*Fukue*/*Nagasaki *asa* ‘hemp’, Shuri *Ɂasa* ‘hemp’, Yonamine *Ɂasaa* ‘hemp’, Kohama (Yaeyama) *basa* (< **bu: asa*),

Proto-Koreanic **ʌsa-ma*

K *sam*, MK *·sam* ‘hemp; a cannabaceous perennial plant. Its stems are thin and long, its compound leaves are palmate, and its individual leaves are lanceolate with fine saw teeth and thick, fine hair. It blooms from July to August dioeciously. Its fruit is similar to that of a dandelion but round-shaped. It is sowed and cultivated from March to May. Its seeds are edible and also used as medicinal oil, feed, or manure. The shell of the seeds is used as ingredients for textile, hemp cloth, net, or hawser’, JJ *maphey*, KB *sam*, KN *sampey*, JN *sam*, JB *sam*, KW *sam*, CN *kam* ~ *peyccachi*, CB *sam*, KG *sam po* ‘hemp’

The words for ‘hemp (*Cannabis sativa Linne*)’ in different Japonic and Koreanic languages seem to be compounds of three elements, all meaning ‘hemp’: **(a)sa*, **po* and **ma*. The latter element corresponds to the Sino-Korean loan morpheme *ma* (麻) 'hemp', e.g. in the Sino-Korean synonym *taema* (大麻）'hemp' (Francis-Ratte 2016). In Korean, we find it in the final -*m* of *sam* ‘hemp’, but also as the initial morpheme in Cheju *maphey* ‘hemp’. Some Ryukyuan forms such as Yoron (Amami) *mahuu* ‘hemp’ and Sesoko *maːwuː* include the element **ma* as well.

The Cheju and Yoron forms also include a reflex of the second element Proto-Japano-Koreanic **po* ‘hemp’. This element is also reflected in J *o*, OJ *wo* ‘hemp, flax’ as well as in Kyushu dialects, i.e. Miyazaki *wo,* Kumamoto*/*Ōita *o* and Ryukyuan languages, i.e. Hirara (Miyako) *bu:*, Nagahama (Miyako) *bu:*, Ikema (Miyako), Ōgami (Miyako) *pu:,* Tarama (Miyako) *bu: nunu*, Ishigaki (Yaeyama) *bu:,* Hateruma (Yaeyama) *bu:* Taketomi (Yaeyama) *bu:,* Yonaguni *bu:* (< PR **wo:* and PJ **wo* ‘hemp’). In Proto-Koreanic the element **po* ‘hemp’ is reflected in KG *sam po* ‘hemp’, KN *sampey*, JJ *maphey* and CN *peyccachi.*

The third element Proto-Japano-Koreanic **ʌsa* has a limited distribution in Japonic, restricted to Mainland Japanese and Kyushu dialects in addition to the Northern Ryukyan languages Shuri and Yonamine and the Southern Ryukyan language Kohama. OJ *so_1_* ‘hemp, cloth’ is probably not related with OJ *asa* ‘hemp’, but a possible cognate may be MK *swom* ‘cotton’ (Francis-Ratte 2016).

Whitman (1985: 232), Vovin (2010: 173) and Francis-Ratte (2016) suggest that the high tone of MK *·sam* ‘hemp' indicates the loss of an initial vowel in pK **ʌsam.* Francis-Ratte proposes that the final -*m* in the Korean form originates from a compound with the Sino-Korean loan morpheme *ma* (麻) 'hemp', e.g. in the Sino-Korean synonym *taema* (大麻）'hemp'. This analysis is supported by the Cheju word *maphey* ‘hemp’, which seems to incorporate *ma* ‘hemp’.

Along with the poor distribution of the word in Ryukyuan, the existence of an older Scythian form **sana* ‘hemp’ (cf. Old Indo-Aryan *śaṇ* ‘hemp’, Mayrhofer 1992–2001/2: 605; Gamkrelidze–Ivanov 1995: 570) may provide an alternative explanation for the Korean form as a *Wanderwort*, but the proposed common ancestorship is probably more sensible given the long history of the use of hemp in East Asia.

**(11) Proto-Japano-Koreanic **mosi*** **‘ramie (cloth)’**

Proto-Japonic **mosi* ‘ramie (cloth)’

OJ *karamusi* ‘ramie (Boehmeria silvestrii), Chinese ramie; ramie fabric, grass cloth’, OJ *musi* ‘ramie (Boehmeria silvestrii),’ e.g. in *musi-tareginu* ‘a ramie veil on the back of a woman’s hat’, OJ *musiro_2_* ‘*ramie* (Boehmeria silvestrii); straw mat’, J *musiro* ‘straw mat’, Yamatohama (Amami) *musiro*, Asama (Amami) *mussju*,Yoron (Amami) *mussjuu*, Yonamine musu (Okinawa), Shuri (Okinawa) *musiru*, Old Shuri *musiru*, Hirara (Miyako) *mussu*, Nagahama (Miyako) *mussu*, Ikema (Miyako) *mussu*, Tarama (Miyako) *mussu*, Ishigaki (Yaeyama) *musu*, Hatoma (Yaeyama) *musu* Hateruma (Yaeyama) *mussu,* Taketomi (Yaeyama) *mussu,* Yonaguni *musu*, PR **mosi-su* ‘straw mat’

Proto-Koreanic **mosi* ‘ramie cloth’

MK *mwosi* ‘ramie cloth; fabric weaved out of the fiber of the cover of ramie. It is smoother than hemp cloth and commonly used as summer cloth.’, K *mosi* ‘ramie fabric, ramie cloth’, JJ *mosay* ~ *mosi* ‘ramie cloth’, KB *mosi*, KN *mosi*, JN *mosul* ~ *mosi*, JB *mosey*, KW *mosi*, CN *mosi*, CB *mosi*, KG *mosi* ‘ramie cloth’

It has been proposed that the Old Japanese element *musi* ‘ramie’ in OJ *karamusi* ‘Chinese ramie’ is a borrowing from MK *mwosi* ‘ramie cloth’ because the Old Japanese word is a compound with *kara* ‘(Han) China, Korea, foreign’, indicating a foreign provenance and because it has a narrowed meaning and limited distribution in comparison with OJ *asa* ‘hemp’ and OJ *wo* ‘hemp’ discussed above (Frellesvig & Whitman 2008: 38; Unger 2009: 119; Francis-Ratte & Unger 2020: 708). However, against this viewpoint, it can be observed that ramie is native to Japan, that the word does not represent a merely narrowed meaning from ‘hemp’ and that, considering its derivation OJ *musiro_2_* ‘Boehmeria silvestrii; straw mat’, it is well distributed across the Ryukyuan languages.

First, ‘ramie’ is not a mere narrowed meaning derived from ‘hemp’. Both terms refer to bast fibers used for textile production in ancient Japan and Korea that are part of the local vegetation, but they designate two different plants: ramie is a perennial, flowering plant in the nettle family of the *Boehmeria* genus, while hemp is an annual and dioecious (having distinct male and female organisms) plant in the *Cannabaceae* family of the *Cannabis* genus. Unlike ramie, which grows in designated fields, hemp is seeded in rice fields or vegetable fields and harvested in late June or July when the rice or vegetable seedlings are transplanted. There is only one hemp plant being cultivated in Korea and Japan, but there are two kinds of ramie plant, aboriginal ramie and “China Grass” or “white ramie”, which has thicker and longer stalks and thus produces thicker yarns than the aboriginal kind (Hwang 2010). As such OJ *musi* probably refers to the native variant, while *karamusi* ‘Chinese ramie’ refers to the thicker variant imported from China.

The Jomon people already produced items made with bast fibres such as ramie and hemp, but these fibres were originally used without joining them into longer threads. Splicing of threads is likely to date back to the beginning of the Yayoi (3000 – 1750 BP) period, when innovative textile technologies were brought from Korea to Japan, leading to the development of weaving. Production of bast fibres for weaving dates further back than silk production, which is thought to have been introduced from China with sericulture in the Middle Yayoi period (Barnes 1993: 173; Omura and Kizawa 2017; see SI 7).

Given the introduction of spliced ramie for weaving at the beginning of the Yayoi period, OJ *musi* ‘ramie’ may be cognate with MK *mwosi* ‘ramie cloth’. Although the simplex word for ‘ramie’ is not well distributed across the Ryukyuan languages, the derived form for ‘straw mat’ is. The word can be derived with a collective suffix going back to proto-Japonic *-*ra* ~ -*rǝ*, used among others in the derivation of plant names and products thereof (Labrune 1998, Antonov 2007), e.g. OJ *asi* ‘reed’ → J *aziro* ‘wickerwork, a mat of split bamboo, a reed mat’, OJ *kinu* ‘silk’, *wata* ‘cotton’ → OJ *kinu-wata-ra* ‘clothing’, OJ *sasa* ‘dwarf bamboo, bamboo grass’ → J *sasara* ‘bambou percussion instrument’, OJ *tane_2_* ‘tissue’ → OJ *tane_2_-ro_2_* ‘tissue, cloth’, etc. The Ryukyuan forms reflect a stem **mosi-su* ‘straw mat’, in which the suffix may be assimilated to the sibilant in the stem.

**3. Language contact in the Bronze Age**

**3.1 Ancient borrowings from a Proto-Turkic model**

Ancient linguistic borrowings from Turkic are associated with agropastoral economies on the eastern steppes in the Bronze Age. They include terms for agricultural crops imported from the west such as ‘barley’ and ‘wheat’, livestock such as ‘cattle’ and ‘sheep’, exploitation of horses such as ‘stallion’ and dairying products such as ‘fermented butter milk’. Some of these words are the outcome of language-internal coining in Proto-Turkic, such as the proto-Turkic words for ‘wheat’, ‘stallion’ and 'fermented buttermilk', which are derived from verbs ‘to stir porridge’ and 'to separate'. Others such as ‘barley’ can be identified as borrowings from Eastern Iranian spoken in an area from the Aral Sea to the Altai in the first millennium BC.

These borrowings are transmitted from Proto-Turkic to Proto-Mongolic in the third millennium BP, before 2200 BP. Although words for ‘stallion’, ‘sheep’ and ‘lamb’ are borrowed into Proto-Tungusic by the turn of our era, dairying vocabulary such as ‘cattle’ and ‘fermented buttermilk’ and terms for crops such as ‘barley’ do not reach the Primorye until the last millennium BP.

The earliest evidence for herding of sheep and cattle and dairying being introduced from the western to the eastern steppes can be traced to ca. 5000 BP but the origins of horse-based pastoral economy on the eastern steppe goes only as far back as the late Bronze Age (ca. 3500-2700 BP). While horses first played a comparatively limited role in Early and Middle Bronze Age pastoral economies across both Mongolia and Central Asia, around 3200 BP horses started to take an important role due to the innovation of mounted horseback riding. The relatively late adoption of horse-based nomadic culture on the eastern steppe is consistent with the dating of the borrowings to the late Bronze Age. Sheep and horses do not appear in the Primorye until the late Iron Age, while cattle and dairying appear much later. This observation is in line with the timing of the borrowings below.

**(1) BARLEY**

Proto- Indo-European **h_2_elb^h^-* ‘white’ -> **h_2_elb^h^i-t-* ‘barley; barley flour’ ? > Eastern Iranian **arbasya:* ~ *arpasya:* >> Proto-Turkic **arba* ~ **arpa* ‘barley’ >> Proto-Mongolic *arbai* ‘barley’ >> Manchu *arfa* ‘barley, oats’

Eastern Iranian **arbasya:* ~ *arpasya* (Blazek 2019)

Pashto *orbǝša*, Wanetsi *arbasa* ‘barley’, Khwar. *rsy* barleycorn’, Khot. *rrusā*-, obl. *Ruśe ‘*barley’, Sangleči *urwəs / vərvəs*, Yidgha *yεršio*, Wakhi *arbəsi*, Pashto *orbəša*, pl. *ōrbašē*, dial. Afridi *warbaše*, Wanetsi *urbūsa /arbasa* pl. *arbaši/ arbusi / arbəsē*, Waziri *rebəše*

Proto-Turkic **arpa* ~ **arba* ‘barley’

OT (Old Uighur) *arpa* ‘barley’, MTk. *arpa* ‘barley’, Az. *arpa* ‘barley; a kind of women's jewelry resembling barley’, Bashk. *arpa* ‘barley; stye (on the eye)’, Crim Tat. *arpa* ‘barley’, Gag. *arpa* ‘barley’, Kar. *arpa* ‘barley’, Kaz. *arpa* ‘barley’, KBalk. *arpa* ‘barley’, Khak. *arba* 'barley; roasted barley', Kirgh. *arpa* ‘barley’, KKalp. *arpa* ‘barley’, Kumyk *arpa* ‘barley’, , Noghai *arpa* ‘barley’, Oir. *arba* ‘barley’, Sal. *are^p^a* ‘barley; a kind of wheat (with small ears)’, Tat. *arpa* ‘barley; stye (on the eye)’, Tkm. *arpa* ‘barley’, Tur. *arpa* ‘barley’, Uzb. *arpa* ‘barley’, Uigh. *arpa* ‘barley’, Khalaj *arpa* ‘barley, Hordeum vulgare, Hordeum distinctum’, Chuv. *urba* ‘barley; stye (on the eye)’ (Bulgharic or Early Kipchak **arpa* >> Hungarian *árpa* ‘barley, Hordeum’). Sal. *arfa* ‘barley’ is borrowed from Manchu and Tuv. *arbay* ‘barley’ from Mongolian.

Proto-Mongolic *arbai* ‘barley’

MMo. *arbǝi*, *arbăi*, *a:rbăi* ‘barley’, WMo. *arbai*, Khal. *arvay*, Bur. *Arbay*, Ordos *arwǟ*, Kalm. *arwǟ, arwā*, Dongxian *apa*, Mogol *arfɛi, arfā*, Mgr. *šbǝ̄* ‘spelt’

Manchu *arfa* ‘barley, oats’

Proto-Japonic **apa* ‘Foxtail millet (*Setaria italica*)’

J *awa* ‘Foxtail millet (*Setaria italica*)’, OJ *apa* ‘millet’, Yamatohama (Amami) *Ɂoo*, Asama (Amami) *Ɂoo*,Yoron (Amami) *oo*, Yonamine (Okinawa) *ʔawaa*, Shuri (Okinawa) *Ɂawa*, Old Shuri *Ɂawa*, Hirara (Miyako) *a:*, Nagahama (Miyako) *a:*, Ikema (Miyako) *a:*, Ōgami (Miyako) *a:*, Tarama (Miyako) *a:*, Ishigaki (Yaeyama) *a:*, Hatoma (Yaeyama) *a:*, Hateruma (Yaeyama) *a:,* Kohama (Yaeyama) *a:,* Taketomi (Yaeyama) *a:,* Yonaguni *a:*, PR awa ‘Foxtail millet (*Setaria italica*)’

Barley was domesticated in the Fertile Crescent about 10 000 BP. Via the Near East and South Asia, it ultimately reached central and eastern China by the third millennium BP (Boivin et al. 2012: 457; Liu et al. 2018). In our database (SI 6, SI 7) barley is not found in the West Liao or Primorye regions in the Bronze Age. Barley was part of the farming package consisting of millet, rice, wheat and other crops, which expanded very quickly across Bronze Age Korea and Japan.

There are indications that the term for ‘barley’ was borrowed across the Transeurasian languages following an eastward trajectory. Its ultimate source probably lies in the Eastern Iranian branch of Indo-European, spoken over most of Central and Western Asia in the first millennium BC and expanding as far east as the Upper Yenisei in the Altai mountains, where Proto-Turkic speech communities were situated.

Ultimately, it may be possible to trace this word back to Indo-European **h_2_elb^h^i-(t-)* ‘barley; barley flour’, which is an early derivation form pIE **h_2_elb^h^-* ‘white’. This form may be reflected in Greek *alfi* ‘barley flour or groats’, Albanian *elp*, *elpbi* ‘barley’ and Eastern Iranian **arbasya:* ~ *arpasya* ‘barley’, reflected in various eastern languages. Hyllested (2020) has recently argued against inheritance from ancestral Indo-European, pointing instead to a Central Asian substrate word. If this is indeed the case, the Central Asian substrate word or the underived base of the Eastern Iranian words probably served as a model for Proto-Turkic **arpa* ~ **arba* ‘barley’. The Turkic forms lead to the reconstruction of pTk **arpa* ‘barley’, but variation with **arba* cannot be excluded on the basis of the alternation in Old Turkic and Chuvash and the Siberian Turkic reflexes.

From Eastern Iranian the term was borrowed into proto-Turkic, which in its term served as a model for Proto-Mongolic. The voiced alternant pTk **arba* was borrowed into proto-Mongolic, where it was suffixed with an element –*i*, perhaps in analogy with WMo. *buɣudai* ‘wheat’. This borrowing probably took place before the break-up of Proto-Turkic around 2200 BP. The Siberian Turkic form Tuvan *arbay* is a later reborrowing from Mongolian.

As the word is not attested in Jurchen or other Tungusic languages, the borrowing from the Mongolian word into the Manchuric branch of Tungusic may be rather recent, dating back to the second millennium AD. This is consistent with the absence of the crop in the Primorye regions in the Bronze Age. The absence of a glide and the presence of a fricative in Ma. *arfa* indicate that it may be borrowed after the thirteenth century from Western Mongolic, i.e. the ancestor of Kalmuck and Oirat. In the thirteenth century, the Oirat moved from the south of Lake Baikal to the Altai region, from where they dispersed over various regions, including Western Mongolia, Manchuria and the Xinjiang, Gansu and Qinghai provinces in China. The Kalmuck were forced to emigrate from their original homeland in northern Xingjiang to the Volga region in the seventeenth century. The Salar Turkic form *arfa* is probably a reborrowing from Manchu.

Although Proto-Japonic *apa* ‘millet’ is a look-alike, the similarity with Proto-Turkic **arpa* is probably purely coincidental. As cognates of this word are well distributed in the Ryukyuan languages, the adoption of the word and its meaning shift must have happened before Proto-Japonic separated, i.e. before 300 BC. Given the similarity with Turkic, the word should have been borrowed directly from Proto-Turkic, when both languages were situated on the continent. The relatively late adoption of barley in Est Asia taken together with the problematic semantic shift from ‘barley’ to ‘foxtail millet’ in Japonic makes this scenario rather unlikely.

**(2) WHEAT**

Proto-Turkic **bod*- ~ **bud*- ‘to soak (cereals), stir (porridge)’ + -*xA* ~ -*kA* deverbal resultative noun (Robbeets 2015: 411) > **budgay* ~ **bugday* ‘wheat’ >> Proto-Mongolic **bugudaï* ‘wheat’

Proto-Turkic **bodgay* ~**budgay* ~ **bugday* ‘wheat’

Old Turkic *buɣday*, *budɣay* 'wheat', OT (Karakhanid) *buɣday* 'wheat', Chag. *buɣday* 'wheat', MKypch. *boɣday*, *buɣday*, *boday*, *buday*, *buyday* 'wheat', Azer. *buɣda* 'wheat, wheat grain', Gag. *bo:day*, *buuday* 'wheat', Tk. *buɣday*, dial. *buɣda*, *boɣda* 'wheat', Tkm. *buɣday* 'wheat', Crim Tat. *boɣday* 'wheat', Salar *boɣde*, *boɣdï*, *poɣde* 'wheat', Khalaj *buɣda*, *boɣda* (<< Az.) 'wheat, Triticum vulgare, Tr. Sativum’, Tat. *boday* 'wheat', Bash. *boyðay* 'wheat', Karaim *buday* ~ *bogday* ~ *boɣday* 'wheat', KBalk. *buday* 'wheat', KKalp. *biyday* 'wheat', Kumuk *buday* 'wheat', Kaz. *biday* 'wheat', MChul. *pu:day* 'wheat', Nogh. *biyday* 'wheat'; *ḳara* *biyday* 'rye' (*ḳara* 'black'), Oir. *buuday* 'wheat', Shor *puɣday* 'wheat', Uigh. *buɣday* 'wheat', Uzb. *buɣdɔy* 'wheat', Chuv. *pə^w^ri* 'emmer wheat'

Proto-Mongolic **bugudai* ‘wheat’.

MMo. *bu’udai* ~ *buɣdai* ‘wheat’, WMo. *buɣudai*, *buudai* ‘wheat’, Khal. *buuday* ‘wheat’, Ordos *bu:dä:* ‘wheat’, *buidä:* ‘wheat’, Kalm *buudya* ‘grain’, Eatern Yughur *bogdüi* ‘wheat’, Huzu Mgr. *bu:də* ‘wheat’, Minhe Mgr. *bidi*, Bao *baoġdəi*, *bɵġdi*, *boġdi*, Kgj. *bəġdi* ~ *baġdi*, Dgx *baudəi*, *baodəi* ‘wheat’, Mog *buɣdäi*, *bʉydäi*, *bi:da:* ‘wheat’

Our archaeological database shows that wheat (*Triticum aestivum*) was introduced earlier to Central and East China than barley: by around 4600 BP it was present in Shandong (SI 6, SI 7). Like ‘barley’, the borrowing of the word for ‘wheat’ also reflects an eastward direction, from Proto-Turkic into Proto-Mongolic.

Given the attestation of Old Turkic *budɣay* 'wheat', Turkic words reflecting the shape **bugday* may go back to an early process of metathesis in Proto-Turkic. The ultimate source of Proto-Turkic **bodgay* ~**budgay* may be a deverbal resultative noun in -*xA-i* ~ -*kA-i* of the Proto-Turkic verb * *bod*- ~ **bud*- 'to stir (porridge)', reflected in Yakut *butuj*- 'to stir (porridge)'. The direction of the borrowing is supported by the fact that Proto-Mongolic only borrowed the Proto-Turkic metathesized alternant **bugday* and by the lack of morphological segmentability in Mongolic.

**(3) STALLION**

Proto-Turkic **adïr*- ‘to separate’ + *-*g* deverbal object noun / + -*xA* ~ -*kA* deverbal resultative noun -> pTk **adrï-g* ~ **adgïr* / **adïr-ga* ‘non-castrated male’ >> proto-Mongolic **adirga* 'male animal, stallion' >> proto-Tungusic **ajirga* 'stallion'

Proto-Turkic **adgïr* ‘non-castrated male animal’

OT (Türkü) *adɣïr*, (Uygh) *adɣïr* ‘stallion’, MTurk. (Chag) *ayɣïr* ‘stallion’, (Kypch) *ayɣïr* ‘stallion’, Az. *ayɣïr* ‘stallion, stud-horse’, Bash. *ayɣïr* ‘stallion’, *ayɣïr ügeð* ‘non-castrated bull’ (*ügeð* ‘bull’), Crim Tat. *ayɣïr* ‘stallion, lustful, lascivious’, Dolgan *atï:r* ‘Reindeer-breeding stallion, bull’, *atï:r at*, *at atï:ra* ‘breeding stallion’ (*at* ‘horse’), *atï:r börö* ‘male wolf (*börö* ‘wolf’), Gagauz *χayɣïr* ‘stallion; lustful, lascivious’, KBalk. *ayɣïr* ‘evil, angry; irrepressible, fiery; stallion, a hero’s horse’, Karaim *ayɣïr* ‘stallion’, Kaz. *ayɣïr* ‘stallion’, *ayɣïr žuwa* ‘wild leek’ (*žuwa* ‘leek, onion’), Khak. asχïr ‘stallion’, Kirg. ayɣïr ‘stallion’, *ayɣïr at* ‘gelded horse’ (*at* ‘horse’), KKalp. *ayɣïr* ‘stallion’, Kum. *ayɣïr* ‘stallion’, MChul. *asqər*, *asχər* ‘stallion’, *äygir* ‘black riding horse’, Nog. *ayɣïr* ‘stallion; bellicose, dashing, brave (of a woman)’, Oir. *ayɣïr* ‘stallion’, Shor *asqïr* ‘stallion’, *asqïr aŋ* ’male sable’ (*aŋ* ‘sable’)

SYugh. *azɣïr* ‘stallion’, Tat. *ayɣïr* ‘stallion’, Tof. *asqïr* ‘stallion; male of Siberian musk deer’

Tkm. *ayɣïr* ‘male donkey or horse; ardent, fervent’, dial. ‘five-year stallion; donkey’

Tur. *aygïr* ‘stud-horse; bumpkin, tough man’, Tuv. *asqïr* ‘stallion’, *asqïr ït* ‘male dog’ (ït ‘dog’), *asqïr qas* ‘male goose, gander’ (*qas* ‘goose’), Uygh. *ayɣir* ‘stallion’, Uz. *ayɣir* ‘stallion’, Yak. *atï:r* ‘stallion; (non-castrated) male (pig, wolf, bull); big, strong, powerful’, Chuv. *ə̑yə̑r(ə̑),* *ïyə̑r(ə̑), ïŕə̑* ‘stallion’

Proto-Mongolic **adirga* ~ **ajirga* 'male animal, stallion'

MMo SH *ajirqa*, *aǰirɣa*, WMo. *aǰirɣa(n)* ‘stallion, before the names of other animals, it means the male animal’ Khalkha *ajirga,* azraga(n) 'stallion', Bur *azarga* ‘stallion’, Kalm *aǰrɣ*, Dag. *adirag*, *adyrəɣ,* *aǰrəɣ*, *adrəɣ, ad́irga*, Eastern Yughur *ajirɣa*, Minhe Monguor *aǰirga,* Mog. *aǰərɣa-du ira*- ‘to mate (said of female animals)’.

Proto-Tungusic **ajirga* 'stallion'

Evk. *ajirga*, Ma. *ajirgan*, Jurchen *ajir*, Na. *ajirga* ‘stallion’

Although pastoralism was practiced on the Eastern Steppe from ca. 5000 BP onward, there is no evidence for dietary exploitation of horses on the Eastern Steppe prior to the late Bronze Age, ca. 3200 BP – at which point horses come to dominate ritual assemblages, play a key role in pastoral diets, and influence pastoral mobility (Taylor et al. 2020). In our database (see SI 6, SI 7), horses were reported from 9 sites, with finds from the West Liao, Ordos and Heilongjiang regions in the late Bronze Age. The horses from Neolithic Haminmangha and Houtaomuga may be wild or due to contamination. Horses are not known in the Primorye until the Iron Age. Horses only reach Japan from the end of the fourth century AD. This relatively late adoption of horse-based nomadic culture on the Eastern Steppe, the West Liao region and the Primorye are corroborated by the evidence of borrowing horse terminology in from west to east in the late Bronze Age, such as the word for ‘stallion’ discussed here.

Judging from the semantics in the contemporary Turkic languages, the original Turkic meaning is ‘non-castrated male animal’ rather than being restricted to ‘non-castrated male horse’, as witnessed by Yakut, Dolgan, Tuvan, Tofalar, Shor, Bashkir, etc. This supports the derivation as the 'separated one', as non-castrated animals need to be kept separate from the herd. Therefore, Proto-Turkic **adgïr* ‘non-castrated male animal’ can be analyzed as deverbal noun from Proto-Turkic **adïr*- ‘to separate’. The observation that this morphological segmentability is lacking in Mongolic supports the direction of the borrowing from Turkic into Mongolic. Besides, the restriction of ‘non-castrated male’ to secondary semantics in Mongolic ‘male animal, male horse’ and Tungusic ‘male horse’ is also indicative of the direction of the borrowing.

**(4) BOVINE**

Proto-Turkic **pökü* ‘ox, bull, cattle’ + **-r_2_* collective suffix >> Proto-Mongolic **püker* ‘ox, cow’ >> Proto-Northern Tungusic [**pukur*](https://starling.rinet.ru/cgi-bin/response.cgi?single=1&basename=%2fdata%2falt%2ftunget&text_number=1878&root=config) ‘ox, cow’

Proto-Turkic **pökü-r_2_* ‘ox, bull, cattle’

OT (Türkü, OUygh.) *öküz* ‘ox’, (Xak.) *öküz* ‘bull, the constellation Taurus’, MTk. (Kypch.) *öküz* ‘a castrated bull’, Az. *öküz* 'bull', 'ox', Bashk. *ügeδ* 'bull, ox', Crim Tat. *ögüz* 'ox', Gag. *Öküz, yöküz* 'ox', Kaz. *ögiz* ‘bull, ox’, KBalk. *ögüz* 'ox', Kirgh. *ögüz* ‘ox, gelded bull’, KKalp. *ögiz* ‘bull, ox’, Kar. *ögüz, egiz* ‘cattle’, Kum. *ögüz* ‘ox’, Nogh. *ögiz* ‘ox, bull’, SUig. *qus* ‘cow, ox, bull’, Tat *ügez* ‘bull’, Tur. *öküz* ‘bull, ox’, Tkm. *öküz* ‘bull, ox’, Uigh. *Öküz, höküz* ‘ox’, Uz. *họkiz* ‘ox’, Yak. *oɣus* ‘bull’, Chu. *vъ^w^gъ^w^r* 'bull'

Proto-Mongolic **püker ~ *pöker* ‘ox, bovine’

MMo. *huker*, *hüger*, *hüker*, *üker*, WMo *üker* ‘bovine animal, ox, cow; large, big; the second of the twelve animals of the zodiac’, Khal. *üxer*, Ordos *üker*, Bur. *üxer*, Kalm. *ükr* ‘cow’, Dag *xukur*, *hukure*, Eastern Yughur *hgor*, Huzu Mgr. *fugor*, *xgor*, Minhe Mgr. *xugor*, *χukur*, Bao *fgor*, *gor*, *xgur*, Kgj. *gɵr*, Dgx *fugie*, *fuġə*, *fuġər*, *fugər*, Mog *ʉkȧr*, *ükär* [*ʊkʌr*] ‘ox, bovine’

The Eastern Yughur, Monguor, Baoan and Dongxian forms support the reconstruction **pöker* rather than **püker*.

Proto-Northern Tungusic **pukur* ‘ox, cow’

Evenki *hukur*, Even *höken*, *hökön*, Solon *uxur* 'ox'

Domesticated cattle (*Bos taurus*) were introduced from West Asia by the third millennium BC. This corroborates the eastward direction of borrowing of the word for ‘bovine’, first from Proto-Turkic into Proto-Mongolic in the Bronze Age. Only five sites in our archaeological sample have reported cattle remains (SI 6, SI 7). In the Primorye, cattle appear only after the Iron Age, which is consistent with the relatively late borrowing of the word in Northern Tungusic.

In addition to the problematic vowel correspondence in Tungusic, the borrowing of the word for ‘ox’ is supported by the morphological complexity of the Turkic form, probably containing a petrified plural or collective suffix, used to indicate the collectivity of the species. The plural or collective -*z* was no longer productive in Old Turkic, but it is attested in a number of petrified forms including the plural pronouns *biz* ‘we’ and *siz* ‘you’, paired body parts such as OT *kö-z* 'eyes', *ti-z* 'knees', *agï-z* 'lips' and *kökü-z* 'breasts' and ethnonyms such as OT *ogu-z* and *kïrgï-z*.

Given that the morphological segmentability is restricted to Turkic, the word for ‘ox’ must have been borrowed from Proto-Turkic into Proto-Mongolic. The transmission probably took place before the break-up of Turkic ca. 2200 BP because Mongolic has copied the original Proto-Turkic initial **p*- and the coda liquid * -*r_2_* as such, whereas these sounds developed into a zero initial and a coda -*z* in all Common Turkic languages. It is unlikely that the form was borrowed from Proto-Bulgharic into Proto-Mongolic because the Proto-Bulgharic form was borrowed into Hungarian as *ökör* and we can thus assume that the initial **p*- had already lenited in Proto-Bulgharic.

The word for ‘ox’ was further transmitted to Tungusic, where it has a limited distribution in the Northern Tungusic languages. This observation suggests that the borrowing took place before the break-up of these languages around ca. 750 BP but after the separation between Southern and Northern Tungusic languages ca. 950 BP.

**(5) SHEEP**

Proto-Turkic **koni* ‘ram, sheep’ >> Proto-Mongolic **koni* ‘sheep’ + *-*n* stem-final singular suffix >> Proto-Tungusic **konin* ‘sheep’

Proto-Turkic **koni* ‘ram, sheep’

OT (Türkü) *qoń* ‘sheep’, (Xak.) *qoy* ‘sheep’, MTk. (Chag., Xwar.) *qoy* ‘sheep’, Az. *Goyun* 'sheep, ram', Bash. *quy* ‘fat-tailed sheep’, Crim Tat. *qoy*, *qoyun* 'sheep', Karaim *qoy*, *koy* ‘sheep’, Kaz. *qoy* ‘ram’, KBalk. *qoy* 'sheep, ram', Khak. *xoy* ‘sheep (generic term)’, Khalaj *qo:n* ‘sheep’, Kirgh. *qoy* ‘sheep (generic term), year of the sheep’, KKalp. *qoy* ‘sheep, ram’, Kum. *qoy* ‘sheep’, Nogh. *qoy* ‘sheep, ram’, Oyr. *koy* ‘sheep (generic term)’, S.-Yugh.  *qoy* ‘sheep’, Tat. *quy* ‘fat-tailed sheep’, dial. ‘sheep’, Tofa *hoy* ‘sheep, ram’, Tur. *koyun* ‘ram, sheep’, Tkm. *Goyun* ‘sheep, ram’, Tuv.  *xoy* ‘sheep’, Uigh. *qoy* ‘sheep, ram’, Uzb. *qọj* ‘ram, sheep’

Proto-Mongolic **koni-n* ‘sheep’

MMo. *qoni(n),* *qonin*, *ɣonin*, WMo. *qoni(n)* ‘sheep; the eighth year in the 12-year cycle; period from 1 to 3 PM’, Khal. *xon’* (*xonin*), Ordos *χoni*, Bur. *xoni(n),* Kalm. *xön* (gen. xöönä), Dag. *xɔny*, EasternYughur *χɔ:nə*, *χo:nə*, Huzu Mgr. *xonə*, Minhe Mgr. *qoni*, Bao. *ġonə*, *xonə*, *ġɵni*, Kgj. *χɔni* ~ *χuni*, Dgx. *ġoni*, Mog *qonin*, *qɔnin* ‘sheep’

Proto-Tungusic **konin* ‘sheep’

Evenki *konin*, Kamnigan Evenki *konin*, Solon *xonin*, Amur/Birare Solon *konin*, Manchu *xonin*, Jurchen *xoni*, Kili *xonin*, Nanai *xonin*, Olcha *xonin*, Orok *xonin* ‘sheep’, Udehe *xuani* ‘mutton’ (borrowed from Manchu).

Domesticated sheep (*Ovis* sp.) are reported from northern China as early as the fifth millennium BC. Although the vowel and consonant correspondences between the three protoforms are regular, it is generally agreed that the word for ‘sheep’ was borrowed from Turkic into Mongolic into Tungusic (Doerfer 1985, Starostin et al. 2003, Janhunen 2012). The first borrowing of the word for ‘sheep’ postdates 6800 BP, the time that Proto-Turkic separated from Proto-Mongolo-Tungusic. If the word for ‘sheep’ is adopted around the same time as the term for ‘lamb’ below, the borrowing probably predates the primary split of the Turkic languages around 2200 BP. This dating corroborates the timing in our archaeological database (SI 6, SI 7), where sheep were reported at 4 Neolithic and 8 Bronze Age sites.

The observation that the primary meaning of the Turkic word is specialized for gender ‘male sheep, ram’, while the Mongolic meaning is restricted to the generic term for ‘sheep’, supports the borrowing scenario. It is clear that the direction of the borrowing was from Mongolic into Tungusic rather than the other way around because the proto-Tungusic form **konin* ‘sheep’is morphologically unsegmentable, while the proto-Mongolic form is a derived form with the stem-final singular suffix *-*n*. This borrowing has taken place before the primary break-up of the Tungusic languages, i.e. before ca. 2000 BP, a dating, which is in line with the late Iron Age appearance of sheep in the Primorye (SI 6). The Udehe word for ‘mutton’ is borrowed after 350 BP from Manchu within a culinary context.

**(6) LAMB**

Proto-Turkic **kor_2_ï* ‘lamb’ >> Proto-Mongolic **kuri-gan* ‘lamb’

Proto-Turkic **kor_2_ï* ‘lamb’

OT (OUygh.) *quiz* ‘lamb’, (Xak.) *quzï* ‘lamb’, Az. *Guzu* 'lamb', Crim Tat. *qozu* 'lamb', Gag. *quzu* 'lamb', Kaz. *qozï* ‘lamb’, KBalk. *qozu* 'lamb (younger than 6 months old)', Khal. *quzï* ‘lamb’, Kirgh. *qozu* ‘lamb (domestic or wild)’, KKalp. *qozï* ‘lamb’, Karaim *kozu* ‘lamb’, Kum. *qozu* ‘lamb’, MTk. (Chag., Xwar.) *quzï* ‘lamb’, Nogh. *qozï* ‘lamb’, Sal. *ko(:)zï*, *ku:zï*, *qu:zï* ‘lamb’, SUig. *qozï* ‘lamb’, Tat. *quzï*, *quzïy* ‘lamb’, Tur. *kuzu* ‘lamb’, Tkm.  *Guzï* ‘lamb’, Uig. *qoza*, *qozi* ‘lamb’, Uzb. *qụzi* ‘lamb’

Proto-Mongolic **kuri* ‘lamb’ + *-*gAn* diminutive suffix

MMo. *quriqa(n),* *qurixan*, *qəriɣan*, WMo. *quriɣan*, *quraɣa(n),* *qurɣa(n*); Khal. *xurga(n),* Ordos *χurġa*, Bur. *xuŕga(n);* Kalm. *xurɣn*, Mog. *qɔrɣan*, *qurɣana*, *qurğʌn*, Dgx. *quɣan*, *ġuğaŋ*; Bao. *Ġurġaŋ*, Kgj *ğurğun*, Eastern Yughur *χurğan*, Huzu Mgr. *xurġan*, Minhe Mgr. *qurġur*, *kurga* ‘lamb’

The irregular vowel correspondence between Turkic **o* and Mongolic **u* indicates that the word for ‘lamb’ is a borrowing. The Middle Mongolian forms in the old documents suggest an original trisyllabic **kurigan*. The suffix *-*GAn* is a petrified diminutive suffix, e.g., MMo. *keü* -> *keüken* ‘child’, MMo. *sain* ‘good’ -> *saiqan* ‘beautiful, good-looking’, MMo. *unaɣan* ‘foal, colt’, etc. Since the Turkic word lacks the diminutive suffix, it is clear that the direction of the borrowing is from Turkic into Mongolic. As the Mongolic forms have a liquid -*r*-, where the attested Turkic forms have a fricative -*z*-, the borrowing has probably taken place before the primary split of Proto-Turkic, i.e., before 2200 BP. Proto-Turkic *-*r_2_*- developed into -*z*- in the Common Turkic branch, whereas the Bulgharic branch with Chuvash as the only surviving representative preserved *-*r*-.

**(7) FERMENTED BUTTERMILK**

Proto-Turkic **adïr*- ‘to separate’ + *-*(X)n* deverbal noun ➝ **adrïn* > **adran* > Proto-Bulgharic **ayran* >> Proto-Oghuric **ayran* 'separated matter such as whey or fermented buttermilk’

Proto-Turkic **adïr*- ‘to separate’ + *-*(X)k* deverbal noun ➝ **adrï-k* 'separated matter such as peat' > Proto-Bulgharic **ayrïk*  >> Proto-Mongolic **ayirag* ‘fermented buttermilk’

Proto-Oghuric **ayran* ‘whey; fermented drink made from buttermaking by-products from milk of cows, sheep and goats’

OTurk. (Xak.) *ayran* ‘buttermilk’, *ayran yaɣ-ï* ‘fresh and melted butter’ (*yaɣ* ‘butter’), MTk. (Kypch.) *ayran* ‘butter-milk, sour milk’, Az. *ayran* ‘buttermilk, ayran, skimmed milk’, Bash. *ayran* ‘ayran, buttermilk’, Crim Tat. *ayran* ‘ayran, whey; buttermilk’, Gagauz *ayran* ‘ayran’, Karaim *ayran* ‘ayran, whey’, Kaz. *ayran* ‘ayran, varenets, kefir, curdled milk’, KBalk. *ayran* ‘ayran’, Khak. *ayran* ‘ayran’ Kirg. *ayran* ‘ayran, buttermilk’, KKalp. *ayran* ‘ayran’, Kum. *ayran* ‘ayran’, Nog. *ayran* ‘ayran, buttermilk’, Oir. *ayran* ‘ayran’, Tat. *äyrän* ‘ayran, buttermilk’, Tkm. *ayran* ‘ayran’, Tk. *ayran* ‘ayran, buttermilk’, Uygh. *ayran* ‘ayran’, Uz. *ayrɔn* ‘ayran’, Chuv. *uyran* ‘buttermilk’

Proto-Mongolic **ayirag* ‘fermented buttermilk of mares or donkeys’

MMo. *ayirax*, WMo. *ayiraɣ* ‘kumys, fermented buttermilk of mares or donkeys’, Khal. *ayrag*, Bur. *ayrag*, Kalm. *ä:rǝg*, Ordos *ä:raq*, Dag. *airag*

Kamnigan Evenki *aörak* ‘sour milk, buttermilk’

Ma. *ayara* ‘sour milk, buttermilk’

Olcha *ayara*- ‘to take off fat (while melting)’, *ayaraqụ* ‘spoon for taking fat off’

Na. *ayaraχo* ‘spoon for taking fat off’

Recent research has provided direct molecular evidence suggesting that dairy pastoralism was transferred from the Western to the Eastern Eurasian steppe in the Late Bronze Age through cultural transmission rather than through population replacement (Jeong et al. 2018, Wilkin et al. 2020). There is evidence for dairying in Central Mongolia by around 3000 BC; see SI 6. This location is to the west of the Altaic homeland and the dating postdates the separation of Turkic from Mongolo-Tungusic. It is, therefore, in line with our finding that the Altaic languages did not inherit dairying vocabulary from a common ancestor (Section 2.2.), but acquired it independently through a chain of borrowings. Similar to the transmission of domesticated ruminants, there is evidence for a West to East direction of cultural diffuson of dayiring products in the first millennium BC: from Eastern Iranian people, descendants of Andronovo who lived as far as the Altai mountains, words for dairying products were transferred to Turkic-speaking populations in present-day Mongolia and from there to Mongolic speakers in present-day Northeast China. Dairying reached the Primorye only after the Iron Age. This is illustrated by the borrowing of the word for *ayran*.

A common view (e.g., Sevortjan 1974: 111) is that Proto-Turkic **ayran* ‘whey; fermented buttermilk’ derives from a basic verb ‘to separate’. Semantically this derivation is plausible because ‘whey’ is the watery part of milk that is separated from the coagulable part or curd in the process of making butter or cheese and *‘ayran*’ is the watery part of milk separated from the coagulable part in the process of making butter, mixed with water and salt and fermented.

Morphologically, the derivation can be explained through the deverbal noun suffixes *-(*X)k* and *-*(X)n*, such as in the derivation of, e.g., *sa*- ‘to count’ -> *san* ‘number, set of things counted’, *tüg*- ‘to bind a knot’ -> *tügün* ‘knot’, *käl*- to come’ -> *kälin* ‘bride’ and, e.g., *al*- ‘to take’ -> *alïk* ‘habits’, *kat*- ‘to mix, to add on’ -> *katïk* ‘a condiment such as vinegar or churned milk, something mixed in anything’, *sogï*- ‘be cold’ -> *sogïk* ‘coldness; cold’. Note that the derivation of Proto-Turkic **adr-ïk* 'separated matter; peat' is reflected in OT *aδrïk*, *ayrïk* ‘peat’.

However, the derivation of *ayran* is phonologically problematic because the reflex of Proto-Turkic **d* is **d* in Proto-Oghuric in the East and **y* in Proto-Bulgharic in the West of the Turkic speech community. Whereas some reflexes of the Proto-Turkic verb **adïr*- ‘to separate’ preserve an Oghuric reflex of the original **d*, e.g. OT *adɨr*-, Karakhanid *aδɨr*-, Tuva/ Tofalar *adɨr*-, Yakut *atɨr*-, Khalaj *hadru*-, *hadur*-, none of the Turkic forms for *ayran* reflects this **d*. Therefore, we assume that Proto-Oghuric borrowed the forms for *ayran* with **y* from Proto-Bulgharic at an early stage. This puts the time of the borrowing in the late Bronze Age, after the primary break-up of Proto-Turkic around 2200 BP and before the break-up of Proto-Oghuric around 1700 BP.

As the Turkic form is originally morphologically segmentable, whereas the Mongolic word for fermented buttermilk’ is not, the direction of the borrowing is clearly form Turkic, probably Proto-Bulgharic into Proto-Mongolic around the same time. *Kumis* is fermented buttermilk, but unlike *ayran*, which is made from the milk of cows, sheep and goats, it uses the milk of mares or donkeys as it base.

Given the phonologically irregular correspondences between the Tungusic forms, they do not reconstruct back to a single common Proto-Tungusic form, but rather represent later borrowings from individual Mongolic languages, probably over the last millennium.

- 1. **Ancient borrowings from a Proto-Mongolic model**

In the third millennium BP, we observe two borrowing chains crossing Eurasia from west to east. In addition to a northern borrowing chain from Eastern Iranian (between the Aral Sea and the Altai) to Proto-Turkic (in present-day Mongolia) to Mongolic (West Liao and present-day northeastern Mongolia) to Tungusic (Khanka-Ussuri), discussed in the previous section, there is a southern borrowing chain from Tocharian (Tarim Basin) to Tibeto-Burman (Tibet) to Sinitic (Yellow River) to Proto Mongolic to Turkic and/or Tungusic. Whereas the northern borrowing chain involves the transmission of words for western crops such as barley and wheat, ruminants such as bovines and sheep and dairy products, the southern chain transfers words relating to horse breeding, such as ‘horse’ and ‘mare’ to the Transeurasian languages. In contrast to the northern borrowing chain, in which Turkic serves as the hub for Transeurasian, these words are spread from a Mongolic model into Turkic and Tungusic. Whereas the spread into Turkic dates to the third millennium BP, the spread of these terms to Tungusic is more recent, taking place after the beginning of our era or even in the Middle Ages.

**(1) HORSE**

Proto-Indo-European **mark(o)* ‘horse’ ? > Tocharian ? >> Proto-Tibeto-Burman **mraŋ* ‘horse’ >> Old Chinese**馬** **mˤraʔ* ‘horse’ >> Proto-Khitan-Mongolic **mori* ‘horse’ + *-*n* stem-final singular suffix >> Proto-Tungusic **murin* ‘horse’

Proto-Tibeto-Burman **s/m-raŋ* ‘horse’ (Matisoff n.d.)

Tujia *ma^53^*‘horse’, Gazhuo *m^31^*‘horse’, Yidu (Deng) *ma^55^ ɹoŋ^53^*‘horse’, Darang *mɑ³¹ɹoŋ⁵* ‘horse’, Kaman *pɑ³¹xoŋ³⁵* ‘horse’, Lai (Hakha) *ràŋ* ‘horse’, Wancho *man* ‘horse’, Jingpho *ma³¹ lau³³ ‘*fodder, feed (horse)’, Sak *məráŋ* ‘horse’, Pattani *mà gri* mane (of horse, lion)

Guiqiong *mbu^35^*‘horse’, Zhaba (Daofu County) *mbʐo¹³* ‘horse’, rGyalrong *mbra rme* mane (of horse, lion), *mbro* ‘horse’, Nung *mɛn³¹* horse, Achang (Lianghe) *m̥jɑŋ³¹* horse’, Bola (Luxi) *mjɔ̃³¹* horse’, Burmese (Rangoon) *mjĩ^55^* ‘horse’, Burmese (Written) *mrâi  /mrâñ* ass, Lipo *mu^21^* ‘horse’, Nesu *mo^33^* ‘horse’, Nusu (Central) *mɹə⁵⁵* ‘horse’, Nusu (Bijiang) *mɹɯ³⁵* ‘horse’

Yi (Weishan) *a⁵⁵ m̩²¹* ‘horse’, Yi (Wuding) *mv̩³³ ‘*horse, year of the horse’, Ahi *mo^21^* ‘horse’

Sani *mu^21^* ‘horse’, Yi (Sani) *m̩⁵⁵ ‘*horse, year of the horse’

Old Chinese**馬** **mˤraʔ* ‘horse’ (Baxter & Sagart 2014)

Proto-Khitan-Mongolic **mori* ‘horse’ + *-*n* stem-final singular suffix

MMo *mori, morin, murin,* WMo *mori(n),* Dag. *moryi, mory,* Khal. *mory,* Bur. *mori(n)*

Ordos *mori(n)*, Kalm. *mörn*, Oirat *mörn*, Moghol *morin, muren*, Shira-Yughur *mōrǝ*

Mgr. *mori*, Dgx. *Mori*, Baoan *more*, Khitan **m(o)ri* ‘horse’

Proto-Tungusic **murin* ‘horse’

Jurchen *murin*, Sibe *morin*, Manchu *morin*, Even *muran*, Evk. *morin, murin*, Neg. *moyịn*

Solon *morĩ*, Olcha *murin*, Orok *murin*, Oroch *muri(n)*, Udihe *muyi,* Nanai *morĩ*

Proto-Koreanic **mʌl* ‘horse’

Korean *mal*, HB *mali*, *moli*, GN *mol*, JL *mol*, HG *mol*, MK *mol* ‘horse’,

Proto-Japonic **uma ~ muma ‘horse’*

J *uma*, MJ *(m)uma* ‘horse’, Miyako *nuuma*, Yaeyama *nnma*, Hateruma *qman*/*nman* ‘horse’

Horse-based nomadic culture was introduced to the eastern steppe around 3200 BP and there is evidence for horse-riding from the Qijia culture (4200-3600 BP) in the Upper Yellow River region in Gansu and eastern Qinghai (Flad et al. 2007), but it had not yet encroached upon the northern edges of East Asia by that time (Barnes 1993: 157; Taylor et al. 2020). The first evidence for horse-riding in Northeast Asia goes back to the third millennium BP; see SI 6, SI 7. By that time, two innovations are apparent in Upper Xiajiadian culture sites, namely the presence of animal-style bronzes and the addition of the horse to the faunal repertoire. Horses are not known in the Primorye until the Iron Age and do not reach Japan before the end of the fourth century AD.

The long borrowing chain for the term for ‘horse’, reaching all the way from Indo-European to Tibetan to Old Chinese to Mongolic and Tungusic and the observation that the parallels extend to numerous non-Transeurasian languages as well suggest that the horse was introduced to East Asia in a rapid wave of cultural influence.

One possible Indo-European reconstruction for the word for ‘horse’ is **mark(o).* It is reflected in Proto-Germanic **márx-a*- ‘horse’ (e.g. in Old Norse *mar-r* ‘horse’, German *Mähre*, Eng. *mare*, Dutch *merrie*, Danish *mær*, Swedish *märr*, etc. ‘steed, female horse’) and in Proto-Celtic **mark* (e.g. in Irish / Gaelish *marc*, Welsh *march*, Breton *marc’h* ‘horse’, etc.). There are no Tocharian or Iranian cognates for this root preserved, but it is not impossible that the word reached the Tibeto-Burman speakers on the eastern edge of the Tibetan plateau through contact with Tocharian, the speakers of which lived in the Tarim Basin in Northwest China in the third millennium BP. The linguistic ancestors of Proto-Tocharian are associated with the Qäwrighul culture, situated south of the Altai in the fourth millennium BP (Mallory & Adams 1997: 593) and with the Afanasievo culture (5000-4500 BP) in the Altai in the fifth millennium BP (Kroonen et al. 2019), but these connections remain to be demonstrated.

The term for ‘horse’ was later transferred to Sinitic. The irregular lack of the nasal ending in OC 馬**mˤraʔ* signals that the term for ‘horse’ is not cognate, but has been borrowed within the Sino-Tibetan family (Sagart et al. 2019).

From Old Chinese, the word for ‘horse’ was transmitted into proto-Mongolic. Given that the Old Chinese final glottal stop was developing into a tonal structure and that proto-Mongolic lacked initial consonant clusters, the closest imitation of the Chinese root in proto-Mongolic probably was **mori*. In the Mongolic languages we find an unstable stem-final nasal element, morphophonologically alternating with zero, that expresses singularity in contrast with plural forms on -*d*. This stem-final -*n* was added to the simple stem, yielding pMo **mori-n*.

It is clear that the direction of the borrowing was from Mongolic into Tungusic rather than the other way around because the Proto-Tungusic form **murin* ‘horse’ is morphologically unsegmentable, while the Proto-Mongolic form is a derived form. The borrowing of Proto-Tungusic **murin* ‘horse’ happened around the turn of our era but the Tungusic forms of the shape *morin* are late Ming (1368-1644) borrowings. From Tungusic the word spread to non-Transeurasian languages such as Nivkh, e.g. the terms for ‘horse’, Sakhalin Nivkh *murng* and Amur Nivkh *mur.*

Old Chinese**馬** **mˤraʔ* ‘horse’ was transmitted separately into proto-Korean as **mol* and into proto-Japanese as **(m)uma*. Beckwith (2007) reconstructs the Old Koguryo word **meru* ‘colt’. Given the phonological discrepancy, the word cannot be reconstructed back to proto-Macro-Japonic, the common ancestor of Koguryo and Japonic. The disyllabic structure and the presence of a liquid rather suggests that the word is a separate borrowing from Mongolic or Tungusic. In the final centuries BC, the Koguryo people were attested in the western part of present-day Liaoning Province, west of the Liaodong Peninsula, where they were in contact with Tungusic, Mongolic, Turkic and Chinese people.

The initial **(m)u-* in Proto-Japonic **(m)uma* may have been added, in an attempt to imitate the initial cluster in Old Chinese. The Middle Japanese variant *(m)uma* ‘horse’ as well as the Ryukyuan cognates Miyako *nuuma*, Yaeyama *nnma* and Hateruma *qman*/*nman* support this idea. In addition, the Japanese imitation of Old Chinese**梅** **C.mˤə* ‘plum tree’ is *ume* ‘plum’ and it has a similar variant *(m)ume* ‘plum’ in Middle Japanese. This example seems to indicate that Old Chinese glottalized labial nasal clusters were imitated by way of a prothetic *(*m)u*- in Proto-Japonic.

All Japanese horse breeds can be descended from Mongolian horses that migrated through the Korean Peninsula and arrived in Japan about 2,000 years ago (Tozaki et al. 2003). According to our archaeological database (SI 6, SI 7), horses do not reach Japan before the end of the fourth century AD, but the occurrence of cognates for the word for ‘horse’ in the Ryukyuan languages suggest that the horse was present before 2200 BP.

The chronicles of both the Kojiki and Nihon Shoki indeed mention that Silla and Paekche authorities presented the Japanese emperor with horses as a gift between the mid-fourth and mid-seventh centuries. However, in Japan there is archaeological evidence for early horse sacrifice before horses became a valued military possession through contacts with the Korean three Kingdoms (Barnes 1993: 231). In the Nihon Shoki it is also stated that horse sacrifice became prohibited. Given the early contacts in the first and second centuries AD between chieftains of various Wo tribes from Japan with Chinese authorities at the commandery of Lelang, established in northern Korea in 108 BC by the Han dynasty (206 BC-220 AD), the historical context leaves room for the horse being imported in Japan geographically, through the Korean Peninsula but linguistically, through contact with speakers of Old Chinese. During the period of Han economic expansion, many Chinese artifacts flowed into the surrounding area’s, particularly bronze mirrors, iron, lacquerware, silks, wine and salt (Barnes 1993: 198, 202.)

**(2) MARE**

Proto-Indo-European **gʷow*- 'cow' > Tokharian A *ko*, B *keŭ* `cow' >> proto-Mongolic **ge(g)ü* ‘female livestock, mare’ >> pTg **gegu* ‘mare’

Proto-Mongolic **geü-n* (~ **gewü-n* ~ **gexü-n* ~ **gehu-n*~ **gegü-n*) ‘female livestock, mare’

MMo. *ge'un*, *guun-du*, *keun*, *ge’ün*, *gü’ün*, WMo *gegü(n),* *gegüü*, *geü* ‘mare’, Khalkha *gü:(n)* 'mare', Kalmuck *gü:n* 'mare', Ordos *gǖ*, Dagur *geu* 'mare', Eastern Yoghur *gǖn*, Monguor *gu*: 'female of an animal kept for use, such as horse, mule, donkey or dog’ (Mostaert 1933: 138).

Proto-Tungusic **gegu* ‘mare’

Jurchen *ge*, Manchu *geo, geo murin*, Sibe *geu*, *geo*, Evenki *ge:Ɣ*, Solon *ge:Ɣ* ‘mare’

There is some disagreement in Mongolic linguistic literature about the origin of the intervocalic velar in WMo. *gegü(n).* Whereas Poppe (1966: 190), derives it from an original intervocalic velar **g*, Janhunen (1999) proposes a laryngeal origin **x*, Svantesson et al. (2005) propose **h*, Starostin et al. (2003) propose a labial glide **w* and Nugteren (2011: 342) reconstructs a -*Ø*- here. An ancestral form **geü-n* or **gewü-n* seems to be the most parsimoneous fit for the borrowing chain, proposing Tokharian B *keŭ* `cow' as a model. If the Monguor meaning ‘female of an animal kept for use such as horse, mule, donkey or dog’ (e.g., *gu: mori* ‘mare’, *gu: noxue:* ‘bitch’; Mostaert 1933: 138, Li 1988) indeed represents a preservation of the original semantics, the intermediate meaning ‘female livestock/ cattle’ explains the borrowing of ‘cow’ as ‘mare’. This observation is supported by the Manchu borrowing *geo murin*, which is a combinational copy with *morin* ‘horse’, suggesting that the meaning of the word in isolation in the model language was ‘female animal’ rather than ‘mare’.

The Tungusic form is a borrowing from Mongolic as it imitates the presumably secondary **g*, which developed shortly after the Middle Mongolian stage. The relative late borrowing into Proto-Tungusic is supported by the distribution and shape of the Tungusic forms. Although the regular reflex of Proto-Tungusic *-*g*- is *-*g*- in Manchu and Jurchen, it is missing here. This observation suggests that the ancestral Manchuric form **geu* has been borrowed from Proto-Mongolic **geü* at an earlier time than Mongolic **gegü* was transferred into the Northern Tungusic languages. Therefore, the borrowing of the word for ‘mare’ into the Manchuric branch must have taken place after 1950 BP.

Rona-Tas (1988: 393-394) refers to contemporary Chinese *kè*騍 ‘female horse/mule, mother horse/mule’ as a potential member of this borrowing chain. As our word goes back to Middle Chinese MC *kwa* ‘female horse, mule’ and is attested on bamboo slips, around 475-221BC, we tentatively reconstruct Old Chinese **[k]ˤo[j]-s* ‘female horse, mule’. Reminiscent of the word for ‘horse’ in the previous etymology, this word may have been borrowed from Proto-Tibeto-Burman **kre[j]* ‘mule’, reflected in Tibetan *dre(l)* ‘mule’ and Dapka *kreʔ* ‘mule’. However, these ancestral forms are difficult to reconcile with the proposed borrowing chain.

**(3) ASS**

Proto-Mongolic **kula*- ‘to have the ears laid back’ > **kulan* ‘ass’ >> Proto-Turkic **kulun* ‘foal’

Proto-Turkic **kulun* ‘foal’

OT *qulun* ‘foal’, MTk. (Kypchak) *qulun* ‘foal’, Az. *Gulun* 'foal (between 3 and 4 months old)', Bashk. *qolon* 'foal', Chu. *xъ^w^m* 'foetus (foal)', Crim Tat. *qulun*, *quluntay* 'foal', Kaz. *qulїn* ‘foal’, Khak. *xulun* ‘suckling foal’, Kirgh. *qulun* ‘suckling foal’, KKalp. *qulїn* ‘suckling foal’, Nogh. *qulїn* ‘foal’, Oyr. *qulun* ‘suckling foal’, SUig. *qulum*, *qulun* ‘foal’, Tat. *qolïn* ‘foal (younger than one-year old)’, Tk. *kulun* ‘suckling foal’, Tkm. *Gulun* ‘(suckling) foal’,

Tuv. *qulun* ‘suckling foal’, Uig. *qulun* ‘suckling foal’, Uzb. *qulun* ‘foal (younger than one-year old)’, Yak. *kulun* ‘foal’

Proto-Mongolic **kulan* ‘ass’

MMo. *qulan* (SH), *qulan* (MA); WMo. *qulan*, *kulen* ‘wild ass of the Asiatic steppe, kulan, Equus hemionus’, Khal. *xulan*, Bur. *xulan*, Kalm. *xulŋ*, *xulṇ*, Ord. *xulan*.

The Mongolic term for ‘ass’ can be internally derived as a deverbal noun in *-*n* of a base verb **kula*- ‘to have the ears laid back’. The suffix derives other nouns from verbs such as MMo. *ayu*- ‘be afraid’ -> *ayun* ‘fear’ and *hice*- ‘be ashamed’ -> *hicen* ‘shame’ (Robbeets 2015: 392). The verb **kula*- underlies in several deverbal derivations such as Written Mongolian *qulayi*- ‘to have cropped ears (intr.)’, *qulmayi*- ‘to have the ears laid back, to be crop-eared’ and *quluƔur* ‘laid or pressed back of ears, crop-eared’. Since the Mongolic word is morphologically complex, while the Turkic word does not reflect this segmentation, the direction of the borrowing can be identified as going from Mongolic into Turkic. As the word is also attested in Chuvash, the borrowing must have taken place before the break-up of Proto-Turkic around 2200 BP. More recently, the Mongolic word has been independently borrowed into Manchu *ku:lan* (Rozycki 1994: 148), but it is not attested in other Tungusic languages.

- 1. **Ancient borrowings from a Proto-Tungusic model**

In contrast to the Eurasian borrowing chains discussed above, spreading from a Turkic or Mongolic model into other Transeurasian languages during the Late Bronze Age and Early Iron Age, the borrowings from a Tungusic model into surrounding Transeurasian branches such as Mongolic, Koreanic and Japanic seem to be of a more recent age. They have taken place in a period from the Late Iron Age to the Middle Ages and may have had a subbranch of Tungusic as their starting point rather than Proto-Tungusic *per se*. They include words for agricultural imports such as ‘barley’ and ‘wheat’, farming tools such as ‘plough’ and iron utensils such as ‘sabre, sword’.

**(1) BARLEY, WHEAT**

Proto-Indo-European **mṛk* ‘seeds of barley, products derived from barley’ ? > Tocharian? >> Proto-Tibeto-Burman **mrə(k)* **‘**wheat, barley’ >> Old Chinese **來****m*ə*.rˤ*ə*k* > **mə.rˤə*  **‘**wheat, barley’ >> Proto-Tungusic **mirgi* ‘barley (*Hordeum vulgare*)’ >> Proto-Koreanic **milk* ‘wheat’ ~ Proto-Tungusic **murgi* ‘barley’ >> Proto-Japonic **munki* ‘wheat, barley’

Proto-Tibeto-Burman **mrə(k)* **‘**wheat, barley’

Hani (Caiyuan) *mɤ³⁵ tsɿ̄³¹* ‘wheat’, Hani (Mojiang) *mɛ³¹ tsɿ̄³¹* ‘straw (wheat)’, Jinuo *mə⁴² tsi³³* / *mɯ³¹ tsi⁴⁴ ‘*wheat, straw (wheat)’, rGyalrong (Jinchuan Guaninqiao Wobzi) *mjæ̌nfə̂n* ‘wheat flour’, Laze (Xiangjiao) *mie˧* ‘wheat bread’, Pumi (Jiulong) *m̥e⁵⁵* ‘awn of wheat’, Qiang (Mawo) *ʁlə* ‘wheat’, Qiang (Taoping) *ʁuə²⁴¹ ‘*wheat’, Xumi *mɛ̃⁵⁵* ‘wheat’, Limbu *ma:si* ‘wheat’, Bai (Jianchuan) *mə̱³³ ‘*wheat’. The meaning ‘barley’ is also found in Qiang *ʁə* ‘wheat, barley, etc.’ and rGBenzhen *kɐr* ‘wheat, barley’.

Proto-Tungusic **murgi* < **mirgi* ‘barley (*Hordeum vulgare*)’

Solon *mụrgil* ‘spring crops, spring-sown field, barley’, Jurchen *mirɣei* ‘product of agriculture’, Manchu *muji* ‘barley (*Hordeum vulgare*)’, Sibe *muji* ‘barley (*Hordeum vulgare*)’, Olcha *muji* ‘barley, oats’, Nanai *muji* ‘oats’, Ud. *muji* ~ *muju* 'barley', Oroch *muji* ‘barley’, *mudi* ‘Russian flour’

Proto-Koreanic **milk* ‘wheat’

MK *·milh* ‘wheat’, K *mil* ‘wheat’, JJ *mil* ~ *mel*, KB *mil*, KN *mil*, JN *mil*, JB *mil*, KW *mil*, CN *mil*, CB *mil*, KG *mil*

Proto- Japonic **munki* ‘wheat, barley’

J *mugi* ‘wheat, barley’, OJ *mugi_1_*‘wheat, barley’, Kagoshima *muʔ*, Koshiki (Kamikoshiki) *muːgi*, Miyazaki *mugi,* Kumamoto *mugi*, Ōita *mugi*, Fukuoka *mugi*, Saga *mugi*, Fukue *mugi ~ mun,* Nagasaki *mugi* ‘wheat, barley, rye, oat’, Yamatohama (Amami) *mugi*, Asama (Amami) *mugii*, Yoron (Amami) *mugi*, Yonamine *muzii* (Okinawa), Shuri (Okinawa) *muzi*, Old Shuri *muzi*, Hirara (Miyako) *mugï*, Nagahama *mugï* (Miyako), Ikema (Miyako) *mudzï*, Ōgami (Miyako) *mukï*, Tarama (Miyako) *mugï*, Ishigaki (Yaeyama) *muŋ*, Hatoma (Yaeyama) *muŋ*, Hateruma (Yaeyama) *muŋ,* Kohama (Yaeyama) *mui,* Taketomi (Yaeyama) *muŋ,* Yonaguni *muŋ,* PR **mugi* ‘barley’

Similar to barley, wheat was domesticated in the Fertile Crescent area in the Near east around 10 000 BP. Between 7000 and 4500 BP, wheat and barley cultivation expanded into eastern Central Asia. Wheat grains reported from the Altai Mountains are dated to 3000-1500 cal. BC (Liu et al. 2019). Between 4500 BP and 3500 BP the Fertile Crescent crops were introduced to eastern China. Early wheat finds dating back to 2500-2000 cal. BC have been reported from the Shandong Peninsula but it was not until after 4000 BP that wheat and barley were grown on a significant scale (Boivin et al. 2012: 457, Liu et al. 2018). Wheat cultivation moved to China along a series of mountain corridors to the north of the Tibetan Plateau. Barley, on the other hand, may have spread into China via Tibet (Liu et al. 2018). The dispersal model for ‘barley’ fits the proposed borrowing chain: originating in Indo-European, the word may have been transferred via Tocharian to Tibeto-Burman and to Old Chinese, from where it entered Proto-Tungusic and was further borrowed into Proto-Koreanic and Proto-Japonic.

Based on reflexes such as proto-Celtic **mraki-* ‘corn or seed of barley’ (e.g., in Old Irish *mraich* ‘malt’ or in Welsh *brag* ‘barley corns, malt’), Luvian *marwali-* ‘barley-stem’, Hittite *marnuwa(nt)-* ‘a kind of beer’ and Old Indic *markaṭaka-* ‘a kind of corn’, Blazek (2019) suggested to reconstruct Proto-Indo-European **mṛk* ‘seeds of barley, products derived from barley’, but it cannot be excluded that these words are ultimately borrowed from a Central Asian substrate word rather than being inherited from Proto-Indo-European. Although the cereal terminology of Iranian is relatively well known, there is no plausible cognate available. A cognate candidate also lacks from Tocharian, but the Tocharian lexicon is not abundantly attested in the agricultural sphere (Peyrot 2018) and the word may thus have been lost.

Therefore, it cannot be excluded that a reflex of **mṛk* ‘barley’ in Tocharian ultimately served as a model for Proto-Tibeto-Burman **mrə(k)* **‘**wheat, barley’. Similar to the borrowing chain for ‘horse’ in Section 3.2, the term for ‘wheat, barley’ may be a borrowing within the Sino-Tibetan family, from Proto-Tibeto-Burman into Sinitic. Old Chinese is the Chinese spoken from the beginning of written records around 3200 BP to 2300 BP, but the word for ‘barley, wheat’ probably arrived in Sinitic when the crop was introduced, that is between 4500 and 3500 BP. The late date of the adoption of barley and wheat in China contradicts the reconstruction of the root to Proto-Sino-Tibetan, as proposed by Schuessler (2007: 374) as well as its reconstruction to Proto-Transeurasian as proposed by Starostin (2008).

Archaeological evidence suggests that barley first arrived in the Southern Primorye through Chinese contact at the time of the Krounovska culture (2600–1800 BP), situated south of Lake Khanka (Wang & Robbeets 2020, Sergusheva & Vostretsov 2009: 214–215; Leipe et al. 2019). This observation yields a time frame for the borrowing of Old Chinese **mə.rˤə* ‘barley, wheat’ into Proto-Tungusic **mirgi*, the break-up of which we estimated at 1950 BP (ED Figure 1; SI 4). Only Jurchen, which was the official language of the Jin dynasty (1115-1234) of Northern China and Manchuria, reflects a form **mirgi*, while the other Tungusic languages reflect **murgi*. Both forms are probably related through labial attraction whereby the original high vowel *i* assimilated to the initial labial nasal *m*. The vocalism suggests that the word was transferred separately into Koreanic and Japonic.

It was Proto-Tungusic **mirgi* that served as the model for Proto-Koreanic **milk* ‘wheat’. Middle Korean has a final fricative in *·milh* ‘wheat’ but it is known that velar lenition (**Ck* > **Ch*) has taken place in **Ck* clusters at an early stage in Korean. Beckwith (2007) reconstructed Old Koguryo **miŋpar* ‘grain’ in which the second syllable was connected with **par* ‘second-growth paddy rice’. The first syllable **miŋ* may mean ‘kind of grain’ and represent a borrowing of the Tungusic form.

It is safe to assume that during the Late Chulmun (4000-3300 BP) and Mumun (3300 – 2000 BP) period, contacts took place between Tungusic and Koreanic populations since in that time megalith dolmen constructions were spread from Manchuria to Korea and a bronze culture resembling that of the Lower Xiajiadian culture (4200-3600 BP) diffused from Siberia (Nelson 1993: 159-163; Barnes 1993: 153, 165).

The more recent Tungusic form pTg **murgi* served as a model for pJ **munki* ‘wheat, barley’. It is generally agreed that voiced stops in Japanese derive from prenasalized voiceless stops in Old Japanese and ultimately from nasal clusters (Robbeets 2005: 55-56). Clusters including voiced obstruents such as –*rg*- in pTg **murgi* tend to be borrowed as a proto-Japanese nasal cluster *-*nk*-. The sound distribution of Ryukyuan cognates for the Japanese word indicates that borrowing may have occurred in proto-Japonic, that is before 2139 BP (ED Figure 1; SI 4). This indicates that Japonic and Tungusic speakers were in contact, probably at a time when Japonic was still spoken on the Korean Peninsula and had not yet spread to the Japanese Islands. Although the direction of the borrowing is opposite, the context of the borrowing reminds of the borrowing of *miso* ‘fermented bean paste’ explained in Section 3.4. below.

**(2) IRON**

Proto-Tungusic **sele* ‘iron’ ➝ **sele-me ‘*made of iron’ >> Proto-Mongolic **seleme* ‘sabre’

Proto-Tungusic **sele* ‘iron’

Evenki *sele*, Even *hel*, Negidal *sele*, Solon *sele,* Sibe *selǝ*, Manchu *sele*, Jurchen *sele*, Olcha *sele*, Orok *sele*, Nanai *sele*, Oroch *sele*, Udehe *sele* ‘iron’

Proto-Mongolic **seleme* ‘sword, sabre’

Written Mongolian *seleme*, *selme* ‘sword, sabre’, Khalkha *selm*, Burial *helme*, Kalmuck *selmǝ*, Ordos *seleme*, Dagur *selmi*:, *selemij*, Eastern Yoghur *selme*, Monguor *silǝm*

The Proto-Tungusic word **sele-me ‘*made of iron’ derives from the noun **sele* ‘iron’ and a denominal suffix *-*mA* deriving substances, which is well represented across the Tungusic languages (Benzing 1955: 1039). As the Mongolic languages lack such a denominal suffix, it is clear that the word is morphologically segmentable in Tungusic, but not in Mongolic. Therefore, the direction of the borrowing is from Tungusic into Mongolic. The borrowing has taken place before the break-up of Proto-Mongolic, dated at 939 BP (ED Figure 1; SI 4), but it may have occurred after the break-up of Proto-Tungusic in 1950 BP.

**(3) PLOUGH**

Proto-Tungusic **ana*- ‘to push apart’ → **anja* ‘plough’ >> proto-Mongolic **anja* ‘plough’

→ **anji* 'adze'

Proto-Tungusic **ana*- ‘to push apart’

Evenki *ana*- ‘to push, push apart’, Even *a:n*- ‘fell (trees), push, to set traps, dump wood’, an ‘trap’, Neg *ana*- ‘push’, Oroch *ana*- ‘push’, Ud. *ana*- ‘push’, Olcha *ana*- ‘push’

Orok *ana*- ‘push’, Na. *ana*- ‘push’, Ma. *ana*- ‘push, urge, prompt, extend (a deadline), to push wider (a battue, the beating of wood to flush game)’

Proto-Tungusic **anja* 'plough', *anji* 'adze'

Ma. *anja* 'plough', *anji*- 'to hack, chop with an adze', *anji* 'adze', Olcha *anja* 'plough', Na. *anja* 'plough', Solon *anjasu* ‘plough’

proto-Mongolic **anja-sun* ‘plough’ ~ **anji-sun* ‘plough’

MMo. *anjasun* ‘plough’, WMo. *anjisu(n)* ‘plough’, Khal. *anjis(an),* Bur. *anzaha(n),* Ordos *anjasu*, *anjus(u),* *andüs*, Kalm. *andsx*, *ancăsn* (*ancn*), Dag *anǰa:s*, Eastern Yughur *anǰagsən*, Huzu Mgr. *nʒ́asə*, Minhe Mgr. *(a)nƺasï*, Baoan *anʒ́isuŋ*, Kgj *anjasun*, Dgx *anjasuŋ* ‘plough’

Agricultural tools such as hand ploughs were discovered at Neolithic sites in the Southern Primorye starting from the Zaisanovka culture (5300-2500 BP) (Sergusheva 2009: 210) If the Tungusic words **anja* 'plough' and *anji* 'adze' are indeed derivations from a base verb **ana*- ‘to push apart’ and an instrumental suffix *-*ja* (cf. Ulcha, Na. *say-ja* ‘sieve’*)*, then the direction of the borrowing is from Tungusic into Mongolic. The palatal element in WMo. *anjisu(n)* ‘plough’ and Khal. *anjis(an)* may reflect the borrowing of **anji* from Proto-Tungusic *anji* 'adze', while the other forms are borrowed from pTg **anja* 'plough'. The collective suffix -*sun* was added in Mongolic. Mongolic forms with -*d*- are probably due to dissimilation with this suffix. The suffixed word was re-borrowed into Tungusic as Solon *anjasu* ‘plough’.

It is interesting to note that Middle Japanese has a compound *adi-suki* ‘a good plough’, in which the second element reflects *suki* ‘spade, plow, plough’. It cannot be excluded that the first element, which derives from pJ **anti* may be a borrowing from the Tungusic word as well. The borrowing has taken place before the break-up of Proto-Mongolic, dated at 939 BP (ED Figure 1; SI 4) and before Middle Japanese (1200-400 BP).

- 1. **Ancient borrowings from a Proto-Koreanic model**

Ancient borrowings from a Proto-Koreanic model are occasionally transmitted into Tungusic, mostly into Manchuric after the break-up of Proto-Tungusic, but more commonly the recipient language is Japonic or pre-Old Japanese. Basically, there are two types of ancient borrowings, depending on the time and space of the contact.

First, we find borrowings, such as the one for ‘bean paste’, dating back to the Bronze Age when ancestral forms of Japonic were still spoken on Peninsula. Here the early timing and location of the borrowing are supported by the distribution of cognates in the Ryukyuan languages and by the continued transmission of the borrowing from Japonic into Manchuric.

Second, there are more recent borrowings dating back to the Iron Age and Antiquity and transmitted into pre-Old Japanese when it was already spoken on the Japanese Islands. The second type includes cultural borrowings, such as the one for ‘silk’ taking place in the context of the Silkroad exchange of goods before the introduction of Buddhism around 550 AD as well as borrowings for items, such as ‘rice cake’, ‘measuring bowl’, ‘spade’ and ‘rake’ (see also Section 2.1.(9)) brought to the Nara area by Korean refugees, escaping the Silla unification (660-668) in the middle Asuka period (6^th^ C to 710). These words can be easily identified as borrowings in pre-Old Japanese as they mostly lack Ryukyuan cognates.

**(1) BEANPASTE**

Proto-Koreanic **micu* ‘fermented bean paste’ >> Proto-Japonic **misə* ‘fermented bean paste >> Proto-Manchuric **misu* ‘fermented bean paste’

Proto-Koreanic **micu ~ mico ~ micʌ* ‘fermented bean paste’

EMK *micwu* [*mitsu*] ‘fermented bean paste’, MK *meycwu* ~ *mye·cwu* ~ *myeycwo* ~ *myecwo* ~ *meyco* ‘soybean malt’, K *meycwu* ’soybean malt; malt made of soybean, salt and water’, JJ *meycwi*, KB *meycwuk*, KN *mici*, JB *meycwu*, JN *micwu* ~ *meycwu*, CB *meyca*, CN *meycwu*, KG *meycwu*, KW *micwu*

Proto-Japonic **miso* ~ **misə* ‘fermented bean paste’

J *miso*, OJ *miso* ‘fermented bean paste; a condiment used as a soup base and as a seasoning for various dishes. The semi-solid sediment that remains after the soy sauce is removed from the surface when fermenting malted and boiled soybean paste and placing it in in highly salted water’, Miyazaki, Kagoshima/ Koshiki/ Kumamoto*/*Ōita*/*Fukuoka */*Saga*/*Fukue */*Nagasaki *miso* ‘fermented bean paste’, Yamatohama (Amami) *misu*, Asama (Amami) *misjuu*,Yoron (Amami) *misju*, Yonamine (Okinawa) *misuu*, Shuri (Okinawa) *Nsu* / *misu*, Old Shuri *Nsu*, Hirara (Miyako) *msu*, Nagahama (Miyako) *msu*, Ikema (Miyako) *ŋsu*, Tarama (Miyako) *mʃu*, Ishigaki (Yaeyama) *miʃu* , Hatoma (Yaeyama) *misu*, Hateruma (Yaeyama) *miʃu,* Kohama (Yaeyama) *miʃu,* Yonaguni *nsuo*, PR **misu* ‘fermented bean paste’

Proto-Manchuric **misu* ‘fermented bean paste’

Manchu *misu*, *misun* ‘fermented bean paste; a thick, salty, reddish vinegar made by fermenting pea- or bean-meal paste with salt, and sometimes with sharped spices such as peppers, used in flavoring foods, soya and pickles’ (Zakharov 1875), Jurchen *misu*, *is(g)un* ‘bean paste’, Sibe *misun* ‘fermented bean paste’

Miso, which is still popular as a condiment in East Asia has a long history in the Transeurasian region (Rozycki 2001). The borrowing chain indicates that the technique of fermenting bean paste was developed on the Korean Peninsula by Koreanic speakers who transferred it to Manchuric speakers in Manchuria through intermediary of Japonic speakers.

The original model word can be traced to Proto-Koreanic **micu.* The word is first attested in the Kyerim Yusa, phonetically represented as [*mitsu*]. The high front vowel underwent so-called “i-breaking” in the history of Korean, whereby the /i/ assimilated properties of the vowel of the second syllable. The phoneme /c/ was still pronounced as a dental affricate [ts] in Early Middle Korean. The closest imitation within the limits of the Proto-Japonic sound inventory, which lacked a phoneme /c/, was to imitate the dental affricate with the phoneme /s/. The presence of /s/ in the Manchuric words suggests that the model was Japonic rather than Koreanic because the Manchuric sound inventory has a phoneme /c/ to imitate the Korean dental affricate.

The evidence for the Old Japanese word for ‘bean paste’, written in logographic Chinese characters (not in phonographic *man’yōgana*) goes back to a wooden tablet (*mokkan)* dug up from the old Nara capital of Heijo (Bentley, pc). This fragment preserves the following inscription: 御末醤一石二斗, i.e. HON-“bean paste” reads as *miso*. The Wamyoshō, a Chinese to Middle Japanese dictionary compiled in the mid Heian period has the same *kanji* (末醤) glossed as *miso* (美蘇).

As we do not know the quality of the final vowel in Old Japanese, the word can go back to both **miso* or **misə*, a mid back vowel would be a plausible imitation of Proto-Koreanic **mico,* while a mid-central vowel would be a more plausible imitation of the alternant Proto-Koreanic **micʌ.*

The word was transferred into Japonic before its primary breakup, i.e. before 2139 BP (ED Figure 1; SI 4). Reminiscent of the Wanderwort ‘wheat, barley’ in Section 3.3. above, this implies that the borrowing from Koreanic may have taken place when the Japonic speakers were still present on the Korean Peninsula, before their spread to the Japanese Islands.

The ancestral Manchuric language borrowed proto-Japonic **miso* ~ **misə* as **misu(n)* with a final high back vowel and an occasional word-final nasal. This is probably due to morphological analogy with a large group of food terms on final -*sun* in Manchuric, resulting from the borrowing of Mongolic words for food items with the collective suffix -*sUn*, e.g. Ma. *fursun* 'shoots, sprouts', Ma. *alisun* ‘grain that has sprouted from lost or abandoned seeds', Ma. *amsun* ‘offerings of wine and food to a deity’, Ma. *ekšun* ‘the dregs of yellow rice wine’, etc.

The borrowing of the Japonic word ‘beanpaste’ into the Manchuric branch of Tungusic must have taken place between the primary break-up of Proto-Tungusic and the break-up of Manchuric, notably between 1950 and 900 BP (ED Figure 1; SI 4). In this period, some Japonic speakers had already left to the Japanese Islands, while other pockets of Japonic speakers remained on the Korean Peninsula until the linguistic diversity of the Peninsula became completely erased through the Silla unification (660-668 AD).

**(2) SILK**

Old Chinese **kʷen-s* ‘a kind of silk material’ >> Pre-Middle-Korean **kyǝn* ‘silk’ >> Pre-Old Japanese **kinu* ‘silk’

Old Chinese **kʷen-s* ‘a kind of silk material’

This term was borrowed in just a few Tibeto-Burman languages, belonging to different subgroups, such as Tangut (Qiangic) *ŋwər* ‘coloured silk’, Qiang (Mawo/ Qiangic) *ʁuɛʴ* ‘fabric (silk)’, Tibetan (Tibetic) *khruɦu tse* ‘fabric (silk)’ and Meithei (Kuki-Chin) *kəbrəŋ*‘silk’

Pre-Middle Korean **kyǝn* ‘silk’

MK *kyen-pho* ‘silk, linen and cotton’, K *kyen* ‘silk’ (Sino-Korean)

pre-Old Japanese/ Proto-Japonic **kinu* ‘silk’

OJ *ki_1_nu* ‘silk; garment’, J *kinu* ‘silk’ , Nagahama *kinu*, Hirara *kɨN*, Tarama *kɨN*, Ishigaki *kɨŋ*

Silk was a precious good, widely used for gifts, tribute, and exchange, leading to the development of Silk roads during the early Han period (206 BC-220 AD). Silk production is thought to have been introduced from China, probably through Korean intermediary, together with sericulture in the Middle Yayoi period (Barnes 1993: 173; Omura and Kizawa 2017; see SI 7). As such the use of silk for weaving postdates the use of bast fibers, which goes back to Early Yayoi.

Old Chinese **kʷen-s* ‘a kind of silk material’ is the model for Sino-Korean *kyen* ‘silk’. It is glossed as ‘a kind of silk material’ because it is a type of silk product in contrast to the Old Chinese word 絲 **[s]ə* ‘silk’, which represents the original material. The loss of *k*- was relatively late in the history of Chinese: It is preserved in Middle Chinese, and Early Mandarin as spoken during the Yuan dynasty (1279-1368). This observation situates the borrowing of the word into Koreanic in the first millennium or earlier. The labial glide in the reconstruction may be a mere prosodic factor, which was therefore not copied into Proto-Koreanic.

The Proto-Koreanic word was probably transferred to pre-Old Japanese before 550 AD. The relatively early dating of the borrowing is supported by a phonological phenomenon, known as Pre-Old Japanese mid vowel raising, i.e. the raising of the vowel **e* to **i*. As mid vowel raising was not applied to borrowings related to Buddhism such as OJ *tera* ‘Buddhist temple’ and OJ *potoke* ‘Buddha’, we can deduce that this phenomenon took place before the introduction of Buddhism into Japan in the first half of the 6^th^ century AD (Frellesvig & Whitman 2008: 38). Since the pre-Old Japanese word for ‘silk’ was borrowed from a model yielding the closest imitation **kenu* with **e*, but raised its original vowel to **i*, the borrowing must thus have taken place before the application of mid vowel raising, *i.e.* before 550 AD. The vocalism of the Nagahama word *kinu* suggests that it was a borrowing from Mainland Japanese. However a few other Ryukyuan languages, such as Hirara, Tarama and Ishigaki seem to preserve a cognate for this word. If this is indeed the case the word can be reconstructed back to Proto-Japonic, i.e., to before 2200 BP.

**(3) RICE CAKE**

Proto-Koreanic **siteki* ‘steam cake made of cereals or rice’ >> pre-Middle Japanese *sitogi* ‘rice cake for ceremonial purposes’

Proto-Koreanic **siteki* ‘steam cake made of cereals or rice’

MK ·*stek* ‘steamed cake made of rice or any cereal, sometimes pounded or shaped’, K *ttek* ‘rice cake’, JJ *ttek*, KB *ttek*, KN *ttek*, JN *tteyk* ~ *ttek* ~ *meyk*, JB *ttek*, KW *siteki*, CN *kelmok*, *hinttek*, CB *ttek*, KG *ttek*, CN *ttayk*, PB *sitekwu* ~ *sitekwi*, HN *sitek* ‘rice cake’

EMJ *sitogi* ‘rice cake for ceremonial purposes’

The Japanese word for ‘rice cake’ is first attested in the Myōgi-shō in 1081. It has no cognates in the Ryukyuan languages. Therefore, it is likely to be a borrowing from Proto-Koreanic **siteki* ‘steam cake made of cereals or rice’, dating back to the first millennium. The three-syllabic shape including high front vowels reconstructed for the Koreanic model is supported by North Korean dialects such as P’yŏnganbukdo and Hamgyŏngnamdo and by the Kangwǒndo dialect in South Korea.

The most probable time and place of borrowing was the Asakusa period (600-710 AD) in the area of Nara in Japan. This is the period of the Silla unification (660-668) on the Korean Peninsula, which caused Paekche aristocrats to flee to Japan and led to the transfer of cultural borrowings from Paekche Old Korean into pre-Old Japanese. (Francis-Ratte & Unger 2020, Martin 1996: 45, Miller 1996: 185)

**(4) MEASURING BOWL**

Proto-Koreanic **mal* ‘measuring bowl’ >> pre-Old Japanese *mari* ‘a vessel in the shape of a bowl for serving sake or water’

Proto-Koreanic **mal* ‘measuring bowl’

MK ·*mal* ‘bowl for measuring grains, liquid, or flour; a unit of measure’, K *mal* ‘a measure containing about 18 liters; a unit of measure’, JJ *mal*, KB *mal*, KN *mal*, JN *mal*, JB *mal*, KW *mal*, CN *mal*, CB *mal*, KG *mal* ‘a unit of measure’

OJ *mari* ‘a vessel in the shape of a bowl for serving sake, water, rice, etc.’

As the word *mari* is already attested in Old Japanese, its borrowing must have preceded that period. As in the previous example, the most probable context of borrowing was the cultural contact with Paekche aristocrats in Asakusa period (600-710 AD).

- 1. **Ancient borrowings from a Proto-Japonic model**

In contrast to most of the ancient borrowings from Koreanic to Japonic, those that go the other way around, from Japonic to Koreanic are all well distributed in the Ryukyuan languages. This indicates that the time of the borrowing predated the break-up of Proto-Japonic (2136 BP) and thus took place at an earlier time than most of the Koreanic borrowings discussed in the previous Section.

As the context of borrowings such as ‘early ripening grain/rice’ and ‘rice plant’ seems to be related to the adoption of rice agriculture, it is inviting to situate the contact on the Korean Peninsula in the Bronze Age, after 3500 BP when rice agriculture was introduced from the Shandong-Liaodong interaction sphere (SI 7). The borrowing for ‘(metal) cooking pot’ may also go back to this Bronze Age setting.

**(1) EARLY RIPENING GRAIN/RICE**

Proto-Japonic **wasa-ra* ‘early ripening grain, rice’ >> Proto-Koreanic **pʌsal* ‘hulled variety of grain, rice’

Proto-Japonic **wasa-ra* ~**wǝsǝ-rǝ* ‘early ripening grain, early ripening rice’

J *wase* (2.4?), OJ *wase* ‘an early ripening variety; ripening early, precocious; early-ripening variety of rice plant,’ J *wasa-mono* ‘early produce,’ OJ *wasa-ipi_1_* ‘rice cooked from early rice,’ *wasa-po_1_* ‘early ears of grain/rice,’ *wasa-ki_2_* ‘rice wine made from early rice’; OJ *woso_2_* ‘precocious, early ripening,’ OJ *woso_2_ ro_2_* ‘precocious, early ripening’

Proto-Koreanic **pʌsal* ‘hulled variety of grain, rice’

K *ssal* ‘uncooked rice, any hulled variety of grain,’ e.g., K *ssal poli* ‘hulled barley’; *poli ssal* ‘a grain of barley, a barley corn’ (with *poli* ‘barley’), *copssal* ‘millet grain’ (with *co* ‘millet’), MK ·*psol* ‘uncooked rice, hulled variety of grain,’ LOK 菩薩 **pʌsal* ‘rice’ (LMC 菩薩 *pɦuǝ̆ sar*), KN *mayssal* ~ *maypssal* ~ *meyssal* ~ *meysssal* ~ *missal*, KW *meypssal* ~ *ipssal* ~ *chapssal*, KS *meypssal* ~ *sal* ~ *chapsal*, JL *maypssal* ~ *ipssal* ~ *meypssal* ~ *mopssal*, CC *meypssal* ~ *meysssal* ~ *ipssal* ~ *chapssal*, KB *mipsal* ~ *mipssal* ~ *misssal* ~ *ipsal* ~ *ipssal*, JJ *sal*, PA *ipsal*, HG *ipsal*, HH *ipsal*, JB *chapsal* ~ *chapssal*, CN *chapsal*, KS *chapssal* ‘uncooked rice, any hulled variety of grain’.

Vovin (2015) reconstructed proto-Japonic **wasay* ‘early rice’ and proposes that the proto-Japonic form was borrowed into proto-Koreanic **pasar* ‘rice.’ However, he recognized that this reconstruction is problematic: “[i]f PK **pasar* is a loan from PJ **wasay*, one has to explain how proto-Koreanic has got its final *-*r* from PJ *-*y*” (Vovin 2015: 234). This issue can be solved by taking into consideration the vowel alternation between OJ *wasa*- and *woso_2_* ‘precocious, early ripening’ in addition to the fact that *woso_2_* ‘precocious, early ripening’ is attested next to OJ *woso_2_ -ro_2_* ‘precocious, early ripening.’ The alternation with PJ **wǝsǝ-rǝ* supports the reconstruction of PJ **wasa-ra* ‘early ripening crop’ with a final liquid syllable.

The Proto-Japonic suffix **-ra ~ -rǝ* derived property nouns from verbal adjectives, e.g. in OJ *aka*- ‘to be red’ → *akara* ‘red’ (*akara tatibana* ‘red mandarin-oranges (MYS XVIII: 4060)), *usu*- ‘to be fine’ → *usura* ‘fine’, *uma*- ‘to be tasty’ → *umara* ‘tasty’, *yo_2_*- ‘to be good’ → *yo_2_ra* ‘good’, *sakasi*- ‘to be wise’ → *sakasira* ‘wisdom’, *kanasi*- ‘to be sad’ → *kanasira* ‘sadness’, EMJ *be*- necessitive -> *bera nar*- necessitive, etc. (Labrune 1998; Antonov 2007: 102, 111, 128-132, 153, 160, 196; Robbeets 2015: 339–346). The vowel alternation between pJ **wasa-ra* and **wǝsǝ-rǝ* may be due to labial assimilation of the initial vowel, followed by a restriction on the shape of Old Japanese root morphemes whereby the vowel *o_2_* cannot occur in a root together with the vowels *u*, *o_1_* or *a*, a phenomenon known as Arisaka’s law. There are no cognates for this word in the Ryukyuan languages, but the derivation of Ishigaki *payano:rɨ* ‘early rice’ as ‘maturing quickly’ supports the proposed derivation.

The reconstruction of Proto-Japonic **wasara* ‘early ripening grain, rice’ strengthens Vovin’s (2015) suggestion that the form was borrowed into Proto-Koreanic as **pʌsal* ‘rice.’ Even if the absence of cognates in the Ryukyuan languages seems to suggest a reverse direction, the direction of the borrowing from Japonic into Koreanic is supported by the morphological segmentability of the Japonic form in addition to the observation that pJ **w*- was borrowed as pK **p*-, given that Japonic had both **w*- and **p*-, while Koreanic had only **p*-. Given the fact that rice agriculture reached the Korean peninsula after 3500 BP, presumably through speakers of ancestral Japonic languages, migrating from the Shandong-Liaodong interaction sphere, the context of this borrowing can probably be situated in the Bronze Age on the Korean Peninsula (see SI 7).

Similar to the Japonic model, the Koreanic word can be used in reference to any grain. The attestation of the Korean form as LOK 菩薩 leads to the phonological reconstruction **pʌsal* ‘any hulled grain, rice’ in line with the Late Middle Chinese phonographic reading of these characters as 菩薩 *pɦuǝ̆ sar*. Since the logographic reading of these characters in Late Middle Chinese is ‘Boddhisatva’, there is a folk etymology suggesting that the Korean meaning ‘rice’ developed from the meaning ‘Boddhisatva’ after the introduction of Buddhism in 500 AD, similar to how *bosatsu* ‘Boddhisatva’ is used as a synonym for ‘rice’ in the Totomi dialect of Japanese (Ogura 1943). This explanation is doubtful because the original meaning in Koreanic is ‘hulled variety of any grain’, while ‘rice’ is a secondary development, which makes the semantic association more far-fetched.

The Proto-Koreanic **may* > *mey > mi > i* ‘rice’, reflected in the Korean dialectal forms, is probably an early loan from OC 米 **(C.)mˤ[e]jʔ* ‘millet or rice grains, dehusked and polished’ (Baxter & Sagart 2014). The Chinese loan morpheme is also found in Sino-Japanese *mai* and entered Proto-Ryukyuan as **maï* ‘rice’, reflected in Yamatohama (Amami) *misi* ‘cooked rice’, Asama (Amami) *miisɨ*, Yoron (Amami) *mai* ‘cooked rice, rice plant’, Yonamine (Okinawa) *mee* ‘cooked rice, rice plant’, Shuri *me:* ‘cooked rice’, Hirara (Miyako) *maï*, Nagahama (Miyako) *maï*, Ikema (Miyako) *maï*, Ōgami (Miyako) *maï*, Tarama (Miyako) *maï*, Ishigaki (Yaeyama) *mai*, Hatoma (Yaeyama) *mai*, Hateruma (Yaeyama) *më*: < *mayï,* Kohama (Yaeyama) *mai,* Taketomi (Yaeyama) *mai,* Yonaguni *mai* ‘rice’.

**(2) RICE PLANT**

Proto-Japonic **ipi* ‘steamed rice, cooked millet’ >> proto-Koreanic **ipi* > **pi* > **pye* ‘(unhusked) rice’

Proto-Japonic **ip-i* (eat-NMLZ) ‘steamed rice, cooked millet’

J *ii* ‘cooked rice’, OJ *ipi_1_* ‘cooked millet, steamed rice’, MJ *if*- ‘to eat’, Yamatohama (Amami), Asama (Amami),Yoron (Amami), Yonamine (Okinawa), Shuri (Okinawa), Old Shuri, Hirara (Miyako) *ï:*, Nagahama (Miyako) *ï:*, Tarama (Miyako) *ï:*, Ishigaki (Yaeyama) , Hatoma (Yaeyama) *i:*, Hateruma (Yaeyama) *ï:,* Kohama (Yaeyama) *i:,* Taketomi (Yaeyama) *i:,* Yonaguni *i:*, PR **i:* ‘cooked rice’

Proto- Koreanic **ipi* > **pi* > **pye* ‘(unhusked) rice’

K *pey* ~ K *pye* ‘rice plant, unhusked kernel of rice’, MK ·*pye* ‘rice plant, unhusked kernel of rice; annual plant. Its stem is 1.-1.5 meter tall and empty inside. Its long, crisscrossing leaves have parallel veins. Its flowers bloom during early autumn as pinacles at the end of the stems, each flower having one pistil, three stamens, no petals, inner cover around the pistal and stamens, and a glume within the covers. Some flowers have thorns. This term can also refer to the kernel of the rice plant. ·*pye* refers to the aristae that ripens during the autumn, and when pounded, it is called *ssal*, which is a staple food that can be steamed, boiled into porridge, or made into cakes, biscuits, or alcohol.’, KW *pey*, KG *pey*, KB *pey*, JL *pey*, CC *pey*, PA *pey*, HG *pey*, HH *pey*, HH *poy*, CN *wukkay ~ pey* ~ *peys* ~ *pye* ~ *poy*, KN *wukki ~ wukhi*, KS *wukhey*, CB *pey* ~ *pye* ~ *wukhey*, JN *pye*, JB *pey* ‘rice plant, unhusked kernel of rice’

Proto-Japonic **ip-i* ‘steamed rice, cooked millet’ is probably derived as a deverbal noun in PJ *-*i* from a verb ancestral to Middle Japanese *if*- ‘to eat’ (Vovin 1998: 371–372). This verb is not attested in Old Japanese, but absence of evidence is not necessarily evidence of absence.

Given the potential morphological complexity of the Japonic form and the rich distribution of the form in the Ryukyuan languages, the direction of the borrowing was probably from Japonic into Koreanic. Therefore, the borrowing goes back to a time before the break-up of Proto-Japonic and, also considering the context related to rice agriculture, probably before its arrival on the Japanese Islands.

We can reconstruct PK **ipi* on the basis of initial vowel loss and *i-*breaking, a common phenomenon, the result of which can be observed in e.g., M *khi*- ~ *khye*- ~ *hhye* ‘to kindle’ and MK *ni*- ‘go’ ~ *nye*- ‘go around.’ Here the proposed phonological development is supported by the final high front vowel in KN *wukki ~ wukhi* ‘rice plant, unhusked kernel of rice’.

**(3) VEGETABLE JAR**

Proto-Japonic **na-n-pe* (vegetable-GEN-jar) ‘pan, pot, kettle’ >> proto-Koreanic **nanpi* ‘pan, pot, kettle’

Proto-Japonic **na-n-pe* (vegetable-GEN-pot) ‘pan, pot, kettle’

J *nabe* ‘pan, pot, kettle’, OJ *nabe_2_* ~ *mi-nape_2_* ‘pan’ (< OJ *na* ‘greens, side-dish’ and OJ *pe_2_* ‘pot, jar’), Yamatohama (Amami) *nabï*- / -*nabï* ‘pan (in compounds)’, Asama (Amami) *naabɨ* ‘pan’,Yoron (Amami) *nabi* ‘pot’, Yonamine (Okinawa) *nabi* ‘pot’, Shuri (Okinawa) *naabi* ‘pot’, Hirara *nabi*, Nagahama *nabi*, Tarama *nabi*, Ishigaki *nabi*, Yonaguni *nabi* ‘pot’

proto-Koreanic **nanpi* ‘pan, pot, kettle’

K *nanpi* ‘pan, pot, kettle’, JJ *naympi*, KN *naympi*, JB *nampi*, JN *naympi* ~ *noympi*, CB *naympi*, CN *naympi*, KG *naympi*, KW *naympi*

The Japanese word *nabe* ‘pan, pot, kettle’ can be derived from a word for ‘greens, vegetables’ followed by a nasal genitive and a word for ‘pot’. The segment **pe* ‘pot’ also underlies in *turube* ‘well bucket’, *kanae* ‘a tripod kettle’, OJ *itupe_2_* ‘jar for sacrificial wine’, etc.

Due to the morphological segmentation in Japonic, the direction of the borrowing can be determined as being from Japonic to Koreanic. The nasal cluster in Korean illustrates that the word was borrowed from a Japonic model with a prenasalized pronunciation of the labial stop. This was the case in Old Japanese and before. However, the word is not attested in Middle Korean. Besides, foreign written sources, such as the *Nihon Kan-yakugo*, a 15th century Chinese language guide, the Japanese-Portuguese dictionary of 1604 and the *Wago-ruikai*, an 18th-century Korean glossary of Japanese, suggest that the voiced obstruent series in Late Middle Japanese and Modern Japanese was still prenasalized to a certain extent. Therefore, we cannot exclude that this word concerns a recent borrowing dating back to the Middle Ages. The attestation of the word in Cheju, however, suggests that the borrowing took place before the 13^th^ Century (SI 24).

**(4) METAL COOKING POT**

Old Chinese 坩 **[k]ˤ[a]m* ‘pot’ >> Proto-Japonic **kama* ‘(metal) cooking pot, kettle’ >> Proto-Koreanic **kama* ‘(metal) cooking pot, kettle’

Proto-Japonic **kama* ‘(metal) cooking pot, kettle’

J *kama* ‘iron pot, kettle’, MJ *kama* ‘cooking pot’, J *kame* ‘jar’, OJ *kame*_2_ ‘jar’, J *kamado* ‘kitchen range, furnace, oven’, OJ *kamado_1_* ‘cooking place, hearth’, Old Shuri *ka:mi*, Hirara (Miyako) *kami*, Nagahama (Miyako) *kami*, Ikema (Miyako) *kami*, Ōgami (Miyako) *kami*, Tarama (Miyako), Ishigaki (Yaeyama) *kami*, Hateruma (Yaeyama) *kami,* Kohama (Yaeyama) *kami,* Yonaguni *kami* ‘pot’

Proto-Koreanic **kama* ‘(metal) cooking pot, kettle’

MK *ka·ma* ‘cauldron; large cast iron pot; big, deep kettle for cooking or boiling over an open fire’, K *kama* ‘iron pot, cauldron, kettle; kiln, furnace, oven’, KW *kamasos* ~ *kamay* ~ *kamaysos* ~ *sos* ~ *khunsos ~ cwi-kama* ~ *cwi-kamay*, KS *kamasos* ~ *kamay* ~ *kamaysos*, JL *kamasos* ~ *kamaysos*, CC *kamasos* ~ *kamaysos*, CN *cwi-kama* ~ *cwi-kamay ~ kamassos* ~ *kamay* ~ *kamayssos* ~ *kaymaysos* ~ *khunsos*, JJ *kamay* ~ *kamey ~ sang* *kamey* ~ *sangkam*, CB *kamay* ~ *sos ~ kama* ~ *melikama*, KG *kamaysos ~ kama-ses* ~ *kama-sos*, PA *kamaysos*, HG *kamaysos*, HH *kamaysos*, KB *kamey* ~ *cilkama* , KN *kama ~ kami* ~ *kaamay*, KS *sos*, JN *kaymay*, *kaymas*, *kayma*, *kamma*, *kamays*, JB *kamay*, *komay* ‘iron pot, cauldron, kettle; kiln, furnace, oven’

Old Japanese *kame*_2_ ‘jar’ may be derived from PJ **kama* ‘pot, kettle’ and the element **pe* ‘pot’, discussed in (3) above. The semantic development from ‘pot’ to ‘oven’ is cross-linguistically attested, for instance, also in the comparison of English *oven* with Sanskrit *ukha* ‘pot’.

As this word is well distributed in the Ryukyuan languages, we reconstruct Proto-Japonic **kama* ‘pot, kettle’. If this word is indeed borrowed from Old Chinese **[k]ˤ[a]m* ‘pot’ (Miyake 1997: 198), the borrowing has taken place before the break-up of Proto-Japonic, which we estimate at 2136 BP (ED Figure 1; SI 4). A form ancestral to the Old Chinese form is a likely donorword for Proto-Japonic, since Japonic is expected to add a stem-final vowel due to its syllabicity constraints. The presence of the echo-vowel in the Proto-Koreanic form indicates that it is borrowed from Japonic rather than from Sinitic.

**4. Rejection of the Pastoralist Hypothesis**

Our research challenges the traditional ‘Pastoralist Hypothesis’ that identifies the primary dispersals of the Transeurasian languages with nomadic expansions starting in the eastern Steppe near the Altai mountains in the fourth millennium BP (Menges 1977, Miller 1990, Dybo 2013) by proposing a ‘Farming hypothesis’. In order to determine which hypothesis is most suited, we tested different predictions with regard to the homeland, age, inherited vocabulary and contact profile, summarized in Table SI 5.1 below.

Table SI 5.1 Testing the validity of the Pastoralist Hypothesis as compared to the Farming Hypothesis on the basis of spatiotemporal and comparative linguistic predictions.

|  | **Pastoralist Hypothesis**  **predicts** | **Farming Hypothesis**  **predicts** | **Our research**  **shows** |
| --- | --- | --- | --- |
| **Homeland** | Grasslands: Eastern steppe near the Altai mountains | Center of domestication: West Liao River/ Yellow River | West Liao River  (SI 4) |
| **Age ceiling** | 5 000 BP | 9 000 BP | 9181 BP  5595-12793 95% HPD  (SI 24) |
| **Inherited vocabulary** | Pastoral | Agricultural | Agricultural /No pastoral  (SI 5) |
| **Contact profile** | Agricultural terms are borrowed or inherited | Pastoral terms are borrowed | Pastoral terms are borrowed  Agricultural terms are borrowed or inherited  (SI 5) |

The Pastoralist Hypothesis predicts a homeland in the grasslands of the Eastern steppes near the Altai mountains, a location that originally lead to the coining of the label "Altaic" in reference to the Transeurasian languages. By contrast, the "Farming Hypothesis" predicts a homeland at a nuclear center where plant domestication emerged, such as the West Liao River or Yellow River areas in Northeast Asia.

According to Taylor et al. (2020), evidence for livestock-based herding subsistence on the Eastern Steppe is only found from the late 5th and early 4^th^ millennia BP onwards, while horse-based pastoralism is as late as 3200 BP. If Proto-Transeurasian indeed was the ancestral language of pastoralists on the steppe, 5000 BP is a ceiling for its existence. Since agriculture is thought to have emerged from around the 9^th^ millennium BP onwards in the domestication centres in Northeast Asia, 9000 BP serves as a ceiling for the Farming Hypothesis.

As far as inherited vocabulary is concerned, the ancestral language of pastoralists is expected to contain pastoral vocabulary relating, for instance, to domesticated ruminants, herding, dairying or horse exploitation. By contrast, ancient farmers are expected to be familiar with terms for agriculture, such as those relating to plant cultivation, agricultural tools or domesticated crops.

As agriculture preceded pastoralism in Northeast Asia, all pastoral terms are expected to represent later innovations through language-internal change or borrowing under the Farming Hypothesis, whereas a Pastoral Hypothesis leaves room for agricultural terms to be inherited or borrowed.

In reality, different homeland detection methods such as Bayesian phylogeography, diversity hotspot principle and cultural reconstruction converge on the location of the Transeurasian homeland in the West Liao River area, as proposed by the Farming Hypothesis. Even taking into account the large credible interval (5595 -12793 95%HPD), the dating of the first break-up in the Transeurasian family excludes the Pastoral Hypothesis since the interval predates the development of nomadic pastoralism on the Eastern Steppe.

Linguistic criteria applied above attribute all similarities between pastoral words across the Transeurasian languages to borrowing, while some of the similarities in the agricultural vocabulary are residues of inheritance. Therefore, it is safe to exclude the Pastoralist Hypothesis as an explanation of the linguistic homology.

**5. Sound correspondences underlying the above reconstructions**

**5.1 Reconstruction of the basic consonant inventory of Proto-Japonic**

| pJ | OJ | J | Amami | Okinawa | Miyako | Yaeyama | Yonaguni |
| --- | --- | --- | --- | --- | --- | --- | --- |
| *p | p | h-  -w- -ø- | ɸ- h- ç-  -ø- | p- ɸ-  -ø- | p- f-  -ø- | p-  -ø- | ɸ- h- tɕˀ- ç-  -ø- |
| *np | ^n^b | b | b | b | b | b | b |
| *t | t | t | t | t | t | t | t |
| *nt | ^n^d | d | d | d | d | d | d |
| *k | k | k | k- k^h^-  -k- -k^h^- | k-  -k- | k- f-  -k- -f- | k- ɸ- f- h-  -k- | k- k^h^- ø-  -g- |
| *nk | ^n^g | g | g | g | g | g ŋ ø | ŋ |
| *s | s | s | s | s | s | s | s c |
| *ns | ^n^z | z | ^d^z | z | z | z | d |
| *m | m | m | m | m | m | m | m |
| *n | n | n | n | n | n | n | n |
| *r | r | r | r | r | r | r | r |
| *w | w | w | w- b- y- g- ɸ-  -ø- | w- b- g- ʔ-  -ø- | b-  -ø- | b-  -ø- | b-  -ø- |
| *y | y | y | y | y | y | y | d-  -y- |

**5.2 Reconstruction of the basic vowel inventory of Proto-Japonic**

| pJ | OJ | J | Amami | Okinawa | Miyako | Yaeyama | Yonaguni |
| --- | --- | --- | --- | --- | --- | --- | --- |
| *a | a | a | a | a | a | a | a |
| *ə | o_(2)_ | o | u | u | u | u | u |
| *o | o_(1)_ | o | u | u | u | u | u |
| *o | u | u | ʰu | u | u | u | u |
| *u | u | u | ^ʔ^u, N | u, N | u, N, ø | u, N, ø | u, N, ø |
| *ɨ | o_(2)_ | o | u | u | u | u | u |
| *i | i_(1)_ | i | ^ʔ^i, N | ^ʔ^i, ^y^i, N | ɿ, ɯ, s, N, ø | N, ø | i, N, ø |
| *e | i_(1)_ | i | ʰɨ, i | ʰi, i | i | i | i |
| *e | e_(1)_ | e | yu | yu | yu | yu | du |

**5.3. Reconstruction of the basic consonant inventory of Proto-Koreanic**

| pK | MK |
| --- | --- |
| *p | p, W /β/ *>* w (lenition) |
| *t | t, l /r/ (lenition) |
| *c | c |
| *k | k, G /ɣ/ > ø (lenition) |
| *h | h |
| *s | s, ž (lenition) |
| *m | m |
| *n | n |
| *r | l |

**5.4 Reconstruction of the basic vowel inventory of Proto-Koreanic**

| pK | MK |
| --- | --- |
| *a | a |
| * ʌ | o /ʌ/ |
| *ɨ | u /ɨ/ |
| *e | e |
| *o | wo /o/ |
| *u | wu /u/ |
| *i | *i |
| *ia | *ye |

**5.5 Reconstruction of the basic consonant inventory of Proto-Tungusic**

| pTg | Jur. | Ma. | Sibe | Oroch | Ud. | Na. | Na. Bikin | Orok | Olcha | Even | Sol. | Evk. | Neg. |
| --- | --- | --- | --- | --- | --- | --- | --- | --- | --- | --- | --- | --- | --- |
| *p- | f- | f- | f- | x- | x- | p- | f- x- | p- | p- | h- | ø- | h- | x- |
| *-p- | f | f b ø | v ø | p w ø | p f w | p | f | p | p | b w ø | p w g ø | p b  w ø | p w |
| *b- | b- | b- | b- | b- | b- | b- | b- | b- | b- | b- | b- | b- | b- |
| *-b- | b w ø | b f w ø | v ø | b w ø | b w ø | b w ø | w ø | b w ø | b w ø | b w ø | b p w ø | w ø | w ø |
| *t- | t- | t- | t- | t- | t- | t- | t- | t- | t- | t- | t- | t- | t- |
| *-t- | t | t | t s | t | t | t | t | t | t | t | t | t | t |
| *d-  *ji- | d-  ʤi- | d-  ʤi- | d-  ʤi- | d- | d- | d-  ʤi- | d-  ʤi- | d-  ʤi- | d- | d- | d- | d- | d- |
| *-d-  *-ji- | d  ʤi | d  ʤi | d  ʤi | d | d | d  ʤi | d  ʤi | d  ʤi | d | d | d | d | d |
| *k- | x- | x- | x- | k- ø- | k- ø- | k- ø- | k- ø- | k- ø- | k- ø- | k- ø- | x- ø- | k- ø- | k- ø- |
| *-k- | k x ø | k x | k ɣ | k ø | k x ɣ ø | k ɣ ø | k ɣ ø | k ø | k ɣ ø | k | k x | k x | k x |
| *g- | g- | g- | g- | g- ŋ- | g- ŋ- | g- ø- | g- ø- | g- ŋ- | g- ŋ- | g- ŋ- | g- n- | g- ŋ- | g- ŋ |
| *-g- | ɣ w ø | ɣ w y ø | ø | ɣ w y ø | ɣ w y ø | ɣ w y ø | y ø | ɣ w y ø | ɣ w y ø | ɣ y | ɣ ø | ɣ | ɣ y w |
| *č- | č- | č- | č- | č- | č- | č- | č- | č- > t- | č- > t- | č- | s- | č- | č- |
| *-č- | č | č | č | č | s | č | č s | č > t | č | č | š | č | č |
| *x- | w- ø- | w- ø- | v- ø- | x- ø- | w- ø- | x- s- | x- s- | x- s- | x- s- | ø- | ø- | ø- | ø- |
| *-x- | x | x | x k ɣ | k | ø | x ø | x k | x ø | x ø | k | x | k | k x |
| *s- | s- | s- | s- | s- | s- | s- | s- | s- | s- | s- | s- | s- | s- |
| *-s- | s | s | s | s | s h ø | s | s | s | s | s | s | x | s |
| *m- | m- | m- | m- | m- | m- | m- ŋ- | m- | m- | m- ŋ- | m- | m- | m- | m- |
| *-m- | m | m | m | m | m | m | m | m | m | m | m | m | m |
| *n- | n- | n- | n- | n- | n- | n- l- | n- l- | n- l- | n- l- | n- | n- | n- l | n- |
| *-n- | n | n | n | n | n | n | n | n | n | n | n | n | n |
| *-r- | r | r | r | y ø | y ø | r | r | r | r | r | r | r | y ø |
| *-l- | l | l | l | l | l | l | l n | l | l | l | l | l | l |

**5.6 Reconstruction of the basic vowel inventory of Proto-Tungusic**

| pTg | Jur. | Ma. | Sibe | Oroch | Ud. | Na. | Na. Bikin | Orok | Olcha | Even | Sol. | Evk. | Neg. |
| --- | --- | --- | --- | --- | --- | --- | --- | --- | --- | --- | --- | --- | --- |
| *a | a | a | a | a | a | a | a | a | a | a | a | a | a |
| *e | e | e | e | e | e | e | e | e | e | e | e | e | e |
| *o | o | o | o | o | o | o | o | o | o | o | o | o | o |
| *ö | u | u | u | o u | o | u | u | o u | o u | o | u | u | u |
| *u | u | u | u | u | u | u | u | u | u | u ö | u ö | u ö | u ö |
| *ü | u ei | u ei | u i | i | i u | u o | i o | i u | i u o | i | i | i u | i o |
| *i | i | i | i | i | i | i ị | i ị | i | i ị | i | i | i ị | i ị |
| *ia | ie ^i^a | iya ai ^i^a | ia a | ^i^æ ei | ^i^æ æ a | ^i^a ea | ^i^æ | ị: | ị: | ia | ị: | ị: | ị: |

**5.7 Reconstruction of the basic consonant inventory of Proto-Mongolic**

| PMo | MMo | WMo | Khal. | Bur. | Kalm. | Dag. | EYu. | Mgr. | Dgx. | Bao. | Mog. |
| --- | --- | --- | --- | --- | --- | --- | --- | --- | --- | --- | --- |
| *p | h  VøV  Cb | ø | ø | ø | ø | x š | h | x f š | x f š | x f š | ø Ɂ |
| *b | b | b | b-  -v- | b | b-  -w- | b  -v-  -r | b- p-  -w- | b  -ø-  -yi | b- p-  -v- | b- p-  -v- | b  -b- -f |
| *t | t | t | t | t | t | t | t d | t d | t  -d | t | t |
| *d | d  -t | d | d | d | d | d  -r | d | d | d j | d | d |
| *k(A) | q x | q | x | x | x | k x | q x | x | q | x | q |
| *k(E) | k | k | x | x | k | k x | k | k | k | k | k |
| *k(i) | k | k | x | x | k | k x | k | ć | k | k | k |
| *g(A) | q  -ɣ- | ɣ | g | g | g | g | ġ | ġ | ġ | ġ | ɣ |
| *g(E) | g | g | g | g | g | g | g | g | g | g | g |
| *g(i) | g | g | g | g | g | g | g | g | g | g | g |
| *č | č | č | č | s š | č | č | ć ǯ | č  -ǯ- | č  -ǯ- | č  -ǯ-  -ć | č |
| *s | s š | s | s š | h š  -t | s š | s š  -r | s š | s š ʒ | s š | s š | s š |
| *m | m | m | m  -m -n | m  -m -n | m  -m -n | m  -m -n | m | m | m  -n | m | m |
| *n | n | n | n | n | n | n | n | n | n | n  -ŋ | n |
| *-r- | -r-  -r | -r-  -r | -r-  -r | -r-  -r | -r-  -r | -r-  -r | -r-  -r | -r-  -r | -r-  -ø | -r-  -r | -r-  -r |
| *-l- | -l-  -l | -l-  -l | -l-  -l | -l-  -l | -l-  -l | -l-  -l | -l-  -l | -l-  -l | -l-  -n | -l-  -l | -l-  -r |

**5.8 Reconstruction of the basic vowel inventory of Proto-Mongolic**

| PMo | MMo | WMo | Khal. | Bur. | Kalm. | Dag. | EYu. | Mgr. | Dgx. | Bao. | Mog. |
| --- | --- | --- | --- | --- | --- | --- | --- | --- | --- | --- | --- |
| *a | a | a | a | a | a ä | a | a | a ä i  ø- | a ə | a e i | a o ö |
| *e | e | e | e i ö | e ü | e i ö | e ü | e  i- o- ø- | e ə a i u  ø- | e ie ü  ye- | e  -iN  -aN | e ü |
| *o | o | o | o | o | o ö | o  -(u)a-  wa- | o ö | o u ö  u- ø- | o u  -uaN | o u  o- | o u |
| *ö | o u | ö | ö | ü | ö | ü | ö o: | u o o:  o- ø- | o u  -uaN | o u  o- | ö ü |
| *ü | u | ü | ü | ü | ü | ü | u ə  ø- u- | u ə i  ø- u- | u | u e  u-  -oŋ | ü |
| *u | u | u | u | u | u ü | u o  -(u)a-  wa- | u ə  ø- | u o ə  ø- | u | u e a o | u |
| *i | i | i | i | i e | i | i | i | i | i ə | i | i |

**5.9 Reconstruction of the basic consonant inventory of Proto-Turkic**

|  | OT | Chu. | Kh. | CTat | Kum. | KBal. | Krm. | Tat. | Bash. | Kaz. | Kirg. | Kpak | Nog. |
| --- | --- | --- | --- | --- | --- | --- | --- | --- | --- | --- | --- | --- | --- |
| *-p-  *-p | p  -p | b  -p | b  -p | b p  -p | b  -p | b  -p | b  -p | b p  -p | b  -p | b  -p | b p  -p | b p  -p | b  -p |
| *b- | b- | p- | b- | b- | b- | b- | b- | b- | b- | b- | b- | b- | b- |
| *-b-  *-b | b | v ø | v ø | v | w y ø | w y ø | w y ø | w y ø | w y ø | b w y ø | b y  -ø | b w y ø | b w y ø |
| *t- | t- | t- č- | t- | t- | t- | t- | t- | t- | t- | t- | t- | t- | t- |
| *-t-  *-t | t | -d-  -t | t | -d-  -t | t | t | t | t | t | t | t | t | t |
| *y- | d- | y- | d- | y- | y- | y- | y- | y- | y- | y- | y- | y- | y- |
| *-d-  *-d | d | y r  -ø | d | y | y | y | y | y | y | y | y | y | y |
| *k(A)- | q- | x- | q- | q- | q- | q- | q- | q- | q- | q- | k- | q- | k- |
| *k(E) | k- | k- | k- | k- | k- g- | k- | k- | k- | k- | k- | k- | k- | k- |
| *-k(A)-  *-k | q | -g-  -k -ø | q | -ɣ-  -q | -ɣ-  -q | -ɣ-  -q | -ɣ-  -q | -ɣ-  -q | -ɣ-  -q | -ɣ-  -q | -ɣ-  -q | -ɣ-  -q | -ɣ-  -q |
| *-k(E)-  *-k(E) | k | -g-  -k -ø | k | -g-  -k | -g-  -k | -g-  -k | -g-  -k | -g-  -k | -g-  -k | -g-  -k | -g-  -k | -g-  -k | -g-  -k |
| *-g(A)-  *-g(A)  *-g | ɣ | ø | ø | w y  -ø -w -y | w y  -ø -w -y | w y  -ø -w -y | g w y ø | w y  -ø -w -y | w y  -ø -w -y | w y  -ø -w -y | w y  -ø -w -y | w y  -ø -w -y | w y  -ø -w -y |
| *-g(E)-  *-g(E)  *-g | g | v y | ɣ w y | w y  -ø -w -y | w y  -ø -w -y | w y  -ø -w -y | g w y ø | w y  -ø -w -y | w y  -ø -w -y | w y  -ø -w -y | w y  -ø -w -y | w y  -ø -w -y | w y  -ø -w -y |
| *-č-  *-č | č | -ź- -ś | č | j č  -č | č | č | j  -č | č | s | š | č | š | š |
| *-lč | -š | -ś | -š | -š | -š | -š | -š | -š | -š | -s | -š | -s | -s |
| *s- | s- | s- š- | s- | s- | s- | s- | s- | s- | h- | s- | s- | s- | s- |
| *-s-  *-s | s | z ž  -s -š | s | s | s | s | s | s | θ | s | s | s | s |
| *-m-  *-m- | m  -n -m | m  -n  -m | m  -n  -m | m  -n  -m | m  -n  -m | m  -n -m | m  -n  -m | m  -n  -m | m  -n  -m | m  -n  -m | m  -n  -m | m  -n  -m | m  -n  -m |
| *-n-  *-n | n | n m | n | n | n | n | n | n | n | n | n | n | n |
| *-r- | r | r | r | r | r | r | r | r | r | r | r | r | r |
| *-r_2_- | z | r | z | z | z | z | z | z | δ | z | z | z | z |
| *-l- | l | l | l | l | l | l | l | l | l | l | l | l | l |

| PTk | Tk. | Az. | Gag. | Tkm. | Uz. | Uigh. | Tuv. | Tofa | Shor | Khak. | Yak. | Dolg. |
| --- | --- | --- | --- | --- | --- | --- | --- | --- | --- | --- | --- | --- |
| *-p-  *-p | b p  -p | b p  -p | b p  -p | b p  -p | v p b  -v -p -b | p  -p | -v-  -p | b p  -p | b  -p | b  -p | b  -p | b  -p |
| *b- | b- | b- | b- | b- | b- | b- | b- | b- | p- | p- | b- | b- |
| *-b-  *-b | v y ø | v y ø | v ø | w y ø | v g y ø | b v g y ø | v ø  -g | b ø  -g | b ø  -g | b ø  -g | b ø | b ø |
| *t- | t- d- | t- d- | t- d- | t- d- | t- | t- | t- d- | t- d- | t- | t- | t- | t- |
| *-t-  *-t | t d | t d | t d | -t- -d-  -t | t | t | -d-  -t | -d-  -t | -d-  -t | -d-  -t | t | t |
| *y- | y- | y- | y- | y- | y- | y- | d-  t- | d- | z- | z-  s- | t- | t- |
| *-d-  *-d | y | y | y | y | y | y | -d-  -t | d | z | -z-  -s | t | t |
| *k(A)- | k- g- | k- g- | k- g- | k- g- | q- | q- | q- x- | q- | q- | x- | q- x- | k- |
| *k(E)- | k- g- | ġ- | k- | ġ- | k- | k- | k- | k- | k- | k- | k- | k- |
| *-k(A)-  *-k(A) | -ɣ-  -q | -ɣ-  -q | -k-  -q | -k-  -q | q | q | -g-  -q | -g-  -q | -ɣ-  -q | -ɣ-  -q | -ɣ-  -x | -g-  -k |
| *-k(E)-  *-k(E) | -g-  -k | -g-  -k | -g-  -k | -g-  -k | k | k | -g-  -k | -h-  -k | -g-  -k | -g-  -k | -g-  -k | -g-  -k |
| *-g(A)-  *-g | ɣ w y ø | ɣ w y ø | ɣ w y ø | ɣ w y ø | -ɣ-  -q | -ɣ-  -q | ɣ ø | ɣ ø | ɣ ø | ɣ ø | ø | ø |
| *-g(E)-  *-g | ɣ w y ø | ɣ w y ø | ɣ w y ø | ɣ w y ø | -g-  -k | -g-  -k | g ø | g ø | g ø | g ø | ø | ø |
| *-č-  *-č | č j  -č | č j  -č | č j  -č | č j  -č | č | č | -ž-  -š | -j-  -š | č  -š | č  -s | -h-  -s | č  -s |
| *-lč | -š | -š | -š | -š | -š | -š | -š | -š | -š | -s | -s | -s |
| *s- | s- | s- | s- | s- | s- | s- | s- | s- | s- | s- | ø- | ø- |
| *-s-  *-s | s | s | s | s | s | s | -z-  -s | s | -z-  -s | -z-  -s | t | t |
| *-m-  *-m | m  -n  -m | m  -n  -m | m  -n  -m | m  -n  -m | m  -n  -m | m  -n  -m | m  -n  -m | m  -n  -m | m  -n  -m | m  -n  -m | m  -n  -m | m  -n  -m |
| *-n-  *-n | n | n | n | n | n | n | n | n | n | n | n | n |
| *-r- | r | r | r | r | r | r | r | r | r | r | r | r |
| *-r_2_- | z | z | z | z | z | z | s | s | s | s | s | s |
| *-l- | l | l | l | l | l | l | l | l | l | l | l | l |

**5.10 Reconstruction of the basic vowel inventory of Proto-Turkic**

| PTk | OT | Chu. | Kh. | CTat | Kum. | KBal. | Krm. | Tat. | Bash. | Kaz. | Kirg. | Kpak | Nog. |
| --- | --- | --- | --- | --- | --- | --- | --- | --- | --- | --- | --- | --- | --- |
| *a | a | u | a | a | a | a | a | a | a | a | a | a | a |
| *e | e | i | ä e | e | e | e | e | i | i | e | e | e | e |
| *o | o | u | o | o | o | o | o | u | o | o | o | o | o |
| *ö | ö | ü ă | e ö | ö o | ö | ö | ö | ü | ü | ö | ö | ö | ö |
| *u | u | vă-  -ă- | u | u | u | u | u | o | o | ŭ | u | u | u |
| *ü | ü | ă | ü i | ü u | ü | ü | ü | ö | ö | ü | ü | ü | ü |
| *ï | ï | ă ĕ | ï | ï i | ï | ï | ï | ï | ï | ï | ï | ï | ï |
| *i | i | ă ĕ | i | i | i | i | i | e | e | ĭ | i | i | i |
| *ia | a | yu | a | a | a | a | a | a | a | a | a | a | a |

| PTk | Tk. | Az. | Gag. | Tkm. | Uz. | Uigh. | Tuv. | Tofa | Shor | Khak. | Yak. | Dolg. |
| --- | --- | --- | --- | --- | --- | --- | --- | --- | --- | --- | --- | --- |
| *a | a | a | a | a | a e | ɔ a ä | a | a | a | a | a | a |
| *e | e | ä e | e | e | e | ä e | e | e | e | i | i | i |
| *o | o | u | o | o | ọ | o | o | o | o | o | o | o |
| *ö | ö | ö | ö | ö | ọ | ö | ö | ö | ö | ö | ö | ö |
| *u | u | u | u | u | u | u | u | u | u | u | u | u |
| *ü | ü | ü | ü | ü | u | ü | ü | ü | ü | ü | ü | ü |
| *ï | ï | ï i | ï | ï | i | i | ï | ï | ï | ï | ï | ï |
| *i | i | i | i | i | i | i | i | i | i | ɘ | i | i |
| *ia | a | a | a | a | a e | ɔ | a | a | a | a | a | a |

**5.11 Consonant correspondences between the Transeurasian languages**

|  | pJ | pK | pTg | pMo | pTk | pTEA |
| --- | --- | --- | --- | --- | --- | --- |
| 1. | *p- | *p- | *p- | *p- | *b-/ *p- | *p- |
| 2 | *-p- | *-p- | *-p- | *-ɣ- | *-p- | *-p- |
| 3. | *p- / *w- | *p- | *b- | *b- | *b- | *b- |
| 4. | *-p-/*-w- | *-p- | *-b- | *-b-/ -ɣ- | *-b- | *-b- |
| 5. | *-np- | *-pC- | *-PC- | *-PC- | *-P(C)- | *-m^(P)^T- |
| 6. | *-np- | *-Rp- | -RP- | *-RP- | *-RP- | *-Rp- |
| 7. | *t- | *t- | *t- | *t- | *t- | *t- |
| 8. | *-t- | *-t- | *-t- | *-t- | *-t- | *-t- |
| 9. | *t- /*y- | *t- (ci-) | *d- (ji-) | *d- (ji-) | *y- | *d- |
| 10. | *-t-/ *-y- | *-l- | *-d- (-ji-) | *-d- (-ji-) | *-d- | *-d- |
| 11. | *-nt- | *-c- | *-TC- | *-TC- | *-TC- | *-n^(T)^K- |
| 12. | *-nt- | *-Rc- | *-RT- | *-RT- | *-RT- | *-Rt- |
| 13. | *k- | *k- | *k- | *k- | *k- | *k- |
| 14. | *-k- | *-k- (-h-) | *-k- | *-k- | *-k- | *-k- |
| 15. | *k- | *k- | *g- | *g- | *k- | *g- |
| 16. | *-k- | *-k- (-h-) | *-g- | *-g- | *-g- | *-g- |
| 17. | *-nk- | *-kC- | *-KC- | *-KC- | *-KC- | *-ŋ^(K)^T- |
| 18. | *-nk- | *-Rk- | *-RK- | *-RK- | *-RK- | *-Rk- |
| 19. | *t- | *c- | *č- | *č- | *č- | *č- |
| 20. | *-t- | *-c- | *-č- | *-č- | *-č- | *-č- |
| 20b. | *-si | *-l(i)/ -c | *-l(č) | *-l(č) | *-l(č)~ -š | *-lč |
| 21. | *k- | *k-, h- | *x- | *k- | *k- | *x- |
| 22. | *-k- | *-k- | *-x- | *-g-~-k- | *-g-~-k- | *-x- |
| 23. | *s- | *s- | *s- | *s- | *s- | *s- |
| 24. | *-s- | *-s- | *-s- | *-s- | *-s- | *-s- |
| 25. | *m- | *m- | *m- | *m- | *b- | *m- |
| 26. | *-m- | *-m- | *-m- | *-m- | *-m- | *-m- |
| 27. | *n- | *n- | *n- | *n- | *y- | *n- |
| 28. | *-n- | *-n- | *-n- | *-n- | *-n- | *-n- |
| 29. | *-r- | *-l- | *-r- | *-r- | *-r- | *-r- |
| 30. | *-r- | *-l- | *-r- | *-r- | *-r_2_- | *-r- |
| 31. | *-r- | *-l- | *-l- | *-l- | *-l- | *-l- |

**5.12. Vowel correspondences between the Transeurasian languages**

|  | OJ < pJ | MK < pK | pTg | pMo | pTk | pTEA |
| --- | --- | --- | --- | --- | --- | --- |
| 32. | -a- < *-a- | -a- < *-a- | *-a- | *-a- | *-a- | *-a- |
| 32b. | *CaCa | *CʌCʌ | *CaCa | *CaCa | *CaC | *CaCa |
| 33. | -a- < *-a- | -e- < *-e- | *-e- | *-e- | *-e- | *-ə- |
| 34. | -o- < *-ə- | -e- < *-e- | *-e- | *-e- | *-e- | *-ə- |
| 35. | -o-  <? *-o- | -wo-  < *-o- | *-o- | *-o- | *-o- | *-ɔ- |
| 36. | -u- < *-o- | -wo-  < *-o- | *-o- | *-o- | *-o- | *-ɔ- |
| 37. | -o- < *-ɨ- | -u- < *-ɨ- | *-ö- | *-ö- | *-ö- | *-o- |
| 38. | -u- < *-u- | -wu-  < *-u- | *-u- (gü) | *-ü- | *-ü- | *-u- |
| 39. | -u- < *-u- | -o- < *-ʌ- | *-u- | *-u- | *-u- /-ï- | *-ʊ- |
| 39b. | PaRu- < *PauRu- | *PʌRʌ- ~ *PɨRɨ- | *PuRu- | *PuRu- | *PuR- | *PʊRʊ- |
| 40. | -i- < *-i- | -i- < *-i- | *-i- | *-i- | *-i-/-ï- | *-i- |
| 40b | -i- < *-e- | -ye-  < *-ia- | *-ia- | *-ia- | *-ia- | *-ia- |
| 40c | -e_(1)_ -  < *-e- | -ye-  < *-ia- | *-ia- | *-ia- | *-ia- | *-ia- |
| 40 d | -o- < *-ə- | -e- < *-ə- | *-ü- | *-ö- | *-ö- | *-iu- |
| 41. | a- < *-a- | a- < *a- | *a- | *a- | *a- | *a- |
| 41b. | a- < *-a- | e- < *e- | *e- | *e- | *e- | *ə- |
| 42. | o- < *ə- | e- < *e- | *e- | *e- | *e- | *ə- |
| 43. | o- <? *o- | wo-  < *o- | *o- | *o- | *o- | *ɔ- |
| 44. | o- < *ɨ- | ø < ? *ɨ- | *ö- | *ö- | *ö- | *o- |
| 45. | u- < *u- | wu- < *u- | *u- | *u- | *u- | *u- |
| 46. | i- < *i- | i- < *i- | *i- | *i- | *i- | *i- |
| 46b | i- < *e- | ye- < *ia- | *ia- | *ya- | *ya- | *ia- |
| 46c | ya- < *ia- | ye-  < *ia- | *ia- | *ya- | *ya- | *ia- |
| 46d | o- < *-ə- | ye- <*iə | *ü- | *ö- | *ö- | *iu- |

**References mentioned in Supplementary Information 5**

An, Z. Zhongguo gudai de shidao [Stone knives in ancient China]. *Acta Archaeologica Sinica* **10**, 27–51 (1955).

Antonov, A. *Le rôle des suffixes nominaux en /+rV/ dans l’expression du lieu et de la direction en japonais et l’hypothèse de leur origine “altaïque”* (Institut National des Langues et Civilisations Orientales Ph.D. dissertation, 2007).

Barber, E. *The Mummies of Urumchi* (Norton, 1985).

Barnes, G. *The rise of civilization in East Asia. The archaeology of China, Korea and Japan* (Thames and Hudson, 1993).

Baxter, W. H., & Sagart, L. *Old Chinese: A New Reconstruction* (Oxford Univ. Press, 2014). [https://www.researchgate.net/publication /265110209_Baxter-Sagart_Old_Chinese_reconstruction_version _of_20_February_2011](https://www.researchgate.net/publication/265110209_Baxter-Sagart_Old_Chinese_reconstruction_version_of_20_February_2011)

Blažek, V. in *Etymology and the European Lexicon. Proceedings of the 14th Fachtagung der Indogermanischen Gesellschaft (17-22 September 2012, Copenhagen)* (eds Sandgaard Hansen, B. S., Nielsen Whitehead, B., Olander, T. & Olsen, B. A.) 53-68 (Reichert Verlag, 2017).

Beckwith, C. I. (2007). Koguryo, the Language of Japan’s Continental Relatives: An Introduction to the Historical-Comparative Study of the Japanese-Koguryoic Languages with a Preliminary Description of Archaic Northeastern Middle Chinese (Brill, 2007).

Bentley, J. R. A New Look at Paekche and Korean: Data from Nihon Shoki. Language Research 36.2, 417–443 (2000).

Benzing, J. Die tungusischen Sprachen: Versuch einer vergleichenden Grammatik. *Abhandlungen der geistes- und sozialwissenschaftlichen Klasse* *1955* **11** (Franz Steiner Verlag, 1955).

Boivin, N., Fuller, D. & Crowther, A. Old World globalization and the Columbian exchange: comparison and contrast. *World Archaeology* **44(3)**, 452-469 (2012).

De Smedt, A. & Mostaert, A. *Le dialecte monguor parlé par les mongols du Kansou occidental* (Imprimerie De L'Universite Catholique, 1933).

Doerfer, G. *Türkische und mongolische Elemente im Neupersischen, unter besonderer Berücksichtigung älterer neupersischer Geschichtsquellen, vor allem der Mongolen- und Timuridenzeit.* *Vol. 1-4* (Franz Steiner, 1963-1965-1967-1975).

Doerfer, G. *Mongolo-Tungusica* (Harrassowitz, 1985).

Dybo, A. Language and archeology: some methodological problems. 1. Indo-European and Altaic landscapes. *J. Lang. Relation.* **9**, 69–92 (2013).

Erdal, M. *Old Turkic word formation: A functional approach to the lexicon* (Turcologica 7.) (Harrassowitz, 1991).

Francis-Ratte, A. *Proto-Korean-Japanese: A New Reconstruction of the Common Origin of the Japanese and Korean Languages* (The Ohio State University PhD dissertation, 2016).

Francis-Ratte, A. & Unger, J. M. in *The Oxford Guide to the Transeurasian Languages* (eds Robbeets, M. & Savelyev, A.) 705–714 (Oxford Univ. Press, 2020).

Frellesvig, B. & Whitman, J. in *Proto-Japanese: Issues and prospects* (Current Issues in Linguistic Theory 249) (eds Frellesvig, B. & Whitman, J.) 15–41 (Benjamins, 2008).

Fuller, D.Q. & Rowlands, M. in *Interweaving Worlds: Systemic Interactions in Eurasia, 7th to the 1st Millennia BC* (eds Wilkinson, T.C., Sherratt, S. & Bennet, J.) 37-60 (Oxbow, 2011).

Gruntov, I. Razvitie pramongol’skogo gortannogo spiranta *h- v načal’noj pozitsii v jazyke pamjatnika mongol’skogo jazyka XV veka slovarja “Muqaddimat al-adab*”.* *Orientalia et Classica* **6**, 39-48 (2005).

Hudson, M. J. & Robbeets, M. Archaeolinguistic evidence for the farming/language dispersal of Koreanic. *Evolutionary Human Sciences* **2***,* e52 doi:10.1017/ehs.2020.49 (2020).

Hyllested, A. in *Dispersals and diversification: Linguistic and archaeological perspectives on the early stages of Indo-European (*eds Serangeli, M. & Olander, T.) (Brill, Brill's Studies in Indo-European Languages & Linguistics, 2020).

Hwang, M. S, Morphological Differences between Ramie and Hemp: How These Characteristics Developed Different Procedures in Bast Fiber Producing Industry. *Textile Society of America Symposium Proceedings 23*. <https://digitalcommons.unl.edu/tsaconf/23> (2010).

Janhunen, J. Laryngeals and pseudolaryngeals in Mongolic Problems of phonological interpretation. Central Asiatic Journal **43 (1)**, 115-131 (1999).

Janhunen, J. in *Recent Advances in Tungusic Linguistics* (Turcologica 89.) (eds Malchukov, A. L. & Whaley, L. J.) 5–16 (Harrassowitz, 2012).

Jeong, Choongwon, et al. Bronze Age population dynamics and the rise of dairy pastoralism on the eastern Eurasian steppe. *Proceedings of the National Academy of Sciences of the United States of America* 10.1073/pnas.1813608115 (2018).

Jeong, C., Wang, K., Wilkin, S., et al. A Dynamic 6,000-Year Genetic History of Eurasia’s Eastern Steppe. *Cell* **183**, 890-904 <https://doi.org/10.1016/j.cell.2020.10.015> (2020).

Kane, D. *The Kitan Language and Script* (Brill, 2009).

Kawashima, T. Food processing and consumption in the Jōmon. *Quaternary International* **404**, 16–24 (2016).

Kroonen, G., Barjamovic, G. & Peyrot, M. in The first horse herders and the impact of early bronze age steppe expansions into Asia (eds Damgaard, P., Martiniano, R., Kamm, J., et al) *Science* **360**, 6396 eaar7711 https://doi.org/10.1126/science.aar7711 (2018).

Kuzmin, Y. V. The Beginnings of Prehistoric Agriculture in the Russian Far East: Current Evidence and Concepts. *Documenta Praehistorica* **40**, 1–12 (2013).

Labrune, L. De l'iconicité en japonais: suffixes en r+voyelle et réduplication. *Cahiers de linguistique - Asie orientale* **27**–**1**, 79–118 doi : <https://doi.org/10.3406/clao.1998.1527> (1998).

Leipe, C., Long, T., Sergusheva E.A. et al. Discontinuous spread of millet agriculture in eastern Asia and prehistoric population dynamics. *Science Advances* **5**, eaax6225 (2019).

Li, K. (ed) *Mongghul Qidar Merlong* [Mongghul-Chinese dictionary] (Qinghai Peoples Press, 1988).

Liu, L. et al. Plant-based Subsistence Strategies and Development of Complex Societies in Neolithic Northeast China: Evidence from Grinding Stones. *Journal of Archaeological Science: Reports* **7**, 247–261 (2016).

Liu, L. & Xingcang, C. *The Archaeology of China: From The Late Paleolithic to the Early Bronze Age* (Cambridge Univ. Press, 2012).

Liu X. et al. From ecological opportunism to multi-cropping: Mapping food globalisation in prehistory. *Quaternary Science Reviews* **206**, 21–28 <https://doi.org/10.1016/j.quascirev.2018.12.017> (2019).

Mallory, J. P. & Adams, D. Q. in Encyclopedia of Indo-Europe*an culture (*eds Mallory, J. P. & Adams, D. Q.) 590-594 (Taylor & Francis, 1997).

Martin, S. E. *Consonant lenition in Korean and the Macro-Altaic question* (University of Hawai‘i Press, 1996).

Matisoff, J. STEDT The Sino-Tibetan Etymological Dictionary and Thesaurus

<https://stedt.berkeley.edu/~stedt-cgi/rootcanal.pl/etymon/487> (n.d.).

Menges, K. Dravidian and Altaic. *Anthropos* 72: 129-179 (1977).

Miller, R. A. Archaeological light on Japanese linguistic origins. *Asian Pac. Quart. Soc. Cult. Affairs* **22**, 1-26 (1990).

Miller, R. A. *Languages and History. Japanese, Korean, and Altaic* (White Orchid Press, 1996).

Miyake, M. H. Pre-Sino-Korean and Pre-Sino-Japanese: Reexamining an Old Problem from a Modern Perspective. *JKL* **6**, 179-211 (1997).

Nelson, S. *The archaeology of Korea* (Cambridge Univ. Press, 1993).

Nelson, S. et al. Tracing population movements in ancient East Asia through the linguistics and archaeology of textile production. *Evolutionary Human Sciences,* 1-20 doi:10.1017/ehs.2020.4 (2020).

Ning, C., Li, T., Wang, K., *et al.* Ancient genomes from northern China suggest links between subsistence changes and human migration. *Nat. Comm.* **11**, 2700 (2020).

Nugteren, H. *Mongolic phonology and the Qinghai-Gansu languages* (LOT. Leiden University Ph.D. dissertation, 2011).

Ogura, S. Ine to bosatsu. *Minzokugakukenkyū*, **309 (7)**, 695–725 (1943).

Omura, M. & Kizawa, N. in *Textile terminologies from the Orient to the Mediterranean and Europe, 1000 BC to 1000 AD (*eds Gaspa, S., Michel, C. & Nosch, M.-L.) 451–482 (Zea Books) doi: 10.13014/K2RN361H (2017).

Peyrot, M. in *Talking Neolithic: Proceedings of the workshop on Indo-European origins held at the Max Planck Institute for Evolutionary Anthropology, Leipzig, December 2–3, 2013* (eds Kroonen, G., Mallory, J. P. & Comrie, B.) 242–277 (Institute for the Study of Man, 2018).

Poppe, N. *Vergleichende Grammatik der altäischen Sprachen* [Comparative Grammar of the Altaic Languages]. *Teil I: Vergleichende Lautlehre* (Otto Harrassowitz, 1960).

Poppe, N. On some ancient Mongolian loanwords in Tungus. *Central Asiatic Journal* **11,** 189-197 (1966).

Renfrew, C. *Archaeology and Language: The Puzzle of Indo-European Origins* (Penguin Books, 1987).

Robbeets, M. *Is Japanese related to Korean, Tungusic, Mongolic and Turkic?* (Harrassowitz, 2005).

Robbeets, M. in *Copies vs. cognates in bound morphology* (Brill’s Studies in Language, Cognition and Culture) (eds Johanson, L. & Robbeets, M.) 427-446 (Brill, 2012).

Robbeets, M. *Diachrony of verb morphology. Japanese and the Transeurasian languages* (Trends in Linguistics 291.) (De Gruyter Mouton, 2015).

Robbeets, M. in *The Uppsala Meeting. Proceedings of the 13th International Conference on Turkish Linguistics* (eds Csató, É., Karakoç, B. & Menz, A.) 199-212 (Harrassowitz, 2016).

Rozycki, W. *Mongol Elements in Manchu* (Indiana University, 1994).

Rozycki, W. in *Proceedings of the 40^th^ meeting of the Permanent International Altaistic Conference (PIAC). Indiana University Research Institute for Inner Asian Studies* (eds Honey, D. & Wright, D.) 236-245 (Indiana University, 2001).

Sagart, L. [The names of the rice plant. II ‘Tibeto-Burman’.](https://stan.hypotheses.org/248) *Sino-Tibetan-Austronesian* <https://stan.hypotheses.org/tag/rice> (2018).

Sagart, L., Jacques, G., Lai, Y. et al. Dated language phylogenies shed light on the ancestry of Sino-Tibetan. *Proc. Natl Acad. Sci*. **116**, 10317–10322 (2019).

Schuessler, A. *ABC etymological dictionary of Old Chinese* (University of Hawaii Press, 2007).

Shelach-Lavi, G., Teng, M., Goldsmith, Y. et al. Sedentism and plant cultivation in northeast China emerged during affluent conditions. *PLoS ONE* 14:e0218751 (2019).

Sergusheva, E. A. Archaeobotanical Studies of Late-Neolithic Sites in Primorye. *OPUS: Interdisciplinary Investigation in Archaeology* **6,** 180–195 (2009).

Sergusheva, E. A., & Vostretsov, Y. E. in *From Foragers to Farmers: Papers in Honour of Gordon C. Hillman* (eds Fairbairn, A. & Weiss, E.) 205–219 (Oxbow Books, 2009).

Taylor, W. T. T., Clark, J., Bayarsaikhan, J., Tuvshinjargal, T., Thompson Jobe, J. et al. Early Pastoral Economies and Herding Transitions in Eastern Eurasia. Nature Scientific Reports **10**, 1001 <https://doi.org/10.1038/s41598-020-57735-y> (2020).

Starostin, S. in *Past Human Migrations in East Asia: Matching Archaeology, Linguistics and Genetics* (Routledge Studies in the Early History of Languages 5) (eds Sanchez-Mazas, A., Blench, R., Ross, M. D., Peiros, I. & Lin, M.) 254-262 (Routledge, 2008).

Starostin, S. A., Dybo, A. & Mudrak, O. *Etymological Dictionary of the Altaic Languages* (Brill, 2003).

Stevens, C., Shelach-Lavi G., Zhang, H., Teng, M. and Fuller, D. A model for the domestication of Panicum miliaceum (common, proso or broomcorn millet) in China. *Vegetation History and Archaeobotany*, <https://doi.org/10.1007/s00334-020-00804-z> (2020).

Soffer, O. J., Adovasio, M. & Hyland, D. C. The “Venus” figurines. Textiles, basketry, gender, and status in the Upper Paleolithic. *Current Anthropology* **41**, 511-537 (2000).

Susano, Y. Procedure of brewing alcohol as a staple food: case study of the fermented cereal liquor “Parshot” as a staple food in Dirashe special woreda, southern Ethiopia. *Food Science & Nutrition* **4(4)**, 544–554 doi: 10.1002/fsn3.316 (2016).

Svantesson, J.-O., Tsendina, A., Mukhanova Karlsson, A. & Franzén, F. *The Phonology of Mongolian* (Oxford, 2005).

Tedlock, B. *The Woman in the Shaman's Body: Reclaiming the Feminine in Religion and Medicine* (Random House Publishing Group, 2009).

Tozaki, et al. Microsatellite variation in Japanese and Asian horses and their phylogenetic relationship using a European horse outgroup. *Journal of Heredity* **94(5)**, 374–380 (2003).

Unger, J. M. *The Role of Language Contact in the Origins of the Japanese and Korean Languages* (University of Hawai‘i Press, 2009).

Vovin, A. *A Reconstruction of Proto-Ainu* (Brill’s Japanese Studies Library 4.) (Brill, 1993).

Vovin, A. in *Archaeology and Language II* (eds Blench, R. & Spriggs, M.) 366–378 (Routledge, 1998).

Vovin, A. *Korea-Japonica: A Re-Evaluation of a Common Genetic Origin* (University of Hawai‘i Press, 2010).

Vovin, A. On the Etymology of Middle Korean *psʌr* ‘rice’. *Türk Dilleri Araştırmaları* **25.2**, 229–238 (2015).

Wang, C-C. & Robbeets, M. The homeland of Proto-Tungusic inferred from contemporary words and ancient genomes. *Evolutionary Human Sciences* **2**, e8 doi:10.1017/ehs.2020.8 (2020).

Whitman, J. *The Phonological Basis for the Comparison of Japanese and Korean* (Harvard University Ph.D. dissertation, 1985).

Wilkin, S. et al. Dairy pastoralism sustained eastern Eurasian steppe populations for 5000 years. *Nature Ecol. Evol.* **4**, 346-355 (2020).

Yu, J., Wang, Y., He, J., Feng, Y., Li, Y., Li, W. Xinjiang Jimunai Xian Tongtiandong

yizhi (Tongtiandong at Jimunai county, Xinjiang). *Kaogu (Archaeology)* **610**, 723e734 (2018).

Zakharov, I. I. *Polnyj maňčžursko-russkij slovaŕ* [A comprehensive Manchu-Russian dictionary] (Tipografija imperatorskoj Akademii nauk, 1875).

Zhang, H., Ji, T., Pagel, M., et al. Dated phylogeny suggests early Neolithic origin of Sino-Tibetan languages. *Nature* *Scientific Reports* **10**, 20792 (2020).

**Additional References SI 23a Turkic**

Ašmarin, N. *Thesaurus Linguae Tschuvaschorum*. *Vol. 1–17* (Krasnyj Pečatnik & Čuvašskaja Kniga & Čuvašpoligrafizdat, 1928-1950).

Bailey, H. W. *Dictionary of Khotan Saka* (Cambridge Univ. Press, 1979).

Bammatov, Z. Z. *Russko-kumykskij slovar'* [Russian-Kumyk dictionary] (Gosudarstvennoe izdatel'stvo inostrannyx i nacional'nyx slovarej, 1960).

Bammatov, Z. Z. *Kumyksko-russkij slovar'* [Kumyk-Russian dictionary] (Sovetskaja enciklopedija, 1969).

Baskakov, N. A. & Toščakova, T. M. *Ojrotsko-russkij slovar'* [Oyrot-Russian dictionary] (Gosudarstvennoe izdatel'stvo inostrannyx i nacional'nyx slovarej, 1947).

Baskakov, N. A. *Karakalpaksko-russkij slovar'* [Karakalpak-Russian dictionary] (Gosudarstvennoe izdatel'stvo inostrannyx i nacional'nyx slovarej, 1958).

Baskakov, N. A. *Nogajsko-russkij slovar'* [Noghai-Russian dictionary] (Gosudarstvennoe izdatel'stvo inostrannyx i nacional'nyx slovarej, 1963).

Baskakov, N. A. *Turkmensko-russkij slovar'* [Turkmen-Russian dictionary] (Sovetskaja enciklopedija, 1968).

Baskakov, N. A. *Gagauzsko-russko-moldavskij slovar'* [Gagauz-Russian-Moldovan dictionary] (Sovetskaja enciklopedija, 1973).

Baskakov, N. A., Zajączkowski, A. & Szapszał, S. *Karaimsko-russko-pol'skij slovar'* [Karaim-Russian-Polish dictionary] (Russkij jazyk, 1974).

Baskakov, N. A. *Turecko-russkij slovar'* [Turkish-Russian dictionary] (Russkij jazyk, 1977).

Bektaev, Q. *Bol'šoj kazaxsko-russkij i russko-kazaxskij slovar'* [Big Kazakh-Russian and Russian-Kazakh dictionary] (Kazaxstanskij projekt razvitija gosudarstvennogo jazyka, 1995).

Bekturov, Š. & Bekturova, A. *Kazaxsko-russkij slovar'* [Kazakh-Russian dictionary] (Foliant, 2001).

Čeremisov, K. M. & Tsydendambaev, Ts. *Burjat-mongol'sko-russkij slovar'* [Buryat Mongol-Russian dictionary] (Gosudarstvennoe izdatel'stvo inostrannyx i nacional'nyx slovarej, 1951).

Chen Z. et al. *Folktales of China’s Minhe Mangghuer* (2005).

Clauson, G. *An Etymological Dictionary of Pre-Thirteenth-Century Turkish* (At the Clarendon Press, 1972).

Doerfer, G. & Tezcan, S. *Wörterbuch des Chaladsch (dialekt von Charrab)* (Akadémiai Kiadó, 1980).

Dybo, A. V. *Lingvističeskije kontakty rannix tjurkov. Leksičeskij fond. Pratjurkskij period* [Linguistic contacts of the early Turks. Vocabulary. Proto-Turkic period] (Vostočnaja literature, 2007).

Dybo, A. V. Vokalizm rannetjurkskix zaimstvovanij v vengerskom [Vocalism of early Turkic borrowings in Hungarian]. *Gedenkschrift von E.A. Helimsky* [*Finnisch-Ugrische Mitteilungen* **32–33**, 83–132 (2010).

Judakhin, K. K. *Kirgizsko-russkij slovar'* [Kirghiz-Russian dictionary] (Sovetskaja enciklopedija, 1985).

Kary-Nijazov, T. N. & Borovkov, A. K. *Uzbeksko-russkij slovar'* [Uzbek-Russian dictionary] (Izdatel'stvo Uzbekistanskogo filiala akademii nauk SSSR, 1941).

Kurpeško-Tannagaševa, N. N. & Apon'kin, F. Ja. *Šorsko-russkij i russko-šorskij slovar'* [Shor-Russian and Russian-Shor dictionary] (Kemerovskoje knižnoje izdatel'stvo, 1933).

Lessing, F. *Mongolian-English dictionary* (University of California Press, 1960).

Luwsandendew, A & Tsedendamba, Ts. *Bol'šoj akademičeskij mongol'sko-russkij slovar'* [Big Russian-Mongol dictionary] *Vols. 1–4* (Academia, 2001-2002).

Malov, S. Je. *Jazyk želtyx ujgurov* [The language of the Yellow Uyghurs] (Izdatel'stvo Akademii nauk Kazaxskoj SSR, 1957).

Mostaert, A. *Dictionnaire Ordos* (Johnson Reprint Company, 1968).

Muniev, B. D. Kalmycko*-russkij slovar'* [Kalmyk-Russian dictionary] (Russkij jazyk, 1977).

Nadžip, E. N. *Ujgursko-russkij slovar'* [Uyghur-Russian dictionary] (Sovetskaja enciklopedija, 1968).

Nugteren, H. *Mongolic Phonology and the Qinghai-Gansu Languages* (Utrecht University, 2011).

Osmanov, M. M. et al. *Tatarsko-russkij slovar'* [Tatar-Russian dictionary] (Sovetskaja enciklopedija, 1966).

Rassadin, V. I. *Tofalarsko-russkij i russko-tofalarskij slovar'* [Tofa-Russian and Russian-Tofa dictionary] (Drofa, 2005).

Róna-Tas, A. & Berta, Á. West Old Turkic: Loanwords in Hungarian. *Turcologica* ***84***, Vol. 1–2 (2011).

Sauranbaev, N. T. *Russko-kazaxskij slovar'* [Russian-Kazakh dictionary] (Gosudarstvennoe izdatel'stvo inostrannyx i nacional'nyx slovarej, 1954).

Sevortjan, E.V. *Etimologičeskij slovar' tjurkskix jazykov. Obščetjurkskije i mežtjurkskije osnovy na glasnyje* [An Etymological dictionary of the Turkic languages. Common vowel-initial stems] (Nauka, 1974).

Sevortjan, E. V. Etimologičeskij slovar' tjurkskix jazykov. Obščetjurkskije i mežtjurkskije osnovy na bukvu B [An Etymological dictionary of the Turkic languages. Common b-initial stems] (Nauka, 1978).

Sevortjan, E. V. *Etimologičeskij slovar' tjurkskix jazykov. Obščetjurkskije i mežtjurkskije osnovy na bukvy V, G i D* [An Etymological dictionary of the Turkic languages. Common *v*-, *g-* and *d-*initial stems] (Nauka, 1980).

Sleptsov, P. A. *Jakutsko-russkij slovar'* [Yakut-Russian dictionary] (Sovetskaja enciklopedija, 1972).

de Smedt, A., & Mostaert, A. *Dictionnaire monguor-francais* (Imprimerie de l'Université Catholique, 1933).

Stachowski, M. *Dolganischer Wortschatz* (Uniwersytet Jagielloński, 1993).

Subrakova, O. V. *Xakassko-russkij slovar'* [Khakas-Russian dictionary] (Nauka, 2006).

Taǧiyev, M. T. et al. *Azərbaycanca- Rusca lüğət* [Azerbaijani-Russian dictionary] (2006).

Tenišev, E. R. *Stroj salarskogo jazyka* [The system of Salar] (Nauka, 1976).

Tenishev, E. R. et al. *Sravnitel'no-istoričeskaja grammatika tjurkskix jazykov. Leksika* [A historical comparative grammar of the Turkic languages. Lexicon] *2nd edn.* (Nauka, 2001).

Tenišev, E. R. *Tuvinsko-russkij slovar'* [Tuvan-Russian dictionary] (Sovetskaja enciklopedija, 1968).

Tenišev, E. R. & Sujunčev, X. I. *Karačajevo-balkarsko-russkij slovar'* [Karachay-Balkar-Russian dictionary] (Russkij jazyk, 1989).

Tumurdej, G. & Tsybenov, B. D. *Dagursko-russkij slovar'* [Dagur-Russian dictionary] (Izdatel'stvo BNTs SO RAN, 2014).

Uraksin, Z. G. *Baškirsko-russkij slovar'* [Bashkir-Russian dictionary] (Digora, Russkij jazyk, 1996).

Useinov, S. M. *Russko-krymskotatarskij, krymskotatarsko-russkij slovar'* [Russian-Crimean Tatar, Crimean Tatar-Russian dictionary] (Tezis, 2007).

Verbitskij, V. I. *Slovar' altajskogo i aladagskogo narečij tjurkskogo jazyka* (Izdanie Pravoslavnogo Missionerskogo Obščestva, 1884).

**Additional References SI 23b Mongolic**

Bawden, Ch. *Mongolian-English dictionary* (Kegan Paul International, 1997).

Bolčuluu, et al. *Yuɣur kelen-ü üges / Dōngbù Yùgùyŭ cíhuì* [Vocabulary of Eastern Yugur] (1984, 1985).

BAMRS. Bolshoy akademicheskiy mongolsko-russkiy slovar [Big academic Mongolian-Russian dictionary] (2001)

Čenggeltei, et al. Mongɣor kele ba Mongɣol kele / Tǔzúyǔ hé Mĕnggŭyŭ [Monguor and Mongolian] (Nèi Měnggǔ rénmín chūbǎnshè, 1988 [1991]).

Chen, Y. *Dongxiang zu zizhixian Naleisi xiaoxue shuangyu jiaoxue shiyan ban shiyong jiaocai* [Textbook for bilingual teaching experimental class of Narisi Primary School in the Dongxiang Autonomous County] (Manuscript, 2002).

Chen Z. et al. *Folktales of China’s Minhe Mangghuer* (LINCOM Publishers, 2005).

Cheremisov, K. M. *Buryat-mongolsko-russkiy slovar’* [Buryat-Mongol-Russian dictionary] (Gos. izd-vo-innostrannyx i nacionalnyx slovarej, 1951).

Damdinov D.G. & Sundueva E.V. Khamnigansko-Russkiy slovar [Khamnigan-Russian dictionary] (Irkutsk, 2015).

Dpal-ldan-bkra-shis, et al. Language Materials of China’s Monguor Minority: Huzhu Mongghul and Minhe Mangghuer. *Sino-Platonic Papers* **69**, (University of Pennsylvania, 1996).

Enkhbat et al. *Daɣur kelen-ü üges / Dáwò’ĕryŭ cíhuì* [Vocabulary of Dagur] (Neimenggu renmin chubanshe, 1984).

Godziński, S. *Język średniomongolski: słowotwórstwo, odmiana wyrazów, składnia* (Wydawnictwa Uniwersytetu Warszawskiego, 1985).

Haenisch, E. *Wörterbuch zu Manghol un niuca tobca'an (Yüan- chcao pi-shi), Geheime Geschichte der Mongolen* (Otto Harrassowitz, 1939).

Iwamura, S. *The Zirni Manuscript. A Persian-Mongolian Glossary and Grammar* (Kyoto University, 1961).

Junast [Zhaonasitu] *Dongbu Yuguyu Jianzhi* [Introduction to the Eastern Yugur language] (Minzu chubanshe, 1981).

Junast [Zhaonasitu] Tŭzúyŭ jiănzhì [Concise grammar of Monguor] (Minzu chubanshe, 1981).

Junast [Zhaonasitu], & Lĭ, K. *Tŭzúyŭ Mínhé fāngyán gàishù* [General overview of the Mínhé dialect of Monguor]. *Mínzú Yŭwén Yánjiū Wénjí*, 458-487 (1982).

Kalużyński, S. Dagurisches Wörterverzeichnis nach F. V. Muromskis handschriftlichen Sprachaufzeichnungen. *Rocznik Orientalistyczny* **33/1**, 103-44, **33/2,** 109-43 (1970).

Kane, D. *The Kitan Language and Script* (Brill, 2009).

Khasbaatar, et al. *Mong*ɣ*or kelen-ü üges / Tŭzúyŭ cíhuì* [Vocabulary of Monguor] (1985 [1986]).

*Langjun 郎君. Da Jin huang di dutong jinglüe langjun xingji.大金皇弟都統經略郎君行記.* [Record of the Journey of the Younger Brother of the Emperor of the Great Jin Dynasty] (Engraved on a stele, 1134).

Lefort, J. *Contacts de langues dans le Nord-Ouest de la Chine: Le cas du Dongxiang* [Contact language in North-West Gansu: The case of Dongxiang] (École des Hautes Études en Sciences Sociales, Doctoral dissertation, 2012).

Lessing, F. *Mongolian-English Dictionary* (University of California Press, 1960).

Lĭ, K. *Monghul Qidar Merlong / Tŭ Hàn Cídiăn* [Monguor-Chinese dictionary] (Qinghai Renmin, 1988).

Tuotuo *Liaoshi* [Official history of the Liao dynasty] (Zhonghua Shuju, 1974).

Ligeti, L. Un vocabulaire d’Istanboul. AOH **XIV**, 3-99 (1962).

Ligeti, L. O mongol’skix i tyurkskix yazykax i dialektax Afganistana. Acta

Orientalia Hungarica **4**, 93–117 (1955).

Ligeti, L. Le lexique moghol de R. Leech. Acta Orientalia Hungarica **4**, 119–58 (1955).

Ma Peiting, Z. *保安语汉语词典 bǎo'ān yǔ hànyǔ cídiǎn* [A Bonan-Chinese Diectionary] (民族出版社 Mínzú chūbǎn shè, 2016).

Ma, G. & Chen, Y. *Dōngxiāngyǔ hànyǔ cídiǎn* (东乡语汉语词典) [Dongxiang-Chinese language dictionary] (Gansu minzu chubanshe, 2001).

*Měnghàn cídiǎn* *蒙汉词典*[Mongolian-Chinese dictionary] (Nèiměnggǔ dàxué chūbǎnshè, 1999).

Muniev, B. D. *Kalmytsko-russkij slovar’* [Kalmyk-Russian dictionary] (Russkij jazyk, 1977).

Naixiong, Ch. (Ed.). *保安语词汇 Bǎo'ān yǔ cíhuì [*Vocabulary of the Bonan Language]. *蒙古语族语言方言研究丛书 Ménggǔ yǔzú yǔyán fāngyán yánjiū cóngshū* [Mongolian Language Dialect Research Series] (Nèiménggǔ rénmín chūbǎn shè, 1986).

Nugteren, H. *Mongolic phonology and the Qinghai-Gansu languages* (LOT. Leiden University Ph.D. dissertation, 2011).

Potanin, G. N. *Tangutsko-tibetskaja okraina Kitaja i central'naja Mongolija* (Imperatorskoe Russkoje Geograficheskoe Obshchestvo, 1893).

Ramstedt, G. J. Mogholica. Beiträge zur Kenntnis der Moghol-Sprache in Afghanistan. *Journal de la Société finno-ougrienne* **23:4, I-IV**, 1-60 (1906).

Ramstedt, G. J. *Kalmückisches Wöerterbuch* (Suomalais-Ugrilainen Seura, 1935).

Róna-Tas, Á. Khitan studies I. The graphs of the Khitan Small Script 1. General Remarks, Dotted Graphs, Numerals. *Acta Orientalia Academiae Scientiarum Hungaricae* **69(2)**, 117-138 (2016).

Shagdarov, L. D. and Kazanceva, A. M. O yazyke mogolov Afganistana (po materialam Sh. Iwamura i X. F. Shurmana*.* *O zarubezhnyx mongolovednyx issledovaniyax po yazyku* (Buryatskoe knizhnoe izdatel’stvo, 1968).

Saitō, Y. *The Mongolian Words in the Muqaddimat al-Adab: Romanized Text and Word Index* (The Japan Society for the Promotion of Science, 2008).

Sečenčogt Kāngjiāyŭ yánjiū [Kangjia language research] (Yuandong chubanshe, 1999).

Sevortyan E.V. *Etimologicheskiy slovar tyurkskih yazykov* [Etymological Dictionary of Turkic languages] (Moscow, 1978).

Slater, K. W. *A Grammar of Mangghuer: A Mongolic Language of China’s Qinghai-Gansu Sprachbund* (Routledge, 2003).

Smedt, A. de, & Mostaert, A. Le dialecte Monguor parlé par les Mongols du Kansou occidental. IIIe partie: Dictionnaire monguor-français (Imprimerie de l'Université Catholique, 1933).

Starostin, G. *Tower of Babel: an etymological database project.* http://starling. rinet.ru

(2008).

Starostin, S. A., Dybo, A. & Mudrak, O. *Etymological Dictionary of the Altaic Languages* (Brill, 2003).

Todaeva, B. Kh. *Mongorskij jazyk. Issledovanie, teksty, slovar’* (Nauka, 1973).

Todayeva, B. Kh. *Dagurskij jazyk* [Dagur] (Nauka, 1986).

Tumurdei, G. & Cybenov, B. D. *Kratkij dagursko-russkij slovar’* [Brief Dagur-Russian dictionary] (BNC SO RAN, 2014).

Weiers, M. Das Moghol-Vokabular von W. R. H. Merk (Otto Harrassowitz, 1971).

Weiers, M. Die Sprache der Moghol der Provinz Herat in Afghanistan. Sprachmaterial, Grammatik, Wortliste*. Abhandlungen der Rheinisch- Westfahlischen Akademie der Wissenschaften* ***49*** (1972).

Weiers, M. *Schriftliche Quellen in Mogoli* (Westdeutscher Verlag, 1975).

Yingzhe, W. & Janhunen, J. *New Materials on the Khitan Small script* (Global Oriental, 2010).

*Xuwang muzhi* [Epitath of Prince Xu] (Epitaph, 1109).

Zhòng, S. *Dáwò’ĕryŭ jiănzhì* [Concise grammar of Dagur] (1982).

**Abbreviations for languages**

Ama. Amami

Az. Azerbaijani

Bao. Bao'an

Bash. Bashkir

Bur. Buriat

CB Chwungpwuk (Ch’ungbuk)

CN Chwungcengnamto (Ch’ungchǒngnamdo)

Chu. Chuvash

Dag. Dagur

Dlg. Dolgan

Dong. Dongxiang

EYugh. Eastern Yughur

Even Even

Evk. Evenki

Gag. Gagauz

HB Hamkyengpukto (Hamgyŏngbukdo)

HN Hamkyengnamto (Hamgyŏngnamdo)

J Japanese

JB Cenpwuk (Chǒnbuk)

JJ Ceycwu (Cheju)

JN Cennam (Chǒnnam)

Jur. Jurchen

Kalm. Kalmuk

KBalk. Karachay-Balkar

Krm. Karaim

Kkp. Karakalpak

Karakh. Karakhanide

Kaz. Kazakh

Khak. Khakas

Khal. Khalkha

Khalaj Khalaj

Kir. Kirgiz

K Korean

KB Kyengpwuk (Kyǒngbuk)

KG Kyengki (Kyǒnggi)

KN Kyengnam (Kyǒngnam)

KS Kyengsang (Kyǒngsang)

KW Kangwento (Kangwǒndo)

Kum. Kumyk

Ma. Manchu

MK Middle Korean

MMo. Middle Mongolian

Miy. Miyako

Mogh. Moghol

Mgr. Monguor

MT Middle Turkic

Na. Nanai

Neg. Negidal

Nog. Nogai

Oki. Okinawa

Olcha Olcha

OJ Old Japanese

OT Old Turkic

Ord. Ordos

Oroch Oroch

PB Pyenganpukto (P’yŏnganbukdo)

pJ proto-Japonic

pK proto-Koreanic

pMo proto-Mongolic

PN Pyengannamto (P’yŏngannamdo)

pTEA proto-Transeurasian

pTg proto-Tungusic

pTk proto-Turkic

Shor Shor

Sibe Sibe

Sol Solon

Tat. Tatar

Tofa. Tofalar

Tk. Turkish

Tkm. Turkmen

Tuva Tuva

Ud. Udehe

Uigh. Uighur

Uz. Uzbek

WMo. Written Mongolian

Yae. Yaeyama

Yak. Yakut

Yon. Yonaguni

**Abbreviations for linguistic forms**

COLL collective

INS instrumental

NMLZ nominalizer/deverbal noun suffix
