# Supplementary material for: Triangulation supports agricultural spread of the Transeurasian languages
Source: Nature. 2021 Nov 10;599(7886):616–21. doi: 10.1038/s41586-021-04108-8 (PMC8612925; doi:10.1038/s41586-021-04108-8)
Supplement: Supplementary file 4 — This zipped file contains Supplementary Data Files 7–11; see Supplementary Information file for full descriptions. [file 41586_2021_4108_MOESM4_ESM.zip › 2021-02-02920E-s4/27_Eurasia3angle_synthesis_SI 7_qualitative analysis_REV30.08.pdf]

Supplementary Information 7

Qualitative Analysis of the Archaeological Databases

§1. Methods

We scored 172 cultural traits for a total of 255 Neolithic-Bronze Age archaeological sites/phases from the West Liao river basin (33), the Amur (Jilin, Heilongjiang and Liaoning) (32), the Primorye (4), the Liaodong peninsula (37), the eastern steppes (1), the Shandong peninsula (4), the Yellow River basin (2), the Korean peninsula (57) and the Japanese Islands (85). Sites with several major cultural phases were scored separately. The sites date from 12,900-1700 BP and include the Early Neolithic to Bronze Age in northeast China, the Middle Neolithic Zaisanovka culture in the Primorye, the Middle-Late Neolithic Chulmun and Bronze Age Mumun cultures in Korea, and the Bronze Age Yayoi culture in western Japan. A few of the Japanese sites also contain features from the Neolithic Final Jōmon (n=5) and the following Kofun era (n=1). Categories of cultural traits scored comprised ceramics (70), stone tools (38), buildings and houses (9), plant and animal remains (26), shell and bone artefacts (17), and burials (12). All features were scored as present (1) or absent (0) following published site reports or other literature (SI 6). In addition to the database of archaeological features, we compiled a list of the earliest cultivated crops from each region of Northeast Asia directly dated by radiocarbon from published sources and from the radiocarbon database of the National Museum of Japanese History (https://www.rekihaku.ac.jp/up-cgi/login.pl?p=param/esrd/db_param) (SI 9). This list comprises a total of 268 samples with (China: 82; Primorye: 12; Korea: 31; Japan (excluding the Ryukyus): 119; Ryukyu Islands: 24). Radiocarbon dates on rice from the Nabatake site (Saga) were omitted since several of the results from that site published in the early 1980s appear unreliable. Our two databases were supplemented by published datasets for faunal remains[1,2], dolmens[3], and spindle whorls[4].

For the database of archaeological features (SI 6), we scored a wide range of artefact categories available across the Northeast Asia region. The features scored comprised those considered likely to be connected to agricultural dispersals in the region. At the same time, given different histories of archaeology and preservation contexts across Northeast Asia, scoring was designed to be as broad and inclusive as possible. In archaeology, ceramics are generally regarded as one possible indicator of population movement. In certain areas of Northeast Asia, previous research has examined links between ceramic typologies and migration in the Neolithic and Bronze Age[5,6,7,8], but no comprehensive regional analysis has yet been attempted. Pottery is also a key functional aspect of the transition from hunter-gatherer to farming in Northeast Asia as new ceramic forms including tripods and steamers were adopted with agriculture[9]. Ceramics were not scored on established typologies but by using basic decorative techniques and vessel shapes as a way to facilitate inter-regional comparisons. For example, steamers were scored as one feature even though as many as six different types have been identified in Chinese Neolithic sites[9].

Stone tools are linked to farming in Northeast Asia through a distinctive series of tools including harvesting knives and sickles[8,9]. Though less diagnostic, certain types of stone axes and hoes are also thought to be related to cultivation[10,11]. The stone tool category scored here includes jade and obsidian, raw materials both known to be widely distributed in Northeast Asia.

The category of buildings includes common domestic architecture in prehistoric Northeast Asia such as pit and surface dwellings, post holes and hearths. Public architecture, fortifications and ditches were also scored, reflecting the growing complexity of Neolithic and Bronze Age settlements in the region.

Twelve plants and 14 animals were scored to obtain an overall picture of the subsistence economy of the scored sites. These included 10 wild species and 16 thought to have been domesticated.

Burials are a way to understand changing social and ideological contexts as well as cultural interactions during the Neolithic and Bronze Age in Northeast Asia[12]. The interpretation of mortuary customs can be complex[13,14,15], but here we scored the main types of burial architecture from Northeast Asia during these periods, with the exception of adult burial jars which are not found outside western Japan. One burial category, dolmens, has been much discussed in terms of possible links to migrations, though controversies remain[3,16].

In Korea and Japan, full reports which typically illustrate every artefact were available for most sites. In China, we also relied on shorter preliminary reports published in academic journals. Where reports provided no further details, features were scored as broad categories. For instance, some reports would just mention the presence of 'microliths' while others would list specific categories of microlithic tools. When no details of ceramic manufacturing techniques were provided in published reports, we scored 'handmade' and 'coarse fabric' as default conditions. Important farming-related features not scored in our database include paddy and other field remains and wooden agricultural tools. These features are common in Bronze Age Korea and Japan, but not found in Northeast China and the Primorye.

The definition and translation of archaeological features, especially ceramic types, has been a controversial issue in East Asia. In China, the same terms may be used in different ways in different parts of the country[17]. Our database gives provisional English translations for the scored features together with the original terminology from the source materials (SI 6).

§2. Archaeological periodisation

Northeast Asia has very early pottery dating back to the terminal Pleistocene[18], and Holocene societies in the region are characterised by high levels of sedentism and material complexity. The term 'Neolithic' is widely applied for such societies in the region even in the absence of cereal farming[19,20,21]. A growing archaeobotanical record shows that several plants, including soybeans,

adzuki beans and barnyard millet, were cultivated in Northeast Asia prior to the spread of the cereal crops discussed here[22]. Bronze was introduced into western China in the late third millennium BC[23]. By the second millennium BC, bronze was widely used on the steppes and in the Central Plains area of the Yellow River[24]. Bronze also spread into Northeast Asia, initially from the steppe region, and broader social changes influenced by new patterns of trans-Eurasian interaction make it appropriate to label the Mumun (1300 – 400 BC) and Yayoi (1000 BC – AD 250) cultures of Korea and Japan, respectively, as Bronze Age. We also use this term for the early metal cultures of the Primorye often classified as 'Palaeometal' by Russian archaeologists.[25]

§3. Neolithic and Bronze Age population dispersals in Northeast Asia: archaeological interpretation

The West Liao river area was one of several apparently independent centres of millet domestication in northern China[26]. Soybeans, adzuki beans and other plants such as *Perilla* sp. were also cultivated in several regions of Northeast Asia[22,27], but the cereal plants discussed in this paper spread from the West Liao and other agricultural centres. This raises the question of the role of population migration versus cultural diffusion, a long-standing debate in archaeology. In Northeast Asia, colonial legacies have made ancient population migrations especially controversial[28,29], and it is sometimes argued that farming spread primarily through diffusion rather than migration[30]. A common assumption in Northeast Asian archaeology is that agriculture could be added to existing subsistence systems with minimal social disturbance through the transmission of new 'information' about farming[31,32]. However, such assumptions play down the extent to which cultural repertoires consist of linked complex behaviours transmitted through social learning at a population level[33]. This has further implications for how social identity is reproduced in material culture[34]. Complex cultural systems like agriculture require 'direct and durable experience' for transmission, implying either the migration of colonising groups or stable, long-term interaction between native foragers and incoming farmers[35]. The latter is often assumed in Northeast Asia but, with some exceptions[36], archaeological research typically looks at technical *variants* of material traits rather than the whole system reproducing those traits. For instance, variation in the direction of burnishing on the necks of pottery vessels between southern Korea and northern Kyushu has been interpreted as a direct reflection of cultural ancestry and even used to estimate the number of peninsular migrants into Bronze Age Japan[37]. In this example, later research has shown greater variety in burnishing direction than originally understood and has also suggested that burnishing direction may be influenced by functional considerations related to vessel size and shape[5]. In contrast to such approaches, here our main interest is to identify ancestor-descendant relationships. Very few previous studies have attempted to analyse the material remains of long-distance farming dispersals across Northeast Asia[6,11], and the present study represents the first attempt to analyse this process in a quantitative as well as a qualitative fashion.

Agricultural dispersals in Northeast Asia involved several levels of ecological and behavioural complexity. While the high productivity of wet rice cultivation may have encouraged population 'packing' rather than migration, millet farming tended to spread outwards by migration albeit producing low-density settlement patterns[38]. From the Late Neolithic, however, the spread of rice together with West Asian barley and wheat changed these earlier population dynamics leading to Bronze Age maritime farming expansions around the Yellow Sea and on to Japan. Bronze Age Korea and Japan both have evidence for the simultaneous diffusion of both artificially irrigated paddies and rain fed (so-called 'dry') fields[39]. Crops were moved beyond previous ecological boundaries and there was a significant expansion in arable land[40].

§3.1. Domesticated plants and animals

One of five apparently separate centres of the early cultivation of foxtail (*Setaria italica*) and broomcorn (*Panicum miliaceum*) millet was the West Liao basin where millets were being grown by 6000 BC[26]. The domestication of these crops likely reflects a slow process[41,42]. In our data, foxtail millet was found at slightly more sites (17%) than broomcorn (14%). Millet cultivation spread north and east to the Primorye and the Korean peninsula by the fourth millennium BC[43]. By around 4000 BC, rice began to spread north from the Yangtze basin and was combined with millet in numerous Middle Neolithic sites in northern China[26,44]. Millets also moved south at this time and were combined with rice at Middle Yangtze sites such as Chengtoushan[45]. Wheat (*Triticum aestivum*) was present in Shandong by around 2600 BC[46], and barley reached central/eastern China by the first millennium BC[47]. In our data, wheat and barley are not found in the West Liao or Primorye regions. Wheat may have been first introduced to the central Yellow river zone of China as an elite crop[46]. The combination of millets with rice, barley and wheat formed a farming package which expanded very quickly across Bronze Age Korea and Japan.

Rice is not found in the Liao river basin or the Russian Far East during the prehistoric period. The *Xin Tang shu* mentions rice from Nosŏng (Ch. Lucheng) at the time of the Parhae kingdom (AD 698-926), probably referring to a place in eastern Jilin. Archaeobotanical analyses of Parhae sites have produced a range of crops including millet (foxtail, broomcorn and barnyard), barley, wheat, soybeans and adzuki beans but no rice[48].

Cannabis was only reported at 5 sites in our sample but is known from other sites in Northeast Asia where it appears to have been cultivated very early. A marked increase in cannabis remains from many regions of Northeast Asia ca. 3000-2000 BC has been linked with new Bronze Age patterns of cultural interaction across Eurasia, possibly involving ritual use of the plant as a drug[49]. The pattern of cannabis finds from Japan is consistent with such an interpretation.

Our data show that the consumption of acorns and walnuts remained common in farming societies in the Primorye, Korea and Japan, but was rare in northeast China. This may reflect the

scarcity of systematic flotation in northeast China. Previous research has noted a decline in nut use associated with the development of rice agriculture in the lower Yangtze[50]. In the Amur basin, Korea and Japan, nuts were widely exploited into ethnohistoric times[51]. In Japan, Yayoi nut processing is usually seen as an influence from Jōmon culture, or else as evidence that early farming was unstable, but, as with fishing, evidence for specialised production needs to be considered. At the Nagano-konishida site (Fukuoka), as many as 15 water reservoirs for processing nuts were discovered[52], suggesting more intensive production than typical for Jōmon villages[53].

Domesticated animals played an important role in the spread of the Neolithic in western Eurasia. In Europe, dairying was a key factor in expansions north of the Mediterranean climatic zone[54] and was present in Mongolia by around 3000 BC[55]. In Northeast Asia, however, uncertainties remain over the role of domesticated animals in Neolithic/Bronze Age population dispersals.

Although it is likely that pigs were being raised in northeast China by at least the Middle Neolithic[56], the history of pig domestication in Northeast Asia is complicated by introgression with wild boar populations and is poorly understood[57]. Domesticated pigs were reported at 14% of sites in our database but not all of the reports provided morphological or other criteria to support that identification. The earliest domesticated pigs in our data are from Xinglongwa in the West Liao basin (c. 8400-7200 BP)[58]. Pigs are reported from the Primorye by the Bronze Age[59,60]. In line with earlier observations[61], we find no evidence for domesticated pigs from Neolithic or Bronze Age Korea. Pigs seem to have been introduced to Japan with cereal farming in the early first millennium BC[57], implying that they were also present in Korea by at least that time. All pig identifications from Japan reported in SI 6 were based on morphological criteria (brachycephalisation, brevicollis, frequencies of alveolar pyorrhea and 3rd molar size) developed by T. Nishimoto in the 1990s[62,63]. While Nishimoto argued that pigs were introduced to Japan with cereal farming, other zooarchaeologists have proposed that management of some wild boar populations had already begun in the preceding Jōmon period, perhaps associated with legume cultivation[64].

Recent studies have argued that the chicken was rare or absent in Neolithic north China to where it was introduced in the Bronze Age or later from south China and perhaps also from West Asia[65,66,67,68,69]. In our data, chickens are only found at 9 sites, 7 of which are from Japan. The reported chicken remains from the Neolithic Haminmangha site (c. 6000-5000 BP) require further confirmation. Chickens first appear in Korea and Japan around 2000 years ago[70], but are rare in Japan until the medieval period[71]. Chickens are rarely found in the Primorye until the modern era[59].

Aurochs (*Bos primigenius*) were exploited in Neolithic northeast China and are known from one of the sites in our sample (Houtaomuga)[72], but were not scored in our database. Domesticated cattle (*Bos taurus*) were introduced from West Asia by the third millennium BC[73]. The earliest domesticated cattle in China are from the Yellow river basin[74]. Only 5 sites in our sample have reported cattle remains from both Neolithic and Bronze Age contexts (Jimo Beiqian, Shandong, 5500-5200 BP; Qixia Yangjiaquan, Shandong, 5500-4600 BP; Huangjiaweizi, Jilin, 4000 BP;

Zhaogezhuang, Shandong, ca. 4000-3500 BP; and Xiajiadian, Inner Mongolia, 4200-3600 BP). At two of these sites (Jimo Beiqian and Zhaogezhuang), the reported cattle remains were bone tools. In the Primorye, cattle appear from the Iron Age [59]. In Japan, the earliest direct radiocarbon dates on cattle are from the Matsuzaki site (Aichi) in Kofun period contexts. (Two earlier first to third centuries AD dates from the same site appear to be unreliable).

Horses were reported from 9 sites/phases (3.5%) in our database with finds from the Bronze Age West Liao, Ordos and Heilongjiang regions. The horses reported from Neolithic Haminmangha and Houtaomuga may be wild or represent later contamination. The first evidence for the use of horses in transportation dates to around 2000 BC with chariot burials of the Sintashta culture. Mounted horseback riding developed by the early first millennium BC. In the eastern steppe region, recent research finds no evidence for the dietary exploitation of horses before the Late Bronze Age, around 1200 BC. After that point, however, horses started to play a key role in diet, ritual and mobility, enabling the first effective use of the dry eastern steppe lowlands[75]. Horses are not known in the Primorye until the Iron Age[59, 60]. The date of the arrival of horses in Korea is unclear. Horses reach Japan from the end of the fourth century AD[76].

Introduced from West Asia, domesticated sheep (*Ovis* sp.) are reported from northern China as early as the fifth millennium BC[77]. In our sample, sheep were reported at 4 Neolithic (Baiyinchanghan, Inner Mongolia; Houtaomuga, Zuojiashan and Huangjiaweizi in Jilin) and 8 Bronze Age (Zhukaigo, Ordos; Upper and Lower Xiajiadian, Yaowangmiao, Zhizhushan and Jinggouzi in Inner Mongolia; Dawangshan, Liaodong; and Zhaogezhuang, Shandong) sites/phases. A single *Ovis* sp. bone came from Houtaomuga Phase III; while no direct date is available, the phase itself dates to 4800-3500 BC. The sheep remains from Bronze Age Zhaogezhuang (Shandong) were oracle bones. Sheep have been reported from at least two medieval sites in the Primorye but are extremely rare in that region[59]. It is unclear when ovicaprids first reached Korea and Japan. The *Nihon shoki* (AD 720) records a gift of 'one camel, two sheep, and one white pheasant' from the Korean kingdom of Paekche to the Japanese court in AD 599[78]. Goats were part of the last Transeurasian farming/language dispersal in the medieval era when Ryukyuan spread with agriculture to the Ryukyu islands[79].

Dogs were surprisingly rare in our data, present at only 16% of sites. This result is consistent with previous findings that dogs are numerically few in Neolithic and Bronze Age sites in Northeast Asia. Previous research has reported 26 Yayoi (Bronze Age) sites with dogs from Japan (forming a total MNI of around 230) but only 5 such sites from Korea[80]. Most Yayoi sites have canid MNIs <5 but a few have much higher numbers with MNIs >50. In our sample, the Haru-no-tsuji, Aoyakamijichi, Kamei and Asahi sites fall into the latter category; at Haru-no-tsuji, dogs comprised more than half of the mammal sample. Morphological differences between Yayoi- and Jōmon-period canids have been used to suggest that domestic dogs were re-introduced to Japan with agriculture in the Bronze Age[81], a scenario supported by genetic evidence, although the Yayoi timing is assumed

rather than proven in such genetic research[82]. Other archaeologists have been less certain about the morphological changes, emphasising instead the cultural change involved in the frequent consumption of dogs in Yayoi society[80].

Common carp (*Cyprinus carpio*) aquaculture, known in China by 6000 BC, spread to Japan by the late first millennium BC in association with wet-rice farming and has been reported at one of the Japanese sites we scored (Asahi)[83]. This aquaculture was presumably also present in the wet-rice cultures of Liaodong and Korea but no evidence is currently available. A decline in fishing at the onset of the Neolithic has been reported for several parts of the world including Japan[84,85,86]. Our data show a number of sites with both fish/shellfish remains and domesticated plants. There are 8 such sites in Liaodong, 3 in south Korea, 2 in the Primorye and 9 in western Japan. In Japan, the introduction of cereal agriculture in the Bronze Age led to new economic niches wherein farmers engaged in much less fishing than previously, but more specialised maritime adaptations developed in coastal areas[71,87].

§3.2. Agricultural tools

Early farming systems in East Asia utilised many similar stone tools to those used for wild plant gathering and processing. However, wet rice farming involved more specialised tools than early millet agriculture[9]. By the fourth millennium BC, specialised farming tools including ploughs, sickles and harvesting knives had started to appear[38]. In Northeast Asia, these farming tools became especially common after the Late Neolithic/Bronze Age integration of millet with rice, barley and wheat. Though wooden farming tools were not scored (see §1 Methods), wooden mortars and pestles become widely distributed from the Late Neolithic. The main farming tools scored in our database were triangular stone ‘ploughs’, reaping knives and sickles. Stone reaping knives were found at 44% of sites but stone sickles were much rarer, recorded at only 7% of sites. Our data support the close relationship between reaping knives and other polished stone tools in Bronze Age Korea and Japan found in previous research[88].

Our data generally support models of an east/west Eurasian dichotomy in Neolithic and Bronze Age culinary traditions[89]. Although wheat and barley are found at 15% and 4% of sites, respectively, there is no evidence for the roasting of these cereals or for bread production. Instead, cereals in Northeast Asia were boiled or steamed. However, our data do show major regional variations in the use of ceramic steamers (see §3.4). Grinding slabs also display considerable regional variation. Common in Neolithic northeast China, grinding slabs are almost always present in both Neolithic and Bronze Age sites in Korea. In Japan, however, grinding slabs are not common in the Bronze Age Yayoi period.

The presence of hunting tools into the Bronze Age in our data is consistent with ethnohistoric information showing the persistence of hunting across Northeast Asia[90]. In the West Liao basin,

microliths remained common in the Neolithic and were still in use by the late Hongshan (5500-5000 BP)[91], but the timing and processes behind their eventual disappearance remain poorly understood[92]. In other parts of Northeast Asia, microliths disappeared by the end of the Pleistocene[93].

Obsidian was the only lithic raw material scored in our data, but was found at 21% of sites. The main obsidian sources in Northeast Asia are limited to the Mount Paektu/Changbai range in north Korea, the Basaltic Plateau of the Primorye, and several sources in Japan[94]. Our data confirm the importance of long-distance obsidian exchange networks in Neolithic Northeast Asia, networks which were not necessarily directly linked to population dispersals.

While metal tools were not scored in our database, it should be noted that bronze was first introduced to northeast China in the second millennium BC. In the Primorye, bronze was found from around 900 BC and iron from 500 BC[95]. In Korea, bronze appeared by at least the eighth century BC. Bronze and iron became widely used in Kyushu from around 400 BC and iron gradually replaced stone tools such as axes, chisels and sickles. By the third century AD, iron tools had spread into the eastern archipelago as far as the southern Tohoku region[96]. The Korean roots of the earliest bronze objects to reach Japan are clear but there is debate over whether the introduction of bronze to the archipelago was associated with renewed large migrations[97].

§3.3. Houses and settlements

The semi-subterranean pit house remained ubiquitous across Northeast Asia from the Neolithic to the Bronze Age and later. 71% of our sites have pit houses as compared to 17% for surface buildings. Only 5 sites (Erdaojingzhi, Dalian Dawangshan II, Aoyakamijichi, Nishikawazu, Yokono) have surface buildings only. Though not reflected in our data, evidence for the diffusion of specific types of domestic architecture has been discussed in the literature. The clearest examples are the Songguk-ri and T'eppyong'ni type pit houses which diffused from southern Korea to north Kyushu[5,8].

Walls, ditches and other defensive architecture such as watch towers spread widely across Northeast Asia in the Bronze Age. There is little evidence of inter-group violence associated with millet dispersals of the Early to Middle Neolithic. Skeletal trauma at the Boisman site in the Primorye appears to show violent conflict in a hunter-gatherer context[98]. By contrast, the integration of millet with rice, barley and wheat led to the widespread occurrence of moated, defensive villages, weapons, and human skeletons with violent trauma in Late Neolithic and Bronze Age contexts[99,100,101].

§3.4. Ceramics and Food Preparation

Fig. SI 7.1 shows the overall frequency of the 25 scored ceramic decorations. Not surprisingly, the most common were generic techniques such as incised wares (present in 32% of sites), diagonal designs (25%), impressed and applique wares (20%). Despite previous claims that cordmarked pottery is diagnostic of early rice cultivation[102], cordmarked and string/cord designs were rare in our sample.

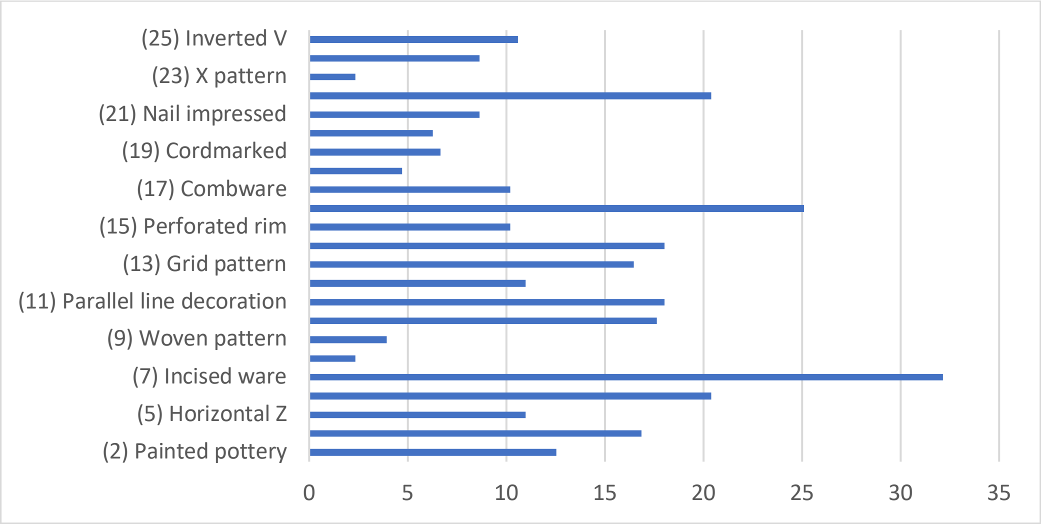

Fig. SI 7.1. Frequency (%) of the 25 ceramic decorative techniques across our data in SI 6. Numbers in parenthesis refer to the features scored in SI 6.

Many of the ceramic decorations show similar relative frequencies across the regions scored. Over time there is a clear toward reduced levels of decoration (Fig. SI 7.2). Plain (undecorated) pottery (which was not scored in our sample) is especially common in Bronze Age Korea and Japan; in fact none of the pottery from Japan has any of the decorative features scored here.

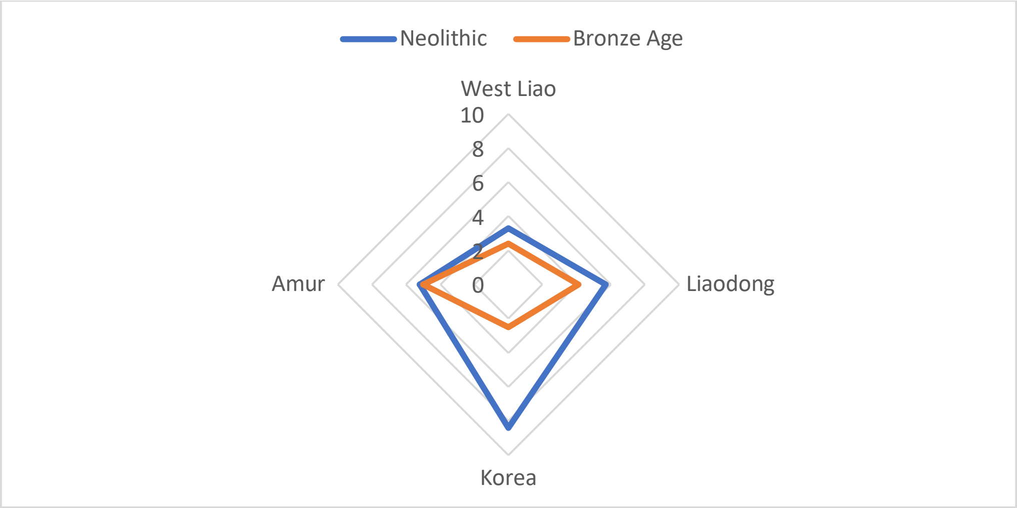

Fig. SI 7.2. Decorative design indices from Neolithic and Bronze Age West Liao, Liaodong, the Korean peninsula, and the Amur (= Liaoning excluding the Liaodong peninsula, Jilin and Heilongjiang). The index was calculated as total number of scored decorative features ÷ number of sites for each region. The figure shows a clear decrease in ceramic decoration in the Bronze Age, especially in Korea.

In terms of vessel shapes, Late Neolithic and Bronze Age China is characterised by a high diversity of forms[103]. An increase in the diversity of ceramic vessel forms is also found in Japan from the third millennium BC, perhaps a result of continental influences. Across East Asia, cereals were

boiled or (later) steamed, a tradition which predates agriculture[89]. In northeast China, tripods appeared from the Lower Xiajiadian phase[30]. Tripods and steamers were not, however, found in the Primorye. In South Korea, tripods and flat-based perforated steamers do not appear until the Proto-Three Kingdoms period (first to third centuries AD). In Japan, steamers were introduced from the Korean peninsula in the fifth century AD, probably from the kingdom of Paekche[104]. Steamers were used with clay ovens (*kamado*) but did not become widely used and most archaeologists assume that cereals were usually boiled until the contemporary era.

Many parts of Eurasia saw the spread of new food processing technologies such as dairying and beverage fermentation during the Neolithic and Bronze Age, but the evidence for such techniques in Northeast Asia is poor. Milking is found in Mongolia by around 3000 BC[55], but there is currently no evidence for milking in Neolithic/Bronze Age Northeast Asia. The use of dairy products is known from historical records in Shandong, Korea and Japan in the sixth to eighth centuries AD but seems to have been mainly limited to elites[105]. Beverage fermentation using rice, honey and fruit has been reported from Jiahu in central China from as early as the seventh millennium BC[106]. In Neolithic northern China, millet was widely used for alcohol production[107]. While it is likely that fermented millet and, later, rice beverages were found in Neolithic Northeast Asia, no direct evidence has so far been reported. In Japan, fruit may have been fermented into wine at Neolithic Jōmon sites such as Sannai Maruyama[108].

§3.5. Ornaments

The most common ornaments in our data were stone beads (24 % of sites), ornamental stone rings (7 %), unspecified bone ornaments (4%), phallic stones (2%), and bone *huang* rings (0.39%). Jade or jadeite was reported at 23% of sites. Ornaments are a poor indicator of farming dispersals in our data. Jade exchange networks existed prior to farming across Northeast Asia. The exchange of jade may have involved some small-scale migrations[109], but in most cases probably reflects trade. In China, jade ornaments remained auspicious symbols of power even in the Bronze Age[110]. A similar role for jade in Bronze Age and later Japan may reflect this Chinese ideology. The widespread distribution of cowrie shells in China also suggests long-range exchange networks not directly associated with farming or language dispersals. At Dadianzi, a site in Chifeng belonging to the Lower Xiajiadian culture (2000-1200 BC), grave goods included 659 cowrie shells which may have come from the Indian Ocean[58]. In Bronze Age Japan, a shell exchange network developed with the Ryukyu Islands but there is no evidence of farming or language dispersals there until around the tenth century AD[111].

§3.6. Textile technology

Our results confirm previous findings that spindle whorls are one of the most diagnostic archaeological traits of Neolithic expansions in Northeast Asia[112]. During the Neolithic, spindle

whorls seem to have been primarily used with previously utilised fibres (hemp, ramie etc). In the Bronze Age, the wool of sheep and goats began to be widely used in Europe and Central Asia[113], but wool was rare in Northeast Asia. A burial at the Xingxingshao site in Jilin, dated to the first millennium BC, produced a cloth woven from hemp together with goat and dog hair[114]. The use of dog hair in weaving amongst northern tribal groups in Northeast Asia is mentioned in Tang sources[115]. In China, silk may have been used from as early as 8500 BP[116]. By the Bronze Age, silk was used for at least elite clothing across Northeast Asia including Japan.

§3.7. Burials

Prehistoric mortuary customs have been an important topic of research across Northeast Asia and burials often provide evidence for shared links between elite cultures in particular[114]. However, our data show few direct links between mortuary customs and population dispersals. While the agricultural societies analysed here all developed elaborate mortuary architecture by at least the Bronze Age, there was nevertheless considerable regional diversity. The earliest millet farming societies in Northeast Asia largely lacked elaborate burials, although the Hongshan culture (4500-3000 BC) is an exception with its platforms and stone cairns. Various ritual activities seem to have been performed around these cairns for long periods, possibly suggesting a cult of ancestors[117]. Cairn burials are rare in Jilin and Heilongjiang where earthen pit graves remained the norm into the Bronze Age. At the Lower Xiajiadian culture (2000-1200 BC) Dadianzi site, a cemetery of over 800 burials mainly consisted of earthen pits in which individuals were laid in wooden coffins, though some tombs contained stone cists. Thirteen tombs at Dadianzi contained bronze vessels of local production imitating Erlitou bronzes from the Central Plains. Biological anthropologists have suggested two groups of different ancestry in this cemetery, one similar to populations from the Yellow river, the other closer to Amur basin groups. The majority of the Erlitou-like bronze vessels were found in burials of the 'Yellow river related' group[58].

There are currently no burial sites known for the Zaisanovka culture associated with the earliest millet farming in the Primorye. The only well-documented cemetery of the Primorye is Boisman-2 (Boisman culture c. 7500-4000 BP) which consists of two groups of earthen pit tombs located under a shell midden[118]. At Zaisanovka 7, a Bronze Age pit burial was also found in a shell midden context[119]. Ancient DNA from a Bronze/Iron Age burial at Pospelovo-1 has recently been reported[120]. In Korea, Neolithic burials are few but show a wide variation including earthen pit burials (sometimes in shell middens), cremation in jar burials buried inside a house (Sangch'on B), or multiple burials covered with stones (Yokchido)[121]. In Japan, burials of the Neolithic Jōmon period were mainly primary single graves in earthen pits containing few or no grave goods, though other types such as secondary jar burials for adults, primary jar burials for infants, cremation, and inhumation inside abandoned houses also occur[122]. In the Late and Final Jōmon phases in northern

Japan, burials become more elaborate[123], possibly under the influence of Bronze Age Eurasia. The following Yayoi period saw the adoption of many mortuary customs into Japan from the Korean peninsula but this process was characterised by considerable complexity and negotiation[16].

Dolmens may be the clearest evidence of a link between farming dispersals and mortuary customs. A dataset published in 1997 reported 66 dolmen sites in Liaoning (mostly on the Liaodong peninsula), 34 in Jilin, at least 1929 on the Korean peninsula, and 123 in Kyushu (Fukuoka, Saga, Nagasaki, Kumamoto and Kagoshima prefectures)[3]. However, the lack of absolute dating for the dolmens of northeast China and northern Korea makes it difficult to validate the common argument that dolmens first appeared in Jilin or Liaoning and then spread to Korea[124,125]. On the Korean peninsula, dolmens appear some time after the beginning of rice farming and could thus be markers of emerging social stratification in Bronze Age society[126]. Dolmens similar to those of southern Korea appear in northwest Kyushu at the beginning of the Yayoi period along with Korean-style polished stone daggers and red burnished pottery as grave goods[127]. Within the Japanese archipelago, dolmens extended only as far as northwest Shikoku. In other areas of Bronze Age Japan, a range of mortuary traditions including jar burials and moated burial precincts soon developed. Large jar burials were primarily limited to northern Kyushu, but the spread of moated burial precincts into the eastern archipelago may reflect population dispersals.[128]

In summary, our data are consistent with previous interpretations of mortuary traditions in Neolithic-Bronze Age Northeast Asia. The diffusion of dolmens from Liaodong to Korea to western Japan may be an example of a mortuary custom which spread in association with agricultural dispersals but further radiocarbon dating is needed to confirm this hypothesis. In other cases, it is harder to identify specific cultural ancestries from burial features alone but there is nevertheless a clear trend for cemeteries to become more marked and elaborate as agriculture developed. To the extent that formal cemeteries developed as a way to make claims to land, we might expect mortuary customs to be under selection for new features that differentiated them from previous customs.

§3.8. Ritual

The wide range of vessel shapes in Neolithic and Bronze Age China is often linked to the provision of food and wine for family ancestors[124,129,130]. In Japan, the performance of rituals for agricultural production and elite ancestors has been interpreted as a continental custom introduced with the ‘Yayoi package’[131]. However, our data show that few of the elaborate ceremonial vessels used in China were found in other regions of Northeast Asia.

Oracle bones were found in 13 sites in our dataset with a wide geographical range from the Ordos to Liaodong to western Japan. None of the Korean sites scored here had oracle bones although the custom is known from at least 15 Iron Age/Proto-Three Kingdoms sites on the peninsula[132]. The oldest oracle bones in East Asia are from Inner Mongolia from the late fourth millennium BC[133]. By

the second half of the third millennium BC, plastromancy became more common in the Late Neolithic Longshan culture. Oracle bones with written inscriptions characterise the Bronze Age Shang state in the Yellow river valley but the custom, usually without writing, spread widely across Northeast Asia. In Japan, oracle bones are known from 54 sites dating from the fourth century BC to the third century AD[132].

§3.9. Demography

Across Eurasia, the increased sedentism of the Neolithic led to population growth[134]. In many areas of Northeast Asia, sedentism developed before farming[135]. As cereal farming became more important, the type of agriculture engaged in by East Asian societies affected their demographic patterns. Wet rice farmers, who had invested time and labour in elaborate paddy fields, tended to stay in one place, absorbing population growth through extra labour. Millet farmers, by contrast, often adopted a more dispersed or expansionary settlement pattern[38]. Within millet farming societies in northern China, community structure was also influenced by different risk buffering strategies resulting from environmental conditions[136].

Neolithic population growth could be subject to fluctuations due to climate, disease, or the low resilience of early farming systems[137,138]. Climate change, the immigration of steppe pastoralists, trade and plague have been suggested as possible causes of a Late Neolithic population decline which was also found in Northeast Asia[139,140,141,142]. Previous research has identified large population increases in the Bronze Age in China[141], Korea[143], and Japan[144].

We collated published demographic estimates for Neolithic and Bronze Age Northeast Asia and plotted them together in Extended Data Fig. 3. The red bars in the figure show the first evidence for millet cultivation based on direct radiocarbon dates. The site numbers for the West Liao basin and Liaoning province were extracted from an analysis of 51,074 archaeological sites in China dating to ca. 8000 – 500 BC[141]. These data were taken from the 25 published volumes of the *Atlas of Chinese Cultural Relics*. Site numbers for the eastern Inner Mongolia Autonomous Region are used as a proxy for the West Liao basin. The site numbers shown in Extended Data Fig. 3 for the West Liao and Liaoning are totals for each time period.

For Korea we used summed probability distributions of radiocarbon dates analysed in a recent study[143]. Building on the high number of radiocarbon dates available in South Korea, that study used 3127 dates from Chulmun and Mumun pit houses. Some 94% of the dates were on charcoal thought to derive from the wooden structures of the houses which were likely re-built every decade or so. Multiple dates from the same house were combined and temporal outliers and samples with a standard error >100 years were removed to leave 2190 dates from 513 sites ranging between 5900 – 2200 cal BP. The generated SPD was tested using methods from Timpson et al. (2014)[145] and Crema et al. (2016)[146].

For the southern Primorye, we used estimates of site numbers provided by Irina Zhushchikhovskaya of the Institute of History, Archaeology and Ethnology of the Peoples of the Far East. These estimates are 55-70 for the pre-millet farming Neolithic Boisman and Rudnaya cultures, around 150 for the Zaisannovka culture, and 200-300 for the Bronze Age Yankovskaya culture. In Fig. 3 we used the higher end of these estimates since archaeological survey work is still scarce in the region and more sites would plausibly be found in future research.

For Japan, we used the population estimates made by Koyama in the 1970s[144]. Koyama collected data on site numbers and size for mainland Japan excluding Hokkaido and Okinawa from the Initial Jōmon to the Yayoi periods. Population was calculated using the relationship between site size and populations as recorded in early historic tax records with a correction factor applied for Jōmon sites. Our graph shows Koyama's estimates for the total population of mainland Japan. Methodologically, Koyama's estimates are perhaps the weakest of the proxies used for Fig. 3. However, later research has supported the general trends identified by Koyama using a range of approaches at various regional scales, including site and pit house numbers as well as radiocarbon dates[146, 147 148, 149,150].

The graphs A1 and C1 in Extended Data Fig. 3 show proxies for demographic change following the initial adoption of millet farming in the West Liao basin and Korea. The West Liao graph in A1 shows changes in pottery found during surface surveys covering a total of 1339 km^2 in the Chifeng and Fuxin areas. Varying quantities of pottery found during these surveys are hypothesised to scale to population density[151]. The graph for Korea in C1 is taken from the same radiocarbon SPD analysis used for the graph in C.

Extended data Fig. 3 is designed to show broad demographic *trends* in Northeast Asia in the Neolithic and Bronze Ages. Despite the use of different proxies in the published literature, the trends identified are comparable, especially the large population increase reconstructed for the Bronze Age in all regions.

§3.9. Qualitative analyses: summary

Our results generally confirm and provide broader context for previous archaeological analyses of Neolithic and Bronze Age population dispersals in Northeast Asia. Our results clearly show that all scored archaeological features did not disperse in unison as monothetic cultures. Several categories of archaeological artefacts were associated with Middle Neolithic millet farming expansions to Korea and the Primorye[43]. In the Bronze Age, a larger inventory of items became associated with agricultural dispersals, including wooden farming tools, stone reaping knives, mortars and pestles, and rice paddy fields. Between Bronze Age Korea and Japan, many more specific material culture parallels have been discussed in the literature[25,6,11,23,39,88,91,112,127].

Based on our archaeological database and previous research, several major differences in the dispersal of farming cultures across Northeast Asia can be noted. The great variety in ceramic—and later bronze—vessel forms found in northeast China did not spread to other regions of Northeast Asia, perhaps because the ritual functions of these vessels were not shared outside China. Overall, ceramics were a poor indicator of population dispersals in our data but the macro approach to ceramics employed here could be further expanded with traditional typological methods, which already suggest clear links between Korea and Japan. Mortuary customs also became more diverse over time and, except for dolmens, none of the specific burial types found in northeast China spread beyond that area. Although a combination of five domesticated cereals (barley, broomcorn millet, foxtail millet, rice and wheat) spread quickly across Northeast Asia from the Bronze Age, the spread of domesticated animals was slower and more variable.

Our qualitative analyses support four types of interaction in Neolithic-Bronze Age Northeast Asia: (1) Long-range interaction spheres conducted through trade and exchange without population-level dispersals. Examples here are jade and obsidian exchange networks; (2) Clear association between population dispersals and cultural traits: cereals are the most direct evidence but this category is also supported by spindle whorls, wooden farming tools and semi-lunar stone reaping knives. On a local level, for well-studied regions such as Korea and Kyushu, we can also propose Songgukri and T'eppyong'ni house forms and ceramics as further examples of the association between farming dispersals and material culture; (3) Less secure but nevertheless suggestive associations include artefacts associated with Middle Neolithic millet dispersals, such as chipped stone hoes and polished stone arrowheads; and (4) Cases where the transformation into an agricultural society engendered comparable responses without direct parallels. The best example here is the use of elaborate yet diverse burial customs to make links to territory in increasingly stratified societies in the Bronze Age.

---

[1] Kōmoto, M. in *A Study on the Environmental Change and Adaptation System in Prehistoric Northeast Asia* (ed Kōmoto, M.) 8-34 (Faculty of Letters, Kumamoto Univ., 2007).

[2] An, S. (ed), *Nongŏbŭi kogohak* (Seoul: Sahoep'yŏngnon, 2013).

[3] Nishitani, T. (ed.) *Higashi Ajia ni okeru shisekibo no sōgōteki kenkyū* (Dept. Archaeology, Kyushu Univ., 1997).

[4] Furusawa, Y. in *A Study on the Environmental Change and Adaptation System in Prehistoric Northeast Asia* (ed Kōmoto, M.) 86-109 (Faculty of Letters, Kumamoto Univ., 2007).

[5] Hashino, S. in *Coexistence and Cultural Transmission in East Asia* (eds Matsumoto, N., Bessho, H. & Tomii, M.) 203-221 (Left Coast Press, 2011).

[6] Miyamoto, K. Archaeological explanation for the diffusion theory of the Japonic and Koreanic languages. *Jpn. J. Archaeol*. **4**, 53-75 (2016).

[7] Kawakami, Y. in *Coexistence and Cultural Transmission in East Asia* (eds Matsumoto, N., Bessho, H. & Tomii, M.) 257-275 (Left Coast Press, 2011).

[8] Mizoguchi, K. *The Archaeology of Japan: From the Earliest Rice Farming Villages to the Rise of the State* (Cambridge Univ. Press, 2013).

[9] Makibayashi, K. The transformation of farming cultural landscapes in the Neolithic Yangtze area. *J. World Prehist.* **27**, 295-307 (2014).

[10] Itakura, Y. in *Coexistence and Cultural Transmission in East Asia* (eds Matsumoto, N., Bessho, H. & Tomii, M.) 181-201 (Left Coast Press, 2011).

[11] Miyamoto, K. The initial spread of early agriculture into Northeast Asia. *Asian Archaeol.* **3**, 1-12 (2014).

[12] Gilaizeau, L. Diplomacy from the grave: interactions between western Japan and the East Asian continent from a burial point of view. *Crossroads* **9**, 45-62 (2014).

[13] Shelach, G. Apples and oranges? A cross-cultural comparison of burial data from northeast China. *J. East Asian Archaeol.* **3**, 53-90 (2001).

[14] Mizoguchi, K. Time and genealogical consciousness of the mortuary practices of the Yayoi period, Japan. *J. East Asian Archaeol.* **3**, 173-197 (2001).

[15] Gilaizeau, L. & Guillon, M. in *Qu'est-ce qu'une sépulture? Humanités et systèmes funéraires de la Préhistoire à nos jours*, (ed Lauwers, M. & Zemour, A.) 293-310 (Antibes: APDCA, 2016).

[16] Nakamura, D. in *Coexistence and Cultural Transmission in East Asia* (eds Matsumoto, N., Bessho, H. & Tomii, M.) 223-256 (Left Coast Press, 2011).

[17] Heine, A.M. *Cultural Geography and Interregional Contacts in Prehistoric Liangshan (Southwest China)*. PhD dissertation, UCLA, 2013.

[18] Iizuka, F. The timing and behavioral context of the Late-Pleistocene adoption of ceramics in greater East and Northeast Asia and the first people (without pottery) in the Americas. *Paleoamerica* **4**, 267-324 (2018).

[19] Gibbs, K. & Jordan, P. A comparative perspective on the 'western' and 'eastern' Neolithics of Eurasia: ceramics, agriculture and sedentism. *Quat. Int.* **419**, 27-35 (2016).

[20] Kuzmin, Y. Two trajectories in the Neolithization of Eurasia: pottery versus agricultural (spatiotemporal patterns). *Radiocarbon* **55**, 539-556 (2013).

[21] Hudson, M.J. in *Routledge Handbook of the Bioarchaeology of Climate and Environmental Change* (ed Schug, G.) 379-395 (Routledge, 2020).

[22] Crawford, G.W. in *Handbook of East and Southeast Asian Archaeology* (eds Habu, J., Lape, P.V. & Olsen, J.W.) 421-435 (Springer, 2018).

[23] Barnes, G.L. *Archaeology of East Asia: The Rise of Civilisation in China, Korea and Japan* (Oxbow, 2015).

[24] Rawson, J. China and the steppe: reception and resistance. *Antiquity* **91**, 375-388 (2017).

[25] Popov, A., Zhushchikhovskaya, I.S. & Nikitin, Y.G. Paleometal epoch in the Primorye (south of the Far East of Russia). *World Archaeol*. **51**, 382-407 (2020).

[26] Stevens, C.J. & Fuller, D.Q. The spread of agriculture in eastern Asia: archaeological bases for hypothetical farming/language dispersals. *Lang. Dyn. Chang.* **7**, 152-186 (2017).

[27] Lee, G-A. Archaeological perspectives on the origins of azuki (*Vigna angularis*). *Holocene* **23**, 453-459 (2012).

[28] Pai, H.I. *Constructing 'Korean' Origins: The Roles of Archaeology, Historiography and Racial Myth in Korean State-Formation Theories* (Harvard Univ. Press, 2000).

[29] Hudson, M.J. Pots not people: ethnicity, culture and identity in postwar Japanese archaeology. *Crit. Anthropol.* **26**, 411-434 (2006).

[30] Jia, P.W.M. *Transition from Foraging to Farming in Northeast China* (Oxford: BAR Publishing, 2007).

[31] Kim, J.S. & Park, J. Millets vs rice: an evaluation of the farming/language dispersal hypothesis in the Korean context. *Evol. Hum. Sci.* **2**, e12 (2020).

[32] Mizogichi, K. Re-thinking the origin of agriculture through the 'beginnings' in the Japanese archipelago. *Jpn. J. Archaeol*. **6**, 95-107.

[33] Boyd, R., Richerson, P.J. & Henrich, J. The cultural niche: why social learning is essential for human adaptation. *Proc. Natl. Acad. Sci USA* **108** (Supp. 2), 10918-10925 (2011).

[34] Bettinger, R.L. in *Cultural Transmission and Archaeology: Issues and Case Studies* (ed O'Brien, M.J.) 1-9 (Society for American Archaeology, 2008).

[35] Ibáñez-Estévez, J.J., Gibaja Bao, J.F., Gassin, B. & Mazzucco, N. in *Times of Neolithic Transition along the Western Mediterranean* (eds García-Puchol, O. & Salazar-García, D.C.) 339-371 (Springer, 2017).

[36] Uchiyama, J., Gillam, J.C., Hosoya, L.A., Lindström, K. & Jordan, P. Investigating Neolithization of cultural landscapes in East Asia: the NEOMAP project. *J. World Prehist*. **27**, 197-223 (2014).

[37] Yane, Y. Chōsen mumon doki kara Yayoi doki e. *Ritsumeikan Daigaku Kōkogaku Ronshū* **1**, 39-64 (1997).

[38] Qin, L. & Fuller D.Q in *Prehistoric Maritime Cultures and Seafaring* (eds Wu, C. & Rolett, B.) 159-191 (Springer, 2019).

[39] Shitara, H. in *Yayoi jidai no kōkogaku 5: shokuryō no kakutoku to seisan* (ed Shitara, H., Fujio, S. & Matsugi, T.) 3-22 (Tokyo: Dōseisha, 2009).

[40] Liu, X., Jones, P.J., Matuzeviciute, G.M., Hunt, H.V., Diane L. Lister, D.L., An, T., Przelomska, N., Kneale, C.J., Zhao, Z. & Jones, M.K. From ecological opportunism to multi-cropping: mapping food globalisation in prehistory. *Quat. Sci. Rev.* **206**, 21-28 (2019).

[41] Shelach-Lavi, G., Teng, M., Goldsmith, Y., Wachtel, I., Stevens, C.J., Marder, O., Wan, X., Wu, X., Tu, D., Shavit, R., Polissar, P., Xu, H. & Fuller, D.Q. Sedentism and plant cultivation in northeast China emerged during affluent conditions. *PLoS ONE* **14**, e0218751 (2019).

[42] Stevens, C.J., Shelach-Lavi, G., Zhang, H., Teng, M. & Fuller, D.Q. A model for the domestication of *Panicum miliaceum* (common, proso or broomcorn millet) in China. *Veg. Hist. Archaeobot.* (2020).

[43] Li, T., Ning, C., Zhushchikhovskaya, I.S., Hudson, M.J. & Robbeets, M. Millet agriculture dispersed from Northeast China to the Russian Far East: integrating archaeology, genetics and linguistics. *Archaeol. Res. Asia* **22**, e100177 (2020).

[44] d'Alpoim Guedes, J., Jin, G. & Bocinsky, R.K. The impact of climate on the spread of rice to north-eastern China: a new look at the data from Shandong province. *PLoS ONE* **10**, e0130430.

[45] Nasu, H., Gu, H., Momohara, A. & Yasuda, Y. Land-use change for rice and foxtail millet cultivation in the Chengtoushan site, central China, reconstructed from weed seed assemblages. *Archaeol. Anthropol. Sci.* **4**, 1-14 (2012).

[46] Long, T. et al. The early history of wheat in China from ^{14}C dating and Bayesian chronological modelling. *Nat. Plants* **4**, 272-279.

[47] Liu, X. et al. Journey to the east: diverse routes and variable flowering times for wheat and barley *en route* to prehistoric China. *PLoS ONE* **12**, e0187405.

[48] Sergusheva, E.A. Cultivated plants of the Bohai population of Primorye according to archaeobotanical data. *Archaeol. Ethnol. Anthropol. Eurasia* **42**, 111-118.

[49] Long, T., Wagner, M., Demske, D., Leipe, C. & Tarasov, P.E. Cannabis in Eurasia: origin of human use and Bronze Age trans-continental connections. *Veget. Hist. Archaeobot.* **26**, 245-258.

[50] Fuller, D.Q. & Qin, L. Declining oaks, increasing artistry, and cultivating rice: the environmental and social context of the emergence of farming in the Lower Yangtze region. *Environ. Archaeol.* **10**, 139-159 (2010).

[51] Hosoya, L.A. Staple or famine food? Ethnographic and archaeological approaches to nut processing in East Asian prehistory. *Archaeol. Anthropol. Sci.* **3**, 7-17 (2011).

[52] Shitara, H. in *Yayoi jidai no kōkogaku 5: shokuryō no kakutoku to seisan* (eds Shitara, H., Fujio, S. & Matsugi, T.) 3-22 (Tokyo: Dōseisha, 2009).

[53] Kawashima, T. Food processing and consumption in the Jōmon. *Quat. Int.* **404**, 16-24 (2016).

[54] Ethier, J. et al. Earliest expansion of animal husbandry beyond the Mediterranean zone in the sixth millennium BC. *Sci. Rep.* **7**, 7146 (2017).

[55] Wilkin, S. et al. Dairy pastoralism sustained eastern Eurasian steppe populations for 5000 years. *Nat. Ecol. Evol.* **4**, 346-355 (2020).

[56] Nelson, S.M. in *Ancestors for the Pigs: Pigs in Prehistory* (ed Nelson, S.M.) 99-107 (Univ. Pennsylvania Museum of Archaeology and Anthropology, 1998).

[57] Price, M. & Hongo, H. The archaeology of pig domestication in Eurasia. *J. Archaeol. Res.* **28**, 557-615 (2020).

[58] Liu, L. & Chen, X. *The Archaeology of China: From the Late Palaeolithic to the Early Bronze Age* (Cambridge Univ. Press, 2012).

[59] Kuzmin, Y.V. Vertebrate animal remains from prehistoric and medieval settlements in Primorye (Russian Far East). *Int. J. Osteoarchaeol*. **7**, 172-180 (1997).

[60] Kuzmin, Y.V. & Rakov, V.A. Environment and prehistoric humans in the Russian Far East and neighbouring East Asia: main patterns of interaction. *Quat. Int*. **237**, 103-108 (2011).

[61] Lee, G.A. in *Handbook of East and Southeast Asian Archaeology* (eds Habu, J., Lape, P. & Olsen, J.) 451-481 (Springer, 2017).

[62] Nishimoto, T. Pigs in the Yayoi period. *Bull. Nat. Mus. Japan. Hist*. **36**, 175-193 (in Japanese with English summary).

[63] Nishimoto, T. The physical character of the pig in the Yayoi period. *Bull. Nat. Mus. Japan. Hist.* **50**, 49-69 (in Japanese with English summary).

[64] Uchiyama, J. in *Jōmon jidai no kōkogaku 4: hito to dōbutsu no kakawariai* (ed Kosugi, Y., Nishida, Y., Mizunoe, K., Taniguchi, Y. & Yano, K.) 167-179 (Tokyo: Dōseisha, 2010).

[65] Eda, M., Lu, P., Kikuchi, H., Li, Z., Li, F. & Yuan, J. Reevaluation of early Holocene chicken domestication in northern China. *J. Archaeol. Sci*. **67**, 25-31 (2016).

[66] Peters, J., Lebrasseur, O., Deng, H. & Larson, G. Holocene cultural history of Red jungle fowl (*Gallus gallus*) and its domestic descendant in East Asia. *Quat. Sci. Rev*. **142**, 102-119 (2016).

[67] Peters, J., Lebrasseur, O., Best, J., Miller, H., Fothergill, T., Dobney, K., Thomas, R.M., Maltby, M., Sykes, N., Hanotte, O. & O'Connor, T. Questioning new answers regarding Holocene chicken domestication in China. Proc. Nat. Acad. Sci. USA, **112**(19), e2415 (2015).

[68] Huang, X.H., Wu, Y.J., Miao, Y.W., Peng, M.S., Chen, X., He, D.L., Suwannapoom, C., Du, B.W., Li, X.Y., Weng, Z.X. & Jin, S.H., 2018. Was chicken domesticated in northern China? New evidence from mitochondrial genomes. *Sci. Bull. Fac. Agric. Kyushu Univ*. **63**, 743-746 (2018).

[69] Wang, M.S., Thakur, M., Peng, M.S., Jiang, Y., Frantz, L.A.F., Li, M., Zhang, J.J., Wang, S., Peters, J., Otecko, N.O. & Suwannapoom, C. 863 genomes reveal the origin and domestication of chicken. *Cell Res*. **30**, 693-701 (2020).

[70] Eda, M. Yayoi jidai no niwatori, saikō. *Kikan Kōkogaku* **144**, 43-46 (2018).

[71] Hudson, M.J. Towards a prehistory of the Great Divergence: the Bronze Age roots of Japan's premodern economy. *Doc. Praehist*. **46**, 30-43 (2019).

[72] Cai, D. et al. Ancient DNA reveals evidence of abundant aurochs (Bos primigenius) in Neolithic northeast China. *J. Archaeol. Sci*. **98**, 72-80 (2018).

[73] Lu, P. et al. Zooarchaeological and genetic evidence for the origins of domestic cattle in ancient China. *Asian Perspect.* **56**, 92-120 (2017).

[74] Yu, C. *The Origin of Cattle in China from the Neolithic to the Early Bronze Age* (Oxford: BAR Publishing, 2020).

[75] Taylor, W.T.T. et al. Early pastoral economies and herding transitions in eastern Eurasia. *Sci. Rep.* **10**, 1001 (2020).

[76] Sasaki, K. in *Early Korea-Japan Interactions* (eds Byington, M.E, Sasaki, K. & Bale, M.T.) 271-371 (Korea Institute, Harvard Univ., 2018).

[77] Dodson, J. et al. Oldest directly dated remains of sheep in China. *Sci. Rep*. **4**, 7170 (2014).

[78] Aston, W.G. *Nihongi* (Rutland VT & Tokyo: Tuttle, 1972).

[79] Toizumi, T. Okinawa no jūnikushoku. *Kikan Kōkogaku* **144**, 63-64 (2018).

[80] Uchiyama, S. in *Yayoi jidai no kōkogaku 5: shokuryō no kakutoku to seisan* (ed Shitara, H., Fujio, S. & Matsugi, T.) 117-131 (Tokyo: Dōseisha, 2009).

[81] Nishimoto, T. in *Hito to dōbutsu no Nihonshi 1: dōbutsu no kōkogaku* (ed Nishhimoto, T.) 180-191 (Tokyo: Yoshikawa Kōbunkan, 2008).

[82] Tanabe, Y. Phylogenetic studies of dogs with emphasis on Japanese and Asian breeds. *Proc. Jpn. Acad., Ser. B* **82**, 375-387 (2006).

[83] Nakajima, T. et al. Common carp aquaculture dates back 8000 years in Neolithic China. *Nat. Ecol. Evol.* **3**, 1415-1418 (2019).

[84] Richards, M.P., Schulting, R.J. & Hedges, R.E.M. Sharp shift in diet at onset of Neolithic. *Nature* **425**, 366 (2003).

[85] Schulting, R.J. in *The Oxford Handbook of the Archaeology of Diet* (eds Lee-Thorp, J. & Katzenberg, M.A.), DOI: 10.1093/oxfordhb/9780199694013.013.35 (Oxford Univer. Press, 2018).

[86] Wada, S. in *Yayoi bunka no kenkyū 2: seigyō* (eds Kanaseki, H. & Sahara, M.) 153-161 (Tokyo: Yūzankaku, 1988).

[87] Takase, K. Long-term marine resource use in Hokkaido, northern Japan: new insights into sea mammal hunting and fishing. *World Archaeol*. **51**, 408-428 (2019).

[88] Chon, Y.N. in *Pacific Northeast Asia in Prehistory: Hunter-Fisher-Gatherers, Farmers and Sociopolitical Elites* (eds Aikens, C.M. & Rhee, S.N.) 161-169 (Pullman: Washington State Univ. Press, 1992).

[89] Fuller, D.Q. & Rowlands, M. in *Interweaving Worlds: Systemic Interactions in Eurasia, 7th to the 1st Millennia BC* (eds Wilkinson, T.C., Sherratt, S. & Bennet, J.) 37-60 (Oxford: Oxbow, 2011).

[90] Zgusta, R. *The Peoples of Northeast Asia through Time: Precolonial Ethnic and Cultural Processes Along the Coast Between Hokkaido and the Bering Strait* (Leiden: Brill, 2015).

[91] Wa, Y. in *Pacific Northeast Asia in Prehistory: Hunter-Fisher-Gatherers, Farmers and Sociopolitical Elites* (eds Aikens, C.M. & Rhee, S.N.) 139-156 (Pullman: Washington State Univ. Press, 1992).

[92] Zhang, M. Microblade-based societies in north China at the end of the Ice Age. *Quaternary* **3**, e20 (2020).

[93] Takakura, J. Rethinking the disappearance of microblade technology in the terminal Pleistocene of Hokkaido, northern Japan: looking at archaeological and palaeoenvironmental evidence. *Quaternary* **3**, e21 (2020).

[94] Kuzmin, Y.V. Obsidian as a commodity to investigate human migrations in the Upper Paleolithic, Neolithic, and Paleometal of Northeast Asia. *Quat. Int*. **442**, 5-11 (2017).

[95] Popov, A.N., Zhushchikhovskaya, I.S. & Nikitin, Y.G. Paleometal epoch in the Primorye (south of the Far East of Russia). *World Archaeol*. **51**, 382-407 (2019).

[96] Matsugi, T. in *Burial Mounds in Europe and Japan: Comparative and Contextual Perspectives* (eds Knopf, T., Steihaus, W. & Fukunaga, S.) 173-181 (Oxford: Archaeopress, 2018).

[97] Iwanaga, S. in *Early Korea-Japan Interactions* (eds Byington, M.E, Sasaki, K. & Bale, M.T.) 91-131 (Korea Institute, Harvard Univ., 2018).

[98] Tabarev, A. in *Neolithisation and Modernisation: Landscape History on East Asian Inland Seas* (eds Uchiyama, J., Lindström, K., Zeballos, C. & Nakamura, O.) 131-138 (Kyoto: RIHN, 2009).

[99] Shelach, G., Raphael, K. & Jaffe, Y. Sanzuodian: the structure, function and social significance of the earliest stone fortified sites in China. *Antiquity* **85**, 11-26 (2011).

[100] Sanft, C. in *The Cambridge World History of Violence, Vol. 1: Prehistory and Antiquity*, (eds Fagan, G., Fibiger, L., Hudson, M. & Trundle, M.) 418-437 (Cambridge Univ. Press, 2020).

[101] Hudson, M., Schulting, R. & Gilaizeau, L. in *The Cambridge World History of Violence, Vol. 1: Prehistory and Antiquity*, (eds Fagan, G., Fibiger, L., Hudson, M. & Trundle, M.) 160-177 (Cambridge Univ. Press, 2020).

[102] Kharakwal, J.S., Yano, A., Yasuda, Y., Shinde, V.S., Osada, T. Cord impressed ware and rice cultivation in South Asia, China and Japan: possibilities of inter-links. *Quat. Int*. **123-125**, 105-115 (2004).

[103] Wagner, M. *Neolithikum und Frühe Bronzezeit in Nordchina vor 8000 bis 3500 Jahren* (Berlin: Deutsches Archäologisches Institut, 2006).

[104] Woo, J-P. in *Early Korea-Japan Interactions* (eds Byington, M.E, Sasaki, K. & Bale, M.T.) 183-230 (Korea Institute, Harvard Univ., 2018).

[105] Ishige, N. *The History and Culture of Japanese Food* (London: Kegan Paul, 2001).

[106] McGovern, P.E., Zhang, J., Tang, J., Zhang, Z., Hall, G.R., Moreau, R.A., Nuñez, Butrym, E.D., Richards, M.P., Wang, C-S., Cheng, G., Zhao, Z. & Wang, C. Fermented beverages of pre- and proto-historic China. *Proc. Natl. Acad. Sci. U.S.A*. **101**, 17593-17598 (2004).

[107] Liu, L., Wang, J., Levin, M.J., Sinnott-Armstrong, N., Zhao, H., Zhao, Y., Shao, J., Di, N. & Zhang, T. The origins of specialized pottery and diverse alcohol fermentation techniques in Early Neolithic North China. *Proc. Natl. Acad. Sci. U.S.A*. **116**, 12767-12774 (2019).

[108] Habu, J., Kim, M., Katayama, M. & Komiya, H. Jomon subsistence-settlement systems at the Sannai Maruyama site. *Bull. Indo-Pac. Prehist. Assoc*. **21**, 9-21 (2001).

[109] Takahashi, R., Toizumi, T. & Kojo, Y. Archaeological studies of Japan: current studies of the Jomon archaeology. *Nihon Kōkogaku* **5**, 47-72 (1998).

[110] Rawson, J. China and the steppe: reception and resistance. *Antiquity* **91**, 375-388 (2017).

[111] Takamiya, H., Hudson, M., Yonenobu, H., Kurozumi, T. & Toizumi, T. (2015). An extraordinary case in human history: prehistoric hunter-gatherer adaptation to the islands of the central Ryukyus (Okinawa and Amami archipelagos), Japan. *Holocene* **26**, 408-422 (2015).

[112] Nelson, S.M. et al. Tracing population movements in ancient East Asia through the linguistics and archaeology of textile production. *Evol. Hum. Sci.* **2**, e5 (2020).

[113] Sabatini, S & Bergerbrant, S. (Eds.) *The Textile Revolution in Bronze Age Europe: Production, Specialisation, Consumption* (Cambridge Univ. Press, 2019).

[114] Byington, M.E. *The Ancient State of Puyŏ in Northeast Asia: Archaeology and Historical Memory* (Harvard Univ. Asia Center, 2016).

[115] Wada, S. The Northeast Asian tribes in the T'ang period. *Mem. Toyo Bunko* **17**, 1-25 (1958).

[116] Gong, Y., Li, L., Gong, D., Yin, H. & Zhang, J. Biomolecular evidence of silk from 8,500 years ago. *PLoS ONE* **11**, e0168042 (2016).

[117] Drennan, R.D., Lu, X. & Peterson, C.E. A place of pilgrimage? Niuheliang and its role in Hongshan society. *Antiquity* **91**, 43-56 (2017).

[118] Popov, A.N., Tabarev, A.V. & Mikishin, Y.A. Neolithization and ancient landscapes in southern Primorye, Russian Far East. *J. World Prehist*. **27**, 247-261 (2014).

[119] Komoto, M. & Obata, H., eds. *Zaisanovka 7 Site, in Primorsky, Russia* (Kumamoto Univ. & Russian Academy of Science, Far Eastern Branch, 2005).

[120] Wang, C-C. et al. Genomic insights into the formation of human populations in East Asia. *Nature* **591**, 413-419 (2021).

[121] Shin S.C., Rhee S.N. & Aikens. C.M. Chulmun Neolithic intensification, complexity, and emerging agriculture in Korea. *Asian Perspect*. **51**, 68-109 (2012).

[122] Matsumoto, N. Changing relationship between the dead and the living in Japanese prehistory. *Phil. Trans. R. Soc. B* **373**, 20170272.

[123] Sakaguchi, T. Mortuary variability and status differentiation in the Late Jomon of Hokkaido based on the analysis of *shuteibo* (communal cemeteries). *J. World Prehist.* **24**, 275-308 (2011).

[124] Nelson S.M. in *The Prehistory of Food: Appetites for Change* (eds Gosden, C. & Hather, J.) 147-165 (Routledge, 1999).

[125] Nesterkina, A.L., Solovieva, E.A., Tabarev, A.V. & Ivanova, D.A. The megaliths of Korea and Japan: an analysis of origins and functions. *Archaeol. Ethnol. Anthropol. Eurasia* **45**, 106–114 (2017).

[126] Kim, B. Socioeconomic development in the Bronze Age: archaeological understanding of the transition from the Early to Middle Bronze Age, South Korea. *Asian Perspect.* **54**, 144-184 (2015).

[127] Miyamoto, K. The spread of rice agriculture during the Yayoi period: from the Shandong peninsula to Japanese archipelago via Korean peninsula. *Jpn. J. Archaeol.* **6**, 109-124 (2019).

[128] Ege, K. *Bosei no tenkai ni miru Yayoi shakai* (Tokyo: Dōseisha, 2015).

[129] Liu, L. Ancestor worship: an archaeological investigation of ritual activities in Neolithic north China. *J. East Asian Archaeol.* **2**, 129-164.

[130] Nelson, S.M. in *The Archaeology and Politics of Food and Feasting in Early States and Empires* (ed Bray, T.) 65-89 (Kluwer/Plenum, 2003).

[131] Leipe, C., Kuramochi, S., Wagner, M. & Tarasov, P.E. Ritual practices and social organization at the Middle Yayoi culture settlement of Maenakanishi, eastern Japan. *Archaeol. Anthropol. Sci.* **12**, 134 (2020).

[132] Kokubun, A. Yayoi jidai-Kofun jidai shotō no bokkotsu. *Chiba Univ. Grad. School Hum. & Soc. Sci. Res. Proj. Rep.* **276**, 97-121 (2014).

[133] Flad, R.K. Divination and power: a multiregional view of the development of oracle bone divination in early China. *Curr. Anthropol.* **49**, 403-437 (2008).

[134] Bocquet-Appel, J-P. When the world's population took off: the springboard of the Neolithic demographic transition. *Science* **333**, 560-561 (2011).

[135] Pearson, R. Jomon hot spot: increasing sedentism in south-western Japan in the Incipient Jomon (14,000-9250 cal. BC) and Earliest Jomon (9250-5300 cal. BC). *World Archaeol.* **38**, 239-258 (2006).

[136] Drennan, R.D., Peterson, C.E. & Berrey, C.A. Environmental risk buffering in Chinese Neolithic villages: impacts on community structure in the Central Plains and the Western Liao Valley. *Archaeol. Res. Asia* **21**, e100165 (2020).

[137] Stevens, C.J. & Fuller, D.Q. Did Neolithic farming fail? The case for a Bronze Age agricultural revolution in the British Isles. *Antiquity* **86**, 707-722 (2012).

[138] Shennan, S., Downey, S.S., Timpson, A., Edinborough, K., Colledge, S., Kerig, T., Manning, K. & Thomas, M.G. Regional population collapse followed initial agriculture booms in mid-Holocene Europe. *Nat. Comms.* **4**, e2486 (2013).

[139] Kristiansen, K. in *The Oxford Handbook of Neolithic Europe* (eds Fowler, C., Harding, J. & Hofman, D.) 1093-1117 (Oxford Univ. Press, 2015).

[140] Rascovan, N., Sjögren. K-G., Kristiansen, K., Nielsen, R., Willerslev, E., Desnues, C. & Rasmussen, S. Emergence and spread of basal lineages of *Yersinia pestis* during the Neolithic decline. *Cell* **176**, 1-11 (2019).

[141] Hosner. D., Wagner, M., Tarasov, P.E., Chen, X. & Leipe, C. Spatiotemporal distribution patterns of archaeological sites in China during the Neolithic and Bronze Age: an overview. *Holocene* **26**, 1576-1593 (2016).

[142] Hudson, M.J. & Robbeets, M. Archaeolinguistic evidence for the farming/language dispersal of Koreanic. *Evol. Hum. Sci.* **2**, e52 (2020).

[143] Oh, Y., Conte, M., Kang, S., Kim, J. & Hwang, J. Population fluctuation and the adoption of food production in prehistoric Korea: using radiocarbon dates as a proxy for population change. *Radiocarbon* **59**, 1761-1770 (2017).

[144] Koyama, S. Jomon subsistence and population. *Senri Ethnol. Stud.* **2**, 1-65 (1978).

[145] Timpson, A., Colledge, S., Crema, E., Edinborough, K., Kerig, T., Manning, K., Thomas, M.G. & Shennan, S. Reconstructing regional population fluctuations in the European Neolithic using radiocarbon dates: a new case-study using an improved method. *J. Archaeol. Sci*. **52**, 549-557 (2014).

[146] Crema, E., Habu, J., Kobayashi, K. & Madella, M. Summed probability distribution of 14C dates suggests regional divergences in in the population dynamics of the Jomon period in eastern Japan. *PLoS ONE* **11**, e0154809.

[147] Imamura, K. *Prehistoric Japan: New Perspectives on Insular East Asia* (London: UCL Press, 1996).

[148] Hudson, M.J. *Ruins of Identity: Ethnogenesis in the Japanese Islands* (Univ. Hawai'i Press, 1999).

[149] Yano, K. in *Cultural Diversities and Comparative Archaeology* (ed Society of Archaeological Studies) 159-168 (Okayama: Kōkogaku Kenkyūkai, 2004) (in Japanese with English summary).

[150] Crema, E.R. & Kobayashi, K. A multi-proxy inference of Jōmon population dynamics using Bayesian phase models, residential data, and summed probability distribution of 14C dates. *J. Archaeol. Sci.* **117**, e105136 (2020).

[151] Drennan, R.D., Peterson, C.E., Indrisano, G.G., Mingyu, T., Shelach, G., Yanping, Z., et al. in *Regional Archaeology in Eastern Inner Mongolia: A Methodological Exploration* (ed Chifeng International Collaborative Archaeological Project) 152-165 (Beijing: Kexue chubanshe, 2003).
