# Supplementary material for: Triangulation supports agricultural spread of the Transeurasian languages
Source: Nature. 2021 Nov 10;599(7886):616–21. doi: 10.1038/s41586-021-04108-8 (PMC8612925; doi:10.1038/s41586-021-04108-8)
Supplement: Supplementary file 4 — This zipped file contains Supplementary Data Files 7–11; see Supplementary Information file for full descriptions. [file 41586_2021_4108_MOESM4_ESM.zip › 2021-02-02920E-s4/28_Eurasia3angle_synthesis_SI 8_Bayesian cultural interpretation_REV01.10.pdf]

Supplementary Information 8

Interpretation of our Bayesian phylogenetic analysis of the archaeological database in SI 25.

1 Methods

We scored 172 cultural traits as present (1) or absent (0) for a total of 255 Neolithic-Bronze Age archaeological sites phases from the West Liao river basin (36), the Amur (Jilin, Heilongjiang and inland Liaoning) (32), the Primorye (4), the Liaodong peninsula (37), the eastern steppes (1), the Shandong peninsula (4), the Yellow River basin (2), the Korean peninsula (58) and the Japanese Islands (85) in our archaeological database (SI 6). This scoring was analyzed through Bayesian phylogenetic methods using the models, priors, hyperpriors and settings specified in the method section of our manuscript and in the supplementary information (SI 20, 21). On the basis of current thinking in archaeology, we applied two constraints to our analysis. The first was a monophyletic constraint, grouping the Xinglongwa (8200–7400 BP), Zhaobaogou (7400–6500 BP) and Hongshang (6500–4900 BP) cultures from the West Liao together, in line with the observation that the defining features of the Hongshan society originated in the Xinglongwa and Zhaobaogou periods (Shelach and Teng 2013). The second was a constraint, grouping our 4 sites from the Zaisanovka culture (5200-3300 BP) in the Southern Primorye (i.e., Zaisanovka 7, Krounovka 1, Novoselishche-4 and Vodopadnoe-7) together with the Yabuli (4500-4000 BP) culture in the adjacent part of the Amur region, in line with the viewpoint of Yabuli as possible source for cultural influences on the Zaisanovka culture (Miyamoto 2014; Li et al. 2020). The results of our Bayesian analysis are visualized as a phylogenetic tree of archaeological cultures in Northeast Asia (SI 25).

We marked the main clades clustering in the tree by different colours: a Xinglongwa-Zhaobaogou-Hongshang cluster in red, an eastern Amur cluster in orange, a Liaodong-western Amur cluster in blue, a Xiaohexi-Neolithic Korean cluster in pink and a Bronze Age Xiajiadian-Korean-Japanese cluster in green (SI 25; Fig. SI 8.1). To visualize this cultural similarity in space, we applied the same colour coding to the sites marked on the map with the spatiotemporal distribution of our archaeological sites (Figure 2; Fig. SI 8.2).

The clustering of sites in the Bayesian tree can be taken as a measure of cultural similarity between different archaeological sites. This similarity can be caused by various factors such as areal diffusion due to geographical proximity, chronological simultaneity by belonging to the same time period and, cultural continuity through inheritance from previous generations. As such, we recognize that the features investigated in our archaeological dataset, such as ceramics, stone tools, buildings and houses, plant and animal remains, shell and bone artefacts and burials are not necessarily always transmitted through inheritance from one generation to the next but can also be transmitted through social contact, from one cultural society to its neighbour. Our phylogenetic tree thus captures not only a historical signal of descent of these cultures, but also a signal of interaction and contemporaneous existence. Nevertheless, it provides a solid quantitative basis for cultural similarity across time and space and allows us to make some inferences about cultural diffusion and migration.

Fig. SI 8.1 Bayesian phylogenetic analysis of the archaeological database with cultural clusters marked in different colours

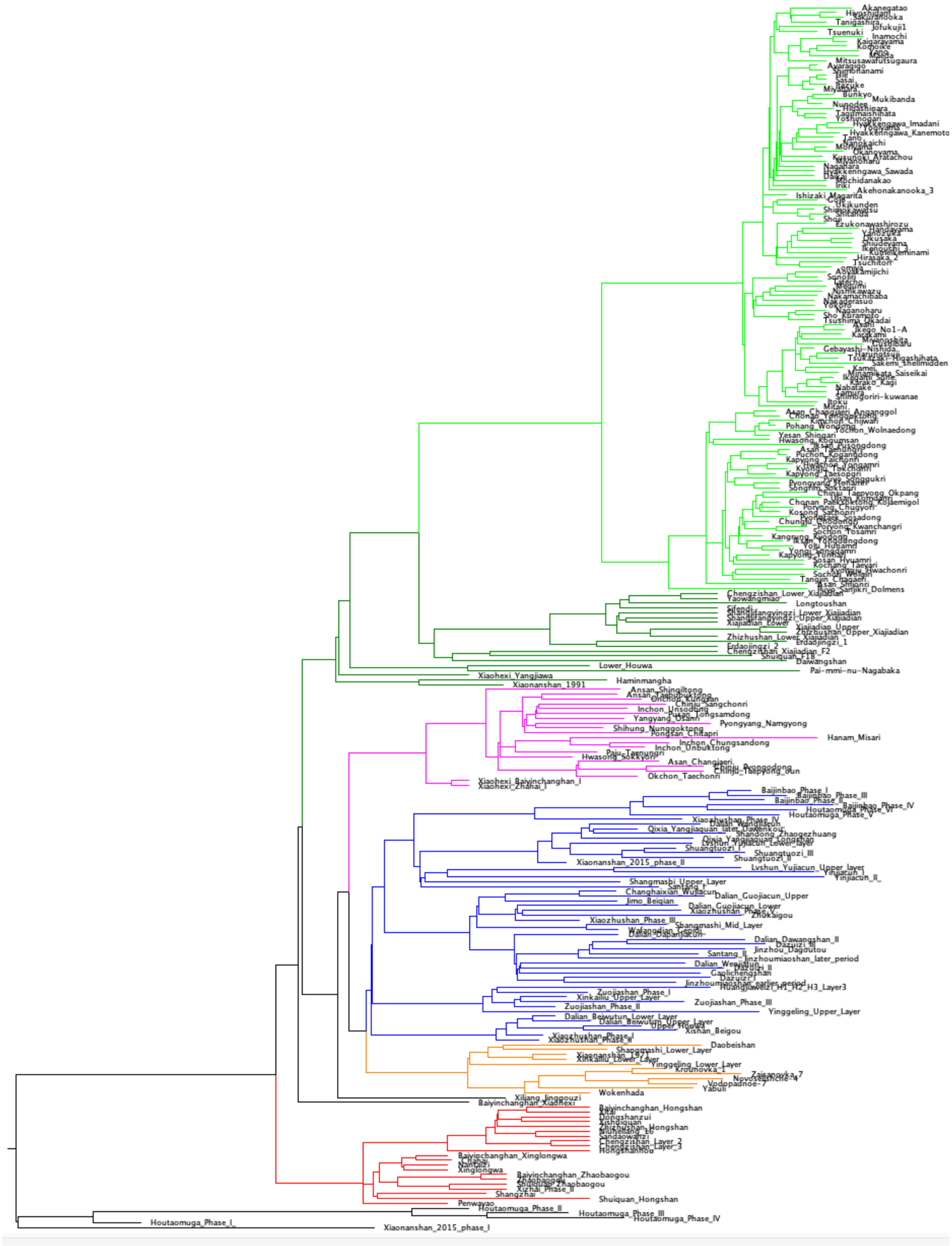

Fig. SI 8.2 Distribution of sites included in the archaeological database (a) with cultural clusters marked in different colours (b) in line with the Bayesian tree in Fig. SI 8.1.[1]

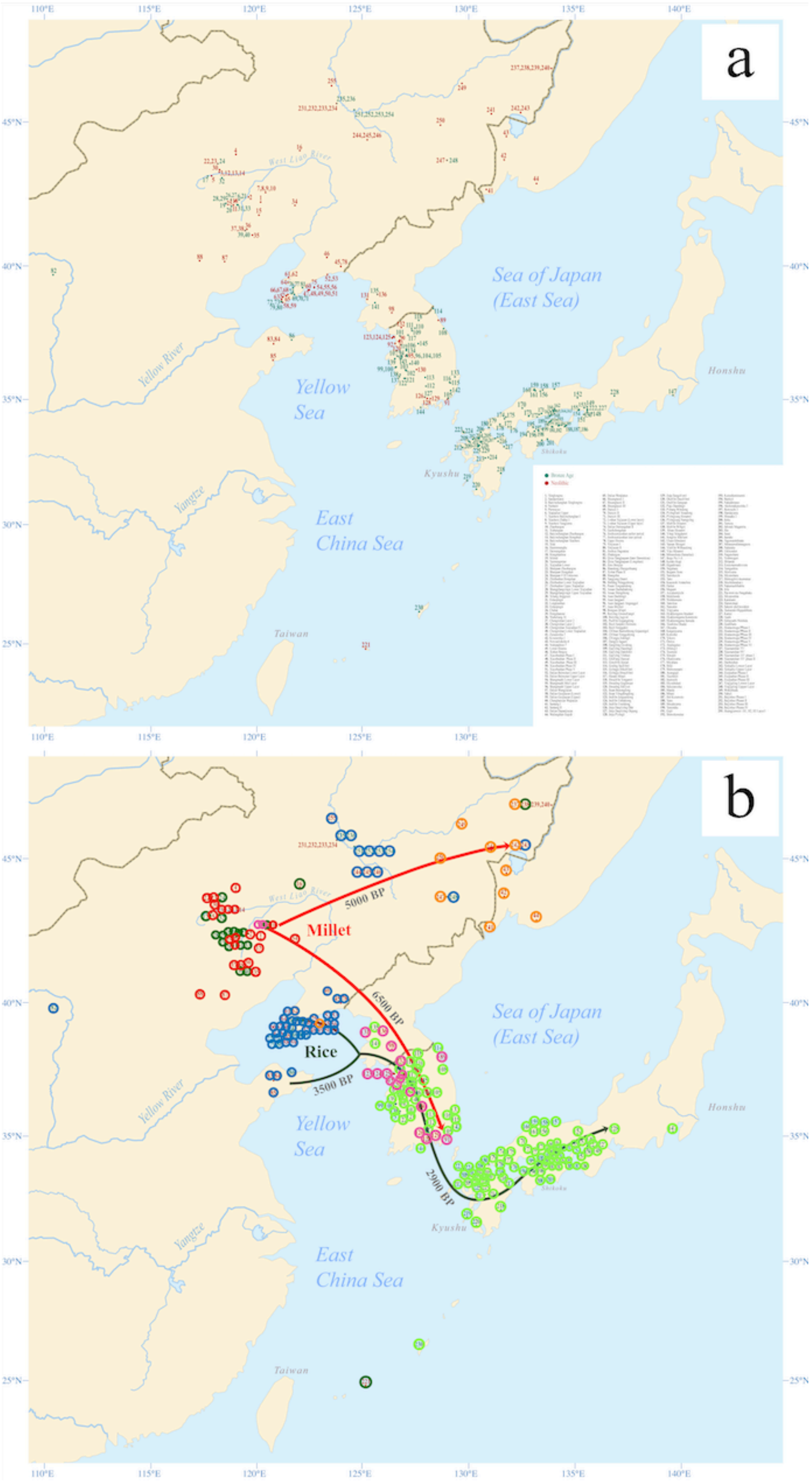
a
b
110°E
115°E
120°E
125°E
130°E
135°E
140°E
45°N
40°N
35°N
30°N
25°N
West Liao River
Yellow River
Yangtze
Sea of Japan
(East Sea)
Yellow
Sea
East
China Sea
Taiwan
Kyushu
Shikoku
Honshu
Millet
Rice
5000 BP
6500 BP
3500 BP
2900 BP

2 Results

We find that the major cultural complexes known from archaeology cluster correctly on the tree: Japanese Yayoi (3000–1700 BP) in light green, Korean Mumun (3500–2300 BP) in light green, Korean Middle-Late Chulmun (5500–2300 BP) in pink, the West Liao Xinglongwa-Zhaobaogou-Hongshan complex in red, West Liao Xiajiadian in dark green, the Liaodong Xiaozhushan (7000-4000 BP) and Shuangtuozi (4000 – 3500 BP) cultures in blue and Zaisanovka (5200-3300 BP) in the Primorye in orange.

In addition, cultural connections proposed by archaeologists in the past such as the Shandong-Liaodong interaction sphere (Xu 1995: 78-79) and the Yayoi-Mumun cluster (Miyamoto 2014, 2016) are well reflected in the tree. This is also the case for previously shown cultural discontinuity in particular regions, such as the discontinuity between Neolithic Chulmun and Bronze Age Mumun culture in Korea (Hudson & Robbeets 2020) between the Neolithic Xinglongwa-Zhabaogou-Hongshan complex and the Bronze Age Xiadiajian culture in the West Liao region (Shelach-Lavi 2015) and the alleged differences between the contemporaneous Hongshan (red) and Haminmangha (green) cultures in the West Liao region (Duan et al. 2018).

Remarkably, the sites belonging to the Xiaohexi culture (8000-7000 BP) do not fall within the Xinglongwa-Zhabaogou-Hongshan complex, but are scattered across different branches in the tree, one clustering with the non-Xinglongwa-Zhabaogou-Hongshan clade, another one appearing in the Xiajiadian complex while yet others cluster with the Korean Chulmun complex. This may indicate that all cultures marked in colour descend from different groups in the Xiaohexi phase of the West Liao area, preceding the Xinglongwa-Zhabaogou-Hongshan complex. The common root of all coloured branches, estimated at 9690 BP roughly coincides with the onset of sedentism and cultivation and the transition to a wetter climate in the region and is close to the root of our linguistic tree, estimated at 9181 BP (5595 -12793 95% HPD).

The observation that early sites with limited evidence for agriculture, such as Houtoumuga (Phase I to IV) as well as the Xiaonanshan (Phase I) site fall completely outside the tree, indicate that they do not derive from the same common ancestor as the other sites that we link to the spread of agriculture.

Archaeobotanical studies show that wet-rice agriculture reached the Korean peninsula as part of the Mumun culture in the late fourth millennium BP (3300–3000 BP) via the Shandong and Liaodong Peninsulas, from where it was transmitted to Japan after 3000 BP, by farmers who established the Yayoi culture (3000–1700 BP) (Ahn 2010, Miyamoto 2014,

2016). Against our expectation, however, the Bronze Age Mumun-Yayoi complex (light green) forms a clade together with the Xiajiadian complex (dark green) rather than clustering with the Liaodong-Shandong cultures (blue). This may be explained by cultural interaction with Xiajiadian groups in the Bronze Age, when the ancestors of the Mumun and Yayoi were living on the coasts between Liaodong and Shandong, neighbouring the Xiajiadian in the north. The cultural interaction may be mirrored by the fact that Mumun and Yayoi can be modelled in terms of Xiajiadian in our genetic admixture plot (Fig. 3b).

Archaeological evidence (Kuzmin 2013; Miyamoto 2014; Li et al. 2020) shows that millet agriculture dispersed directly across the Amur region to the Primorye. This is confirmed by the clustering of the eastern Amur sites from the Neolithic with the Zaisanovka cultures in the Primorye (orange). In contrast to the eastern Amur sites, the western Amur sites (blue), most of which date back to the Bronze Age, cluster with those on the Liaodong Peninsula. This may suggest that the Liaodong-Shandong cultural complex, for which our tree displays continuity from the Neolithic into the Bronze Age, spread northward into the western Amur region.

Only a few sites show an aberrant colour, unexpectedly different from neighbouring sites in the same region and/or time period. This is the case for the Neolithic Lower Houwa site (6500-5000 BP; green) on the Liaodong peninsula, the Neolithic Haminmangha site (6000-5000 BP; green) in the West Liao region, the Neolithic Xiaonanshan site excavated in 1991 (8000-6000 BP; green), the Neolithic Age Shangmashi Lower Layer site (6000-5000 BP; orange) in Liaodong and the Bronze Age Hanam Misari site (2600-2300 BP, pink) in Korea.

As Haminmangha is situated north of the Liao and Xiaonanshan further northeast in the Amur, the unexpected clustering with the Xiajiadian sites may mirror our genetic observation that Lower Xiajiadian individuals cause an influx of less admixed Amur genome into the West Liao region, suggesting migration from more northern regions (Fig. 3b; ED Fig. 8, 9).

References mentioned in Supplementary Information 8

Ahn, S. M. 2010. The emergence of rice agriculture in Korea: Archaeobotanical perspectives. *Archaeol. Anthropol. Sci.* **2**, 89–98.

Duan, Tianjing, Shiqi Ma, Shanshan Li & Yuhan Chen 2018. Petrographic analysis of pottery from the Haminmangha site (2010–2011), Inner Mongolia. *Asian Archaeol.* **2**, 43–50 https://doi.org/10.1007/s41826-018-0014-3

Hudson, M.J. & Robbeets, M. 2020. Archaeolinguistic evidence for the farming/language dispersal of Koreanic. *Evol. Hum. Sci.* **2**, e52.

Kuzmin, Y.V., 2013. The beginnings of prehistoric agriculture in the Russian Far East: Current evidence and concepts. *Documenta Praehist.* **40**, 1–12.

Li, T., Ning, C., Zhushchikhovskaya, I.S., Hudson, M.J. & Robbeets, M. 2020 Millet agriculture dispersed from Northeast China to the Russian Far East: integrating archaeology, genetics and linguistics. *Archaeol. Res. Asia* **22**, e100177.

Miyamoto, K., 2014. The initial spread of early agriculture into Northeast Asia. *Asian Archaeol.* **3**, 1–12.

Miyamoto, K., 2016. Archaeological explanation for the diffusion theory of the Japonic and Koreanic languages. *Jpn. J. Archaeol.* **4**, 53–75.

Shelach-Lavi, G. 2015. *The Archaeology of Early China: From Prehistory to the Han Dynasty* (Cambridge Univ. Press).

Shelach, G. & Teng, M. 2013, in *A Companion to Chinese Archaeology* (ed Underhill, A.) 37-54 (Wiley–Blackwell).

---

[1] The base map in the Figures of this SI file was downloaded from the Nature Earth map dataset (https://www.naturalearthdata.com/), granted for the public domain use and is free for use in any type of project. We designed the maps by ourselves and supplemented the maps with our own data points.
