# Supplementary material for: Triangulation supports agricultural spread of the Transeurasian languages
Source: Nature. 2021 Nov 10;599(7886):616–21. doi: 10.1038/s41586-021-04108-8 (PMC8612925; doi:10.1038/s41586-021-04108-8)
Supplement: Supplementary file 5 — This zipped file contains Supplementary Data Files 12–16; see Supplementary Information file for full descriptions. [file 41586_2021_4108_MOESM5_ESM.zip › 2021-02-02920E-s5/32_Eurasia3angle_synthesis_SI 12_site info_REV01.10.docx]

**Supplementary Information 12**

**Archaeological Context for Ancient DNA Samples Used in this Study**

This supplementary information provides background information on the samples used for ancient DNA analysis. Newly-reported samples are described referring to published excavation reports where available. The DNA samples from Nagabaka derive from a previously unpublished area of excavation at the site and are reported in detail. For previously published DNA samples, original sources are listed and a short summary provided.

**§1. North China**

Ancient DNA from 2 sites in China is newly reported in this study.

**§1.1 Chengjialiang**

Chengjialiang is an Early Neolithic Xinglongwa culture site in Fuxin, Liaoning province. One radius sample was subject to ancient DNA analysis. The sample is cross-dated to around 5550 BC.

**§1.2 Tachixigou**

The Tachixigou site is located in Liujiazi village, Fuxin city, Liaoning province. The site was first excavated in 2017 by the Liaoning Provincial Institute of Cultural Relics and Archaeology. It is an Early Neolithic site dated to ca. 6000 BC and associated with the Chahai type of the Xinglongwa culture based on the archaeological excavations. DNA from a tooth sample was shotgun sequenced in this study.

Previously reported samples used here comprise 16 sites from Northeast China (Longtoushan, Banlashan, Erdaojingzi, Haojiatai, Haminmangha, Pingliangtai, Wadian, Wanggou, Xiaowu, Lajia, Zhalainuoer, Wuqi, Miaozigou, Yuming, Mogushan, Ganga), 4 sites from Shandong (Bianbiandong, Boshan, Xiaojingshan & Xiaogao), and 2 sites from southern China (Xitoucun & Tanshishan, both in Fujian province)^[[1]](#endnote-1),^^[[2]](#endnote-2),^^[[3]](#endnote-3)^.

**§2. Taiwan**

The present study includes aDNA from 36 individuals from the Hanben (Hanpen) site, eastern Taiwan. At least two layers are reported at the site, an Iron Age Layer 4 dated AD 350-750 and a transitional late Neolithic/early Metal Age Layer 6 dated 50 BC to AD 350^3^. However, one of the aDNA samples has a much earlier direct radiocarbon date of 1402-1262 cal BC. Another direct date produced a calibrated result of AD 395-536. The other sampled skeletons from Hanben are divided into two phases, AD 160-465 (n=15) and AD 510-870 (n=19). Two individuals from Liangdao (Liangtao, Liang island) in the Matsu archipelago were directly dated to 8320-8060 cal BP (Liangdao 1, male) and 5640-5610 cal BC (Liangdao 2, female). Two individuals dated 4633-4287 and 2843-2457 cal BC came from the Suogang (Sokang) site located in the Penghu (Pescadores) islands.

**§3. Russia**

Our study used previously published aDNA samples from 4 sites in eastern Russia. Twelve individuals come from the pre-farming Neolithic Boisman site in the Primorye with direct radiocarbon dates ranging from 5371 to 3600 cal BC^3^. Six individuals come from the pre-farming Neolithic Devil’s Gate Cave, also in the Primorye, with direct dates spanning 7688 to 7438 BP^[[4]](#endnote-4)^. Of 16 individuals from the Shamanka site in Baikal, 10 were Neolithic (7160-6296 BP) and 6 were from the Bronze Age (ca. 3750 BP). Two individuals from Roshino 4 in the Primorye are thought to be associated with the Heishui (Blackwater) Mohe, a medieval Tungusic group. Direct radiocarbon dates from Roshino 4 produced calibrated results of AD 1039-1224 (Poz-83439) and 1050-1220 (PSUAMS-2115).

**§4. Mongolia**

The present study includes aDNA from 52 sites in Mongolia. Of these, 32 sites date to the late medieval era and 14 to the Early Iron Age Slab Grave culture, 3 to the Bronze Age, and 2 to the Neolithic^3,^^[[5]](#endnote-5)^. Direct radiocarbon dates are available for most of the Neolithic and Bronze Age samples (SI 11).

**§5. Korea**

**§5.1. Ando shell midden**

Belonging to the Incipient to Early Neolithic, this shell midden is located on Ando island at the southern end of the Yŏsu peninsula on the central coast of southern Korea. The site is 200 m^2^ in size, 10 m above sea level and contained 4 burials with 5 skeletal individuals, 9 hearths, 3 stone piles and 11 pit structures.^[[6]](#endnote-6),^^[[7]](#endnote-7),^^[[8]](#endnote-8)^ Artefacts include appliqué *yungkimun* pottery, stone tools, animal bone points and fish hooks, and shell bracelets, 3 animal tusk pendants and a slit stone earring. The site also has sherds of Initial Jōmon pottery and obsidian, presumably from sources in Japan. The shell midden consisted of 80% mussels and c. 17% oysters, and contained some fish bones (mainly red sea bream *Pagrus major*), as well as bones of terrestrial mammals such as deer and wild boar and marine mammals (whale, dolphin and sea lion). None of the skeletal remains analysed here for DNA have direct dates but the following radiocarbon results are available for the site, which dates from ca. 6400-3000 BC.

**Table SI 12.1. Radiocarbon dates from the Ando site**

| **Provenance** | **Material** | **Lab number** | **Uncalibrated date BP** | **Calibrated range BC (95.4%) (OxCal 4.4)** |
| --- | --- | --- | --- | --- |
| Trench 5 | Bone (whale) | SNU07-633 | 7410 ± 60 | 6417-6089 |
| Trench 5 | Bone (whale) | SNU07-634 | 7430 ± 60 | 6430-6091 |
| Trench 2 | Shell (oyster) | SNU07-A025 | 6650 ± 80 | 5715-5476 |
| Hearth 1 | Shell (oyster) | SNU07-A027 | 6780 ± 60 | 5787-5564 |
| Trench 2 | Bone (whale) | SNU07-635 | 6620 ± 110 | 5730-5365 |
| Hearth 1 | Shell (oyster) | SNU07-A026 | 5370 ± 60 | 4721-4446 |
| Trench 2 | Shell (oyster) | SNU07-A024 | 4490 ± 60 | 3366-2936 |

Among the five burials at Ando, one is a joint burial of a female (aged 20-30) and a male (aged 30-40). Human No. 2 (the male) wore a broken shell bracelet. One of the DNA samples analysed here was from burial number 3 and is a female aged 50-60 (Human No. 4). This skeleton was not well preserved, but 5 undamaged shell bracelets were wrapped around a wrist. The other human remains (two females and 1 male) do not have grave goods^7^.

Stable isotope analysis of human (n=5) and faunal remains (n=8) from Ando has suggested that the population was strongly dependent on marine resources. Isotopic ratios were very similar to those found at other southern peninsular Neolithic shell midden sites such as Tongsamdong and Daep’o, but differed from those at Neolithic western coastal sites such as Taejungni and K’onamri, leading to the hypothesis that marine dependence in the latter regions was not as strong^6^. External auditory exostoses, suggesting diving for marine resources, have been found at Ando (1 male and 1 female), as well as at Yokchido (1 male) and Yŏndaedo (3 males and 2 females).

**§5.2. Changhang**

Changhang (also romanised as Janghang), on the southeast coast of the peninsula, is the largest Neolithic cemetery site so far excavated in Korea^[[9]](#endnote-9),^^[[10]](#endnote-10)^. The site is located on Gadŏkto island, Pusan. Excavations in 2010-2011 produced skeletal remains of 48 individuals with grave goods including pottery, stone axes, 2 jade tubular beads (out of a total of 6 nephrite pendants), a perforated shark tooth, a cracked pendant of deer bone decorated with incised dots, and many shell bracelets (more than 20 shell bracelets were found in one burial alone). More than 500 pieces of obsidian found in the cemetery (raw material as well as finished tools such as arrowheads) suggest connections with Kyushu, the probable source of the obsidian. The burials come from the boundary between Layers VIII and IX and are dated to the Middle Neolithic. An OSL date of ca. 6400 BP is reported from Layer IX. Both extended (n=8) and flexed (n=23) burials were found, with the remaining 17 being too disturbed to confirm the original position. The presence of 10 females and 8 males and an age distribution between the 20s and 50s suggests Changhang was a general community cemetery.

Building on our earlier genomic analysis of two individuals^[[11]](#endnote-11)^, ancient DNA from a total of 5 individuals from Changhang was used in the present study. The samples and direct radiocarbon dates are shown in Table SI 12.2. The DNA analysis of GD1018 was not successful but the date is included in the table. The dates assign the samples to the fifth millennium BC and the Early Neolithic phase^[[12]](#endnote-12)^. Our present GD1002 and previous No. 2 samples are both said to derive from burial 46. We conducted a genetic kinship analysis which showed that both samples share a low Pairwise mismatch rate (pmr), suggesting that they are indeed the same individual. The same analysis conducted on the present GD1008 and the previous No. 8 samples confirmed that they are different individuals (Table SI 12.3).

**Table SI 12. 2. Radiocarbon dates from Changhang**

| **DNA lab ID** | **Burial number** | **Material** | **C14 Lab code** | **Uncalibrated date BP** | **Calibrated range BC (95.4%) OxCal 4.4** | **DNA used in present study** |
| --- | --- | --- | --- | --- | --- | --- |
| GD1002 | 46 | Tooth | MAMS-39298 | 5754+28 | 4696-4505 | Yes |
| GD1008 | 38 | Tooth | MAMS-39299 | 5768+28 | 4706-4542 | Yes |
| GD1009 | 19 | Tooth | MAMS-39300 | 5713+27 | 4673-4457 | Yes |
| GD1018 | 01 | Tooth | MAMS-39301 | 5584+28 | 4486-4352 | No |
| No. 2* | 46 | Temporal bone | KRKJH-2 | 5580+21 | 4451-4356 | Yes |
| No. 8* | 35 | Temporal bone | KRKJH-8 | 5583+20 | 4452-4357 | Yes |

Note: * Samples from our previous study^11,^^[[13]](#endnote-13)^.

**Table SI 12.3** Genetic kinship identification.

| **Sample 1** | **Sample 2** | **nSNPs** | **nmismatch** | **pmismatch** |
| --- | --- | --- | --- | --- |
| GDI002^ | No. 2* | 4486 | 441 | 0.09831 |
| GDI009^ | No. 2* | 2551 | 528 | 0.20698 |
| GDI009^ | No. 8* | 4174 | 864 | 0.207 |
| GDI002^ | No. 8* | 7285 | 1512 | 0.20755 |
| GDI008 | No. 2* | 7609 | 1634 | 0.21475 |
| GDI008 | No. 8* | 12196 | 2620 | 0.21482 |

Note: The first column is the samples from this study. ^ Genetic data from damage-restricted reads;

* Samples from our previous study^11^.

**§5.3. Taejungni**

Taejungni (also romanised as Daejukri) is located in Sŏsan on the west coast of South Korea. Three Neolithic shell midden sites were excavated at the Taejungni locality, which also has remains from the Chosŏn period (1392-1897). The human remains came from the shell midden area excavated by the Chungcheong Institute of Cultural Heritage^[[14]](#endnote-14),^^[[15]](#endnote-15)^. The Taejungni excavation location which produced the human remains analysed here has three published radiocarbon dates (Table SI 12.4). The provenance and lab numbers for these three dates are not available, and two of the dates have very large uncertainty values. These three dates have a wide range from ca. 4000 to 2500 BC. The DNA sample analysed here came from below the Neolithic shell midden. While no stratigraphic disturbance was reported by the excavators, a direct radiocarbon date on the sample gave a Bronze Age result (Table SI 12.4). Although unexpected, this Bronze Age date is supported by the results of our DNA and isotope (SI 15) analyses.

**Table SI 12.4. Radiocarbon dates from Taejungni (this study and previously published**^8^**).**

| **Sample ID/Provenance** | **Material** | **Lab code** | **Uncalibrated date BP** | **Calibrated range BC (95.4%) (OxCal 4.4)** |
| --- | --- | --- | --- | --- |
| DAJ001 | Human bone (this study) | MAMS-39297 | 2488+18 | 768-542 |
| Unknown | Charcoal | N/A | 4530 ± 60 | 3491-3024 |
| Unknown | Charcoal | N/A | 5150 ± 150 | 4323-3649 |
| Unknown | Charcoal | N/A | 3960 ± 170 | 3320-1767 |

**§5.4. Yokchido**

Yokchido (also romanised as Yokjido) is a shell midden on the island of the same name. Burials at the site included a pile of stones covering the remains of three people including a young adult female and a male. This male displayed external auditory exostoses, suggesting he regularly engaged in diving for marine resources^[[16]](#endnote-16)^. In addition to local Chulmun ceramic wares, several types of Jōmon artefacts from Kyushu have been found at Yokchido. These include Middle Jōmon Funamoto II and small quantities of Late Jōmon pottery (several styles related to the Adaka type), arrowheads and harpoons made of obsidian and sanukite, and a so-called Kamasaki-type scraper^[[17]](#endnote-17),^^[[18]](#endnote-18),^^[[19]](#endnote-19)^. Yokchido has also produced two small, crudely-made clay figurines interpreted as depicting a wild boars from Middle Chulmun contexts ^[[20]](#endnote-20),^^[[21]](#endnote-21)^. These are plausibly interpreted as an influence from Japan where more than 70 boar figurines are known from the Jōmon period^[[22]](#endnote-22)^. Specifically, the Yokchido figurines have been compared to a similar figurine from the Miyashita shell midden on Gotō island, Nagasaki ^19,20,^^[[23]](#endnote-23)^.

Only 1 radiocarbon date from Yokchido has been published (Table SI 12.5). The sample used here for DNA analysis was from the fragmentary skeleton No. 3 from Pit D2. This sample was not directly dated but can be dated on stratigraphic grounds to the Middle Chulmun Neolithic phase, ca. 3500-2000 BC.

**Table SI 12.5. Radiocarbon date from Yokchido**^8^**.**

| **Provenance** | **Material** | **Lab Code** | **Uncalibrated date BP** | **Calibrated range BC (95.4%) (OxCal 4.4)** |
| --- | --- | --- | --- | --- |
| Hearth 1 | Charcoal | PLD-24965 | 3850+20 | 2454-2205 |

**§5.5. Yŏndaedo**

Yŏndaedo (also romanised as Yeondae-do) is a shell midden site in T’ongyŏng, on the southern coast of South Korea. The site has 15 pit burials, 13 of which contained human skeletal remains. With the exception of burial No. 2 (a joint grave with 3 individuals), all were single inhumations. While all burials contained grave goods, the fact that Nos. 7, 11 and 14 contained more artefacts in terms of both quality and quantity has been interpreted as evidence for social inequality. Grave goods included ankle and arm bracelets made of shell and animal teeth, a hairpin and pendants (in grave No. 7, a male in his 30s) and 2 tubular beads of agate and nephrite (in grave No. 14, age and sex unknown)^16^. The ankle bracelets in grave 7 consisted of >100 perforated animal teeth (otter, raccoon dog and dolphin)^[[24]](#endnote-24)^.

Evidence of interaction with northern Kyushu consists of Early Jōmon Todoroki B and Middle Jōmon Kasuga pottery. Large quantities of obsidian are also reported, both as raw materials and finished tools including arrowheads (86% of tools) and scrapers. Sanukite debitage was also found together with sanukite arrowheads, scrapers and harpoon heads including a harpoon type typical of northwest Kyushu^17,18^.

Isotope analysis of human remains (N=3; 2 males, 1 female) indicated a plant-based diet with considerable protein consumption from marine resources, mainly shellfish and fish, with no significant dietary differences between the sexes^[[25]](#endnote-25)^. Oxygen isotope analysis of oyster samples (N=3) suggested that these were harvested mainly during spring, during which the site was occupied^[[26]](#endnote-26)^. None of the 5 individuals analysed here for aDNA were directly dated. Two radiocarbon dates from the site have been published (Table SI 12.6)^8^.

**Table SI 12.6. Radiocarbon dates from Yŏndaedo**

| **Provenance** | **Material** | **Lab code** | **Uncalibrated date BP** | **Calibrated range BC (95.4%) (OxCal 4.4)** |
| --- | --- | --- | --- | --- |
| Trench S | Shell | NUTA-2314 | 6090 ± 160 | 5459-4614 |
| Trench T1 | Shell | NUTA-2315 | 6010 ± 160 | 5306-4544 |

**§6. Japan**

**§6.1. Antokudai**

Antokudai is a multi-component settlement and cemetery site with both Bronze Age and historic occupations located on the edge of the Fukuoka plain in Nakagawa town, northern Kyushu. Excavations were conducted from 1997-2003. At the site cemetery, 10 Yayoi jar burials and 5 Kofun wooden coffin burials were identified. Grave goods in the most elaborate Yayoi burial included 44 shell (*Sinustrombus latissimus*) bracelets from Okinawa and over 300 glass beads^[[27]](#endnote-27)^. The sample analysed here comes from jar burial 5 which dates to the Bronze Age Middle Yayoi (Fig. SI 12.1). The skeleton is an older adult female. A direct radiocarbon date on the left femur produced a result of 1985+ 20 bp (PLD-36861), calibrated to 25 BC – AD 80^[[28]](#endnote-28)^, or to 41 BC – AD 113 (95.4%) using OxCal 4.4. Building on our recent preliminary analysis of the DNA from burial 5^[[29]](#endnote-29)^, we here report full genomic data for this individual.


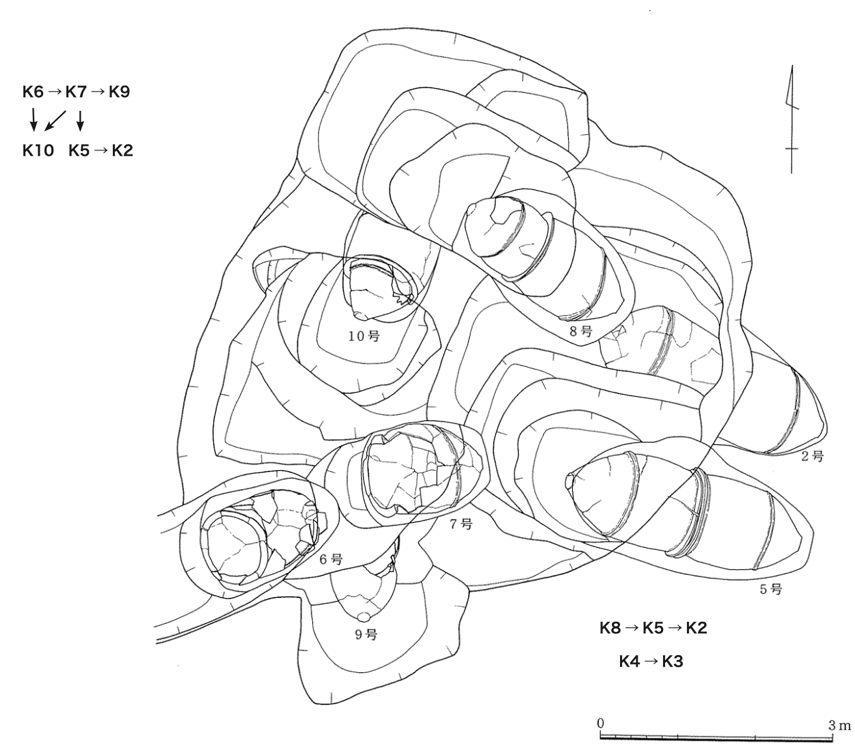


**Fig. SI 12.1.** Jar burials at Antokudai. The DNA sample used in this study comes from jar No. K5^28^.

**§6.2. Funadomari**

The Funadomari site is located on the northern coast of Rebun Island, Hokkaido, in the extreme north of the Japanese archipelago (Figs. SI 12.2 & 12.3). The site is on a sand dune approximately 5 m above sea level. Excavations in 1998 found 28 primary inhumation burials in flexed position associated with artefacts including necklaces, bracelets and anklets made of the marine clam *Mercenaria stimpsoni*. Ceramic cross-dating suggests the burials date to the early to middle Late Jōmon (ca. 2500-1500 BC). The Funadomari Jōmon crania show morphological resemblances to other known Jōmon crania from Honshu and also display evidence for ritual tooth ablation, a custom known at many other Jōmon sites from the Late phase onwards^[[30]](#endnote-30)^. Long-distance cultural relations are evident from the presence of asphalt on tools, 4 Itoigawa jade pendants/beads from Niigata, and exotic shells such as cowrie (Cypraeidae), cone shells (*Conus* sp.) and Olivoidea (J. *makuragai*)^[[31]](#endnote-31)^.


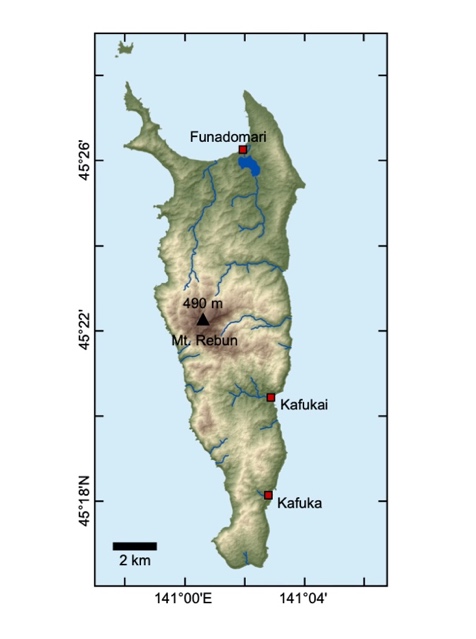


**Fig. SI 12.2.** Rebun island, Hokkaido with the location of the Funadomari site. Map by C. Leipe, Baikal-Hokkaido Archaeology Project

The aDNA data used here consists of previously-published results from a female (F23) and a male (F5)^[[32]](#endnote-32)^. A direct radiocarbon date on a rib from F23 gave a date of 4025±20 bp, calibrated to 3960-3550 cal BP or 2010-1600 BC. Re-calibration with OxCal 4.4 gives a result of 2617-2471 cal BC. This result is consistent with reported radiocarbon date on charcoal from a hearth at Funadomari which produced a range between 3635±65 and 3850±55 cal BP.


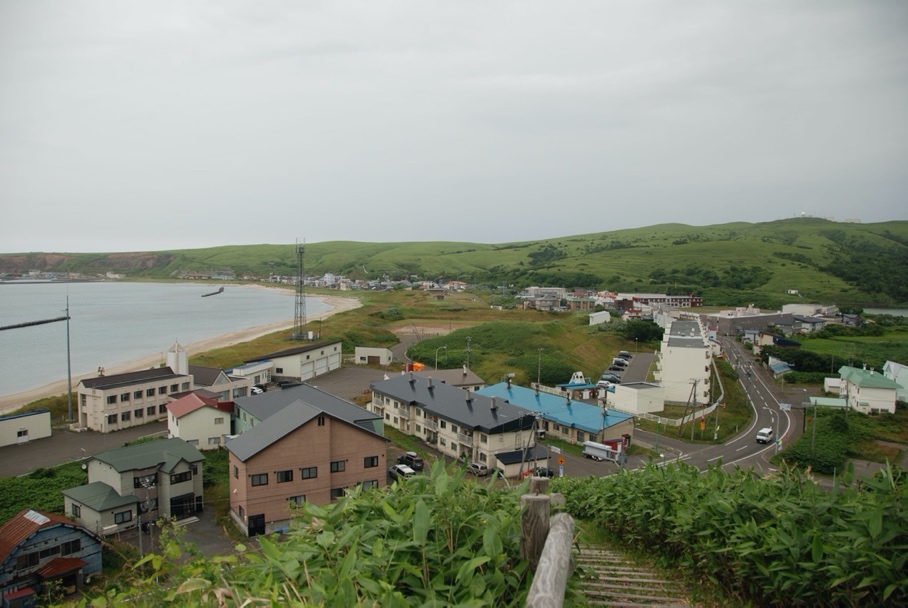


**Fig. SI 12.3**. General view of the Funadomari site. Photo: M. Hudson.

**§6.3. Ikawazu**

Ikawazu is a Late-Final Jōmon shell midden site located in Tahara city, Aichi prefecture. Although the site was first excavated in 1918, producing over 100 Jōmon skeletons, the ancient DNA sample used here (IK002) derives from new excavations conducted in 2010. IK002 was found in Pit 4 in association with Gokan-no-mori pottery. This pottery type belongs to the Final Jōmon, *not* the Late Jōmon as reported in a previous publication^[[33]](#endnote-33)^. A radiocarbon date on IK002 produced a result of 2681+16 bp, calibrated (95.4%) to 2720-2418 BP or 770-468 BC. The OxCal 4.4 calibration of this date gives an earlier age of 897-803 BC. This radiocarbon date is consistent with the Final Jōmon in the Tōkai region.

**§6.4. Kuma-Nishioda**

Kuma-Nishioda is a large Yayoi-period settlement site in Chikushino city, Fukuoka prefecture, northern Kyushu. Around 1550 burials have been found at the site, comprising several different mortuary systems such as jar burials, wooden coffins, stone coffins, stone cists and moated burial precincts. The cemetery may have been a communal resting place for several local settlements. Human skeletons were found in 429 graves. Significantly more males than females have been identified in the cemetery (Table SI 12.7). An elite burial possessed a Han Chinese bronze mirror, an iron sword, an iron halberd and 41 shell bracelets as grave goods. A hoard of 23 bronze halberds was also found in the area of the site^[[34]](#endnote-34)^. Building on our previous studies of mtDNA from the site^[[35]](#endnote-35),^^[[36]](#endnote-36)^, here we report genomic data from two Middle Yayoi jar burials at Kuma-Nishioda. Both jars come from area 10 of the site. Burial 10-K-138 is an adult male with a stone arrowhead found in the chest area. 10-K-175 is a male older adult. No direct radiocarbon dates are available but the burial jars are typologically dated to the second century BC for 10-K-138 and the second to first centuries BC for 10-K-175.

**Table SI 12.7. Yayoi burials at Kuma-Nishioda**^27^**.**

|  | **Sex** | **MNI** | **Percentage (rounded)** |
| --- | --- | --- | --- |
| **Adults** | Male | 170 | 40 |
|  | Female | 96 | 22 |
|  | Indeterminate | 98 | 23 |
| **Subadults** |  | 65 | 15 |
|  | Total | 429 | 100 |

**§6.5. Nagabaka (Paimmi-nu-Nagabaka)**

Paimmi-nu-Nagabaka (hereafter abbreviated as Nagabaka) is a rock shelter and shell midden site located on the central ridge of the northern peninsula of Miayko Island, Okinawa Prefecture (site 22 in main text Fig. 2). The site is approximately 25 m above sea level facing east. The distance from the site to the ocean on both sides is currently less than 1 km.

The Nagabaka rock shelter contains a large cemetery of the early modern period (ca. 1600-1879) with commingled skeletal remains. In 2005, while conducting survey and mapping work on this cemetery we discovered *Telescopium telescopium* shells on the talus slope in front of the rock shelter. This mangrove gastropod is now extinct in Okinawa and Taiwan^[[37]](#endnote-37)^. Eight seasons of excavations conducted from 2006 to 2013 confirmed that this was a previously unknown prehistoric site. Eight test trenches were dug (Fig. SI 12.4). Trenches 3, 4, 6 and 7 were opened to determine the spatial extent of the midden deposits but produced almost no artefacts. The DNA results reported here come from the North Area trench inside the rock shelter. Excavation of the North Area trench involved first setting to one side the early modern human remains on the surface of the rock shelter; those remains were replaced after excavation and backfilling (Fig. SI 12.5). No human remains from the early modern cemetery were removed, except for a small sample of long bones diagnosed as bearing traces of yaws which is now curated by Miyakojima City Board of Education^[[38]](#endnote-38)^. All of the DNA samples from Nagabaka analysed here come from our excavations not from the cemetery.

**Fig. SI 12.4.** Location of excavation trenches and the North Area of the rock shelter cemetery at Nagabaka. The Central and South Areas of the rock shelter are not shown in this plan

Our aDNA results show two very different populations at Nagabaka. Archaeologically these relate to two distinct cultural phases at the site. The lower levels belong to a prehistoric phase with radiocarbon dates ranging from 2452 BC to AD 790 (see below). Although there is also one radiocarbon date of AD 977-1118 on a *T. telescopium* shell from the surface of the site, Nagabaka otherwise seems to have been abandoned from the ninth century AD and no artefacts can be assigned to the medieval (Gusuku) period. The next use of the site was from the seventeenth century when the rock shelter began to be used as a cemetery.


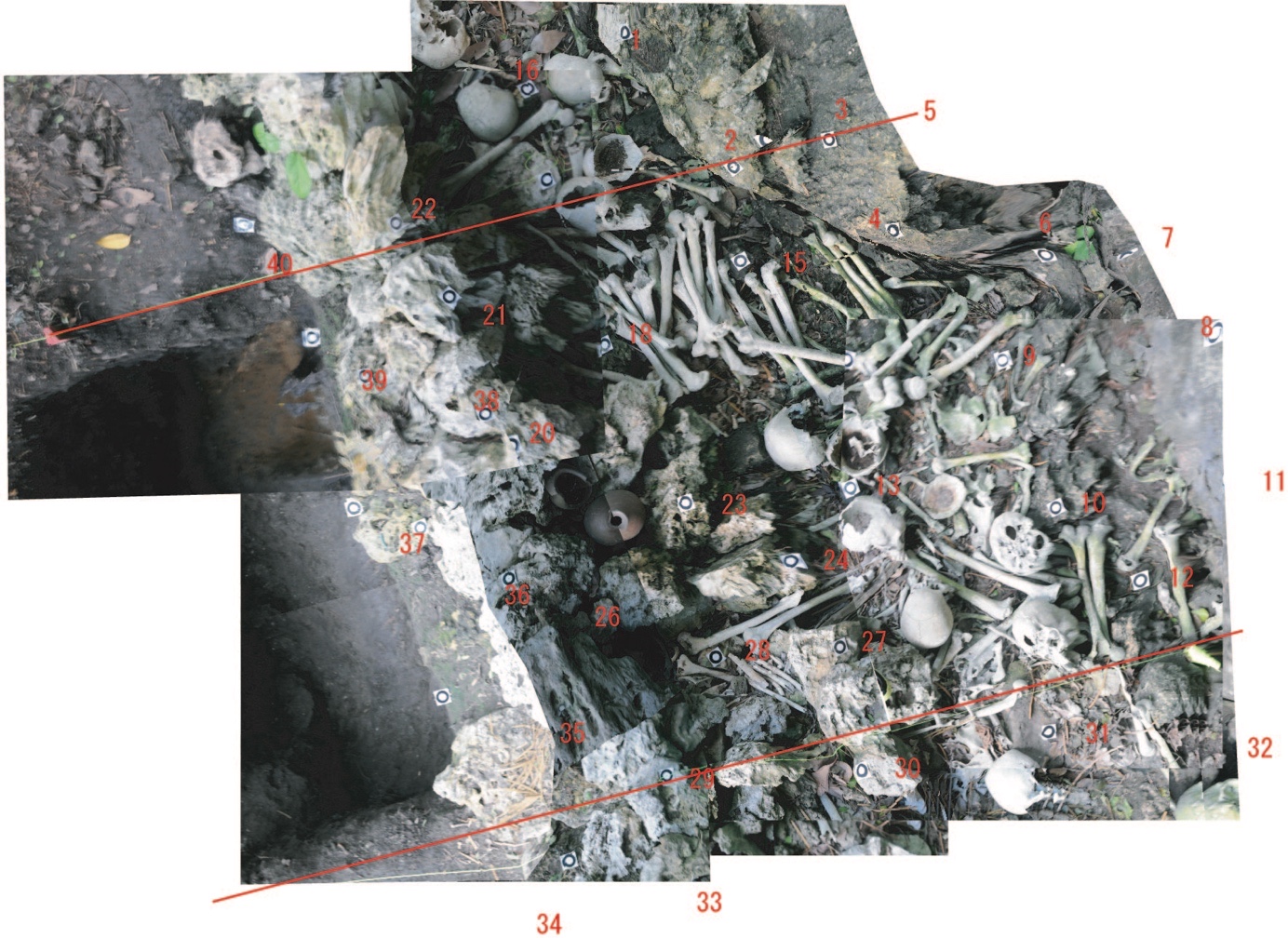


**Fig. SI 12.5.** Trench 2 (left) after excavation and early modern skeletal remains in the North Area of the rock shelter cemetery (right). A low wall of unmodified coral limestone blocks delineates the edge of the cemetery. The accumulation of long bones at centre-top is not an *in situ* deposit but appears to reflect bones collected for measurement during previous anthropological investigations. The North Area Trench was dug under the skeletal remains between the two red lines.

**§6.5.1. Chronology**

*Early modern cemetery*: Nagabaka is a ‘wind burial’ cemetery (*fūsōbo*), a mortuary custom common in the Ryukyu islands in the early modern era^[[39]](#endnote-39)^. Bodies were left exposed in caves or rock shelters until the flesh had decayed; the bones were then ritually washed (usually with rice wine) and often placed in ceramic jars or wooden coffins^[[40]](#endnote-40),^^[[41]](#endnote-41)^. The first anthropological analysis of Nagabaka, published in 1974, noted the presence of fragments of a ‘boat-shaped wooden coffin’^[[42]](#endnote-42)^. This coffin was not mentioned by later researchers and no trace of it remained in 2005 when we began our fieldwork. There were, however, some 185 pieces of wood found in the rock shelter. Most of these were fragments but some wooden boards were also present and may originally have formed a coffin or coffins.

Seven radiocarbon dates from Nagabaka relate to the cemetery (Table SI 12.8). Six of these are from human bones or teeth found either in the topsoil layer of Trench 1 or Layer 1 of the North Area Trench. The Trench 1 bones are assumed to have washed down the slope from the rock shelter above. The teeth from the North Area Trench are likewise interpreted as having moved down the stratigraphic sequence from the surface cemetery layer. All of these dates unfortunately have large uncertainty ranges. The seventh date is on a wood fragment from the North Area and also has a large span from the late fifteenth to early seventeenth centuries.

**Table SI 12.8**. Radiocarbon dates from the Nagabaka site.

| Lab. No. | Sample substance | Sample context | Uncalibrated date BP | Calibration curve | Calibrated range BC/AD (95.4%) |
| --- | --- | --- | --- | --- | --- |
| MAMS-39293 | Human tooth | North Area trench, Layer 1 | 207+21 | OxCal 4.4 | AD 1650-1929 |
| MAMS-39294 | Human tooth | North Area trench, Layer 1 | 165+22 | OxCal 4.4 | AD 1664-1911 |
| MAMS-39296 | Human tooth | North Area trench, Layer 1 | 171+21 | OxCal 4.4 | AD 1662-1915 |
| PLD-26944 | Human bone | Tr.1, topsoil | 141+18 | OxCal 4.4 | AD 1673-1944 |
| PLD-26945 | Human bone | Tr.1, topsoil | 197 ± 19 | OxCal 4.4 | AD 1656-1927 |
| PLD-26946 | Human bone | Tr. 1, surface? | 152 ± 19 | OxCal 4.4 | AD 1668-1908 |
| PLD-15678 | Wood (Podocarpaceae) | North Area surface | 342+19 | OxCal 4.4 | AD 1477-1635 |
| PLD-17965 | Shell (*T. telescopium*) | Tr.1, surface | 1367+22 | MARINE09 | AD 977-1118 |
| TERRA-010407a08 | Fish bone | Tr. 1, L2 | 1846+37 | MARINE04 | AD 620-790 |
| TERRA-010407a09 | *Sus* P3 | Tr. 1, L2 | 1547+38 | OxCal 4.4 | AD 429-596 |
| TERRA-010407a10 | Scaridae bone | Tr. 1, L2 | 1693+38 | MARINE04 | AD 450-660 |
| TERRA-010407a13 | Mammal bone | Tr.1, L2 | 1466+72 | OxCal 4.4 | AD 426-671 |
| UGAMS-R03213 | Mammal bone (*Sus*?) | Tr.1, L3 | 2080+30 | OxCal 4.4 | 175 BC – AD 8 |
| PLD-15679 | Charcoal | Tr.1, L3 | 1742+23 | OxCal 4.4 | AD 245-383 |
| PLD-15680 | Shell (*Tectus niloticus*) | Tr.1, L2 | 2032+21 | MARINE09 | AD 269-424 |
| PLD-15681 | Human ulna fragment | Tr.1, L3 | 1452+20 | OxCal 4.4 | AD 582-648 |
| PLD-15682 | *Sus* cranium | Tr.1, L2 | 1922+22 | OxCal 4.4 | AD 30-205 |
| PLD-17967 | *Turbo argyrostomus*  operculum | Tr.1, L3 | 2813+22 | MARINE09 | 733-512 BC  (95.4%) |
| IAAA-123796 | Shell (*T. telescopium*) | North Area surface | 1660+25 | MARINE09 | AD 669-790  (95.4%) |
| IAAA-123797 | Charcoal | Tr.1, L4 | 3050+28 | OxCal 4.4 | 1403-1224 BC |
| PLD-26943 | Human bone | Tr.1, surface | 2383+22 | OxCal 4.4 | 539-396 BC |
| MAMS-30697 | Human tooth (molar) | North Area trench, Layer 2 | 2621+19 | OxCal 4.4 | 811-781 BC |
| MAMS-30699 | Human bone (distal phalange) | North Area trench, Layer 4 | 2543+20 | OxCal 4.4 | 795-567 BC |
| MAMS-30700 | Human tooth (incisor) | North Area trench, Layer 3 | 2622+20 | OxCal 4.4 | 812-782 BC |
| MAMS-39295 | Human tooth | North Area trench, Layer 1 | 2632+19 | OxCal 4.4 | 819-787 BC |
| MAMS-30698 | Human tooth (molar) | North Area trench, Layer 3 | 3607+20 | OxCal 4.4 | 2027-1897 BC |
| IAAA-113321 | *Turbo argyrostomus*  operculum | Tr.1, L7 | 4038+27 | MARINE09 | 2209-2004 BC (95.4%) |
| PLD-17966 | *Turbo argyrostomus*  operculum | Tr.1, L7 | 4203+24 | MARINE09 | 2452-2258 BC (95.4%) |

Artefacts associated with the Nagabaka historic cemetery include pottery and tobacco pipes. The pottery was found inside the rock shelter as well as on the surface below, and included earthenware, stoneware and porcelain. One rim sherd of earthenware may date to as early as the late sixteenth century, but the other ceramics suggest a range of dates from the seventeenth to nineteenth centuries (Fig. SI 12.6). Tobacco pipes made of metal, stone and pottery can also be dated to the early modern period. None of the skulls in the Nagabaka cemetery have dental fillings or other modern dental treatment. Modern dentistry began in Japan in the late nineteenth century but on Miyako island the first dental practice was established in 1925^[[43]](#endnote-43)^. An ethnographic study of Karimata village near Nagabaka conducted in 1951 reported that an earlier tradition of ‘cave burials along the coast’ was no longer employed except occasionally for very young children^[[44]](#endnote-44)^. The Nagabaka cemetery has few grave goods and likely consists of the local peasant population during the early modern era when, while nominally independent, the Ryukyus were under the semi-colonial control of the Satsuma domain of southern Kyushu.


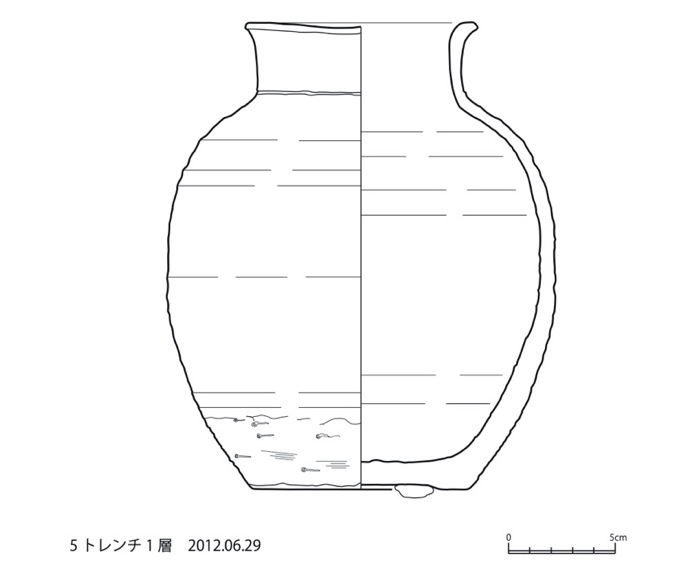


**Fig. SI 12.6.** Stoneware from Layer 1 of Trench 5 at Nagabaka

*The prehistoric site*: Trench 1, on the talus slope in front of the rock shelter, has ca. 2 m of cultural deposits divided into 7 stratigraphic layers (Figs. SI 12.7 & 12.8). No pottery was discovered in any of the prehistoric layers and dating relies on radiocarbon dates.


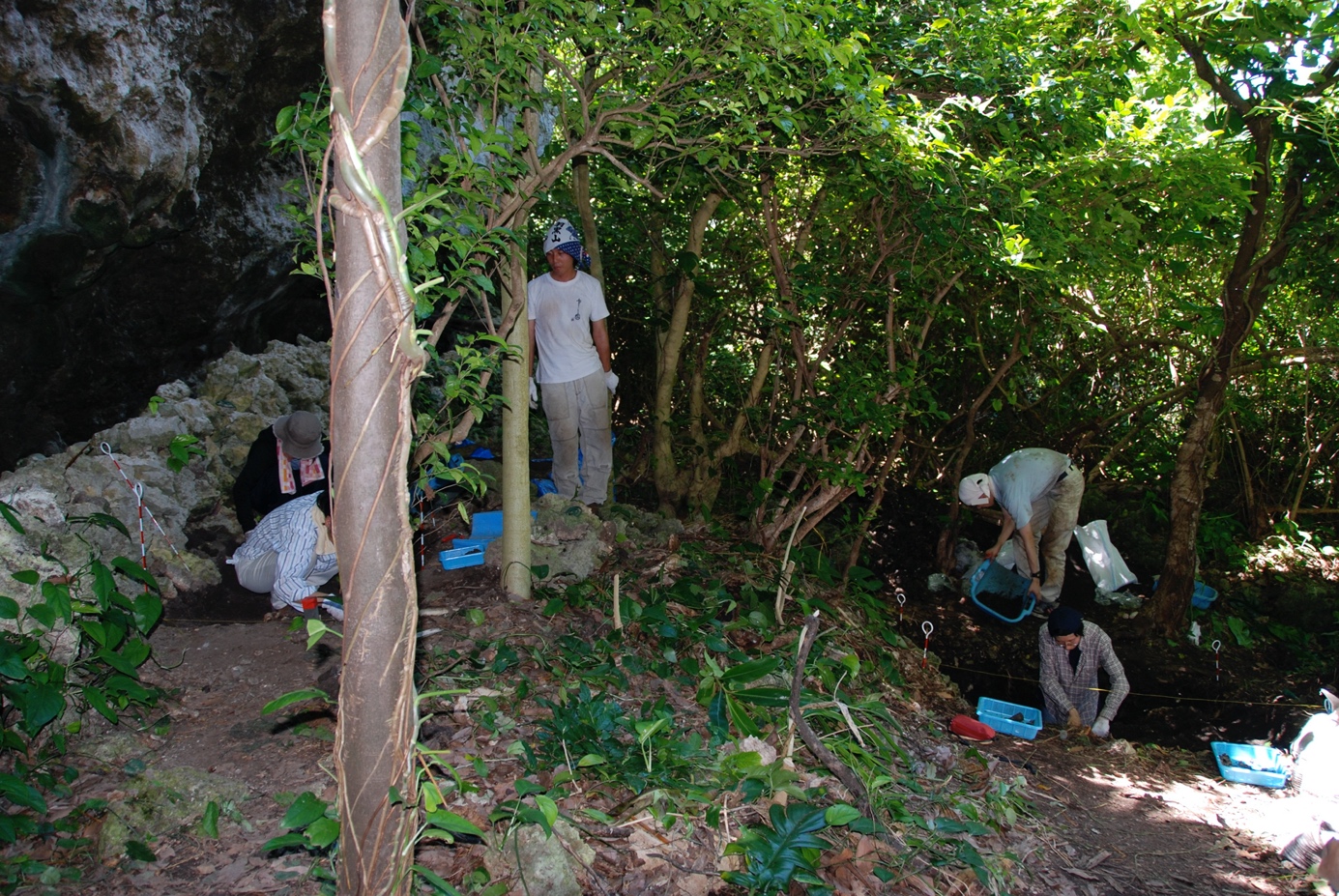


**Fig. SI.7.** Excavation of Trench 1 (right) and Trench 2 (left). Taken from the south, the photo shows the North Area of the rock shelter with its retaining wall at left


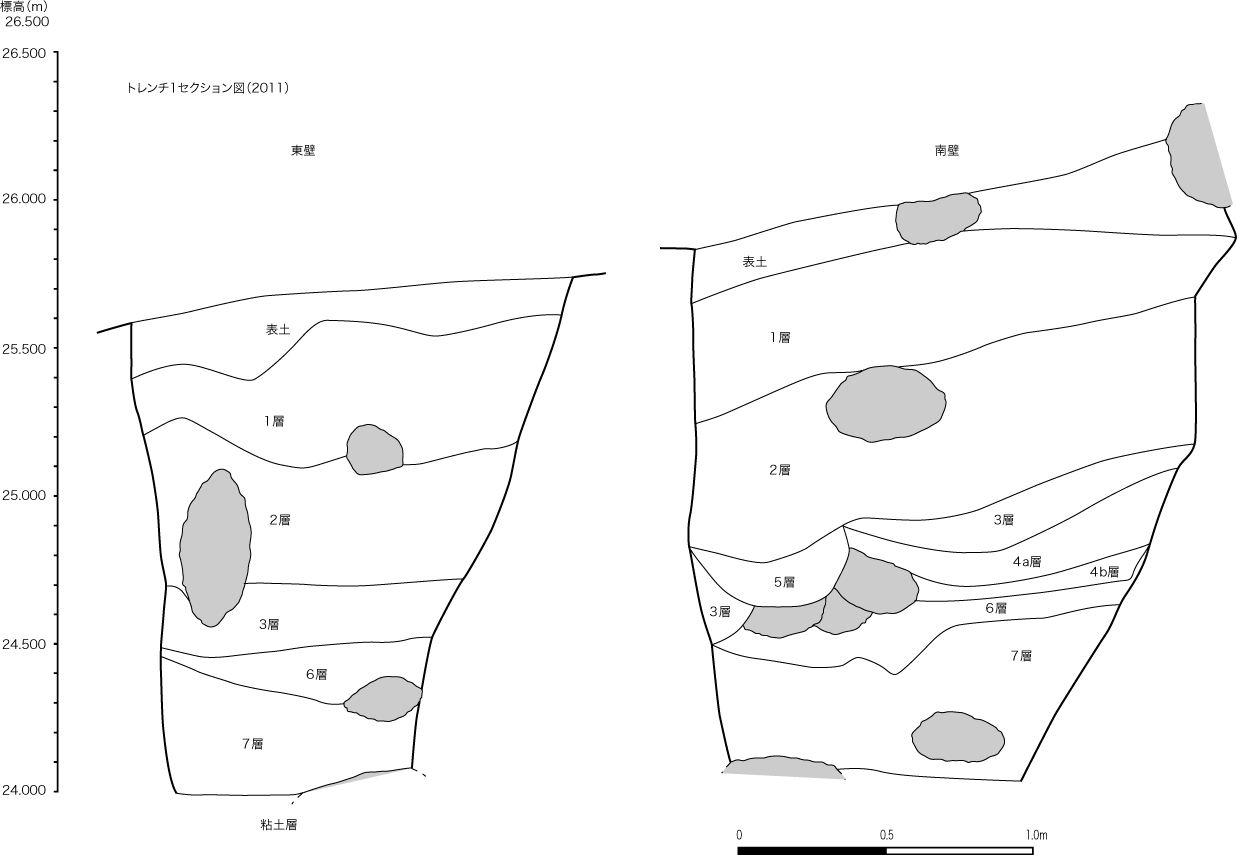


**Fig. SI 12.8.** Stratigraphy of Trench 1 showing 7 layers below the topsoil. Scale at left displays height above sea level.

Previous research on the southern Ryukyus has proposed two prehistoric cultural periods: an Early Neolithic Shimotabaru phase (ca. 2300-1500 BC) and a Late Neolithic Non-ceramic phase (ca. 800 BC – AD 1100)^[[45]](#endnote-45)^. Given the hiatus between these two phases, it is widely assumed that a new population who lacked pottery re-settled the islands during the Late Neolithic. The origins of the prehistoric peoples of the southern Ryukyus have been much debated but most archaeologists have concluded that links with Taiwan and the Austronesian world are likely^45,^^[[46]](#endnote-46),^^[[47]](#endnote-47)^. The appearance of *Tridacna* shell adzes during the Late Neolithic has suggested links with the Philippines^[[48]](#endnote-48)^.

**§6.5.2. The foraging-farming transition at Nagabaka**

Previous research has indicated that agriculture began in the Ryukyu islands ca. AD 1000 during the medieval Gusuku period^[[49]](#endnote-49)^. The spread of farming from Kyushu has been associated with the expansion of the Ryukyuan languages. Although extensive archaeobotanical research has been conducted on the Okinawa and Amami islands, in the southern Ryukyus flotation has only been carried out at three sites—Arafu^[[50]](#endnote-50)^, Mizunuma^[[51]](#endnote-51)^, and Nagabaka (this study). At Arafu, a prehistoric site dating to ca. 1000 BC - AD 400, *Rubus*, *Sambucus* and Polygonaceae seeds were the only remains identified and no cultigens were found. At Mizunuma, a medieval site, more than 300 carbonised seeds were recovered: foxtail millet (109), barley (90), wheat (31), *mugi* (barely or wheat (53)), Fabaceae (possibly the adzuki group) (16), and rice (6). ^14^C dates were barley: 1272-1309 and 1338-1397 cal. AD; and wheat: 1297-1373 and 1279-1318 cal. AD.

At Nagabaka, a total of 125.3 litres of soil were floated but only 4 seeds or fragments were recovered, two of which were identified as the pepper *Zanthoxylum ailanthoides* (Table SI 12.9 & Fig. SI 12.9). No cereal remains have been recovered from other prehistoric sites in the southern Ryukyus although such finds are common at sites from the twelfth to thirteenth centuries AD onwards.


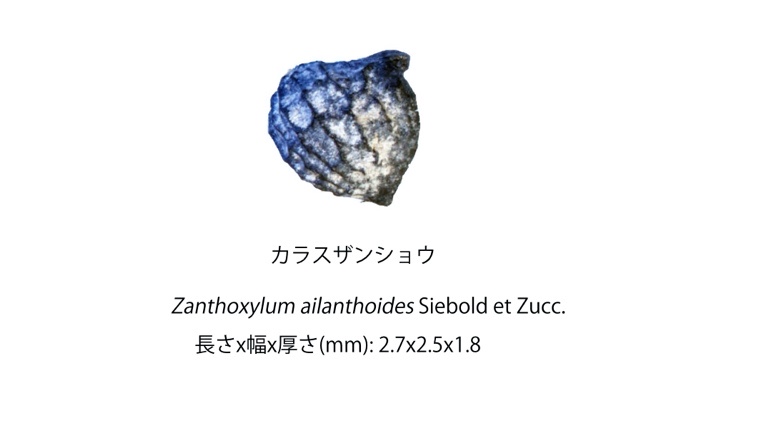


**Fig. SI 12.9.** *Zanthoxylum ailanthoides* seed from Layer 2 of the North Area Trench (seed size: 2.7 x 2.5 x 1.8 mm)

**Table SI 12.9. Soil flotation results from Nagabaka**

| **Light fraction No.** | **Sampling trench** | **Layer** | **Soil sample (*l*)** | **Light fraction (*g*)** | ***Zanthoxylum ailanthoides* Siebold et Zucc.** | **Fragments (unidentifiable)** |
| --- | --- | --- | --- | --- | --- | --- |
| 1 | North Area (south) | 2 | 6 | 4.6 | 1 |  |
| 2 | North Area (south) | 5 | 8 | 0.96 |  |  |
| 3 | North Area (south) | 2 | 5.5 | 9.76 |  |  |
| 4 | North Area (sub-trench) | 2 | 9 | 3.55 |  |  |
| 5 | North Area | 4 | 6 | 1.2 |  | 1 |
| 6 | 5 | 1 | 4 | 8.71 |  |  |
| 7 | North Area | 3-4 upper surface | 0.2 | 0.14 |  |  |
| 8 | North Area (south) | Pit 3 | 0.1 | 0.22 |  |  |
| 9 | 6 | Surface & 1 | 5 | 48.98 |  |  |
| 10 | North Area | 5 | 4.5 | 0.36 |  |  |
| 11 | North Area (sub trench north) | 3 | 4.5 | 1.1 |  |  |
| 12 | North Area (south) | 4 | 6 | 2.24 |  | 4 |
| 13 | North Area | 1 | 5.5 | 43.29 |  |  |
| 14 | North Area (sub- trench north) | 3 | 5.5 | 5.68 |  |  |
| 15 | North Area (south) | 2 | 4 | 1.99 |  |  |
| 16 | North Area (south) | 4 | 10 | 0.85 |  |  |
| 17 | North Area (sub-trench) | 2 | 4.9 | 3.09 |  |  |
| 18 | 5 | 2 | 2 | 6.7 |  |  |
| 19 | North Area (sub-trench) | 2 | 8 | 4.58 |  |  |
| 20 | North Area | 2 | 6 | 4.64 |  |  |
| 21 | 5 | Surface | 1 | 12.19 |  |  |
| 22 | North Area (sub-trench north) | 3 | 5.5 | 1.67 |  |  |
| 23 | North Area | 1 | 0.5 | 11.88 |  |  |
| 24 | North Area | 2 (charcoal layer) | 0.1 | 0.32 |  |  |
| 25 | North Area | 2 | 6 | 4.85 | 1 |  |
| 26 | North Area | 1 | 0.5 | 4.11 |  |  |
| 27 | North Area (sub-trench) | 2 | 3 | 1.28 |  |  |
| 28 | North Area (sub-trench) | 2 | 3 | 1.21 |  |  |
| 29 | 6 | 3 | 1 | 1.68 |  |  |

The Nagabaka shell midden has abundant faunal remains. A full analysis of fish bones excavated in the 2008 season found a heavy reliance on coral reef species with around 80% of the assemblage comprising Scaridae^[[52]](#endnote-52)^. Wild boar bones are found in all layers. Two dog bones were reported previously^[[53]](#endnote-53)^. A re-examination of one of those bones concluded that it is most likely a juvenile wild boar. The second bone remains a likely canine but awaits direct dating. Analysis of shellfish remains excavated from 2006-2011 reported a large sample of 6958 NISP from Trenches 1 and 2. Common shellfish varieties included Conidae, Strombidae, Tridacnidae as well as *Turbo argyrostomus*, *Geloina coaxans*, *Telescopium telescopium*, and chitons^[[54]](#endnote-54)^.

**§6.5.3. Cultural context of the Nagabaka prehistoric site**

Prior to the discovery of Nagabaka, Miyako island had only produced sites of the Late Neolithic non-ceramic phase. The lower levels at Nagabaka have radiocarbon dates overlapping with the Early Neolithic phase in the Yaeyama islands but without Shimotabaru pottery. Nagabaka is thus difficult to place within the existing archaeological chronology of the southern Ryukyus.

Only a few lithic artefacts were discovered from the prehistoric layers at Nagabaka. Two slate artefacts are morphologically similar to sickles and reaping knives known from Late Neolithic and Bronze Age societies in East Asia (Fig. SI 12.10). Both of these tools have use wear. Slate is not found on Miyako and these artefacts or the raw material must have been imported from outside the island. Another stone artefact is a grinding stone found in association with a shell adze in the North Area Trench (Figs. SI 12.11 & 12.12).


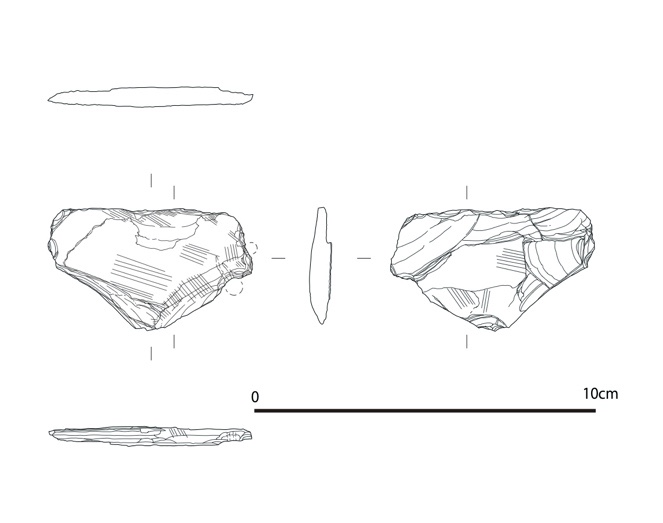

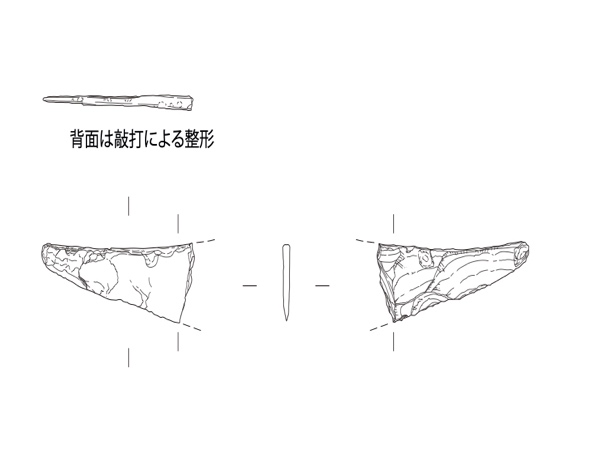


**Fig. SI 12.10.** Slate artefacts from North Area Trench, Nagabaka. Left: reaping-knife-shaped tool (61 x 36 x6 mm); right: sickle-shaped tool (remaining size: 44 x 17 x 2 mm).

**Fig. SI 12.11.** Grinding stone from North Area Trench


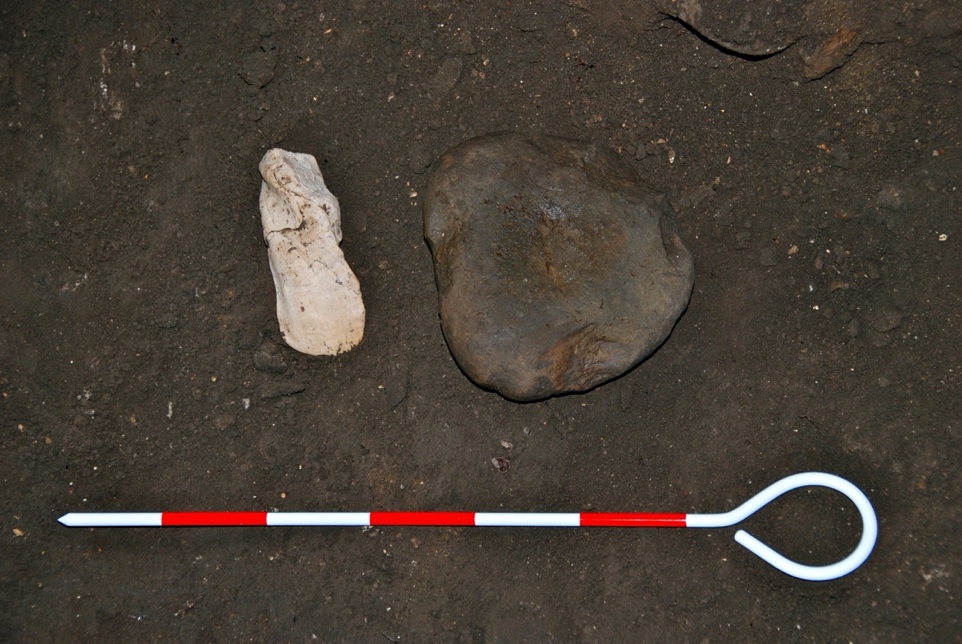


**Fig. SI 12.12.** Grinding stone (Fig. SI 12.11) found in association with a shell adze (North Area Trench).

The Nagabaka excavations produced a large number of shell artefacts including 6 shell adzes and 5 shell adze blanks (Fig. SI 12.13). These adzes are the only shell tools, except for several perforated bivalves which might be fishing net sinkers. Shell ornaments include shell beads, cowrie artefacts and shell discs. The excavations also produced 39 shark teeth, many with perforations and most from tiger shark (*Galeocerdo cuvier*). Appearing after around 800 BC, shell adzes are the most diagnostic artefact of the non-ceramic Late Neolithic phase in the southern Ryukyus but are not found in Okinawa or other islands to the north. At Nagabaka, shell adzes are not present in the lower layers but appear from Layer 3 in Trench 1, a layer which has radiocarbon dates ranging from 733 BC to AD 646. This may suggest contact with at least the Yaeyama islands in the early first millennium BC. However, the cultural relationship between the earlier layers at Nagabaka and islands outside Miyako remains unclear from the archaeological remains.


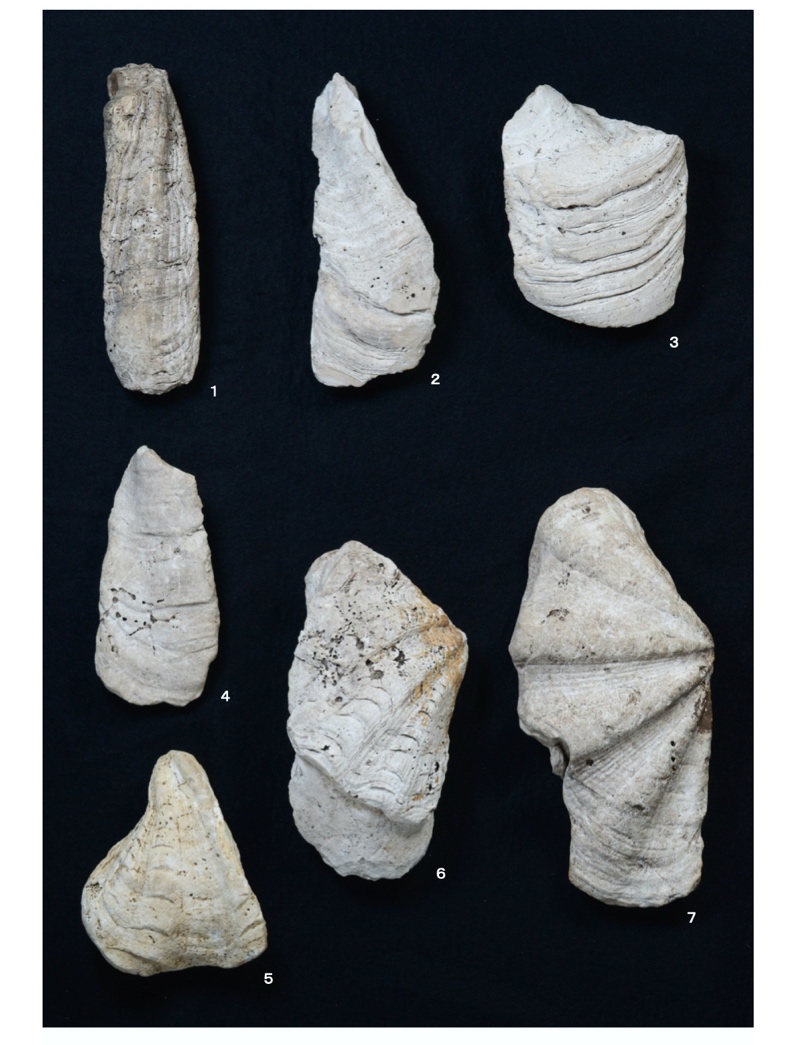


**Fig. SI 12.13.** Shell adzes from Trench 1, Nagabaka (measurements in mm). 1: Layer 2, *Hippopus hippopus* (146 x 44 x 42); 2: top of Layer 3, *Tridacna squamosa* or *T. derasa* (135 x 52 x 46); 3: Layer 2, *Hippopus hippopus* (106 x 81 x 40); 4: Layer 1, *T. squamosa* or *T. derasa* (114 x 55 x 32); 5: Layer 2, *T. derasa* (95 x 80 x 26); 6: Layer 2, *T. maxima* (148 x 79 x 27); 7: surface layer, *T. squamosa* or *T. derasa* (184 x 90 x 25).

**§6.5.4. Human skeletal remains at Nagabaka**

Although several Pleistocene sites in the Ryukyu islands have produced human skeletal remains^[[55]](#endnote-55)^, until recently there were no such remains from prehistoric Holocene sites in the southern Ryukyus. At present, three sites with human remains dating to before the medieval era are known from the Yaeyama archipelago. A left femur from the Amitori shell mound (Iriomote island) is cross-dated to the Late Neolithic. The Ōdomarihama shell mound on Hateruma island has produced a female burial with a new-born baby, cross-dated to the end of the prehistoric period in the eleventh to twelfth centuries AD^[[56]](#endnote-56)^. The Shiraho-Saonetabaru cave on Ishigaki island has human skeletal remains from dating from the late Pleistocene to the Holocene^[[57]](#endnote-57)^.

Nagabaka is the only Holocene prehistoric site in the Miyako archipelago which has produced human skeletal remains. These remains derive primarily from the shell midden deposits in Trench 1, Trench 2 and the North Area Trench. The human remains excavated from Nagabaka are currently being analysed at Kyushu University prior to their eventual return to Miyakojima City. (In addition to the remains reported here, a sample of human teeth is under analysis at Kagoshima Women’s Junior College). SI 14 provides a preliminary inventory of the human bones and teeth excavated from Nagabaka. This inventory is organised by labelled bags collected during the excavation; each bag often contains more than one bone or tooth.

Human skeletal remains were uncovered from all layers at Nagabaka. Except for a few phalanges, all bones were fragmentary. Some bones display evidence for thermal alteration. Some human bones also have spiral fractures reminiscent of processing seen on faunal remains from the site, but no cut marks have been identified. The human skeletal remains at Nagabaka were found primarily in midden deposits together with discarded food refuse including animal and fish bones and shells. While a detailed reconstruction of the techniques used for processing human remains requires further research, the same techniques seem to have been used throughout the Nagabaka sequence, a period of around 3000 years. A question for future analysis is whether some form of cannibalism, ritual or otherwise, was involved.

**§6.6. Rokutsū**

Rokutsū is a shell midden site located in Chiba City on the eastern coast of Tokyo Bay. The main occupations at the site date from the early Late Jōmon to the first half of the Final Jōmon (ca. 2500-500 BC), though pottery from the late Final Jōmon to Middle Yayoi (ca. 500 BC – AD 100) has also been found^[[58]](#endnote-58)^. Excavations in 2006 produced 7 inhumation burials. No direct radiocarbon dates are available but the skeletal remains were dated on pottery typology to the Late Jōmon and are thought to date to ca. 2000-1500 BC^[[59]](#endnote-59)^. The DNA results used here have been previously published^3^.

**§6.7. Shimomotoyama**

Shimomotoyama is a rock shelter site in Sasebo city, Nagasaki prefecture, northwest Kyushu. Facing the Akemi river, the rock shelter has a 30 m wide opening at the front and is about 4 m deep and 3 m high. Excavations in 1970 produced evidence for burials and habitation dating to the Early and Late Jōmon and the Yayoi periods. Two burials date to the Early Jōmon and have direct radiocarbon dates of 6515-6427 and 6544-6453 cal BP (1𝜎). The Early Jōmon layers produced faunal remains including wild boar, deer and pheasant. Over 1000 sherds of Early phase Sobata pottery were found as well as stone tools, fishing implements and shell rings and other ornaments. The aDNA samples used here are from a Late Yayoi stone slab-coffin burial which contained 2 individuals, a female aged 30-50 (No. 2) and a male aged over 50 (No. 3) (Fig. SI 12.14). From the stratigraphic relationship between the 2 burials, it has been suggested that the male was buried first and the female added later. The style of slab-coffin and the pottery found nearby have suggested a Late Yayoi date. However, skeleton No. 3 was radiocarbon dated to 2001-1931 cal BP (1𝜎) or 51 BC-AD 19^[[60]](#endnote-60)^, a result which implies a Middle Yayoi date. The Shimotoyama Yayoi-period skeletons have been classified as ‘Northwest Kyushu Yayoi’, a type which retains many Jōmon morphological elements. The ancient DNA data used here is taken from our recent publication in Japanese^[[61]](#endnote-61)^.


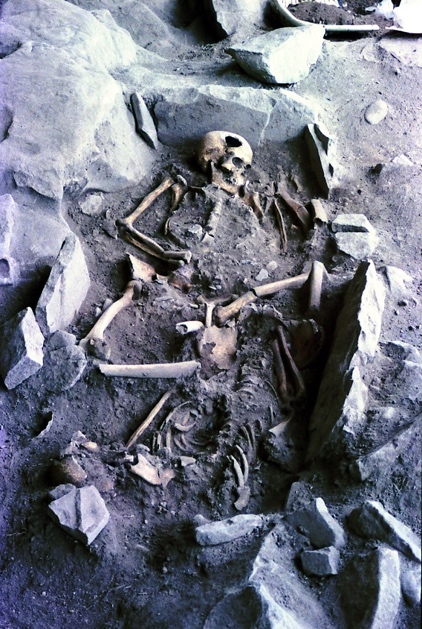


**Fig. SI 12.14.** Yayoi period slab-coffin with skeletons No. 2 and No. 3, Shimomotoyama. Source: Courtesy of Sasebo City Board of Education.

**References**

1. Ning, C. et al. Ancient genomes from northern China suggest links between subsistence changes and human migration. *Nat. Comm*. **11**, e2700 (2020). [↑](#endnote-ref-1)
2. Yang, M. et al. Ancient DNA indicates human population shifts and admixture in northern and southern China. *Science* **10**, 1126 (2020). [↑](#endnote-ref-2)
3. Wang, C.C. et al. Genomic insights into the formation of human populations in East Asia. *Nature* **591**, 413-419 (2021). [↑](#endnote-ref-3)
4. Siska, V. et al. Genome-wide data from two early Neolithic East Asian individuals dating to 7700 years ago. *Sci. Adv*. **3**, e1601877 (2017). [↑](#endnote-ref-4)
5. Jeong, C. *et al.* A dynamic 6,000-year genetic history of Eurasia’s eastern steppe. *Cell* **183**, 890-904 (2020). [↑](#endnote-ref-5)
6. Choy, K., An, D. & Richards, M.P. Stable isotopic analysis of human and faunal remains from the Incipient Chulmun (Neolithic) shell midden site of Ando Island, Korea. *J. Archaeol. Sci*. **39**, 2091-2097 (2012). [↑](#endnote-ref-6)
7. Gwangju National Museum, *Ando Shell Midden* (Kwangju: Gwangju National Museum, 2009) (in Korean). [↑](#endnote-ref-7)
8. Kim, M., Shin, H-N, Kim, S., Lim, D-J., Jo, K., Ryu, A., Won, H., Oh, S. & Noh, H. Population and social aggregation in the Neolithic Chulmun villages of Korea. *J. Anthropol. Archaeol*. **40**, 160-182 (2015). [↑](#endnote-ref-8)
9. Kim, S-H. Largest-scale cemetery of the lower Neolithic. *J. Korean Archaeol*. **2011**, 22-31 (2011). [↑](#endnote-ref-9)
10. Korea Archaeology & Art History Research Institute (eds.), *Pusan Gadŏkto Changhang Site* (Korea Archaeol. & Art Hist. Res. Inst., 2014) (in Korean). [↑](#endnote-ref-10)
11. Shinoda, K., Kanzawa, H., Kakuda, T. & Adachi, N. Kankoku Gadŏkto Janghang [Changhang] iseki shutsudo jinkotsu no DNA bunseki. *Munmul (Korea Archaeol. Art Hist. Res. Instit. J.)* **9**, 167-186 (2019). [↑](#endnote-ref-11)
12. Hudson, M.J. & Robbeets, M. Archaeolinguistic evidence for the farming/language dispersal of Koreanic. *Evol. Hum. Sci*. **2**, e52 (2020). [↑](#endnote-ref-12)
13. Yamada, Y., Takigami, M., Sakamoto, M. & Fujio, S. Kankoku Pusan-shi Gadŏkto Janghang [Changhang] iseki shutsudo shinsekki jidai jinkotsu no nendaigakuteki chōsa ni tsuite. *Munmul (Korea Archaeol. Art Hist. Res. Instit. J.)* **9**, 151-158 (2019). [↑](#endnote-ref-13)
14. Chungcheong Institute of Cultural Heritage, *Sŏsan Taejungni p'aech'ong* (Seoul: Ch'ungch'ŏng maejang munhwa jaeyŏn'guwŏn, 2000). [↑](#endnote-ref-14)
15. *Taejungni yujŏk* (Seoul: Hansŏdaehakkyo pangmulgwan, 2001). [↑](#endnote-ref-15)
16. Shin, S-C., Rhee, S-N. & Aikens, C.M. Chulmun Neolithic intensification, complexity, and emerging agriculture in Korea. *Asian Perspect*. **51**, 68-109 (2012). [↑](#endnote-ref-16)
17. Ha, I. in *Nichi, Kan kōryū no kōkogaku* (eds Kyushu Archaeol. Soc. & Ryŏngnam Archaeol. Soc.) 131-153 (Pusan: Kyushu Archaeol. Soc. & Ryŏngnam Archaeol. Soc., 2004). [↑](#endnote-ref-17)
18. Tanaka, S. & Furusawa, Y. Kanhantō to Kyūshū. *Kikan Kōkogaku* **125**, 79-84 (2013). [↑](#endnote-ref-18)
19. Bausch, I. in *The Routledge Handbook of Archaeology and Globalization* (ed Hodos, T.) 413-437 (Routledge, 2017). [↑](#endnote-ref-19)
20. Jinju National Museum, eds. *Yokchido* (Chinju: Jinju Nat. Mus., 1989). [↑](#endnote-ref-20)
21. Kimura, K. & Nakayama, K. Higashi Ajia ni kyōtsū suru yōso. *Kikan Kōkogaku* **38**, frontispiece (1992). [↑](#endnote-ref-21)
22. Naumann, N. *Japanese Prehistory: The Material and Spiritual Culture of the Jōmon Period* (Wiesbaden: Harrassowitz, 2000). [↑](#endnote-ref-22)
23. Shimazu, Y. Nichikan no bunbutsu kōryū. *Kikan Kōkogaku* **38**, 54-58 (1992). [↑](#endnote-ref-23)
24. Jinju National Museum, *Yŏndaedo Site I* (Chinju: Jinju Nat. Mus., 1993). [↑](#endnote-ref-24)
25. An, D. Dietary reconstruction through trace element (Ba, Sr, Zn) analysis of human bones from the Neolithic Yondae-do site. *J. Korean Ancient Hist. Soc.* **66**, 5-26 (2009) (in Korean with English summary). [↑](#endnote-ref-25)
26. An, D. & Lee, I. Seasonality of shellfish gathering using oxygen isotope analysis of *Crassostrea gigas* from the Neolithic Yeondae-do shell midden site, Tongyeong, Korea. *Korean J. Malacol.* **31**, 43-8 (2015) (in Korean with English summary) [↑](#endnote-ref-26)
27. Padgett, B.D. *The Bioarchaeology of Violence During the Yayoi Period of Japan* (PhD dissertation, Ohio State University, 2020). [↑](#endnote-ref-27)
28. Fujio, S., Sakamoto, M. & Takigami, M. Fukuokaken Nakagawashi Antokudai iseki shutsudo Yayoi chūki jinkotsu no nendai sokutei chōsa. *Bull. Nat. Mus. Japan. Hist*. **219**, 53-62 (2020) (in Japanese). [↑](#endnote-ref-28)
29. Shinoda, K., Kanzawa, H., Tsunoda, T. & Adachi, N. Fukuokaken Nakagawashi Antokudai iseki shutsudo Yayoi chūki jinkotsu no DNA bunseki. *Bull. Nat. Mus. Japan. Hist*. **219**, 75-84 (2020) (in Japanese). [↑](#endnote-ref-29)
30. Matsumura, H., Anezaki, T. & Ishida, H. A morphometric analysis of Jomon skeletons from the Funadomari site on Rebun island, Hokkaido, Japan. *Anthropol. Sci*. **109**, 1-21 (2001). [↑](#endnote-ref-30)
31. Hokkaidō Rebunchō Kyōiku Iinkai, *Rebunchō Funadomari iseki chōsa hōkokusho: Heisei 10 nendō hakkutsu chōsa hōkoku* (Rebun Board of Education, 2000). [↑](#endnote-ref-31)
32. Kanzawa-Kiriyama, H., Jinam, T.A., Kawai, Y., Sato, T., Hosomichi, K., Tajima, A., Adachi, N., Matsumura, H., Kryukov, K., Saitou, N. & Shinoda, K. Late Jomon male and female genome sequences from the Funadomari site in Hokkaido, Japan. *Anthropol. Sci*. **127**, 83-108 (2019). [↑](#endnote-ref-32)
33. Gakuhari, T. et al. Ancient Jomon genome sequence analysis sehds light on migration patterns of early East Asian populations. *Commun. Biol*. **3**, e437 (2020). [↑](#endnote-ref-33)
34. Mizoguchi, K. *The Archaeology of Japan: From the Earliest Rice Farming Villages to the Rise of the State* (Cambridge Univ. Press, 2013). [↑](#endnote-ref-34)
35. Shinoda, K. & Kunisada, T. Analysis of ancient Japanese society through mitochondrial DNA sequencing. *Int. J. Osteoarchaeol*. **4**, 291-297 (1994). [↑](#endnote-ref-35)
36. Shinoda, K. Ancient DNA analysis of skeletal samples recovered from the Kuma-Nishioda Yayoi site. *Bull. Nat. Sci. Mus. Tokyo D* **30**, 1-8 (2004). [↑](#endnote-ref-36)
37. Ohgaki, S. & Kurozumi, T. Historical decline of the mangrove gastropods, *Telescopium* and *Terebralia* in the Ryukyu islands and Taiwan: evidence from shell middens. *Asian Mar. Biol.* **17**, 125-135 (2000). [↑](#endnote-ref-37)
38. Hernandez, M. & Hudson, M.J. 2015. Diagnosis and evaluation of causative factors for the presence of endemic treponemal disease in a Japanese sub-tropical island population from the Tokugawa period. *Int. J. Paleopathol.* **10**, 16-25 (2015). [↑](#endnote-ref-38)
39. Shimabukuro, A. Miayko, Yaeyama shotō no furubaka. *Kōkogaku Jānaru* **552**, 17-23 (2006). [↑](#endnote-ref-39)
40. Haguenauer, C. *Origines de la civilisation Japonaise* (Paris: C. Klincksieck, 1956). [↑](#endnote-ref-40)
41. Lebra, W.P. *Okinawan Religion: Belief, Ritual and Social Structure* (Hawai‘i Univ. Press, 1966). [↑](#endnote-ref-41)
42. Ikeda, J. Craniometry of Miyako islanders, the Ryukyus. *J. Anthropol. Soc. Nippon* **82**, 150-160 (1974) (in Japanese with English summary). [↑](#endnote-ref-42)
43. Sunakawa, G., Kinjō, T. & Higa, Y. in *Okinawa no rekishi to iryōshi* (ed Ryūkyū Daigaku Igakubu Fuzoku Chiiki Iryō Kenkyū Sentā) 195-206 (Fukuoka: Kyushu Univ. Press, 1998). [↑](#endnote-ref-43)
44. Burd, W.W. *Karimata: A Village in the Southern Ryukyus* (Berkeley: unpublished typescript, 1952). [↑](#endnote-ref-44)
45. Pearson, R. *Ancient Ryukyu: An Archaeological Study of Island Communities* (Univ. of Hawai‘i Press, 2013). [↑](#endnote-ref-45)
46. Summerhayes, G.R. & Anderson, A. An Austronesian presence in southern Japan: early occupation in the Yaeyama islands. *Bull. Indo-Pac. Prehist. Assoc*. **29**, 76-91 (2009). [↑](#endnote-ref-46)
47. Hudson, M.J. in *New Perspectives in Southeast Asian and Pacific Prehistory* (ed Piper, P., H. Matsumura, H. & Bulbeck, D.) 189-199 (Canberra: ANU Press, 2017). [↑](#endnote-ref-47)
48. Asato, S. The distribution of Tridacna shell adzes in the southern Ryukyu islands*. Bull. Indo-Pac. Prehist. Assoc*. **10**, 282-291 (1991). [↑](#endnote-ref-48)
49. Takamiya, H., Hudson, M., Yonenobu, H., Kurozumi, T. & Toizumi, T. An extraordinary case in human history: prehistoric hunter-gatherer adaptation to the islands of the central Ryukyus (Okinawa and Amami archipelagos), Japan. *Holocene* **26**, 408-422 (2015). [↑](#endnote-ref-49)
50. Takamiya, H. in *Arafu iseki chōsa kenkyū I* (ed Arafu Iseki Hakkutsu Chōsadan) 105-106 (Ginowan: Arafu Iseki Chōsadan, 2003). [↑](#endnote-ref-50)
51. Manabe, A., Chida, H., Kugai, M. & Obata, H. Miyako-shi Minuzuma iseki shutsudo Gusuku doki no akkon seika. *Nantō Kōko* **38**, 51-66 (2019). [↑](#endnote-ref-51)
52. Najima, Y. in *Pai-mmi-nu-Nagabaka: The Archaeology of a Prehistoric and Early Modern Site on Miyako Island, Okinawa, Vol. 1* (ed Nagabaka Archaeology Project) 83-105 (Kanzaki: Human Activity & Sustainability Group, 2013) (in Japanese). [↑](#endnote-ref-52)
53. Takahashi, R. & Hongo, H. in *Pai-mmi-nu-Nagabaka: The Archaeology of a Prehistoric and Early Modern Site on Miyako Island, Okinawa, Vol. 1* (ed Nagabaka Archaeology Project) 106-118 (Kanzaki: Human Activity & Sustainability Group, 2013) (in Japanese). [↑](#endnote-ref-53)
54. Kobayashi, R. & Nagaya, S. in *Pai-mmi-nu-Nagabaka: The Archaeology of a Prehistoric and Early Modern Site on Miyako Island, Okinawa, Vol. 1* (ed Nagabaka Archaeology Project) 54-82 (Kanzaki: Human Activity & Sustainability Group, 2013) (in Japanese). [↑](#endnote-ref-54)
55. Matsu’ura, S. in *Interdisciplinary Perspectives on the Origins of the Japanese* (ed Omoto, K.) 181-197 (Kyoto: International Research Center for Japanese Studies, 1999). [↑](#endnote-ref-55)
56. Asato, S. & Harunari, H., eds. *The Odomarihama Shell Mound, Okinawa Prefecture, Japan* (National Museum of Japanese History, 2001) (in Japanese with English summary). [↑](#endnote-ref-56)
57. Nakagawa, R., Doi, N., Nishioka, Y., Nunami, S., Yamauchi, H., Fujita, M., Yamazaki, S., Yamamoto, M., Katagiri, C., Mukai, H., Matsuzaki, H., Gakuhari, T., Takigami, M. & Yoneda, M. Pleistocene human remains from Shiraho-Saonetabaru cave on Ishigaki island, Okinawa, Japan and their radiocarbon dating. *Anthropol. Sci*. **118**, 173-183 (2010). [↑](#endnote-ref-57)
58. Kobayashi, K., Rokutsū kaizuka no Yayoi doki. *Chiba Univ. Grad. School Hum. & Soc. Sci. Res. Proj. Rep*. **290**, 57-60 (2015). [↑](#endnote-ref-58)
59. Chibaken Kyōiku Shinkō Zaidan, eds, *Chiba Tōnanbu New Town 37: Chiba-shi Rokutsū Kaizuka* (Chiba Pref. Board of Education, 2007). [↑](#endnote-ref-59)
60. Kaifu, Y., Sakaue, K. & Kono, R.T. Early Jomon and Yayoi human skeletal remains from Shimomotoyama rock shelter, Sasebo, Nagasaki prefecture, Japan. *Anthropol. Sci. (Japan. Ser.)* **125**, 25-38 (2017) (in Japanese with English summary). [↑](#endnote-ref-60)
61. Shinoda, K., Kanzawa-Kiriyama, H., Kakuda, T. & Adachi, N. Genetic characteristics of Yayoi people in northwestern Kyushu: ancient genome analysis of human bones excavated from Shimomotoyama rock shelter, Sasebo, Nagasaki prefecture, Japan. *Anthropol. Sci. (Japan. Ser.)* **127**, 25-43 (2019) (in Japanese with English summary). [↑](#endnote-ref-61)
