# Supplementary material for: Triangulation supports agricultural spread of the Transeurasian languages
Source: Nature. 2021 Nov 10;599(7886):616–21. doi: 10.1038/s41586-021-04108-8 (PMC8612925; doi:10.1038/s41586-021-04108-8)
Supplement: Supplementary file 5 — This zipped file contains Supplementary Data Files 12–16; see Supplementary Information file for full descriptions. [file 41586_2021_4108_MOESM5_ESM.zip › 2021-02-02920E-s5/33_Eurasia3angle_synthesis_SI 13_Archaeogenetic interpretation_REV21.09.pdf]

**Supplementary Information 13**

**Archaeolinguistic interpretation of our ancient DNA analyses**

## §1. Authentication of Ancient DNA Data Generated in this Study

For the 33 initially screened individuals, we applied multiple methods to ensure the authentication of our ancient genetic data. We first tabulated the C -> T and G -> A deamination rate at 3' and 5' ends respectively. As expected, a high DNA damage rate at 5' and 3' ends was observed (SI 13 Fig. 1). Such a pattern is considered as a signature of large quantities of ancient DNA preserved in the samples. Second, we estimated the mitochondrial DNA contamination for all the ancient individuals with sufficient coverage (>10X) in this study with the Schmutzi programme[1]. Specifically, we mapped adapter-removed reads to the revised Cambridge Reference Sequence of the human mitochondrial genome (rCRS; NC_012920.1), with an extension of 500 bp at the end to preserve DNA sequences passing through the origin. We then wrapped the alignment to the circular reference genome using circularmapper v1.1[2]. The contDeam and schmutzi modules of the Schmutzi program were successively run with the world-wide allele frequency database from 197 individuals, resulting in estimated mitochondrial DNA contamination rates for each individual. We restricted our analysis to samples with mitochondrial DNA coverage higher than 10X to ensure the accuracy of the estimation. Eight out of 33 were successfully estimated, and a low mitochondrial DNA contamination was obtained (See sequencing details in SI 17). Last, we estimated the nuclear contamination rate in males based on the heterogeneity on the X chromosome. Since males only have a single copy of the X chromosome, there should be no polymorphisms on X chromosome sequences if contamination is absent. Because many individuals were low in coverage, and the contamination was not adequately estimated by the above-mentioned methods, 8 individuals with SNPs overlapping with the '1240k' data set smaller than 10,000 were directly excluded from the down-stream analyses. We further applied a PCA constructed from present-day Eurasian populations and projected the ancient Korean and Japanese individuals as well as the 40,000-year-old Tianyuan individual from China and the Hòabìnhian hunter-gatherer from Laos onto the top two variation. As is shown in SI 13 Figure 2A, 8 individuals were projected outside of the East Asian variation, beyond the positioning of Tianyuan and Hòabìnhian and shifted to the west Eurasians, indicating some degree of modern human DNA contamination. We further plotted another PCA with the damaged-restricted reads only for all the ancient individuals, in the assumption that modern human DNA does not have such characters. As observed in SI 13 Figure 2B, almost all

individuals fall onto the East Asian variation between the ancient Tianyuan/Hòabìnhian hunter-gatherers and other present-day East Asians with the exclusion of TYD004, which was directly excluded from down-stream analyses. As analyses on damage-restricted reads also rule out the endogenous DNA in large numbers, we only applied this method to the Eurasia outlier samples to leverage the resolution of the data and the contamination. Five individuals were further excluded due to the poor quality after reads filtering. After these criteria, a final total of 20 individuals were left for down-stream analyses (i.e., AND001, AND004, DAJ001^, GDI002^, GDI008, GDI009^, TYD006^, TYD007, TYJ001, NAG007, NAG012, NAG016^, NAG019, NAG035, NAG036, NAG037, NAG038, NAG039, YAK002, YAK006^; see SI 17).

To minimise the potential west Eurasian contamination that we observed in the previous analyses, we further utilised qpAdm[3] to characterise the above 20 individuals/libraries with ancient populations from both East (e.g. Rokutsu_Jomon and various ancient populations from mainland East Asia) and West Eurasians (e.g. Sintashta_MLBA and LBK_EN) as sources. If an individual/library could not be modelled by either Sintashta_MLBA or LBK_EN as one source without restricting analysis to damaged reads, then we regarded it as authenticated. Or if an individual could be well modelled by Sintashta_MLBA or LBK_EN but such a signal disappears or reaches a low level (< 5%) using damage-restricted reads, then we treated the damage-restricted data as authenticated and used it for downstream group-based analyses. With this criteria, 1 individual (GDI009^) was further removed from the final analyses as even with damaged reads alone, she can still be adequately modelled with Sintashta_MLBA or LBK_EN indicating some degree of modern West Eurasian contamination (SI 22), resulting in a final of 19 authenticated data (SI 17). However, we should note that although TYD006^ and TYD007 showed no obvious signal of West Eurasian contamination, they only have very limited data available (around 7000 SNPs overlap with the '1240K' panel) and the admixture modelling of the genetic ancestries for them should only serve as a reference.

## §2. Admixture modelling of the ancient populations in this study

We determined the genetic ancestry of the ancient individuals in this study using qpAdm[3]. qpAdm works by computing all possible statistics of the form *f*4(Left1, Left2, Right1, Right2) relating to a test population and a set of outgroups ("Right" populations) assuming that they are symmetrically and differentially related to the source populations ("Left"

populations). We used the following 7 populations as base outgroups. We used Mbuti, Onge, Iran_N, Villabruna, Karitiana, Naxi and Funadomari Jomon. We examined all the comparisons of 2-way models from the following potential source populations: Rokutsu_Jomon, Jalainur (AR_EN), Devil's Gate (Devil'sGate_N), Haminmangha (HMMH_MN), Yumin, Hongshan (WLR_MN), Lower Xiajiadian (WLR_LN), Upper Xiajiadian (WLR_BA), Yangshao (YR_MN), Longshan (YR_LN), YR_LBIA, Houli (Shandong_EN), Fujian_EN, Fujian_LN and Hanben. As a result, almost all populations from this study can be well modelled as two-way admixture between an ancestry related to Jōmon and another source related to mainland East Asians with the exception of the Late Neolithic Nagabaka individuals (Nagabaka_late) who are genetically indistinguishable from the existing Jōmon ancestry (SI 13 Fig. 8) and the Neolithic Ando individuals from Korea who are entirely derived from an ancestry related to mainland East Asians (SI 16; Fig. 3B). However, our current model lacks the resolution to distinguish the mainland East Asian ancestry. In other words, the majority of ancient populations can be well modelled (p-value > 0.05) as the second source due to the relatively homogenous genetic profile of the populations there. Almost all the ancient populations published in north China were genetically admixed by 1- or 2-way admixture of populations in the Amur River (Jalainur) and the Yellow River Basins (Yangshao). The Middle Neolithic Hongshan individuals from the West Liao River Basin geographically close to the Korean Peninsula were already genetically admixed by the Amur and Yellow River ancestry represented by the Jalainur and the Yangshao, respectively[4]. Although based on ancient genomic studies in China[4,5], it is plausible that Early Neolithic populations in the West Liao River Basin might have more of an Amur River genetic profile (see §3.1). Further studies in the region with direct ancient DNA data such as those from the Xinglongwa or Zhaobaogou cultures will provide more concrete evidence.

To quantitively assess the robustness of our models, we then re-evaluated the modelling by sequentially adding the potential source populations into the above base outgroups, the so-called "model competition", and again found the mainland East Asian source could not be distinguished very well (SI 16). We then report populations who are geographically proximal and contemporary to the test populations (Fig. 3b). Ancient individuals from the Korean Peninsula such as Ando, Yŏndaedo, Changhang and Yokchido were all dated to be older than 4200 BP were reported with WLR_MN as a proxy and Taejungni which was radiocarbon dated to be only about 2500 BP with WLR_BA as a better proxy. Archaeological and previous genetic studies had suggested that compared with the preceding Jōmon ancestry,

there was another wave of population admixture in the Japanese islands relating to mainland East Asians. Although this mainland East Asian ancestry was probably via the ancient Korean populations and they may serve as a more direct ancestry to the Yayoi groups in Japan (SI 16), again we present WLR_BA in Fig. 3b to have a direct comparison of the mainland East Asian ancestry proportions in various populations in this study (Fig. 3b).

**§3. Archaeolinguistic Interpretation of the Ancient DNA Results**

**§3.1 West Liao Region**

As the Xinglongwa (8200–7400 BP) samples included in our study failed, we do not have direct evidence about the genetic profile of the farmers in the West Liao River region in the Early Neolithic. However, there is indirect evidence suggesting that the prevalent Early Neolithic profile in the West Liao area was Amur-related and that Yellow River influence increased over time and space, i.e., from the Middle Neolithic onwards and proportional to the distance from the Yellow River region. The indirect evidence comes from the observation that hunter-gatherers from the Baikal (7000–6000 BP)[6], from the eastern steppe east of West Liao (Yumin 8400–7800 BP; ED Fig. 8, 9), from the Amur (Jalainur 6500±30 BP; ED Fig. 8, 9) and from Boisman (7500- 6000 BP; ED Fig. 8, 10) are all Amur-related; see also ED Table 1. Since the West Liao area falls within the continuum marked by these geographical locations, it is plausible that the populations here belonged to the same Amur-related gene pool.

We find high proportions of Amur-like ancestry in the Middle Neolithic Haminmangha individuals (5700-5600 BP; 88%) situated on the northern banks of the Liao River as well as in the Late Neolithic Angangxi individuals (4000 BP; 75%) situated in the Amur region to the northeast of the West Liao Region (ED Fig. 8, 9, Fig. 3b). However, the Amur component decreases at the expense of a Yellow River component with proximity to the Yellow River and elapse of time: The Middle Neolithic Hongshan (Banlashan 5400-5100 BP; 40%) to Lower Xiajiadian (Erdaojingzi 4000 BP 20%) situated to the south of the West Liao River Region show considerably lower proportions of Amur ancestry and high proportions of Yellow River ancestry. This indicates that the Amur-related genetic component was original to the West Liao River in the Early Neolithic and suggests a gradual shift towards the Yellow River genome over time and space. If the transition from hunter-gathering to farming in the West Liao River area cannot be correlated with gene flow from the Yellow River Region, then it is likely that farming developed free from external influence. This inference is

consistent with the multi-centric origins of early millet cultivation suggested in archaeobotany[7].

Since Amur-related ancestry can be traced back to the earliest domestication centre of millet in northeast China and the proposed homeland of the Transeurasian language family in the West Liao river area, it appears to be the original genetic correlate of the Transeurasian speech communities. This is corroborated by the fact that we find a genetic Amur component common in contemporary speakers belonging to the five different Transeurasian subgroups, i.e., Turkic, Mongolic, Tungusic, Koreanic and Japonic.

**§3.2 Russian Far East**

Early hunter-gatherers from the Southern Primorye, such as Devil's Gate (7750-7450 BP) and Boisman (7500- 6000 BP), show high proportions of Amur ancestry (95%) and limited admixture with Jōmon ancestries (ED Fig 7i, Fig. 3b). These ancient genomes cluster with contemporary southern Tungusic populations, such as Ulcha, Negidal and Nanai in the lower Amur Region, as well as with Nivkh people in nearby Sakhalin island (ED Fig 7i)[8,9], suggesting genetic continuity within the original Amur-related gene pool. The PCA plot in ED Fig 7i further shows that contemporary northern Tungusic populations, such as Evenki and Even cluster with surrounding Siberian populations because they received gene flow from West Eurasian related populations.

Our archaeolinguistic results confirm earlier findings about the dispersal of millet agriculture and language from the West Liao River Region via the Amur to the Southern Primorye around 5000 BP (SI 7)[10]. As this route went over the region northeast of the West Liao River and as our samples from the Middle and Late Neolithic in that region suggest high proportions of Amur ancestry (Haminmangha 5700-5600 BP: 88% and Angangxi 4000 BP: 75%), we can infer that the people bringing agriculture to the Russian Far East displayed high proportions of Amur ancestry.

Since both the incoming farmers and the local hunter-gatherers in the Primorye in the Middle Neolithic had an Amur-like genetic profile, the proposed Farming/ Language dispersal falls within the Amur gene pool. By consequence, we expect low statistical power to detect traces of genetic admixture even if population movement was involved, because the admixing of two similar Amur-like genomes would not be perceivable. This explains why in the southern Primorye both Tungusic-speaking populations and non-Tungusic speaking populations such as the Nivkh are genetically similar and continuous with the ancient

genomes in the region. Bringing the archaeolinguistic and genetic evidence together thus makes a case for imperceptible genetic admixture.

### §3.3 Eastern Steppe

Recent studies have shown that Amur-like ancestry is the dominant genetic profile in Mongolia until the Early Iron Age[11]. This is in line with our findings that the Amur gene pool in the Early Neolithic covered a large geographical area reaching from the Baikal in the West to the Primorye in the East to present-day Inner-Mongolia in the South and included hunter-gatherers as well as early farmers. This explains why Amur ancestry is the common genetic component common to all speakers of Transeurasian, while it is not limited to the Transeurasian linguistic family alone, as it also occurs in descendants from hunter-gatherer groups, such as, for instance, the speakers of Nivkh.

The non-farming populations on the Eastern steppe until the Early Iron Age, such as the Ulaanzuukh and Slab Grave people, should not be associated with Mongolic languages just because they are situated in present-day Mongolia. They may well have spoken a non-Transeurasian language. On the basis of the location of ethnolinguistic groupings, such as Khitan of the Liao Empire (907–1125), the Tabghach of the Northern Wei (386–534), the Xianbei or "Serbi" (208 BC–235 AD) and the Donghu (the first millennium BC), the source region of Proto-Mongolic has been located in the West Liao River region at the intersection of present-day eastern Mongolia and northeastern China (SI 4). It was probably not until the Late Bronze Age that the Mongolic languages started to move westwards into present-day Mongolia. This appears consistent with an isotope analysis which finds an increase in millet consumption in Mongolia after ca. 800 BC[12], although more samples are needed from central Mongolia to further test this hypothesis as the current analysis, for instance, does not include Slab Grave individuals.

The Xiongnu (ca. 300 BC-200 AD) were a multi-ethnic steppe people, dominating the area from the Ordos Plateau to the Altai Mountains to south of Lake Baikal between the third century BC and the second century AD. Genetically, the Xiongnu samples are diverse, showing various degrees of admixture between Slab Grave people from present-day Eastern Mongolia and Saka-related populations from southeastern Kazakhstan and the Tian Shan mountains. [6,13] Xiongnu subsistence was dependent on nomadic pastoralism, probably introduced from their Eastern Iranian neighbours, with a strong agricultural component relating to millet farming.[12] Evidence on the Xiongnu language is scarce, limited to some

fragmentary attestations in Old Chinese chronicles, but historical linguists agree that it was a multilingual confederation, including Turkic speakers in addition to Eastern Iranian, Yenisean, Tocharian and Mongolic elements[13],[14,15,16]. Our linguistic results (SI 4, 5) indicate that the speakers of Proto-Turkic, gradually spreading from the Ordos Plateau over the Eastern steppe and across the Altai Mountains in the Bronze Age, inherited terms related to millet agriculture from their Transeurasian ancestors in the Neolithic, while they borrowed pastoral vocabulary from Eastern Iranian or Tocharian neighbors in the West. This is consistent with the identification of the Xiongnu as partially Turkic speaking. It also suggests that the non-farming populations of the Slab Grave culture spoke a language unrelated to Proto-Turkic. In line with the Farming/Language Dispersal, a part of the non-farming ancestors of the Xiongnu may thus have shifted their native language to Proto-Turkic with the adoption of millet agriculture.

**§3.4 Korea**

By analysing the first collection of ancient genomes from the Korean Peninsula, we provide direct evidence that Jōmon ancestry was present on the Peninsula by at least 6000 years ago (SI 13 Fig. 3B). All our ancient Korean samples fall within the East Eurasian variation (ED Fig. 7). This observation echoes the admixture models in that all ancient Korean individuals can be explained by 2-way admixture — one related to Jōmon and the other mostly related to mainland East Asian millet farmers (Fig. 3B; SI 16). One individual from the Late Neolithic Yokchido site on the southern coast of Korea shows a high affinity to individuals with Jōmon ancestry (SI 13 Fig. 3) and was modelled as harbouring as much as 95% Jōmon ancestry, suggesting migration from Japan to Korea.

The Japanese islands were never isolated from the Eurasian continent and, in particular, there is considerable archaeological evidence for contact and exchange between Kyushu and southern Korea in both the Palaeolithic and Neolithic[17]. In the Neolithic, these relations are often interpreted as localised and limited exchange between deep-sea fishing groups[18]. Our present results suggest a quite different model of more extensive admixture between Jōmon and Korean Chulmun populations. However, it is presently unclear to what extent this admixture reflects extensive ancient Jōmon ancestry on the peninsula or more recent exchange. Archaeologists have so far only discussed the latter possibility. There is evidence for the exchange of pottery, obsidian, sanukite, fishing tools and ornaments between Neolithic Korea and Kyushu[13,19]. Previous research has identified 23 or 24 sites in South

Korea with evidence of interaction with Jōmon Japan and 27 Jōmon sites in Kyushu with Chulmun pottery from the peninsula[20,21]. Despite such interaction, many archaeologists have previously concluded that a basic cultural or ethnic boundary was maintained between Kyushu and Korea. Several scholars have proposed that this boundary was also *linguistic*: because of linguistic differences, 'the meaning of Jōmon pottery was not understood' on the peninsula[22]. Our results suggest such conclusions are overly simplistic. Jōmon individuals who voyaged to Korea did not necessarily make Jōmon pottery if they stayed on the peninsula for any length of time; those individuals could have simply adopted the local ceramics in use in Korean Neolithic societies.

The broader question of Jōmon genetic ancestry on the Korean peninsula requires further research. The idea of a Southeast Asian origin for Jōmon populations has a long history, drawing originally on von Eickstedt's concept of 'Palaeomongoloids' and ethnological theories of links between Austronesians and the Ainu[23,24]. By the 1970s, studies of skeletal and dental morphology were showing links between Jōmon and Southeast Asian populations[25,26], although alternative interpretations included links with Polynesians[27]. The Southeast Asian origin of the Jōmon continues to be discussed from a genomic perspective[28]. However, whatever their ultimate origins, it is likely that many Jōmon populations reached the Japanese archipelago via Korea, presenting the possibility of a very ancient Jōmon genetic heritage on the peninsula.

### §3.5. The Japanese Islands

Since the late nineteenth century, the population history of Japan has been one of the most debated problems in anthropology[29]. The so-called 'dual structure hypothesis', which proposes that Neolithic Jōmon populations admixed with Bronze Age (Yayoi period) migrants to form the modern Japanese, has been the consensus theory for the last three decades[22]. However, several unresolved issues with the hypothesis have continued to be discussed[30]. Our present study provides the clearest support so far obtained for large-scale immigration and admixture in Japanese population history. Indigenous Jōmon ancestry was very homogenous (ED Fig. 7; SI 13 Figs. 4 and 5) even though the representative samples were from different parts of Japan. All the Yayoi individuals, as well as modern Japanese including the Ryukyuans, show their affinity not only to the Jōmon but also to mainland East Asians (SI 13 Fig. 6), and were genetically modelled to contain not only Jōmon ancestry, but also large ancestry proportions of mainland East Asians, such as West Liao River farmers from west of the Korean Peninsula (Fig. 3B).

Archaeological and linguistic evidence[31], together with the mountainous topography of the Japanese archipelago, lead us to expect complex regional differences in admixture between Jōmon and immigrant Bronze Age populations. Recent work in France has used genomic analyses to highlight diverse patterns of interaction between Mesolithic hunter-gatherers and Neolithic farmers[32], and future work in Japan needs to look at regional histories of admixture in similar detail.

**§3.5.1. Northwest Kyushu and the Shimomotoyama site**

In terms of cranial morphology, the two skeletons from Shimomotoyama (Nagasaki) have been classified as belonging to a 'northwest Kyushu Yayoi type', usually understood as representing hunter-gatherer populations who continued a 'traditional' Jōmon lifestyle long after the arrival of cereal farming in north-central Kyushu[33,34]. However, our genetic analyses confirm previous findings that, notwithstanding the continued presence of Jōmon type cranial morphology, genetic admixture with Yayoi populations was already quite advanced in the region. In fact, the two Shimomotoyama individuals have more than 60% mainland East Asian related ancestry (Fig. 3B). In the light of these results, the relationship between farming and foraging economies in northwest Kyushu during the Yayoi and Kofun periods requires further research.

**§3.5.2. The Ryukyu Islands**

Several Palaeolithic sites are known in the Ryukyu islands, some with human skeletal remains[35]. Debate continues over whether this Palaeolithic settlement was from the north or south[36,37]. At most Ryukyu sites there is no evidence of settlement continuity between the Palaeolithic and Neolithic. In the northern (Amami) and central (Okinawa) islands, there seems to have been re-settlement from Kyushu in the middle Holocene[38]. In the southern (Sakishima) islands, the Shiraho-Saonetabaru site on Ishigaki has a longer sequence of human skeletal remains[39], but it is unclear if the same population continued to inhabit the island. Elsewhere in the southern Ryukyus, no Holocene sites are known until the third millennium BC. Archaeological research began in the southern Ryukyus in 1889 and over 50 prehistoric sites are currently known dating to between ca. 2300 BC and AD 1000. None of these sites have any clear archaeological evidence of contact with Okinawa or Japan to the north. Two cultural phases are usually recognised: an Early Neolithic or Shimotabaru phase dated ca. 2300 – 1200 BC and a Late Neolithic or a-ceramic phase dated ca. 800 BC to AD 1000[40]. A hiatus of perhaps 400 years between these two phases is usually understood as

reflecting a transition between two different populations. Given that no archaeological evidence exists of contact with the Jōmon world, most scholars have assumed that the Neolithic populations of the southern Ryukyus arrived from the Austronesian world to the south. Early Neolithic Shimotabaru pottery has similarities with Taiwan and an origin on that island has been seen as most probable[41,42]. The appearance of the first Early Neolithic sites in the southern Ryukyus at around the same time as the onset of Austronesian voyaging from Taiwan may suggest a link between the two processes[43]. In the Late Neolithic, pottery was no longer used or made and *Tridacna* shell adzes became the most diagnostic artefacts (see SI 12 Fig. 13). For some archaeologists this has suggested the possibility that the Late Neolithic people re-colonised the southern Ryukyus from the Philippines[44].

Our results suggest the need for a major re-evaluation of existing interpretations of the prehistory of the southern Ryukyus. Instead of an origin in Taiwan or the Philippines, the DNA of the prehistoric inhabitants of the Nagabaka site shows that they were probably originated in the Jōmon world to the north. The Okinawa islands 250 km to the north are the most likely source, but a longer voyage from Japan cannot be ruled out. As explained in SI 12, the stratigraphy of the Nagabaka site provides no evidence for a hiatus in occupation between the Early and Late Neolithic phases. We have samples from both the Early ('Nagabaka_early') and Late ('Nagabaka_late') Neolithic phases on Miyako island. Our DNA data likely supports continuity between the Early and Late Nagabaka samples (SI 13 Figs. 3 and 7); although the former was low in coverage and might be subject to some degree of West Eurasian contamination (See qpAdm admixture modelling in SI 16), the latter can be safely identified as harbouring a characteristic Jōmon genetic profile (SI 13 Figs. 3 and 8). By historic times, new ancestry ultimately related to West Liao River Basin millet farmers was added to the preceding populations, changing their genetic profile from majority Jōmon ancestry to majority millet farmer ancestry (SI 13 Fig. 9). We find no evidence of genetic contribution from Taiwan to the Nagabaka individuals (See qpAdm admixture modelling in SI 16), although we cannot rule out the possibility that other Neolithic sites in the southern Ryukyus were settled from Taiwan. The southern Ryukyu islands are extremely important as the prehistoric boundary between the Jōmon and Austronesian worlds and further research is needed to explore the population history of this region.

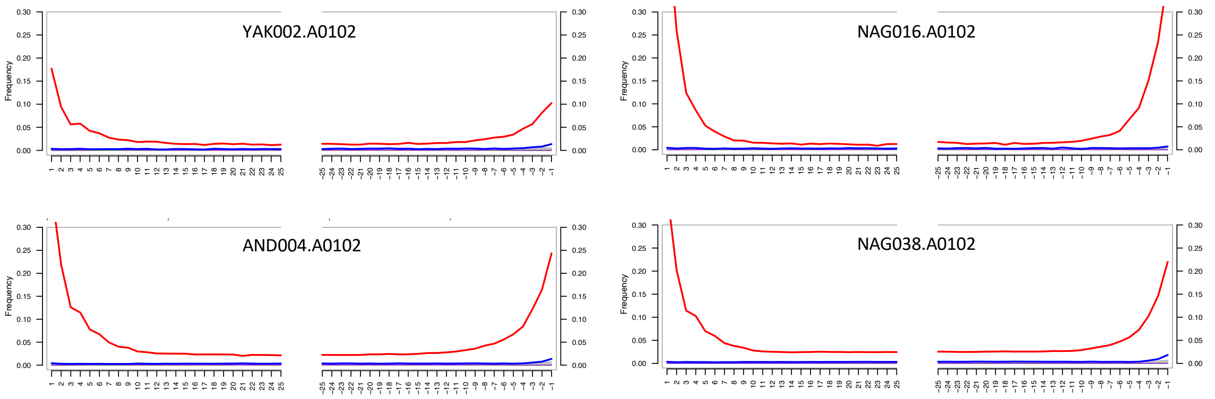


**SI 13 Fig. 1. Ancient DNA damage patterns for selected ancient individuals in this study.** The level of DNA damage is measured by the rate of cytosine deamination-based misincorporation of bases as a function of position on reads. Red and blue lines represent C>T and G>A misincorporations, respectively.

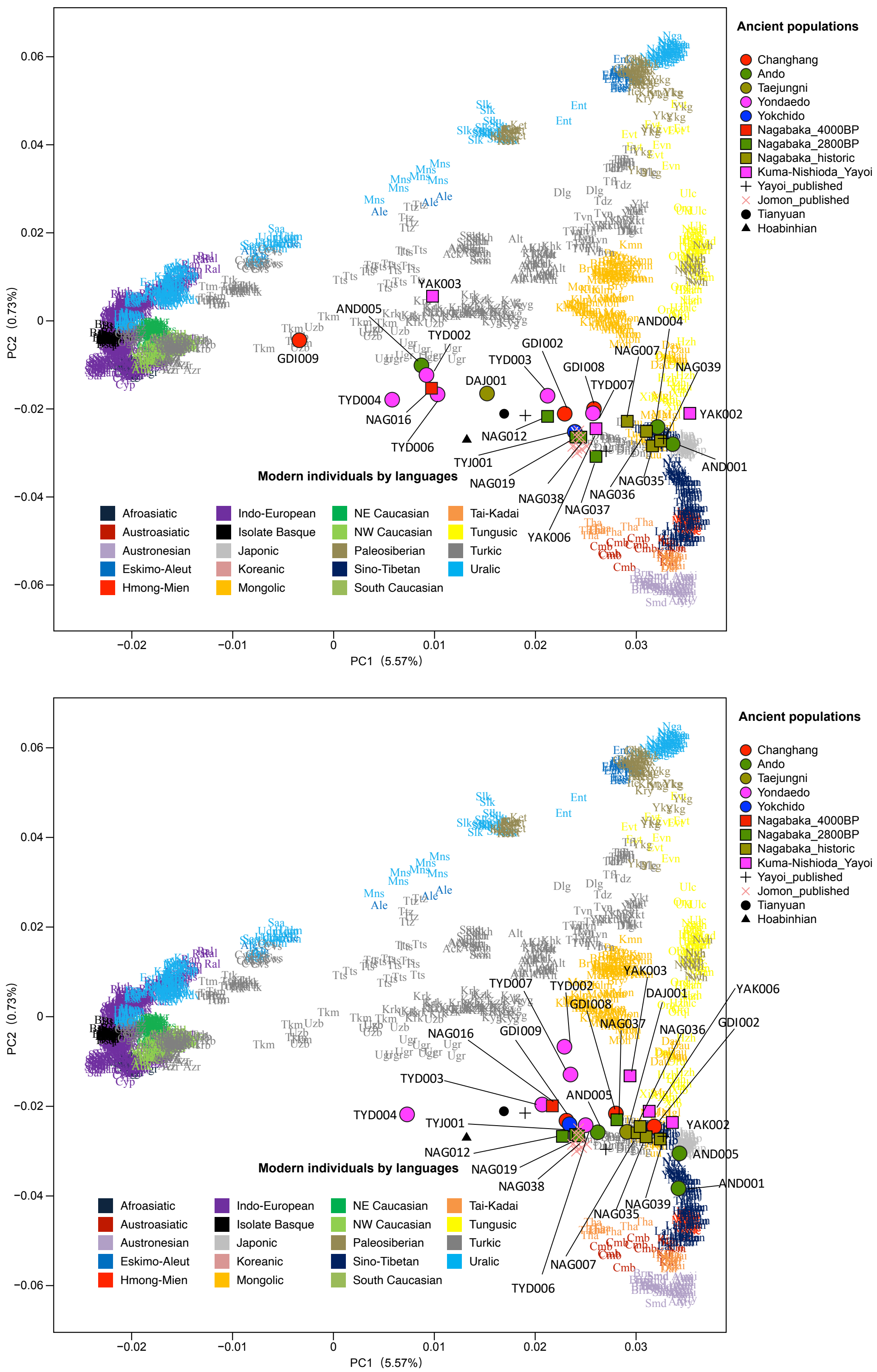


**SI 13 Fig. 2. PCA constructed from present-day Eurasian populations.** Individuals from this study were projected on the top two variations of the PCA constructed from the modern Eurasian populations.

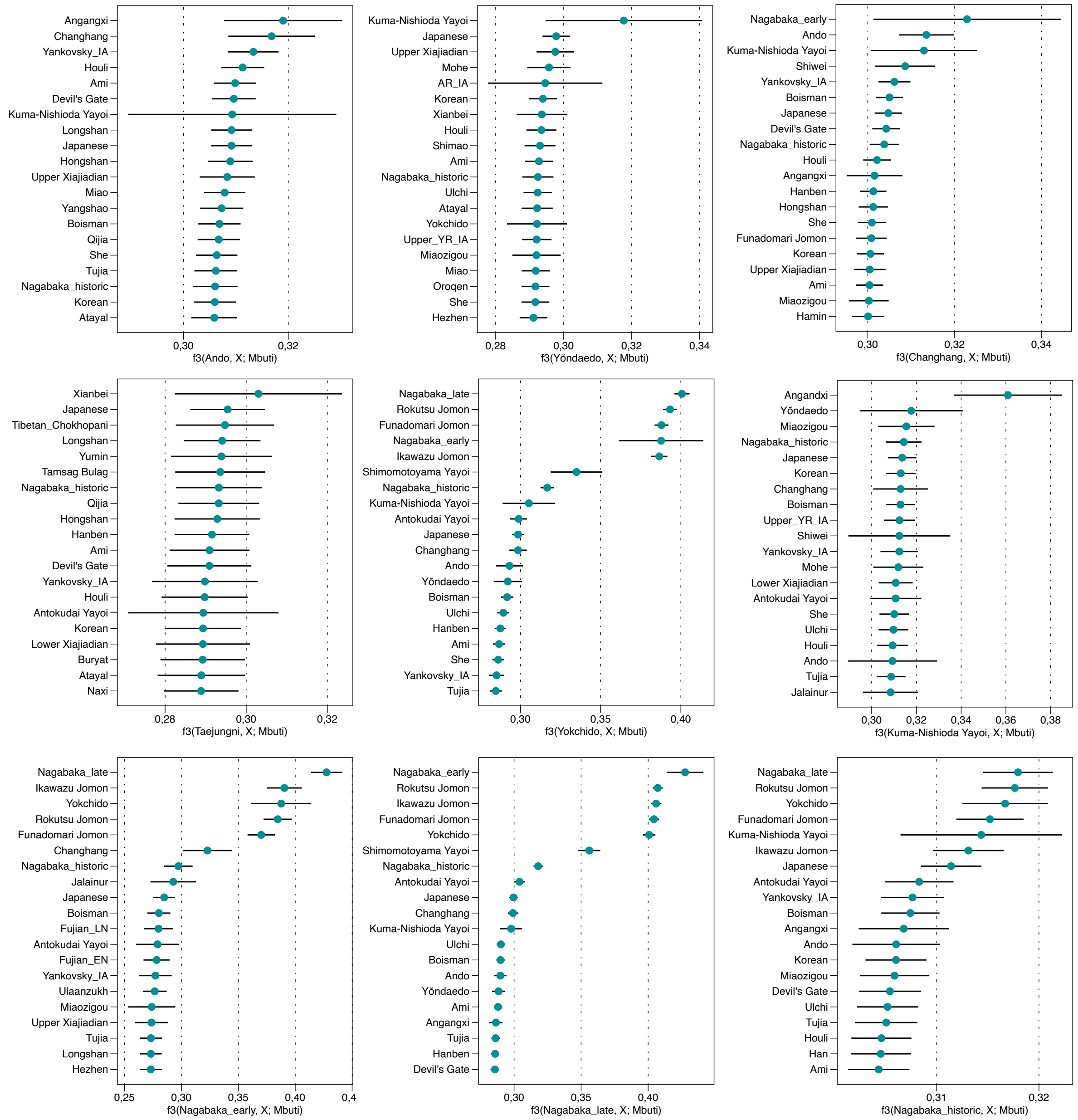


**SI 13 Fig. 3. Close genetic affinity of ancient Korean and Japanese populations to the Jōmon-related populations from Japan and ancient populations from China.** We present the top 20 outgroup-*f*3 signals for each population among the 174 non-sub-Saharan African populations. Horizontal bars represent the point estimate ± 1 s.e.m, respectively. s.e.m. are estimated using 5cM block jackknifing.

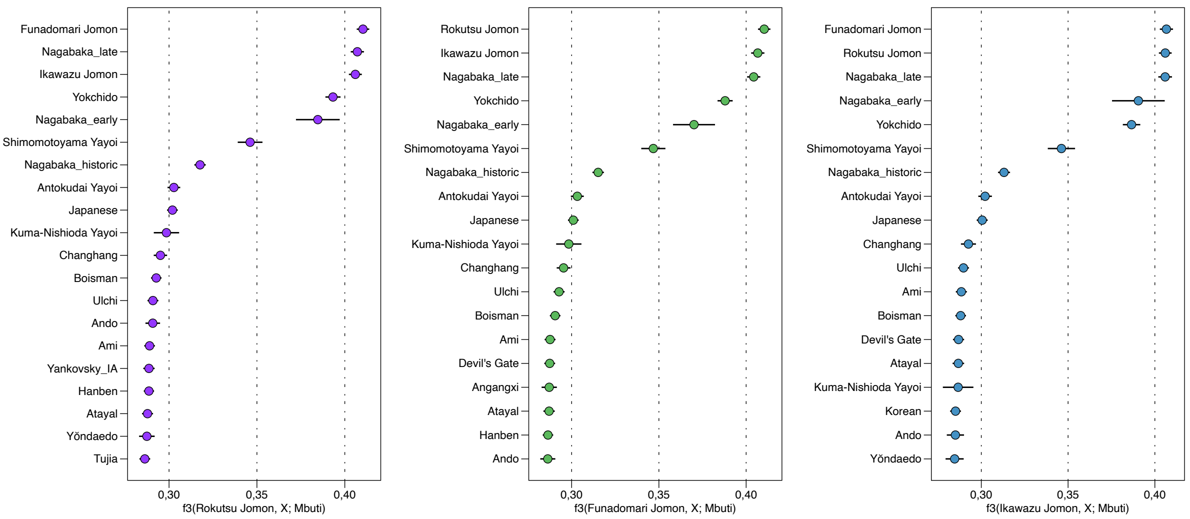


**SI 13 Fig. 4**. **Close genetic affinity between Jōmon individuals.** We present the top 20 outgroup-*f*3 signals for each population among the 174 non-sub-Saharan African populations. Horizontal bars represent the point estimate ± 1 s.e.m, respectively. s.e.m. are estimated using 5cM block jackknifing.

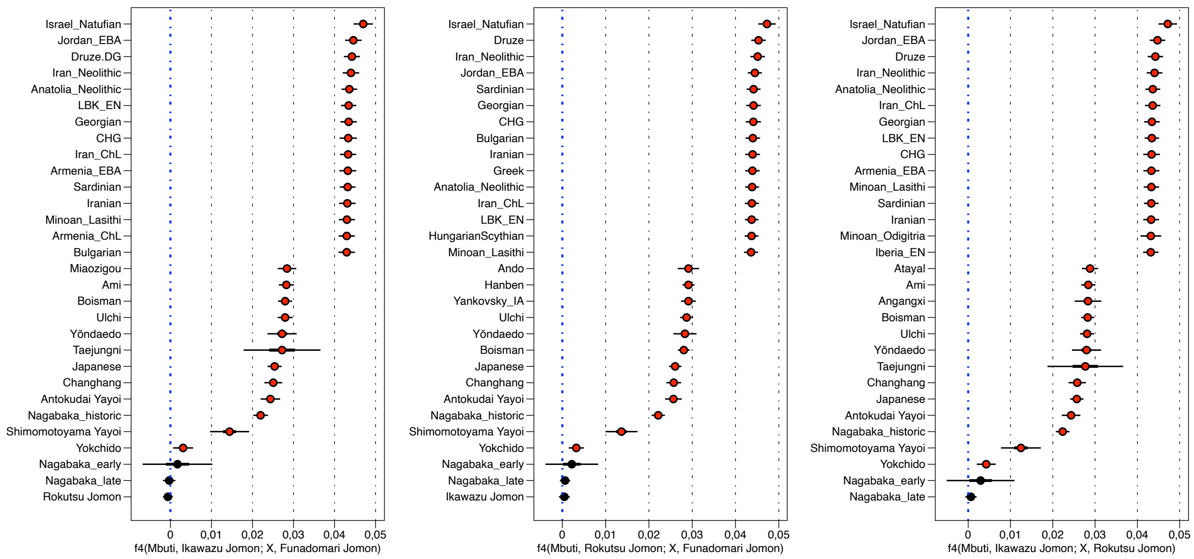


**SI 13 Fig. 5. Cladality of Jōmon individuals with respect to other modern and ancient Eurasian populations.** We present 15 most positive (upper side) and 15 most negative (lower side) *f4*(Jōmon1, X; Jōmon2, Mbuti) statistics across 174 world-wide populations. Horizontal bars represent the point estimate ± 3 (thin) and ± 1 (thick) s.e.m, respectively. s.e.m. are estimated using 5 cM block jackknifing.

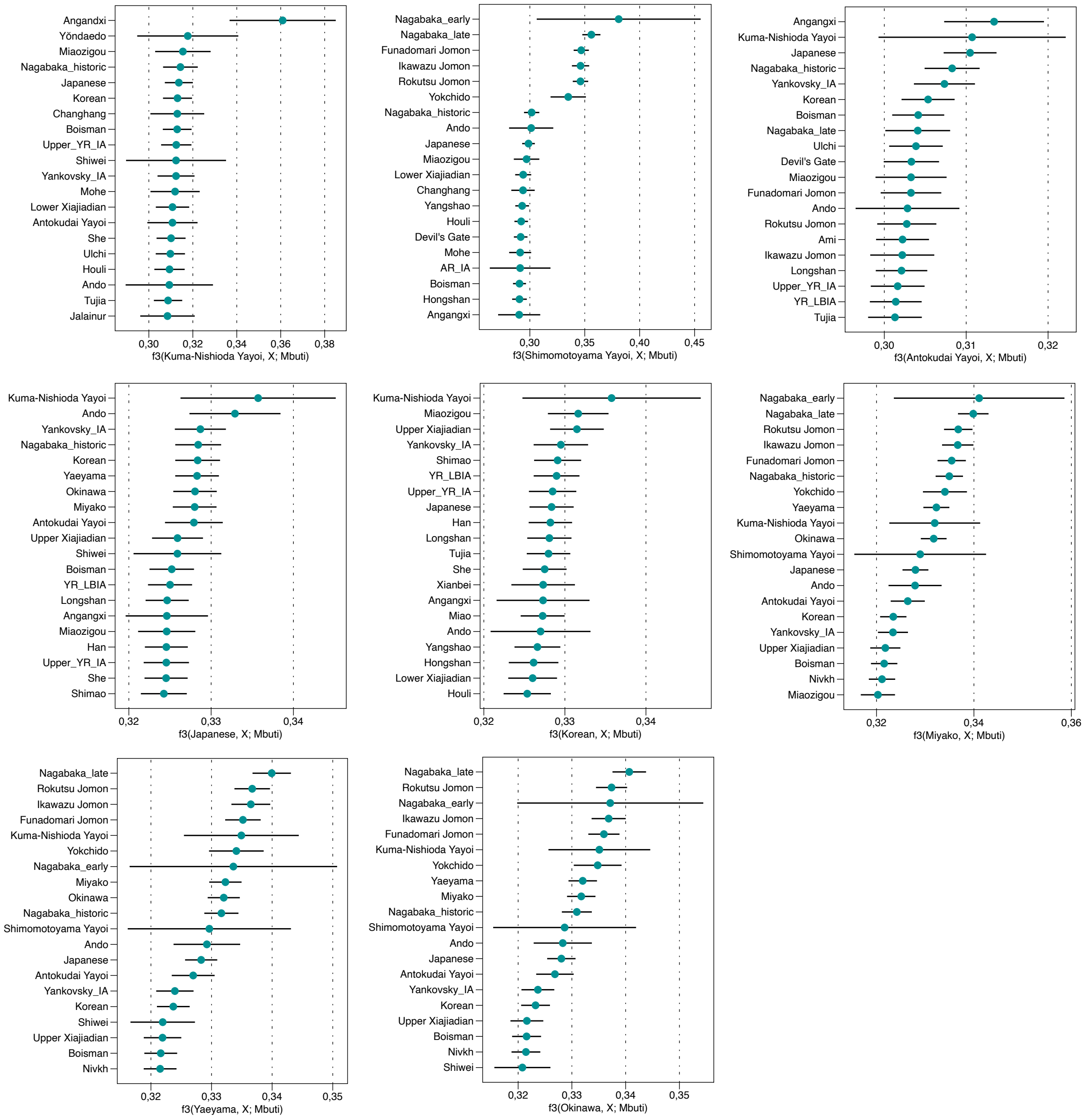


**SI 13 Fig. 6. Genetic affinity of Yayoi individuals and modern Ryukyuans from Japan.** We present the top 20 outgroup-*f*3 signals for the Yayoi populations among the 174 non-sub-Saharan African populations in the '1240k' panel. Outgroup-*f*3 signals for modern mainland Japanese and Korean as well as populations from Ryukyu islands such as Miyako, Yaeyama and Okinawa were estimated from '1240k-Illumina' panel. Horizontal bars represent the point estimate ± 1 s.e.m, respectively. s.e.m. are estimated using 5cM block jackknifing.

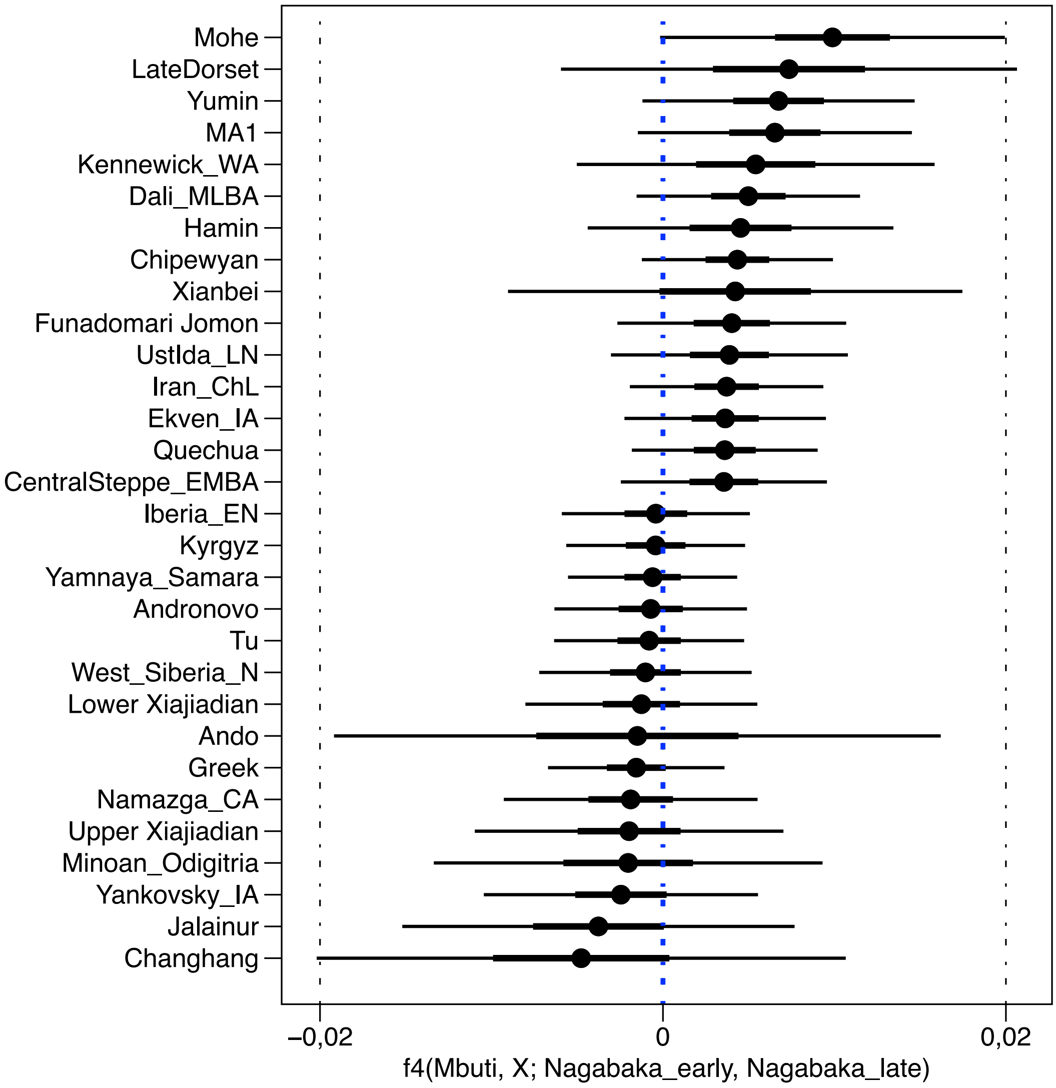


**SI 13 Fig. 7. The genetic difference between Early and Late Nagabaka individuals from Miyako island.** We present 15 most positive (upper side) and 15 most negative (lower side) *f4*(Mbuti, X; Nagabaka_early, Nagabaka_late) statistics across 174 world-wide populations. Horizontal bars represent the point estimate ± 3 (thin) and ± 1 (thick) s.e.m, respectivley. s.e.m. are estimated using 5 cM block jackknifing. *F4* statistics deviating three s.e.m. or more from zero are marked in red. No significant positive or negative statistics are shown, suggesting a general cladality of between the early and late phase of Nagabaka populations.

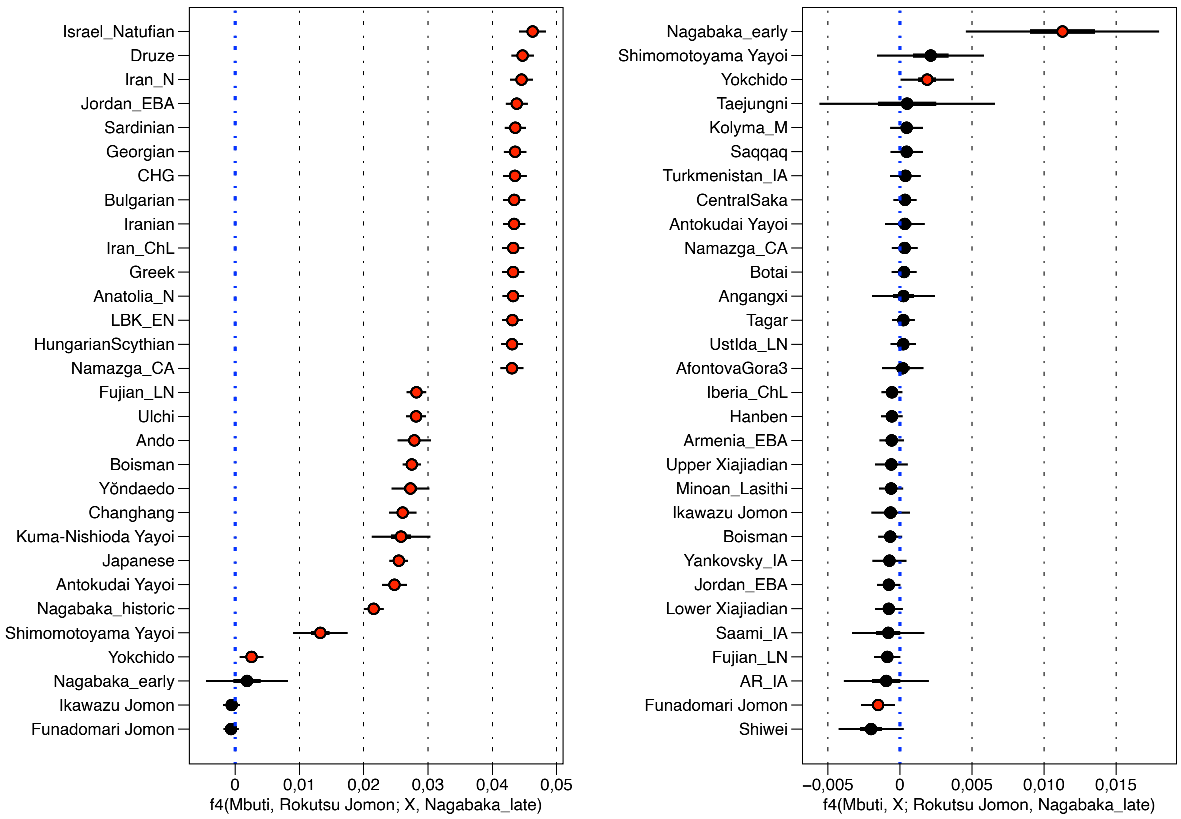


**SI 13 Fig. 8. The genetic cladality between the Late Nagabaka individuals and Jōmon.** We present 15 most positive (upper side) and 15 most negative (lower side) *f4*(Mbuti, Rokutsu Jomon; X, Nagabaka_late) statistics across 174 world-wide populations. Horizontal bars represent the point estimate ± 3 (thin) and ± 1 (thick) s.e.m, respectivley. s.e.m. are estimated using 5 cM block jackknifing. *F4* statistics deviating three s.e.m. or more from zero are marked in red.

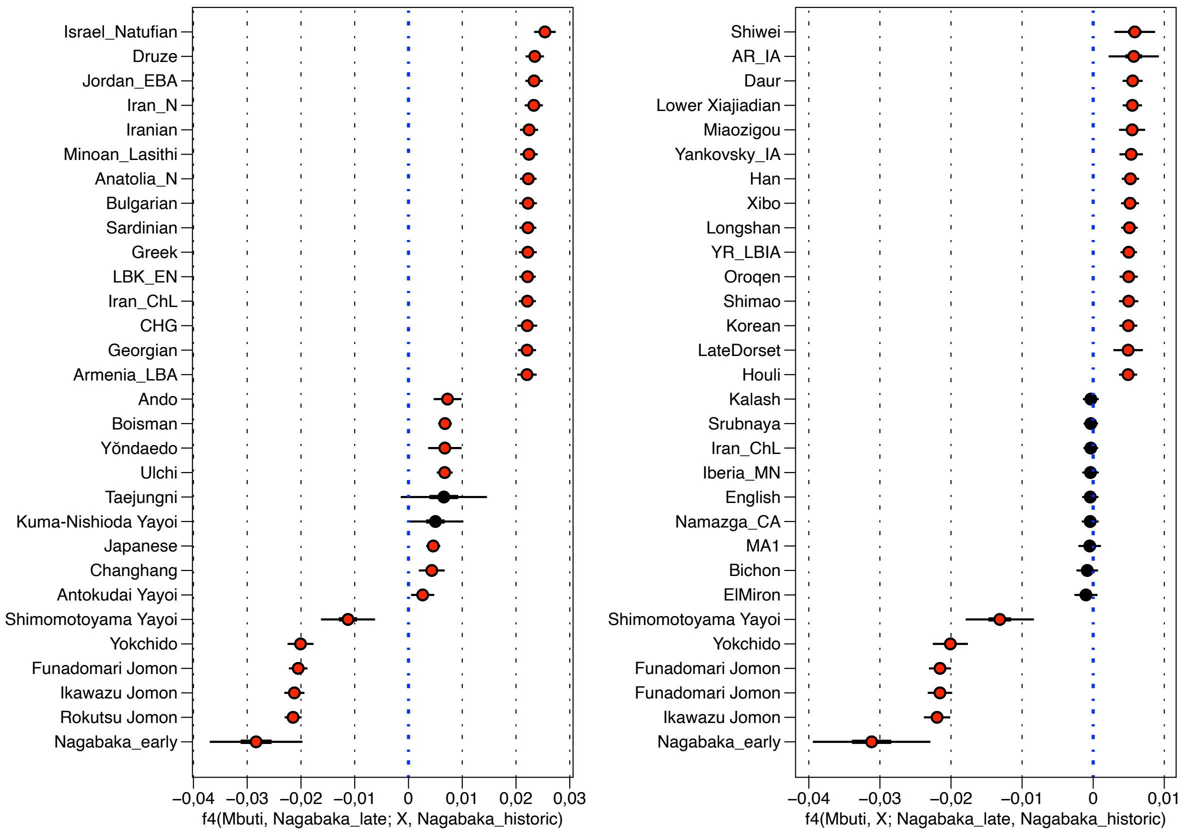


**SI 13 Fig. 9. The genetic difference between Late and historic Nagabaka individuals from Miyako island.** We present 15 most positive (upper side) and 15 most negative (lower side) *f4*(Mbuti, Nagabaka_historic; X, Nagabaka_late) and *f4*(Mbuti, X; Nagabaka_historic, Nagabaka_late) statistics across 174 world-wide populations. Horizontal bars represent the point estimate ± 3 (thin) and ± 1 (thick) s.e.m, respectivley. s.e.m. are estimated using 5 cM block jackknifing. *F4* statistics deviating three s.e.m. or more from zero are marked in red. .

## References


[1] Renaud, G., Slon, V., Duggan, A.T. & Kelso, J. Schmutzi: estimation of contamination and endogenous mitochondrial consensus calling for ancient DNA. *Genome Biol*. **16**, e224 (2015).

[2] Peltzer, A., Jäger, G., Herbig, A., Seitz, A., Kniep, C., Krause, J. & Nieselt, K. EAGER: efficient ancient genome reconstruction. *Genome Biol*. **17**, e60 (2016).

[3] Lazaridis, I. et al. Genomic insights into the origin of farming in the ancient Near East. Nature 536, 419-424 (2016).

[4] Ning, C. et al. Ancient genomes from northern China suggest links between subsistence changes and human migration. *Nat. Comm*. **11**, e2700.

[5] Yang, M.A. et al. Ancient DNA indicates human population shifts and admixture in northern and southern China. *Science* **369**, 282-288 (2020).

[6] Damgaard, P., Martiniano, R., Kamm, J., *et al*. The first horse herders and the impact of early bronze age steppe expansions into Asia. *Science* **360**, 6396, eaar7711, https://doi.org/10.1126/ science.aar 7711 (2018).

[7] Stevens, C., Shelach-Lavi, G., Zhang, H., *et al*. A model for the domestication of Panicum miliaceum (common, proso or broomcorn millet) in China. *Veg. Hist. Archaeobot.* (2020) https://doi.org/10.1007/s00334-020-00804-z

[8] Wang, C-C. & Robbeets, M. The homeland of Proto-Tungusic inferred from contemporary words and ancient genomes. *Evolutionary Human Sciences* **2**, e8 doi:10.1017/ehs.2020.8 (2020).

[9] Wang, C. C., Yeh, H. Y., Popov, A. N., *et al*. Genomic insights into the formation of human populations in East Asia. *Nature* **591**, 413-419 (2021).

[10] Li, T., Ning, C., Zhushchikhovskaya, I. S., *et al*. Millet agriculture dispersed from Northeast China to the Russian Far East: integrating archaeology, genetics and linguistics. *Archaeol. Res. Asia* **22**, 100177 (2020).

[11] Jeong, C., Wang, K., Wilkin, S., *et al*. A Dynamic 6,000-Year Genetic History of Eurasia's Eastern Steppe. *Cell* **183**, 890-904 (2020) https://doi.org/10.1016/j.cell.2020.10.015

[12] Wilkin, S. et al. Economic diversification supported the growth of Mongolia's nomadic empires. *Sci. Rep*. **10**, e3916.(2021)

[13] Savelyev, A. & Jeong, C. Early nomads of the Eastern Steppe and their tentative connections in the West. *Evol. Human Sci.* **2**, e20 (2020). doi:10.1017/ehs.2020.18

[14] Doerfer, G. *Zur Sprache der Hunnen*. CAJ **17**, 1–50 (1973).

[15] Shimunek, A. *Languages of ancient Southern Mongolia and North China: A historical-comparative study of the Serbi or Xianbei branch of the Serbi-Mongolic language family, with an analysis of Northeastern Frontier Chinese and Old Tibetan phonology* (Harrassowitz, 2017).

[16] Vovin, A., Vajda, E., & de La Vaissière, É. (2016). Who were the *kjet (羯) and what language did they speak? Journal Asiatique, 304(1), 125–144.

[17] Bausch, I. in *The Routledge Handbook of Archaeology and Globalization* (ed Hodos, T.) 413-437 (Routledge, 2017).

[18] Imamura, K. *Prehistoric Japan: New Perspectives on Insular East Asia* (London: UCL Press, 1996).

[19] Shimazu, Y. Nikkan no bunbutsu kōryū. *Kikan Kōkogaku* **38**, 54-58 (1992).

[20] Tanaka, S. & Furusawa, Y. Kanhantō to Kyūshū. *Kikan Kōkogaku* **125**, 79-84 (2013).

[21] Ha, I. in *Nichi, Kan kōryū no kōkogaku* (eds Kyushu Archaeol. Soc. & Ryŏngnam Archaeol. Soc.) 131-153 (Pusan: Kyushu Archaeol. Soc. & Ryŏngnam Archaeol. Soc., 2004).

[22] Kobayashi, T. *Jōmon bunka ga Nihonjin no mirai o hiraku* (Tokyo: Tokuma shoten, 2018).

[23] Von Eickstedt, E. *Rassenkunde und Rassengeschichte der Menschheit* (Stuttgart: Enke, 1936).

[24] Sternberg, L. The Ainu problem. *Anthropos* **24**, 755-799 (1929).

[25] Turner, C.G. Dental evidence on the origins of the Ainu and Japanese. *Science* **193**, 911-913 (1976).

[26] Hanihara, K. Dual structure model for the population history of the Japanese. *Jpn. Rev*. **2**, 1-33 (1991).

[27] Brace, C.L., Brace, M.L., Leonard, W.R. Reflections of the face of Japan: a multivariate craniofacial and odontometric perspective. *Am. J. Phys. Anthropol*. **78**, 93-113 (1989).

[28] Gakuhari, T. et al. Ancient Jomon genome sequence analysis sheds light on migration patterns of early East Asian populations. *Comm. Biol*. **3**, e437 (2020).

[29] Hudson, M.J. *Ruins of Identity: Ethnogenesis in the Japanese Islands* (Univ. Hawai'i Press, 1999).

[30] Hudson, M.J., Nakagome, S. & Whitman, J.B. The evolving Japanese: the dual structure hypothesis at 30. *Evol. Hum. Sci*. **2**, e6 (2020).

[31] De Boer, E., Yang, M.A., Kawagoe, A. & Barnes, G.L. Japan considered from the hypothesis of farmer/language spread. *Evol. Hum. Sci*. **2**, e13 (2020).

[32] Rivollat, M. et al. Ancient genome-wide DNA from France highlights the complexity of interactions between Mesolithic hunter-gatherers and Neolithic farmers. *Sci. Adv*. **6**, e5344 (2020).

[33] Nakahashi, T. in *Interdisciplinary Perspectives on the Origins of the Japanese* (ed Omoto, K.) 127-142 (Kyoto: International Research Center for Japanese Studies, 1999).

[34] Hoover, K. & Hudson, M.J. Resilience in prehistoric persistent hunter-gatherers in northwest Kyushu, Japan as assessed by population health and archaeological evidence. *Quat. Int.* **405(B),** 22-33 (2016).

[35] Kaifu, Y., Fujita, M., Yoneda, M. & Yamasaki, S. in *Emergence and Diversity of Modern Human Behavior in Paleolithic Asia* (ed Kaifu, Y., Izuho, M., Goebel, T., Sato, H. & Ono, A.) 345-361 (Texas A&M Univ. Press, 2015).

[36] Kaifu, Y. et al. Palaeolithic seafaring in East Asia: testing the bamboo raft hypothesis. *Antiquity* **93**, 1424-1441 (2019).

[37] Kaifu, Y., Kuo, T-H., Kubota, Y. & Jan, S. Palaeolithic voyage for invisible islands beyond the horizon. *Sci. Rep*. **10**, e19785 (2020).

[38] Takamiya, H., Hudson, M., Yonenobu, H., Kurozumi, T. & Toizumi, T. An extraordinary case in human history: prehistoric hunter-gatherer adaptation to the islands of the central Ryukyus (Okinawa and Amami archipelagos), Japan. *Holocene* **26**, 408-422 (2015).

[39] Nakagawa, R., Doi, N., Nishioka, Y., Nunami, S., Yamauchi, H., Fujita, M., Yamazaki, S., Yamamoto, M., Katagiri, C., Mukai, H., Matsuzaki, H., Gakuhari, T., Takigami, M. & Yoneda, M. Pleistocene human remains from Shiraho-Saonetabaru cave on Ishigaki island, Okinawa, Japan and their radiocarbon dating. *Anthropol. Sci*. **118**, 173-183 (2010).

[40] Pearson, R. *Ancient Ryukyu: An Archaeological Study of Island Communities* (Univ. of Hawai'i Press, 2013).

[41] Summerhayes, G.R. & Anderson, A. An Austronesian presence in southern Japan: early occupation in the Yaeyama islands. *Bull. Indo-Pac. Prehist. Assoc*. **29**, 76-91 (2009).

[42] Hudson, M.J. 'Austronesian' and 'Jōmon' identities in the Neolithic of the Ryukyu islands. *Doc. Praehist.* **29**, 257-262.

[43] Hudson, M.J. in *New Perspectives in Southeast Asian and Pacific Prehistory* (ed Piper, P., H. Matsumura, H. & Bulbeck, D.) 189-199 (Canberra: ANU Press, 2017).

[44] Asato, S. The distribution of Tridacna shell adzes in the southern Ryukyu islands. *Bull. Indo-Pac. Prehist. Assoc*. **10**, 282-291 (1991).
