# Supplementary material for: Triangulation supports agricultural spread of the Transeurasian languages
Source: Nature. 2021 Nov 10;599(7886):616–21. doi: 10.1038/s41586-021-04108-8 (PMC8612925; doi:10.1038/s41586-021-04108-8)
Supplement: Supplementary file 5 — This zipped file contains Supplementary Data Files 12–16; see Supplementary Information file for full descriptions. [file 41586_2021_4108_MOESM5_ESM.zip › 2021-02-02920E-s5/35_Eurasia3angle_synthesis_SI 15_isotope.pdf]

Supplementary Information 15

Isotope Analyses of Key Samples Included in this Study

We conducted stable carbon ($\delta^{13}C$) and nitrogen ($\delta^{15}N$) isotope analysis of human and faunal bone collagen and stable carbon and oxygen ($\delta^{18}O$) isotope analysis of human and faunal tooth enamel from two sites studied for DNA in this study. The two sites are Pai-mmi-nu-Nagabaka (hereafter abbreviated as Nagabaka), Miyako Island, Japan and Taejungni, Korea. The aim was to track the potential spread of crops (e.g. millet, rice) in human diets as well as to better understand the potential marine reservoir effect that might be impacting radiocarbon dates.

§1. Background

Human tissues, such as bone collagen or tooth enamel, reflect the isotopic values of consumed food macronutrients with an offset arising from isotopic fractionation[1]. Through controlled feeding experiments on humans or animals and from studies on natural consumers with monodiets or quasi monodiets the specific contribution from food macronutrients to a particular tissue and the magnitude of the isotopic offset are estimated[2,3,4]. In the case of human bone collagen, its $\delta^{13}C$ and nitrogen $\delta^{15}N$ values are primarily determined by those respective stable isotope ratios in dietary protein[2]. In contract, $\delta^{13}C$ values in tooth enamel, and also in bone carbonate, are defined by the dietary carbon mix, that is, the mix of all food macronutrients (protein, carbohydrates and lipids)[2]. Given that the isotopic offsets between food macronutrients for both tooth enamel and bone collagen have been estimated it then becomes possible to add these to know $\delta^{13}C$ values in food protein to generate reference isotopic ranges that can then be compared with archaeological measurements for the purpose of dietary reconstruction. Of particular interest here is the assessment of the dietary contributions from C3 vs. C4 plants. These two groups of plants follow different chemical pathways during photosynthesis and present distinctive $\delta^{13}C$ values[5]. Vegetation following a C3 pathway (e.g. trees, shrubs, crops such as wheat, rice or barley) is usually associated to less arid environmental conditions whereas C4 grasses (e.g. millet) are typically associated with drier conditions. For the sub-tropical climate of the Ryukyu Islands it is expected that the dominant vegetation will be C3 and animals consuming this vegetation are also classified as C3. The $\delta^{13}C$ values for these C3 plant and animal protein sources can be contrasted with those from millet, a C4 crop introduced into the Ryukyus. However, the $\delta^{13}C$ values for C4 vegetation overlap with those from marine foods[6]. To separate

respective dietary contributions, nitrogen stable isotopes can be employed. There is a direct relationship between trophic level position and $\delta^{15}N$ values[4]. Given that marine ecosystems have a larger number of trophic levels, the $\delta^{15}N$ values for marine fish or mammals are typically considerably higher than those from terrestrial plants or animals[4]. Furthermore, $\delta^{15}N$ values can also be employed to separate the dietary contributions of terrestrial plants and animals.

Since we lack an exact food isotopic baseline for the Ryukyu Islands, we relied instead on previously published food reference values from comparable environmental conditions and values for diet to consumer offsets. We took reference values for $\delta^{13}C$ endpoints associated to the consumption of C3 ($\delta^{13}C$ = -6‰) and C4 ($\delta^{13}C$ = -20.4‰) plant protein[7]. The $\delta^{13}C$ for protein from C3 herbivores was estimated ($\delta^{13}C$ = -20.4‰) by adding a herbivore to diet isotopic offset[8]. The human $\delta^{15}N$ endpoint values for full reliance on either C3 or C4 plants were assumed to match those of herbivores ($\delta^{15}N_{herbivores}$ = 4.7±1.0‰)[7], plus a correction ($\delta^{15}N_{human}$ = 6.7‰) to adjust for metabolic differences between humans and herbivores[9,10]. The human endpoint for full reliance on C3 animal products was assumed to match those of carnivores plus an isotopic correction ($\delta^{15}N_{human}$ = 10‰)[7]. In the case of marine endpoints one must consider the possibility of human consumption of both lower (e.g. molluscs) and higher (e.g. carnivorous fish, mammals) trophic level foods and wide ranges for carbon ($\delta^{13}C$ = -9±2.8‰) and nitrogen ($\delta^{15}N$ = 16.8±5.9‰) consumer endpoints were taken as reference[7]. In the case of endpoints without a given uncertainty, we employed conservative uncertainties of 1.5‰ to account for environmental effects in local isotopic baselines. The isotopic values for food endpoints, and their respective uncertainty, are shown in Figure SI 15.1. The reference endpoints for tooth enamel $\delta^{13}C$ differ from those of collagen given their different carbon source (protein vs. carbon mix) and diet to tissue offset. For a full reliance on C3 food we relied on the value (14.8‰) given by Froehle et al. (2012) in their compilation of data from omnivorous mammals[3]. We defined a single $\delta^{13}C$ value (-1.3‰) reference in tooth enamel for consumption of C4/marine foods established from known $\delta^{13}C$ values for C4 vegetation and marine foods and marine mammals, fish and shellfish in coastal areas[11,12]. To both C3 and C4/marine reference endpoints we also associated an uncertainty of 1.5‰.

§2. Stable isotope analysis: laboratory methods

Collagen

We sampled the bone/dentine collagen from 10 human individuals from Nagabaka and one individual from Taejungni for $\delta^{13}C$ and $\delta^{15}N$ analysis and the tooth enamel of 14 human individuals from Nagabaka for $\delta^{13}C$ and $\delta^{18}O$ analysis (Table S1 15.1). Four Nagabaka samples were analysed at the University of Tokyo (UT), Japan; all other samples were analysed at the Max Planck Institute for the Science of Human History (MPI-SHH), Jena, Germany.

For the UT samples, the extraction method of collagen was based on the gelatinisation method[13]. First, a cleaned piece of compact bone was reacted with 0.2 M NaOH for 18 hours, and then washed with pure water and lyophilized. The piece was crushed into fine powder in a metal mortar. Demineralisation was conducted with 1.2 M HCl for 20 hours at the temperature of 4ºC in a cellulose tube, and residual organic matter was washed with pure water until neutral pH. Remaining organic matter was heated at 90ºC in acidified water (pH 4) for 22 hours to extract gelatin, and dissolved gelatin was purified by a Whatman GF/F filter and lyophilized. The weight of extracted gelatin was recorded and a fraction was applied for the following analyses.

For the MPI-SHH samples, all specimens were first externally cleaned using a sandblaster. We then sampled *c.* 0.5 g of dentine or bone powder using a handheld Dremel drill. We extracted collagen from the powder using a modified Longin methodology[13]. Samples were demineralised through immersion in 0.5M HCl for 1-7 days. When demineralisation was complete, samples were rinsed with ultra-pure H_2O three times. The residue was gelatinized in pH3 HCl at 70°C for 48 hours and Ezee-filters were used to remove insoluble residues from the soluble collagen solution[14]. Samples were lyophilized for 48hrs.

1.0 mg of the resulting purified collagen was weighed in duplicates into tin capsules for analysis. The $\delta^{13}C$ and $\delta^{15}N$ ratios of the weighed-out bone collagen were measured using a Thermo Scientific Flash 2000 Elemental Analyser coupled to a Thermo Delta V Advantage Isotope Ratio Mass Spectrometer at the Stable Isotope Laboratory of the Department of Archaeology, MPI-SHH. Values are reported as the ratio of the heavier isotope to the lighter isotope ($^{13}C/^{12}C$ or $^{15}N/^{14}N$) as δ values in parts per mill (‰) relative to international standards,

VPDB for $\delta^{13}C$ and atmospheric N2 (AIR) for $\delta^{15}N$. The results reported for the samples were calibrated against international standards of (IAEA-CH-6: $\delta^{13}C$ = -10.80 ± 0.47 ‰, IAEA-N-2: $\delta^{15}N$ = 20.3 ± 0.2 ‰, and USGS40: $\delta^{13}C$ = -26.38 ± 0.042 ‰, $\delta^{15}N$ = 4.5 ± 0.1 ‰). Machine error was determined using repeat runs of a laboratory standard (fish gelatin: $\delta^{13}C$ = ~ -15.1 ‰, $\delta^{15}N$ = ~14.3 ‰). Based on replicate analyses machine error over the course of a year is ± 0.2‰ for $\delta^{13}C$ and ± 0.2‰ for $\delta^{15}N$. Overall measurement precision was studied through the measurement of repeats of fish gelatin (n= 80, ± 0.2‰ for $\delta^{13}C$ and ± 0.2‰ for $\delta^{15}N$).

Tooth enamel

All external sampled tooth surfaces were cleaned using air-abrasion. Gentle abrasion with a diamond-tipped drill along the full length of the buccal surface was performed to obtain a bulk sample of enamel powder representative of the complete period of enamel formation. All enamel powder was pretreated to remove organic or secondary carbonate contaminates involving first a was in 1.5% sodium hypochlorite for 60 minutes, followed by three rinses in purified H_2O and centrifuging, before 0.1M acetic acid was added for 10 minutes, followed by another three rinses in purified H_2O. The remaining residues were then subjected to lyophilization for 24 hours.

Gases were evolved from the samples using 100% phosphoric acid. The resulting gases were analyzed for their stable carbon and oxygen isotope composition using a Thermo Gas Bench 2 connected to a Thermo Delta V Advantage Mass Spectrometer at the MPI-SHH. $\delta^{13}C$ and $\delta^{18}O$ values were compared against International Standards (IAEA-603 ($\delta^{13}C$ = 2.5; $\delta^{18}O$ = -2.4); IAEA-CO-8 ($\delta^{13}C$ = -5.8; $\delta^{18}O$ = -22.7); USGS44 ($\delta^{13}C$ = -42.2)) and in-house standard (MERCK ($\delta^{13}C$ = -41.3; $\delta^{18}O$ = -14.4)). Replicate analysis of MERCK standards suggests machine measurement error to be *c.* ± 0.1‰ for $\delta^{13}C$ and ± 0.2‰ for $\delta^{18}O$. Overall measurement precision was studied through the measurement of repeat extracts from a bovid tooth enamel standard (n = 20, ± 0.2‰ for $\delta^{13}C$ and ± 0.3‰).

§3. Stable isotope results

Table SI 15.1 shows the bone collagen stable isotope results for the Nagabaka site, Miyako Island, Okinawa. For comparison, we also included previously published results for the Korean archaeological sites of Changhang and Taejungni[15,16]. In Figure SI 15.1 we compare the distribution of isotopic results with reference protein end points.

Table SI 15.1. Results of stable carbon and nitrogen isotope measurements in human bone collagen for the site of Nagabaka and previously published Changhang and Taejungni.

**ID**	$\delta^{13}C_{coll}$ (‰ – VPDB)	$\delta^{15}N_{coll}$ (‰ – AIR)	**Lab**	**Site**
4	-17.1	8.2	UT	Nagabaka
5	-16.6	9.0	UT	Nagabaka
6	-11.4	9.0	UT	Nagabaka
21	-13.5	12.3	UT	Nagabaka
29	-15.4	8.2	MPI-SHH	Nagabaka
32	-13.8	8.4	MPI-SHH	Nagabaka
33	-13.5	9.1	MPI-SHH	Nagabaka
36	-13.1	12.4	MPI-SHH	Nagabaka
37	-15.7	9.6	MPI-SHH	Nagabaka
39	-17.0	9.0	MPI-SHH	Nagabaka
KRKJH-2	-15.3	14.1	Paleo-Lab (Japan)	Changhang
KRKJH-8	-12.9	15.7	Paleo-Lab (Japan)	Changhang
MAMS-39297	-15.4	10.5	MPI-SHH	Taejungni
S-EVA-16412	-15.4	10.6	MPI-EVA	Taejungni

Of particular interest to our study are individuals with higher marine protein intakes which are likely to pre-date the introduction of farming in the Ryukyus. This is notably the case for individuals 21 and 36 from Nagabaka. The two individuals from Changhang were heavy marine consumers. Also relevant for our study are those individuals with high C4 plant consumption which was likely millet and thus these individuals should date after the introduction of farming to Miyako Island in the medieval period. In Figure SI 15.1 we can observe a protein line connecting the C3 and C4 endpoints indicating that individuals along that line had mixed C3/C4 diets with highest consumption of C4 plants observed for individuals 6, 32, and 33. The two individuals from Taejungni also had a predominately mixed C3/C4 diet although it could have also included minor contributions from marine protein. From a chronological point of view, it is important to notice that the consumption of marine protein can lead to age offsets in human radiocarbon measurements due to a likely lower ^{14}C content in marine foods when compared to the contemporaneous atmosphere. This effect, known as a marine radiocarbon reservoir effect, has a magnitude of c. 400 years for the mean ocean although there can be significant local variations. Thus, reported human radiocarbon dates reported in this study should be viewed as *terminus post quem*.

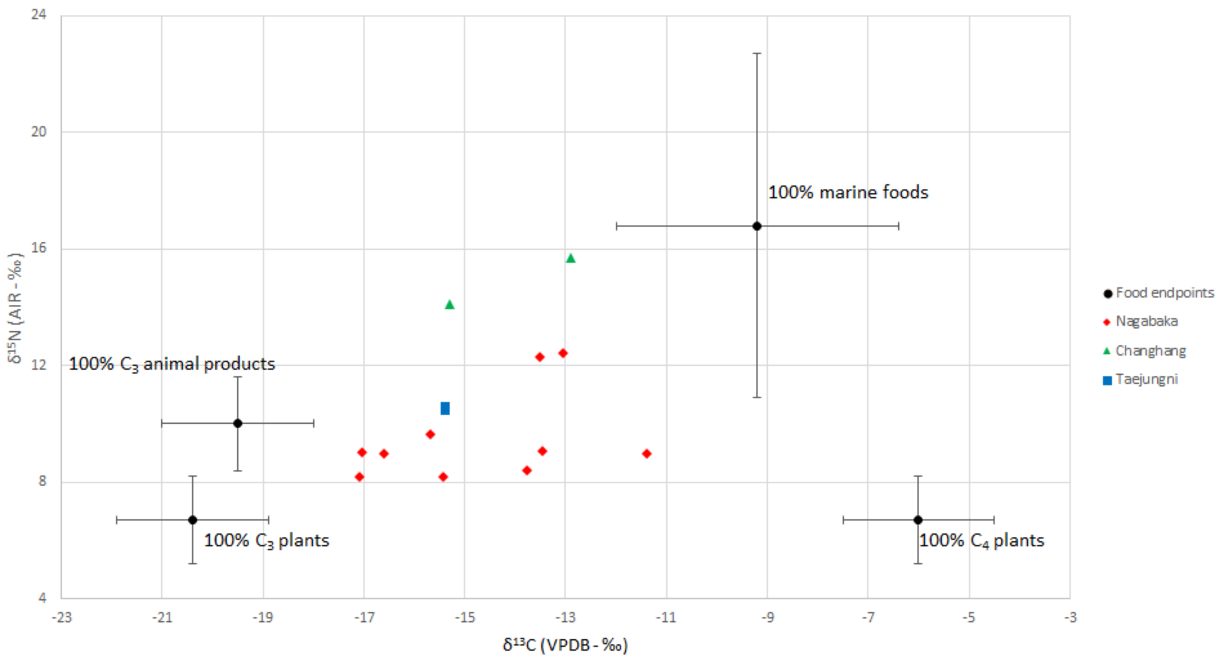

Fig. SI 15.1. Distribution of human isotopic values for the sites of Nagabaka, Changhang and Taejungni compared with food reference end points.

The result so the carbon stable isotope measurements on human tooth enamel are shown in Table SI 15.2. Here we can only compare the dietary contributions from C3 vs. C4/marine food sources, given previously defined reference endpoints (14.8±1.5‰ vs. -1.3±1.5‰, respectively). The results indicate a predominance of mixed diets, in agreement with results observed for bone collagen. Two individuals (49 and 51) had exclusive or almost exclusive C3 diets.

Table SI 15.2. Results of stable carbon isotope measurements in human bone enamel for the site of Nagabaka.

**ID**	$\delta^{13}C_{enamel}$ (‰ – VPDB)
41	-12.4
42	-10.0
43	-11.5
44	-9.0

45	-11.1
46	-9.0
47	-10.6
48	-9.7
49	-14.3
50	-8.9
51	-13.1
52	-12.3
53	-11.5
54	-9.8

§4. Summary

The isotopic results for both human bone collagen and tooth enamel showed that several individuals from Nagabaka, and also from Taejungni, had mixed C3/C4 diets which likely included millet and in a few cases millet consumption was comparatively high. Our study also identified two individuals from Nagabaka with high marine protein intakes although the two individuals from Changhang were those that most heavily relied on marine protein. Marine protein consumption can result in fictitiously older human radiocarbon dates. Thus, human radiocarbon dates, particularly for the pre-historic period, should be treated as *terminus post quem*.

References

[1] Schwarcz, H.P. & Schoeninger, M.J. in *Handbook of Environmental Isotope Geochemistry* (ed Baskaran, M.) 725-742 (Springer, 2012).

[2] Fernandes, R., Nadeau, M.J. & Grootes, P.M. Macronutrient-based model for dietary carbon routing in bone collagen and bioapatite. *Archaeol. Anthropol. Sci*. **4**, 291-301 (2012).

[3] Froehle, A.W., Kellner, C.M., & Schoeninger, M.J. Multivariate carbon and nitrogen stable isotope model for the reconstruction of prehistoric human diet. *Am. J. Phys. Anthropol*. **147**, 352-369 (2012).

[4] Hedges, R.E. & Reynard, L.M. Nitrogen isotopes and the trophic level of humans in archaeology. *J. Archaeol. Sci*. **34**, 1240-1251 (2007).

[5] Ehleringer, J.R. & Cerling, T.E., 2002. C3 and C4 photosynthesis. Encyclopedia of global environmental change, 2(4).

[6] Corr, L.T., Sealy, J.C., Horton, M.C. & Evershed, R.P. A novel marine dietary indicator utilising compound-specific bone collagen amino acid δ13C values of ancient humans. *J. Archaeol. Sci*. **32**, 321-330 (2005).

[7] Dewar, G. & Pfeiffer, S. Approaches to estimating marine protein in human collagen for radiocarbon date calibration. *Radiocarbon* **52**, 1611-1625 (2010).

[8] Lee-Thorp, J.A., Sealy, J.C. & van Der Merwe, N.J.. Stable carbon isotope ratio differences between bone collagen and bone apatite, and their relationship to diet. *J. Archaeol. Sci*. **16**, 585-599 (1989).

[9] Fernandes, R., Grootes, P., Nadeau, M.J. & Nehlich, O. Quantitative diet reconstruction of a Neolithic population using a Bayesian mixing model (FRUITS): the case study of Ostorf (Germany). *Am. J. Phys. Anthropol*. **158**, 325-340 (2015).

[10] O'Connell, T.C., Kneale, C.J., Tasevska, N. & Kuhnle, G.G.. The diet-body offset in human nitrogen isotopic values: A controlled dietary study. *Am. J. Phys. Anthropol*. **149**, 426-434 (2012).

[11] Cerling, T.E., Harris, J.M., MacFadden, B.J., Leakey, M.G., Quade, J., Eisenmann, V. & Ehleringer, J.R.. Global vegetation change through the Miocene/Pliocene boundary. *Nature* **389**(6647), 153-158 (1997).

[12] Yoneda, M., Suzuki, R., Shibata, Y., Morita, M., Sukegawa, T., Shigehara, N. & Akazawa, T. Isotopic evidence of inland-water fishing by a Jomon population excavated from the Boji site, Nagano, Japan. *J. Archaeol. Sci*. **31**, 97-107 (2004).

[13] Longin, R. New method of collagen extraction for radiocarbon dating. *Nature* **230**, 241-242 (1971).

[14] Brock, F., Higham, T. & Bronk Ramsey, C. Comments on the use of Ezee-filtersTM and ultrafilters at ORAU. *Radiocarbon* **55**, 211-212.

[15] Choy, K., An, D. & Richards, M.P. Stable isotopic analysis of human and faunal remains from the Incipient Chulmun (Neolithic) shell midden site of Ando Island, Korea. *J. Archaeol. Sci*. **39**, 2091-2097 (2012).

[16] Yamada, Y., Takigami, M., Sakamoto, M. & Fujio, S. Kankoku Pusan-shi Gadŏkto Janghang [Changhang] iseki shutsudo shinsekki jidai jinkotsu no nendaigakuteki chōsa ni tsuite. *Munmul (Korea Archaeol. Art Hist. Res. Instit. J.)* **9**, 151-158 (2019).
