# Supplementary material for: Triangulation supports agricultural spread of the Transeurasian languages
Source: Nature. 2021 Nov 10;599(7886):616–21. doi: 10.1038/s41586-021-04108-8 (PMC8612925; doi:10.1038/s41586-021-04108-8)
Supplement: Supplementary file 5 — This zipped file contains Supplementary Data Files 12–16; see Supplementary Information file for full descriptions. [file 41586_2021_4108_MOESM5_ESM.zip › 2021-02-02920E-s5/36_Eurasia3angle_synthesis_SI 16_qpAdm_REV07.07.pdf]

Supplementary Information 16 pAdm admixture modelling of Neolithic Ando from South Korea as a result of admixtu

OG: Mbuti.DG, Villabruna, Iran_Ganj_Dareh_Neolithic, Naxi.DG, Karitiana.DG, Onge.DG, Funadomari_Jomon

Test = Ando; Pright = OG

	Source Populations
Pval	Refs1
8,13E-01	Upper Xiajiadian
6,93E-01	Hanben
5,52E-01	Miaozigou
5,05E-01	Xianbei
4,99E-01	Devil's Gate
4,92E-01	Angangxi
4,61E-01	Houli
1,78E-01	Jalainur
1,74E-01	Fujian_LN
4,08E-02	Yangshao
3,03E-02	Hongshan
2,84E-02	Korean.DG
1,95E-02	Lower Xiajiadian
7,72E-03	Longshan
6,11E-03	Yumin
2,47E-03	Eastern Zhou
1,42E-03	Japanese.DG
1,80E-05	Fujian_EN
1,87E-225	Ikawazu Jomon
2,37E-260	Nagabaka late
0,00E+00	Rokutsu Jomon

Test = Ando; Pright = OG

	Source Populations		Ancestry Proportions	
Pval	Refs1	Refs2	C1	C2
9,56E-01	Ikawazu Jomon	Miaozigou	0,024	0,976
9,23E-01	Rokutsu Jomon	Houli	0,032	0,968
9,21E-01	Rokutsu Jomon	Upper Xiajiadian	0,027	0,973
8,61E-01	Ikawazu Jomon	Upper Xiajiadian	0,015	0,985
8,57E-01	Ikawazu Jomon	Houli	0,029	0,971
8,19E-01	Ikawazu Jomon	Xianbei	0,035	0,965
7,65E-01	Rokutsu Jomon	Miaozigou	0,067	0,933
7,62E-01	Rokutsu Jomon	Angangxi	0,112	0,888
7,11E-01	Ikawazu Jomon	Japanese.DG	-0,091	1,091
6,93E-01	Ikawazu Jomon	Devil's Gate	-0,017	1,017
6,60E-01	Rokutsu Jomon	Taiwan_Hanben	0,006	0,994
6,16E-01	Ikawazu Jomon	Lower Xiajiadian	0,053	0,947
6,16E-01	Ikawazu Jomon	Hongshan	0,045	0,955
5,17E-01	Rokutsu Jomon	Xianbei	0,045	0,955
5,02E-01	Ikawazu Jomon	Eastern Zhou	0,057	0,943
4,66E-01	Ikawazu Jomon	Yangshao	0,024	0,976
4,17E-01	Ikawazu Jomon	Jalainur	0,053	0,947
3,94E-01	Rokutsu Jomon	Eastern Zhou	0,067	0,933
3,88E-01	Rokutsu Jomon	Lower Xiajiadian	0,063	0,937
3,72E-01	Rokutsu Jomon	Korean.DG	0,052	0,948
3,65E-01	Ikawazu Jomon	Taiwan_Hanben	0	1
3,42E-01	Rokutsu Jomon	Hongshan	0,053	0,947

3,38E-01	Rokutsu Jomon	Yangshao	0,05	0,95
3,36E-01	Ikawazu Jomon	Angangxi	0,071	0,929
3,22E-01	Ikawazu Jomon	Korean.DG	0,039	0,961
3,06E-01	Rokutsu Jomon	Devil's Gate	0,001	0,999
2,51E-01	Rokutsu Jomon	Japanese.DG	-0,076	1,076
1,63E-01	Rokutsu Jomon	Fujian_LN	0,006	0,994
1,21E-01	Rokutsu Jomon	Yumin	0,067	0,933
8,42E-02	Rokutsu Jomon	Jalainur	0,019	0,981
8,25E-02	Ikawazu Jomon	Yumin	0,053	0,947
5,87E-02	Ikawazu Jomon	Longshan	0,023	0,977
3,12E-02	Rokutsu Jomon	Longshan	0,036	0,964
2,05E-02	Ikawazu Jomon	Fujian_LN	-0,018	1,018
2,50E-05	Rokutsu Jomon	Fujian_EN	-0,024	1,024
2,11E-06	Ikawazu Jomon	Fujian_EN	-0,03	1,03

ıre between lineages related to Jomon and mainland East Asians from the '1240k' datasets

Test = Ando; Pright = OG + Houli

Standard Errors			Source Populations		Ancestry Proportions		Standard Errors	
SE1	SE2	Pval	Refs1	Refs2	C1	C2	SE1	SE2
0,039	0,039	9,68E-01	Rokutsu Jomon	Upper Xiajiadian	0,023	0,977	0,026	0,026
0,019	0,019	9,36E-01	Ikawazu Jomon	Miaozigou	0,032	0,968	0,037	0,037
0,026	0,026	8,47E-01	Rokutsu Jomon	Miaozigou	0,069	0,931	0,033	0,033
0,030	0,030	7,86E-01	Ikawazu Jomon	Upper Xiajiadian	0,003	0,997	0,029	0,029
0,021	0,021	6,99E-01	Ikawazu Jomon	Hongshan	0,042	0,958	0,022	0,022
0,038	0,038	4,78E-01	Ikawazu Jomon	Devil's Gate	-0,022	1,022	0,023	0,023
0,034	0,034	4,12E-01	Rokutsu Jomon	Hongshan	0,05	0,95	0,019	0,019
0,063	0,063	3,98E-01	Ikawazu Jomon	Lower Xiajiadian	0,046	0,954	0,023	0,023
0,022	0,022	2,81E-01	Rokutsu Jomon	Angangxi	0,091	0,909	0,065	0,065
0,023	0,023	2,38E-01	Rokutsu Jomon	Devil's Gate	-0,005	1,005	0,020	0,020
0,017	0,017	2,32E-01	Rokutsu Jomon	Lower Xiajiadian	0,056	0,944	0,021	0,021
0,024	0,024	6,56E-02	Ikawazu Jomon	Jalainur	0,041	0,959	0,037	0,037
0,022	0,022	6,20E-02	Ikawazu Jomon	Angangxi	0,047	0,953	0,084	0,084
0,034	0,034	5,56E-02	Rokutsu Jomon	Yumin	0,057	0,943	0,023	0,023
0,019	0,019	4,85E-02	Ikawazu Jomon	Xianbei	0,005	0,995	0,038	0,038
0,021	0,021	4,19E-02	Rokutsu Jomon	Fujian_LN	-0,004	1,004	0,021	0,021
0,037	0,037	2,88E-02	Ikawazu Jomon	Yumin	0,04	0,96	0,026	0,026
0,017	0,017	2,37E-02	Rokutsu Jomon	Xianbei	0,021	0,979	0,034	0,034
0,021	0,021	1,74E-02	Rokutsu Jomon	Hanben	-0,006	1,006	0,017	0,017
0,018	0,018	8,68E-03	Ikawazu Jomon	Yangshao	0,01	0,99	0,021	0,021
0,019	0,019	8,18E-03	Ikawazu Jomon	Japanese.DG	-0,104	1,104	0,023	0,023
0,020	0,020	6,76E-03	Rokutsu Jomon	Yangshao	0,039	0,961	0,018	0,018

**0,018**	**0,018**	5,41E-03	Rokutsu Jomon	Jalainur	0,002	0,998	0,036	0,036
**0,082**	**0,082**	5,30E-03	Ikawazu Jomon	Longshan	0,015	0,985	0,019	0,019
**0,020**	**0,020**	4,76E-03	Rokutsu Jomon	Longshan	0,028	0,972	0,017	0,017
**0,021**	**0,021**	4,47E-03	Ikawazu Jomon	Fujian_LN	-0,033	1,033	0,025	0,025
**0,020**	**0,020**	3,71E-03	Ikawazu Jomon	Hanben	-0,014	1,014	0,019	0,019
**0,022**	**0,022**	3,62E-03	Rokutsu Jomon	Korean.DG	0,038	0,962	0,018	0,018
**0,023**	**0,023**	3,53E-03	Rokutsu Jomon	Japanese.DG	-0,088	1,088	0,020	0,020
**0,036**	**0,036**	3,04E-03	Rokutsu Jomon	Eastern Zhou	0,052	0,948	0,017	0,017
**0,026**	**0,026**	1,90E-03	Ikawazu Jomon	Korean.DG	0,025	0,975	0,020	0,020
**0,019**	**0,019**	1,67E-03	Ikawazu Jomon	Eastern Zhou	0,041	0,959	0,019	0,019
0,017	0,017	9,67E-08	Rokutsu Jomon	Fujian_EN	-0,045	1,045	0,024	0,024
0,025	0,025	3,79E-09	Ikawazu Jomon	Fujian_EN	-0,056	1,056	0,028	0,028
0,024	0,024							
0,027	0,027							

Test = Ando; Pright = OG + Jalainur

	Source Populations		Ancestry Proportions		Standard Errors	
Pval	Refs1	Refs2	C1	C2	SE1	SE2
9,28E-01	Ikawazu Jomon	Japanese.DG	-0,085	1,085	0,034	0,034
8,55E-01	Ikawazu Jomon	Xianbei	0,035	0,965	0,063	0,063
8,47E-01	Rokutsu Jomon	Eastern Zhou	0,057	0,943	0,027	0,027
8,41E-01	Ikawazu Jomon	Eastern Zhou	0,055	0,945	0,030	0,030
7,82E-01	Ikawazu Jomon	Yangshao	0,023	0,977	0,033	0,033
7,24E-01	Rokutsu Jomon	Hanben	0,006	0,994	0,026	0,026
7,12E-01	Rokutsu Jomon	Upper Xiajiadian	0,044	0,956	0,040	0,040
7,08E-01	Rokutsu Jomon	Korean.DG	0,049	0,951	0,028	0,028
6,49E-01	Ikawazu Jomon	Houli	0,034	0,966	0,033	0,033
6,39E-01	Ikawazu Jomon	Miaozigou	-0,02	1,02	0,059	0,059
6,07E-01	Rokutsu Jomon	Angangxi	0,113	0,887	0,095	0,095
6,07E-01	Ikawazu Jomon	Korean.DG	0,047	0,953	0,030	0,030
5,91E-01	Rokutsu Jomon	Fujian_LN	0,015	0,985	0,033	0,033
5,52E-01	Ikawazu Jomon	Devil's Gate	0,024	0,976	0,033	0,033
5,46E-01	Rokutsu Jomon	Yangshao	0,027	0,973	0,031	0,031
4,93E-01	Rokutsu Jomon	Japanese.DG	-0,094	1,094	0,032	0,032
4,85E-01	Ikawazu Jomon	Hongshan	0,065	0,935	0,034	0,034
4,29E-01	Ikawazu Jomon	Yumin	0,05	0,95	0,038	0,038
3,80E-01	Ikawazu Jomon	Hanben	0,018	0,982	0,029	0,029
3,60E-01	Ikawazu Jomon	Fujian_LN	0,021	0,979	0,038	0,038
3,46E-01	Rokutsu Jomon	Miaozigou	0,011	0,989	0,053	0,053
3,20E-01	Ikawazu Jomon	Lower Xiajiadian	0,05	0,95	0,036	0,036

3,12E-01	Ikawazu Jomon	Upper Xiajiadian	0,032	0,968	0,045	0,045
2,89E-01	Rokutsu Jomon	Houli	0,029	0,971	0,030	0,030
2,21E-01	Rokutsu Jomon	Lower Xiajiadian	0,04	0,96	0,033	0,033
2,11E-01	Ikawazu Jomon	Angangxi	0,115	0,885	0,164	0,164
1,95E-01	Rokutsu Jomon	Xianbei	0,021	0,979	0,058	0,058
1,87E-01	Ikawazu Jomon	Longshan	0,029	0,971	0,030	0,030
1,46E-01	Rokutsu Jomon	Hongshan	0,056	0,944	0,032	0,032
1,32E-01	Rokutsu Jomon	Longshan	0,033	0,967	0,028	0,028
1,20E-01	Rokutsu Jomon	Yumin	0,054	0,946	0,035	0,035
8,08E-02	Rokutsu Jomon	Devil's Gate	0,019	0,981	0,031	0,031
4,78E-02	Rokutsu Jomon	Fujian_EN	-0,01	1,01	0,035	0,035
1,89E-02	Ikawazu Jomon	Fujian_EN	0,001	0,999	0,040	0,040
