# Supplementary material for: Triangulation supports agricultural spread of the Transeurasian languages
Source: Nature. 2021 Nov 10;599(7886):616–21. doi: 10.1038/s41586-021-04108-8 (PMC8612925; doi:10.1038/s41586-021-04108-8)
Supplement: Supplementary file 6 — This zipped file contains Supplementary Data Files 17–20 and 22; see Supplementary Information file for full descriptions (Supplementary Data File 21 is hosted externally; see Supplementary Information file for links). [file 41586_2021_4108_MOESM6_ESM.zip › 2021-02-02920E-s6/38_Eurasia3angle_synthesis_SI 18_Bayesian linguistics_REV07.07.pdf]

Supplementary Information 18

Bayesian phylogenetic analysis of the linguistic dataset

Table SI 18.1 Time calibrations of internal nodes in the Transeurasian phylogenetic tree.

Node	Calibration
Japonic	150 BC +/- 175
Koreanic	1150 AD +/- 175
Tungusic	50 AD +/- 275
Mongolic	1200 AD +/- 50
Turkic	150 BC +/- 175

We informed the node ages of the individual language families under discussion by the age priors given in Table SI 18.1 above. We used the dates generally proposed in linguistic literature to support our estimates of the time intervals for each node.

Our chronological estimation of Proto-Japonic at 150 BC +/- 175 is in line with Lee and Hasegawa's (2011) Bayesian phylogenetic analysis, dating Proto-Japonic divergence at 182 BC. Classical linguistic dating methods estimate the break-up of Proto-Japonic before 500 BC: between 1 AD and 500 BC according to Hattori (1976: 43) and before 500 BC according to Frellesvig and Whitman (2008). However, the former dating is based on somewhat controversial lexicostatistic dating methods and the latter on the association of certain language changes with specific historical events, notably the introduction of Buddhism, which yields a terminus ante quem, a ceiling but not a floor for the time-depth of separation. Since we know that the primary branches Proto-Ryukyuan and Japanese remained in intensive contact after the break-up of Proto-Japonic until at least the 8th-9th Century AD (Pellard 2015), we expect more similarities between both languages than in case the connectivity would have been completely broken. Therefore, lexicostatistic dating methods tend to be biased towards more shallow break-up times than the real times. As a consequence, we consider the earlier part of the spectrum of previous time estimations to be more reliable than the later part and estimate Proto-Japonic at 150 BC +/- 175.

Since the Silla kingdom unified the Korean peninsula politically and linguistically in AD 668, erasing all pre-existing linguistic diversity, Proto-Koreanic is much shallower than Proto-Japonic. Modern Korean dialects show limited variation, most of which can be regarded as derived from Late Middle Korean (1446-1600) (Whitman 2011). An exception is the Cheju dialect, which preserves some phonological and lexical features that are thought to

preserve elements from Early Middle Korean (918-1446) (Shin et al. 2020). Therefore, we estimated Proto-Koreanic, as the ancestor of all contemporary Korean dialects at 1150 AD +/- 17.

Previous research on the time-depth of Proto-Tungusic largely confirm Janhunen's (2012: 8) proposal of an Iron Age dating (500 BC–500 AD), among others based on the reconstruction of the term 'iron' to Proto-Tungusic. This large interval of about one millennium has been reduced by Pevnov (2012: 32) estimating on the basis of a rough measure of mutual intelligibility that Proto-Tungusic could not be younger than two thousand years and by Robbeets who situated the break-up of Proto-Tungusic in the Han period (206 BC–220 AD) on the basis of ethnonym shifts. Recently, Oskolskaya et al. (2021) propose a dating around 450 BC using Bayesian methods. However, this date was calculated on the basis of a separation time of 1599 BC for the split between Jurchen and Manchu–Xibe, while a realistic separation time that aligns with the model should be at least 1185 BC. This difference of at least 400 years is expected to yield a later date for the calculated time depth of Proto-Tungusic than the real one. Therefore, we can safely estimate the Tungusic node age at 50 AD +/- 275.

Proto-Mongolic is nearly equivalent with the language spoken by the historical Mongols around the time of the Mongol Empire (1206-1368), which is documented in historical sources, written in several different scripts and collectively termed Middle Mongolian. As all contemporary varieties can be derived from Middle Mongolian, the depth of the Mongolic family is no more than 700 to 800 years (Rybatzki 2003, Robbeets et al. 2020) and we calibrate at 1200 AD +/- 50. Even if there were historical languages related to Mongolic spoken in Northeast China, such Khitan, the dynastic language of the Liao Empire (907-1125), the written traces of these groups are too fragmentary to be included in our dataset. By consequence, these languages cannot contribute to the calibration of the Proto-Mongolic node.

Based on evidence from contact linguistics, the earliest split between the two principal branches of Turkic, i.e. Bulgharic and Common Turkic, is usually dated to 500 BC-1 AD (Janhunen 2010). Turkic phylogenies relying on quantitative methods basically support the lower estimate. The following dates are obtained by lexicostatistic calculations: 300 BC (Tenišev et al. 2001), 120 BC (Mudrak 2009) and 1 AD (Dybo 2007). Savelyev and Robbeets (2019) date the split of Proto-Turkic to 124 BC on the basis of a Bayesian analysis. Our calibration of 150 BC +/- 175 thus lies within the bounds generally proposed as the time-depth of the Turkic language family.

Table SI 18.2 Comparison of fit of different models estimating the marginal likelihoods using nested sampling. Log likelihood estimates for the various models showing no overlap of 95% HPD ranges of best fitting model (PD Covarion with relaxed clock) and other models.

			95% HPD range log ML		
Substitution Model	Clock model	Mean log ML	lower	upper	SD log ML
CTMC	Strict	-34703.1	-34783.9	-34622.3	40.4
	Relaxed	-34224.1	-34308.5	-34139.7	42.2
Covarion	Strict	-33936.9	-33994.9	-33878.9	29
	Relaxed	-33529	-33591.2	-33466.8	31.1
PD Covarion	Strict	-33787.4	-33864.2	-33710.6	38.4
	Relaxed	-33169.1	-33232.9	-33105.3	31.9

References

Dybo, A. V. *Lingvisticheskije kontakty rannix tjurkov. Leksicheskij fond. Pratjurkskij period* [Language Contact of the Early Turks. The Lexical Stock. The Proto-Turkic Period]. (Vostochnaja literatura, 2007).

Frellesvig, B. & Whitman, J. in *Proto-Japanese: Issues and Prospects*. (ed Frellesvig, B. & Whitman, J.) 15–41 (Benjamins, 2008).

Hattori, S. in *Okinawagaku no reimei* [The dawn of Okinawan studies]. (ed. *Ifa Fuyū seitan hyakunen kinenkai*) 7–55 (Okinawa bunka kyokai, 1976).

Janhunen, Juha in *Anantam śāstram: Indological and Linguistic Studies in Honour of Bertil Tikkanen*. (ed Klaus Karttunen) 283–305 (Societas Orientalis Fennic, 2010).

Janhunen, Juha in *Recent Advances in Tungusic Linguistics*. (ed Malchukov, A. & Whaley, L.) 5–16 (Harrassowitz, 2012).

Lee, S., & Hasegawa, T. Bayesian phylogenetic analysis supports an agricultural origin of Japonic languages. *Proceedings of the Royal Society B*, *278*, 3662-3669 (2011).

Mudrak, O. A. *Klassifikacija tjurkskich jazykov i dialektov s pomosčju metodov glottochronologii na osnove voprosov po morfologii i istoričeskoj fonetike*. [Glottochronological classification of the Turkic languages and dialects based on a questionnaire on morphology and historical phonology] (RGGU, 2009).

Oskolskaya, S., Koile, E. & Robbeets, M. A Bayesian approach to the classification of Tungusic languages. *Diachronica* (2021)

Pellard, T. in *Handbook of the Ryukyu Languages: History, Structure, and Use* (ed. Heinrich, P., Miyara, S. & Shimoji, M.) 13–37 (de Gruyter, 2015).

Pevnov, A. M. in *Recent Advances in Tungusic Linguistics.* (ed Malchukov, A. & Whaley, L.) 17–40 (Harrassowitz, 2012).

Robbeets, M., Janhunen, J., Savelyev, A. & Korovina, E. in *The Oxford Guide to the Transeurasian Languages*. (ed Robbeets, M. & Savelyev, A.) 754-771 (Oxford University Press, 2020). DOI: 10.1093/oso/9780198804628.003.0044

Rybatzki, V. in *The Mongolic Languages* (ed Janhunen, J) 364–390 (Routledge, 2003).

Savelyev, A. & Robbeets, M. Bayesian phylolinguistics infers the internal structure and the time-depth of the Turkic language family. *J. Lang. Evol.* 1-15 (2019) doi: 10.1093/jole/lzz010

Shin, U., Kiaer, J. & Shin, J. in *The Oxford Guide to the Transeurasian Languages*. (ed Robbeets, M. & Savelyev, A.) 258-268 (Oxford University Press, 2020).

Tenishev, Ė. R. & Dybo, A. *Sravnitel'no-istoricheskaja grammatika tjurkskix jazykov* [Comparative-Historical Grammar of the Turkic Languages]. (Nauka, 2001)

Whitman, J. Northeast Asian Linguistic Ecology and the Advent of Rice Agriculture in Korea and Japan, *Rice* 4: 149–158 (2011).
