# Supplementary material for: Triangulation supports agricultural spread of the Transeurasian languages
Source: Nature. 2021 Nov 10;599(7886):616–21. doi: 10.1038/s41586-021-04108-8 (PMC8612925; doi:10.1038/s41586-021-04108-8)
Supplement: Supplementary file 6 — This zipped file contains Supplementary Data Files 17–20 and 22; see Supplementary Information file for full descriptions (Supplementary Data File 21 is hosted externally; see Supplementary Information file for links). [file 41586_2021_4108_MOESM6_ESM.zip › 2021-02-02920E-s6/39_Eurasia3angle_synthesis_SI 19_XML files_README_REV21.09.docx]

**Read-me with Supplementary Information 19 and 21**

SI 19 BEAST XML files specifying the models, priors, hyperpriors and settings used to run the analyses of the linguistic database

SI 21 BEAST XML files specifying the models, priors, hyperpriors and settings used to run the analyses of the archaeological database

# Model summary

## Substitution models:

cov = covarion

ctmc = continuous time Markov chain model

pd = pseudo Dollo model

pdcov = pseudo Dollo covarion model

## Clock models:

strict: strict clock

ucln: uncorrelated relaxed clock with log normal rate distribution

## Tree modes:

fbd: fossilised birth death

bsp: Bayesian skyline plot

# Files

## Language analyses:

./languages/tea254cov-strict-fbd-constrained.xml

./languages/tea254cov-ucln-fbd-constrained.xml

./languages/tea254ctmc-strict-fbd-constrained.xml

./languages/tea254ctmc-ucln-fbd-constrained.xml

./languages/tea254pdcov-strict-fbd-constrained.xml

./languages/tea254pdcov-ucln-fbd-constrained.xml

These analyses contain nested sampling analyses with a subchain-length of 500000 samples and 1 particle. Four instances were run for each of them in order to make sure the marginal likelihoods were within 2 standard deviations (both these quantities estimated by nested sampling) from each other, and were thus consistent internally, which indicates the subchain length is sufficiently long. Marginal likelihoods averaged over the 4 runs are presented in SI 18, which shows that the pseudo Dollo covarion model with uncorrelated relaxed clock fits the data best.

By changing the first line of the tea254pdcov-ucln-fbd-constrained.xml file, it becomes an adaptive coupled MCMC analysis with 4 chain (1 cold, 3 hot) with target acceptance probability of 0.1, resampled every 500 samples and a chain length of 100 million samples. Four instances were run to ensure the same posterior was obtained, which was checked in Tracer by inspecting overlap of marginal distributions of all parameters and other logged entities (prior, posterior, etc.), after removing 10% burn-in (though one chain took a bit longer to become stationary and a burn-in of 30% was required). We verified that all parameters and other logged entities obtained an effective sample (ESS) size of 200 or more.

Note that time is in units of centuries in the language analyses and is going forward, so a tip date of 20 is interpreted as a date at the year 2000 CE, a calibration with mean -1.5 indicates the calibration has a mean at the year 150 BCE.

## Phylogeography analysis for languages:

./languages/tea3geo.xml

This analysis could obtain convergence using a plain MCMC analysis with a chain length of 1000000, since the state space with a fixed tree (the MCC tree from inferred using tea254pdcov-ucln-fbd-constrained.xml) is much reduced. Four instance were run, and convergence verified in Tracer by removing 10% burn-in and making sure ESSs of all logged entities were at least 200 and marginal likelihoods matched.

## Archeological culture analyses:

./cultures/cov-strict-bsp.xml

./cultures/cov-ucln-bsp.xml

./cultures/ctmc-strict-bsp.xml

./cultures/ctmc-ucln-bsp.xml

./cultures/pd-strict-bsp.xml

./cultures/pd-ucln-bsp.xml

./cultures/pdcov-strict-bsp.xml

./cultures/pdcov-ucln-bbp.xml

./cultures/pdcov-ucln-bsp-tips.xml

./cultures/pdcov-ucln-bsp.xml

The above files contain adaptive coupled MCMC analysis with four chains and a chain length of 50 million samples, target acceptance probability of 0.234 resampled every 1000 samples. Four instances of were run and we verified using Tracer after removing 10% burn-in that marginal likelihood estimates of all logged entities matched, and ESSs were over 200.

Further, the 8 models were run with nested sampling with a sub-chain length of 500000 samples and 1 particle. By running four instances of each of them, it was verified by ascertaining log marginal likelihood estimates were within 2 standard deviations of each other, that the subchain length is sufficiently long. Marginal likelihood estimates are presented in SI 20. The XML files above are made into nested sampling runs by replacing the opening run element with <run id="mcmc" spec="beast.gss.NS" chainLength="1000000000" subChainLength="500000" preBurnin="0" particleCount="1" epsilon="1e-13">.

Note that time is in units of years and goes backward in time.
