# Supplementary material for: Triangulation supports agricultural spread of the Transeurasian languages
Source: Nature. 2021 Nov 10;599(7886):616–21. doi: 10.1038/s41586-021-04108-8 (PMC8612925; doi:10.1038/s41586-021-04108-8)
Supplement: Supplementary file 6 — This zipped file contains Supplementary Data Files 17–20 and 22; see Supplementary Information file for full descriptions (Supplementary Data File 21 is hosted externally; see Supplementary Information file for links). [file 41586_2021_4108_MOESM6_ESM.zip › 2021-02-02920E-s6/40_Eurasia3angle_synthesis_SI 20_Bayesian archaeology.pdf]

Supplementary Information 20

Bayesian Phylogenetics analysis of the cultural dataset

Comparison of fit of different models estimating the marginal likelihoods using nested sampling. Log likelihood estimates for the various models showing no overlap of 95% HPD ranges of best fitting model (PD Covarion with relaxed clock) and other models.

			95% HPD range log ML		
Substitution Model	Clock model	Mean log ML	lower	upper	SD log ML
Pseudo Dollo	Strict	-10,094.43	-10,165.48	-10,031.39	31.52
	Relaxed	-9,708.20	-9,799.76	-9,629.31	39.45
CTMC	Strict	-9,946.21	-10,028.88	-9,871.67	37.27
	Relaxed	-9,633.89	-9,729.86	-9,536.48	48.70
Covarion	Strict	-9,718.23	-9,789.31	-9,653.47	32.38
	Relaxed	-9,522.26	-9,608.85	-9,448.74	36.76
PD Covarion	Strict	-9,657.51	-9,735.43	-9,591.09	33.21
	Relaxed	-9,324.93	-9,424.57	-9,246.03	39.45

The pseudo Dollo covarion model estimates a mean tree age of 8746 (7638-10068 95% HPD) wrt to youngest tip (1800ya). The second best fitting model is the covarion model with relaxed clock, which estimates a much larger range but consistent with that of the PD Covarion range (7736-12660 95% HPD).
