# Supplementary material for: Triangulation supports agricultural spread of the Transeurasian languages
Source: Nature. 2021 Nov 10;599(7886):616–21. doi: 10.1038/s41586-021-04108-8 (PMC8612925; doi:10.1038/s41586-021-04108-8)
Supplement: Supplementary file 7 — This zipped file contains Supplementary Data Files 23–26; see Supplementary Information file for full descriptions. [file 41586_2021_4108_MOESM7_ESM.zip › 2021-02-02920E-s7/15_Eurasia3angle_synthesis_ SI 26_triangulation_REV21.09.docx]

**Supplementary Information 26**

Triangulation of linguistic, archaeological and genetic evidence with regard to spatiotemporal and subsistence patterns in Northeast Asia in the Neolithic and Bronze Age

|  | **Linguistics** | **Archaeology** | **Genetics** |
| --- | --- | --- | --- |
| **Entity 1** | **Proto-Transeurasian** | **(Pre-)Xiaohexi** | **Amur ancestry?** |
| **Location** | West Liao River | West Liao River | Baikal to Amur to southeastern steppe and Southern Primorye, probably including West Liao River and Korean Peninsula |
| **Time** | Pre-9181 BP  (5595 -12793 95% HPD) | 9000 BP- 8200 BP | Pre-7000 BP |
| **Diagnostics** | Cultivation, millets (no rice), spades, vegetal fermentation, durable wild food resources, sedentism, dogs, textile production (SI 5) | Transition to wetter climate, incipient broomcorn millet cultivation, onset of sedentism, chipped stone hoes  (Shelach-Lavi et al. 2019; Stevens et al. 2020; SI 7) | Amur-related hunter-gatherers from Baikal (7 000–6 000 BP; Damgaard et al. 2018), from eastern steppe east of West Liao (Yumin 8400–7800 BP; ED Fig. 8, 10)  from Amur (Jalainur 6500±30 BP; ED Fig. 8, 9 and; from Boisman (7 500- 6 000 BP; ED Fig. 8, 10) |
| **Entity 2** | **Proto-Altaic** | **Xinglongwa Zhaobaogou**  **Central groups** | **Amur ancestry?** |
| **Location** | West Liao River  Xar Moron-Laoha delta | West Liao River  Xar Moron-Laoha delta | Baikal to Amur to southeastern steppe and Southern Primorye, probably including West Liao River and Korean Peninsula |
| **Time** | 9181- 6811 BP  (4404-10166 95%HPD) | 8200–7400 BP  7400–6500 BP | 8000 - 6000 BP |
| **Diagnostics** | cultivation, millets (no rice), agricultural tools, fermentation, durable wild food resources, pig husbandry (no dairying, no pastoralism), dogs, textile production (SI 5) | Broomcorn and foxtail millet agriculture,  domesticated pigs, textile technology  (SI 7) | Our Xinglongwa samples (8 000- 7 500 BP; SI 11) failed, but decreasing Amur component from Hamin (Haminmangha 5700-5600 BP; 90%) to Hongshan (Banlashan 5400-5100 BP; 40%) to Lower Xiajiadian (Erdaojingzi 4000 BP 20%) suggests original Amur ancestry (ED Fig. 8, 9) |
| **Entity 3** | **Proto-Japano-Koreanic** | **Xinglongwa**  **Zhaobaogou**  **Peripheral coastal groups** | **Amur + Yellow River?** |
| Location | Coastal region west of Lower Liao River | Coastal region west of Lower Liao River | Coastal region west of Lower Liao River |
| Time | 9181-5458 BP  (3335-8024 95%HPD) | 8200–7400 BP  7400–6500 BP | 8000 - 6000 BP |
| Diagnostics | maritime vocabulary, cultivation, millets (no rice), agricultural tools, no pigs, fermentation, brewing, durable wild food resources, textile production, hemp, ramie (SI 5) | Broomcorn and foxtail millet agriculture,  textile technology  (SI 7) | Our Xinglongwa samples (8 000- 7 500 BP; SI 11) failed, but increasing Yellow River component from Hamin (Haminmangha 5700-5600 BP; 20%) to Hongshan (Banlashan 5400-5100 BP; 60%) suggests increasing Yellow River admixture towards this region |
| **Entity 4** | **Proto-Turkic** | **Zhukaigou**  **Ordos culture**  **Xiongnu** | **Amur+ western Eurasian ancestries** |
| Location | Ordos Plateau  Eastern Steppe | Ordos Plateau  Eastern Steppe | Ordos Plateau  Eastern Steppe |
| Time | 6811 BP- 2195 BP  (1882-2493 95% HPD) | 4000-3400 BP  2500 -1900 BP  2200-1900 BP | 2500-1100 BP |
| Diagnostics | Landscapes and fauna and flora from southern taiga-steppe zone,  millet agriculture, animal husbandry,  dairying, pastoralism (SI 5) | Millet agriculture, pig husbandry, dogs, pastoralism: sheep, cattle, horse | Xiongnu (2159-1852 BP), Türk (1398-1208 BP) and Old Uyghur (1206-1110 BP) are scattered but harbour Amur ancestry with increasing western Eurasian admixture over time (ED Fig. 8, 10). |
| **Entity 5** | **Proto-Tunguso-Mongolic** | **Hongshan** | **Amur + Yellow River** |
| Location | West Liao River | West Liao River | West Liao River |
| Time | 6811- 4491 BP  (2599-6373 95%HPD) | 6500–4900 BP | 6500–4900 BP |
| Diagnostics | Cultivation millets (no rice), agricultural tools, fermentation, pig husbandry (no dairying, no pastoralism), textile production (SI 5) | Broomcorn and foxtail millet agriculture, pig husbandry, textile technology | Increasing Yellow River component from Hamin (Haminmangha 5700-5600 BP; 20%) to Hongshan (Banlashan 5400-5100 BP; 60%) suggests increasing Yellow River admixture over time and towards Yellow River region. (ED Fig. 8, 9). |
| **Entity 6** | **Proto-Tungusic** | **Yinggeling**  **Yabuli**  **Zaisanovka**  **Krounovka** | **Amur + Amur** |
| Location | Amur-Ussuri-Khanka region | Amur-Ussuri-Khanka region | Amur-Ussuri-Khanka region |
| Time | 4491-1950 BP  (1499-2412 95%HPD) | 5500-4195 BP  4500-4000 BP  5200-3300 BP  2600-1800 BP | 4000 BP |
| Diagnostics | Agriculture, broomcorn millet, iron, pig husbandry, fauna and flora specific for Khanka-Ussuri region. Barley, horse, cattle and sheep are Iron Age borrowings (SI 5) | More broomcorn than foxtail millet agriculture, agricultural tools, pig husbandry, no horse cattle and sheep before Iron Age  (Li et al. 2020; SI 7) | Original hunter-gatherers from Southern Primorye (Devil’s gate (7750-7450 BP); Boisman (7500- 6000 BP) have Amur ancestry. Angangxi individuals (4000 BP) have Amur ancestry. Incoming farmers mixing with Primorye hunter-gatherers are thus expected to reflect Amur ancestry. (ED Fig. 8, 9). |
| **Entity 7** | **Proto-Mongolic** | **Lower Xiajiadian**  **Upper Xiadjadian Xianbei**  **Rouran**  **Shiwei**  **Khitan** | **Amur + Yellow River + Western Eurasian** |
| Location | West Liao, Nen River and Eastern Steppe | West Liao, Nen River and Eastern Steppe | West Liao, Nen River and Eastern Steppe |
| Time | 4491-939 BP  (871-1011 95%HPD) | 4900-3600 BP  3000–2600 BP  2158 –1715BP  1620-1395 BP  1564-744 BP  1034-825 BP | 4050-825 BP |
| Diagnostics | Agriculture, millets, pig husbandry, dogs, pastoralism: cattle, sheep, dairying, horse breeding (SI 4, 5) | Millet agriculture, dogs, animal husbandry: pigs, sheep, cattle with addition of nomadic pastoralism and horse breeding from Upper Xiajiadian | Both Upper and Lower Xiajiadian have Amur ancestry with Yellow River admixture, but the Erdaojingzi (4050–3344 BP) sample has more Yellow River influence than the Longtoushan (2851-2775 BP) sample, probably due to its greater proximity to the Yellow River region. Xianbei (Mogushan; 1900-1700 BP) fall closer to the Amur cluster than Shiwei (Gangga 1200 BP), Rouran (Khermen Tal 1500 BP), Khitan (Ulaan Kherem 1043–825 BP) due to lower western Eurasian gene flow (ED Fig. 8, 10). |
| **Entity 8** | **Proto-Koreanic** | **Middle-Late Chulmun**  **Mumun** | **Jomon + Amur+ Yellow River** |
| Location | Korean Peninsula | Korean Peninsula | Korean Peninsula |
| Time | 5458-975 BP  (528-1560 95%HPD) | 5500–3500 BP  3500–2300 BP | 8000– 2300 BP |
| Diagnostics | Inherited words for millet agriculture, textile production and agricultural tools in line with first migratory wave.  Recycling of millet agricultural vocabulary as rice agricultural vocabulary, wheat/barley borrowing, horse borrowing, silk borrowing (SI 4, 5) in line with later migratory waves. | In Middle-Late Chulmun millet agriculture, agricultural tools such as waisted chipped stone hoes, poor evidence for pigs, dogs. In Mumun addition rice, barley and wheat from Shandong-Liaodong. No evidence for domesticated animals, except dogs (SI 7). | Early Neolithic samples such as Changhang (6651-6304 BP), Ando (8300-5000 BP) and Yŏndaedo (7200-6600 BP) are modelled as Jomon with a high proportion of Hongshan ancestry, suggesting a first wave of Amur/Yellow River gene-flow from the West Liao region.  However, the Middle to Late Neolithic Yokchido sample (5500-4000 BP) shows 95% Jomon ancestry.  The Bronze Age Taejungni (2717-2491 BP  (SI 12) sample is modelled as an admixture of Jomon with Upper Xiajiadian ancestry, suggesting another wave of Amur/Yellow River gene-flow from the West Liao. |
| **Entity 9** | **Proto-Japonic** | **Mumun**  **Yayoi** | **Jomon+ Amur + Yellow River** |
| Location | Liaodong-Shandong to Korea to Kyushu and Japan | Korean Peninsula  Kyushu and Japanese Islands | Liaodong-Shandong to Korea to Kyushu and Japan |
| Time | 5458-2136 BP  (1499-2412 95%HPD) | 3500–2300 BP  3000–1700 BP | 4000–1700 BP |
| Diagnostics | Millet, rice, barley, wheat, agricultural tools, textile technology, bronzes | Mumun agricultural package including millet, rice, barley, wheat imported from Korean Peninsula from 3000 BP onwards, causing shift from Jomon (16 000-3000 BP) to Yayoi culture on Kyushu and spreading to Japanese Islands. | Pre-agricultural Jomon samples from Rokutsu (3950-3450 BP),  Funadomari (3960-3550 BP), Ikawazu (2720-2418 BP), Nagabaka (4026-3906 cal BP) and (2821-2794 cal. BP) show that Jomon ancestry stretched from Hokkaido as far south as Miyako Island on the Ryukyus. Yayoi samples from Antokudai (2400-1900 BP), Kuma-Nishioda (2400-1750 BP) and Shimomotoyama (1700 BP) are like Bronze Age Koreans, modeled as Jomon and Upper Xiajiadian, suggesting massive influx of migrants with mixed Amur-Yellow River ancestries from Korea into Japan in the Bronze Age. |

**References mentioned in SI 26**

Damgaard, P., Martiniano, R., Kamm, J., *et al*. The first horse herders and the impact of early bronze age steppe expansions into Asia. *Science* **360**, 6396, eaar7711, https://doi.org/10.1126/science.aar7711 (2018).

Li, T., Ning, C., Zhushchikhovskaya, I. S., *et al*. Millet agriculture dispersed from Northeast China to the Russian Far East: integrating archaeology, genetics and linguistics. *Archaeol. Res. Asia* **22**, 100177 (2020).

Shelach-Lavi, G., Teng, M., Goldsmith, Y. *et al* Sedentism and plant cultivation in northeast China emerged during affluent conditions. *PLoS ONE* 14, e0218751. (2019)

Stevens, C., Shelach-Lavi, G., Zhang, H., *et al*. A model for the domestication of Panicum miliaceum (common, proso or broomcorn millet) in China. *Veg. Hist. Archaeobot.* (2020) https://doi.org/10.1007/s00334-020-00804-z
