# Supplementary figures and images for: Triangulation supports agricultural spread of the Transeurasian languages
Source: Nature. 2021 Nov 10;599(7886):616–21. doi: 10.1038/s41586-021-04108-8 (PMC8612925; doi:10.1038/s41586-021-04108-8)

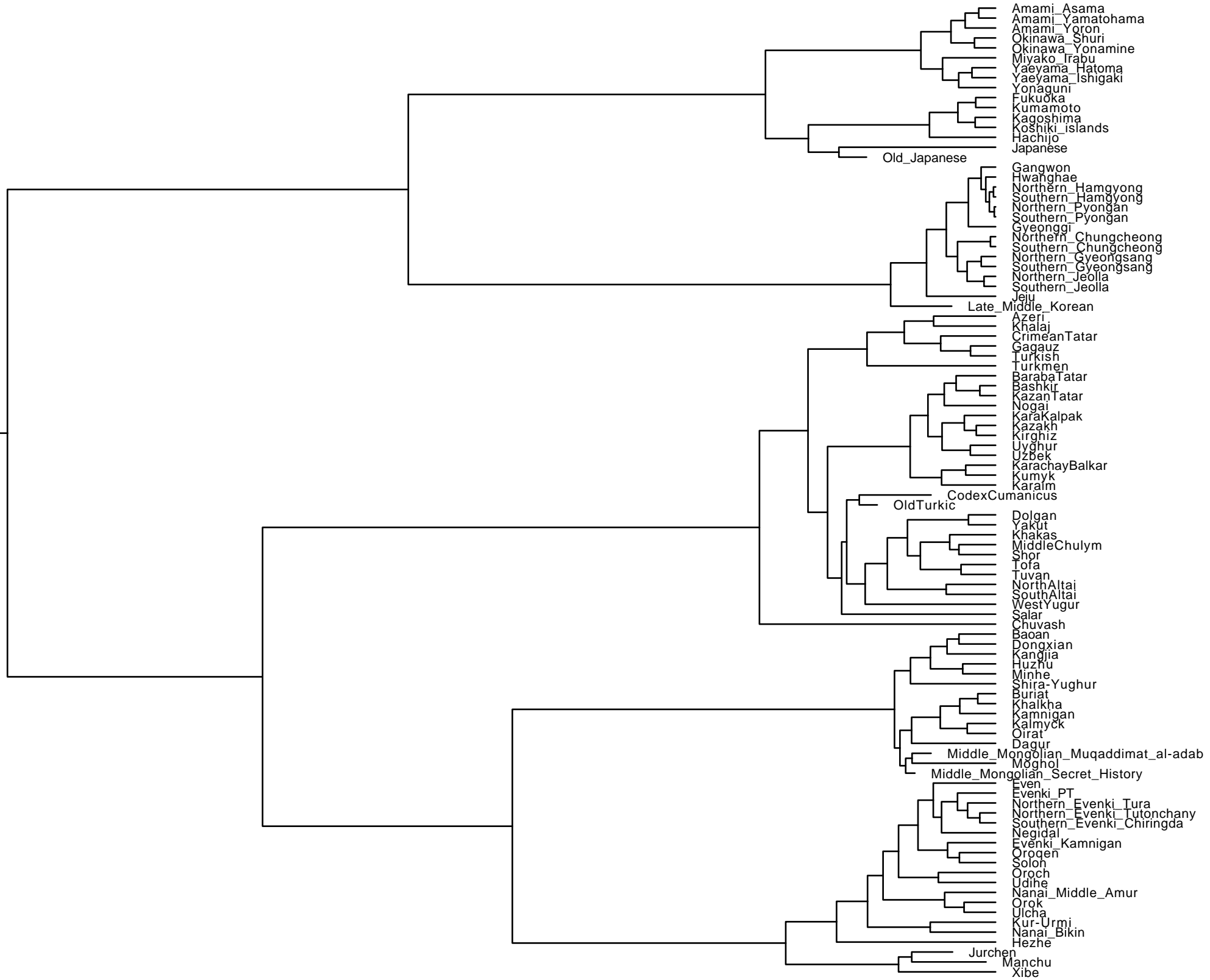

10.0

Supplement: Supplementary file 7 — This zipped file contains Supplementary Data Files 23–26; see Supplementary Information file for full descriptions. [file 41586_2021_4108_MOESM7_ESM.zip › 2021-02-02920E-s7/43_Eurasia3angle_synthesis_SI 24_language phylogeny.pdf]
