# Supplementary material for: Triangulation supports agricultural spread of the Transeurasian languages
Source: Nature. 2021 Nov 10;599(7886):616–21. doi: 10.1038/s41586-021-04108-8 (PMC8612925; doi:10.1038/s41586-021-04108-8)
Supplement: Supplementary file 7 — This zipped file contains Supplementary Data Files 23–26; see Supplementary Information file for full descriptions. [file 44_Eurasia3angle_synthesis_SI 25_cultural phylogeny.pdf › 2021-02-02920E-s7/44_Eurasia3angle_synthesis_SI 25_cultural phylogeny.pdf]

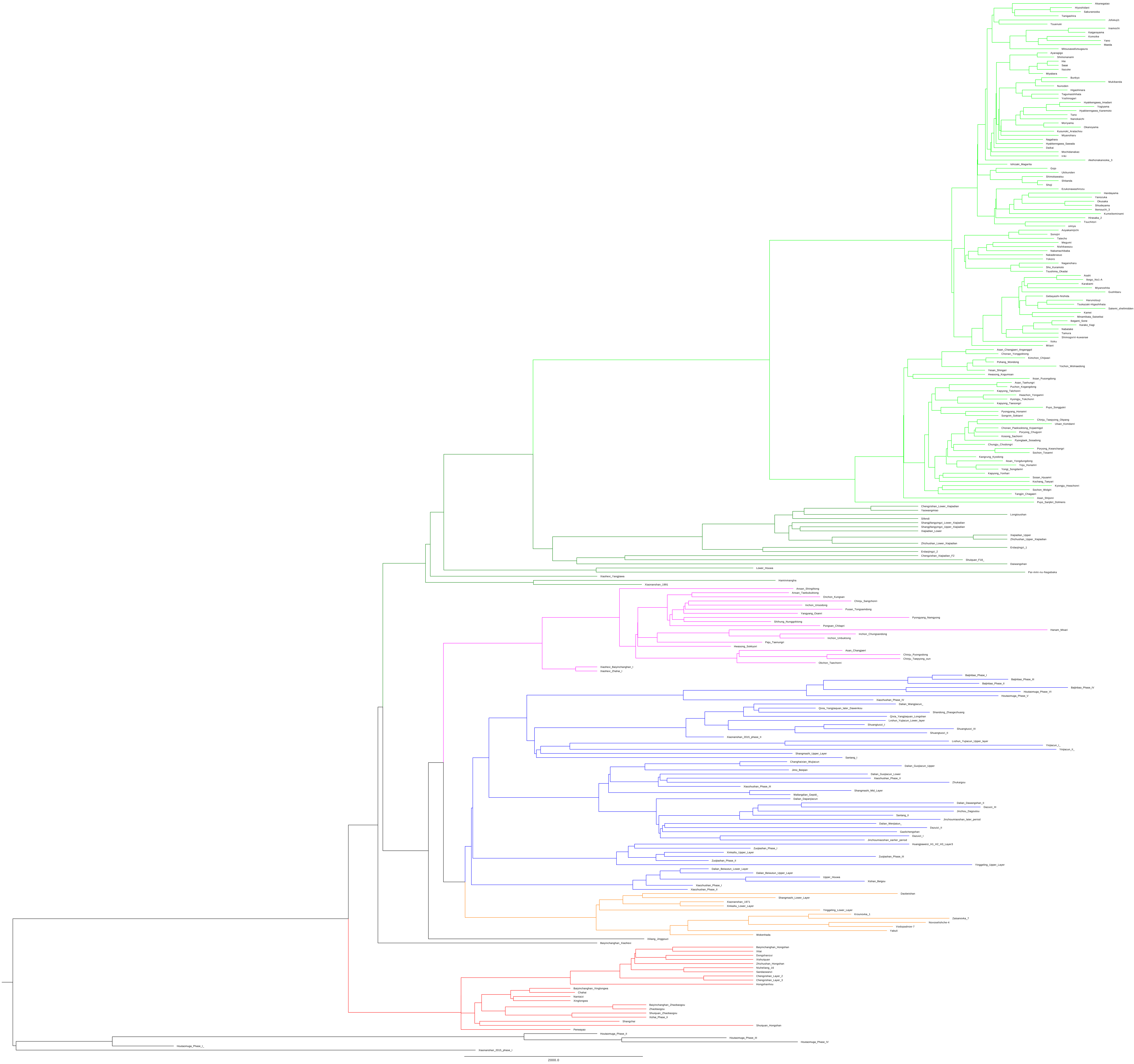
Hiyoshidani
Sakuranooka
Tanigashira
Jofukoji1
Tsuenuki
Inamochi
Kaigarayama
Komoke
Yano
Maeda
Mitsusawafutsugaura
Ayaragigo
Shimonanami
Hie
Saiai
Nazuke
Miyabara
Buriyo
Mukibanda
Nunoden
Higashinara
Tagumaishihata
Yoshinogari
Hyakkengawa_Imadani
Yogyama
Hyakkengawa_Kanemoto
Tano
Nanokaichi
Moriyama
Okanoyama
Kusunoki_Aratachou
Nagahara
Hyakkengawa_Sawada
Daikai
Iriki
Ishizaki_Magarita
Gojo
Ukikunden
Shimokawabu
Shitanda
Shoji
Handayama
Yanozuka
Okusaka
Shudeyama
Ikenouchi_3
Hirasaka_2
Tsuchitori
omiya
Sonojiri
Megumi
Nishikawazu
Nakamachibaba
Yokoro
Naganoharu
Sho_Kuramoto
Tsushima_Okadai
Asahi
Ikego_No1-A
Karakami
Miyanoshita
Gushiharu
Harunotsuji
Tsukazaki-Higashihata
Sakemi_shellmidden
Kamei
Minamikata_Saiseikai
Ikegami_Sone
Karako_Kagi
Nabatake
Tamura
Shimogorri-kuwanae
Itoku
Mitani
Asan_Changjaeri_Anganggol
Chonan_Yonggoktong
Kimchon_Chijwari
Pohang_Wondong
Yochon_Wolnaedong
Yesan_Shingari
Iksan_Pusongdong
Asan_Taehungri
Puchon_Kogangdong
Kapyong_Taichonri
Hwachon_Yonganri
Kyongju_Tokchonri
Kapyong_Taesongri
Puyo_Songgukri
Pyongyang_Honamri
Songnim_Soktanri
Chinju_Taepyong_Okpang
Ulsan_Komdanri
Chonan_Paeksoktong_Kojaemgol
Poryong_Chugyori
Kosong_Sachonri
Pyongtaek_Sosadong
Chungju_Chodongri
Poryong_Kwanchangri
Sochon_Tosanri
Kangnung_Kyodong
Iksan_Yongdungdong
Yoju_Hunamri
Yongi_Songdamri
Kapyong_Yonhari
Sosan_Hyuamri
Kochang_Taeyari
Kyongju_Hwachonri
Sochon_Wolgiri
Tangjin_Chagaeri
Asan_Shijonri
Puyo_Sanjikri_Dolmens
Chengzishan_Lower_Xiajiadian
Yaowangmiao
Longtoushan
Sifendi
Shangjifangyingzi_Lower_Xiajiadian
Shangjifangyingzi_Upper_Xiajiadian
Xiajiadian_Lower
Xiajiadian_Upper
Zhizhushan_Upper_Xiajiadian
Zhizhushan_Lower_Xiajiadian
Erdaojingzi_1
Erdaojingzi_2
Chengzishan_Xiajiadian_F2
Shuiquan_F18_
Dawangshan
Lower_Houwa
Xiaohexi_Yangjiawa
Haminmangha
Xiaonanshan_1991
Ansan_Shingiltong
Ansan_Taebubuktong
Onchon_Kungsan
Chinju_Sangchonri
Inchon_Unsodong
Pusan_Tongsamdong
Yangyang_Osanri
Pyongyang_Namgyong
Shihung_Nunggoktong
Pongsan_Chitapri
Hanam_Misari
Inchon_Chungsandong
Inchon_Unbuktong
Paju_Taenungri
Hwasong_Sokkyori
Asan_Changjaeri
Chinju_Pyongodong
Okchon_Taechonri
Xiaohexi_Baiyinchanghan_I
Xiaohexi_Zhahai_I
Baijinbao_Phase_I
Baijinbao_Phase_III
Baijinbao_Phase_II
Baijinbao_Phase_IV
Houtaomuga_Phase_VI
Houtaomuga_Phase_V
Xiaozhushan_Phase_IV
Dalian_Wangjiacun_
Qixia_Yangjiaquan_later_Dawenkou
Shandong_Zhaogezhuang
Qixia_Yangjiaquan_Longshan
Lvshun_Yujiacun_Lower_layer
Shuangtuozi_I
Shuangtuozi_III
Shuangtuozi_II
Xiaonanshan_2015_phase_II
Lvshun_Yujiacun_Upper_layer
Yinjiacun_I_
Yinjiacun_II_
Shangmashi_Upper_Layer
Santang_I
Changhaixian_Wujiacun
Dalian_Guojiacun_Upper
Jimo_Beiqian
Dalian_Guojiacun_Lower
Xiaozhushan_Phase_V
Zhukaigou
Xiaozhushan_Phase_III
Shangmashi_Mid_Layer
Wafangdian_Gepidi_
Dalian_Dapanjiacun
Dalian_Dawangshan_II
Dazuizi_III
Jinzhou_Dagoutou
Santang_II
Jinzhoumiaoshan_later_period
Dalian_Wenjiatun_
Dazuizi_II
Gaolichengshan
Dazuizi_I
Jinzhoumiaoshan_earlier_period
Huangjiaweizi_H1_H2_H3_Layer3
Zuojiashan_Phase_I
Xinkailiu_Upper_Layer
Zuojiashan_Phase_III
Zuojiashan_Phase_II
Yingqeling_Upper_Layer
Dalian_Beiwutun_Lower_Layer
Dalian_Beiwutun_Upper_Layer
Upper_Houwa
Xishan_Beigou
Xiaozhushan_Phase_I
Xiaozhushan_Phase_II
Daobeishan
Shangmashi_Lower_Layer
Xiaonanshan_1971
Xinkailiu_Lower_Layer
Yingqeling_Lower_Layer
Krounovka_1
Zaisanovka_7
Novoselishche-4
Vodopadnoe-7
Yabuli
Wokenhada
Baiyinchanghan_Xiaohexi
Baiyinchanghan_Hongshan
Xitai
Dongshanzui
Xishuiquan
Zhizhushan_Hongshan
Niuheliang_16
Sandaowanzi
Chengzishan_Layer_2
Chengzishan_Layer_3
Hongshanhou
Baiyinchanghan_Xinglongwa
Chahai
Nantaizi
Xinglongwa
Baiyinchanghan_Zhaobaogou
Zhaobaogou
Shuiquan_Zhaobaogou
Xizhai_Phase_II
Shangzhai
Shuiquan_Hongshan
Houtaomuga_Phase_II
Houtaomuga_Phase_III
Houtaomuga_Phase_IV
Houtaomuga_Phase_I_
Xiaonanshan_2015_phase_I
2000.0
